# Supplementary material for: Electrochemical cascade access to hetero[8]circulenes as potent organophotocatalysts for diverse C–X bond formations
Source: Nat Commun. 2025 Jul 1;16:5682. doi: 10.1038/s41467-025-60889-w (PMC12216941; doi:10.1038/s41467-025-60889-w)
Supplement: Supplementary file 1 — Supplementary Information [file 41467_2025_60889_MOESM1_ESM.pdf]

Supplementary Information

Electrochemical Cascade Access to Hetero[8]circulenes as Potent Organophotocatalysts for Diverse C–X Bond Formations

Ahmed S. Gabr,[1] Mohamed S. H. Salem*, [1,2] Md. Imrul Khalid,[1,3] Ryota Takahashi,[4] Yoshihiro Nishimoto,[4] Makoto Yasuda, [4] and Shinobu Takizawa*[1]

[1] *SANKEN, The University of Osaka, Mihogaoka, Ibaraki-shi, Osaka 567-0047, Japan.*

[2] *Pharmaceutical Organic Chemistry Department, Faculty of Pharmacy, Suez Canal University, Ismailia 41522, Egypt.*

[3] *Organic and Carbon Nanomaterials Unit, Okinawa Institute of Science and Technology Graduate University, 1919-1 Tancha, Onna-son, Kunigami-gun, Okinawa 904-0495, Japan*

[4] *Department of Applied Chemistry, Graduate School of Engineering, Osaka University, Suita, Osaka 565-0871, Japan.*

E-mail: mohamedsalem43@sanken.osaka-u.ac.jp; taki@sanken.osaka-u.ac.jp
Tel: +81-6-6879-8469

Contents

1. **Supplementary Method 1:** *general information* 6

2. **Supplementary Method 2:** *experimental procedures for the synthesis of starting materials* 8

2.1. Synthesis of 2,7-bis(trifluoromethylsulfonyloxy)naphthalene **S1** 8

2.2. Synthesis of 7-(aryl/alkylamino)naphthalen-2-yl trifluoromethanesulfonates **S2** 9

7-(*p*-Tolylamino)naphthalen-2-yl trifluoromethanesulfonate **S2a** 9

7-(Phenylamino)naphthalen-2-yl trifluoromethanesulfonate **S2b** 9

7-(*p*-Chlorophenylamino)naphthalen-2-yl trifluoromethanesulfonate **S2c** 10

7-(*o*-Anisylamino)naphthalen-2-yl trifluoromethanesulfonate **S2d** 10

7-(*p*-Bromophenylamino)naphthalen-2-yl trifluoromethanesulfonate **S2e** 11

7-(Benzylamino)naphthalen-2-yl trifluoromethanesulfonate **S2f** 11

7-(*n*-Hexylamino)naphthalen-2-yl trifluoromethanesulfonate **S2g** 11

2.3. Synthesis of *N*-phenylnaphthalen-2-amine derivatives **S4**, **S6**, **S8**, **S10**, and **S12** and *N*-phenylphenanthren-9-amine **S14** 13

6-Methoxy-*N*-phenylnaphthalen-2-amine **S4** 13

N-Phenyl-6-(thiophen-3-yl)naphthalen-2-amine **S6** 13

N,6-Diphenylnaphthalen-2-amine **S8** 14

6-(Phenylamino)-2-naphthonitrile **S10** 15

6-(Phenylamino)naphthalen-2-yl trifluoromethanesulfonate **S12** 15
N-Phenylphenanthren-9-amine **S14** 16
2.4. Synthesis of 10-hydroxy benzo[*c*]carbazol derivatives **1** 17
10-Hydroxy-7-(*p*-tolyl)-7*H*-benzo[*c*]carbazol-2-yl trifluoromethanesulfonate **1a** 17
10-Hydroxy-7-phenyl-7*H*-benzo[*c*]carbazol-2-yl trifluoromethanesulfonate **1b** 17
7-(*p*-Chlorophenyl)-10-hydroxy-7*H*-benzo[*c*]carbazol-2-yl trifluoromethanesulfonate **1c** 18
10-Hydroxy-7-(*o*-anisyl)-7*H*-benzo[*c*]carbazol-2-yl trifluoromethanesulfonate **1d** 18
7-(*p*-Bromophenyl)-10-hydroxy-7*H*-benzo[*c*]carbazol-2-yl trifluoromethanesulfonate **1e** 19
7-Benzyl-10-hydroxy-7*H*-benzo[*c*]carbazol-2-yl trifluoromethanesulfonate **1f** 19
7-*n*-Hexyl-10-hydroxy-*7H*-benzo[*c*]carbazol-2-yl trifluoromethanesulfonate **1g** 20
10-Hydroxy-8,9-dimethyl-7-(*p*-tolyl)-7*H*-benzo[*c*]carbazol-2-yl trifluoromethanesulfonate **1h** 20
10-Hydroxy-9-methyl-7-(*p*-tolyl)-7*H*-benzo[*c*]carbazol-2-yl trifluoromethanesulfonate **1i** 21
3-Methoxy-7-phenyl-7*H*-benzo[*c*]carbazol-10-ol **1k'** 21
7-Phenyl-3-(thiophen-3-yl)-7*H*-benzo[*c*]carbazol-10-ol **1l'** 22
3,7-Diphenyl-7*H*-benzo[*c*]carbazol-10-ol **1m'** 22
10-Hydroxy-7-phenyl-7*H*-benzo[*c*]carbazole-3-carbonitrile **1n'** 23
10-Hydroxy-7-phenyl-7*H*-benzo[*c*]carbazol-3-yl trifluoromethanesulfonate **1o'** 23
9-Phenyl-9*H*-dibenzo[*a,c*]carbazol-12-ol **1p'** 24
3. **Supplementary Method 3:** *optimization of electrochemical synthesis of dioxaza[8]circulenes* 25
Table S1. Screening of different solvents 25
Table S2. Screening of the electrochemical parameters (electrodes, electrolytes, current density) 25
Table S3. Screening of starting material concentration and ratio 26
4. **Supplementary Method 4:** *electrochemical cascade synthesis of dioxaza[8]circulenes* 27
General procedures 27
Method A: 27
Method B: 27
Method C: 27
Spectral data of dioxaza[8]circulenes 28
Dioxaza[8]circulene **3a** 28
Dioxaza[8]circulene **3b** 28
Dioxaza[8]circulene **3c** 29
Dioxaza[8]circulene **3d** 29
Dioxaza[8]circulene **3e** 30

Dioxaza[8]circulene **3f** 30
Dioxaza[8]circulene **3g** 31
Dioxaza[8]circulene **3h** 31
Dioxaza[8]circulene **3i** 32
Dioxaza[8]circulene **3j** 33
Dioxaza[8]circulene **3k** 33
Dioxaza[8]circulene **3l** 34
Dioxaza[8]circulene **3m** 34
Dioxaza[8]circulene **3n** 35
Dioxaza[8]circulene **3o** 35
Dioxaza[8]circulene **3p** 36
5. **Supplementary Method 5:** optimization and *general procedures for the one-pot synthesis of dioxaza[8]circulene from commercially available starting materials* 37
Optimization of the reaction conditions for step A 37
Optimization of the reaction conditions for step B 38
General procedures for the one-pot synthesis 40
6. **Supplementary Method 6:** *optimization of the organophotocatalytic arylation reaction towards diverse C-X bond formations* 41
Optimization of the reaction parameters using **6** and **7** as model substrates 41
Variation from standard conditions using **6** and **7** as model substrates 42
7. **Supplementary Method 7:** *general procedures for the photocatalytic arylation reactions towards diverse C-X bond formations* 43
General procedures 43
Spectral data 43
1-(4-(Thiophen-2-yl)phenyl)ethan-1-one **8a** 43
1-(4-(Furan-2-yl)phenyl)ethan-1-one **8b** 44
1-(4-(1-Methyl-1*H*-pyrrol-2-yl)phenyl)ethan-1-one **8c** 44
1-(2',4',6'-Trimethoxy-[1,1'-biphenyl]-4-yl)ethan-1-one **8d** 44
1-(2',4',6'-Trimethyl-[1,1'-biphenyl]-4-yl)ethan-1-one **8e** 45
2',4',6'-Trimethyl-[1,1'-biphenyl]-2-carbonitrile **8f** 45
2',4',6'-Trimethoxy-[1,1'-biphenyl]-2-carbonitrile **8g** 45
Ethyl 4-(1-methylpyrrol-2-yl)benzoate **8h** 46
4-(1-Methyl-1*H*-pyrrol-2-yl)benzaldehyde **8i** 46
2',4',6'-Trimethoxy-[1,1'-biphenyl]-4-carbaldehyde **8j** 46

2'-Methoxy-[1,1'-binaphthalene]-2-carbaldehyde **8k** 47
2-(2-Methoxynaphthalen-1-yl)benzaldehyde **8l** 47
9-(2-Methoxynaphthalen-1-yl)phenanthrene **8m** 47
2-(2,4,6-Trimethoxyphenyl)naphthalene **8n** 48
1-Methyl-2-(*p*-tolyl)-1*H*-pyrrole **8o** 48
4-(Furan-2-yl)benzaldehyde **8p** 48
4-(Phenylsulfonyl)benzaldehyde **8q** 49
1-(4-(Phenylsulfonyl)phenyl)ethan-1-one **8r** 49
2-(Phenylsulfonyl)benzonitrile **8s** 49
4-(Phenylsulfonyl)benzonitrile **8t** 50
4-((4-Chlorophenyl)sulfonyl)benzonitrile **8u** 50
2-(4,4,5,5-Tetramethyl-1,3,2-dioxaborolan-2-yl)benzonitrile **8v** 50
2,3',4,5',6-Pentamethoxy-1,1'-biphenyl **8w** 51
5-Fluoro-2',4',6'-trimethoxy-[1,1'-biphenyl]-2-carbonitrile **8x** 51
Diethyl (2-cyanophenyl)phosphonate **8y** 51
Diethyl *p*-tolylphosphonate **8z** 52
2',4',6'-Trimethoxy-3,5-dimethyl-[1,1'-biphenyl]-4-ol **8aa** 52
3-(1-Methyl-1*H*-pyrrol-2-yl)pyridine **8ab** 52
2-Bromo-1,3,5-trimethoxybenzene **8'** 53
1-(4-((2,2,6,6-Tetramethylpiperidin-1-yl)oxy)phenyl)ethan-1-one **9** 53
8. **Supplementary Note 1:** *plausible mechanism of electrochemical synthesis of dioxaza[8]circulenes* ... 54
9. **Supplementary Note 2:** *calculation of Faradic efficiency* 74
10. **Supplementary Note 3:** *monitoring the working electrode potential* 75
11. **Supplementary Note 4:** *plausible reaction mechanism of organo-photocatalytic arylation reaction* 76
Control experiments. 77
UV absorbance of hydroxycarbazole **1a** in pure chloroform. 78
UV absorbance of reaction mixture (4-bromoacetophenone **6** + thiophene **7**) in pure chloroform. 79
Energy profiles, in kcal/mol, for the organo-photocatalytic arylation reaction 80
Theoretical prediction of the pKa values of neutral radicals Int-II & Int-V 81
DFT-based prediction of the reducing power of radical anions for most substrates 82
Computational data for the suggested SET step 83
Light/Dark experiment of compound **8d** 84
Oxidation and reduction potentials of substrates in organophotocatalytic arylation reactions 85

Spin density of neutral and anionic radical intermediates in photocatalytic arylation mechanisms..... 88
Calculation of quantum efficiency 89
12. **Supplementary Note 5:** *X-ray crystallographic analysis of dioxaza[8]circulenes **3a** and **3b*** 91
13. **Supplementary Note 6:** *DFT calculations to study the structural and optoelectronic features of circulenes* 96
Optimization of basis sets for geometries of dioxaza[8]circulenes **3a** and **3b** 97
Molecular orbitals of **3a** and **3b** 101
Aromaticity of dioxaza[8]circulenes **3a** and **3b** 109
Aromaticity of dioxaza[8]circulene **3a** 109
Aromaticity of dioxaza[8]circulene **3b** 112
ACID plots of **3a** and **3b** 115
Time-dependent density-functional theory (TD-DFT) calculations 117
Summary of the TD-DFT calculation results 117
Analysis of the molecular contribution in the UV-Vis absorption pattern of **3a** 130
Theoretically calculated radiative rate constants $k_{f,calcd}$ 132
ETDM and MTDM of dioxaza[8]circulene 132
14. **Supplementary Note 7:** *X-ray crystal data* 139
Dioxaza[8]circulene **3a** 139
Dioxaza[8]circulene **3b** 141
1-(4-(Thiophen-2-yl)phenyl)ethan-1-one **8a** 143
15. **Supplementary Note 8:** *CV charts* 145
16. **Supplementary Note 9:** *calculation of band gap energy from UV–Vis absorption (Tauc Plots)* 150
17. **Supplementary Note 10:** *structural features of previously reported hetero[8]circulenes (Types I – IV) and hetero[9, 10]circulenes* 152
16.1. Alternating bond lengths of reported hetero[*n*]circulenes (comparative study) 152
16.2. Aromaticity of reported hetero[*n*]circulenes (comparative study) 157
18. **Supplementary Note 11:** *DFT calculations of the redox potentials* 165
Calculations of the excited reduction potential of dioxaza[8]circulene $E^{1/2}$ **($3a^{\bullet+}$/3a*)** 165
DFT calculation of solution phase electrochemical redox potential 165
19. **Supplemetary Note 12:** *NMR Spectra* 168
20. **Supplementary References** 287

1. **Supplementary Method 1:** *general information*

^{1}H-, and ^{13}C-NMR spectra were recorded with JEOL JMN ECS400 FT NMR, JNM ECA600 FT NMR or Bruker AVANCE II (^{1}H-NMR 400, 600 or 700 MHz, ^{13}C-NMR 100, 150, or 175 MHz) ^{1}H-NMR spectra are reported as follows: the chemical shift in ppm downfield of tetramethylsilane (TMS) and referenced to residual solvent peak ($CDCl_3$) at 7.26 ppm, or ($(CD_3)_2CO$) at 2.05 ppm, integration, multiplicities (s = singlet, d = doublet, t = triplet, q = quartet, m = multiplet), and coupling constants (Hz). ^{13}C-NMR spectra were reported in ppm relative to the central line of triplet for $CDCl_3$ at 77.16 ppm, or the central line of septet for ($(CD_3)_2CO$) at 29.84 ppm. ESI-MS spectra were obtained with JMS-T100LC (JEOL). FT-IR spectra were recorded on the JASCO FT-IR system (FT/IR4100). Thin-layer chromatography (TLC) analysis of reaction mixtures was performed using Merck silica-gel 60 F254 TLC plates and visualized under UV Column chromatography on SiO_2 was performed with Kanto silica-gel 60 (63-210 µm). Melting points were measured with melting point apparatus MP-S9 (Yanaco, Japan) and were uncorrected. UV–Vis absorption spectra were obtained on a Jasco V–670 spectrophotometers. The absolute PL quantum yields were measured using an Absolute PL Quantum Yield Measurement System (C9920–02, Hamamatsu Photonics [Hamamatsu, Japan]) in the air at room temperature. Differential pulse voltammetry (DPV) measurements were performed with an ALS-600A electrochemical analyzer using a glassy carbon working electrode, a Pt counter electrode, and an $Ag/AgNO_3$ reference electrode at room temperature in CH_3CN containing (0.1 M) n-Bu_4NClO_4 as the supporting electrolyte. Commercially available organic and inorganic compounds were used without further purification. The electrochemical reactions were performed in a 10 mL reaction vessel with two Pt electrodes (1.3 × 1.5 cm^2) connected to Cu wire (**Figure S1a, S1b**). The two electrodes are connected to the DC power supply (KIKUSUI PMX 35-1A) (**Figure S1c**). The reactions were carried out at rt, under air (1 atm.), and at a constant current. Constant current mode of electrolysis –in the case of our substrates- offers many advantages over constant potential mode; related to its easier setup and full conversion of the substrates. UV and visible light irradiations were performed with LED lamp (PER-AMP, Techno Sigma Co., Ltd.) (**Figure S1d**).

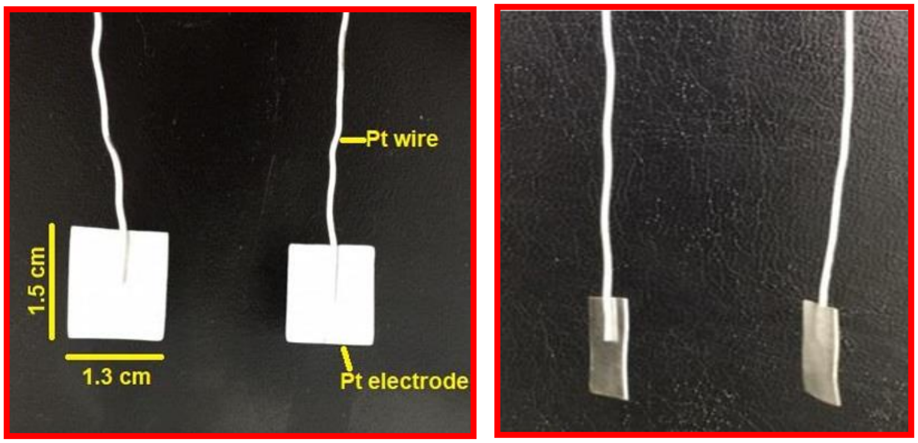

Figure S1a: Pt electrodes

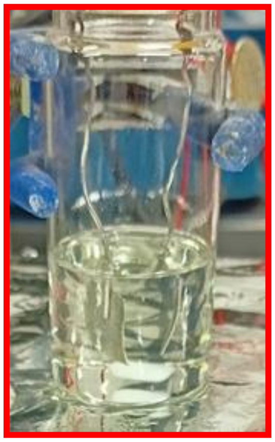

Figure S1b: Rx vessel

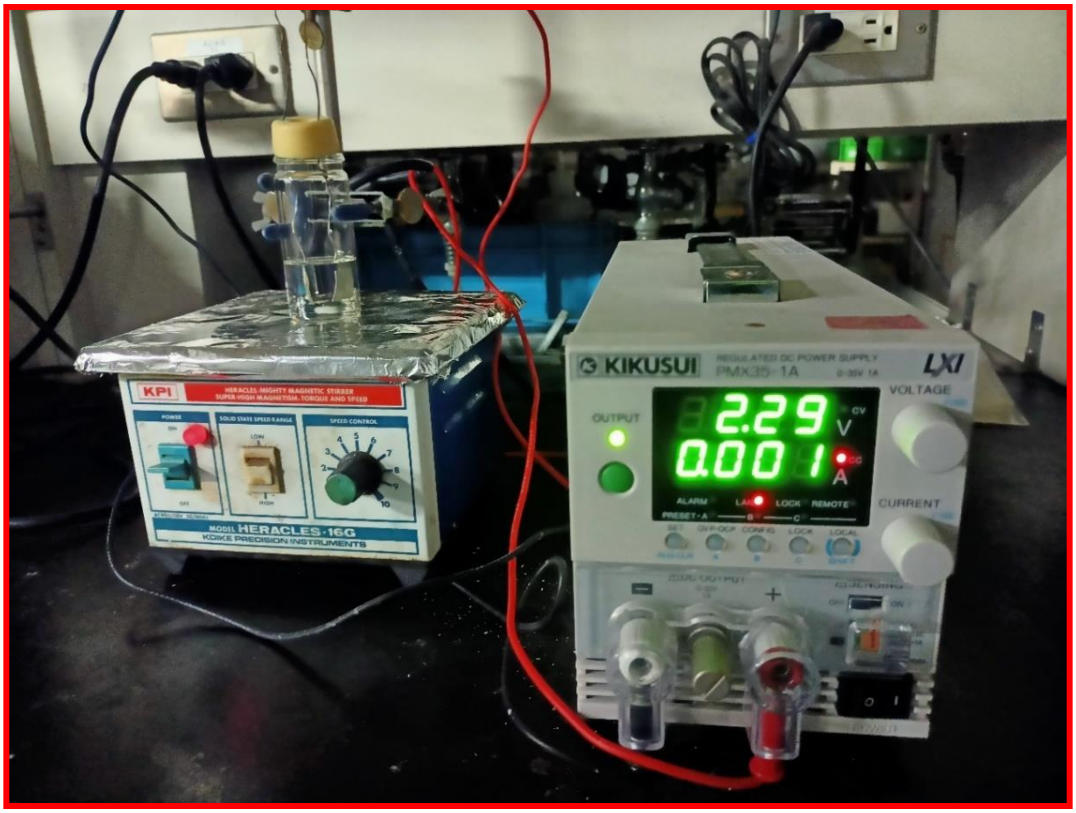

Figure S1c: DC power supply (KIKUSUI PMX 35.0-1.0A)

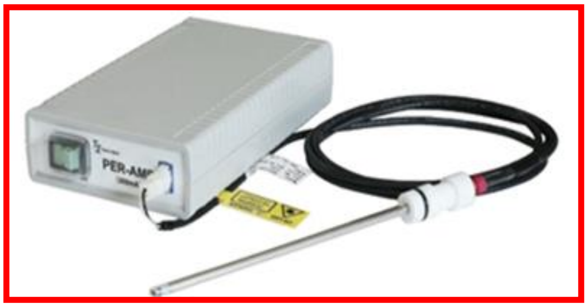

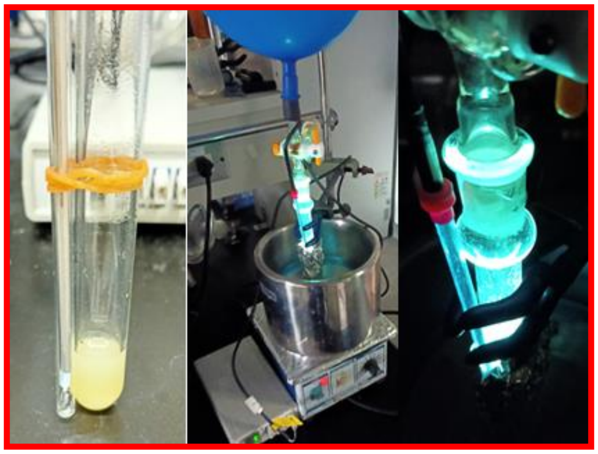

Figure S1d: Reaction setting with LED lamp Techno Sigma

2. **Supplementary Method 2:** *experimental procedures for the synthesis of starting materials*

2.1. Synthesis of 2,7-bis(trifluoromethylsulfonyloxy)naphthalene **S1**

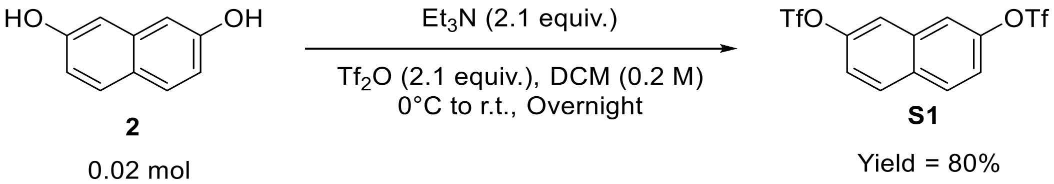

In a 250 mL round-bottom, double-neck flask, a solution of 2,7-dihydroxynaphthalene **2** (3.0 g, 0.02 mol) in CH_2Cl_2 (80.0 mL) and Et_3N (5.0 mL) was cooled to 0°C. Triflic anhydride (Tf_2O) (7.0 mL, 0.04 mol) was added dropwise over 5 minutes while stirring. The dark reaction mixture was then allowed to warm to room temperature and stirred overnight until completion, as confirmed by TLC. Subsequently, H_2O (10.0 mL) and CH_2Cl_2 (10.0 mL) were added to the mixture, and the aqueous phase was extracted with CH_2Cl_2. The combined organic phase was dried over MgSO4 and concentrated *in vacuo*. The resulting dark brown oil was purified by column chromatography (*n*-hexane/EtOAc = 20/1) to yield 2,7-bis(trifluoromethylsulfonyloxy)naphthalene **S1** as a colorless liquid, which solidified under vacuum to a pale white solid (6.0 g, 80% yield). The obtained spectra matched the literature[1].

^{1}H-NMR (400 MHz, $CDCl_3$) δ 7.97 (d, *J* = 9.2 Hz, 2H), 7.80 (d, *J* = 2.3 Hz, 2H), 7.47 (dd, *J* = 8.9, 2.5 Hz, 2H);
^{13}C-NMR (100 MHz, $CDCl_3$) δ 148.37, 133.70, 131.38, 130.93, 121.22, 119.60, 118.92 (q, *J* = 320.13 Hz).

2.2. Synthesis of 7-(aryl/alkylamino)naphthalen-2-yl trifluoromethanesulfonates S2

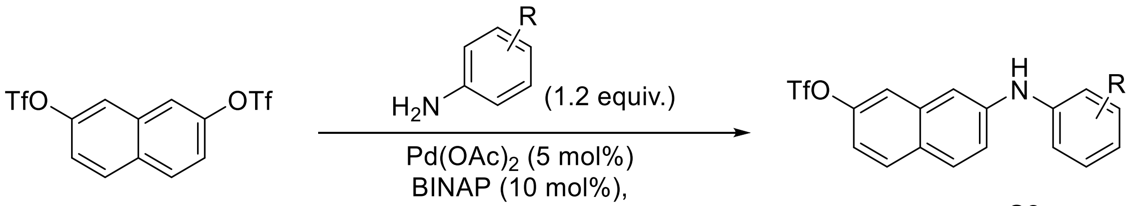

S1
3.5 mmol

S2

7-(*p*-Tolylamino)naphthalen-2-yl trifluoromethanesulfonate S2a

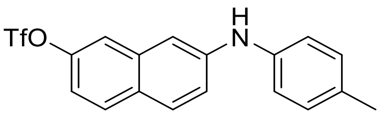

In a 100 mL round-bottom, double-neck flask under a nitrogen atmosphere, a solution of compound **S1** (1.5 g, 3.5 mmol) in dry toluene (20.0 mL), along with $Pd(OAc)_2$ (40.0 mg, 0.18 mmol), BINAP (220.0 mg, 0.35 mmol), and Cs_2CO_3 (1.4 g, 4.2 mmol), was stirred at 60 °C. After 10 minutes, *p*-toluidine (450.0 mg, 4.2 mmol) in dry toluene (15.0 mL) was added dropwise over 3 minutes while stirring, then the temperature was raised to 85 °C and maintained for 4 hours until the reaction was completed, as confirmed by TLC. The reaction mixture was then quenched with water, extracted with ethyl acetate (3 times), dried over anhydrous Na_2SO_4, and concentrated under reduced pressure. The residue was purified by column chromatography on silica gel (*n*-hexane/ethyl acetate = 20/1) to yield product **S2a** as a brownish yellow liquid, which solidified under vacuum (1.1 g, 82% yield).

m.p. 94–95 °C; **^{1}H-NMR** (400 MHz, $CDCl_3$) δ 7.76 (d, *J* = 8.7 Hz, 1H), 7.72 (d, *J* = 8.7 Hz, 1H), 7.48 (d, *J* = 2.3 Hz, 1H), 7.29 (d, *J* = 2.3 Hz, 1H), 7.18–7.21 (m, 3H), 7.12–7.15 (m, 3H), 5.89 (s, 1H), 2.38 (s, 3H); **^{13}C-NMR** (100 MHz, $CDCl_3$) δ 148.02, 143.82, 138.90, 135.21, 132.82, 130.28, 130.22, 129.28, 127.45, 120.79, 120.28, 118.94 (q, *J* = 324.92 Hz), 117.48, 116.21, 108.47, 20.93; **HRMS** (APCI) calcd for $[M + H]^+$ 382.0719, found: 382.0712; **IR** (KBr) 3413, 3057, 3030, 2923, 2865, 1881, 1630, 1513, 1212, 888 cm^{-1}.

7-(Phenylamino)naphthalen-2-yl trifluoromethanesulfonate S2b

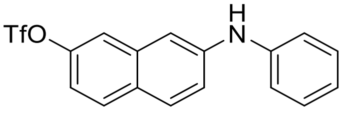

In a 100 mL round-bottom, double-neck flask under a nitrogen atmosphere, a solution of compound **S1** (1.5 g, 3.5 mmol) in dry toluene (35.0 mL), $Pd(OAc)_2$ (40.0 mg, 0.18 mmol), BINAP (220.0 mg, 0.35 mmol), and Cs_2CO_3 (1.4 g, 4.2 mmol) was stirred at 60 °C. After 10 minutes, aniline (391.1 mg, 4.2 mmol) was added dropwise while stirring. The temperature was then raised to 85 °C and maintained for about 4 hours until the reaction was completed, as confirmed by TLC. The reaction mixture was quenched with water,

extracted with ethyl acetate (3 times), dried over anhydrous Na_2SO_4, and concentrated under reduced pressure. The residue was purified by column chromatography on silica gel (*n*-hexane/ethyl acetate = 20/1) to yield product **S2b** as a brown solid (1.086 g, 84% yield).

m.p. 73–74 °C; **^{1}H-NMR** (400 MHz, $CDCl_3$) *δ* 7.76 (d, *J* = 9.2 Hz, 1H), 7.72 (d, *J* = 8.7 Hz, 1H), 7.52 (d, *J* = 2.3 Hz, 1H), 7.40 (t, *J* = 8.0 Hz, 2H), 7.36 (d, *J* = 1.8 Hz, 1H), 7.21–7.23 (m, 3H), 7.18 (dd, *J* = 8.9, 2.5 Hz, 1H), 7.12 (t, *J* = 7.6 Hz, 1H), 5.99 (s, 1H); **^{13}C-NMR** (100 MHz, $CDCl_3$) *δ* 147.94, 142.96, 141.75, 135.05, 130.27, 129.62, 129.24, 127.66, 122.60, 120.60, 119.58, 118.93 (q, *J* = 321.09 Hz), 117.56, 116.39, 109.45; **HRMS** (APCI) calcd for $[M + H]^+$ 368.0563, found:368.0557; **IR** (KBr) 3408, 3088, 2318, 1631, 1600, 1507, 1420, 1308, 1137, 894 cm^{-1}.

7-(*p*-Chlorophenylamino)naphthalen-2-yl trifluoromethanesulfonate S2c

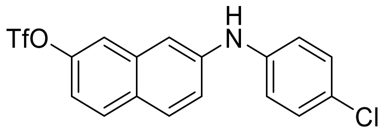

Following the same procedures as **S2a**, the residue was purified by column chromatography on silica gel (*n*-hexane/ethyl acetate = 20/1) to yield product **S2c** as a brown solid, (400 mg, 28% yield).

m.p. 63–64 °C; **^{1}H-NMR** (600 MHz, $CDCl_3$) *δ* 7.77 (d, *J* = 8.9 Hz, 1H), 7.75 (d, *J* = 8.9 Hz, 1H), 7.51 (s, 1H), 7.29–7.32 (m, 3H), 7.20 (dd, *J* = 8.9, 2.1 Hz, 1H), 7.16–7.18 (m, 1H), 7.10–7.12 (m, 2H), 5.96 (s, 1H); **^{13}C-NMR** (150 MHz, $CDCl_3$) *δ* 148.01, 142.54, 140.48, 134.99, 130.36, 129.61, 129.46, 127.89, 127.26, 120.67, 120.60, 118.91 (q, *J* = 320.78 Hz), 117.66, 116.79, 110.02; **HRMS** (APCI) calcd for $[M + H]^+$ 402.0173, found: 402.0168; **IR** (KBr) 3423, 3059, 2920, 1890, 1631, 1496, 1300, 1206, 1139, 767 cm^{-1}.

7-(*o*-Anisylamino)naphthalen-2-yl trifluoromethanesulfonate S2d

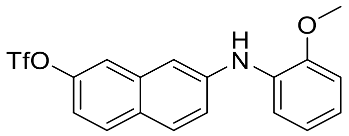

In a 100 mL round-bottom, double-neck flask under a nitrogen atmosphere, a solution of compound **S1** (1.5 g, 3.5 mmol) in dry toluene (35.0 mL), $Pd(OAc)_2$ (40.0 mg, 0.18 mmol), BINAP (220.0 mg, 0.35 mmol), and Cs_2CO_3 (1.4 g, 4.2 mmol) was stirred at 60 °C. After 10 minutes, 2-methoxyaniline (520.0 mg, 4.2 mmol) was added dropwise while stirring. The temperature was then raised to 85 °C and maintained overnight until the reaction was completed, as confirmed by TLC. The reaction mixture was quenched with water, extracted with ethyl acetate (3 times), dried over anhydrous Na_2SO_4, and concentrated under reduced pressure. The residue was purified by column chromatography on silica gel (toluene/$CHCl_3$ = 5/1) to yield the product **S2d** as a brown solid (730 mg, 52% yield).

m.p. 86–87 °C; **^{1}H-NMR** (400 MHz, $CDCl_3$) δ 7.76 (t, J = 9.4 Hz, 2H), 7.55 (d, J = 2.3 Hz, 1H), 7.49–7.52 (m, 2H), 7.32 (dd, J = 8.7, 2.3 Hz, 1H), 7.17 (dd, J = 9.2, 2.3 Hz, 1H), 6.97–7.05 (m, 3H), 6.30 (s, 1H), 3.92 (s, 3H); **^{13}C-NMR** (100 MHz, $CDCl_3$) δ 149.25, 147.94, 142.52, 135.07, 131.39, 130.24, 129.16, 127.78, 121.74, 121.30, 120.90, 118.92 (q, J = 321.09 Hz), 117.65, 116.82, 116.47, 110.90, 109.95, 55.66; **HRMS** (APCI) calcd for $[M + H]^+$ 398.0668, found:398.0662; **IR** (KBr) 3390, 3064, 2969, 2941, 2843, 1632, 1523, 1218, 1141, 888 cm^{-1}.

7-(*p*-Bromophenylamino)naphthalen-2-yl trifluoromethanesulfonate **S2e**

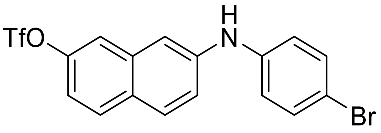

Following the same procedures as **S2a**, the residue was purified by column chromatography on silica gel (*n*-hexane/ethyl acetate = 20/1) to yield product **S2e** as a green solid, (1.3 g, 80% yield).

m.p. 50–51°C; **^{1}H-NMR** (400 MHz, $CDCl_3$) δ 7.75–7.80 (m, 2H), 7.52 (d, J = 2.3 Hz, 1H), 7.44 (d, J = 8.7 Hz, 2H), 7.35 (d, J = 2.3 Hz, 1H), 7.22 (dd, J = 9.2, 2.3 Hz, 1H), 7.17 (dd, J = 8.9, 2.5 Hz, 1H), 7.07 (d, J = 8.7 Hz, 2H), 5.35 (s, 1H); **^{13}C-NMR** (100 MHz, $CDCl_3$) δ 148.08, 142.39, 141.05, 135.03, 132.61, 130.40, 129.54, 128.02, 120.98, 120.73, 118.93 (q, J = 321.09 Hz), 117.76, 117.00, 114.66, 110.41; **HRMS** (APCI) calcd for $[M + H]^+$ 445.9668, found:445.9663; **IR** (KBr) 3421, 3064, 1887, 1633, 1589, 1414, 1210, 1141, 1105, 818 cm^{-1}.

7-(Benzylamino)naphthalen-2-yl trifluoromethanesulfonate **S2f**

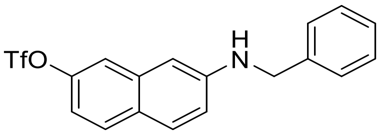

Following the same procedures as **S2d**, the residue was purified by column chromatography on silica gel (*n*-hexane/ethyl acetate = 30/1) to yield product **S2f** as pale yellow solid, (611mg, 45% yield).

m.p. 78–79 °C; **^{1}H-NMR** (600 MHz, $CDCl_3$) δ 7.71 (d, J = 8.9 Hz, 1H), 7.65 (d, J = 8.9 Hz, 1H), 7.46 (s, 1H), 7.37–7.42 (m, 4H), 7.31 (t, J = 7.2 Hz, 1H), 7.06 (dd, J = 8.6, 1.7 Hz, 1H), 6.97 (dd, J = 8.3, 1.4 Hz, 1H), 6.81 (s, 1H), 4.59 (s, 1H), 4.44 (s, 2H); **^{13}C-NMR** (150 MHz, $CDCl_3$) δ 148.10, 146.87, 138.50, 135.66, 130.28, 129.11, 128.96, 127.71, 126.55, 119.22, 118.94 (q, J = 320.78 Hz), 117.25, 115.38, 104.52, 48.34 (one carbon overlapped); **HRMS** (APCI) calcd for $[M + H]^+$ 382.0719, found:382.0710; **IR** (KBr) 3450, 3060, 2924, 1631, 1528, 1415, 1205, 1104, 954, 836 cm^{-1}.

7-(*n*-Hexylamino)naphthalen-2-yl trifluoromethanesulfonate **S2g**

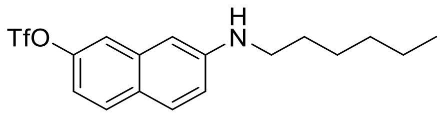

Following the same procedures as **S2d**, the residue was purified by column chromatography on silica gel (toluene/$CHCl_3$ = 5/1) to yield product **S2g** as brown solid, (330 mg, 25 % yield).

m.p. 33–34 °C; **^{1}H-NMR** (400 MHz, $CDCl_3$) δ 7.69 (d, J = 8.7 Hz, 1H), 7.62 (d, J = 9.2 Hz, 1H), 7.50 (d, J = 2.7 Hz, 1H), 7.04 (dd, J = 8.9, 2.5 Hz, 1H), 6.90 (dd, J = 8.7, 2.3 Hz, 1H), 6.75 (d, J = 2.3 Hz, 1H), 3.94 (s, 1H), 3.20 (t, J = 7.1 Hz, 2H), 1.69 (quintet, J = 7.3 Hz, 2H), 1.33–1.49 (m, 6H), 0.94 (t, J = 6.9 Hz, 3H); **^{13}C-NMR** (100 MHz, $CDCl_3$) δ 148.09, 147.46, 135.82, 130.23, 128.91, 126.18, 119.28, 118.94 (q, J = 321.09 Hz), 117.00, 114.87, 103.53, 43.84, 31.74, 29.32, 26.99, 22.76, 14.17; **HRMS** (APCI) calcd for $[M + H]^+$ 376.1189, found 376.1186; **IR** (KBr) 3361, 2930, 2859, 1628, 1462, 1402, 1247, 1207, 1138, 833 cm^{-1}.

2.3. Synthesis of *N*-phenylnaphthalen-2-amine derivatives **S4**, **S6**, **S8**, **S10**, and **S12** and *N*-phenylphenanthren-9-amine **S14**

6-Methoxy-*N*-phenylnaphthalen-2-amine **S4**

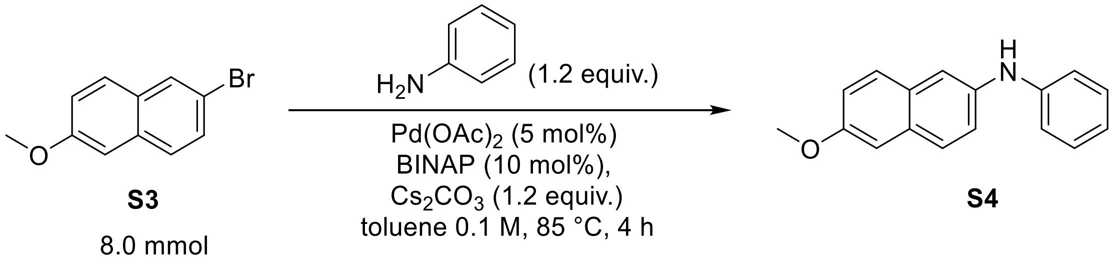

In a 200 mL round-bottom, double-neck flask under a nitrogen atmosphere, a solution of compound **S3** (1.9 g, 8.0 mmol) in dry toluene (80.0 mL), $Pd(OAc)_2$ (90.0 mg, 0.40 mmol), BINAP (500.0 mg, 0.80 mmol), and Cs_2CO_3 (3.1 g, 9.6 mmol) was stirred at 60 °C. After 10 minutes, aniline (0.9 g, 9.6 mmol) was added dropwise while stirring. The temperature was then raised to 85 °C and maintained for 4 hours until the reaction was completed, as confirmed by TLC. The reaction mixture was quenched with water, extracted with ethyl acetate (3 times), dried over anhydrous Na_2SO_4, and concentrated under reduced pressure. The residue was purified by column chromatography on silica gel (*n*-hexane/ethyl acetate = 20/1) to yield the product **S4** as a pale red solid (1.2 g, 60% yield). The obtained spectra matched the literature spectra[2].

m.p. 135–136 °C; **^{1}H-NMR** (400 MHz, $CDCl_3$) δ 7.70 (d, J = 8.7 Hz, 1H), 7.61 (d, J = 9.2 Hz, 1H), 7.45 (d, J = 1.8 Hz, 1H), 7.33 (t, J = 7.8 Hz, 2H), 7.26 (dd, J = 8.7, 2.3 Hz, 1H), 7.13–7.18 (m, 4H), 6.99 (t, J = 7.3 Hz, 1H), 5.78 (s, 1H), 3.94 (s, 3H); **^{13}C-NMR** (100 MHz, $CDCl_3$) δ 156.38, 143.79, 138.90, 130.37, 129.99, 129.49, 128.19, 127.98, 121.25, 120.83, 119.17, 117.47, 113.52, 106.15, 55.40.

N-Phenyl-6-(thiophen-3-yl)naphthalen-2-amine **S6**

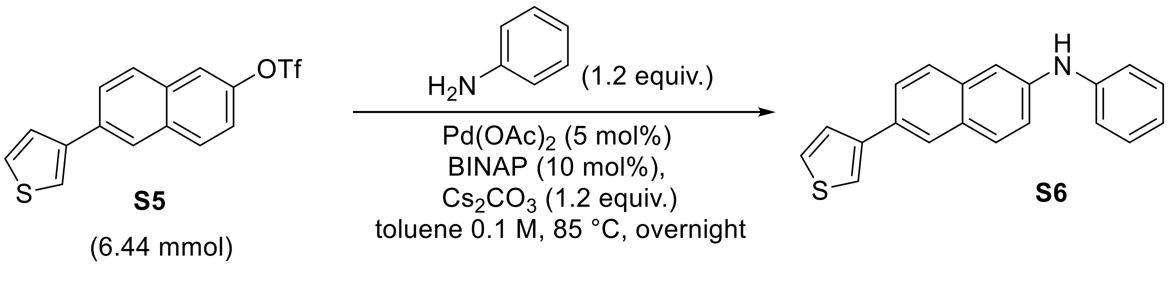

In a 100 mL round-bottom, double-neck flask under a nitrogen atmosphere, a solution of compound **S5** (2.19 g, 6.44 mmol) in dry toluene (64.0 mL), $Pd(OAc)_2$ (72.2 mg, 0.322 mmol), BINAP (401.0 mg, 0.644 mmol), and Cs_2CO_3 (2.52 g, 7.72 mmol) was stirred at 60 °C. After 10 minutes, aniline (719.0 mg, 7.72 mmol) was added dropwise while stirring. The temperature was then raised to 85 °C and maintained overnight until the reaction was completed, as confirmed by TLC. The reaction mixture was quenched with water and extracted with ethyl acetate (3 times), dried over anhydrous Na_2SO_4, and concentrated

under reduced pressure. The residue was purified by column chromatography on silica gel (*n*-hexane/EtOAc = 20/1) to yield the product **S6** as a pale yellow solid (1.089 g, 56% yield).

m.p. 164–165 °C; **^{1}H-NMR** (400 MHz, $(CD_3)_2CO$) δ 8.09 (d, J = 1.4 Hz, 1H), 7.82 (d, J = 9.2 Hz, 1H), 7.77–7.79 (m, 2H), 7.70–7.73 (m, 2H), 7.64 (dd, J = 5.0, 1.4 Hz, 1H), 7.57 (dd, J = 5.0, 3.2 Hz, 1H), 7.54 (d, J = 1.8 Hz, 1H), 7.26–7.35 (m, 5H), 6.93 (tt, J = 6.9, 1.4 Hz, 1H); **^{13}C-NMR** (100 MHz, $(CD_3)_2CO$) δ 144.19, 143.18, 142.65, 134.94, 131.27, 130.10, 129.96, 127.77, 127.27, 127.01, 126.09, 125.20, 121.57, 121.22, 120.60, 118.79, 110.49 (one carbon overlapped); **HRMS** (APCI) calcd for $[M + H]^+$ 302.0998, found: 302.0993; **IR** (KBr) 3421, 3094, 1710, 1627, 1600, 1496, 1311, 1237, 1168, 871 cm^{-1}.

N,6-Diphenylnaphthalen-2-amine **S8**

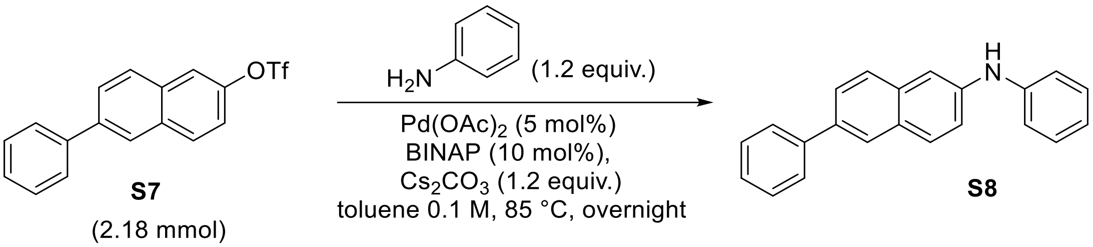

In a 100 mL round-bottom, double-neck flask under a nitrogen atmosphere, a solution of compound **S7** (769.0 mg, 2.18 mmol) in dry toluene (21.0 mL), $Pd(OAc)_2$ (24.5 mg, 0.109 mmol), BINAP (136.0 mg, 0.218 mmol), and Cs_2CO_3 (853.0 mg, 2.62 mmol) was stirred at 60 °C. After 10 minutes, aniline (244.0 mg, 2.62 mmol) was added dropwise while stirring. The temperature was then raised to 85 °C and maintained overnight until the reaction was completed, as confirmed by TLC. The reaction mixture was quenched with water, extracted with ethyl acetate (3 times), dried over anhydrous Na_2SO_4, and concentrated under reduced pressure. The residue was purified by column chromatography on silica gel (*n*-hexane/EtOAc = 20/1) to yield the product **S8** as a pale red solid (450 mg, 70% yield).

m.p. 118–119 °C; **^{1}H-NMR** (400 MHz, $CDCl_3$) δ 7.96 (s, 1H), 7.81 (d, J = 8.7 Hz, 1H), 7.68–7.74 (m, 4H), 7.47–7.50 (m, 3H), 7.31–7.39 (m, 3H), 7.26 (dd, J = 8.7, 2.3 Hz, 1H), 7.20 (dd, J = 8.5, 1.1 Hz, 2H), 7.00 (tt, J = 6.9, 1.4 Hz, 1H), 5.91 (s, 1H); **^{13}C-NMR** (100 MHz, $CDCl_3$) δ 142.90, 141.37, 141.15, 136.30, 133.96, 129.66, 129.60, 129.47, 128.96, 127.30, 127.16, 127.12, 126.27, 125.72, 121.67, 120.51, 118.51, 111.25; **HRMS** (APCI) calcd for $[M + H]^+$ 296.1434, found:296.1428. **IR** (KBr) 3418, 3058, 1600, 1497, 1448, 1307, 1229, 1139, 1078, 866 cm^{-1}.

6-(Phenylamino)-2-naphthonitrile **S10**

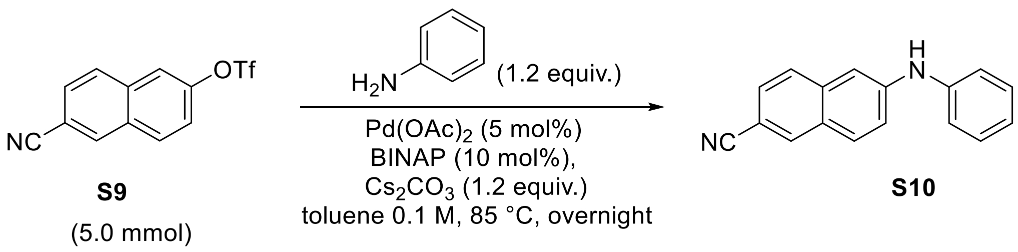

In a 100 mL round-bottom, double-neck flask under a nitrogen atmosphere, a solution of compound **S9** (1.5 g, 5.0 mmol) in dry toluene (50.0 mL), $Pd(OAc)_2$ (56.0 mg, 0.25 mmol), BINAP (310.0 mg, 0.5 mmol), and Cs_2CO_3 (1.9 g, 6.0 mmol) was stirred at 60 °C. After 10 minutes, aniline (560.0 mg, 6.0 mmol) was added dropwise while stirring. The temperature was then raised to 85 °C and maintained overnight until the reaction was completed, as confirmed by TLC. The reaction mixture was quenched with water, extracted with ethyl acetate (3 times), dried over anhydrous Na_2SO_4, and concentrated under reduced pressure. The residue was purified by column chromatography on silica gel (*n*-hexane/EtOAc = 20/1) to yield the product **S10** as a yellow solid (730.0 mg, 60% yield).

m.p. 153–154 °C; **^{1}H-NMR** (400 MHz, $CDCl_3$) δ 8.07 (s, 1H), 7.75 (d, J = 8.7 Hz, 1H), 7.63 (d, J = 8.7 Hz, 1H), 7.49 (dd, J = 8.5, 1.6 Hz, 1H), 7.36–7.41 (m, 3H), 7.24–7.28 (m, 3H), 7.11 (t, J = 7.6 Hz, 1H), 6.01 (s, 1H); **^{13}C-NMR** (100 MHz, $CDCl_3$) δ 144.48, 141.16, 136.62, 133.91, 130.03, 129.74, 127.43, 127.36, 127.25, 123.32, 120.60, 120.38, 120.04, 108.82, 105.81; **HRMS** (APCI) calcd for $[M + H]^+$ 245.1073, found: 245.1070; **IR** (KBr) 3359, 3060, 2213, 1624, 1591, 1535, 1398, 1164, 804 cm^{-1}.

6-(Phenylamino)naphthalen-2-yl trifluoromethanesulfonate **S12**

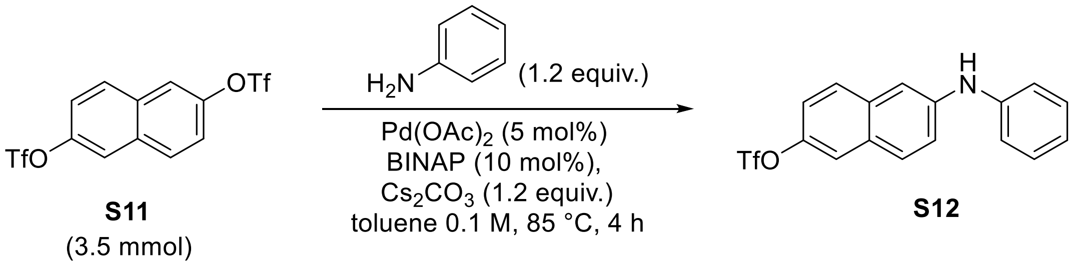

In a 100 mL round-bottom, double-neck flask under a nitrogen atmosphere, a solution of compound **S11** (1.5 g, 3.5 mmol) in dry toluene (35.0 mL), $Pd(OAc)_2$ (40.0 mg, 0.18 mmol), BINAP (220.0 mg, 0.35 mmol), and Cs_2CO_3 (1.4 g, 4.2 mmol) was stirred at 60 °C. After 10 minutes, aniline (391.1 mg, 4.2 mmol) was added dropwise while stirring. The temperature was then raised to 85 °C and maintained for 4 hours until the reaction was completed, as confirmed by TLC. The reaction mixture was quenched with water, extracted with ethyl acetate (3 times), dried over anhydrous Na_2SO_4, and concentrated under reduced pressure. The residue was purified by column chromatography on silica gel (*n*-hexane/ethyl acetate = 20/1) to yield the product **S12** as a brown liquid, which solidified under vacuum (390 mg, 30% yield).

m.p. 80–81 °C; **¹H-NMR** (400 MHz, $CDCl_3$) δ 7.74 (d, J = 8.7 Hz, 1H), 7.67 (d, J = 8.7 Hz, 1H), 7.63 (d, J = 2.7 Hz, 1H), 7.42 (d, J = 2.3 Hz, 1H), 7.36 (t, J = 7.8 Hz, 2H), 7.28–7.30 (m, 2H), 7.20 (d, J = 7.8 Hz, 2H), 7.06 (t, J = 7.3 Hz, 1H), 5.96 (s, 1H); **¹³C-NMR** (100 MHz, $CDCl_3$) δ 145.39, 142.62, 142.00, 133.97, 129.68, 129.38, 128.78, 128.44, 122.52, 121.25, 120.16, 119.38, 119.19, 118.95 (q, J = 321.09 Hz), 110.04; **HRMS** (APCI) calcd for $[M + H]^+$ 368.0563, found: 368.0562; **IR** (KBr) 3418, 3058, 1907, 1751, 1631, 1508, 1414, 1208, 908, 805 cm^{-1}.

N-Phenylphenanthren-9-amine **S14**

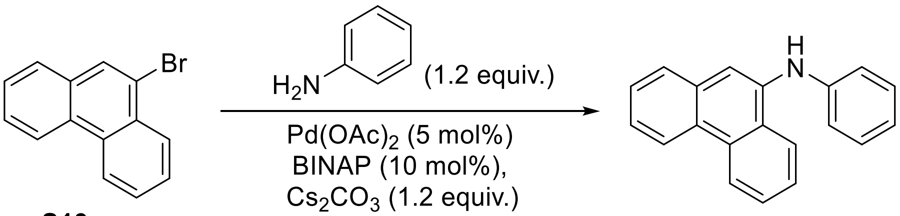

S13
(5.8 mmol)
S14

In a 100 mL round-bottom, double-neck flask under a nitrogen atmosphere, a solution of compound **S13** (1.5 g, 5.8 mmol) in dry toluene (58.0 mL), $Pd(OAc)_2$ (65.0 mg, 0.29 mmol), BINAP (360.0 mg, 0.58 mmol), and Cs_2CO_3 (2.3 g, 7.0 mmol) was stirred at 60 °C. After 10 minutes, aniline (650.0 mg, 7.0 mmol) was added dropwise while stirring. The temperature was then raised to 85 °C and maintained for 4 hours until the reaction was completed, as confirmed by TLC. The reaction mixture was quenched with water, extracted with ethyl acetate (3 times), dried over anhydrous Na_2SO_4, and concentrated under reduced pressure. The residue was purified by column chromatography on silica gel (*n*-hexane/EtOAc = 20/1) to yield the product **S14** as a brown solid (940.0 mg, 34% yield).

m.p. 120–121 °C; **¹H-NMR** (400 MHz, $CDCl_3$) δ 8.76 (d, J = 7.8 Hz, 1H), 8.64–8.67 (m, 1H), 8.15 (dd, J = 8.2, 0.9 Hz, 1H), 7.69–7.74 (m, 2H), 7.62–7.66 (m, 1H), 7.54–7.60 (m, 3H), 7.33 (d, J = 7.3 Hz, 1H), 7.31 (d, J = 7.3 Hz, 1H), 7.07 (dd, J = 8.5, 1.1 Hz, 2H), 6.98 (t, J = 7.3 Hz, 1H), 5.94 (s, 1H); **¹³C-NMR** (100 MHz, $CDCl_3$) δ 144.75, 137.08, 132.64, 131.59, 129.53, 128.24, 127.90, 127.64, 127.04, 127.01, 126.79, 125.20, 123.40, 122.60, 122.51, 120.86, 118.04, 114.81; **HRMS** (APCI) calcd for $[M + H]^+$ 270.1277, found: 270.1276; **IR** (KBr) 3394, 3054, 2372, 1596, 1499, 1425, 1320, 1146, 938, 797 cm^{-1}.

2.4. Synthesis of 10-hydroxy benzo[*c*]carbazol derivatives 1

10-Hydroxy-7-(*p*-tolyl)-7*H*-benzo[*c*]carbazol-2-yl trifluoromethanesulfonate 1a

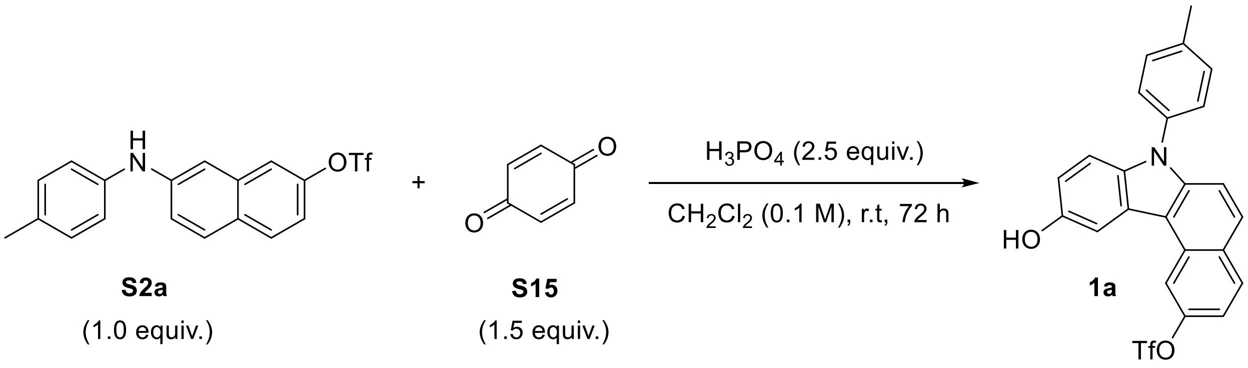

A mixture of **S2a** (950.0 mg, 2.5 mmol), *p*-quinone **S15** (410.0 mg, 3.8 mmol), and *ortho*-phosphoric acid (330.0 µL, 6.3 mmol) in CH_2Cl_2 (25.0 mL) was stirred at room temperature for 72 hours. Upon completion of the reaction, the mixture was quenched with water and further extracted with CH_2Cl_2. The combined organic extracts were washed with brine, dried over anhydrous Na_2SO_4, filtered, and concentrated under reduced pressure. The crude product was purified by silica gel column chromatography to yield the desired product **1a** (710.0 mg, 60% yield) as a white solid.

m.p. 165–166 °C; ^{1}**H-NMR** (600 MHz, $CDCl_3$) δ 8.54 (d, *J* = 2.1 Hz, 1H), 8.03 (d, *J* = 8.9 Hz, 1H), 7.90 (d, *J* = 2.1 Hz, 1H), 7.80 (d, *J* = 8.9 Hz, 1H), 7.56 (d, *J* = 9.3 Hz, 1H), 7.40–7.44 (m, 4H), 7.35 (dd, *J* = 8.9, 2.7 Hz, 2H), 7.04 (dd, *J* = 8.6, 2.4 Hz, 1H), 2.51 (s, 3H); ^{13}**C-NMR** (150 MHz, $CDCl_3$) δ 150.73, 148.31, 139.93, 138.32, 135.68, 134.40, 131.57, 130.74, 130.17, 128.31, 127.53, 126.91, 123.69, 119.05 (q, *J* = 320.78 Hz), 116.06, 114.95, 114.88, 114.45, 113.33, 111.60, 106.73, 21.42; **HRMS** (APCI) calcd for $[M + H]^+$ 472.0825, found: 472.0818; **IR** (KBr) 3367, 3035, 2924, 1623, 1517, 1472, 1368, 1283, 1020, 828 cm^{-1}.

10-Hydroxy-7-phenyl-7*H*-benzo[*c*]carbazol-2-yl trifluoromethanesulfonate 1b

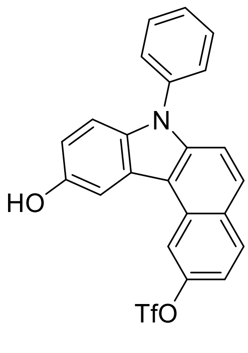

Following the same procedures as **1a**, the desired product **1b** was obtained as a white solid (56% yield).

m.p. 151–152 °C; ^{1}**H-NMR** (600 MHz, $CDCl_3$) δ 8.55 (d, *J* = 2.1 Hz, 1H), 8.05 (d, *J* = 8.2 Hz, 1H), 7.91 (d, *J* = 2.1 Hz, 1H), 7.82 (d, *J* = 8.9 Hz, 1H), 7.64 (t, *J* = 7.6 Hz, 2H), 7.53–7.59 (m, 4H), 7.35–7.38 (m, 2H), 7.04 (dd, *J* = 8.6, 2.4 Hz, 1H), 4.99 (s, 1H); ^{13}**C-NMR** (150 MHz, $CDCl_3$) δ 150.77, 148.37, 139.85, 137.15, 135.60,

131.62, 130.19, 128.39, 128.34, 127.75, 127.04, 123.85, 119.07 (q, *J* = 322.13 Hz), 116.20, 115.06, 114.99, 114.51, 113.28, 111.60, 106.81 (one carbon overlapped); **HRMS** (APCI) calcd for $[M + H]^+$ 458.0668, found: 458.0662; **IR** (KBr) 3367, 3069, 1703, 1622, 1595, 1503, 1418, 1211, 921, 877 cm^{-1}.

7-(*p*-Chlorophenyl)-10-hydroxy-7*H*-benzo[*c*]carbazol-2-yl trifluoromethanesulfonate **1c**

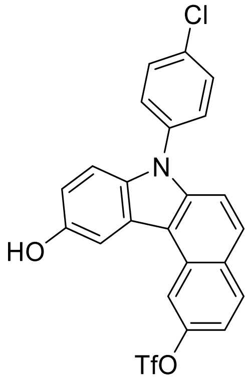

Following the same procedures as **1a**, the desired product **1c** was obtained as a white solid (47% yield).

m.p. 186–187 °C; **^{1}H-NMR** (600 MHz, $CDCl_3$) δ 8.54 (d, *J* = 2.1 Hz, 1H), 8.05 (d, *J* = 8.9 Hz, 1H), 7.90 (d, *J* = 2.1 Hz, 1H), 7.83 (d, *J* = 8.9 Hz, 1H), 7.62 (d, *J* = 8.2 Hz, 2H), 7.54 (d, *J* = 8.9 Hz, 1H), 7.49 (d, *J* = 8.2 Hz, 2H), 7.37 (dd, *J* = 8.9, 2.7 Hz, 1H), 7.34 (d, *J* = 8.9 Hz, 1H), 7.05 (dd, *J* = 8.6, 2.4 Hz, 1H), 5.14 (s, 1H); **^{13}C-NMR** (150 MHz, $CDCl_3$) δ 151.00, 148.42, 139.65, 135.69, 135.38, 134.09, 131.68, 130.46, 130.12, 129.03, 128.45, 127.24, 124.00, 119.06 (q, *J* = 320.78 Hz), 116.39, 115.29, 115.02, 114.67, 112.93, 111.36, 106.94; **HRMS** (APCI) calcd for $[M + H]^+$ 492.0279, found: 492.0276; **IR** (KBr) 3409, 2930, 2853, 1626, 1525, 1496, 1420, 1205, 1013, 823 cm^{-1}.

10-Hydroxy-7-(*o*-anisyl)-7*H*-benzo[*c*]carbazol-2-yl trifluoromethanesulfonate **1d**

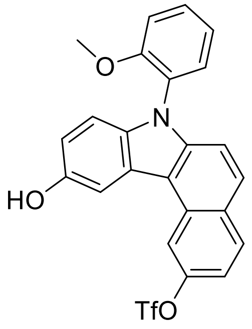

Following the same procedures as **1a**, the desired product **1d** was obtained as a white solid (50% yield).

m.p. 120–121 °C; **^{1}H-NMR (**400 MHz, $(CD_3)_2CO$) δ 8.73 (d, *J* = 2.3 Hz, 1H), 8.47 (s, 1H), 8.13 (s, 1H), 8.09 (d, *J* = 9.2 Hz, 1H), 7.79 (d, *J* = 8.9 Hz, 1H), 7.47–7.51 (m, 1H), 7.41–7.46 (m, 2H), 7.30 (d, *J* = 8.7 Hz, 1H), 7.21–

7.23 (m, 1H), 7.13–7.17 (m, 3H), 3.60 (s, 3H); **^{13}C-NMR** (100 MHz, $(CD_3)_2CO$) δ 156.73, 153.32, 149.05, 140.76, 135.88, 132.58, 131.02, 130.71, 130.58, 129.08, 127.28, 125.68, 124.35, 121.97, 119.88 (q, J = 320.13 Hz), 116.40, 115.49, 115.34, 115.28, 114.55, 113.70, 112.36, 106.92, 55.86; **HRMS** (APCI) calcd for $[M + H]^+$ 488.0774, found: 488.0769; **IR** (KBr) 3383, 3074, 2941, 2838, 1701, 1622, 1527, 1417, 1039, 830 cm^{-1}.

7-(*p*-Bromophenyl)-10-hydroxy-7*H*-benzo[*c*]carbazol-2-yl trifluoromethanesulfonate 1e

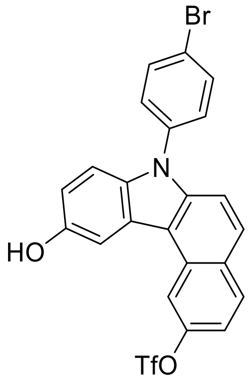

Following the same procedures as **1a**, the desired product **1e** was obtained as a white solid (26% yield).

m.p. 156–157 °C; **^{1}H-NMR** (400 MHz, $CDCl_3$) δ 8.55 (d, J = 2.3 Hz, 1H), 8.06 (d, J = 8.7 Hz, 1H), 7.90 (d, J = 2.3 Hz, 1H), 7.84 (d, J = 9.2 Hz, 1H), 7.78 (d, J = 8.7 Hz, 2H), 7.56 (d, J = 9.2 Hz, 1H), 7.44 (d, J = 8.7 Hz, 2H), 7.34–7.39 (m, 2H), 7.05 (dd, J = 8.7, 2.3 Hz, 1H), 4.98 (s, 1H); **^{13}C-NMR** (100 MHz, $CDCl_3$) δ 151.07, 148.42, 139.57, 136.26, 135.30, 133.45, 131.66, 130.12, 129.32, 128.47, 127.23, 124.04, 121.98, 119.08 (q, J = 321.09 Hz), 116.38, 115.35, 114.99, 114.71, 112.90, 111.34, 106.97; **HRMS** (APCI) calcd for $[M + H]^+$ 535.9774, found: 535.9771; **IR** (KBr) 3393, 3069, 2920, 1619, 1494, 1418, 1213, 1138, 920, 822 cm^{-1}.

7-Benzyl-10-hydroxy-7*H*-benzo[*c*]carbazol-2-yl trifluoromethanesulfonate 1f

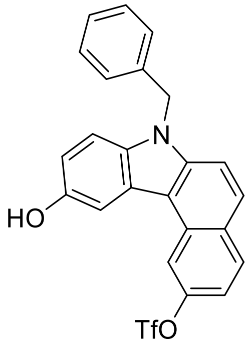

Following the same procedures as **1a**, the desired product **1f** was obtained as a white solid (40% yield).

m.p. 157–158 °C; **^{1}H-NMR** (600 MHz, $(CD_3)_2CO$) δ 8.65 (s, 1H), 8.24 (d, J = 8.6 Hz, 1H), 8.03 (d, J = 8.9 Hz, 1H), 7.98 (s, 1H), 7.90 (d, J = 8.9 Hz, 1H), 7.59 (d, J = 8.9 Hz, 1H), 7.53 (dd, J = 8.6, 1.7 Hz, 1H), 7.32–7.36 (m, 3H), 7.20 (d, J = 6.9 Hz, 2H), 7.15 (dd, J = 8.6, 1.7 Hz, 1H), 5.78 (s, 2H); **^{13}C-NMR** (150 MHz, $(CD_3)_2CO$) δ 153.16, 149.17, 140.22, 138.69, 135.11, 132.74, 130.83, 129.51, 128.89, 128.22, 127.51, 127.30, 124.26, 119.86 (q, J = 320.78 Hz), 116.43, 115.47, 115.27, 114.96, 113.92, 111.88, 107.04, 46.96; **HRMS (APCI)** calcd for $[M + H]^+$ 472.0825, found: 472.0825; **IR** (KBr) 3441, 3032, 2268, 1625, 1484, 1415, 1208, 1136, 968, 831 cm^{-1}.

7-*n*-Hexyl-10-hydroxy-7*H*-benzo[*c*]carbazol-2-yl trifluoromethanesulfonate **1g**

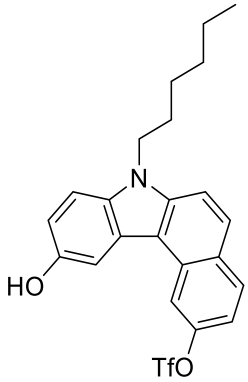

Following the same procedures as **1a**, the desired product **1g** was obtained as a white solid (19% yield).

m.p. 101–102 °C; **^{1}H-NMR** (400 MHz, $CDCl_3$) δ 8.35 (d, J = 1.8 Hz, 1H), 7.92 (d, J = 8.7 Hz, 1H), 7.79 (d, J = 1.8 Hz, 1H), 7.71 (d, J = 8.7 Hz, 1H), 7.47 (d, J = 9.2 Hz, 1H), 7.32 (d, J = 8.7 Hz, 1H), 7.25 (dd, J = 8.9, 2.5 Hz, 1H), 7.12 (dd, J = 8.7, 1.8 Hz, 1H), 5.66 (s, 1H), 4.17 (t, J = 7.3 Hz, 2H), 1.75 (quintet, J = 6.9 Hz, 2H), 1.19–1.33 (m, 6H), 0.84 (t, J = 6.6 Hz, 3H); **^{13}C-NMR** (100 MHz, $CDCl_3$) δ 149.98, 148.07, 138.90, 134.44, 131.35, 130.06, 127.52, 126.41, 123.17, 119.03 (q, J = 321.09 Hz), 115.39, 114.50, 114.05, 112.09, 110.29, 106.87, 43.17, 31.57, 29.48, 26.91, 22.58, 14.04 (one carbon overlapped); **HRMS** (APCI) calcd for $[M + H]^+$ 466.1294, found: 466.1289; **IR** (KBr) 3336, 2956, 2927, 2860, 1624, 1528, 1249, 1146, 919, 877 cm^{-1}.

10-Hydroxy-8,9-dimethyl-7-(*p*-tolyl)-7*H*-benzo[*c*]carbazol-2-yl trifluoromethanesulfonate 1h

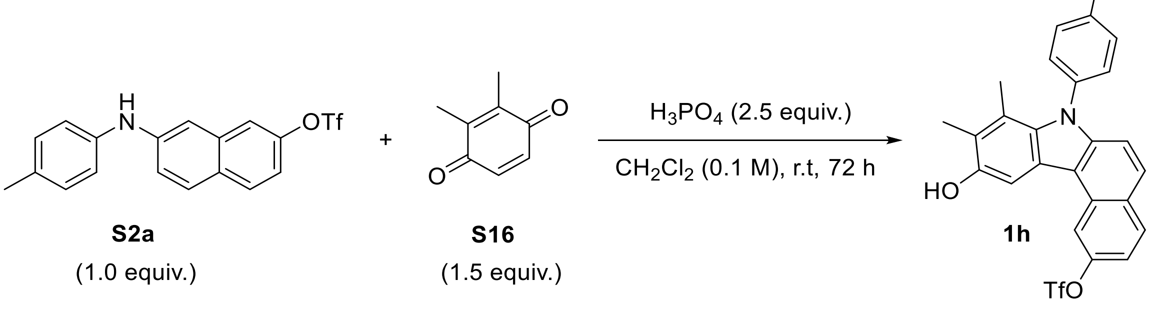

Following the same procedures as **1a**, the desired product **1h** was obtained as a white solid (65% yield).

m.p. 170–171 °C; **^{1}H-NMR** (400 MHz, $CDCl_3$) δ 8.51 (d, J = 2.3 Hz, 1H), 7.97 (d, J = 8.7 Hz, 1H), 7.74 (s, 1H), 7.67 (d, J = 8.7 Hz, 1H), 7.35 (d, J = 7.8 Hz, 2H), 7.30 (dd, J = 8.9, 2.5 Hz, 1H), 7.22–7.27 (m, 3H), 5.11 (s, 1H), 2.51 (s, 3H), 2.34 (s, 3H), 2.00 (s, 3H); **^{13}C-NMR** (100 MHz, $CDCl_3$) δ 149.10, 148.11, 141.51, 138.62, 137.33, 135.11, 131.45, 130.20, 129.67, 129.08, 128.31, 126.08, 122.70, 122.00, 119.05 (q, J = 320.13 Hz), 115.74, 114.87, 114.66, 113.60, 104.02, 21.45, 15.61, 12.54 (one carbon overlapped); **HRMS** (APCI) calcd for $[M + H]^+$ 500.1138, found: 500.1135; **IR** (KBr) 3362, 3073, 2958, 1621, 1515, 1420, 1207, 1148, 917, 830 cm^{-1}.

10-Hydroxy-9-methyl-7-(*p*-tolyl)-7*H*-benzo[*c*]carbazol-2-yl trifluoromethanesulfonate **1i**

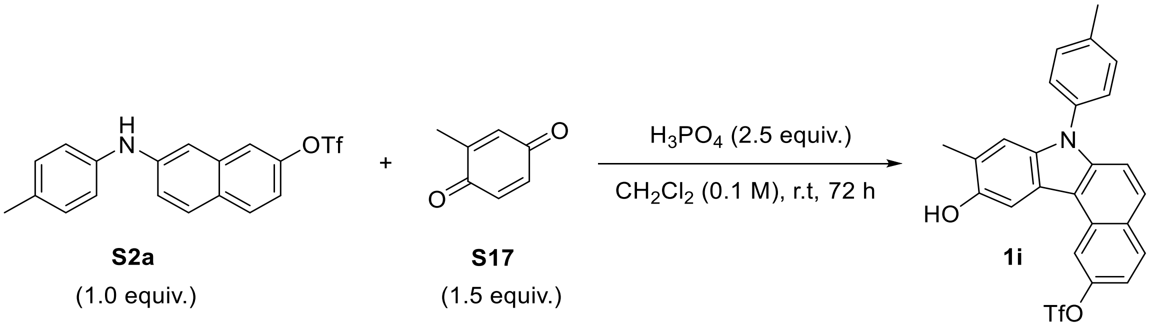

Following the same procedures as **1a**, the desired product **1i** was obtained as a white solid (55% yield).

m.p. 205–206 °C; **^{1}H-NMR** (600 MHz, $CDCl_3$) δ 8.52 (s, 1H), 8.03 (d, J = 8.9 Hz, 1H), 7.84 (s, 1H), 7.77 (d, J = 8.9 Hz, 1H), 7.54 (d, J = 8.9 Hz, 1H), 7.41–7.45 (m, 4H), 7.34 (d, J = 8.9 Hz, 1H), 7.23 (s, 1H), 4.96 (s, 1H), 2.52 (s, 3H), 2.43 (s, 3H); **^{13}C-NMR** (150 MHz, $CDCl_3$) δ 149.39, 148.21, 139.44, 138.26, 135.84, 134.60, 131.53, 130.75, 129.96, 128.30, 127.61, 126.28, 123.56, 121.66, 119.08 (q, J = 320.78 Hz), 115.92, 115.08, 114.95, 113.31, 112.27, 106.30, 21.43, 17.11; **HRMS** (APCI) calcd for $[M + H]^+$ 486.0981, found: 486.0982; **IR** (KBr) 3393, 2921, 2851, 1727, 1621, 1522, 1420, 1196, 923, 827 cm^{-1}.

3-Methoxy-7-phenyl-7*H*-benzo[*c*]carbazol-10-ol **1k'**

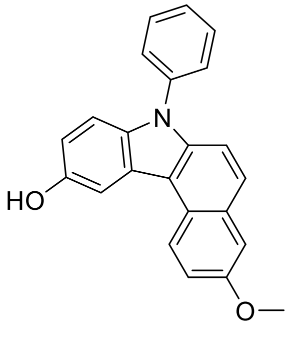

Following the same procedures as **1a**, the desired product **1k'** was obtained as a white solid (49% yield).

m.p. 171–172 °C; **^{1}H-NMR** (400 MHz, $(CD_3)_2CO$) δ 8.67 (d, *J* = 9.2 Hz, 1H), 8.15 (s, 1H), 8.06 (d, *J* = 2.3 Hz, 1H), 7.82 (d, *J* = 8.7 Hz, 1H), 7.70–7.74 (m, 2H), 7.62–7.65 (m, 2H), 7.51–7.59 (m, 3H), 7.41 (dd, *J* = 9.2, 2.7 Hz, 1H), 7.34 (d, *J* = 8.7 Hz, 1H), 7.04 (dd, *J* = 8.7, 2.3 Hz, 1H), 3.96 (s, 3H); **^{13}C-NMR** (175 MHz, $(CD_3)_2CO$) δ 156.57, 153.00, 138.67, 138.55, 135.48, 131.52, 130.92, 128.53, 128.23, 127.22, 125.64, 125.04, 125.01, 119.41, 116.03, 114.88, 112.89, 111.53, 109.26, 107.52, 55.58; **HRMS** (APCI) calcd for $[M + H]^+$ 340.1332, found: 340.1327; **IR** (KBr) 3275, 2922, 2368, 1709, 1593, 1502, 1374, 1164, 1026, 803 cm^{-1}.

7-Phenyl-3-(thiophen-3-yl)-7*H*-benzo[*c*]carbazol-10-ol 1l'

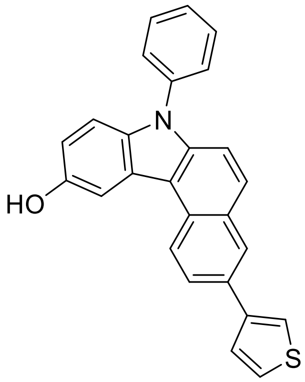

Following the same procedures as **1a**, the desired product **1l'** was obtained as a white solid (62% yield).

m.p. 193–194 °C; **^{1}H-NMR** (600 MHz, $CDCl_3$) δ 8.74 (d, *J* = 8.7 Hz, 1H), 8.20 (d, *J* = 1.8 Hz, 1H), 8.07 (d, *J* = 2.7 Hz, 1H), 7.98 (dd, *J* = 8.5, 2.1 Hz, 1H), 7.84 (d, *J* = 9.2 Hz, 1H), 7.50–7.66 (m, 8H), 7.46 (dd, *J* = 5.0, 3.2 Hz, 1H), 7.38 (d, *J* = 8.7 Hz, 1H), 7.01 (dd, *J* = 8.7, 2.3 Hz, 1H), 4.77 (s, 1H); **^{13}C-NMR** (175 MHz, $CDCl_3$) δ 150.35, 142.57, 139.37, 137.52, 135.55, 130.60, 130.06, 129.67, 129.01, 127.96, 127.76, 127.72, 126.58, 126.44, 126.41, 126.04, 124.44, 123.66, 120.08, 115.10, 113.77, 112.42, 111.18, 107.40; **HRMS** (APCI) calcd for $[M + H]^+$ 392.1104, found: 392.1098; **IR** (KBr) 3327, 3106, 2922, 1700, 1592, 1496, 1387, 1165, 940, 841 cm^{-1}.

3,7-Diphenyl-7*H*-benzo[*c*]carbazol-10-ol 1m'

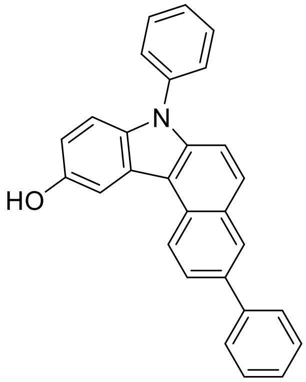

Following the same procedures as **1a**, the desired product **1m'** was obtained as a white solid (59% yield).

m.p. 187–187 °C; **^{1}H-NMR** (400 MHz, $CDCl_3$) δ 8.79 (d, J = 8.7 Hz, 1H), 8.21 (d, J = 1.8 Hz, 1H), 8.09 (d, J = 2.3 Hz, 1H), 7.99 (dd, J = 8.7, 1.8 Hz, 1H), 7.87 (d, J = 9.2 Hz, 1H), 7.80 (d, J = 6.9 Hz, 2H), 7.49–7.66 (m, 8H), 7.37–7.41 (m, 2H), 7.01 (dd, J = 8.7, 2.3 Hz, 1H), 4.83 (s, 1H); **^{13}C-NMR** (100 MHz, $CDCl_3$) δ 150.25, 141.30, 139.35, 137.49, 135.65, 135.54, 129.98, 129.67, 129.05, 128.97, 127.86, 127.65, 127.35, 127.19, 127.10, 126.44, 124.50, 123.61, 115.00, 113.87, 112.29, 111.19, 107.57 (one carbon overlapped); **HRMS** (APCI) calcd for $[M + H]^+$ 386.1539, found: 386.1537; **IR** (KBr) 3316, 3059, 2924, 1622, 1584, 1498, 1367, 1166, 1037, 841 cm^{-1}.

10-Hydroxy-7-phenyl-7*H*-benzo[*c*]carbazole-3-carbonitrile 1n'

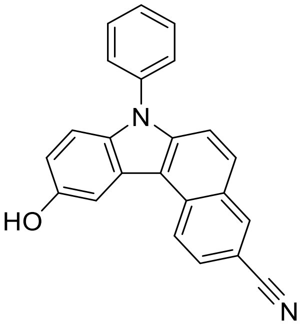

Following the same procedures as **1a**, the desired product **1n'** was obtained as a white solid (71% yield).

m.p. 259–260 °C; **^{1}H-NMR** (400 MHz, $(CD_3)_2CO$) δ 8.90 (d, J = 8.7 Hz, 1H), 8.56 (d, J = 1.8 Hz, 1H), 8.41 (s, 1H), 8.11 (d, J = 2.3 Hz, 1H), 8.03 (d, J = 8.7 Hz, 1H), 7.97 (dd, J = 8.7, 1.8 Hz, 1H), 7.75 (t, J = 7.6 Hz, 2H), 7.61–7.69 (m, 4H), 7.36 (d, J = 8.7 Hz, 1H), 7.11 (dd, J = 8.7, 2.3 Hz, 1H); **^{13}C-NMR** (150 MHz, $(CD_3)_2CO$) δ 153.78, 141.19, 137.80, 135.79, 135.67, 132.46, 131.10, 129.28, 129.25, 128.72, 128.44, 128.30, 124.94, 124.79, 120.17, 115.81, 115.54, 114.51, 112.15, 107.64, 106.62; **HRMS** (APCI) calcd for $[M + H]^+$ 335.1179, found: 335.1175; **IR** (KBr) 3347, 2860, 2714, 2225, 1710, 1618, 1385, 1247, 904, 802 cm^{-1}.

10-Hydroxy-7-phenyl-7*H*-benzo[*c*]carbazol-3-yl trifluoromethanesulfonate 1o'

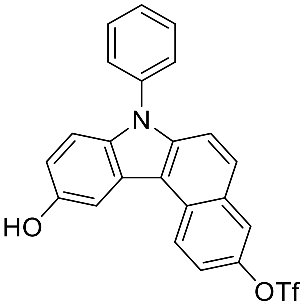

Following the same procedures as **1a**, the desired product **1o'** was obtained as a white solid (54% yield).

m.p. 124–125 °C; **^{1}H-NMR** (600 MHz, $CDCl_3$) δ 8.73 (d, J = 8.9 Hz, 1H), 7.98 (d, J = 2.1 Hz, 1H), 7.87 (d, J = 2.7 Hz, 1H), 7.78 (d, J = 8.9 Hz, 1H), 7.52–7.65 (m, 7H), 7.37 (d, J = 8.9 Hz, 1H), 7.03 (dd, J = 8.9, 2.1 Hz, 1H), 4.98 (s, 1H); **^{13}C-NMR** (100 MHz, $CDCl_3$) δ 150.58, 144.95, 139.59, 137.08, 135.57, 130.14, 129.44, 129.11, 128.25, 127.65, 126.99, 125.25, 123.90, 120.62, 120.15, 119.01 (q, J = 323.96 Hz), 114.98, 114.47, 113.78, 111.47, 107.18; **HRMS** (APCI) calcd for $[M + H]^+$ 458.0668, found: 458.0668; **IR** (KBr) 3336, 3066, 2920, 1623, 1595, 1503, 1419, 1209, 947, 841 cm^{-1}.

9-Phenyl-9*H*-dibenzo[*a,c*]carbazol-12-ol **1p'**

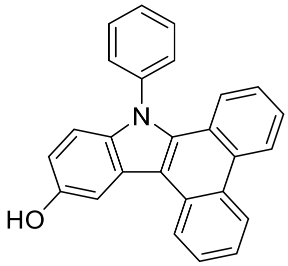

Following the same procedures as **1a**, the desired product **1p'** was obtained as a white solid (44% yield).

m.p. 246–247 °C; **^{1}H-NMR** (400 MHz, $CDCl_3$) δ 8.77–8.81 (m, 3H), 8.08 (s, 1H), 7.77 (dd, J = 8.0, 7.1 Hz, 1H), 7.49–7.67 (m, 7H), 7.46 (d, J = 8.2 Hz, 1H), 7.25–7.28 (m, 1H), 7.07 (d, J = 8.7 Hz, 1H), 6.94 (dd, J = 8.7, 2.3 Hz, 1H), 4.84 (s, 1H); **^{13}C-NMR** (150 MHz, $(CD_3)_2CO$) δ 153.50, 141.41, 137.88, 135.70, 131.59, 131.16, 130.83, 129.75, 128.33, 127.99, 126.75, 126.69, 125.13, 124.78, 124.72, 124.52, 124.21, 124.10, 123.85, 114.75, 114.47, 112.28, 107.19 (one carbon overlapped); **HRMS** (APCI) calcd for $[M + H]^+$ 360.1383, found: 360.1380; **IR** (KBr) 3305, 3063, 2918, 1608, 1594, 1497, 1368, 1205, 1157, 831 cm^{-1}.

3. **Supplementary Method 3:** *optimization of electrochemical cascade synthesis of dioxaza[8]circulenes*

Table S1. Screening of different solvents[a]

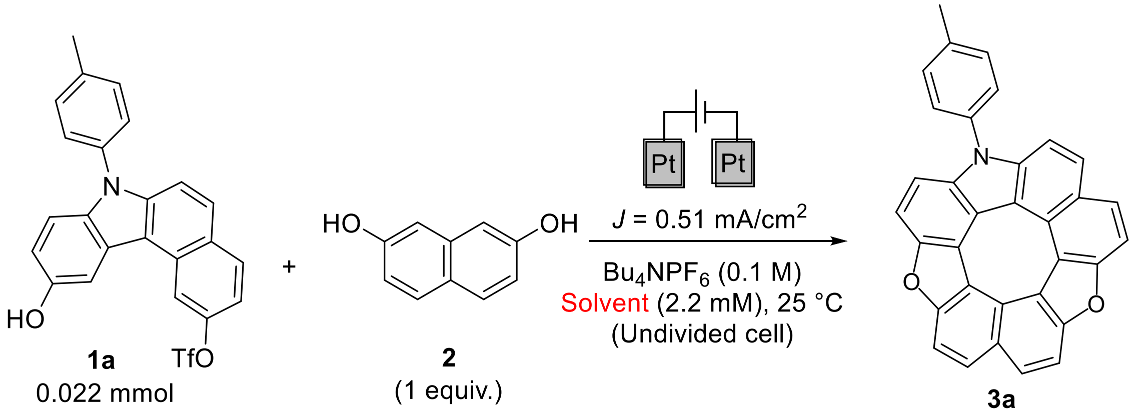

Entry	Solvent	Time	Isolated yield of **3a** (%)
1	CH_2Cl_2	3.5 h	13 %
2	$CHCl_3$	7.5 h	trace
3	MeCN	4.0 h	0 %
4	Acetone	6.5 h	0 %
5	THF	6.0 h	0 %
6	Toluene	0.0 h	N.R.

[a] Carried out in different solvents (10.0 mL) under air (1.0 atm. pressure).

Table S2. Screening of the electrochemical parameters (electrodes, electrolytes, current density)[a]

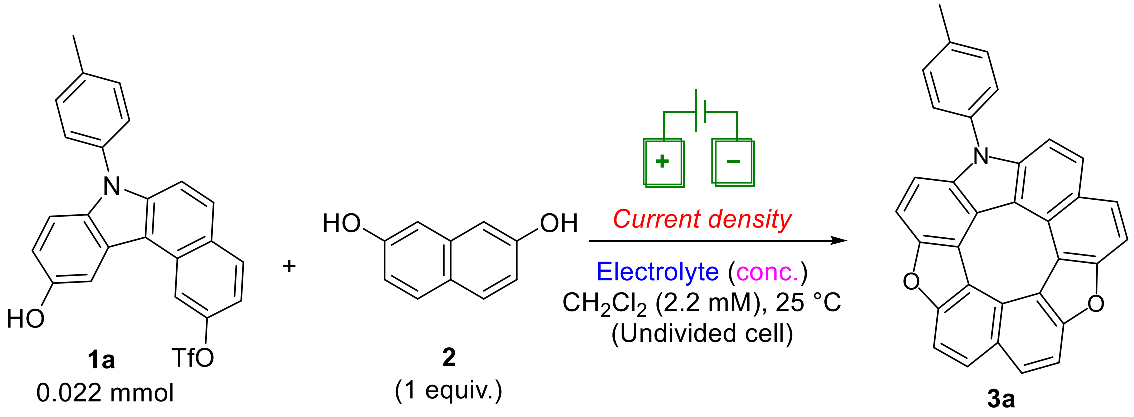

Entry	Electrodes	electrolyte (conc.)	current density (*J*)	Time (h)	Isolated yield of **3a** (%)
1	C(+)-Pt(−)	Bu_4NPF_6 (0.1 M)	0.51 mA/cm^2	4.5 h	12 %
2	FTO(+)-Pt(−)	Bu_4NPF_6 (0.1 M)	0.51 mA/cm^2	7.0 h	12 %
3	FTO-FTO	Bu_4NPF_6 (0.1 M)	0.51 mA/cm^2	6.0 h	2 %
4	Pt-Pt	Bu_4NPF_6 (0.1 M)	0.51 mA/cm^2	3.5 h	13%
6	Pt-Pt	Bu_4NClO_4 (0.1 M)	0.51 mA/cm^2	3.0 h	66 %
7	Pt-Pt	Bu_4NClO_4 (0.2M)	0.51 mA/cm^2	2.5 h	83 %
8	Pt-Pt	Bu_4NClO_4 (0.3 M)	0.51 mA/cm^2	2.5 h	83 %
9	Pt-Pt	Bu_4NClO_4 (0.05 M)	0.51 mA/cm^2	5.0 h	18%
10	Pt-Pt	Bu_4NClO_4 (0.2 M)	1.03 mA/cm^2	1.5 h	58 %
11	Pt-Pt	Bu_4NClO_4 (0.2 M)	0.26 mA/cm^2	4.0 h	64%

[a] The reaction of **1a** (0.022 mmol) and **2** (0.022 mmol) was carried out in CH_2Cl_2 (10.0 mL) at room temperatures under air (1.0 atm. pressure).

Table S3. Screening of starting material concentration and ratio[a]

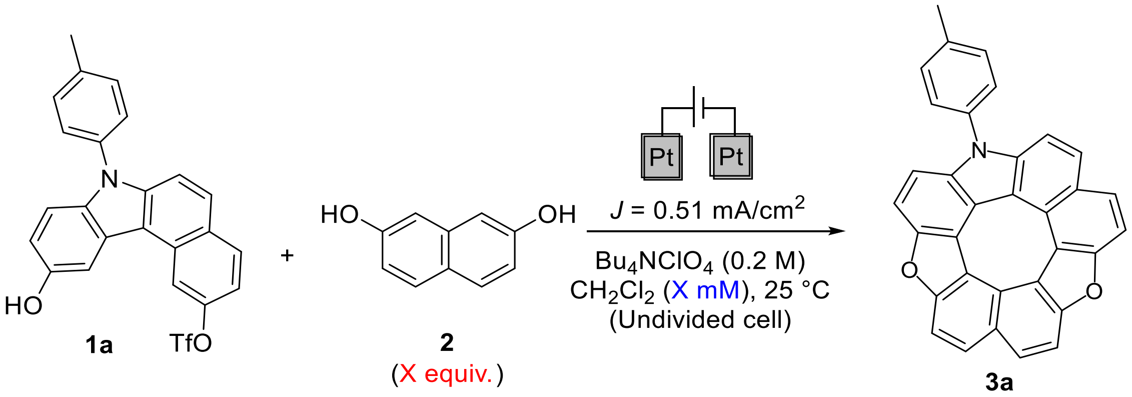

Entry	**1a** (X mM)	**2** (X equiv.)	Time (h)	Isolated yield of **3a** (%)
1	2.2	1.0	2.5 h	83 %
2	4.0	1.0	6.0 h	43 %
3	3.0	1.0	3.0 h	45 %
4	1.5	1.0	2.5 h	80 %
5	1.0	1.0	4.5 h	82 %
7	2.2	1.5	3.0 h	45 %

[a] Carried out in CH_2Cl_2 under air (1.0 atm. pressure).

4. **Supplementary Method 4:** *electrochemical cascade synthesis of dioxaza[8]circulenes*

General procedures

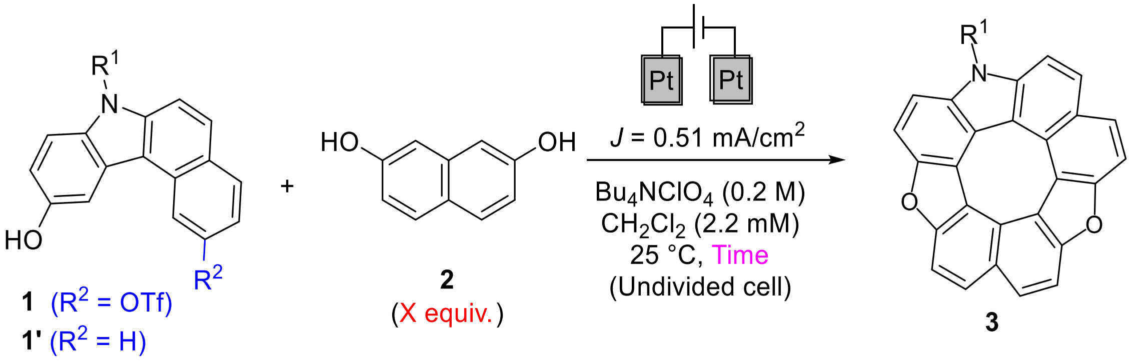

Method A:

A solution of 10-hydroxy-7*H*-benzo[*c*]carbazol-2-yl trifluoromethanesulfonate **1** (0.022 mmol), 2,7-dihydroxynaphthalene **2** (0.022 mmol), and tetrabutylammonium perchlorate (V) (2.0 mmol) in CH_2Cl_2 (10.0 mL) was transferred into an undivided electrolysis cell. This cell was equipped with two Pt electrodes (1.3 × 1.5 cm^2) connected to a DC power supply. At room temperature, a constant current electrolysis with a current density of 0.51 mA/cm^2 was applied. After stirring for 2.5–5.0 hours, the electrolysis was stopped, and the crude products were purified by column chromatography (*n*-hexane/EtOAc = 10/1), yielding the desired dioxaza[8]circulene **3**.

Method B:

A solution of 7*H*-benzo[*c*]carbazol-10-ol **1'** (*prepared according to our previous report*[3]) (0.022 mmol), 2,7-dihydroxynaphthalene **2** (0.022 mmol), and tetrabutylammonium perchlorate (V) (2.0 mmol) in CH_2Cl_2 (10.0 mL) was transferred into an undivided electrolysis cell. This cell was equipped with two Pt electrodes (1.3 × 1.5 cm^2) connected to a DC power supply. At room temperature, a constant current electrolysis with a current density of 0.51 mA/cm^2 was applied. After stirring for around 5 hours, the electrolysis was stopped, and the crude products were purified by column chromatography (*n*-hexane/EtOAc = 10/1), yielding the desired dioxaza[8]circulene **3**.

Method C:

A solution of 7*H*-benzo[*c*]carbazol-10-ol **1'** (0.022 mmol), 2,7-dihydroxynaphthalene **2** (0.044 mmol), and tetrabutylammonium perchlorate (V) (2.0 mmol) in CH_2Cl_2 (10.0 mL) was transferred into an undivided electrolysis cell. This cell was equipped with two Pt electrodes (1.3 × 1.5 cm^2) connected to a DC power supply. At room temperature, a constant current electrolysis with a current density of 0.51 mA/cm^2 was applied. After stirring for around 5 hours, the electrolysis was stopped, and the crude products were purified by column chromatography (*n*-hexane/EtOAc = 10/1), yielding the desired dioxaza[8]circulene **3**.

Spectral data of dioxaza[8]circulenes

Dioxaza[8]circulene **3a**

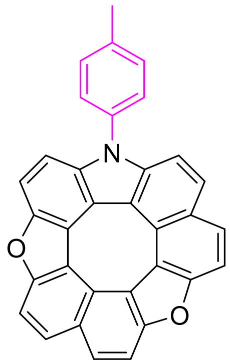

Method A: 83% yield, 78% FE, 2.5 h

Method B: 55% yield, 39% FE, 5.0 h

Method C: 58% yield, 41% FE, 5.0 h

Yellowish white compound; **m.p.** > 300 °C; **^{1}H-NMR** (700 MHz, $CDCl_3$) δ 8.34–8.36 (m, 3H), 8.23 (d, *J* = 8.6 Hz, 1H), 8.09 (d, *J* = 8.6 Hz, 1H), 8.04–8.06 (m, 2H), 7.95 (d, *J* = 8.6 Hz, 1H), 7.74 (d, *J* = 8.6 Hz, 1H), 7.70 (d, *J* = 8.6 Hz, 1H), 7.60 (d, *J* = 8.2 Hz, 2H), 7.54 (d, *J* = 7.7 Hz, 2H), 2.59 (s, 3H); **^{13}C-NMR** (175 MHz, $CDCl_3$) δ 156.01, 155.92, 155.62, 151.19, 141.20, 138.79, 136.77, 134.89, 131.05, 130.86, 130.82, 130.46, 129.97, 128.70, 127.43, 126.55, 123.07, 122.20, 117.67, 117.23, 116.87, 115.73, 114.62, 114.57, 110.92, 110.67, 110.07, 109.64, 108.77, 108.50, 21.56; **HRMS** (APCI) calcd for $[M + H]^+$ 460.1332, found: 460.1331; **IR** (KBr) 2954, 2922, 2853, 1738, 1620, 1549, 1215, 1134, 922, 816 cm^{-1}.

Dioxaza[8]circulene **3b**

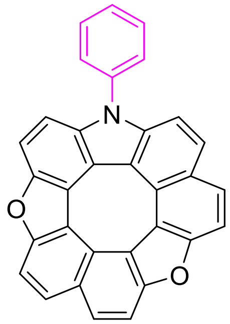

Method A: 77% yield, 61% FE, 3.0 h

Method B: 49% yield, 44% FE, 4.0 h

Method C: 55% yield, 48% FE, 4.0 h

Yellowish white compound; **m.p.** 282–283 °C; **^{1}H-NMR** (700 MHz, $CDCl_3$) *δ* 8.27–8.30 (m, 3H), 8.19 (d, *J* = 8.6 Hz, 1H), 8.02 (d, *J* = 8.6 Hz, 1H), 7.99 (dd, *J* = 8.6, 2.2 Hz, 2H), 7.91 (d, *J* = 8.6 Hz, 1H), 7.71–7.44 (m, 5H), 7.68 (d, *J* = 8.6 Hz, 1H), 7.65 (t, *J* = 7.3 Hz, 1H); **^{13}C-NMR** (175 MHz, $CDCl_3$) *δ* 155.92, 155.83, 155.50, 151.14, 140.96, 137.62, 136.54, 130.96, 130.75, 130.47, 130.36, 130.25, 128.94, 128.75, 127.34, 126.54, 122.96, 122.08, 117.53, 117.09, 116.79, 115.68, 114.66, 114.59, 110.80, 110.53, 109.87, 109.62, 108.59, 108.49; **HRMS** (APCI) calcd for $[M + H]^+$ 446.1176, found: 446.1183; **IR** (KBr) 3056, 2923, 2854, 1712, 1595, 1494, 1352, 1184, 999, 821cm^{-1}.

Dioxaza[8]circulene **3c**

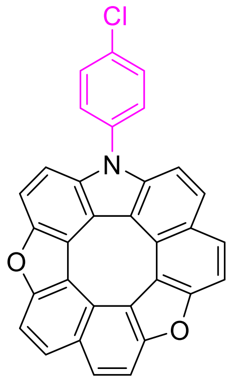

Method A: 40% yield, 31% FE, 3.0 h

Method B: 36% yield, 35% FE, 3.5 h

Method C: 47% yield, 46% FE, 3.5 h

Yellowish white compound; **m.p.** > 300 °C; **^{1}H-NMR** (600 MHz, $CDCl_3$) *δ* 8.31–8.33 (m, 3H), 8.22 (d, *J* = 8.9 Hz, 1H), 8.06 (d, *J* = 8.9 Hz, 1H), 8.02–8.04 (m, 2H), 7.94 (d, *J* = 8.2 Hz, 1H), 7.73 (d, *J* = 8.2 Hz, 2H), 7.65–7.70 (m, 4H); **^{13}C-NMR** (175 MHz, $CDCl_3$) *δ* 155.98, 155.89, 155.55, 151.22, 140.77, 136.35, 136.16, 134.58, 131.00, 130.90, 130.67, 130.55, 130.44, 130.25, 127.37, 126.63, 122.94, 122.08, 117.50, 117.02, 116.81, 115.79, 114.87, 114.77, 110.86, 110.58, 109.82, 109.54, 108.65, 108.29; **HRMS** (APCI) calcd for $[M + H]^+$ 480.0786, found:480.0790; **IR** (KBr) 2960, 2926, 2855, 1618, 1592, 1548, 1214, 1096, 922, 815 cm^{-1}.

Dioxaza[8]circulene **3d**

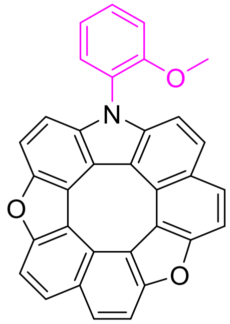

Method A: 50% yield, 24% FE, 5.0 h

Yellowish white compound; **m.p.** 263–264 °C; **^{1}H-NMR** (400 MHz, $CDCl_3$) δ 8.27 (d, J = 8.7 Hz, 1H), 8.15–8.18 (m, 3H), 7.88–7.95 (m, 4H), 7.63–7.67 (m, 2H), 7.52 (t, J = 8.9 Hz, 2H), 7.28–7.33 (m, 2H), 3.72 (s, 3H); **^{13}C-NMR** (175 MHz, $CDCl_3$) δ 156.68, 155.77, 155.67, 155.37, 151.05, 141.12, 136.50, 131.07, 130.82, 130.56, 130.48, 130.22, 130.13, 127.25, 126.46, 125.77, 123.01, 122.00, 121.52, 117.48, 117.08, 116.71, 115.66, 114.60, 114.52, 112.94, 110.58, 110.35, 110.17, 109.34, 108.67, 108.27, 55.94; **HRMS** (APCI) calcd for $[M + H]^+$ 476.1281 found: 476.1282; **IR** (KBr) 3050, 2924, 2849, 1709, 1619, 1550, 1508, 1214, 998, 823 cm^{-1}.

Dioxaza[8]circulene **3e**

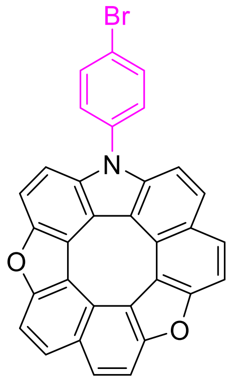

Method A: 52% yield, 41% FE, 3.0 h

Yellowish white compound; **m.p.** > 300 °C; **^{1}H-NMR** (600 MHz, $CDCl_3$) δ 8.32–8.35 (m, 3H), 8.23 (d, J = 8.9 Hz, 1H), 8.08 (d, J = 8.9 Hz, 1H), 8.03–8.05 (m, 2H), 7.94 (d, J = 8.2 Hz, 1H), 7.88 (d, J = 8.9 Hz, 2H), 7.70 (d, J = 8.9 Hz, 1H), 7.66 (d, J = 8.2 Hz, 1H), 7.61 (d, J = 8.2 Hz, 2H); **^{13}C-NMR** (175 MHz, $CDCl_3$) δ 156.05, 155.96, 155.61, 151.28, 140.76, 136.69, 136.32, 133.55, 131.06, 130.96, 130.73, 130.59, 130.51, 128.96, 127.43, 126.69, 123.00, 122.57, 117.56, 117.08, 116.87, 115.86, 114.94, 114.85, 110.93, 110.66, 109.90, 109.58, 108.72, 108.34; **HRMS** (APCI) calcd for $[M + H]^+$ 524.0281 found 524.0288; **IR** (KBr) 3065, 2926, 2854, 1709, 1610, 1548, 1496, 1313, 1274, 815 cm^{-1}.

Dioxaza[8]circulene **3f**

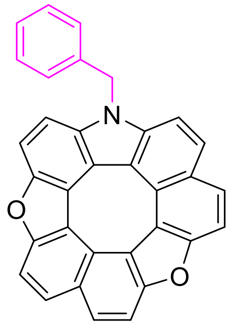

Method A: 58% yield, 39% FE, 3.5 h

Method B: 24% yield, 8% FE, 12.0 h

Method C: 44% yield, 14% FE, 12.0 h

Yellowish white compound; **m.p.** > 300 °C; **^{1}H-NMR** (600 MHz, $CDCl_3$) δ 8.32–8.37 (m, 3H), 8.26 (d, J = 8.9 Hz, 1H), 8.10 (d, J = 8.2 Hz, 1H), 8.05 (t, J = 8.2 Hz, 2H), 7.97 (d, J = 8.2 Hz, 1H), 7.86 (d, J = 8.2 Hz, 1H), 7.80 (d, J = 8.9 Hz, 1H), 7.27–7.29 (m, 2H), 7.23–7.25 (m, 1H), 7.18 (dd, J = 7.9, 2.1 Hz, 2H), 5.95 (s, 2H); **^{13}C-NMR** (175 MHz, $CDCl_3$) δ 156.06, 155.94, 155.62, 151.02, 140.32, 137.12, 135.56, 131.10, 130.90, 130.66, 130.47, 129.08, 127.79, 127.43, 126.28, 126.25, 123.21, 122.23, 117.68, 117.17, 116.74, 115.98, 114.58, 110.92, 110.67, 109.61, 108.96, 108.56, 107.64, 47.18; **HRMS** (APCI) calcd for $[M + H]^+$ 460.1332, found: 460.1326; **IR** (KBr) 3059, 3031, 2925, 2853, 1619, 1601, 1551, 1454, 823 cm^{-1}.

Dioxaza[8]circulene **3g**

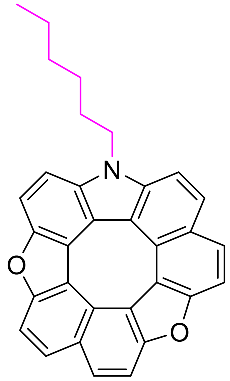

Method A: 50% yield, 34% FE, 3.5 h

Yellowish white compound; **m.p.** 196–197 °C; **^{1}H-NMR** (600 MHz, $CDCl_3$) δ 8.11–8.13 (m, 2H), 8.07 (d, J = 8.2 Hz, 1H), 7.98 (d, J = 8.9 Hz, 1H), 7.86 (d, J = 9.6 Hz, 1H), 7.83 (d, J = 8.9 Hz, 1H), 7.79 (d, J = 7.9 Hz, 1H), 7.73 (d, J = 8.9 Hz, 1H), 7.54 (d, J = 8.2 Hz, 1H), 7.52 (d, J = 8.9 Hz, 1H), 4.37 (t, J = 7.2 Hz, 2H), 1.93 (quintet, J = 7.6 Hz, 2H), 1.43 (quintet, J = 7.6 Hz, 2H), 1.26–1.36 (m, 4H), 0.87 (t, J = 6.9 Hz, 3H); **^{13}C-NMR** (175 MHz, $CDCl_3$) δ 155.46, 155.32, 155.05, 150.29, 139.40, 134.72, 130.54, 130.22, 129.88, 129.73, 127.03, 125.57, 122.70, 121.74, 117.25, 116.77, 116.15, 115.27, 113.77, 110.35, 110.10, 108.77, 108.23, 107.59, 106.81, 43.40, 31.73, 29.51, 27.17, 22.72, 14.19 (one carbon overlapped); **HRMS** (APCI) calcd for $[M + H]^+$ 454.1802, found: 454.1802; **IR** (KBr) 2952, 2927, 2855, 1620, 1456, 1348, 1276, 1216, 1115, 818 cm^{-1}.

Dioxaza[8]circulene **3h**

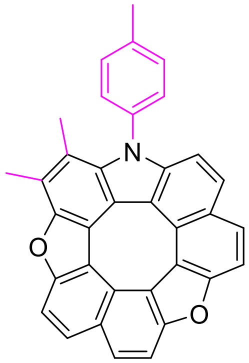

Method A: 30% yield, 20% FE, 3.5 h

Method B: 40% yield, 18% FE, 8.0 h

Method C: 56% yield, 26% FE, 8.0 h

Yellowish white compound; **m.p.** > 300 °C; **^{1}H-NMR** (600 MHz, $CDCl_3$) δ 8.34 (d, J = 8.9 Hz, 1H), 8.31 (d, J = 8.9 Hz, 2H), 8.13 (d, J = 8.9 Hz, 1H), 8.07 (d, J = 8.2 Hz, 1H), 8.06 (d, J = 8.9 Hz, 1H), 8.03 (d, J = 8.2 Hz, 1H), 7.44–7.47 (m, 5H), 2.85 (s, 3H), 2.58 (s, 3H), 2.26 (s, 3H); **^{13}C-NMR** (175 MHz, $CDCl_3$) δ 155.83, 155.32, 155.11, 150.62, 142.74, 138.81, 138.24, 135.91, 130.98, 130.28, 130.26, 130.05, 129.74, 129.69, 127.33, 126.66, 122.75, 122.19, 119.15, 118.38, 117.79, 117.73, 116.94, 114.68, 113.85, 112.98, 110.66, 110.61, 110.54, 109.52, 21.59, 16.01, 12.90; **HRMS** (APCI) calcd for $[M + H]^+$ 488.1645, found 488.1660; **IR** (KBr) 3035, 2921, 2855, 1604, 1547, 1513, 1210, 1100, 901, 821 cm^{-1}.

Dioxaza[8]circulene **3i**

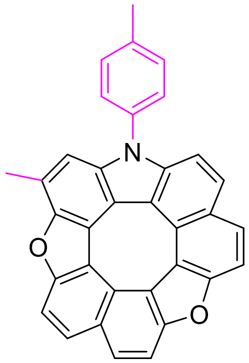

Method A: 44% yield, 26% FE, 4.0 h

Method B: 19% yield, 11% FE, 6.0 h

Method C: 30% yield, 18% FE, 6.0 h

Yellowish white compound; **m.p.** > 300 °C; **^{1}H-NMR** (700 MHz, $CDCl_3$) δ 8.30 (d, J = 8.6 Hz, 1H), 8.28 (d, J = 8.6 Hz, 1H), 8.26 (d, J = 8.6 Hz, 1H), 8.16 (d, J = 8.6 Hz, 1H), 8.03 (d, J = 8.6 Hz, 1H), 8.01 (d, J = 8.6 Hz, 1H), 7.99 (d, J = 8.2 Hz, 1H), 7.69 (d, J = 8.2 Hz, 1H), 7.59 (d, J = 8.2 Hz, 2H), 7.55 (d, J = 7.7 Hz, 2H), 7.46 (s, 1H), 2.90 (s, 3H), 2.60 (s, 3H); **^{13}C-NMR** (175 MHz, $CDCl_3$) δ 155.80, 155.57, 155.44, 150.16, 140.61, 138.64, 136.85, 135.07, 130.89, 130.83, 130.51, 130.25, 129.68, 128.72, 127.35, 126.48, 122.85, 122.27, 119.00, 117.64, 117.43, 116.81, 115.10, 114.70, 112.67, 110.74, 110.57, 109.93, 109.46, 109.25, 21.58, 16.31; **HRMS** (APCI) calcd for $[M + H]^+$ 474.1489, found 474.1483; **IR** (KBr) 3033, 2920, 2852, 1712, 1609, 1513, 1273, 1216, 1100, 815 cm^{-1}.

Dioxaza[8]circulene **3j**

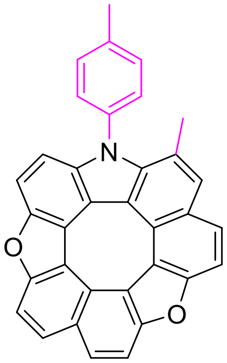

Method B: 63% yield, 56% FE, 4.0 h

Yellowish white compound; **m.p.** 258–259 °C; **^{1}H-NMR** (400 MHz, $CDCl_3$) δ 8.29–8.32 (m, 2H), 8.23 (d, J = 8.7 Hz, 1H), 8.05 (d, J = 8.7 Hz, 1H), 7.98–8.03 (m, 2H), 7.95 (s, 1H), 7.87 (d, J = 8.7 Hz, 1H), 7.42–7.52 (m, 4H), 7.38 (d, J = 8.7 Hz, 1H), 2.58 (s, 3H), 2.29 (s, 3H); **^{13}C-NMR** (175 MHz, $CDCl_3$) δ 155.65, 155.55, 155.34, 151.22, 140.30, 139.22, 138.44, 137.45, 132.43, 130.53, 130.32, 130.14, 130.04, 129.31, 127.20, 126.46, 122.09, 121.94, 120.57, 117.63, 117.19, 116.47, 115.72, 115.36, 114.27, 110.75, 110.44, 109.65, 109.32, 108.38, 21.61, 20.89; **HRMS** (APCI) calcd for $[M + H]^+$ 474.1489, found: 474.1484; **IR** (KBr) 2922, 2854, 1714, 1610, 1514, 1449, 1233, 1135, 921, 824 cm^{-1}.

Dioxaza[8]circulene **3k**

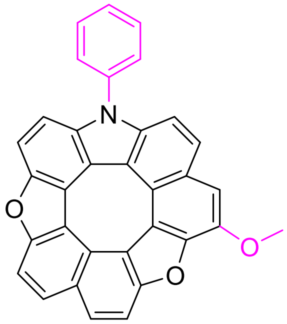

Method B: 68% yield, 48% FE, 5.0 h

Yellowish white compound; **m.p.** > 300 °C; **^{1}H-NMR** (600 MHz, $CDCl_3$) δ 8.24 (t, J = 9.3 Hz, 2H), 8.06 (dd, J = 8.9, 2.7 Hz, 2H), 7.96 (d, J = 8.9 Hz, 1H), 7.87 (d, J = 8.2 Hz, 1H), 7.72–7.76 (m, 4H), 7.67 (d, J = 8.2 Hz, 1H), 7.63–7.66 (m, 3H), 4.30 (s, 3H); **^{13}C-NMR** (150 MHz, $CDCl_3$) δ 155.73, 155.52, 151.00, 146.84, 142.84, 139.72, 137.82, 136.56, 130.58, 130.54, 130.19, 129.22, 128.93, 128.58, 127.49, 126.93, 122.34, 118.71, 118.05, 117.68, 117.21, 115.74, 114.82, 114.50, 110.99, 110.66, 110.21, 109.13, 108.57, 108.49, 56.29; **HRMS** (APCI) calcd for $[M + H]^+$ 476.1281, found: 476.1276; **IR** (KBr) 3061, 2960, 2922, 1611, 1595, 1497, 1261, 1169, 1042, 819 cm^{-1}.

Dioxaza[8]circulene **3l**

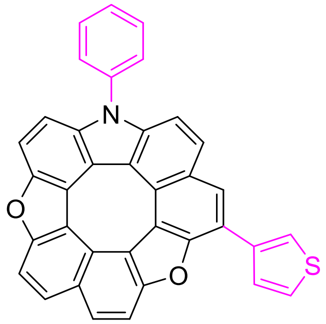

Method B: 70% yield, 50% FE, 5.0 h

Yellowish white compound; **m.p.** 286–287 °C; **^{1}H-NMR** (400 MHz, $CDCl_3$) δ 8.38 (s, 1H), 8.21 (dd, J = 3.0, 1.4 Hz, 1H), 8.09–8.14 (m, 3H), 7.94 (dd, J = 5.0, 0.9 Hz, 1H), 7.87 (d, J = 8.7 Hz, 1H), 7.85 (d, J = 8.7 Hz, 1H), 7.81 (d, J = 8.7 Hz, 1H), 7.75 (t, J = 7.6 Hz, 2H), 7.59–7.71 (m, 6H); **^{13}C-NMR (**175 MHz, $CDCl_3$) δ 155.74, 155.24, 152.97, 151.04, 140.83, 137.58, 137.02, 136.45, 130.70, 130.55, 130.43, 130.23, 129.11, 128.89, 128.70, 128.10, 127.38, 126.61, 125.56, 123.72, 122.06, 122.00, 117.83, 117.60, 117.36, 117.08, 115.63, 114.52, 114.43, 110.75, 110.47, 110.26, 108.53, 108.43; **HRMS** (APCI) calcd for $[M + H]^+$ 528.1053, found 528.1046; **IR** (KBr) 3046, 2925, 2853, 1731, 1618, 1595, 1498, 1267, 1177, 794 cm^{-1}.

Dioxaza[8]circulene **3m**

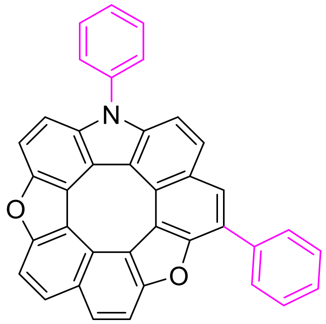

Method B: 65% yield, 47% FE, 5.0 h

Yellowish white compound; **m.p.** > 300 °C; **^{1}H-NMR** (700 MHz, $CDCl_3$) δ 8.42 (s, 1H), 8.30 (d, J = 8.6 Hz, 1H), 8.29 (d, J = 8.2 Hz, 1H), 8.27 (d, J = 9.0 Hz, 1H), 8.12 (dd, J = 8.0, 1.1 Hz, 2H), 8.06 (d, J = 8.6 Hz, 1H), 8.02 (d, J = 8.6 Hz, 1H), 7.93 (d, J = 8.6 Hz, 1H), 7.72–7.76 (m, 5H), 7.70 (d, J = 8.6 Hz, 1H), 7.64–7.68 (m, 3H), 7.53–7.56 (m, 1H); **^{13}C-NMR (**175 MHz, $CDCl_3$) δ 155.91, 155.57, 153.39, 151.17, 141.02, 137.57, 137.15, 136.59, 130.86, 130.75, 130.61, 130.56, 130.29, 130.26, 129.82, 128.95, 128.83, 128.78, 127.84, 127.48, 126.85, 123.33, 122.39, 122.22, 117.62, 117.23, 115.78, 114.67, 114.54, 111.08, 110.61, 110.37, 108.68, 108.55; **HRMS** (APCI) calcd for $[M + H]^+$ 522.1489, found: 522.1483; **IR** (KBr) 3052, 2924, 2852, 1620, 1593, 1502, 1271, 1183, 959, 819 cm^{-1}.

Dioxaza[8]circulene **3n**

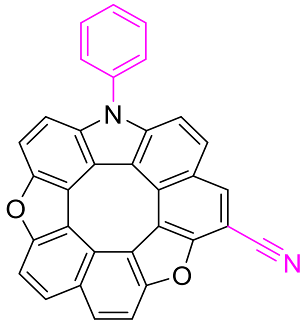

Method B: 10% yield, 8% FE, 5.0 h

Method C: 32% yield, 23% FE, 5.0 h

Yellowish white compound; **m.p.** > 300 °C; **^{1}H-NMR** (700 MHz, $CDCl_3$) δ 8.43 (s, 1H), 8.10–8.11 (m, 2H), 8.05 (d, *J* = 8.6 Hz, 1H), 7.86 (d, *J* = 8.6 Hz, 2H), 7.79–7.82 (m, 3H), 7.75–7.77 (m, 2H), 7.71 (d, *J* = 8.6 Hz, 2H), 7.64 (d, *J* = 8.6 Hz, 1H); **HRMS** (APCI) calcd for $[M + H]^+$ 471.1128, found: 471.1131; **IR** (KBr) 3061, 2923, 2855, 2310, 2228, 1620, 1597, 1501, 1269, 820 cm^{-1}.

Dioxaza[8]circulene **3o**

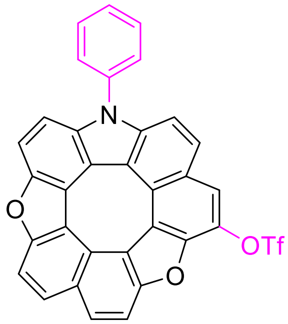

Method B: 20% yield, 14% FE, 5.0 h

Method C: 33% yield, 24% FE, 5.0 h

Yellowish white compound; **m.p.** 241–242 °C; **^{1}H-NMR** (700 MHz, $CDCl_3$) δ 8.23 (d, *J* = 8.6 Hz, 1H), 8.17 (d, *J* = 8.6 Hz, 1H), 8.15 (s, 1H), 8.10 (d, *J* = 9.0 Hz, 1H), 7.96 (d, *J* = 8.6 Hz, 1H), 7.91 (d, *J* = 8.2 Hz, 1H), 7.86 (d, *J* = 8.6 Hz, 1H), 7.78 (t, *J* = 7.7 Hz, 2H), 7.75 (d, *J* = 8.6 Hz, 1H), 7.73 (dd, *J* = 8.2, 1.3 Hz, 2H), 7.68 (t, *J* = 7.5 Hz, 1H), 7.65 (d, *J* = 8.6 Hz, 1H); **^{13}C-NMR** (175 MHz, $CDCl_3$) δ 155.87, 155.72, 151.22, 146.30, 141.10, 137.21, 136.61, 131.45, 131.24, 130.73, 130.39, 130.14, 129.04, 128.85, 127.50, 125.49, 122.14, 121.81, 121.26, 119.99, 119.11 (q, *J* = 321.22 Hz), 117.16, 116.88, 115.43, 114.41, 114.14, 111.39, 110.95, 110.75, 109.05, 108.94; **HRMS** (APCI) calcd for $[M + H]^+$ 594.0618, found: 594.0638; **IR** (KBr) 3060, 2923, 2854, 1732, 1595, 1503, 1211, 1137, 933, 819 cm^{-1}.

Dioxaza[8]circulene **3p**

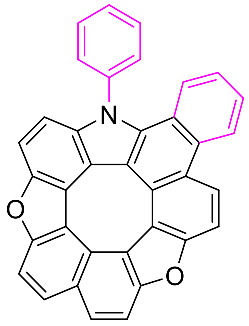

Method B: 61% yield, 54% FE, 4.0 h

Yellowish white compound; **m.p.** 285–286 °C; **^{1}H-NMR** (400 MHz, $CDCl_3$) δ 9.16 (d, J = 9.2 Hz, 1H), 8.90 (d, J = 8.7 Hz, 1H), 8.20 (t, J = 8.5 Hz, 2H), 7.98 (d, J = 9.2 Hz, 1H), 7.91 (t, J = 8.9 Hz, 2H), 7.77 (d, J = 8.7 Hz, 1H), 7.71–7.75 (m, 3H), 7.56–7.64 (m, 4H), 7.38 (d, J = 8.7 Hz, 1H), 7.29 (d, J = 8.2 Hz, 1H); **^{13}C-NMR** (175 MHz, $CDCl_3$) δ 155.94, 155.44, 155.34, 151.56, 141.25, 138.06, 136.84, 132.43, 130.70, 130.61, 130.31, 129.40, 129.27, 127.11, 126.22, 125.19, 124.96, 124.16, 124.01, 123.94, 123.56, 122.31, 121.58, 117.76, 117.19, 115.27, 114.81, 114.20, 110.75, 110.12, 109.35, 109.17, 108.16 (one carbon overlapped); **HRMS** (APCI) calcd for $[M + H]^+$ 496.1332, found:496.1326; **IR** (KBr) 3062, 2924, 2854, 1615, 1594, 1497, 1361, 1230, 1005, 818 cm^{-1}.

5. **Supplementary Method 5:** optimization and *general procedures for the one-pot synthesis of dioxaza[8]circulene from commercially available starting materials*

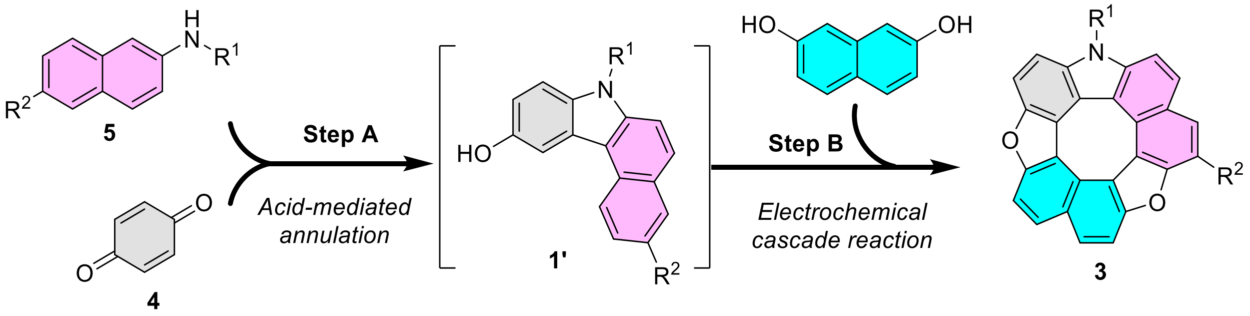

To establish the applicability of our method for concise synthesis, a one-pot protocol using commercially available substrates *p*-benzoquinone **4** and *N*-phenyl-2-naphthylamine **5** was tested. The acid-mediated annulation of these substrates afforded the corresponding benzo[*c*]carbazole **1'** *via* a tandem process of double Michael addition followed by double ring closure. Subsequently, this generated benzo[*c*]carbazole **1'** can undergo an electrochemical cascade reaction with 2,7-dihydroxynaphthalene **2** to form the corresponding circulenes **3**. To optimize the conditions of this one-pot protocol, we divided the overall reaction into two steps (**A** and **B**) and optimized each step individually before combining them.

- Optimization of the reaction conditions for step A

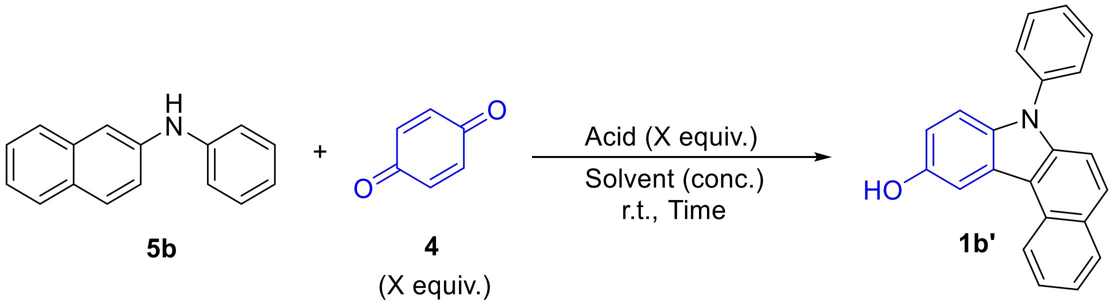

Table S4. Screening of different conditions for step A

Entry	Solvent	**4** (equiv.)	Time (h)	Acid (X equiv.)	NMR yield of **1b'** (%)[a]
1	DCM (0.1 M)	1.0 equiv.	3 h	H_3PO_4 (2.0 equiv.)	64
2	Acetone (0.1 M)	1.0 equiv.	3 h	H_3PO_4 (2.0 equiv.)	41
3	MeCN (0.1 M)	1.0 equiv.	3 h	H_3PO_4 (2.0 equiv.)	9
4	EtOAc (0.1 M)	1.0 equiv.	3 h	H_3PO_4 (2.0 equiv.)	40

5	MeOH (0.1 M)	1.0 equiv.	3 h	H_3PO_4 (2.0 equiv.)	19
6	DCM (0.1 M)	1.5 equiv.	3 h	H_3PO_4 (2.0 equiv.)	71
7	DCM (0.1 M)	2.0 equiv.	3 h	H_3PO_4 (2.0 equiv.)	74
8	DCM (0.1 M)	2.5 equiv.	3 h	H_3PO_4 (2.0 equiv.)	70
9	DCM (0.1 M)	3.0 equiv.	3 h	H_3PO_4 (2.0 equiv.)	71
10	DCM (0.1 M)	2.0 equiv.	3 h	--	N.D.
11	DCM (0.1 M)	2.0 equiv.	3 h	HCl (1.0 equiv.)	31
12	DCM (0.1 M)	2.0 equiv.	3 h	TFA (1.0 equiv.)	N.D.
13	DCM (0.1 M)	2.0 equiv.	3 h	H_3PO_4 (1.0 equiv.)	59
14	DCM (0.1 M)	2.0 equiv.	3 h	$MeCO_2H$ (1.0 equiv.)	N.D.
15	DCM (0.1 M)	2.0 equiv.	3 h	H_3PO_4 (3.0 equiv.)	48[b]
16	DCM (0.1 M)	2.0 equiv.	3 h	H_3PO_4 (4.0 equiv.)	51
17	DCM (0.01 M)	2.0 equiv.	3 h	H_3PO_4 (2.0 equiv.)	31%[b]

[a] Using 1,3,5-trimethoxybenzene as internal standard.
[b] Isolated yield.
N.D. means not detected.

- Optimization of the reaction conditions for step B

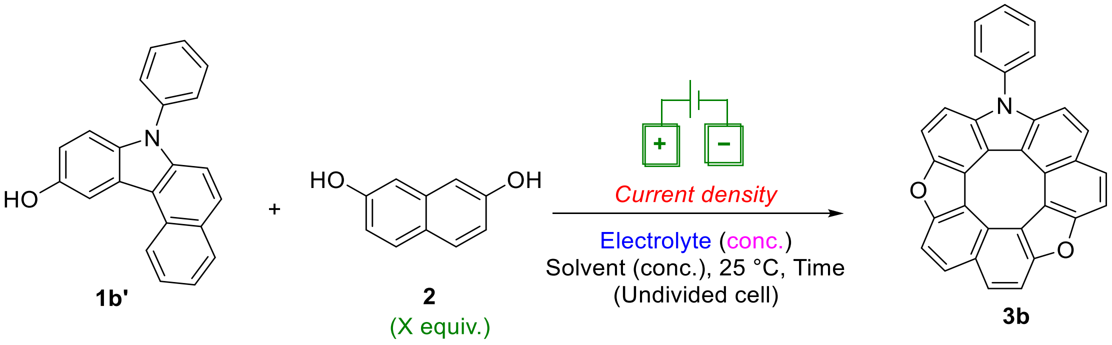

Table S5. Screening of different electrochemical conditions for step B

Entry	Electrodes	Current density	Electrolyte	**2** (X equiv.)	Solvent	Time (h)	yield of **3b** (%)[a]
1	Pt(+)-Pt(−)	0.51 mA/cm^2	Bu_4NClO_4 (0.2 M)	1.0	CH_2Cl_2 (6.5 mM)	12.0	38
2	Pt(+)-Pt(−)	0.51 mA/cm^2	Bu_4NClO_4 (0.2 M)	1.0	MeCN (6.5 mM)	12.0	N.D.
3	Pt(+)-Pt(−)	0.51 mA/cm^2	Bu_4NClO_4 (0.2 M)	1.0	Acetone (6.5 mM)	12.0	N.D.
4	Pt(+)-Pt(−)	0.51 mA/cm^2	Bu_4NClO_4 (0.2 M)	1.0	Toluene (6.5 mM)	12.0	N.R.
5	Pt(+)-FTO(−)	0.51 mA/cm^2	Bu_4NClO_4 (0.2 M)	1.0	CH_2Cl_2 (6.5 mM)	12.0	30
6	Pt(+)-C(−)	0.51 mA/cm^2	Bu_4NClO_4 (0.2 M)	1.0	CH_2Cl_2 (6.5 mM)	12.0	35
7	Pt(+)-Pt(−)	0.51 mA/cm^2	$LiClO_4$ (0.2 M)	1.0	CH_2Cl_2 (6.5 mM)	12.0	N.R.
8	Pt(+)-Pt(−)	0.51 mA/cm^2	Bu_4NPF_6 (0.2 M)	1.0	CH_2Cl_2 (6.5 mM)	12.0	20

9	Pt(+)-Pt(−)	0.51 mA/cm^2	Bu_4NClO_4 (0.2 M)	2.0	CH_2Cl_2 (6.5 mM)	12.0	45
10	Pt(+)-Pt(−)	0.51 mA/cm^2	Bu_4NClO_4 (0.2 M)	3.0	CH_2Cl_2 (6.5 mM)	12.0	35
11	Pt(+)-Pt(−)	0.51 mA/cm^2	Bu_4NClO_4 (0.3 M)	2.0	CH_2Cl_2 (6.5 mM)	12.0	32
12	Pt(+)-Pt(−)	0.51 mA/cm^2	Bu_4NClO_4 (0.1 M)	2.0	CH_2Cl_2 (6.5 mM)	12.0	57
13	Pt(+)-Pt(−)	0.51 mA/cm^2	Bu_4NClO_4 (0.05 M)	2.0	CH_2Cl_2 (6.5 mM)	17.0	trace
14	Pt(+)-Pt(−)	1.02 mA/cm^2	Bu_4NClO_4 (0.1 M)	2.0	CH_2Cl_2 (6.5 mM)	10.0	52
15	Pt(+)-Pt(−)	0.51 mA/cm^2	Bu_4NClO_4 (0.1 M)	2.0	CH_2Cl_2 (13 mM)	12.0	47

[a] Isolated yield.

N.D. means not detected.

N.R. means no reaction

After determining the optimal conditions for steps A and B, we proceeded by combining the two optimized conditions for both steps without any workup in between. This approach yielded the product **3b** from commercially available starting materials **4**, **5**, and **2** in just 15 hours, with only one workup required during the final purification at room temperature.

- General procedures for the one-pot synthesis

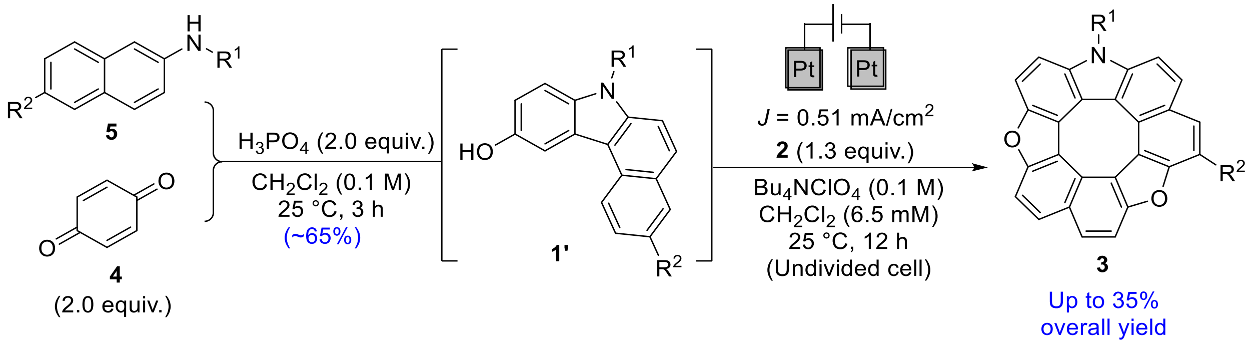

Initially, a mixture of **5** (22.0 mg, 0.1 mmol) and **4** (22.0 mg, 0.2 mmol) was dissolved in dry DCM (0.5 mL). *ortho*-Phosphoric acid (10 µL, 0.2 mmol) in DCM (0.5 mL) was then added dropwise to this mixture. The reaction was stirred for 3 hours at 25 °C. The resulting crude mixture was directly used in the next step without any workup. To this crude mixture, 2,7-dihydroxynaphthalene **2** (21 mg, 0.13 mmol, 1.3 equiv.) and tetrabutylammonium perchlorate (1.0 mmol) in CH_2Cl_2 (10 mL, 0.1 M) were added. The reaction mixture was then transferred to an undivided electrolysis cell equipped with two Pt electrodes connected to a DC power supply. At room temperature, constant current electrolysis with a current density of 0.51 mA/cm² was performed. After stirring for 12 hours, the electrolysis was stopped, and the crude products were purified by column chromatography (SiO_2, EtOAc/*n*-hexane), yielding **3** as a yellowish white solid.

Overall yield of **3b:** 30% yield, 27% FE, 15.0 h

Overall yield of **3a:** 28% yield, 20% FE, 27.0 h

Overall yield of **3c:** 35% yield, 26% FE, 27.0 h

Overall yield of **3f:** 19% yield, 14% FE, 27.0 h

Overall yield of **3m:** 17% yield, 12% FE, 27.0 h

Overall yield of **3n:** 14% yield, 9% FE, 27.0 h

6. **Supplementary Method 6:** *optimization of the organophotocatalytic arylation reaction towards diverse C-X bond formations*

- Optimization of the reaction parameters using **6** and **7** as model substrates

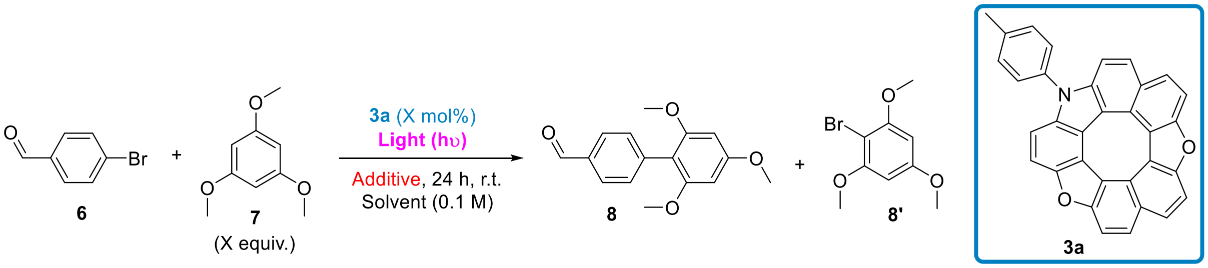

Table S6. Screening of different electrochemical conditions for step B

Entry	Solvent	**3a** (X mol%)	Light (hv)	**7** (X equiv.)	Additive	**8** (%) Yield	**8'** (%) Yield [b]
1	Acetone	10	365 nm	4.0 equiv.	--	6[a]	--
2	DCM	10	365 nm	4.0 equiv.	--	Trace	--
3	MeCN	10	365 nm	4.0 equiv.	--	2[a]	--
4	DMSO	10	365 nm	4.0 equiv.	--	13[a]	--
5	DMSO	10	310 nm	4.0 equiv.	--	N.D.	--
6	DMSO	10	340 nm	4.0 equiv.	--	4[a]	--
7	DMSO	10	385 nm	4.0 equiv.	--	6[a]	--
8	DMSO	10	395 nm	4.0 equiv.	--	5[a]	--
9	DMSO	w/o **3a**	w/o light	4.0 equiv.	--	N.D.	--
10	DMSO	w/o **3a**	365 nm	4.0 equiv.	--	N.D.	--
11	DMSO	10	365 nm	1.0 equiv.	--	8[a]	--
12	DMSO	10	365 nm	10.0 equiv.	--	28	--
13	DMSO	10	365 nm	15.0 equiv.	--	30	--
14	DMSO	10	365 nm	20.0 equiv.	--	38	42
15	DMSO	10	365 nm	25.0 equiv.	--	34	52
16	DMSO	10	365 nm	0.05 equiv.	--	Trace	42
17	DMSO	5	365 nm	20.0 equiv.	--	32	56
18	DMSO	15	365 nm	20.0 equiv.	--	38	37
19	DMSO	10	365 nm	20.0 equiv.	DIPEA (1.0 equiv.)	N.D.	N.D.
20	DMSO	10	365 nm	20.0 equiv.	Cs_2CO_3 (1.0 equiv.)	70	N.D.
21	DMSO	10	365 nm	20.0 equiv.	Cs_2CO_3 (2.0 equiv.)	70	N.D.

[a] NMR yield using dimethyl sulfone as an internal standard. [b] Tracking of **8'** yield started from entry 14.
N.D. means not detected.

- Variation from standard conditions using **6** and **7** as model substrates

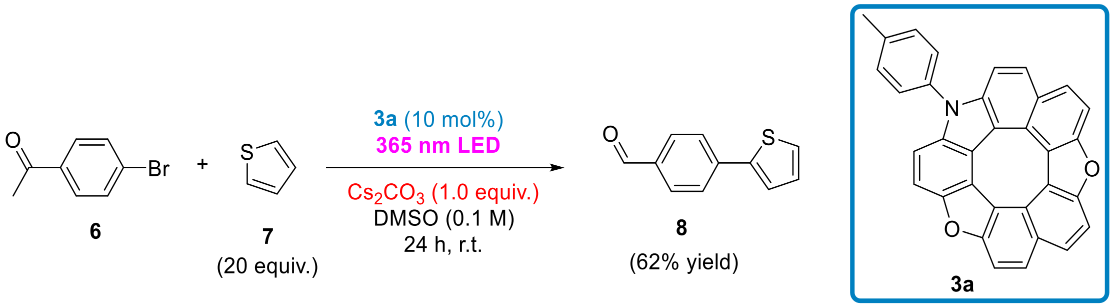

Table S7. Variation from standard conditions

Entry	**Variation from standard conditions**	**8** (%) Yield
1	25.0 equiv. of **7**	55
2	15.0 equiv. of **7**	46
3	Ratio of substrates (**6**/**7** = 20/1)	N.D.
4	catalyst **3a** (15 mol%)	60
5	catalyst **3a** (5 mol%)	30
6	catalyst **3a** (5 mol%), w/o Cs_2CO_3	20
7	Cs_2CO_3 (2.0 equiv.)	58
8	DIPEA instead of Cs_2CO_3	N.D.
9	DBU instead of Cs_2CO_3	N.D.
10	KH_2PO_4 instead of Cs_2CO_3	43
11	Acetone instead of DMSO	Trace
12	MeCN instead of DMSO	Trace
13	448 nm LEDs instead of 365 nm LEDs	Trace
14	340 nm LEDs instead of 365 nm LEDs	Trace
15	395 nm LEDs instead of 365 nm LEDs for 24 h	29
16	395 nm LEDs instead of 365 nm LEDs for 48 h	33
17	w/o catalyst	Trace
18	w/o base, w/o catalyst	Trace
19	w/o base	30
20	No light	N.R.

N.D. means not detected.
N.R. means no reaction.

7. **Supplementary Method 7:** *general procedures for the photocatalytic arylation reactions towards diverse C-X bond formations*

- General procedures

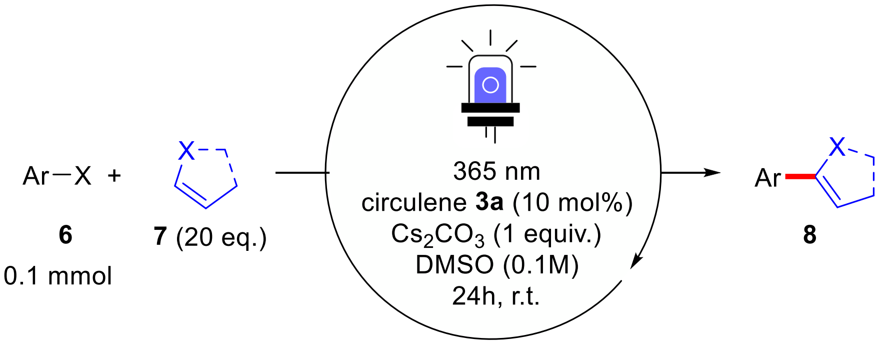

In a 10 mL oven-dried tube, aryl bromide (0.1 mmol), dioxaza[8]circulene **3a** (0.01 mmol, 10 mol%), and Cs_2CO_3 (0.1 mmol, 1.0 equiv.) were added. The reaction mixture was kept under a nitrogen atmosphere, and 1.0 mL of DMSO was added, followed by the addition of 20 equivalents of the radical trap. The tube was then transferred to a water bath to maintain the temperature around 25 °C. The reaction mixture was irradiated using a 365 nm LED and stirred for 24 hours. After completion, the reaction mixture was quenched with water and extracted with ethyl acetate (× 3 times). The combined organic layers were dried over anhydrous Na_2SO_4 and concentrated under reduced pressure. The residue was purified by column chromatography on silica gel (*n*-hexane/ethyl acetate) to yield the desired product.

- Spectral data

1-(4-(Thiophen-2-yl)phenyl)ethan-1-one **8a**

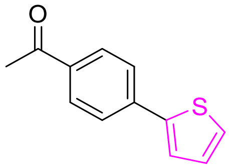

Yellowish white solid (62% yield, 30% yield w/o Cs_2CO_3). **m.p.** 97–98 °C; **^{1}H-NMR** (400 MHz, $CDCl_3$) δ 7.97 (d, *J* = 8.2 Hz, 2H), 7.70 (d, *J* = 8.7 Hz, 2H), 7.44 (dd, *J* = 3.7, 0.9 Hz, 1H), 7.37 (dd, *J* = 5.0, 1.4 Hz, 1H), 7.13 (dd, *J* = 5.0, 3.7 Hz, 1H), 2.62 (s, 3H). The spectral data of the obtained product were consistent with the previously reported values[4].

1-(4-(Furan-2-yl)phenyl)ethan-1-one 8b

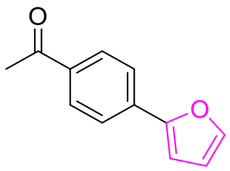

Pale brown solid (40% yield). **m.p.** 89–90 °C; **^{1}H-NMR** (400 MHz, $CDCl_3$) δ 7.98 (d, J = 8.7 Hz, 2H), 7.75 (d, J = 8.2 Hz, 2H), 7.53 (d, J = 1.8 Hz, 1H), 6.81 (d, J = 2.7 Hz, 1H), 6.52 (dd, J = 3.2, 1.8 Hz, 1H), 2.62 (s, 3H). The spectral data of the obtained product were consistent with the previously reported values[5].

1-(4-(1-Methyl-1*H*-pyrrol-2-yl)phenyl)ethan-1-one 8c

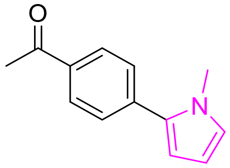

Pale brown solid (97% yield, 80% yield w/o Cs_2CO_3). **m.p.** 46–47 °C; **^{1}H-NMR** (400 MHz, $CDCl_3$) δ 7.99 (d, J = 7.8 Hz, 2H), 7.50 (d, J = 8.2 Hz, 2H), 6.77 (s, 1H), 6.35-6.36 (m, 1H), 6.23 (t, J = 3.2 Hz, 1H), 3.73 (s, 3H), 2.63 (s, 3H). The spectral data of the obtained product were consistent with the previously reported values[6].

1-(2',4',6'-Trimethoxy-[1,1'-biphenyl]-4-yl)ethan-1-one 8d

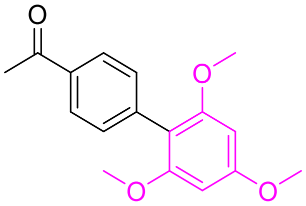

Brown solid (92% yield, 70% yield w/o Cs_2CO_3). **m.p.** 116–117 °C; **^{1}H-NMR** (400 MHz, $CDCl_3$) δ 7.98 (d, J = 8.2 Hz, 2H), 7.44 (d, J = 8.2 Hz, 2H), 6.23 (s, 2H), 3.87 (s, 3H), 3.73 (s, 6H), 2.62 (s, 3H). The spectral data of the obtained product were consistent with the previously reported values[7].

1-(2',4',6'-Trimethyl-[1,1'-biphenyl]-4-yl)ethan-1-one **8e**

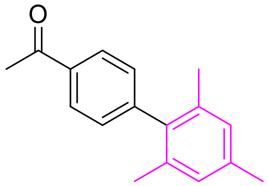

White solid (76% yield). **m.p.** 75–76 °C; **^{1}H-NMR** (400 MHz, $CDCl_3$) δ 8.02 (d, *J* = 8.2 Hz, 2H), 7.25 (d, *J* = 8.2 Hz, 2H), 6.95 (s, 2H), 2.65 (s, 3H), 2.34 (s, 3H), 1.99 (s, 6H). The spectral data of the obtained product were consistent with the previously reported values[8].

2',4',6'-Trimethyl-[1,1'-biphenyl]-2-carbonitrile **8f**

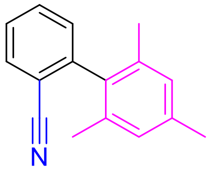

Yellow solid (45% yield). **m.p.** 71–72 °C; **^{1}H-NMR** (400 MHz, $CDCl_3$) δ 7.76 (dd, *J* = 7.8, 0.9 Hz, 1H), 7.65 (td, *J* = 7.7, 1.2 Hz, 1H), 7.45 (td, *J* = 7.7, 1.2 Hz, 1H), 7.28 (dd, *J* = 7.8, 0.9 Hz, 1H), 6.97 (s, 2H), 2.33 (s, 3H), 1.98 (s, 6H). The spectral data of the obtained product were consistent with the previously reported values[9].

2',4',6'-Trimethoxy-[1,1'-biphenyl]-2-carbonitrile **8g**

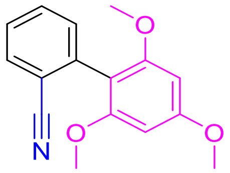

Pale yellow solid (91% yield). **m.p.** 147–148 °C; **^{1}H-NMR** (600 MHz, $CDCl_3$) δ 7.70 (dd, *J* = 7.8, 1.4 Hz, 1H), 7.58 (td, *J* = 7.8, 1.4 Hz, 1H), 7.35-7.41 (m, 2H), 6.24 (s, 2H), 3.87 (s, 3H), 3.76 (s, 6H). The spectral data of the obtained product were consistent with the previously reported values[6].

Ethyl 4-(1-methylpyrrol-2-yl)benzoate **8h**

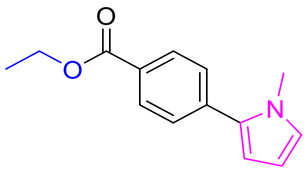

Brownish white solid (77% yield, 60% yield w/o Cs_2CO_3). **m.p.** 67–68 °C; **^{1}H-NMR** (400 MHz, $CDCl_3$) δ 8.06 (d, J = 8.7 Hz, 2H), 7.47 (d, J = 8.5 Hz, 2H), 6.76 (dd, J = 2.8, 2.1 Hz, 1H), 6.33 (dd, J = 3.4, 1.4 Hz, 1H), 6.22 (dd, J = 3.4, 2.8 Hz, 1H), 4.39 (q, J = 7.0 Hz, 2H), 3.71 (s, 3H), 1.41 (t, J = 7.1 Hz, 3H). The spectral data of the obtained product were consistent with the previously reported values[10].

4-(1-Methyl-1*H*-pyrrol-2-yl)benzaldehyde **8i**

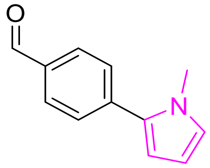

Yellow oil (74% yield). **^{1}H-NMR** (600 MHz, $CDCl_3$) δ 10.02 (s, 1H), 7.90 (d, J = 8.2 Hz, 2H), 7.57 (d, J = 8.2 Hz, 2H), 6.79 (dd, J = 2.8, 2.1 Hz, 1H), 6.39 (dd, J = 3.4, 1.4 Hz, 1H), 6.24 (dd, J = 3.4, 2.8 Hz, 1H), 3.74 (s, 3H). The spectral data of the obtained product were consistent with the previously reported values[6].

2',4',6'-Trimethoxy-[1,1'-biphenyl]-4-carbaldehyde **8j**

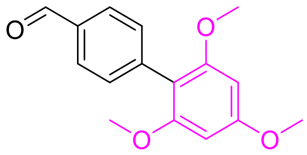

Yellow solid (70% yield, 38% yield w/o Cs_2CO_3, 52% yield using 4-chlorobanzaldehyde as substrate in the presence of Cs_2CO_3). **m.p.** 135–136 °C; **^{1}H-NMR** (400 MHz, $CDCl_3$) δ 10.02 (s, 1H), 7.89 (d, J = 8.5 Hz, 2H), 7.52 (d, J = 8.2 Hz, 2H), 6.24 (s, 2H), 3.88 (s, 3H), 3.74 (s, 6H). The spectral data of the obtained product were consistent with the previously reported values[7].

2'-Methoxy-[1,1'-binaphthalene]-2-carbaldehyde **8k**

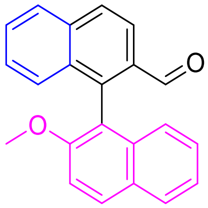

Yellow solid (20% yield). **m.p.** 149–150 °C; **^{1}H-NMR** (600 MHz, $CDCl_3$) δ 9.67 (s, 1H), 8.16 (d, J = 8.9 Hz, 1H), 8.07 (d, J = 8.9 Hz, 1H), 8.02 (d, J = 8.9 Hz, 1H), 7.97 (d, J = 8.2 Hz, 1H), 7.90 (d, J = 8.2 Hz, 1H), 7.58–7.60 (m, 1H), 7.46 (d, J = 8.9 Hz, 1H), 7.31–7.36 (m, 3H), 7.24 (td, J = 7.6, 1.4 Hz, 1H), 6.95 (d, J = 8.2 Hz, 1H), 3.77 (s, 3H). The spectral data of the obtained product were consistent with the previously reported values[11].

2-(2-Methoxynaphthalen-1-yl)benzaldehyde **8l**

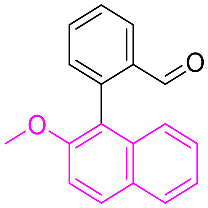

Yellow solid (47% yield). **m.p.** 62–63 °C; **^{1}H-NMR** (400 MHz, $CDCl_3$) δ 9.62 (s, 1H), 8.12 (dd, J = 7.8, 0.9 Hz, 1H), 7.97 (d, J = 8.7 Hz, 1H), 7.85–7.87 (m, 1H), 7.73 (td, J = 7.4, 1.5 Hz, 1H), 7.58 (t, J = 7.6 Hz, 1H), 7.30–7.39 (m, 5H), 3.83 (s, 3H). The spectral data of the obtained product were consistent with the previously reported values[12].

9-(2-Methoxynaphthalen-1-yl)phenanthrene **8m**

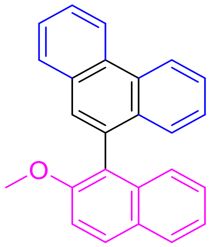

Yellowish white solid (20% yield, 30% yield using 9-Iodophenanthrene). **m.p.** 175–176 °C; **^{1}H-NMR** (600 MHz, $CDCl_3$) δ 8.81–8.80 (m, 2H), 8.01 (d, J = 8.9 Hz, 1H), 7.89 (dd, J = 8.3, 2.8 Hz, 2H), 7.69–7.73 (m, 2H), 7.61–7.66 (m, 2H), 7.48 (d, J = 9.2 Hz, 1H), 7.38–7.39 (m, 2H), 7.31–7.36 (m, 1H), 7.28 (d, J = 7.8 Hz, 1H),

7.19–7.24 (m, 1H), 3.78 (s, 3H). The spectral data of the obtained product were consistent with the previously reported values[13].

2-(2,4,6-Trimethoxyphenyl)naphthalene **8n**

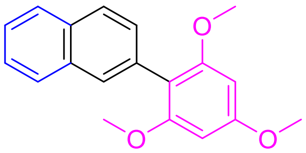

Yellowish white solid (53% yield). **m.p.** 105–106 °C; **^{1}H-NMR** (400 MHz, $CDCl_3$) δ 7.80–7.85 (m, 4H), 7.42–7.46 (m, 3H), 6.27 (s, 2H), 3.89 (s, 3H), 3.73 (s, 6H). The spectral data of the obtained product were consistent with the previously reported values[14].

1-Methyl-2-(*p*-tolyl)-1*H*-pyrrole **8o**

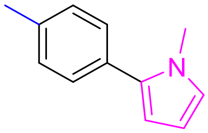

Yellow oil (40% yield using 4-Iodotoluene). **^{1}H-NMR** (400 MHz, $CDCl_3$) δ 7.30 (d, J = 8.2 Hz, 2H), 7.21 (d, J = 8.2 Hz, 2H), 6.71 (t, J = 2.3 Hz, 1H), 6.20 (d, J = 1.8 Hz, 2H), 3.66 (s, 3H), 2.39 (s, 3H). **HRMS** (APCI) calcd for $[M + H]^+$ 172.1121, found: 172.1122. The spectral data of the obtained product were consistent with the previously reported values[10].

4-(Furan-2-yl)benzaldehyde **8p**

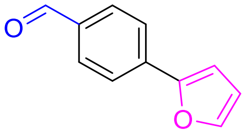

Yellow solid (31% yield, 29% yield using 4-iodobenzaldyehde). **m.p.** 40–41 °C; **^{1}H-NMR** (400 MHz, $CDCl_3$) δ 10.00 (s, 1H), 7.90 (d, J = 8.2 Hz, 2H), 7.82 (d, J = 8.2 Hz, 2H), 7.55 (d, J = 1.8 Hz, 1H), 6.85 (d, J = 3.7 Hz, 1H), 6.54 (dd, J = 3.7, 1.8 Hz, 1H). The spectral data of the obtained product were consistent with the previously reported values[15].

4-(Phenylsulfonyl)benzaldehyde **8q**

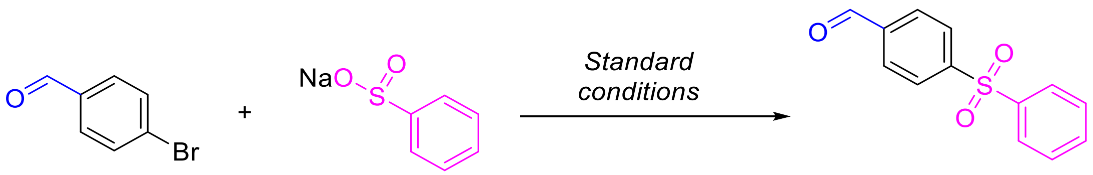

Yellow solid (37% yield). **m.p.** 89–90 °C; **^{1}H-NMR** (600 MHz, $CDCl_3$) δ 10.08 (s, 1H), 8.12 (d, J = 8.2 Hz, 2H), 8.01 (d, J = 7.8 Hz, 2H), 7.97 (d, J = 8.2 Hz, 2H), 7.59–7.64 (m, 1H), 7.54 (t, J = 7.8 Hz, 2H). The spectral data of the obtained product were consistent with the previously reported values[7].

1-(4-(Phenylsulfonyl)phenyl)ethan-1-one **8r**

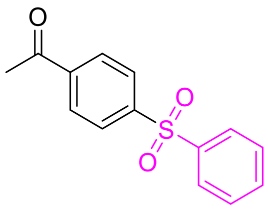

Yellow solid (66% yield, 40% yield w/o Cs_2CO_3). **m.p.** 123–124 °C; **^{1}H-NMR** (600 MHz, $CDCl_3$) δ 8.03–8.06 (m, 4H), 7.95–7.97 (m, 2H), 7.60 (tt, J = 7.9, 1.3 Hz, 1H), 7.52–7.55 (m, 2H), 2.62 (s, 3H). The spectral data of the obtained product were consistent with the previously reported values[16].

2-(Phenylsulfonyl)benzonitrile **8s**

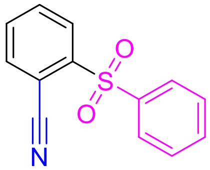

Yellowish white solid (45% yield). **m.p.** 71–72 °C; **^{1}H-NMR** (400 MHz, $CDCl_3$) δ 8.35 (dd, J = 8.0, 1.4 Hz, 1H), 8.09 (d, J = 7.3 Hz, 2H), 7.79–7.82 (m, 2H), 7.63–7.71 (m, 2H), 7.56 (t, J = 7.6 Hz, 2H). The spectral data of the obtained product were consistent with the previously reported values[7].

4-(Phenylsulfonyl)benzonitrile **8t**

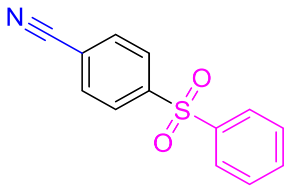

Pale brown solid (75% yield). **m.p.** 134–135 °C; **^{1}H-NMR** (600 MHz, $CDCl_3$) δ 8.05 (d, J = 8.2 Hz, 2H), 7.95 (d, J = 6.9 Hz, 2H), 7.80 (d, J = 8.2 Hz, 2H), 7.63 (t, J = 7.6 Hz, 1H), 7.55 (t, J = 7.6 Hz, 2H). The spectral data of the obtained product were consistent with the previously reported values[7].

4-((4-Chlorophenyl)sulfonyl)benzonitrile **8u**

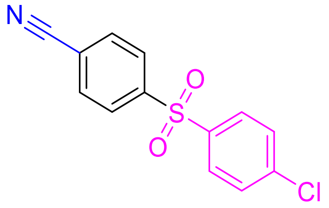

Pale yellow solid (40% yield). **m.p.** 162–163 °C; **^{1}H-NMR** (600 MHz, $CDCl_3$) δ 8.04 (d, J = 8.2 Hz, 2H), 7.88 (d, J = 8.7 Hz, 2H), 7.81 (d, J = 8.2 Hz, 2H), 7.52 (d, J = 8.7 Hz, 2H). The spectral data of the obtained product were consistent with the previously reported value[7].

2-(4,4,5,5-Tetramethyl-1,3,2-dioxaborolan-2-yl)benzonitrile **8v**

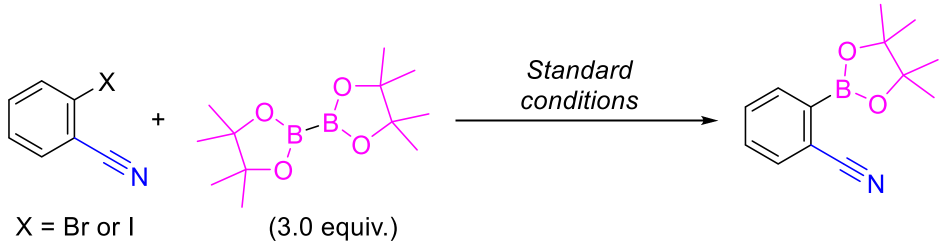

Yellow solid (13% NMR yield, 33% NMR yield using 2-iodobenzonitrile; 1,3,5-trimethoxybenzne was used as an internal standard). **m.p.** 77–78 °C; **^{1}H-NMR** (600 MHz, $CDCl_3$) δ 7.88 (d, J = 6.9 Hz, 1H), 7.70 (d, J = 7.6 Hz, 1H), 7.57 (td, J = 7.6, 1.4 Hz, 1H), 7.52 (td, J = 7.5, 1.6 Hz, 1H), 1.38 (s, 12H). The spectral data of the obtained product were consistent with the previously reported values[6,17].

2,3',4,5',6-Pentamethoxy-1,1'-biphenyl **8w**

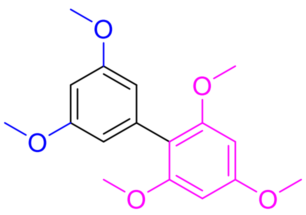

Yellow solid (20% yield). **m.p.** 130–131 °C; **^{1}H-NMR** (400 MHz, $CDCl_3$) δ 6.49 (d, *J* = 2.3 Hz, 2H), 6.42 (t, *J* = 2.3 Hz, 1H), 6.22 (s, 2H), 3.86 (s, 3H), 3.79 (s, 6H), 3.73 (s, 6H). The spectral data of the obtained product were consistent with the previously reported values[7].

5-Fluoro-2',4',6'-trimethoxy-[1,1'-biphenyl]-2-carbonitrile **8x**

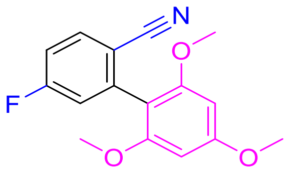

Yellowish white solid (60% yield using 2-chloro-4-fluorobenzonitrile as a substrate). **m.p.** 102–103 °C; **^{1}H-NMR** (400 MHz, $CDCl_3$) δ 7.69 (dd, *J* = 8.6, 5.6 Hz, 1H), 7.11 (dd, *J* = 9.6, 2.7 Hz, 1H), 7.06 (td, *J* = 8.4, 2.4 Hz, 1H), 6.22 (s, 2H), 3.87 (s, 3H), 3.77 (s, 6H). **HRMS** (APCI) calcd for $[M + H]^+$ 288.1030, found: 288.1027. The spectral data of the obtained product were consistent with the previously reported values[18].

Diethyl (2-cyanophenyl)phosphonate **8y**

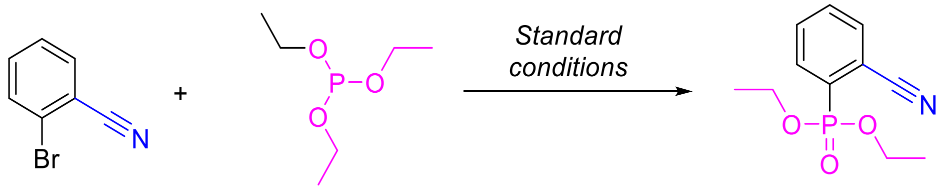

Yellow oil (85% yield). **^{1}H-NMR** (400 MHz, $CDCl_3$) δ 8.12 (qd, *J* = 7.2, 1.5 Hz, 1H), 7.79–7.82 (m, 1H), 7.63–7.72 (m, 2H), 4.15–4.32 (m, 4H), 1.38 (t, *J* = 7.1 Hz, 6H). The spectral data of the obtained product were consistent with the previously reported values[6].

Diethyl *p*-tolylphosphonate **8z**

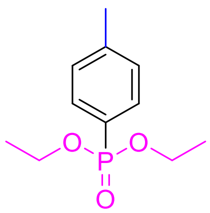

Colorless oil (79% yield using 4-iodotoluene as a substrate). **^{1}H-NMR** (400 MHz, $CDCl_3$) δ 7.70 (dd, J = 13.1, 8.0 Hz, 2H), 7.26–7.29 (m, 2H), 4.02–4.16 (m, 4H), 2.40 (s, 3H), 1.31 (t, J = 6.9 Hz, 6H). The spectral data of the obtained product were consistent with the previously reported values[19].

2',4',6'-Trimethoxy-3,5-dimethyl-[1,1'-biphenyl]-4-ol **8aa**

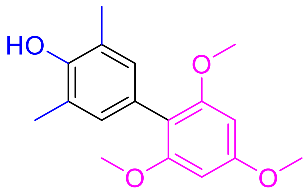

White solid (30% yield). **m.p.** 149–150 °C; **^{1}H-NMR** (400 MHz, $CDCl_3$) δ 6.93 (s, 2H), 6.21 (s, 2H), 4.55 (s, 1H), 3.86 (s, 3H), 3.72 (s, 6H), 2.26 (s, 6H); **HRMS** (APCI) calcd for $[M + H]^+$ 289.1434, found: 289.1431. The spectral data of the obtained product were consistent with the previously reported values[20].

3-(1-Methyl-1*H*-pyrrol-2-yl)pyridine **8ab**

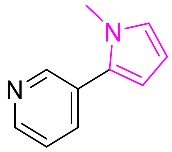

brown oil (32% yield). **^{1}H-NMR** (400 MHz, $CDCl_3$) δ 8.69 (d, J = 2.3 Hz, 1H), 8.53 (dd, J = 5.0, 0.9 Hz, 1H), 7.71 (dt, J = 7.8, 1.8 Hz, 1H), 7.32 (dd, J = 8.0, 4.8 Hz, 1H), 6.77 (t, J = 2.3 Hz, 1H), 6.30 (dd, J = 3.7, 3.2 Hz, 1H), 6.23 (t, J = 3.2 Hz, 1H), 3.68 (s, 3H). The spectral data of the obtained product were consistent with the previously reported values[21].

2-Bromo-1,3,5-trimethoxybenzene **8'**

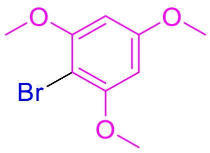

White solid. **m.p.** 93–94 °C; **^{1}H-NMR** (400 MHz, $CDCl_3$) *δ* 6.18 (s, 2H), 3.88 (s, 6H), 3.82 (s, 3H). **HRMS** (APCI) calcd for $[M + H]^+$ 246.9964, found: 246.9966. The spectral data of the obtained product were consistent with the previously reported values[22].

1-(4-((2,2,6,6-Tetramethylpiperidin-1-yl)oxy)phenyl)ethan-1-one **9**

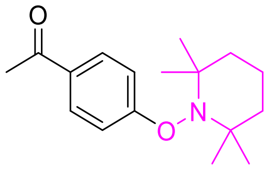

White solid (50% yield). **m.p.** 63–64 °C; **^{1}H-NMR** (400 MHz, $CDCl_3$) *δ* 7.87 (d, *J* = 9.6 Hz, 2H), 7.21–7.25 (m, 2H), 2.54 (s, 3H), 1.56–1.65 (m, 6H), 1.23 (s, 6H), 0.99 (s, 6H); **HRMS** (APCI) calcd for $[M + H]^+$ 276.1958, found, 276.1957. The spectral data of the obtained product were consistent with the previously reported values[23].

8. **Supplementary Note 1:** *plausible reaction mechanism of electrochemical cascade synthesis of dioxaza[8]circulenes*

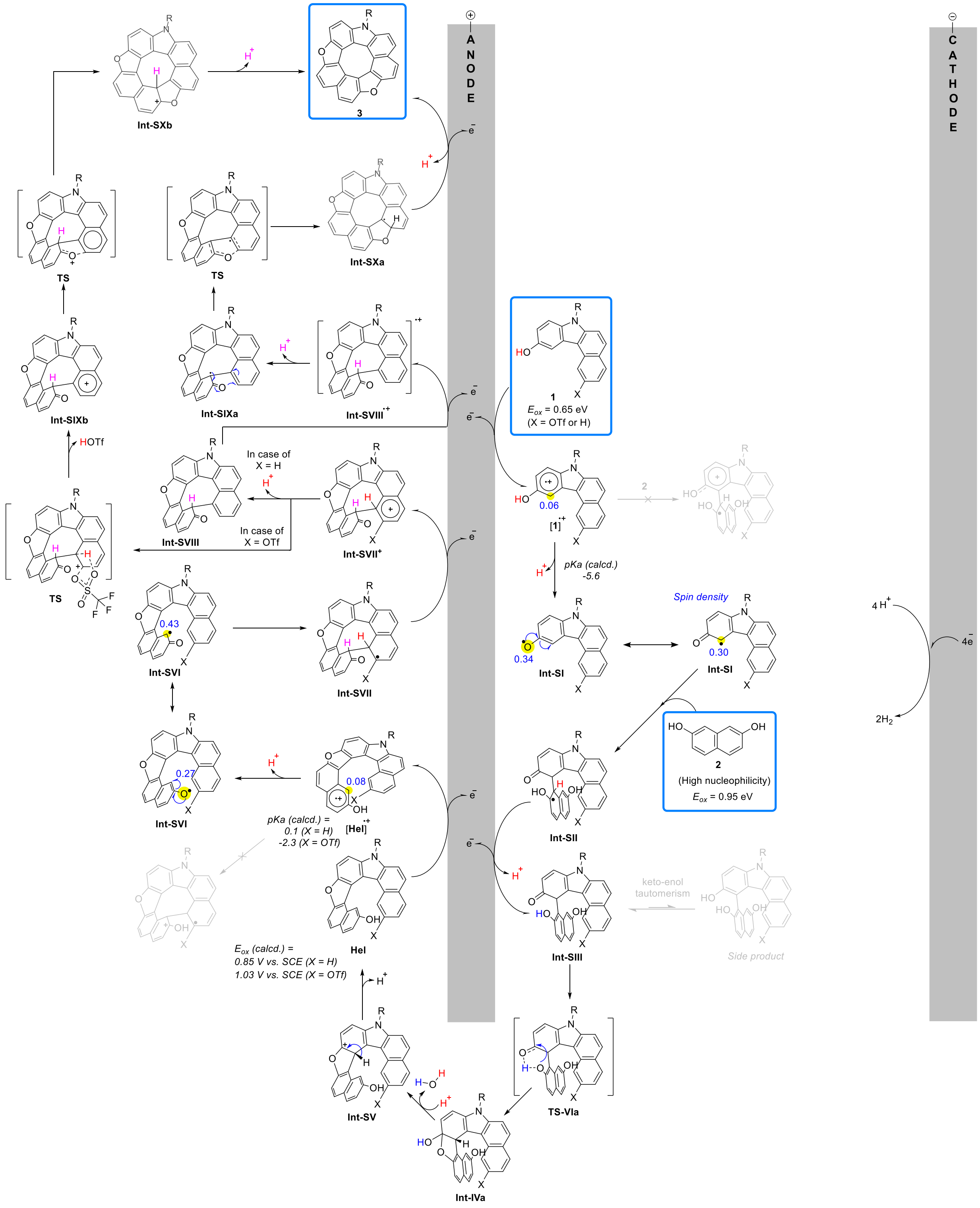

DFT Calculations and Mechanistic Insights into the Sequential Electrochemical Oxidation Reaction

General information and considerations

In our study of the reaction mechanism, the molecular geometries of all stationary points were optimized using Density Functional Theory (DFT) at the B3LYP level with the 6-31G+(d,p) basis set, incorporating the IEFPCM model to account for solvation in dichloromethane[24-26]. Grimme's dispersion correction with the original D3 damping function was applied to enhance the accuracy of the optimized structures[27]. To reduce computational cost, substituents and triflate (OTf) groups were omitted. The nature of each stationary point was confirmed through vibrational frequency analysis. All global minima were verified by the absence of imaginary frequencies, while all transition states were confirmed by the presence of a single imaginary frequency. Additionally, an Intrinsic Reaction Coordinate (IRC) analysis was performed to ensure that each transition state correctly connects the corresponding reactant and product minima along the reaction pathway[28]. Given that our sequential mechanistic process involves both charged and neutral species, careful consideration was given to comparing their relative energies. In DFT-based mechanistic studies, direct comparisons between charged and neutral intermediates can be misleading due to differences in reference states, solvation effects, and charge stabilization[29,30]. To address this, we followed an approach similar to that of King in his study of the Scholl reaction, where charged and neutral species were compared within their respective potential energy surfaces[31,32]. We applied appropriate energy corrections and solvation models to minimize errors and supplemented our mechanistic investigation with redox potential analysis and pKa calculations to support our hypotheses[33].

Mechanistic Insights into the Sequential Electrochemical Oxidation Reaction

- To elucidate the reaction mechanism, we first wanted to determine whether the process proceeds *via* neutral radical intermediates or radical cation intermediates. Based on studies by Siegfried R. Waldvogel[34-40], the initial oxidation of carbazoles **1** occurs at the anode due to its lowest oxidation potential compared to 2,7-dihydroxy naphthalene **2**. This oxidation is immediately followed by deprotonation, as the radical cation $[\mathbf{1}]^{\bullet+}$ is highly acidic. To support this hypothesis, we assessed the acidity (pKa values) of the generated radical cation in comparison to that of carbazole **1** using DFT calculations. The results aligned with the proposed mechanism by Waldvogel, reinforcing the role of radical cation intermediates in the reaction pathway.

- By applying the previously established equation with Custodio approach to minimize calculation errors, we could predict theoretically the acidity of neutral versus the cationic radical species of carbazoles[41].

$$\boldsymbol{pKa = \frac{G\ sol\ (H^{+}) + G\ sol\ (conjugate\ base^{-}) -\ G\ sol\ (Acid}{2.3\ RT}}$$

T(K)	298
Etotal, H (kcal/mol)	−269.1950859
R (kcal/Kmol)	0.001987

Molecule	$E_{(total)}$ Hartree	$E_{(total)}$ kcal/mol	*ΔG* (kcal/mol)	pKa
**1b’-OH**	−977.18835	−613194.4843	31.65155396	23.2
**1b’-O⁻**	−976.70892	−612893.6377		
$[\mathbf{1b'}]^{\bullet+}$	−976.98605	−613067.5392	−7.56775854	−5.6
$[\mathbf{1b'}]^{\bullet}$	−976.56912	−612805.9119		

Optimized at the UB3LYP/6-31G+(d,p) level of theory with IEPCM model as solvation of DCM. Grimme’s dispersion with the original D3 damping function was applied as empirical dispersion correction to the optimized structures.

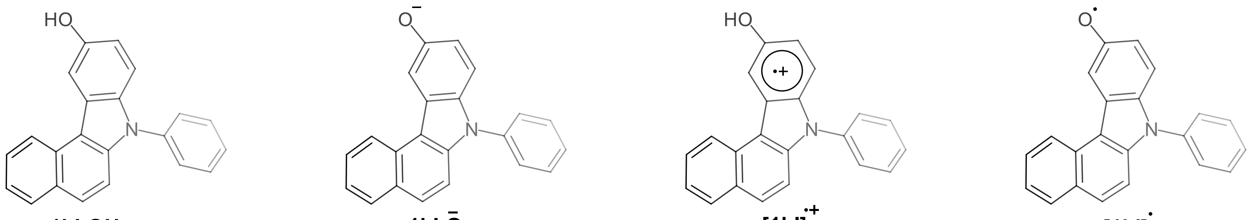

1b'-OH Carbazole | **1b'-Ō** Conjugate base | $[\mathbf{1b'}]^{\bullet+}$ Radical cation | $[\mathbf{1b'}]^{\bullet}$ Neutral radical

- Once these radical cations [**1**]$^{\bullet+}$ are generated, two possible pathways emerge: either proton release due to their high acidity (pKa ~ -5.6) or radical-anion coupling with 2,7-dihydroxynaphthalene **2**[42,43].

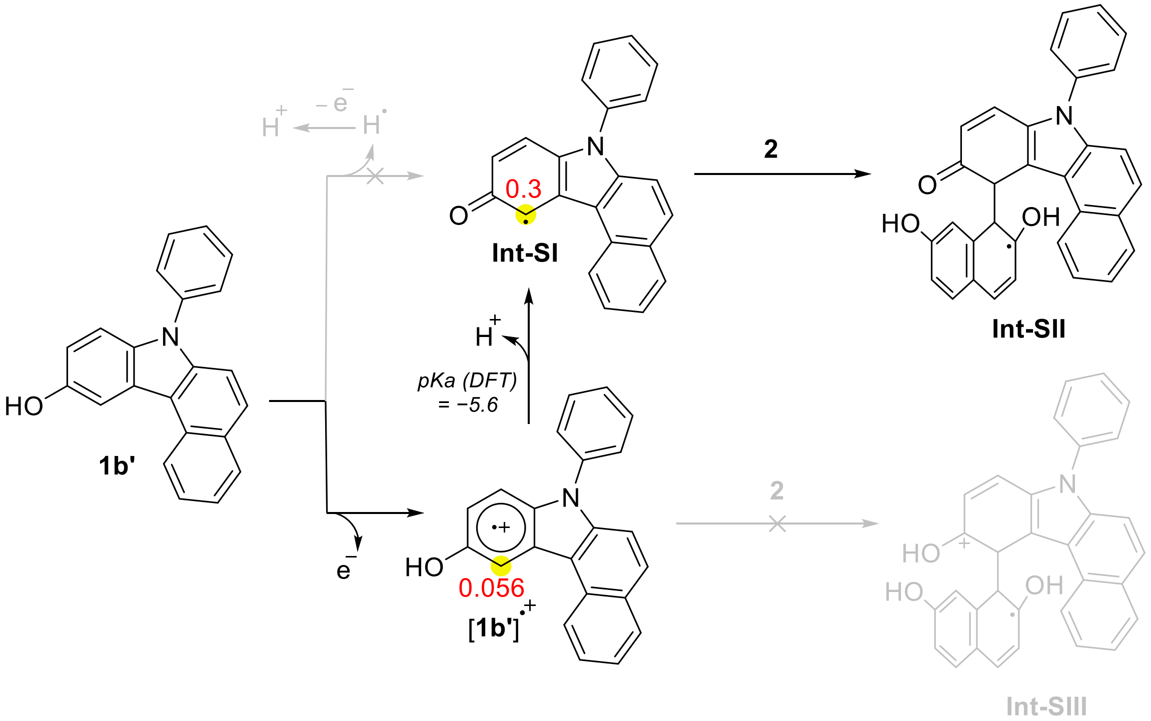

- To investigate this, we analyzed the spin densities of both the radical cation species [**1**]$^{\bullet+}$ and their corresponding neutral radicals **Int-SI** after proton release[44]. These studies revealed a significant difference in spin density at the key reactive position, suggesting that the radical cation [**1**]$^{\bullet+}$ cannot directly undergo coupling with **2** . Instead, it must first convert into the corresponding intermediate, **Int-SI**, before proceeding with the reaction.

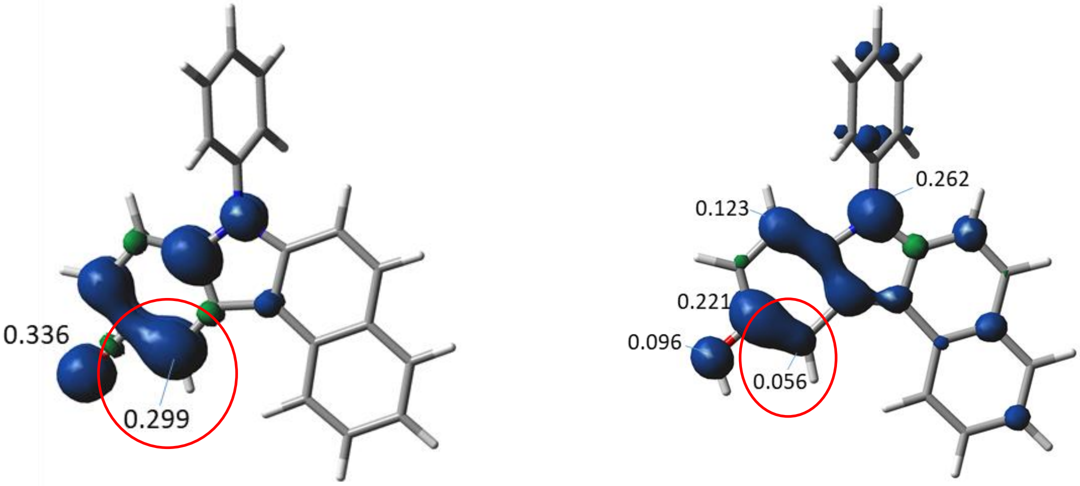

Int-SI [**1**]$^{\bullet+}$

Mulliken spin density contour maps calculated at the UB3LYP/6-31G+(d,p)/IEFPCM=DCM. Grimme's dispersion with the original D3 damping function was applied as empirical dispersion correction to the optimized structures. (isoval = 0.003).

Energy profiles, in kcal/mol, for the sequential electrochemical synthesis of helicenes

The mechanistic insights were derived from the energy profiles of all intermediates and transition states, as well as from pKa values, redox potentials, and spin density analysis. Based on these findings, we propose that the reaction proceeds *via* the previously suggested pathway. Our calculations indicate that among the different possible configurations, pathway A—comprising intermediates **Int-SIIa** to **Int-SVa** and transition states **TS-IIIa** to **TS-VIIIa**—is the most favorable, requiring the least energy. The calculations—along with supporting pKa and redox potential DFT-based predictions—suggest that direct H-atom removal from the **Int-SII** radical is likely less favorable than an alternative pathway involving oxidation followed by deprotonation, a process facilitated by the perchlorate anion. The electron transfer step was evaluated using Marcus theory (see next section for details), and our data also indicate that all deprotonation steps in this mechanism are further facilitated by the perchlorate anion present in the supporting electrolyte.

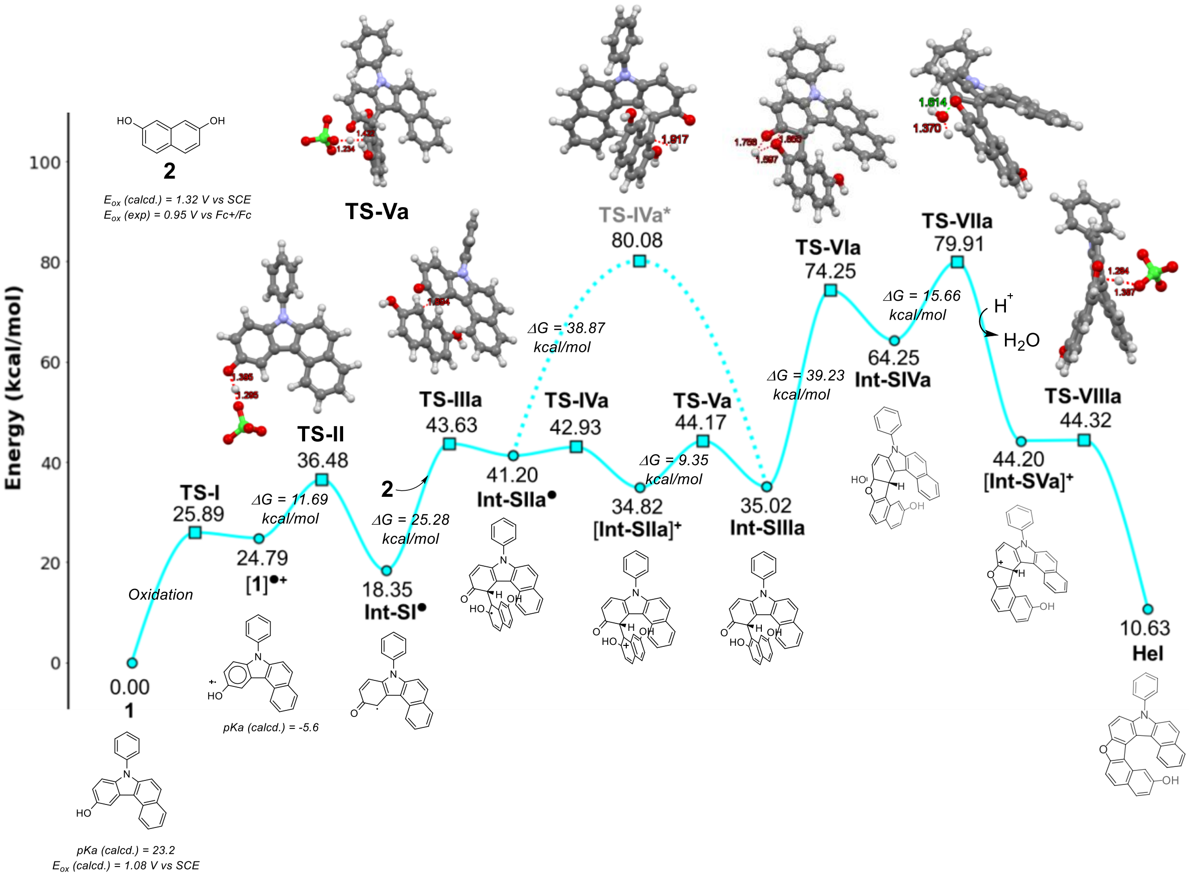

Calculated at the UB3LYP/6-31G+(d,p)/IEFPCM=DCM. Grimme's dispersion with the original D3 damping function was applied as empirical dispersion correction to the optimized structures. All global minima were verified by the absence of imaginary frequencies, while all transition states were confirmed by the presence of a single imaginary frequency. Additionally, an Intrinsic Reaction Coordinate (IRC) analysis was performed to ensure that each transition state correctly connects the corresponding reactant and product minima along the reaction pathway.

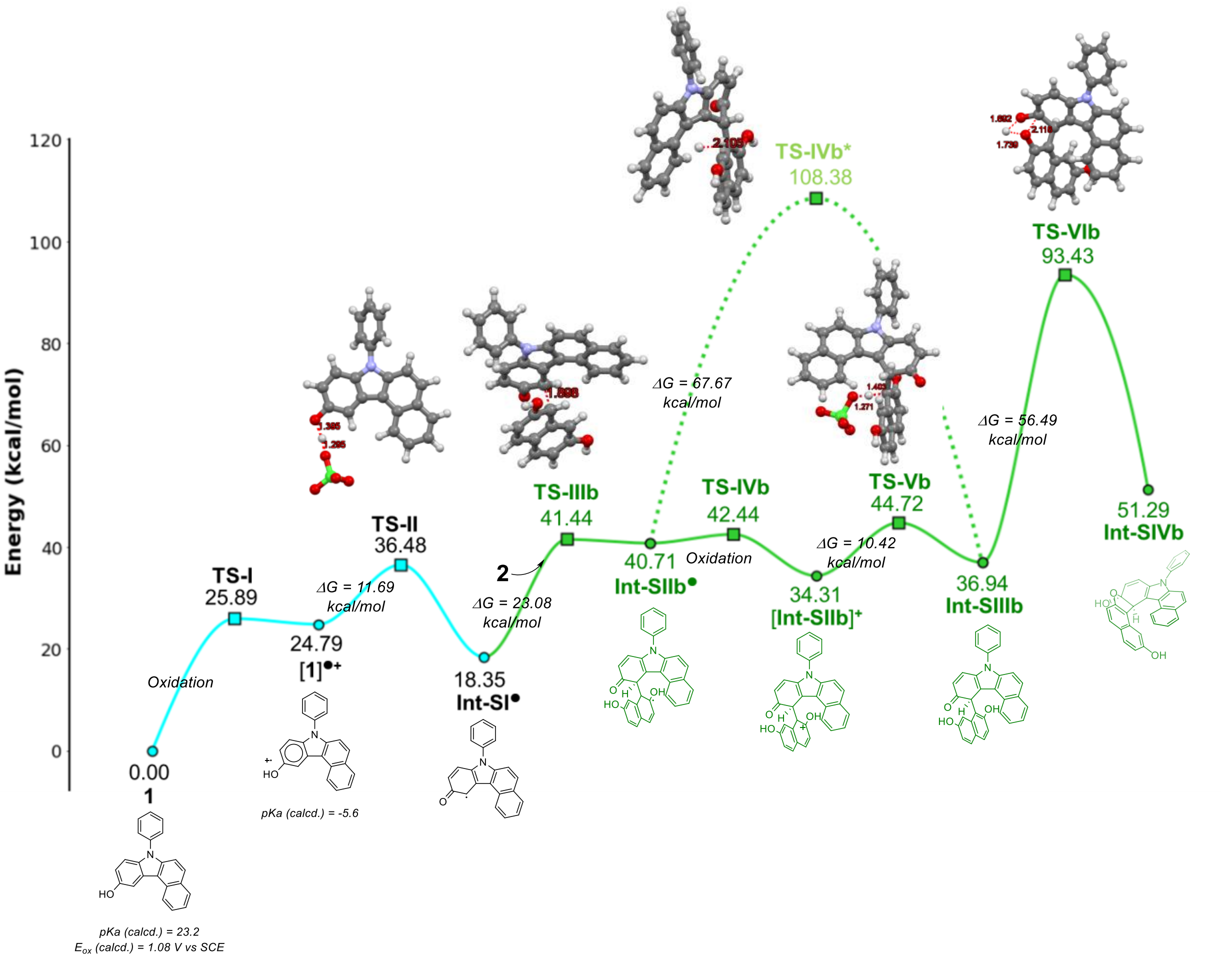

Calculated at the UB3LYP/6-31G+(d,p)/IEFPCM=DCM. Grimme's dispersion with the original D3 damping function was applied as empirical dispersion correction to the optimized structures. All global minima were verified by the absence of imaginary frequencies, while all transition states were confirmed by the presence of a single imaginary frequency. Additionally, an Intrinsic Reaction Coordinate (IRC) analysis was performed to ensure that each transition state correctly connects the corresponding reactant and product minima along the reaction pathway.

Due to the configuration of **Int-SIIIa**, which adopts a more favorable geometry, the formation of the corresponding **Int-SIVa** occurs with a significantly lower energy barrier of approximately 39.23 kcal/mol, compared to 56.49 kcal/mol in **Pathway B**.

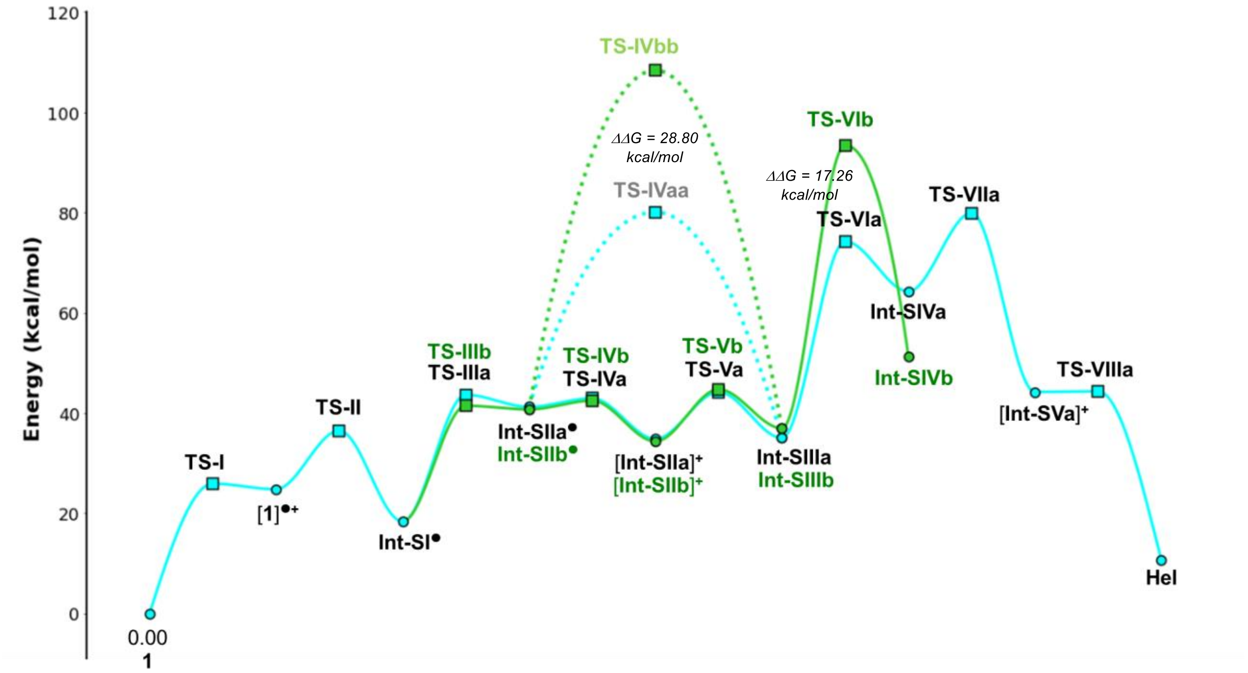

Calculated at the UB3LYP/6-31G+(d,p)/IEFPCM=DCM. Grimme's dispersion with the original D3 damping function was applied as empirical dispersion correction to the optimized structures. All global minima were verified by the absence of imaginary frequencies, while all transition states were confirmed by the presence of a single imaginary frequency. Additionally, an Intrinsic Reaction Coordinate (IRC) analysis was performed to ensure that each transition state correctly connects the corresponding reactant and product minima along the reaction pathway.

- After analyzing the helicenes, their corresponding radical cations, and neutral radicals —along with their pKa values and spin density distributions—we believe they follow a similar reaction pathway. The observed trends in acidity and spin localization suggest that these species undergo initial anodic oxidation to generate the corresponding radical cation, which subsequently loses a proton to form the neutral radical. This neutral radical then undergoes intramolecular coupling, completing the reaction sequence.

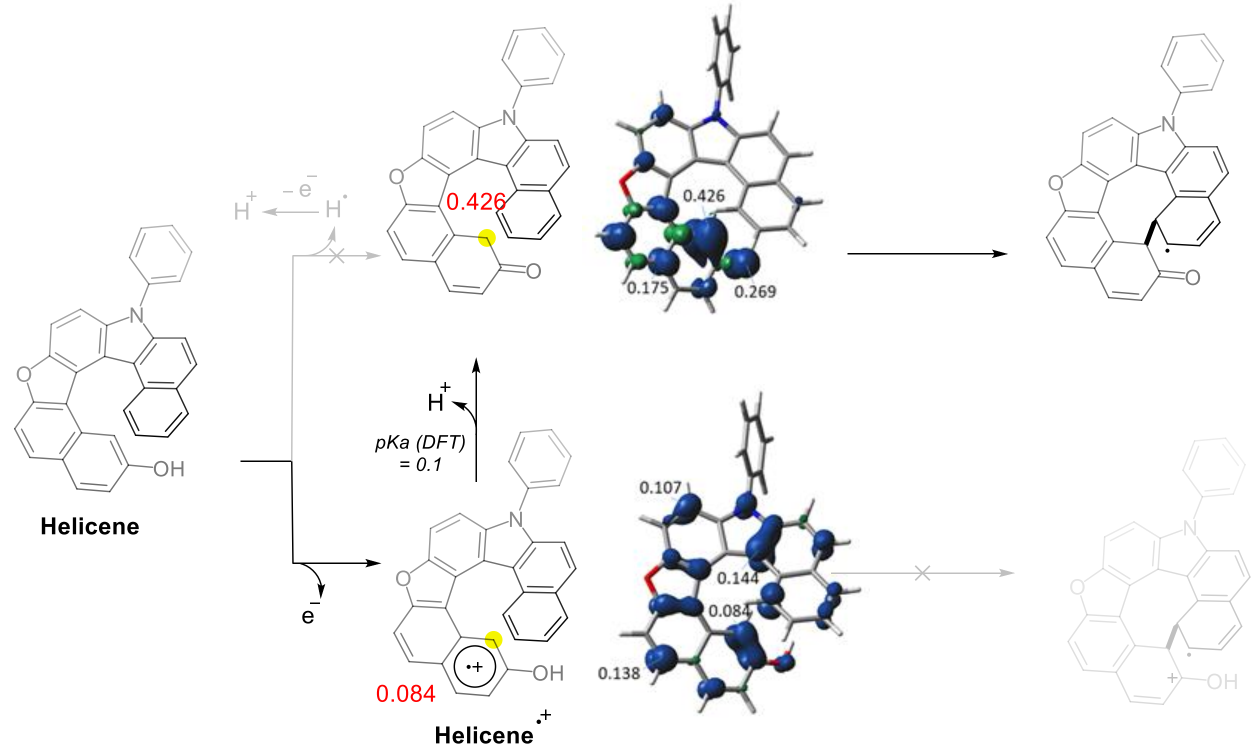

Theoretical prediction of the pKa values of helicenes and their corresponding radical cationic species

Molecule	$E_{(total)}$ Hartree	$E_{(total)}$ kcal/mol	*ΔG* (kcal/mol)	pKa
**Hel-OH**	−1435.8136	−900985.9563	28.30693099	20.8
**Hel-O⁻**	−1435.3395	−900688.4543		
**[Hel]$^{\bullet+}$**	−1435.6199	−900864.4078	0.06902599	0.1
**[Hel]$^{\bullet}$**	−1435.1908	−900595.1437		

Optimized at the UB3LYP/6-31G+(d,p) level of theory with IEPCM model as solvation of DCM. Grimme's dispersion with the original D3 damping function was applied as empirical dispersion correction to the optimized structures.

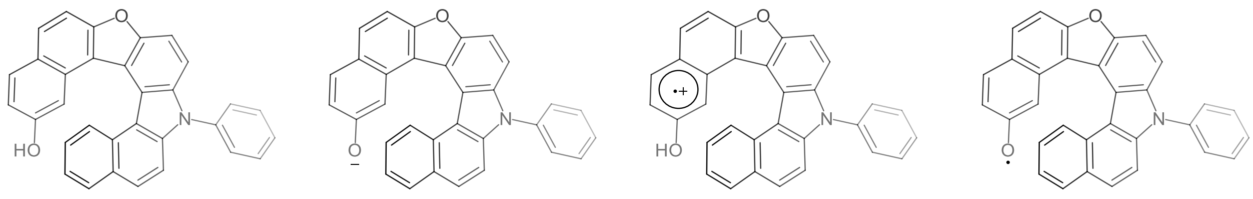

Hel-OH Helicene — **Hel-O⁻** Conjugate base — **[Hel]$^{\bullet+}$** Radical cation — **[Hel]$^{\bullet}$** Neutral radical

Energy profiles, in kcal/mol, for the sequential electrochemical synthesis of circulenes from corresponding helicenes

- Based on mechanistic insights derived from the energy profiles of all intermediates and transition states, as well as pKa values and redox potentials, we propose that the reaction proceeds *via* the following pathway, where oxidation followed by proton loss is more favorable than direct homolytic fission.

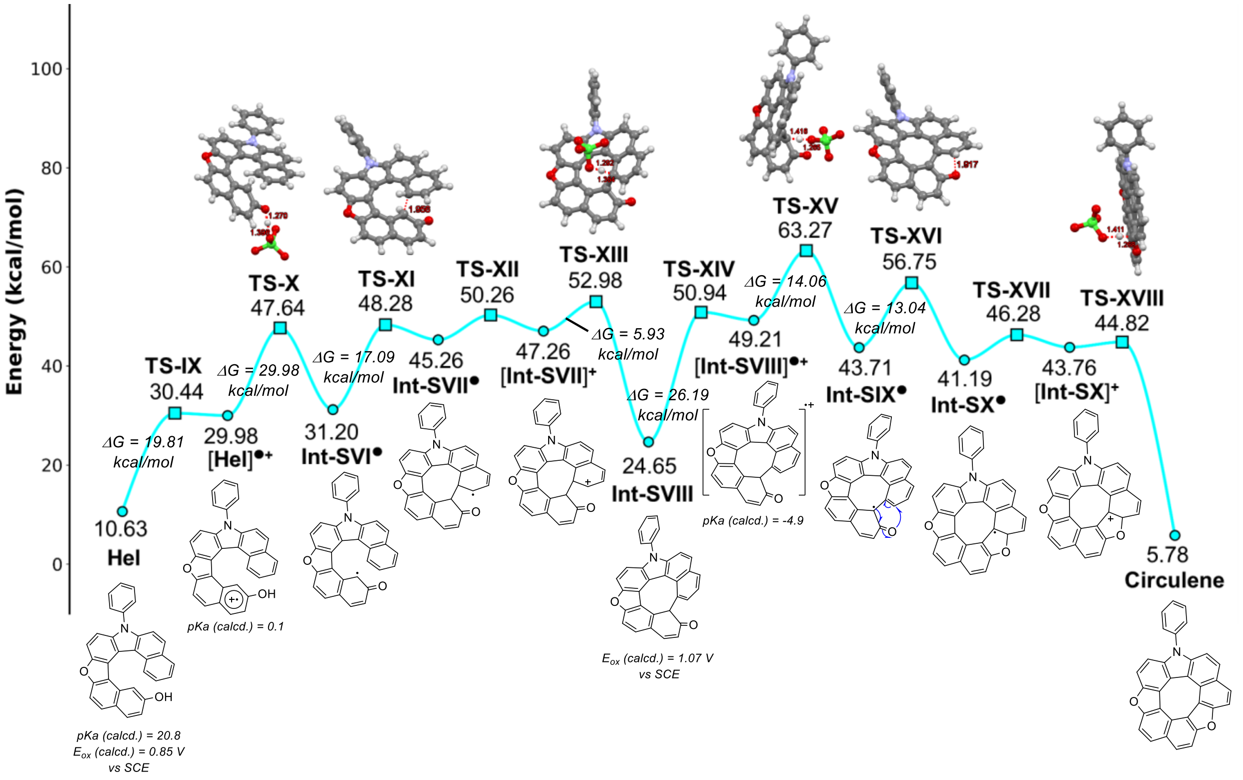

Calculated at the UB3LYP/6-31G+(d,p)/IEFPCM=DCM. Grimme's dispersion with the original D3 damping function was applied as empirical dispersion correction to the optimized structures. All global minima were verified by the absence of imaginary frequencies, while all transition states were confirmed by the presence of a single imaginary frequency. Additionally, an Intrinsic Reaction Coordinate (IRC) analysis was performed to ensure that each transition state correctly connects the corresponding reactant and product minima along the reaction pathway

- The calculations—along with supporting pKa and redox potential DFT-based predictions—suggest that direct H-atom removal (**TS-XII***, **TS-XIV***, and **TS-XVII***) from the intermediates **Int-SVII**, **Int-SVIII**, and **Int-SX** is likely less favorable than an alternative pathway involving oxidation followed by deprotonation.

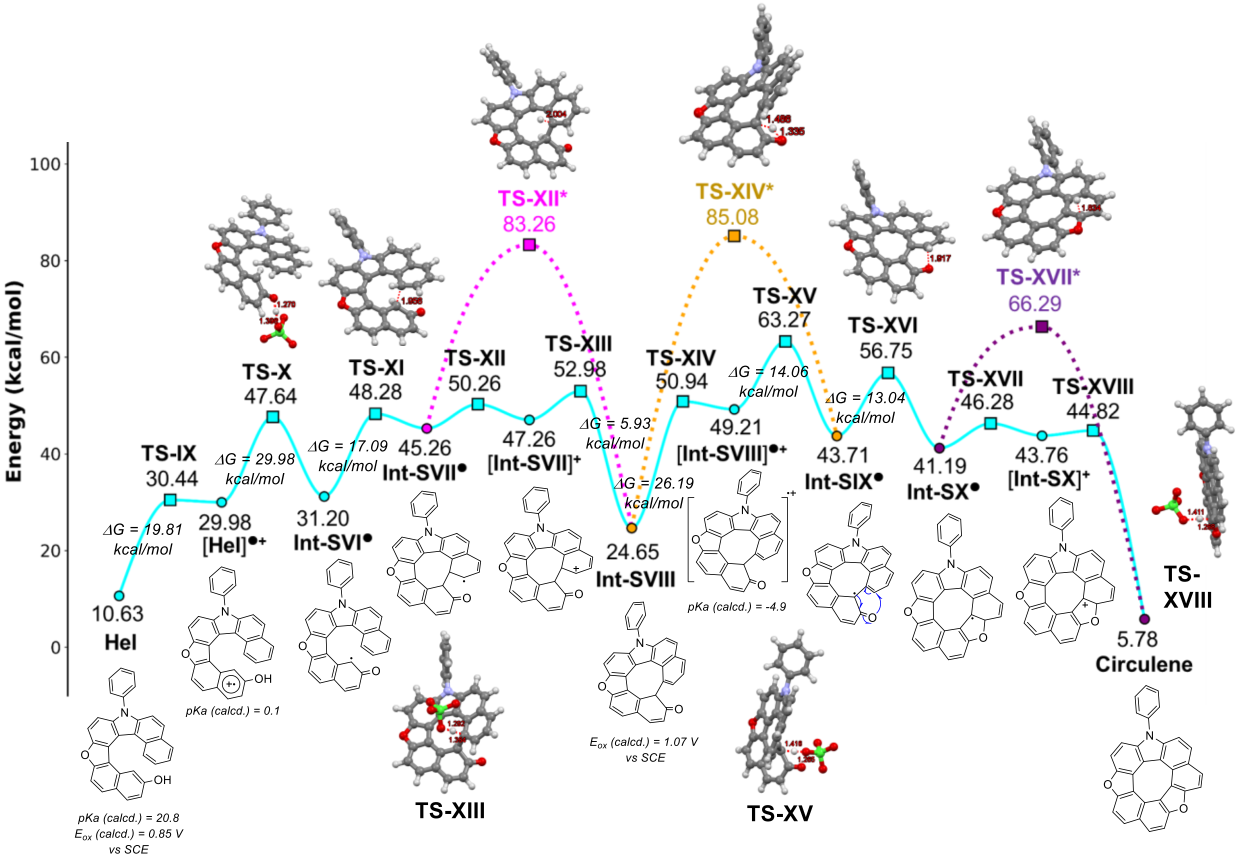

Calculated at the UB3LYP/6-31G+(d,p)/IEFPCM=DCM. Grimme's dispersion with the original D3 damping function was applied as empirical dispersion correction to the optimized structures. All global minima were verified by the absence of imaginary frequencies, while all transition states were confirmed by the presence of a single imaginary frequency. Additionally, an Intrinsic Reaction Coordinate (IRC) analysis was performed to ensure that each transition state correctly connects the corresponding reactant and product minima along the reaction pathway.

- Additionally, we explored an alternative pathway in which **Int-SVII** does not undergo direct anodic oxidation to generate **Int-SVIII** but instead undergoes tautomerization followed by two successive anodic oxidations. However, our calculations indicate that the first pathway is energetically more favorable.

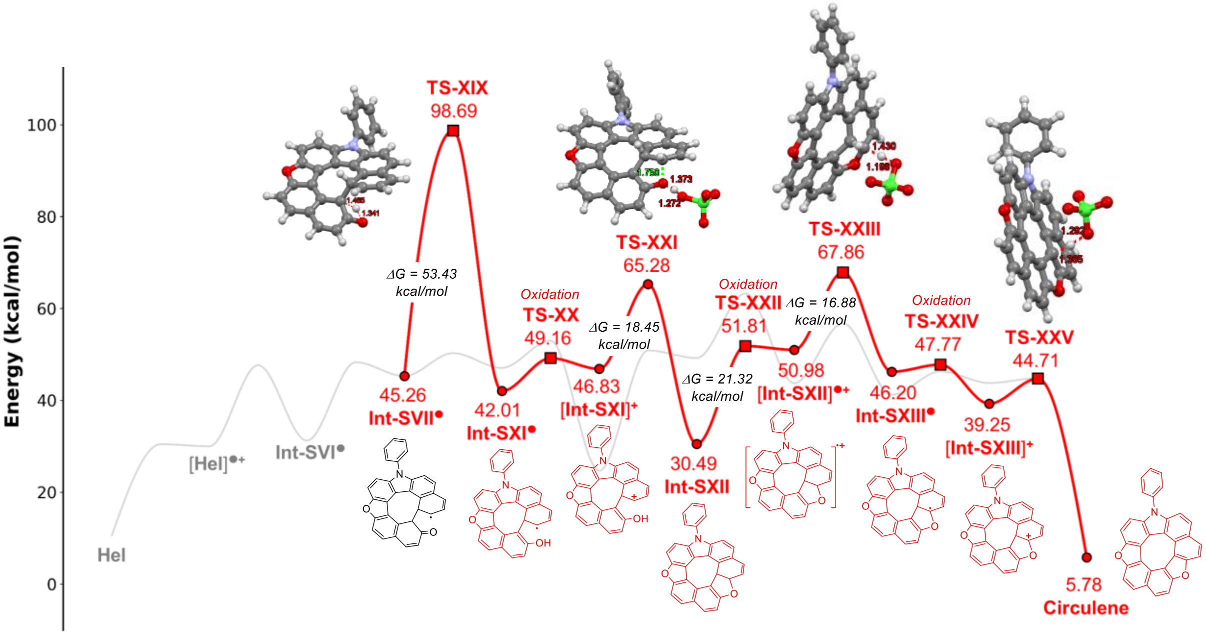

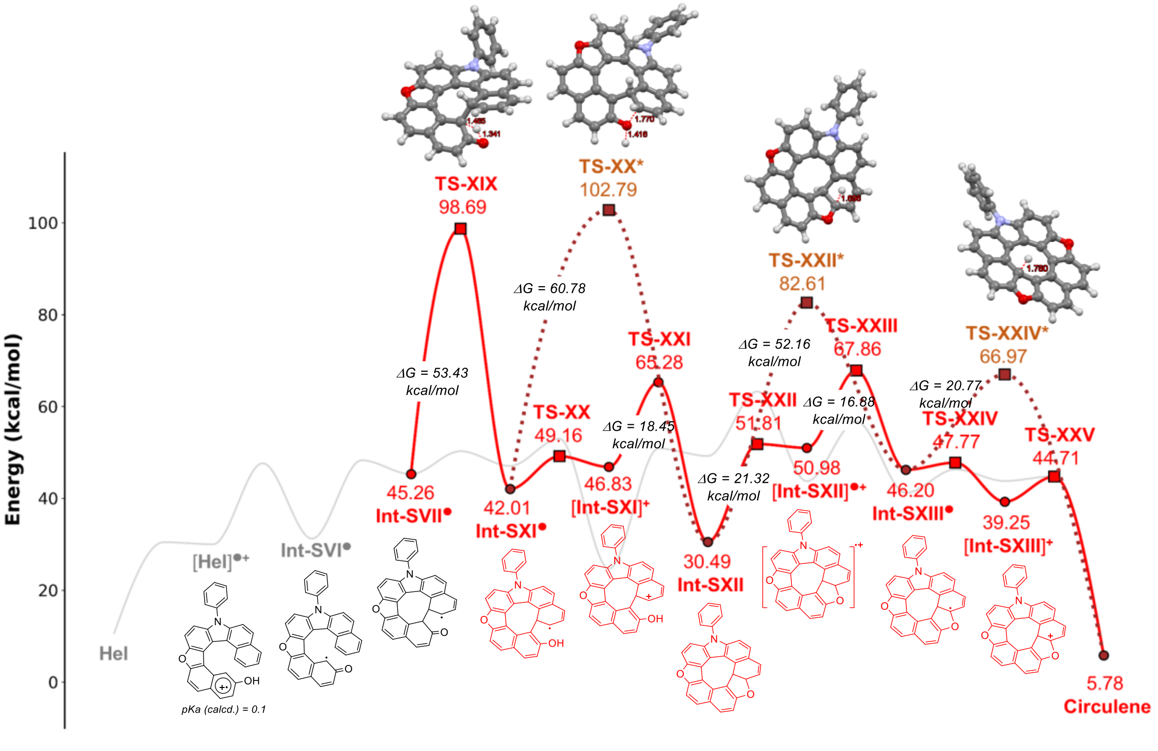

Calculated at the UB3LYP/6-31G+(d,p)/IEFPCM=DCM. Grimme's dispersion with the original D3 damping function was applied as empirical dispersion correction to the optimized structures. All global minima were verified by the absence of imaginary frequencies, while all transition states were confirmed by the presence of a single imaginary frequency. Additionally, an Intrinsic Reaction Coordinate (IRC) analysis was performed to ensure that each transition state correctly connects the corresponding reactant and product minima along the reaction pathway.

- Beyond these two pathways, we also investigated several alternatives connecting them. However, based on the energy profiles of all reaction intermediates and transition states, along with their pKa values, spin densities, and redox behaviors, we conclude that the mechanism presented earlier (shown in black) is the most plausible.

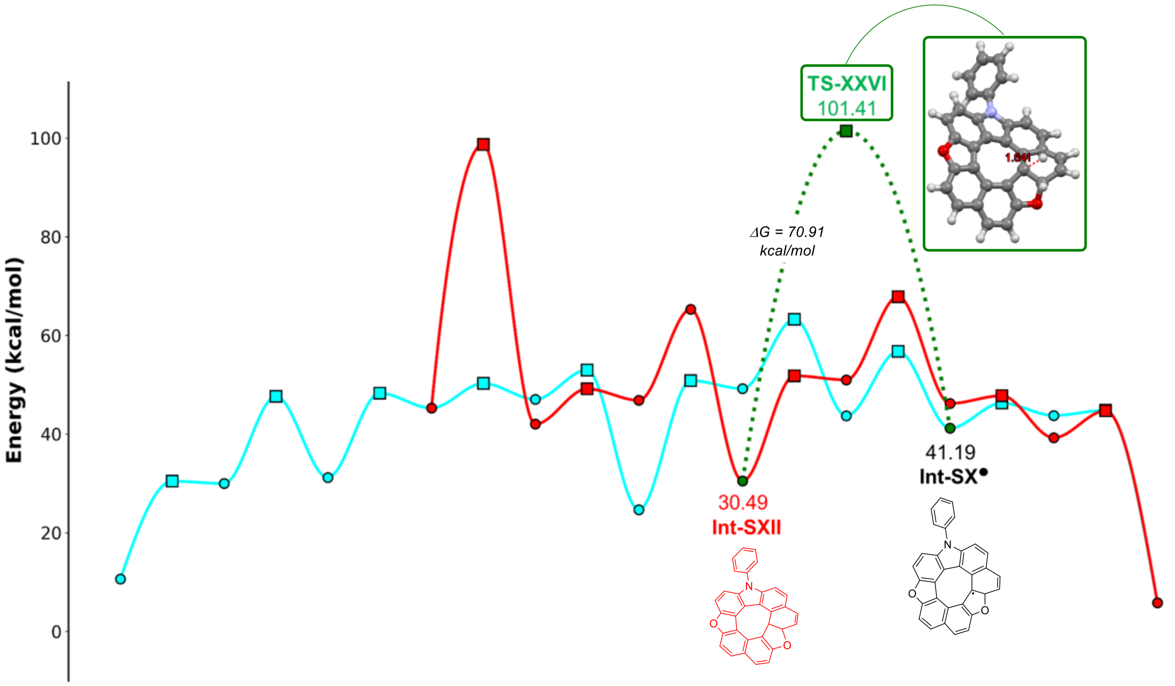

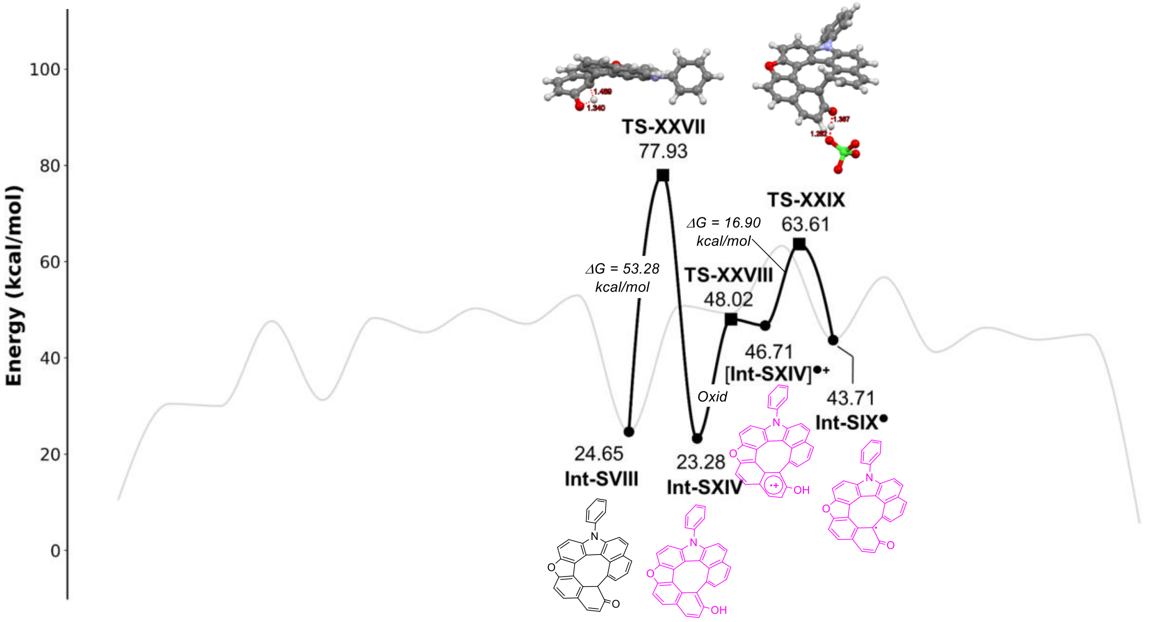

Calculated at the UB3LYP/6-31G+(d,p)/IEFPCM=DCM. Grimme's dispersion with the original D3 damping function was applied as empirical dispersion correction to the optimized structures. All global minima were verified by the absence of imaginary frequencies, while all transition states were confirmed by the presence of a single imaginary frequency. Additionally, an Intrinsic Reaction Coordinate (IRC) analysis was performed to ensure that each transition state correctly connects the corresponding reactant and product minima along the reaction pathway

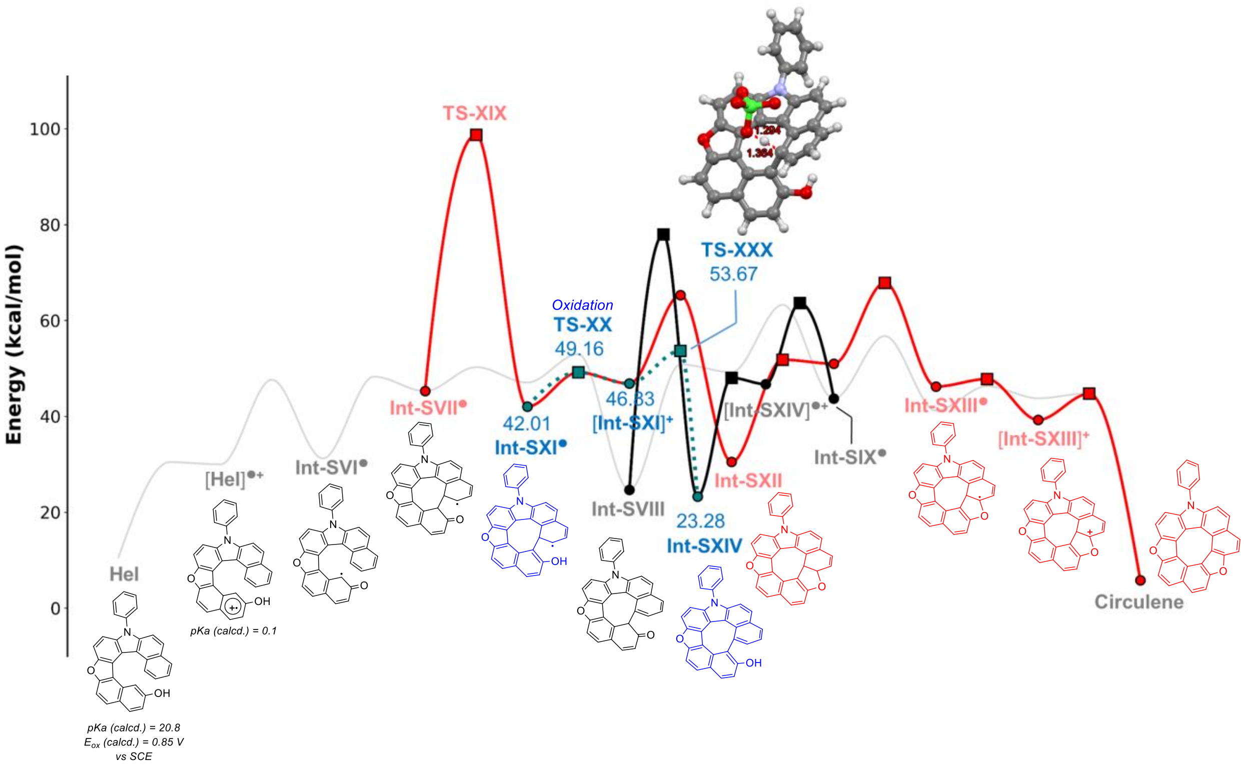

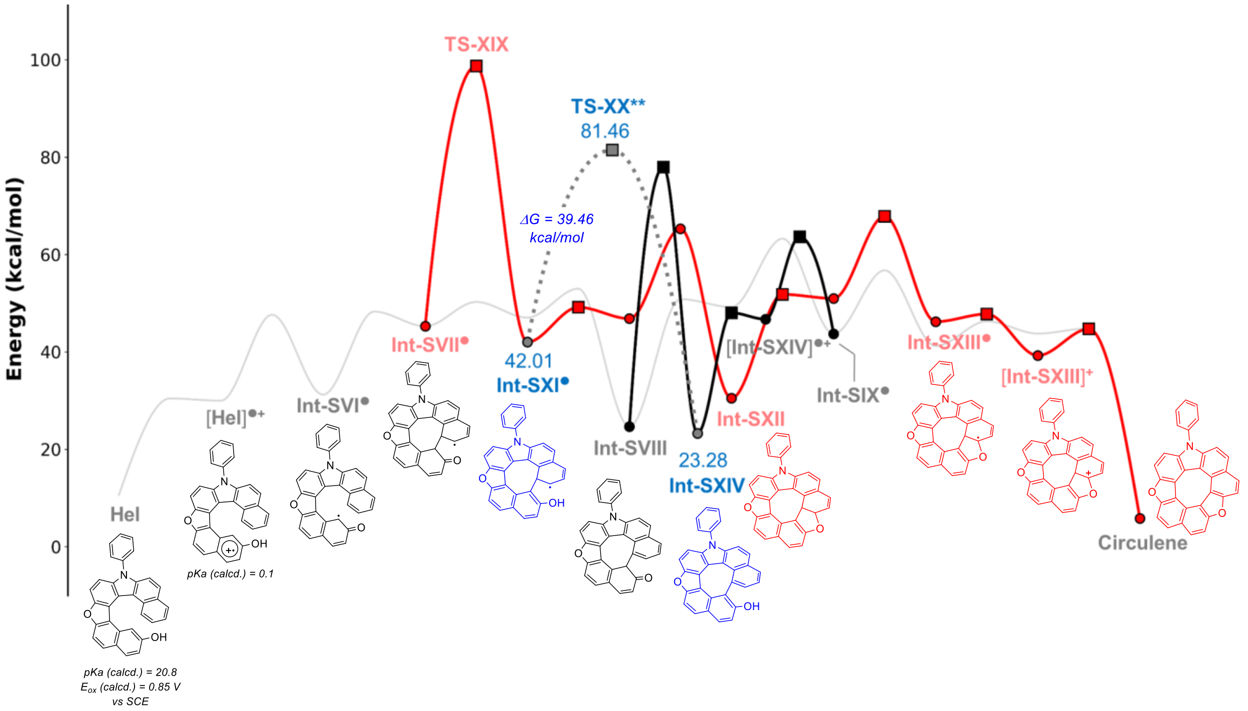

Calculated at the UB3LYP/6-31G+(d,p)/IEFPCM=DCM. Grimme's dispersion with the original D3 damping function was applied as empirical dispersion correction to the optimized structures. All global minima were verified by the absence of imaginary frequencies, while all transition states were confirmed by the presence of a single imaginary frequency. Additionally, an Intrinsic Reaction Coordinate (IRC) analysis was performed to ensure that each transition state correctly connects the corresponding reactant and product minima along the reaction pathway.

Spin Density Analysis of Neutral and cationic Radical Intermediates involved in the mechanism of electrochemical synthesis of circulene

To gain deeper insight into the reaction mechanism, spin density distribution calculations were performed to provide theoretical representations of radical intermediates[44]. Since radical cations and neutral radicals involved in the mechanism are too reactive to be isolated experimentally, direct characterization is impractical. In this context, DFT calculations serve as a powerful tool for visualizing reaction pathways. Particularly for reactions involving radical cations and neutral radicals, spin density analysis helps identify the molecular regions participating in single-electron transfer (SET) processes. Our results indicate that spin densities are predominantly localized on the reactive sites of neutral radicals rather than on the corresponding radical cations, highlighting their crucial role in the reaction mechanism.

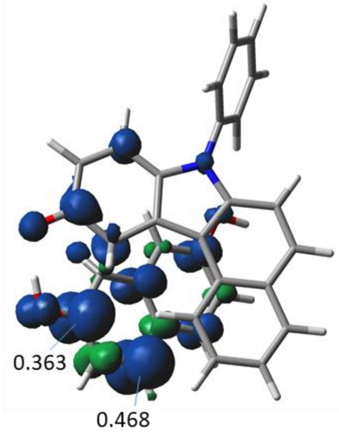

Int-SIIa

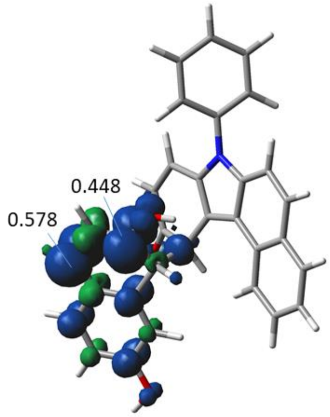

Int-SIIb

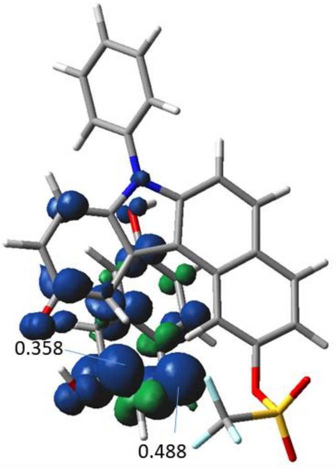

Int-SIIc

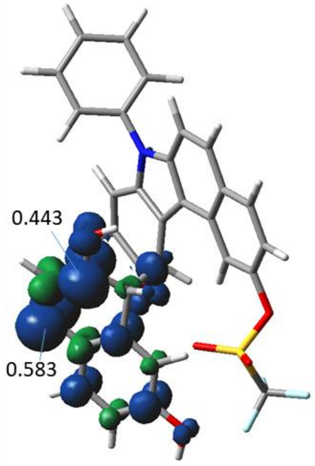

Int-SIId

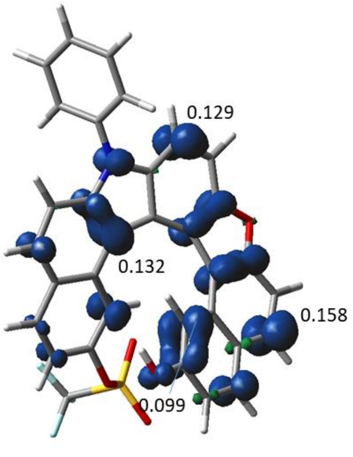

[Hel-OTf]$^{\bullet+}$

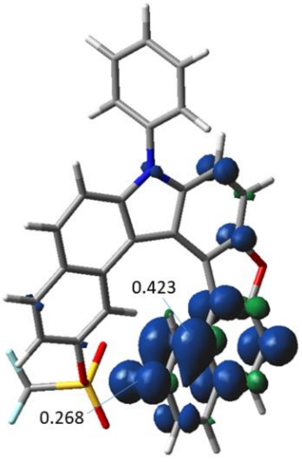

Int-SVIb

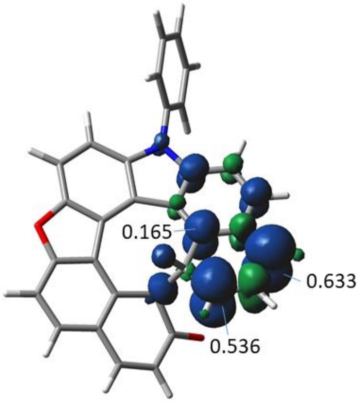

Int-SVIIa

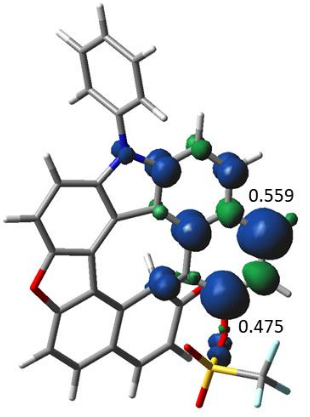

Int-SVIIb

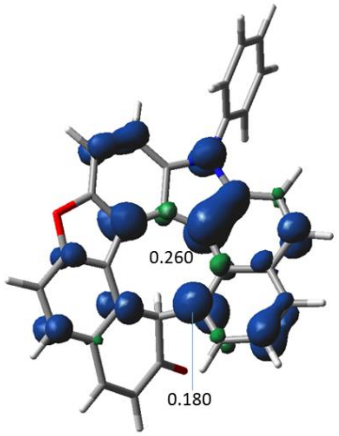

[Int-SVIII]$^{\bullet+}$

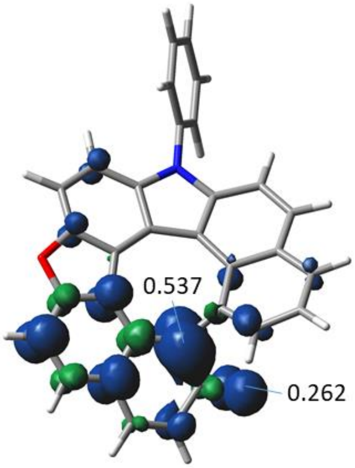

Int-SIX

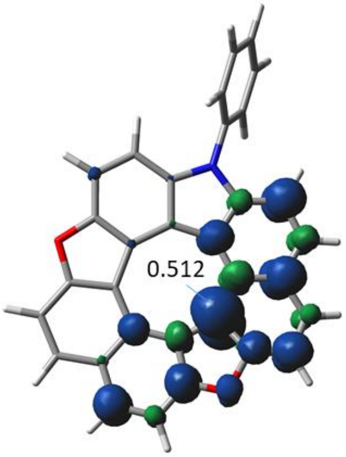

Int-SX

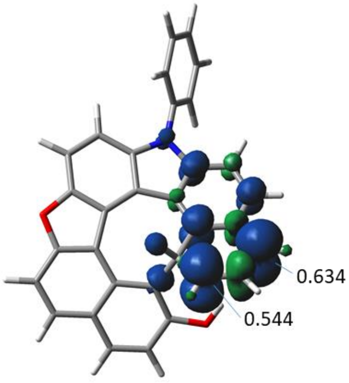

Int-SXI

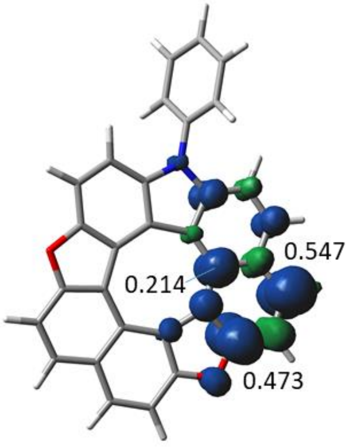

Int-SXIII

Mulliken spin density contour maps calculated at the UB3LYP/6-31G+(d,p)/IEFPCM=DCM (isoval = 0.003).

Theoretical estimation of the pKa and E_{ox} values of various compounds and intermediates

Theoretical prediction of the pKa values of various compounds and intermediates included the mechansitic investigation also suggest that certain radical intermediates, such as **Int-SII**, **Int-SVII**, and **Int-SXI**, may undergo another pathways including anodic oxidation to generate the corresponding cationic species. Due to the high acidity of these cationic species, they are more likely to release a proton subsequently. This behavior is similar to that of neutral species, which generate neutral radicals such as **1**, **Hel**, and **Int-SVIII**. However, these variations do not alter the overall reaction mechanism, as the generated species rapidly lose an electron, forming the corresponding neutral species.

Molecule	$E_{(total)}$ Hartree	$E_{(total)}$ kcal/mol	*ΔG* (kcal/mol)	pKa
[**Int-SIIa**]$^+$	−1512.6105	−949176.7022	−19.25825121	−14.1
**Int-SIIIa**	−1512.2122	−948926.7654		
**[Hel-OTf]$^{\bullet+}$**	−2396.4377	−1503786.225	−3.19402081	−2.3
**[Hel-OTf]$^{\bullet}$**	−2396.0138	−1503520.224		
[**Int-SVIIa**]$^+$	−1435.0028	−900477.172	−23.52531241	−17.3
**Int-SVIIIa**	−1434.6113	−900231.5023		
[**Int-SVIIb**]$^+$	−2395.8169	−1503396.667	−14.61468461	−10.7
**Int-SVIIIb**	−2395.4112	−1503142.087		
**[Int-SVIII]$^{\bullet+}$**	−1434.4093	−900104.7454	−6.64532031	−4.9
**Int-SIX**	−1433.9909	−899842.1957		
**Int- SVIII-OTf**	−2395.2179	−1503020.789	−13.35966661	−9.8
**Int-SIX-OTf**	−2394.8102	−1502764.954		
[**Int-SX**]$^+$	−1433.828	−899739.9745	−39.08753561	−28.7
**3b**	−1433.4613	−899509.8669		

Optimized at the UB3LYP/6-31G+(d,p) level of theory with IEPCM model as solvation of DCM. Grimme's dispersion with the original D3 damping function was applied as empirical dispersion correction to the optimized structures.

- We followed Nicewicz and coworkers' protocol to calculate redox potentials for various compound and intermediates included in our mechanistic study[45].

$$E_{\frac{1}{2}}^{o,calc} = -\frac{(G_{298}[\text{red}] - G_{298}[\text{Ox}])}{n_e\mathcal{F}} - E_{\frac{1}{2}}^{o,SHE} + E_{\frac{1}{2}}^{o,SCE}$$

Solution-phase energies were referenced to SCE by subtracting 4.281 V (the absolute potential of SHE) and 0.141 V (the conversion of SHE to SCE). Where n_e is the number of electrons transferred (here, n_e= 1), $\mathcal{F}$ is the Faraday constant (23.06 kcal mol^{-1} V^{-1}), $E_{1/2}^{o,SH}$ is the absolute value for the standard hydrogen electrode (SHE, 4.281 V), $E_{1/2}^{o,SCE}$ is the potential of the saturated calomel electrode (SCE) relative to SHE

(−0.141 V), and G oxidized and G reduced are the Gibbs free energies as obtained from DFT calculations.

Molecule	G298 [ground state]	G298 [oxidized species]	Oxidation Pot ($E^{calc}_{1/2}$)
**1b'**	−977.18835	−976.98605	1.08
**1b**	−1938.0134	−1937.8109	1.09
**Hel**	−1435.8136	−1435.6199	0.85
**Hel-OTf**	−2396.638	−2396.4377	1.03
**Int-SIIa**	−1512.7631	−1512.6105	−0.27
**Int-SIIc**	−2473.5912	−2473.4301	−0.04
**Int-SIId**	−2473.5846	−2473.4246	−0.07
**Int-SVIIa**	−1435.1684	−1435.0028	0.08
**Int-SVIIb**	−2395.9911	−2395.8169	0.32
**Int-SVIII**	−1434.6113	−1434.4093	1.07
**Int-SVIIIb**	−2395.4112	−2395.2179	0.84
**Int-SX**	−1433.9949	−1433.828	0.12

Optimized at the UB3LYP/6-31G+(d,p) level of theory with IEPCM model as solvation of DCM. Grimme's dispersion with the original D3 damping function was applied as empirical dispersion correction to the optimized structures.

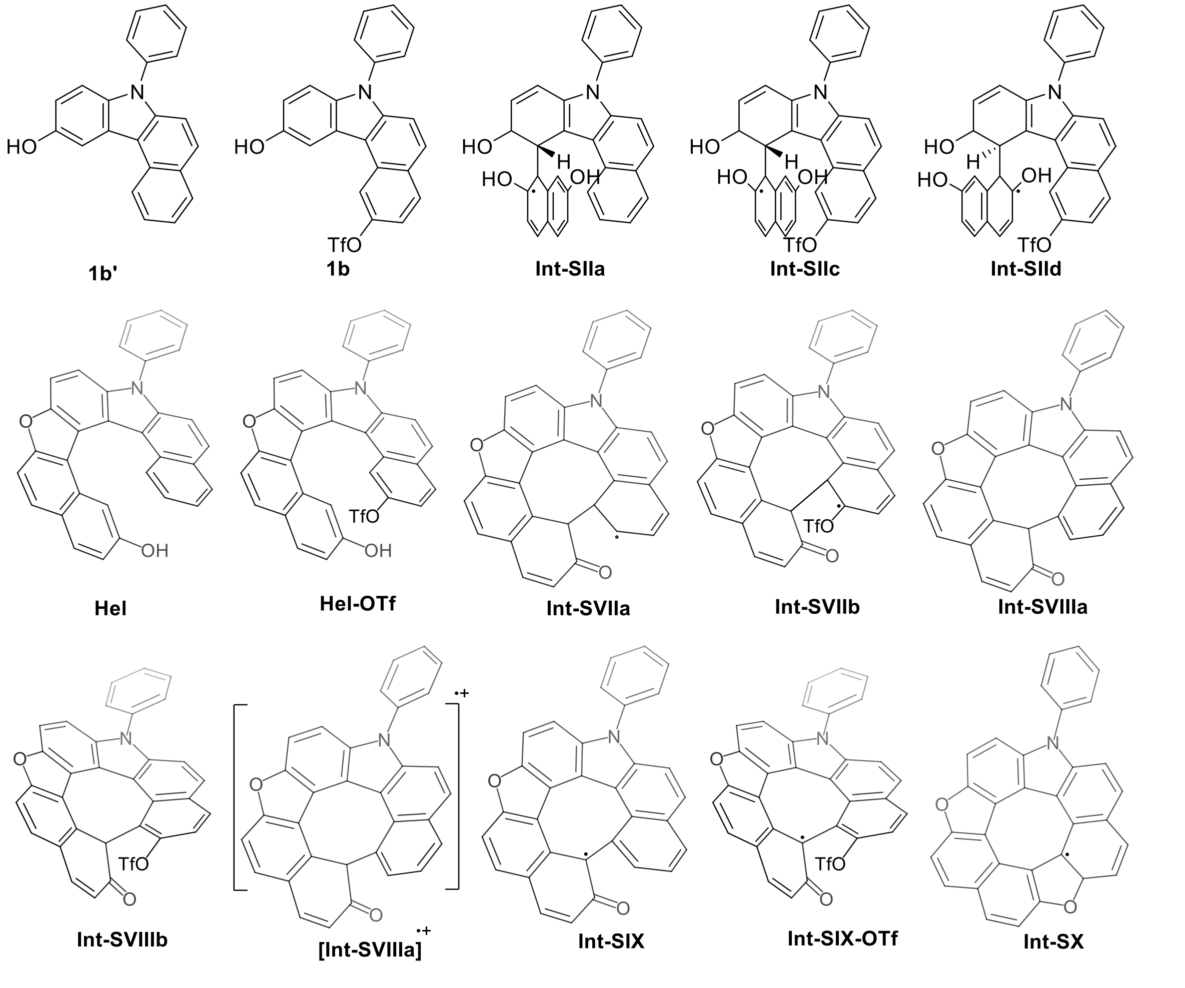

Energy Barrier Calculations via Marcus Theory

Marcus theory, developed by Rudolph A. Marcus, provides a framework to predict the rate of electron transfer (ET) reactions by linking the activation energy barrier (ΔG†) to two key parameters: the reorganization energy (λ) and the standard Gibbs free energy change (ΔG∘) of the reaction[46]. The theory posits that during ET, energy is required to reorganize both the molecular structure (bond lengths/angles) and the surrounding solvent shell to accommodate the charge redistribution[47].

The activation energy barrier is expressed as:

$$\Delta G^{\dagger} = \frac{(\Delta G^{\circ} + \lambda)^2}{4\lambda}$$

where:

- **λ** (reorganization energy): Energy required to reorganize the system (solvent and solute) without electron transfer.
- **ΔG∘** (thermodynamic driving force): Energy difference between the oxidized and reduced states at equilibrium.

Application to Energy Barrier Calculations

- Reorganization Energy (λ):

The reorganization energy (λ) and Gibbs free energy change (ΔG∘) were calculated using the **Buda method**[48], which partitions solvent polarization into **fast** (electronic, ε_{opt}) and **slow** (inertial, ε_{static}) components during vertical electron transfer (ET) transitions. The protocol involves four steps:

Computational Setup

Once the optimized geometries of the substrates and radical cations were obtained, four single-point calculations were performed in order to obtain the equilibrium and non-equilibrium solvation free-energies (corresponding to a vertical transition) in solution:

- **Step 1:** Read the (optimized) geometry of substrate and calculate the equilibrium free energy for substrate in DCM at the ub3lyp/6-31+g(d,p) IOP(3/124=40) level of theory and write the slow solvation charges needed in step 2 for calculating the non-equilibrium free energy for oxidized species (noneq = write), G^0(Sub).
- **Step 2**: Read the (optimized) geometry of substrate and the slow solvation charges computed in step 1 and calculate the non-equilibrium free energy in DCM for oxidized species using the solvent's optical dielectric constant and in the presence of the fixed slow charges form the previous step (noneq = read), $G^*(Sub^{+\bullet})$ (first vertical transition – the free energy for oxidized species is calculated at the geometry of substrate).
- **Step 3**: Read the (optimized) geometry of oxidized species and calculate the equilibrium free energy for oxidized species in DCM and write the slow solvation charges needed in step 4 for calculating the non-equilibrium free energy for substrate (noneq = write), $G^0(Sub^{+\bullet})$.
- **Step 4**: Read the (optimized) geometry of oxidized species and the slow solvation charges computed in step 3 and calculate the non-equilibrium free energy in DCM for substrate using the solvent's optical dielectric constant and in the presence of the fixed slow charges form the previous step (noneq = read), G^*(Sub)

(second vertical transition – the energy for **substrate** is calculated at the geometry of **oxidized species**).

•	1b'	Int-SIIa	Hel	Int-SVII	Int-SVIII	Int-SX
**G^0(Sub)** (Hartree)	-977.548	-1513.332	-1436.329	-1435.676	-1435.107	-1434.474
**G^*($Sub^{+\bullet}$)** (Hartree)	-977.320	-1513.153	-1436.113	-1435.487	-1434.881	-1434.286
**G^0($Sub^{+\bullet}$)** (Hartree)	-977.345	-1513.181	-1436.135	-1435.513	-1434.904	-1434.310
**G^*(Sub)** (Hartree)	-977.521	-1513.305	-1436.305	-1435.650	-1435.084	-1434.450
$\lambda_{\rightarrow}$ (Hartree)	0.025	0.028	0.023	0.026	0.024	0.023
$\lambda_{\leftarrow}$ (Hartree)	0.027	0.028	0.023	0.026	0.024	0.024
$\lambda_{\leftrightarrow}$ (Hartree)	0.026	0.028	0.023	0.026	0.024	0.024
$\lambda_{\leftrightarrow}$ (Kcal/mol)	16.303	17.319	14.464	16.284	14.872	14.746

$$Sub \rightarrow Sub^{+\bullet} + e^- \qquad \lambda_{\rightarrow} = G^*(Sub)^{+\bullet} - G^0(Sub)^{+\bullet}$$

$$Sub^{+\bullet} + e^- \rightarrow Sub \qquad \lambda_{\leftarrow} = G^*(Sub) - G^0(Sub)$$

$$ROE\ \lambda_{\leftrightarrow} = \frac{(\lambda_{\rightarrow} + \lambda_{\leftarrow})}{2}$$

- Driving Force (ΔG∘):

Computed Gibbs free energies were corrected for solvation and thermal contributions (vibrational, rotational, translational) and benchmarked against the absolute potential of the standard hydrogen electrode (SHE, 4.43 eV)[49]. This alignment bridges the inherent energy offset between vacuum-referenced DFT calculations and experimental SHE-referenced redox potentials, enabling direct comparison of neutral and charged species' energetics. The correction ensures $\Delta G°$ reflects realistic, solvated driving forces for electron transfer.

$$\Delta G^0 = \Delta G_{oxid} - E_{SHE,abs}$$

$$\Delta G^{\dagger} = \frac{(\Delta G° + \lambda)^2}{4\lambda}$$

	1b'	Int-SIIa	Hel	Int-SVII	Int-SVIII	Int-SX
***Corrected* G^0(Sub)** (Hartree)	-977.188	-1512.763	-1435.814	-1435.168	-1434.611	-1433.995
***Corrected* G^0($Sub^{+\bullet}$)** (Hartree)	-976.986	-1512.611	-1435.620	-1435.003	-1434.409	-1433.828
ΔG_{oxid}	0.202	0.153	0.194	0.166	0.202	0.167
ΔG^0 (Hartree)	0.040	-0.010	0.031	0.003	0.039	0.004
$\Delta G^{\dagger}$ (Hartree)	0.041	0.003	0.032	0.008	0.042	0.008
$\Delta G^{\dagger}$ (Kcal/mol)	25.891	1.721	19.810	4.997	26.189	5.085

- Other intermediates not included in the main pathway

	Int-SIIb	Int-SXI	Int-SXII	Int-SXIII	Int-SXIV
***G*⁰(Sub)** (Hartree)	-1513.333	-1435.681	-1435.098	-1434.466	-1435.108
***G**(Sub$^{+\bullet}$)** (Hartree)	-1513.154	-1435.486	-1434.880	-1434.290	-1434.886
***G*⁰(Sub$^{+\bullet}$)** (Hartree)	-1513.181	-1435.514	-1434.901	-1434.318	-1434.909
***G**(Sub)** (Hartree)	-1513.306	-1435.652	-1435.076	-1434.438	-1435.085
$\lambda_{\rightarrow}$ (Hartree)	0.028	0.027	0.022	0.028	0.023
$\lambda_{\leftarrow}$ (Hartree)	0.028	0.029	0.022	0.028	0.023
$\lambda_{\leftrightarrow}$ (Hartree)	0.028	0.028	0.022	0.028	0.023
$\lambda_{\leftrightarrow}$ (Kcal/mol)	17.386	17.633	13.617	17.413	14.590

	Int-SIIb	Int-SXI	Int-SXII	Int-SXIII	Int-SXIV
***Corrected G⁰*(Sub)** (Hartree)	-1512.764	-1435.174	-1434.602	-1433.987	-1434.613
***Corrected G⁰*(Sub$^{+\bullet}$)** (Hartree)	-1512.611	-1435.003	-1434.407	-1433.835	-1434.413
ΔG_{oxid}	0.153	0.171	0.195	0.152	0.200
ΔG^0 (Hartree)	-0.010	0.008	0.033	-0.011	0.037
$\Delta G^{\dagger}$ (Hartree)	0.003	0.011	0.034	0.002	0.039
$\Delta G^{\dagger}$ (Kcal/mol)	1.735	7.155	21.316	1.567	24.738

9. Supplementary Note 2: *calculation of Faradic efficiency*

Faradic efficiency was determined according to the equation reported[50,51]:

$$\text{Faradic effeciency (FE)} = \frac{nZF}{Q} = \frac{nZF}{It}$$

Where, ***n*** is the amount of product detected (number of moles, mol)

Q is the total charge passed through the system, recorded during electrolysis (coulombs, C)

F is the Faraday constant (96485.332 C mol^{-1})

Z is the number of electrons required to obtain 1 molecule of the product

I is the recorded current (A)

t is the time required.

For circulene (Z = 4 electrons)

So, the faradic efficiency for compound **3a** obtained from method A, with yield 83% will be as follows:

$$\text{Faradic effeciency (FE)} = \frac{nZF}{Q} = \frac{nZF}{It} = \frac{4 \text{ X } 0.0000022 \text{ X } 0.83 \text{ X } 96485.322}{0.001 \text{ X } 150 \text{ X} 60} = 78\%$$

10. **Supplementary Note 3:** *monitoring the working electrode potential*

To monitor the potential of the working electrode, a three-electrode system was employed using the ElectraSyn 2.0 setup. This system included a reference electrode to provide a stable potential reference and a counter electrode to complete the circuit and facilitate current flow in an undivided cell[52]. Two platinum-plated copper electrodes were used as working electrodes, along with a silver reference electrode. The experiment was conducted at a current density of 0.51 mA/cm² (corresponding to a current of 1.1 mA) in a dichloromethane (DCM) solvent.

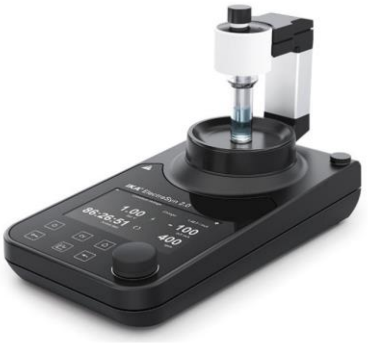

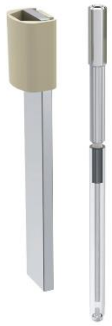

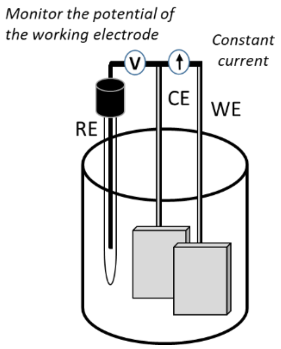

- The potential at the working electrodes was monitored over a period of 1 hour.

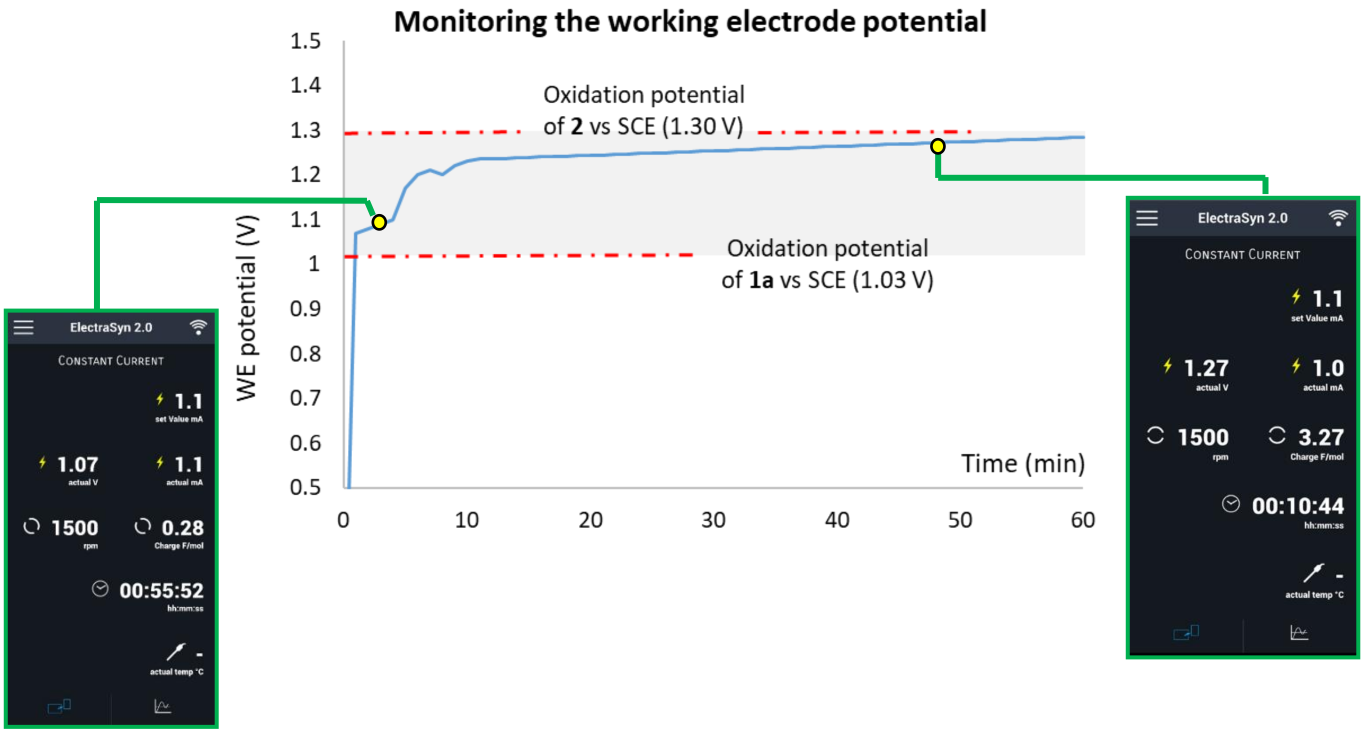

11. **Supplementary Note 4:** *plausible reaction mechanism of organo-photocatalytic arylation reaction*

- Plausible reaction mechanism.

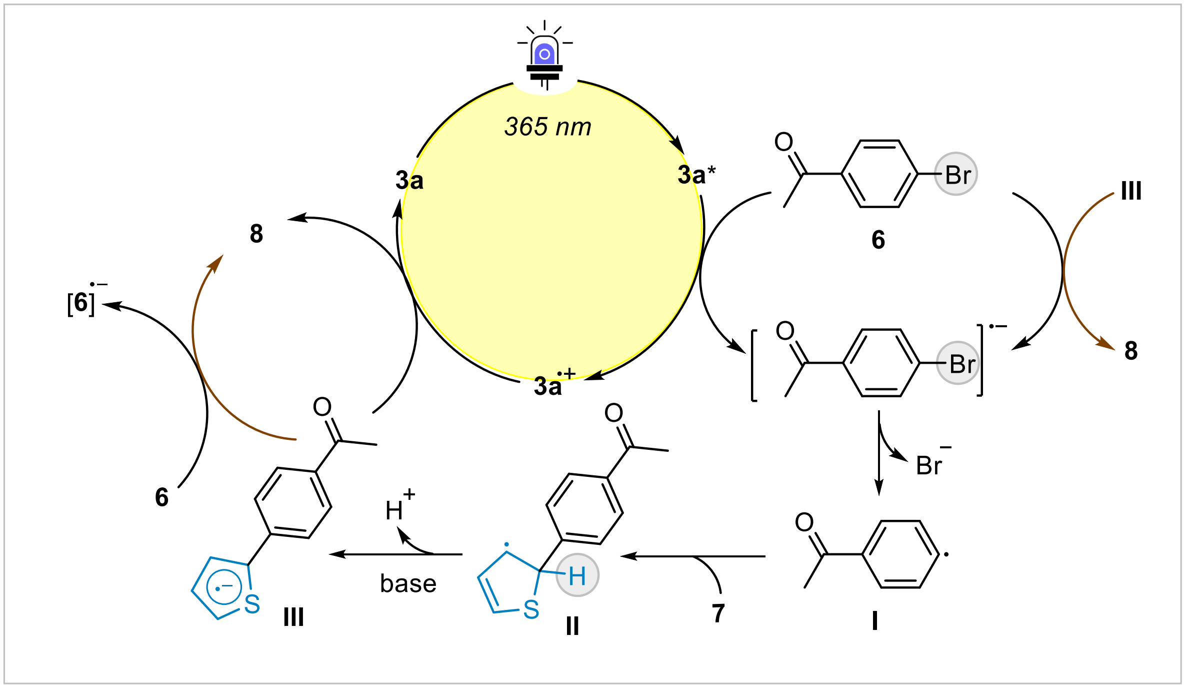

- *Control experiments.*

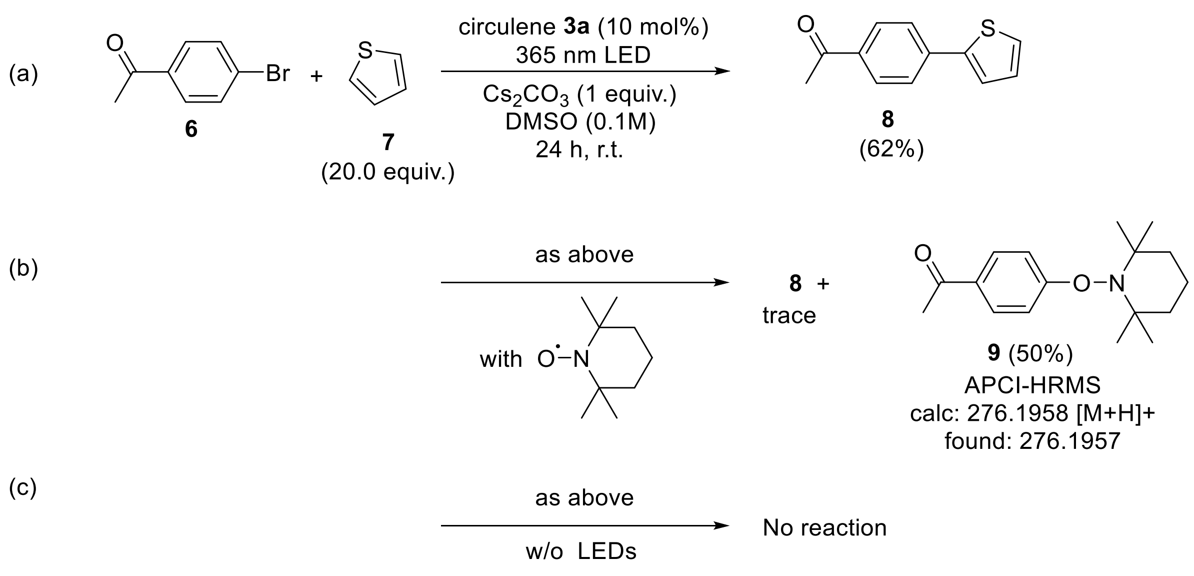

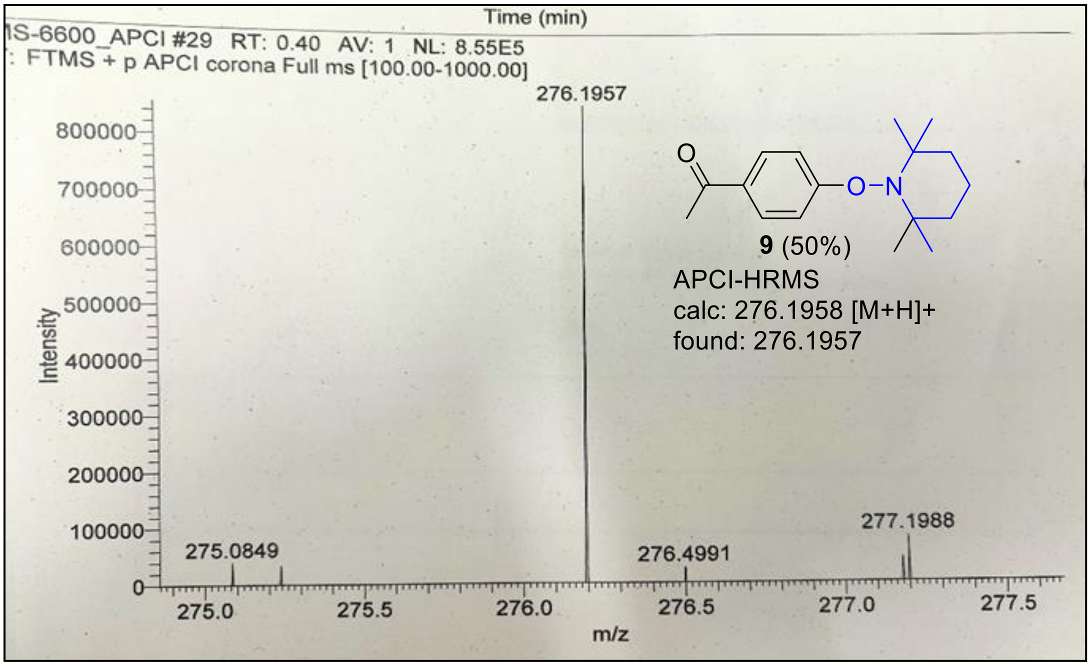

To better understand the photocatalytic properties of circulene **3a** synthesized from hydroxycarbazole **1a** and 2,7-dihydroxynaphthalene **2**, we conducted a comparative study. Specifically, we applied both **1a** and **2** as organophotocatalysts, comparing the results with those of circulene **3a**. Our findings revealed that **1a** is the primary component responsible for the photocatalytic activity of *p*-tolylcirculene **3a**, acting as an electron donor and exhibiting a strong reduction potential. In contrast, using **2** as a photocatalyst did not afford our target compound **8**.

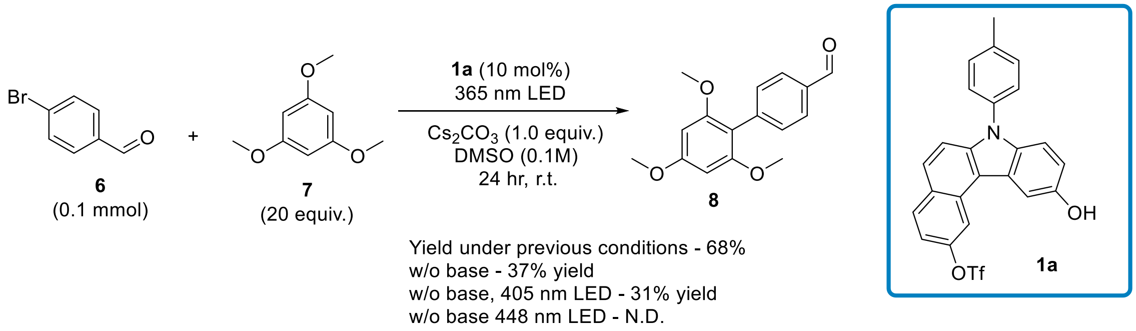

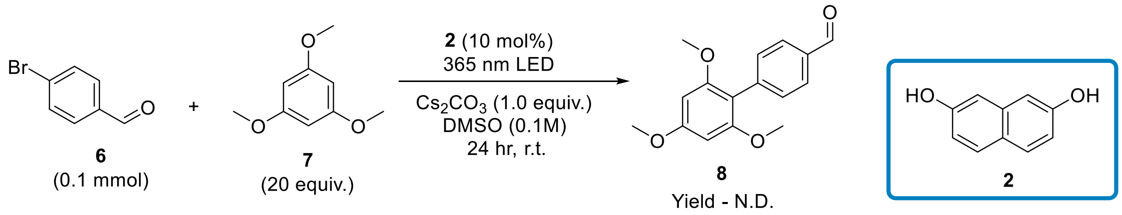

- UV absorbance of hydroxycarbazole **1a** in pure chloroform.

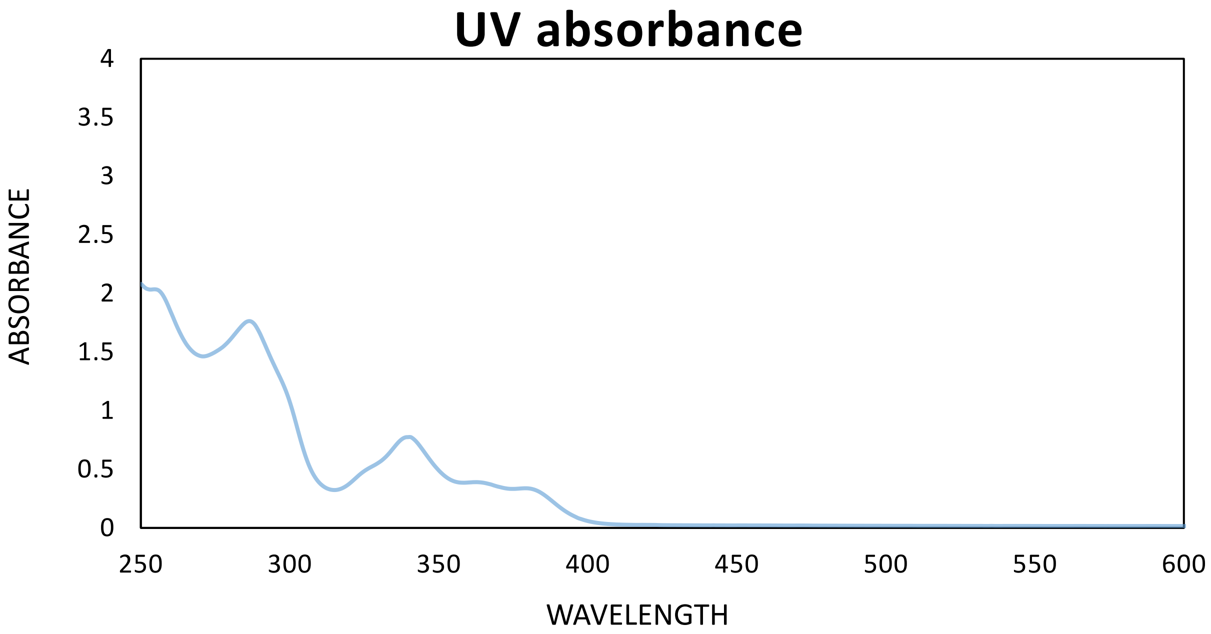

- UV absorbance of reaction mixture (4-bromoacetophenone **6** + thiophene **7**) in pure chloroform.

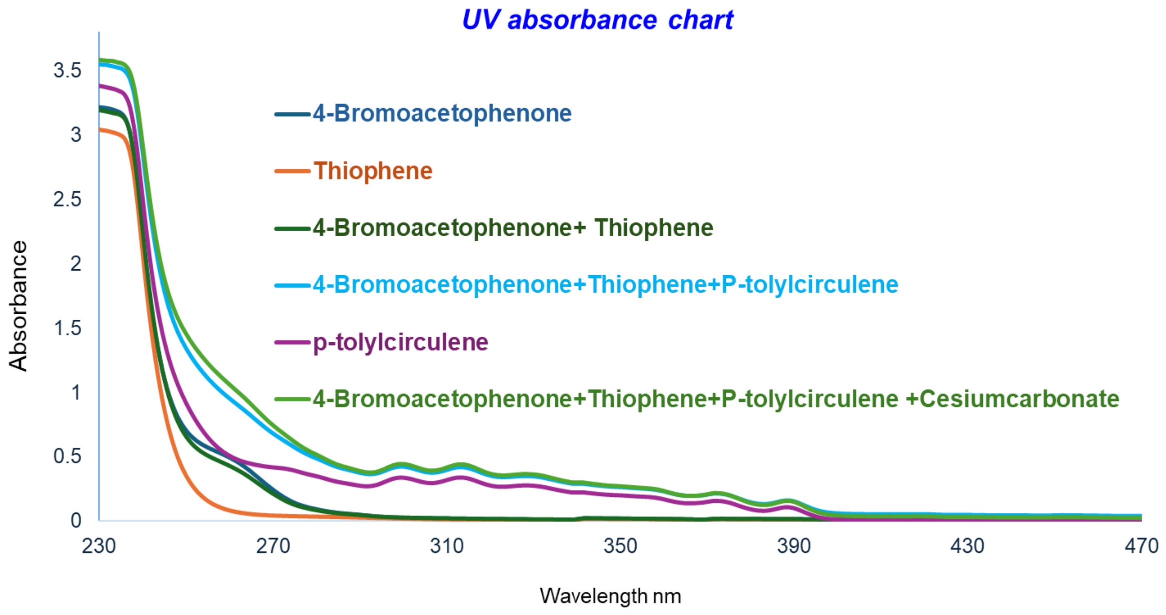

- ## Energy profiles, in kcal/mol, for the organo-photocatalytic arylation reaction

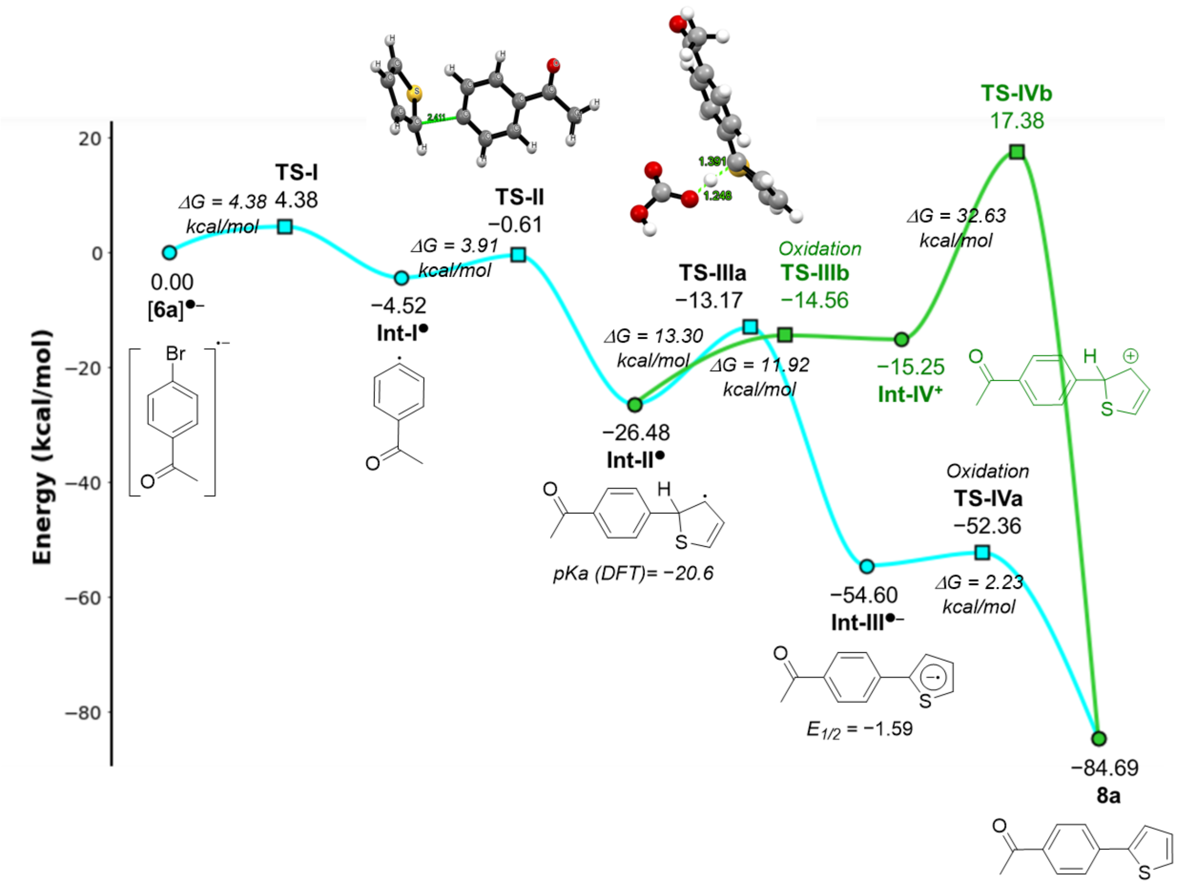

Calculated at the UB3LYP/6-31G+(d,p)/IEFPCM=DMSO. Grimme's dispersion with the original D3 damping function was applied as empirical dispersion correction to the optimized structures. All global minima were verified by the absence of imaginary frequencies, while all transition states were confirmed by the presence of a single imaginary frequency. Additionally, an Intrinsic Reaction Coordinate (IRC) analysis was performed to ensure that each transition state correctly connects the corresponding reactant and product minima along the reaction pathway.

- Theoretical prediction of the pKa values of neutral radicals Int-II & Int-V

$$pKa = \frac{G\ sol\ (H^{+}) + G\ sol\ (conjugate\ base^{-}) - \ G\ sol\ (Acid}{2.3\ RT}$$

We optimized the structures of the neutral radicals **Int-II** and **Int-V** (representing the acidic forms) and the radical anions **Int-III** and **Int-VI** (representing the conjugate basic forms) in DMSO using the UB3LYP/6-31+G(d,p) level of theory, incorporating D3BJ dispersion corrections *via* IOP(3/124=40). Additionally, we accounted for entropy and enthalpy corrections and optimized the solvated proton. Using these refined energy values, we applied the previously established equation to obtain the final results[41].

T(K)	298	
R (kcal/Kmol)	0.001987	
Etotal, H (kcal/mol)	-0.4973	-312.0602257
Molecule	$E_{(total)}$ Hartree	$E_{(total)}$ kcal/mol
**Int-II**	-937.164288	-588079.0252
**Int-III**	-936.711815	-587795.0943
pKa (on DMSO)	*ΔG* (kcal/mol)	*pKa*
**Int-II**	-28.12934594	-20.6

- DFT-based prediction of the reducing power of radical anions for most substrates

We followed Nicewicz and coworkers' protocol to calculate the redox potentials of super-reducing radical-anions[45].

$$E_{\frac{1}{2}}^{o,calc} = -\frac{(G_{298}[\mathrm{red}] - G_{298}[\mathrm{Ox}])}{n_e\mathcal{F}} - E_{\frac{1}{2}}^{o,SHE} + E_{\frac{1}{2}}^{o,SCE}$$

Substrate	G_{298} [**8**] anionic radical	G_{298} [**8**] product	Hartree $\Delta G^{o}_{1/2}$ [Ox]	kcal/mol $\Delta G^{o}_{1/2}$ [Ox]	($E^{calc}_{1/2}$)
**8a**	-936.711815	-936.607635	-0.10418	-65.37326254	-1.59
**8b**	-613.731342	-613.633639	-0.097703	-61.30892561	-1.76
**8c**	-633.143833	-633.044014	-0.099819	-62.63672196	-1.71
**8d**	-959.414966	-959.326113	-0.088853	-55.75552406	-2.00
**8f**	-673.409086	-673.330677	-0.078409	-49.20188273	-2.29
**8g**	-899.028250	-898.950224	-0.078026	-48.96154908	-2.30
**8h**	-728.252042	-728.159851	-0.092191	-57.85012907	-1.91
**8i**	-593.848918	-593.750196	-0.098722	-61.94835117	-1.74
**8j**	-920.120704	-920.025194	-0.09551	-59.93281153	-1.82
**8k**	-998.329442	-998.234016	-0.095426	-59.88010128	-1.83
**8l**	-844.715044	-844.625931	-0.089113	-55.91867484	-2.00
**8m**	-1038.591204	-1038.516127	-0.075077	-47.11104273	-2.38
**8n**	-960.370036	-960.305989	-0.064047	-40.18968464	-2.68
**8o**	-519.767934	-519.712286	-0.055648	-34.91928694	-2.91
**8p**	-574.435859	-574.332559	-0.1033	-64.8210599	-1.61
**8w**	-1035.748666	-1035.697985	-0.050681	-31.80247954	-3.04
**8x**	-998.278539	-998.200362	-0.078177	-49.05630203	-2.29
**8aa**	-960.556133	-960.513223	-0.04291	-26.92615373	-3.25
**8ab**	-496.535601	-496.465031	-0.07057	-44.28288671	-2.50

In the study, molecular geometries of all stationary points were optimized at the UB3LYP level of DFT with the 6-31G+(d,p) basis set with IEPCM model as solvation of DMSO. Grimme's dispersion with the original D3 damping function was applied as empirical dispersion correction to the optimized structures.

- Computational data for the suggested SET step

Following the computational approach proposed by Opatz *et al.*, we assess whether redox upconversion can be thermodynamically favorable when charge transfer is coupled with an exergonic chemical reaction[53].

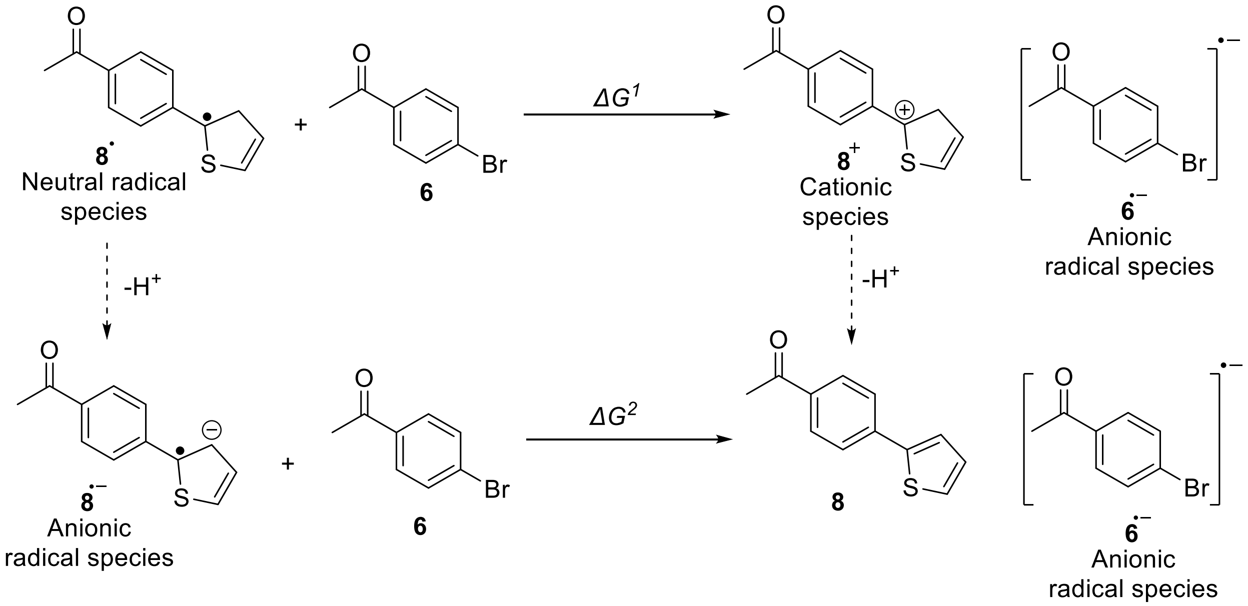

	8	[8]•−	8+	[8]•	6	[6]•−	ΔG^1	ΔG^2	$\Delta G^2 - \Delta G^1$
**8a**	-936.61	-936.71	-936.99	-937.16	-2955.96	-2956.06	45.02	3.70	-41.33
**8b**	-613.63	-613.73	-614.01	-614.18	-2955.96	-2956.06	40.78	-0.37	-41.15
**8c**	-633.04	-633.14	-633.46	-633.60	-2955.96	-2956.06	24.52	0.96	-23.56
**8d**	-959.33	-959.41	-959.73	-959.86	-2955.96	-2956.06	21.21	-5.92	-27.13
**8f**	-673.33	-673.41	-673.71	-673.87	-2895.59	-2895.68	45.92	-6.53	-52.46
**8g**	-898.95	-899.03	-899.33	-899.49	-2895.59	-2895.68	43.54	-6.77	-50.31
**8h**	-728.16	-728.25	-728.54	-728.71	-3070.49	-3070.58	47.45	2.79	-44.66
**8i**	-593.75	-593.85	-594.16	-594.30	-2916.66	-2916.77	22.25	-2.00	-24.25
**8j**	-920.03	-920.12	-920.43	-920.56	-2916.66	-2916.77	21.75	-4.02	-25.77
**8j**[a]	-920.03	-920.12	-920.43	-920.56	-805.13	-805.23	21.96	-3.81	-25.77
**8k**	-998.23	-998.33	-998.62	-998.77	-3070.27	-3070.38	24.89	-9.04	-33.93
**8l**	-844.63	-844.72	-845.02	-845.16	-2916.66	-2916.76	26.50	-7.99	-34.49

**8m**	-1038.52	-1038.59	-1038.91	-1039.07	-3110.56	-3110.64	53.24	-2.86	-56.09
**8n**	-960.31	-960.37	-960.71	-960.84	-2956.95	-2957.02	34.84	-7.91	-42.75
**8o**	-519.71	-519.77	-520.13	-520.26	-2842.63	-2842.73	20.54	-25.85	-46.39
**8p**	-574.33	-574.44	-574.71	-574.88	-2916.66	-2916.77	41.44	0.87	-40.57
**8w**	-1035.70	-1035.75	-1036.10	-1036.24	-3032.34	-3032.44	28.13	-29.66	-57.79
**8x**	-998.20	-998.28	-998.58	-998.74	-883.30	-883.39	44.12	-6.99	-51.12
**8aa**	-960.51	-960.56	-960.92	-961.05	-2957.16	-2957.25	22.05	-33.15	-55.20
**8ab**	-496.47	-496.54	-496.88	-497.02	-2819.38	-2819.45	40.87	-0.75	-41.62

In the study, molecular geometries of all stationary points were optimized at the UB3LYP level of DFT with the 6-31G+(d,p) basis set with IEPCM model as solvation of DMSO. Grimme's dispersion with the original D3 damping function was applied as empirical dispersion correction to the optimized structures. [a] Prepared from 6-Cl instead of 6-Br.

- Light/Dark experiment of compound 8d

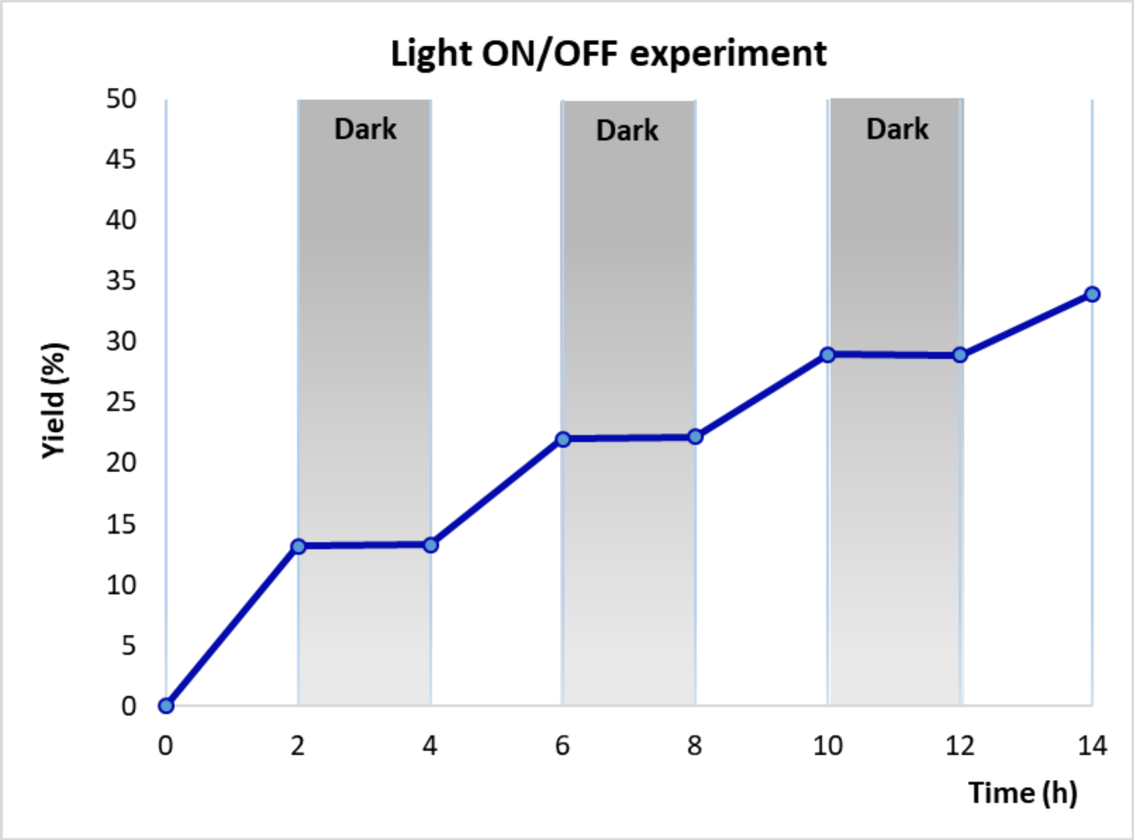

Although the light/dark experiment doesn't suggest chain reaction, this doesn't rule out the possibility of chain-based on HAT in all substrates.

- ## Investigation of oxidation and reduction potentials of substrates in organophotocatalytic arylation reactions

We followed Nicewicz and coworkers' protocol to calculate redox potentials for various substrates included in our substrate scope using the B3LYP functional, combined with the 6-31+G(d,p) basis set and the CPCM and IEFPCM solvent continuum models for MeCN and SMSO solvation. All calculations were performed using Gaussian 16, with structures subjected to geometry optimization and subsequent frequency calculations to confirm that the geometries were true minima and to compute free energies at 298 K. Solution-phase energies were referenced to SCE by subtracting 4.281 V (the absolute potential of SHE) and 0.141 V (the conversion of SHE to SCE) [45].

$$E_{\frac{1}{2}}^{o,calc} = -\frac{(G_{298}[\mathrm{red}] - G_{298}[\mathrm{Ox}])}{n_e \mathcal{F}} - E_{\frac{1}{2}}^{o,SHE} + E_{\frac{1}{2}}^{o,SCE}$$

Where n_e is the number of electrons transferred (here, n_e= 1), $\mathcal{F}$ is the Faraday constant (23.06 kcal mol^{-1} V^{-1}), $E_{1/2}^{o,SH}$ is the absolute value for the standard hydrogen electrode (SHE, 4.281 V), $E_{1/2}^{o,SCE}$ is the potential of the saturated calomel electrode (SCE) relative to SHE (−0.141 V), and G oxidized and G reduced are the Gibbs free energies as obtained from DFT calculations.

**Compound**	**Reduction Potential ($E^{calc}_{1/2}$)**	**Oxidation Potential ($E^{calc}_{1/2}$)**
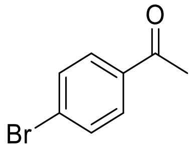	−1.76 (IEFPCM=MeCN)<br>−2.00 (CPCM=MeCN)<br>−1.75 (IEFPCM=DMSO)<br>−1.83 (CPCM=DMSO)	2.36 (IEFPCM=MeCN)<br>2.58 (CPCM=MeCN)<br>2.34 (IEFPCM=DMSO)<br>2.42 (CPCM=DMSO)
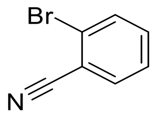	−2.02 (IEFPCM=MeCN)<br>−2.02 (CPCM=MeCN)<br>−2.01 (IEFPCM=DMSO)<br>−2.00 (CPCM=DMSO)	2.67 (IEFPCM=MeCN)<br>2.67 (CPCM=MeCN)<br>2.65 (IEFPCM=DMSO)<br>2.65 (CPCM=DMSO)
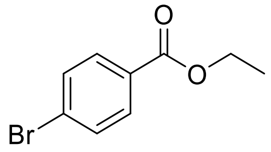	−2.05 (IEFPCM=MeCN)<br>−2.05 (CPCM=MeCN)<br>−2.03 (IEFPCM=DMSO)<br>−2.03 (CPCM=DMSO)	2.55 (IEFPCM=MeCN)<br>2.55 (CPCM=MeCN)<br>2.54 (IEFPCM=DMSO)<br>2.53 (CPCM=DMSO)
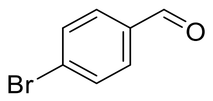	−1.66 (IEFPCM=MeCN)<br>−1.66 (CPCM=MeCN)<br>−1.65 (IEFPCM=DMSO)<br>−1.65 (CPCM=DMSO)	2.58 (IEFPCM=MeCN)<br>2.58 (CPCM=MeCN)<br>2.57 (IEFPCM=DMSO)<br>2.57 (CPCM=DMSO)
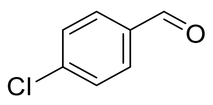	−1.67 (IEFPCM=MeCN)<br>−1.67 (CPCM=MeCN)<br>−1.66 (IEFPCM=DMSO)<br>−1.66 (CPCM=DMSO)	2.66 (IEFPCM=MeCN)<br>2.66 (CPCM=MeCN)<br>2.65 (IEFPCM=DMSO)<br>2.65 (CPCM=DMSO)

Compound	Reduction Potential ($E^{calc}_{1/2}$)	Oxidation Potential ($E^{calc}_{1/2}$)
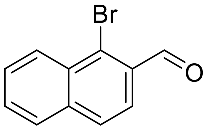	−1.45 (IEFPCM=MeCN)<br>−1.44 (CPCM=MeCN)<br>−1.43 (IEFPCM=DMSO)<br>−1.43 (CPCM=DMSO)	1.82 (IEFPCM=MeCN)<br>1.83 (CPCM=MeCN)<br>1.81 (IEFPCM=DMSO)<br>1.81 (CPCM=DMSO)
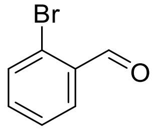	−1.67 (IEFPCM=MeCN)<br>−1.66 (CPCM=MeCN)<br>−1.65 (IEFPCM=DMSO)<br>−1.65 (CPCM=DMSO)	2.53 (IEFPCM=MeCN)<br>2.53 (CPCM=MeCN)<br>2.52 (IEFPCM=DMSO)<br>2.52 (CPCM=DMSO)
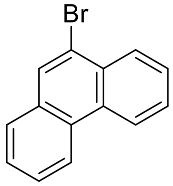	−2.27 (IEFPCM=MeCN)<br>−2.26 (CPCM=MeCN)<br>−2.26 (IEFPCM=DMSO)<br>−2.25 (CPCM=DMSO)	1.54 (IEFPCM=MeCN)<br>1.54 (CPCM=MeCN)<br>1.53 (IEFPCM=DMSO)<br>1.53 (CPCM=DMSO)
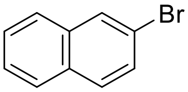	−2.35 (IEFPCM=MeCN)<br>−2.35 (CPCM=MeCN)<br>−2.34 (IEFPCM=DMSO)<br>−2.33 (CPCM=DMSO)	1.66 (IEFPCM=MeCN)<br>1.66 (CPCM=MeCN)<br>1.65 (IEFPCM=DMSO)<br>1.65 (CPCM=DMSO)
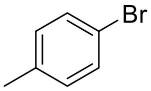	−1.81 (IEFPCM=MeCN)<br>−1.80 (CPCM=MeCN)<br>−1.79 (IEFPCM=DMSO)<br>−1.78 (CPCM=DMSO)	1.92 (IEFPCM=MeCN)<br>2.01 (CPCM=MeCN)<br>1.99 (IEFPCM=DMSO)<br>2.00 (CPCM=DMSO)
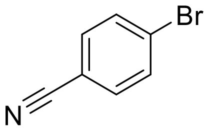	−2.06 (IEFPCM=MeCN)<br>−2.06 (CPCM=MeCN)<br>−2.05 (IEFPCM=DMSO)<br>−2.05 (CPCM=DMSO)	2.62 (IEFPCM=MeCN)<br>2.62 (CPCM=MeCN)<br>2.60 (IEFPCM=DMSO)<br>2.60 (CPCM=DMSO)
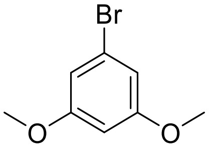	−1.78 (IEFPCM=MeCN)<br>−1.77 (CPCM=MeCN)<br>−1.76 (IEFPCM=DMSO)<br>−1.75 (CPCM=DMSO)	1.66 (IEFPCM=MeCN)<br>1.66 (CPCM=MeCN)<br>1.64 (IEFPCM=DMSO)<br>1.64 (CPCM=DMSO)
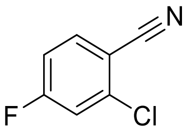	−2.00 (IEFPCM=MeCN)<br>−2.00 (CPCM=MeCN)<br>−1.99 (IEFPCM=DMSO)<br>−1.99 (CPCM=DMSO)	2.85 (IEFPCM=MeCN)<br>2.85 (CPCM=MeCN)<br>2.83 (IEFPCM=DMSO)<br>2.83 (CPCM=DMSO)
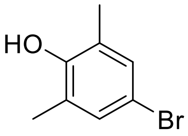	−1.84 (IEFPCM=MeCN)<br>−1.83 (CPCM=MeCN)<br>−1.82 (IEFPCM=DMSO)<br>−1.81 (CPCM=DMSO)	1.54 (IEFPCM=MeCN)<br>1.54 (CPCM=MeCN)<br>1.52 (IEFPCM=DMSO)<br>1.53 (CPCM=DMSO)

Compound	Reduction Potential ($E^{calc}_{1/2}$)	Oxidation Potential ($E^{calc}_{1/2}$)
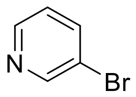	−2.48 (IEFPCM=MeCN)<br>−2.48 (CPCM=MeCN)<br>−2.47 (IEFPCM=DMSO)<br>−2.47 (CPCM=DMSO)	2.57 (IEFPCM=MeCN)<br>2.57 (CPCM=MeCN)<br>2.56 (IEFPCM=DMSO)<br>2.56 (CPCM=DMSO)
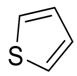	−3.14 (IEFPCM=MeCN)<br>−3.14 (CPCM=MeCN)<br>−3.13 (IEFPCM=DMSO)<br>−3.13 (CPCM=DMSO)	1.99 (IEFPCM=MeCN)<br>2.00 (CPCM=MeCN)<br>1.98 (IEFPCM=DMSO)<br>1.98 (CPCM=DMSO)
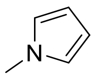	−3.91 (IEFPCM=MeCN)<br>−3.91 (CPCM=MeCN)<br>−3.90 (IEFPCM=DMSO)<br>−3.90 (CPCM=DMSO)	1.28 (IEFPCM=MeCN)<br>1.26 (CPCM=MeCN)<br>1.26 (IEFPCM=DMSO)<br>1.24 (CPCM=DMSO)
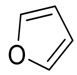	−4.12 (IEFPCM=MeCN)<br>−4.11 (CPCM=MeCN)<br>−4.10 (IEFPCM=DMSO)<br>−4.10 (CPCM=DMSO)	1.95 (IEFPCM=MeCN)<br>1.95 (CPCM=MeCN)<br>1.94 (IEFPCM=DMSO)<br>1.94 (CPCM=DMSO)
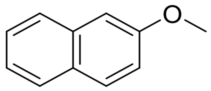	−2.61 (IEFPCM=MeCN)<br>−2.61 (CPCM=MeCN)<br>−2.59 (IEFPCM=DMSO)<br>−2.59 (CPCM=DMSO)	1.31 (IEFPCM=MeCN)<br>1.31 (CPCM=MeCN)<br>1.30 (IEFPCM=DMSO)<br>1.30 (CPCM=DMSO)
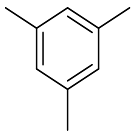	−3.42 (IEFPCM=MeCN)<br>−3.42 (CPCM=MeCN)<br>−3.41 (IEFPCM=DMSO)<br>−3.40 (CPCM=DMSO)	1.81 (IEFPCM=MeCN)<br>1.81 (CPCM=MeCN)<br>1.79 (IEFPCM=DMSO)<br>1.79 (CPCM=DMSO)
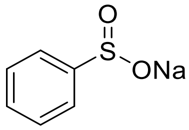	−3.00 (IEFPCM=MeCN)<br>−2.95 (CPCM=MeCN)<br>−2.89 (IEFPCM=DMSO)<br>−2.93 (CPCM=DMSO)	0.95 (IEFPCM=MeCN)<br>0.91 (CPCM=MeCN)<br>0.85 (IEFPCM=DMSO)<br>0.89 (CPCM=DMSO)
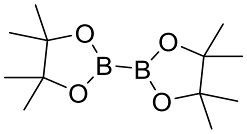	−2.86 (IEFPCM=MeCN)<br>−2.87 (CPCM=MeCN)<br>−2.85 (IEFPCM=DMSO)<br>−2.85 (CPCM=DMSO)	2.42 (CPCM=DMSO)
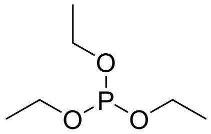	−3.04 (IEFPCM=MeCN)<br>−3.05 (CPCM=MeCN)<br>−3.05 (IEFPCM=DMSO)	1.77 (IEFPCM=MeCN)

- Spin density analysis of neutral and anionic radical intermediates in organophotocatalytic arylation mechanisms

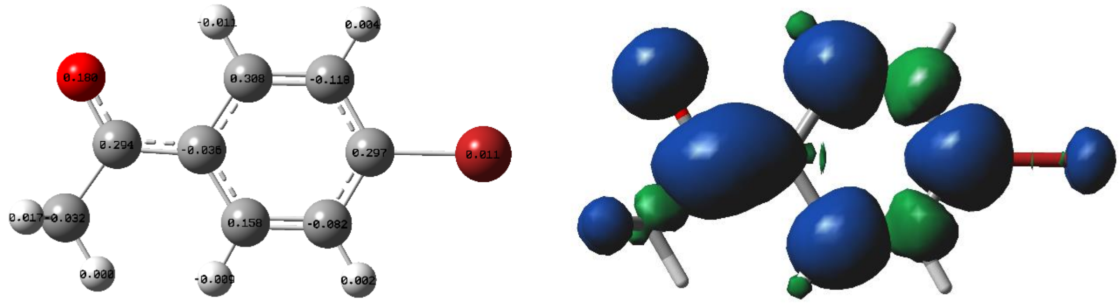

Mulliken spin density contour maps for [**6a**]$^{\cdot-}$ calculated at the B3LYP/6-31G+(d,p)/IEFPCM=DMSO (isoval = 0.001).

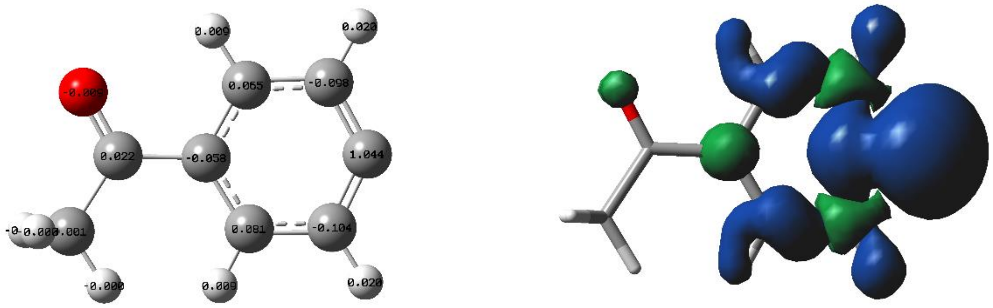

Mulliken spin density contour maps for **Int-I** calculated at the B3LYP/6-31G+(d,p)/IEFPCM=DMSO (isoval = 0.001).

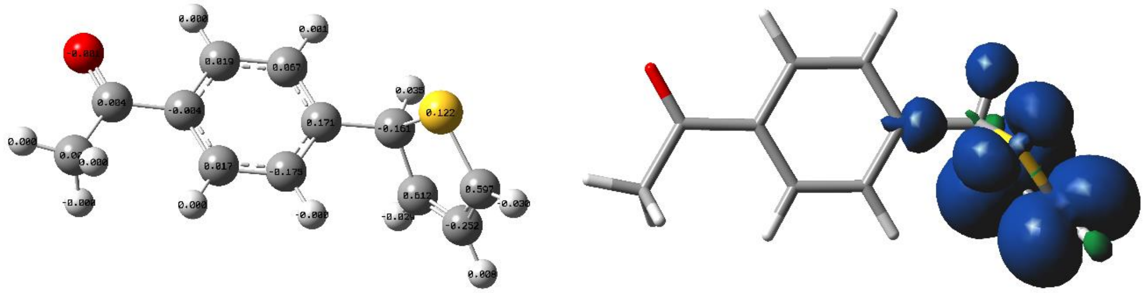

Mulliken spin density contour maps for **Int-II** calculated at the B3LYP/6-31G+(d,p)/IEFPCM=DMSO (isoval = 0.002).

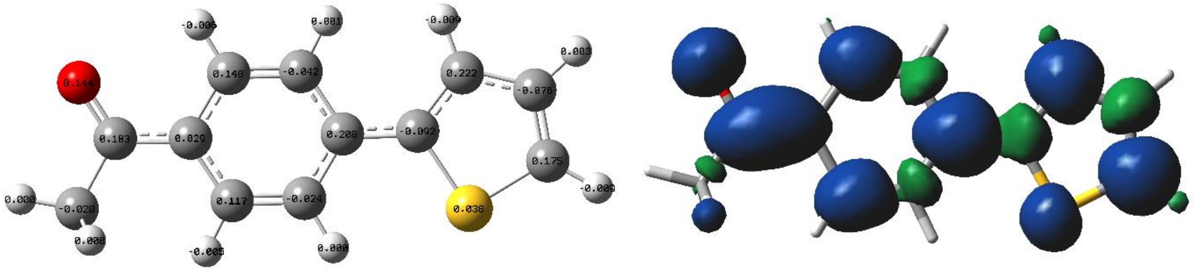

Mulliken spin density contour maps for **Int-IV** calculated at the B3LYP/6-31G+(d,p)/IEFPCM=DMSO (isoval = 0.001).

- Calculation of quantum efficiency

The quantum efficiency or quantum yield (QY) of the photochemical reaction was determined by evaluating the ratio of the number of reacted molecules to the number of absorbed photons using the following equation[54,55]:

$$QY = \frac{\text{Number of molecules reacted}}{\text{Number of photons absorbed}}$$

The number of photons absorbed (N_{photon}) was obtained using the photon flux (I) and the irradiation time (t), based on the relation:

$$N_{photon} = \text{photon flux } (I) \times \text{time}$$

$$I = \frac{Power\ of\ light\ source\ (P)\ in\ W\ (\frac{J}{s})}{Energy\ of\ single\ photon\ (J)} = \frac{P}{h\,.\,C\ /\ \lambda} = 9.32\ \times\ 10^{17}\ Photons/s$$

Whereas $\boldsymbol{P}$ (0.507 W) represents the power of the irradiated light form LED light source, $\boldsymbol{h}$ (6.626×10^{-34} J·s) is Planck's constant, $\boldsymbol{c}$ (2.998×10^{8} m/s) is the speed of light, and $\boldsymbol{\lambda}$ ($365 \times 10^{-}9$ m) is the wavelength of the monochromatic light.

$$N_{photon} = 9.32\ \times\ 10^{17} \times 86400 = 8.05 \times 10^{22}\ photons$$

Whereas $\text{time} = 86400\ \text{s}\ (24\ \text{h})$

The number of photons absorbed should be corrected by accounting for the fraction of light absorbed at 365 nm by our photocatalyst (circulene), and its quantum yield to ensure an accurate estimation of the number of photons absorbed[55].

$$N_{photon} = 8.05 \times 10^{22}\ \times 0.387\ \times 0.065 = 2.02\ \ \times 10^{21}\ photons$$

Given the quantum yield of circulene (6.5%) and the fraction of light absorbed at 365 nm (with an absorbance of 0.2127 a.u.), the absorbed fraction is calculated as $f = 1 - 10^{-A} = 0.387$).

$$\text{Number of molecules reacted} = \text{No. of moles} \times \text{Avogadro}' \text{s number } (6.022 \times 10^{23})$$

$$QY\ for\ \mathbf{8a} = \frac{\text{Number of molecules reacted}}{\text{Number of photons absorbed}} = \frac{0.0001 \times 0.62 \times 6.022 \times 10^{23}}{2.02 \times 10^{21}} = 1.84 \times 10^{-2}$$

Compound	QY	Compound	QY	Compound	QY
**8a**	0.009-0.018	**8k**	0.006	**8u**	0.012
**8b**	0.012	**8l**	0.014	**8v**	0.004-0.010
**8c**	0.024-0.029	**8m**	0.006-0.009	**8w**	0.006
**8d**	0.021-0.027	**8n**	0.016	**8x**	0.018
**8e**	0.023	**8o**	0.012	**8y**	0.025
**8f**	0.013	**8p**	0.009	**8z**	0.023
**8g**	0.027	**8q**	0.011	**8aa**	0.006
**8h**	0.018-0.023	**8r**	0.012-0.020	**8ab**	0.010
**8i**	0.022	**8s**	0.013		
**8j**	0.011-0.021	**8t**	0.022		

All QYs are around 10^{-2} ~ 10^{-1} values. However, this doesn't rule out the possibility of chain-based on HAT all substrates.

12. **Supplementary Note 5:** *X-ray crystallographic analysis of dioxaza[8]circulenes **3a** and **3b***

The sums of all dihedral angles of the inner circulene rims (φ) of **3a** and **3b**

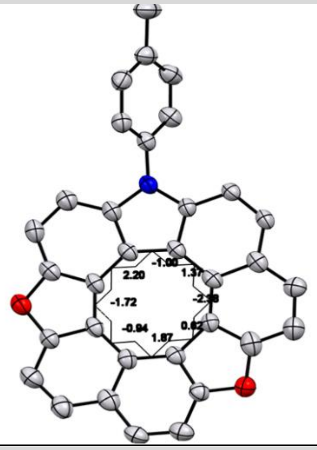

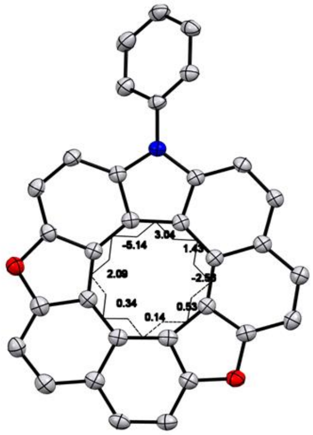

Angles between the heteroatoms of **3a**

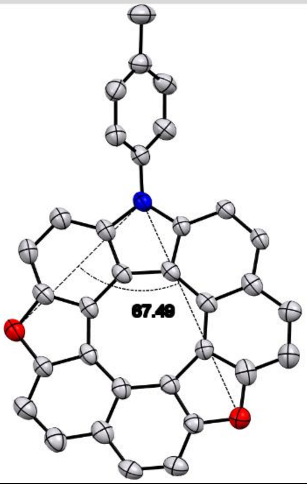

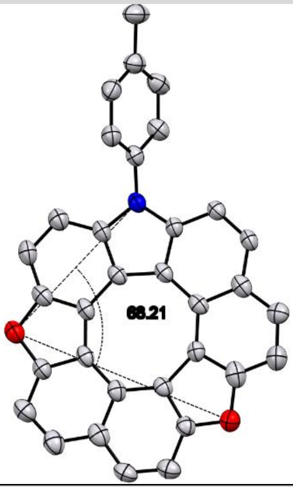

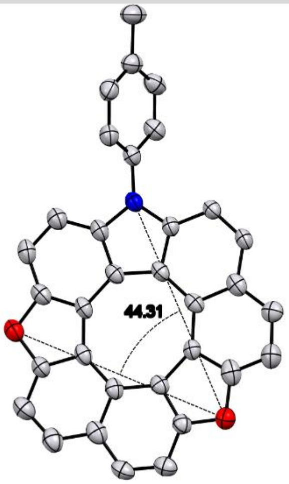

Angles between the heteroatoms of **3b**

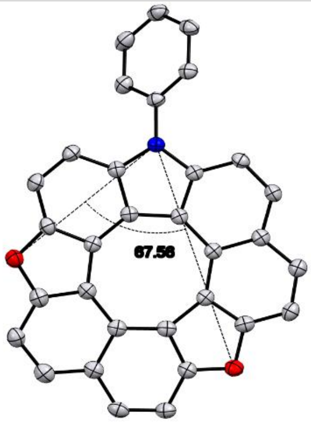

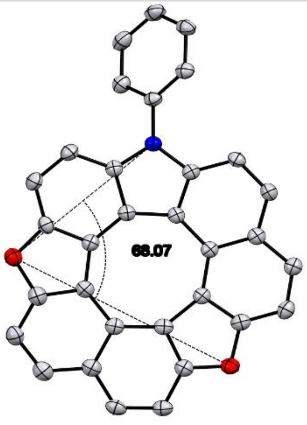

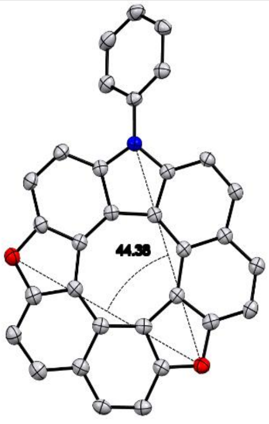

Distances between the centroids of heterocycles of **3a** and **3b**

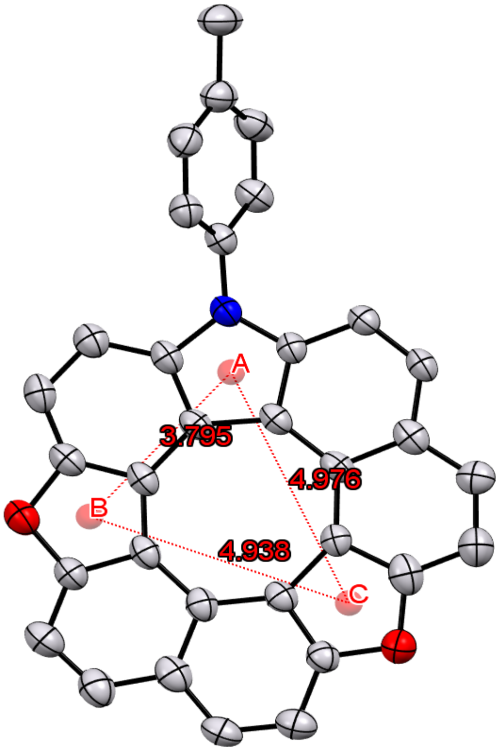

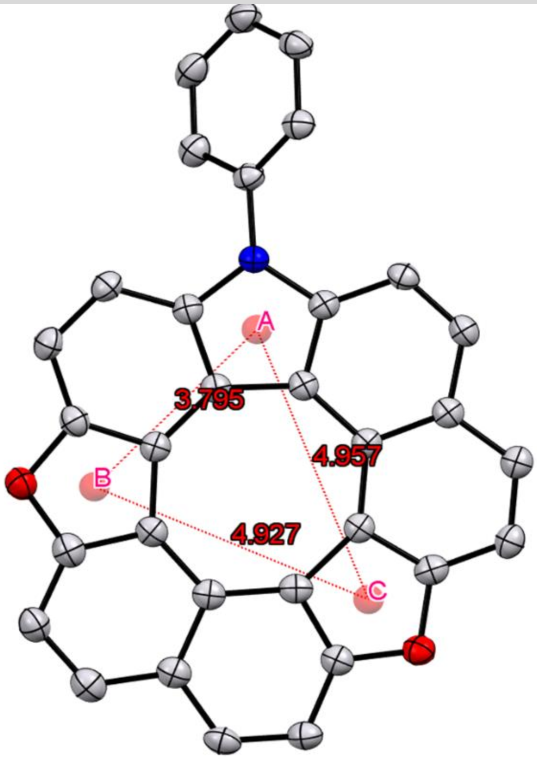

Dihedral angles between the circulene and *N*-substituent of **3a**

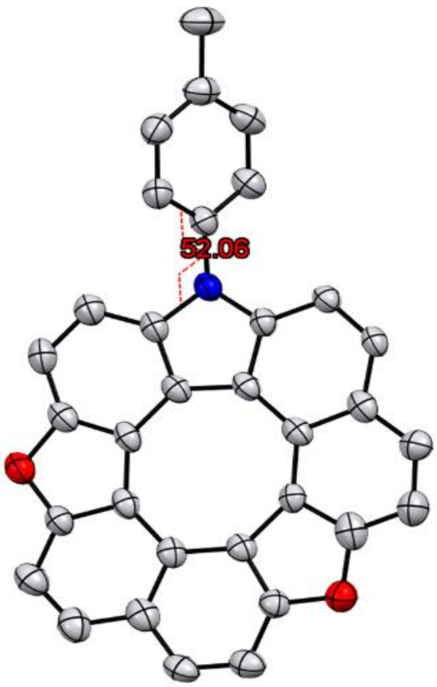

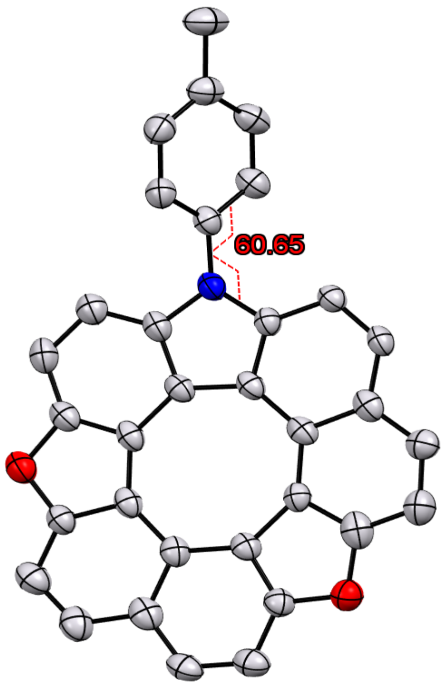

Dihedral angles between the circulene and *N*-substituent of **3b**

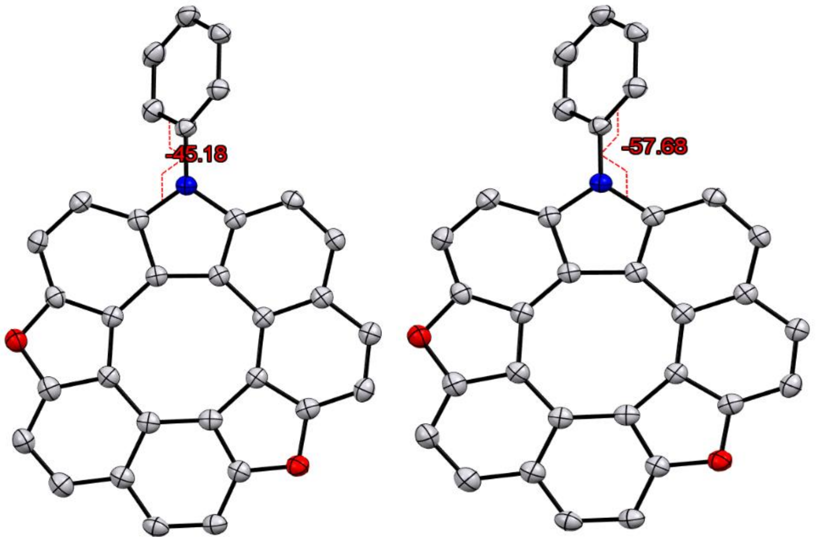

The sectors of furans, pyrrole, and benzene rings in **3a**

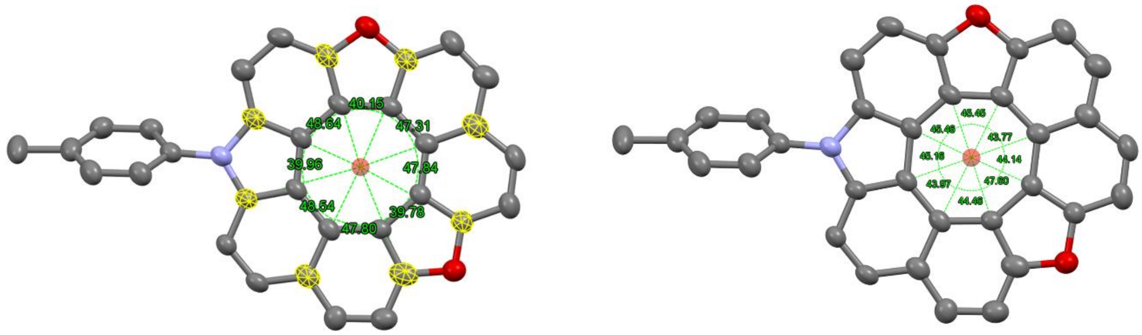

Co-facial lamellar packing of **3a**

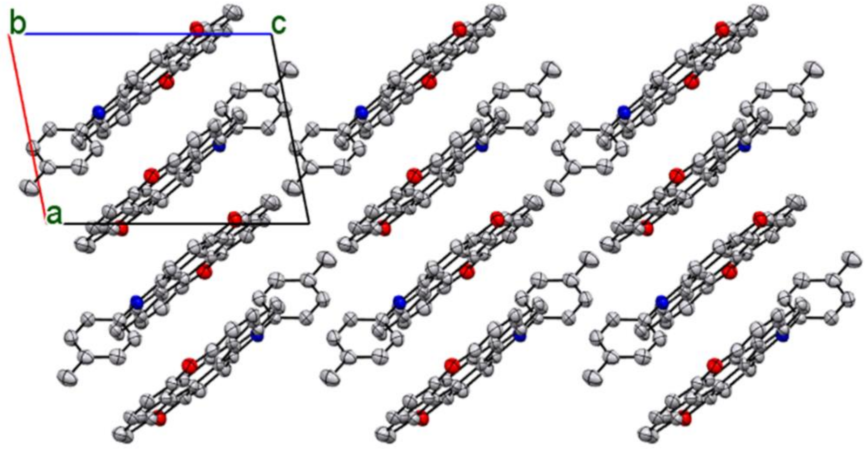

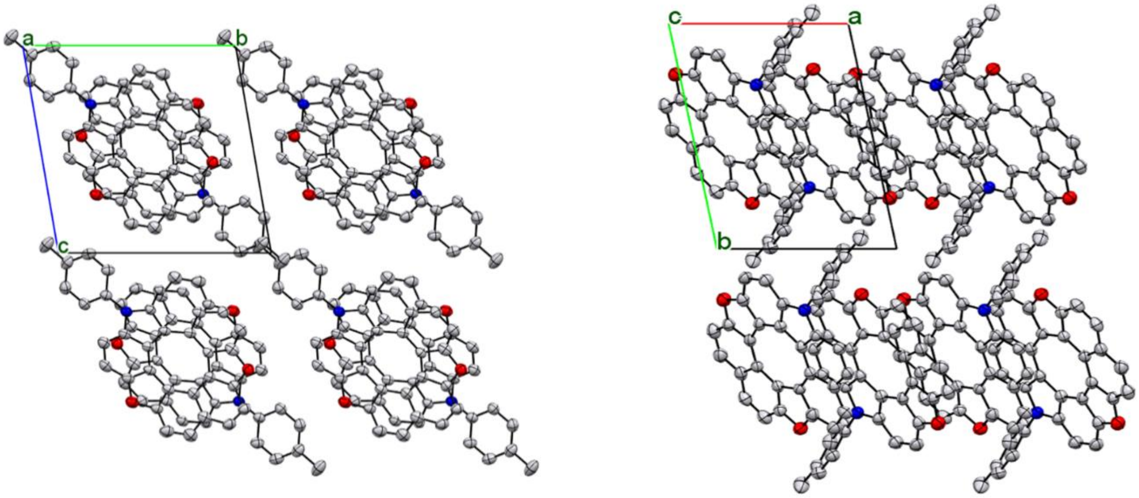

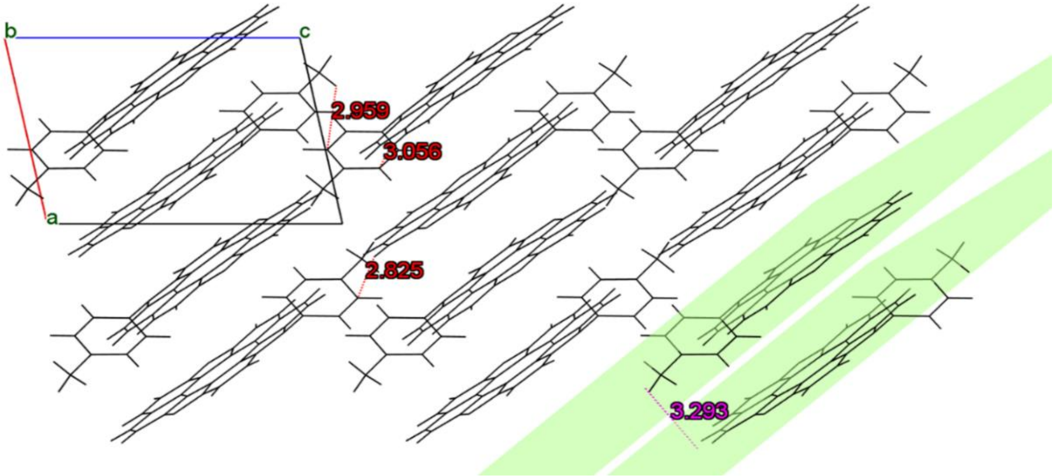

herringbone packing with p – p overlap between adjacent molecules of **3b**

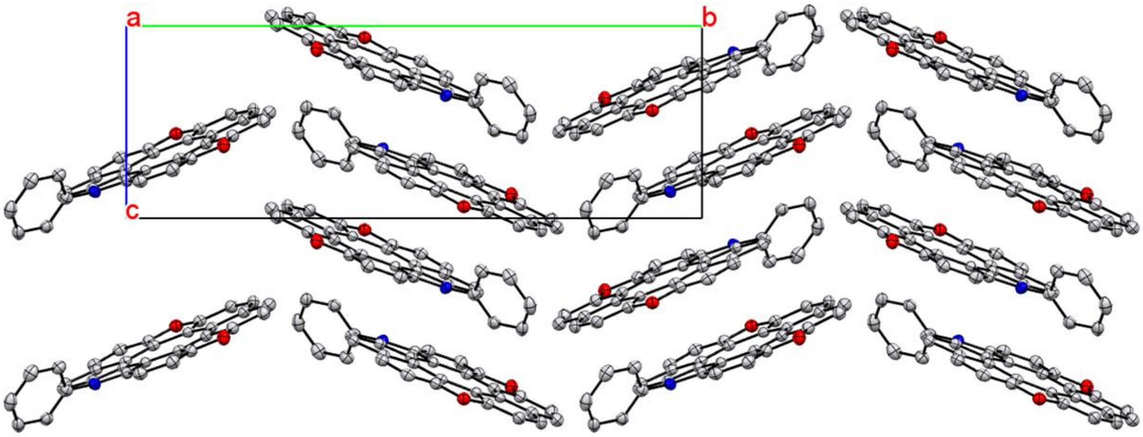

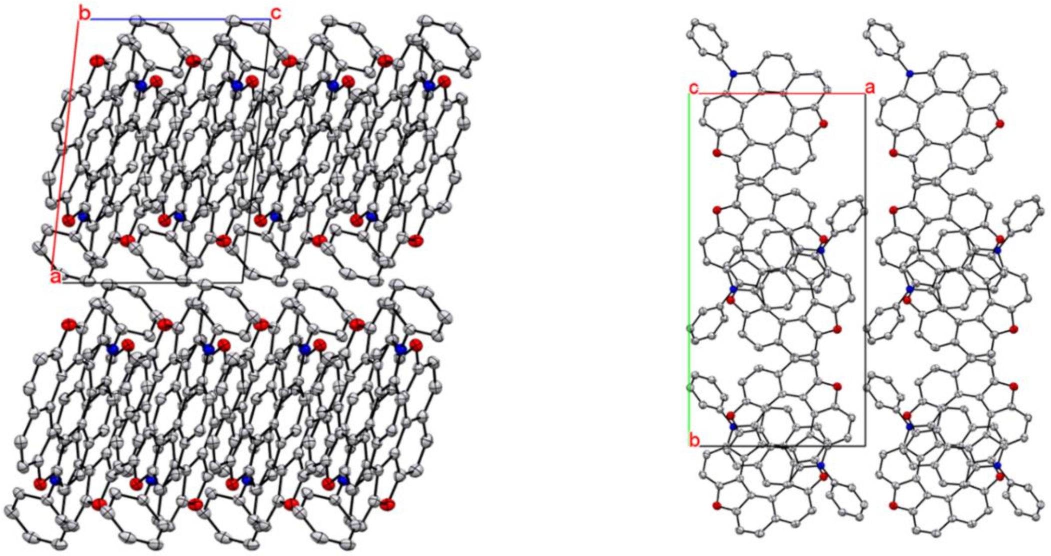
b
c
a
c
a
b

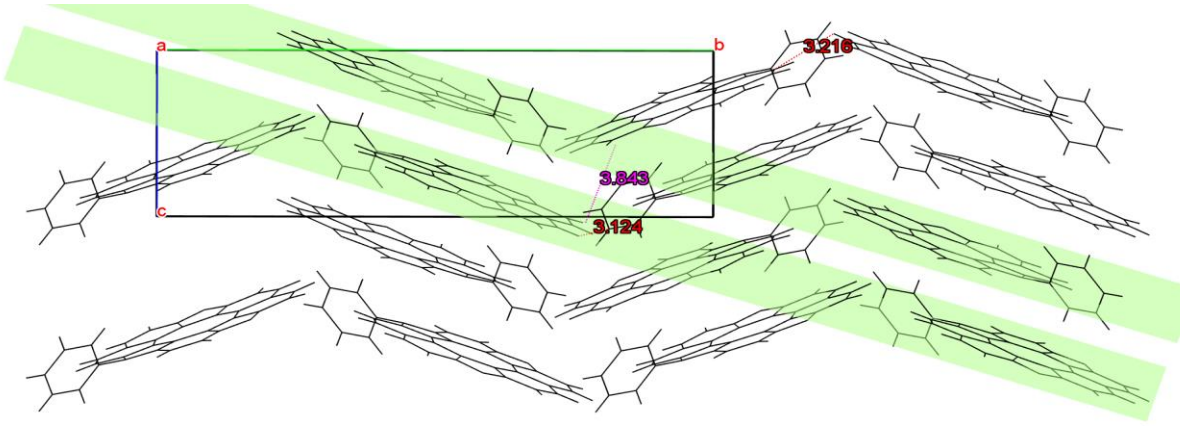
a
b
c
3.216
3.843
3.124

13. Supplementary Note 6: *DFT calculations to study the structural and optoelectronic features of circulenes*

All DFT calculations were performed using the Gaussian 16 and Gaussian 9 packages of programs[56,57]. The geometries of the structures of dioxaza[8]circulenes were optimized at both the ground S_0 and excited S_1 states, calculated with the MN15/cc-pVTZ level of theory[58]. All stationary points were identified as stable minima by frequency calculations, and the geometry optimization was achieved using the standard criteria in Gaussian software[25,26,59,60]. The nucleus-independent chemical shift (NICS)[61,62] indices were calculated at the center of each ring ($NICS_{(0)}$) and 1 Å above/below the center ($NICS_{(1)}$) within the gauge-independent atomic orbital (GIAO) approximation at MN15/cc-pVTZ levels of theory[63] and visualized using py.Aroma: an intuitive graphical user interface for diverse aromaticity analyses[64]. For the anisotropy of the induced current density (AICD) simulations, the AICD-3.0.4 software was used[65]. TD-DFT calculations were performed directly on Cartesian coordinates obtained from the crystal structures of dioxaza[8]circulenes at the MN15/cc-pVTZ level of theory and on those optimized at the lowest energy singlet excited state (S_1)[66]. The electric and magnetic transition dipole moments of dioxaza[8]circulenes were calculated using Multiwfn[67] and visualized using VMD software[68].

- Optimization of basis sets for geometries of dioxaza[8]circulenes **3a** and **3b**

Table S8. Comparison of basis sets for geometry optimization of dioxaza[8]circulene **3a**

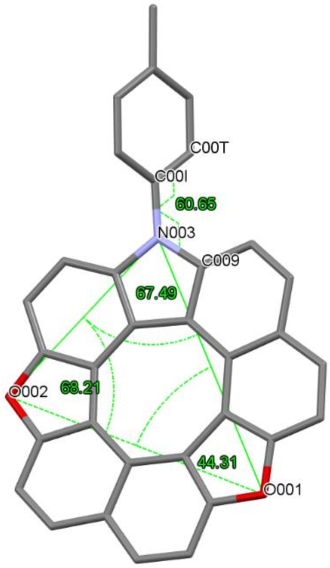

X-ray crystal structure of **3a**

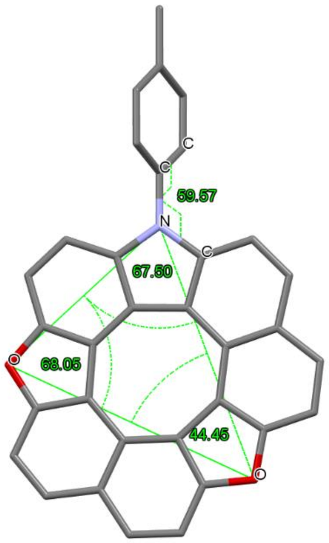

Optimized structure at cc-pVTZ MN15

	Structure or basis set	Torsion angle (C^9,N^3,C^A,C^M)	Angle (O^1,**N^3**,O^1)	Angle (N^3,**O^2**,O^1)	Angle (O^2,**O^1**,N^3)	Cost (hour)
**3a**	Experimental (X-ray)	60.65	67.49	68.21	44.31	
	B3LYP/6-31G	66.58	67.66	67.95	44.39	6.2
	B3LYP/6-31G(d)	65.31	67.51	68.07	44.42	9.3
	B3LYP/6-311G(d)	71.23	67.52	68.05	44.43	6.5
	B3LYP/6-311G(d,p)	72.80	67.53	68.03	44.43	7.1
	B3LYP/6-311+G(d,p)	74.22	67.52	68.05	44.44	52.6
	B3LYP/6-311+G(2d,p)	75.79	67.54	68.04	44.42	91.3
	LSDA/6-31G	51.15	67.68	67.95	44.37	1.7
	LSDA/6-31G(d)	50.89	67.54	68.07	44.39	3.8
	LSDA/6-311G(d)	52.62	67.55	68.05	44.4	8.3
	LSDA/6-311G(d,p)	52.58	67.55	68.05	44.4	9.4
	LSDA/6-311+G(d,p)	53.48	67.55	68.05	44.4	25.3
	LSDA/6-311+G(2d,p)	53.16	67.56	68.04	44.39	18.4
	BVP86/6-31G	62.27	67.75	67.9	44.36	7.2
	BVP86/6-31G(d)	61.14	67.59	68.03	44.38	12.0
	BVP86/6-311G(d)	64.39	67.6	68.02	44.39	5.0
	BVP86/6-311G(d,p)	65.25	67.59	68.02	44.38	5.9
	BVP86/6-311+G(d,p)	66.09	67.6	68.01	44.39	31.3
	BVP86/6-311+G(2d,p)	66.77	67.62	68.00	44.38	26.0
	CAM-B3LYP/6-31G	66.71	67.63	67.95	44.42	4.5
	CAM-B3LYP/6-31G(d)	65.50	67.5	68.06	44.44	11.8
	CAM-B3LYP/6-311G(d)	71.01	67.51	68.04	44.45	9.3
	CAM-B3LYP/6-311G(d,p)	72.58	67.51	68.04	44.45	19.9
	CAM-B3LYP/6-311+G(d,p)	74.46	67.5	68.05	44.45	63.3
	CAM-B3LYP/6-311+G(2d,p)	75.90	67.52	68.03	44.45	157.7

B3PW91/6-31G	66.71	67.68	67.94	44.38	4.0
B3PW91/6-31G(d)	65.17	67.53	68.06	44.41	12.3
B3PW91/6-311G(d)	69.15	67.54	68.04	44.42	6.3
B3PW91/6-311G(d,p)	70.63	67.51	68.11	44.39	196.3
B3PW91/6-311+G(d,p)	70.84	67.54	68.04	44.43	55.1
B3PW91/6-311+G(2d,p)	72.00	67.57	68.02	44.41	36.8
mPW1PW91/6-31G	65.87	67.67	67.94	44.39	4.3
mPW1PW91/6-31G(d)	64.22	67.53	68.06	44.41	8.4
mPW1PW91/6-311G(d)	67.69	67.54	68.04	44.42	5.6
mPW1PW91/6-311G(d,p)	68.94	67.53	68.05	44.41	19.5
mPW1PW91/6-311+G(d,p)	69.42	67.53	68.04	44.43	54.9
mPW1PW91/6-311+G(2d,p)	70.48	67.55	68.03	44.42	44.3
PBEPBE/6-31G	60.53	67.75	67.91	44.35	4.1
PBEPBE/6-31G(d)	59.81	67.59	68.04	44.37	15.7
PBEPBE/6-311G(d)	62.74	67.59	68.02	44.38	5.0
PBEPBE/6-311G(d,p)	63.29	67.6	68.02	44.38	15.1
PBEPBE/6-311+G(d,p)	64.70	67.6	68.02	44.38	36.5
PBEPBE/6-311+G(2d,p)	65.03	67.62	68.00	44.38	26.7
TPSSTPSS/6-31G	61.39	67.84	67.79	44.37	63.7
TPSSTPSS/6-31G(d)	60.45	67.60	68.03	44.37	11.3
TPSSTPSS/6-311G(d)	63.05	67.59	68.02	44.39	6.4
TPSSTPSS/6-311G(d,p)	63.81	67.6	68.02	44.39	7.2
TPSSTPSS/6-311+G(d,p)	64.74	67.61	68.00	44.39	47.3
TPSSTPSS/6-311+G(2d,p)	65.14	67.61	68.00	44.39	27.0
WB97XD/6-31G	66.26	67.68	67.92	44.41	5.3
WB97XD/6-31G(d)	64.92	67.54	68.04	44.42	13.0
WB97XD/6-311G(d)	67.87	67.54	68.03	44.43	9.5
WB97XD/6-311G(d,p)	68.62	67.54	68.03	44.43	28.9
WB97XD/6-311+G(d,p)	69.41	67.54	68.03	44.44	82.1
WB97XD/6-311+G(2d,p)	70.41	67.58	68.00	44.43	68.6
PBE0/6-31G	64.77	67.67	67.93	44.40	55.2
PBE0/6-31G(d)	62.86	67.54	68.05	44.41	250.7
PBE0/6-311G(d)	64.82	67.53	68.05	44.42	754.1
6-31G MN15	59.23	67.58	68.00	44.42	4.2
6-31G(d) MN15	58.68	67.47	68.09	44.45	11.7
6-311G(d) MN15	60.49	67.48	68.07	44.45	21.7
6-311G(d,p) MN15	60.86	67.48	68.07	44.45	21.9
6-311+G(d,p) MN15	61.70	67.49	68.06	44.45	105.0
6-311+G(2d,p) MN15	61.40	67.49	68.06	44.45	111.9
6-311++G(2d,p) MN15	61.40	67.49	68.06	44.45	146.28
6-311++G(d,p) MN15	61.62	67.48	68.07	44.45	80.68
cc-pVDZ MN15	61.73	67.48	68.07	44.45	9.48
**cc-pVTZ MN15**	**59.57**	**67.50**	**68.05**	**44.45**	**85.38**
cc-pVTZ MN15/ PCM = $CHCl_3$	61.90	67.47	68.07	44.46	199.8
cc-pVQZ MN15	59.63	67.50	68.06	44.45	3648.27
LanL2DZ MN15	59.92	67.62	67.97	44.42	5.02
LanL2MB MN15	51.91	67.79	67.88	44.33	3.57
DGDZVP2 MN15	59.54	67.55	68.02	44.43	15.80

Red color indicates that the deviation of the dihedral angle is less than 2%

Table S9. Comparison of basis sets for geometry optimization of dioxaza[8]circulene **3b**

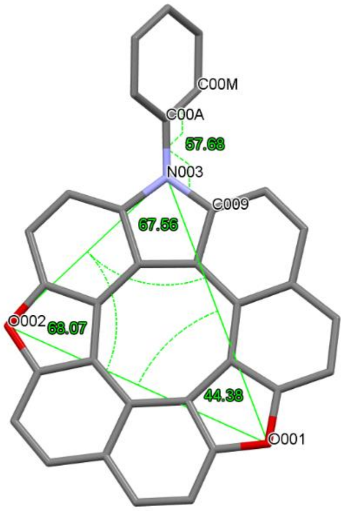

X-ray crystal structure of **3b**

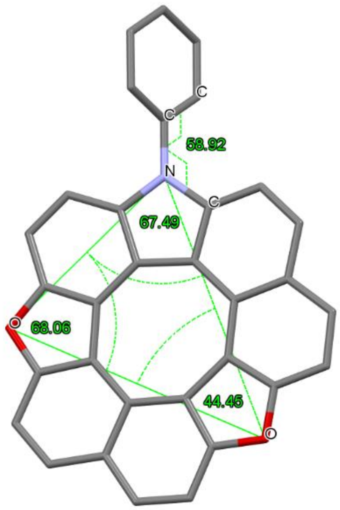

Optimized structure at cc-pVTZ MN15

	Structure or basis set	Torsion angle (C^9,N^3,C^A,C^M)	Angle (O^1,$\mathbf{N^3}$,O^1)	Angle (N^3,$\mathbf{O^2}$,O^1)	Angle (O^2,$\mathbf{O^1}$,N^3)	Cost (hour)
**3b**	Experimental (X-ray)	57.68	67.56	68.07	44.38	
	B3LYP/6-31G	65.22	67.65	67.95	44.4	1.1
	B3LYP/6-31G(d)	64.45	67.51	68.07	44.42	3.5
	B3LYP/6-311G(d)	69.37	67.52	68.05	44.43	9.2
	B3LYP/6-311G(d,p)	70.48	67.53	68.04	44.43	6.7
	B3LYP/6-311+G(d,p)	71.16	67.51	68.06	44.43	22.9
	B3LYP/6-311+G(2d,p)	73.02	67.54	68.04	44.42	41.6
	LSDA/6-31G	50.87	67.66	67.96	44.38	28.8
	LSDA/6-31G(d)	50.55	67.53	68.08	44.4	2.7
	LSDA/6-311G(d)	52.32	67.54	68.06	44.4	2.6
	LSDA/6-311G(d,p)	52.12	67.54	68.06	44.4	3.2
	LSDA/6-311+G(d,p)	52.88	67.54	68.06	44.4	10.0
	LSDA/6-311+G(2d,p)	52.45	67.55	68.05	44.4	16.1
	BVP86/6-31G	61.1	67.76	67.89	44.35	1.1
	BVP86/6-31G(d)	60.32	67.59	68.03	44.38	2.9
	BVP86/6-311G(d)	63.47	67.59	68.02	44.39	4.3
	BVP86/6-311G(d,p)	64.05	67.59	68.02	44.39	4.1
	BVP86/6-311+G(d,p)	64.66	67.6	68.01	44.39	15.9
	BVP86/6-311+G(2d,p)	65.38	67.62	68	44.38	21.6
	CAM-B3LYP/6-31G	65.27	67.62	67.96	44.43	2.1
	CAM-B3LYP/6-31G(d)	64.48	67.49	68.07	44.44	5.2
	CAM-B3LYP/6-311G(d)	69.06	67.5	68.05	44.45	8.2
	CAM-B3LYP/6-311G(d,p)	70.15	67.5	68.05	44.45	9.7
	CAM-B3LYP/6-311+G(d,p)	71.07	67.49	68.05	44.46	33.1
	CAM-B3LYP/6-311+G(2d,p)	72.77	67.53	68.02	44.45	60.3
	B3PW91/6-31G	65.44	67.67	67.94	44.39	1.6
	B3PW91/6-31G(d)	64.23	67.52	68.07	44.41	3.4

B3PW91/6-311G(d)	67.55	67.54	68.05	44.41	6.2
B3PW91/6-311G(d,p)	68.76	67.54	68.05	44.41	6.8
B3PW91/6-311+G(d,p)	68.52	67.54	68.05	44.41	20.1
B3PW91/6-311+G(2d,p)	69.82	67.56	68.02	44.41	38.5
mPW1PW91/6-31G	64.63	67.67	67.94	44.39	1.5
mPW1PW91/6-31G(d)	63.39	67.52	68.07	44.41	2.7
mPW1PW91/6-311G(d)	66.33	67.53	68.05	44.42	5.3
mPW1PW91/6-311G(d,p)	67.23	67.53	68.05	44.42	6.0
mPW1PW91/6-311+G(d,p)	67.36	67.53	68.05	44.42	18.1
mPW1PW91/6-311+G(2d,p)	68.48	67.55	68.03	44.42	36.5
PBEPBE/6-31G	59.71	67.74	67.91	44.35	1.3
PBEPBE/6-31G(d)	59.09	67.58	68.05	44.37	1.8
PBEPBE/6-311G(d)	61.99	67.59	68.02	44.39	4.1
PBEPBE/6-311G(d,p)	62.39	67.59	68.03	44.38	3.7
PBEPBE/6-311+G(d,p)	63.45	67.59	68.02	44.39	12.1
PBEPBE/6-311+G(2d,p)	63.96	67.61	68.02	44.38	24.1
TPSSTPSS/6-31G	60.43	67.75	67.9	44.35	2.3
TPSSTPSS/6-31G(d)	59.76	67.59	68.03	44.38	2.7
TPSSTPSS/6-311G(d)	62.21	67.6	68.01	44.39	5.2
TPSSTPSS/6-311G(d,p)	62.8	67.58	68.03	44.39	7.3
TPSSTPSS/6-311+G(d,p)	63.47	67.59	68.02	44.39	19.4
TPSSTPSS/6-311+G(2d,p)	64	67.62	68	44.39	28.2
WB97XD/6-31G	65.04	67.66	67.93	44.41	2.0
WB97XD/6-31G(d)	64.13	67.52	68.05	44.42	4.6
WB97XD/6-311G(d)	66.54	67.53	68.03	44.43	6.5
WB97XD/6-311G(d,p)	66.95	67.54	68.03	44.43	9.3
WB97XD/6-311+G(d,p)	67.6	67.54	68.03	44.44	31.5
WB97XD/6-311+G(2d,p)	68.54	67.56	68.01	44.43	223.8
PBE0/6-31G	63.82	67.67	67.94	44.39	20.5
PBE0/6-31G(d)	62.21	67.54	68.05	44.41	57.8
PBE0/6-311G(d)	63.93	67.52	68.06	44.42	156.5
PBE0/6-311G(d,p)	64.3	67.53	68.05	44.42	210.2
6-31G MN15	65.22	67.65	67.95	44.4	2.0
6-31G(d) MN15	64.45	67.51	68.07	44.42	3.1
6-311G(d) MN15	69.37	67.52	68.05	44.43	7.0
6-311G(d,p) MN15	70.48	67.53	68.04	44.43	5.7
6-311+G(d,p) MN15	71.16	67.51	68.06	44.43	23.6
6-311+G(2d,p) MN15	73.02	67.54	68.04	44.42	42.1
6-311++G(2d,p) MN15	60.8	67.49	68.06	44.45	44.0
6-311++G(d,p) MN15	60.96	67.48	68.07	44.45	28.5
cc-pVDZ MN15	61.16	67.47	68.08	44.45	8.5
**cc-pVTZ MN15**	**58.92**	**67.49**	**68.06**	**44.45**	68.9
cc-pVTZ MN15/ PCM = $CHCl_3$	61.20	67.47	68.08	44.45	99.1
cc-pVQZ MN15	58.99	67.48	68.06	44.45	1078.8
LanL2DZ MN15	59.25	67.61	67.98	44.42	4.9
LanL2MB MN15	51.77	67.79	67.88	44.33	0.9
DGDZVP2 MN15	58.99	67.55	68.02	44.43	15.4

Red color indicates that the deviation of the dihedral angle is less than 5%

- Molecular orbitals of **3a** and **3b**

Table S10. Selected molecular orbitals of **3a** optimized in the ground state S_0 calculated at MN15/cc-PVTZ level of theory (isosurface value = 0.02 a.u.).

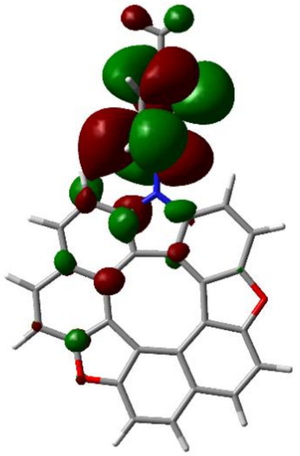<br>LUMO+3 (−0.041 eV)	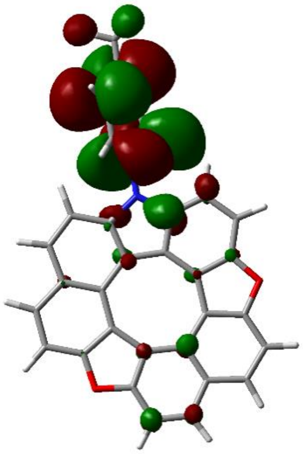<br>LUMO+2 (−0.138 eV)	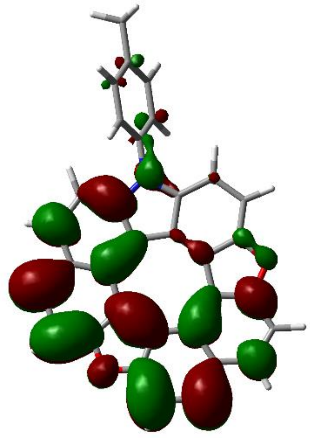<br>LUMO+1 (−0.753 eV)
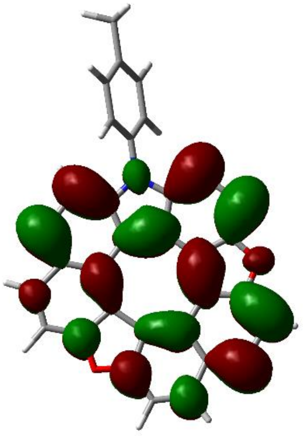<br>LUMO (−1.004 eV)	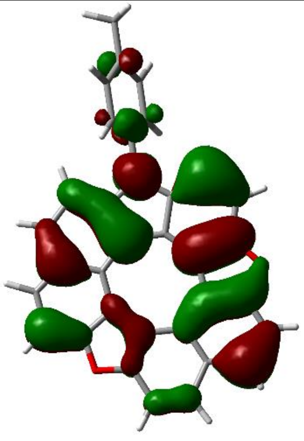<br>HOMO (−6.224 eV)	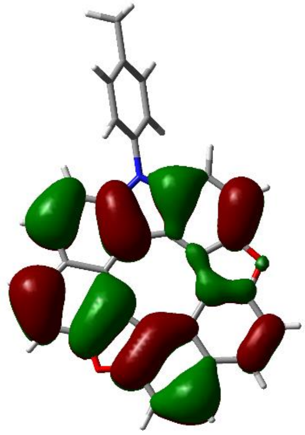<br>HOMO−1 (−6.240 eV)
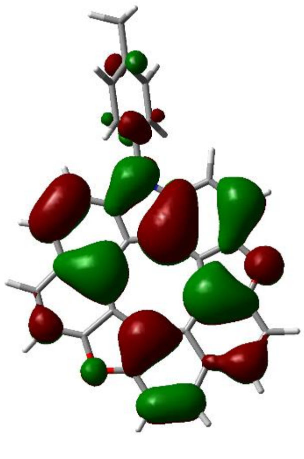<br>HOMO−2 (−6.476 eV)	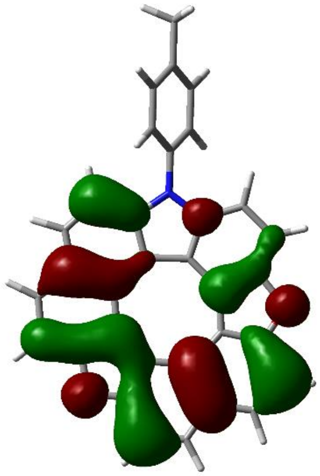<br>HOMO−3 (−6.919 eV)	

Table S11. Selected molecular orbitals of **3b** optimized in the ground state S_0 calculated at MN15/cc-PVTZ level of theory (isosurface value = 0.02 a.u.).

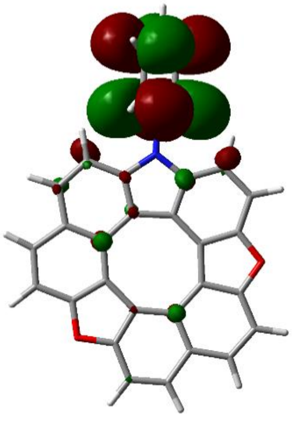<br>LUMO+3 (−0.107 eV)	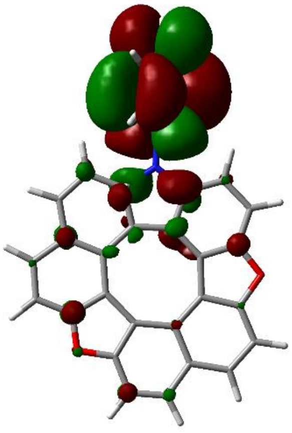<br>LUMO+2 (−0.192 eV)	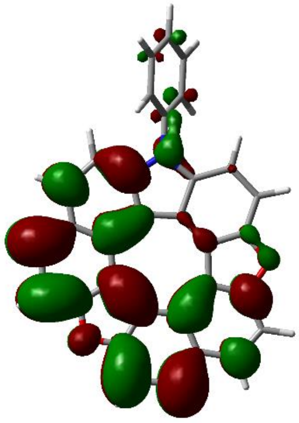<br>LUMO+1 (−0.780 eV)
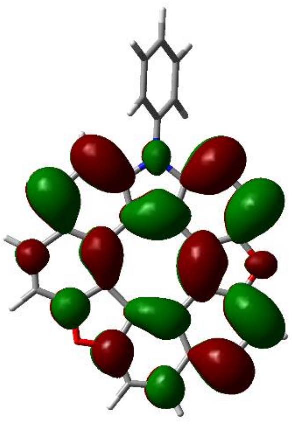<br>LUMO (−1.033 eV)	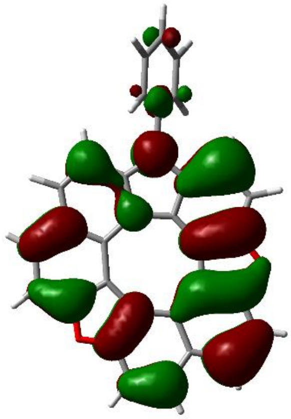<br>HOMO (−6.262 eV)	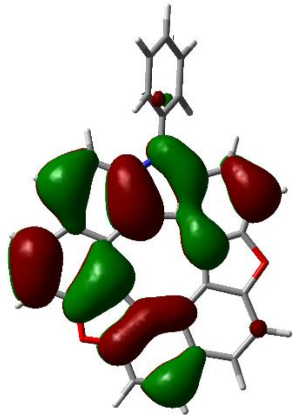<br>HOMO−1 (−6.271 eV)
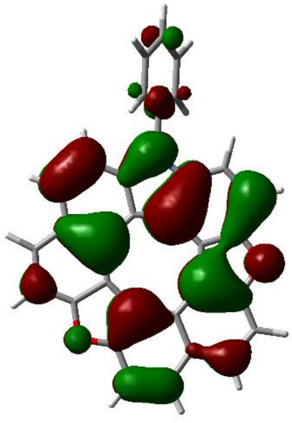<br>HOMO−2 (−6.515 eV)	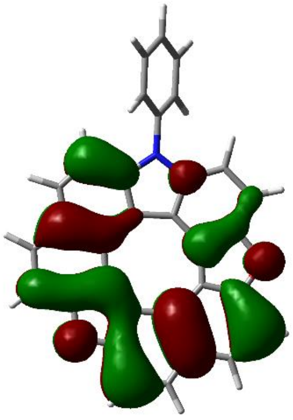<br>HOMO−3 (−6.945 eV)	

Table S12. Selected molecular orbitals of **3a** optimized in the ground state S_0 calculated at MN15/cc-PVTZ/ PCM = chloroform level of theory (isosurface value = 0.02 a.u.).

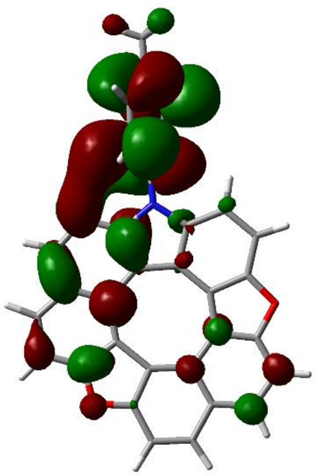	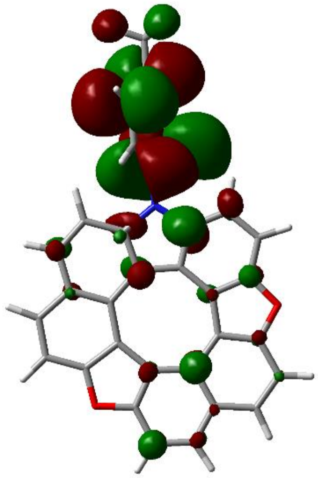	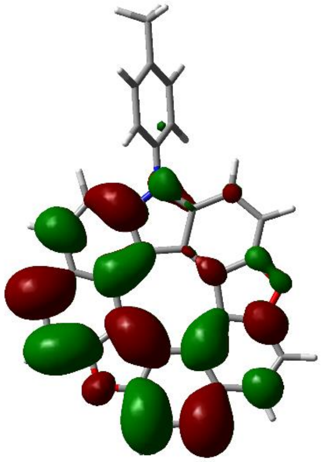
LUMO+3 (0.150 eV)	LUMO+2 (0.070 eV)	LUMO+1 (−0.788 eV)
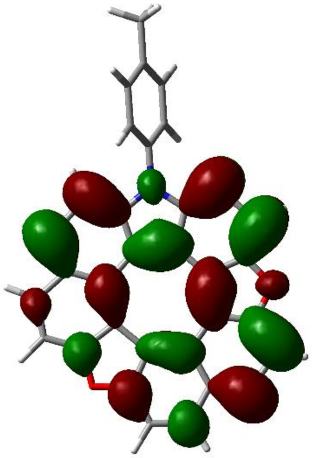	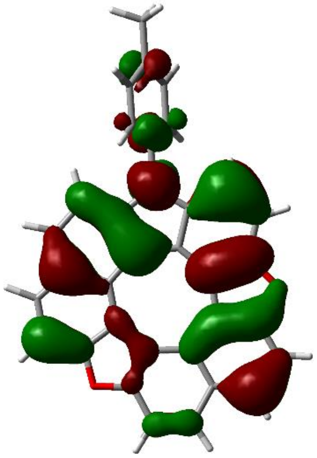	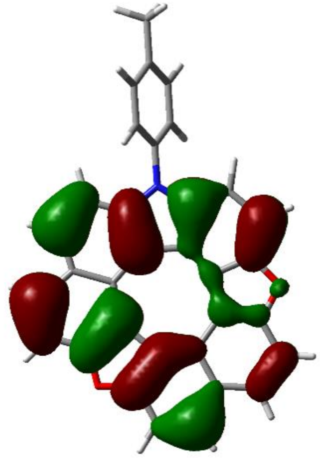
LUMO (−1.035 eV)	HOMO (−6.251 eV)	HOMO−1 (−6.278 eV)
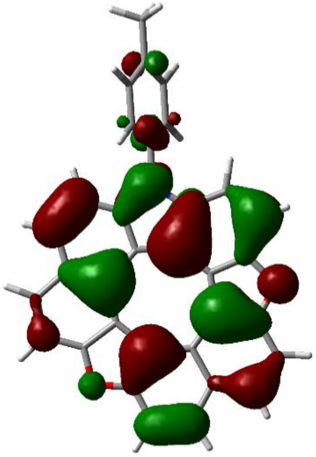	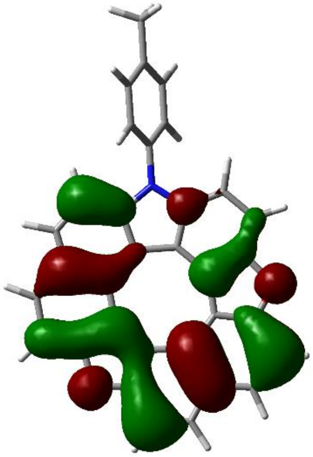	
HOMO−2 (−6.524 eV)	HOMO−3 (−6.979 eV)	

Table S13. Selected molecular orbitals of **3b** optimized in the ground state S_0 calculated at MN15/cc-PVTZ/ PCM = chloroform level of theory (isosurface value = 0.02 a.u.).

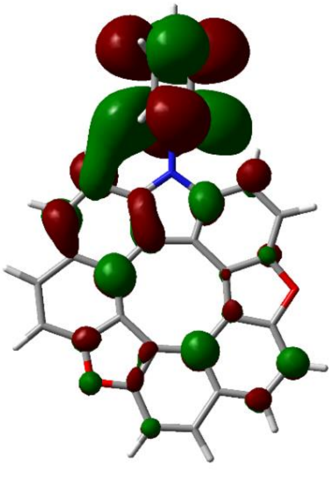 LUMO+3 (0.105 eV)	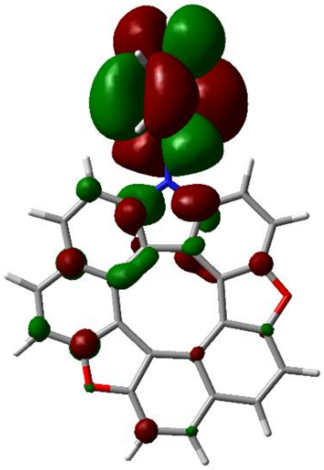 LUMO+2 (0.082 eV)	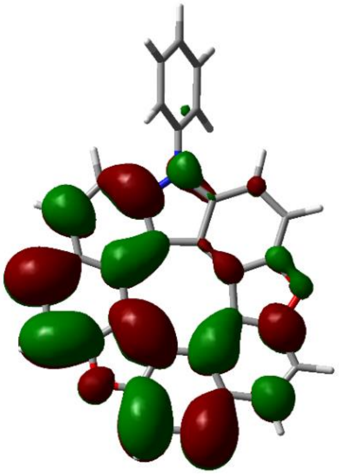 LUMO+1 (−0.797 eV)
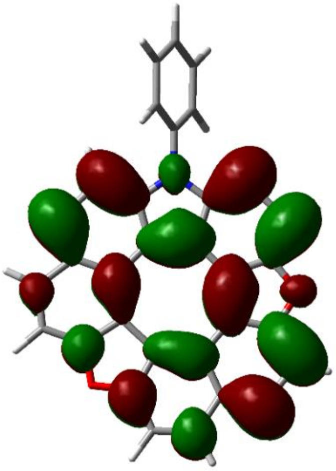 LUMO (−1.047 eV)	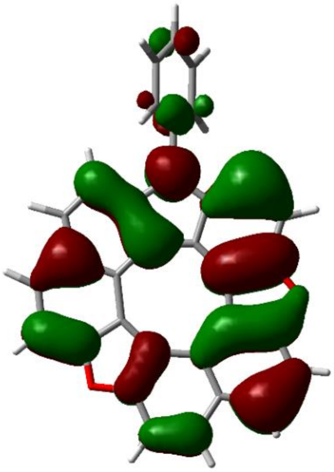 HOMO (−6.275 eV)	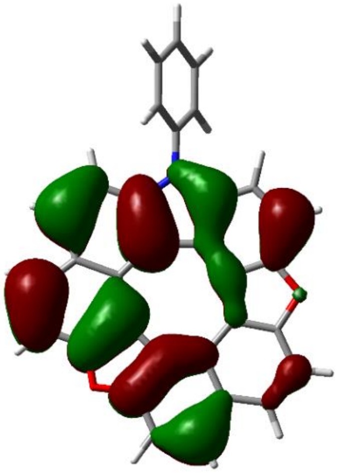 HOMO−1 (−6.291 eV)
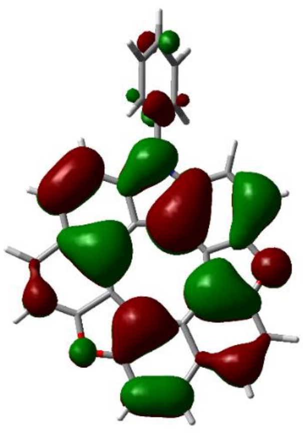 HOMO−2 (−6.545 eV)	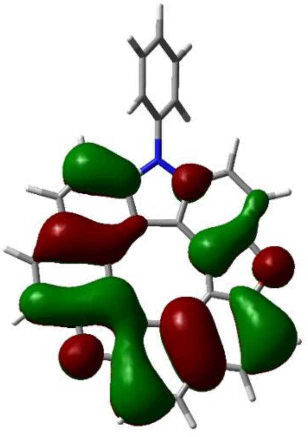 HOMO−3 (−6.988 eV)	

Table S14. Selected molecular orbitals of **3a** optimized in the excited state S_1 calculated at MN15/cc-PVTZ level of theory (isosurface value = 0.02 a.u.).

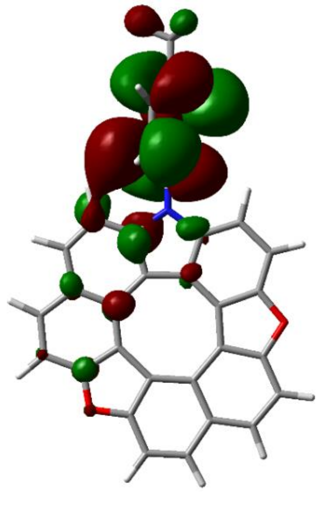 LUMO+3 (−0.039 eV)	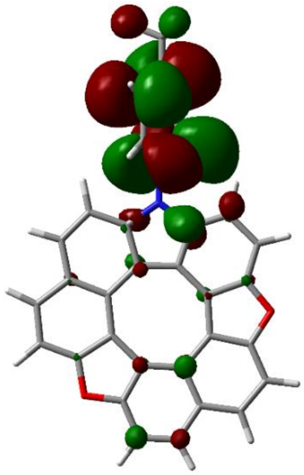 LUMO+2 (−0.132 eV)	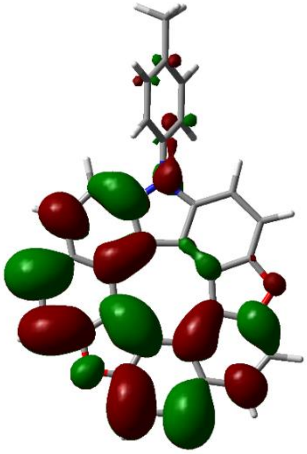 LUMO+1 (−0.779 eV)
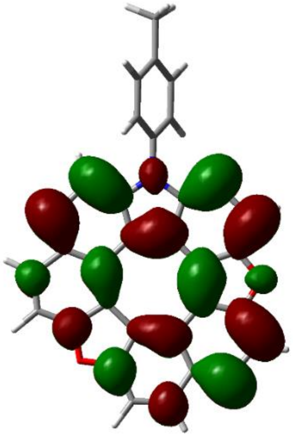 LUMO (−1.309 eV)	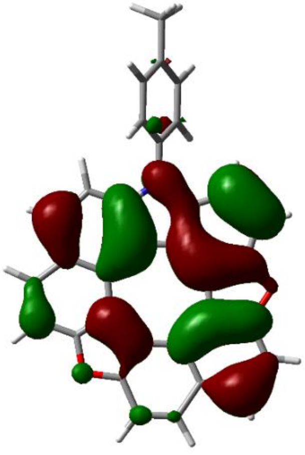 HOMO (−5.954 eV)	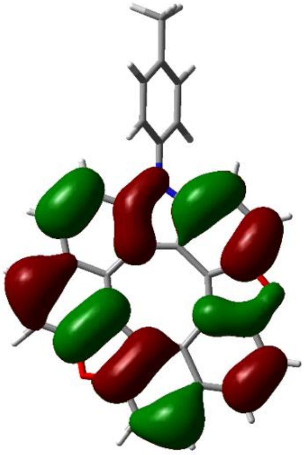 HOMO−1 (−6.209 eV)
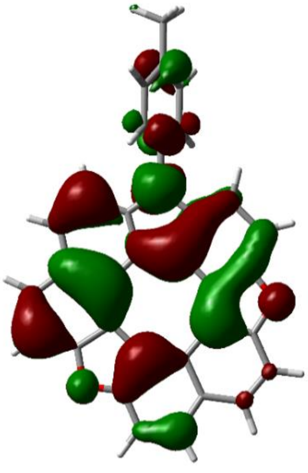 HOMO−2 (−6.490 eV)	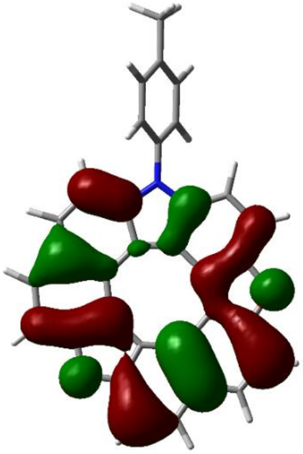 HOMO−3 (−6.939 eV)	

Table S15. Selected molecular orbitals of **3b** optimized in the excited state S_1 calculated at MN15/cc-PVTZ level of theory (isosurface value = 0.02 a.u.).

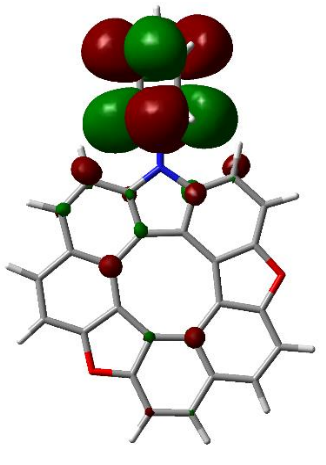 LUMO+3 (−0.106 eV)	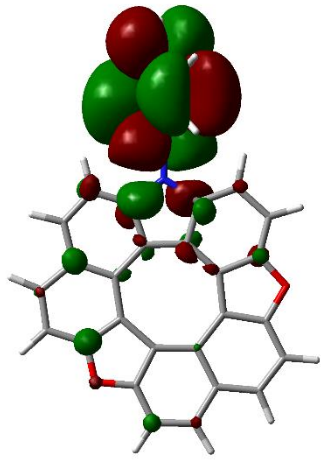 LUMO+2 (−0.185 eV)	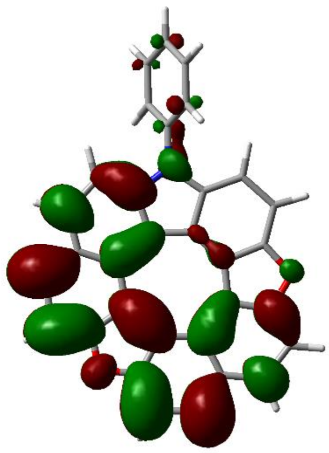 LUMO+1 (−0.805 eV)
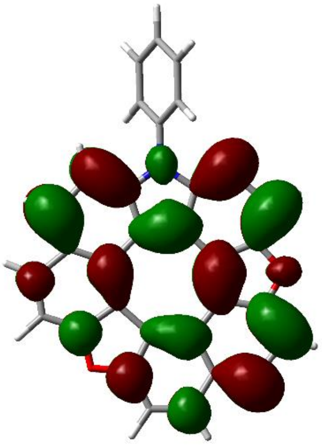 LUMO (−1.340 eV)	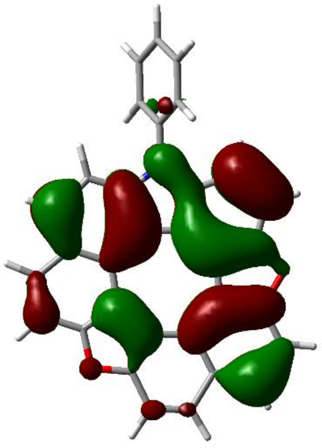 HOMO (−5.987 eV)	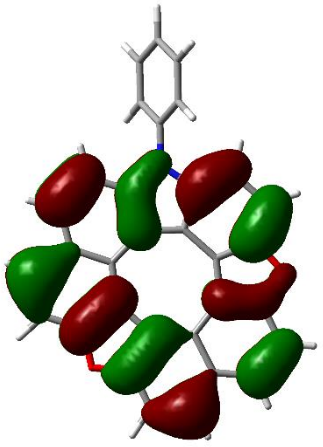 HOMO−1 (−6.237 eV)
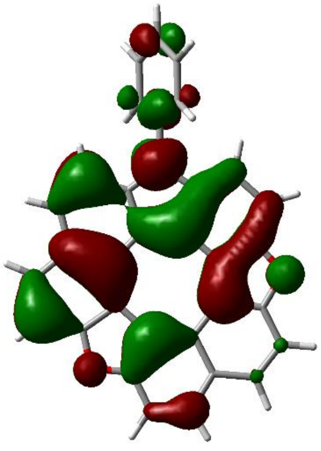 HOMO−2 (−6.535 eV)	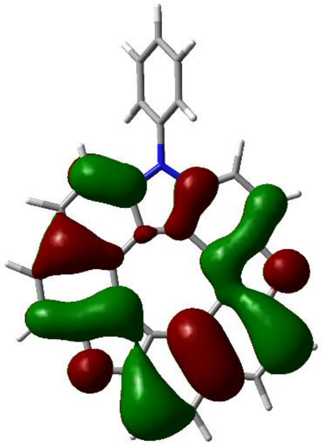 HOMO−3 (−6.964 eV)	

Table S16. Selected molecular orbitals of **3a** optimized in the excited state S_1 calculated at MN15/cc-PVTZ/ PCM = chloroform level of theory (isosurface value = 0.02 a.u.).

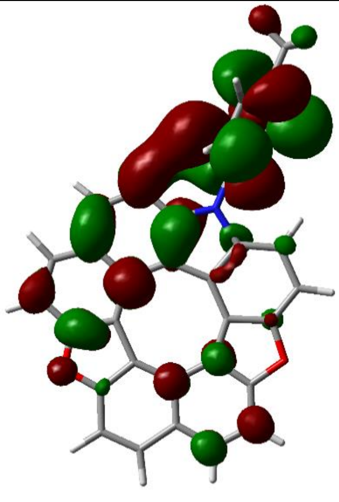<br>LUMO+3 (0. 148eV)	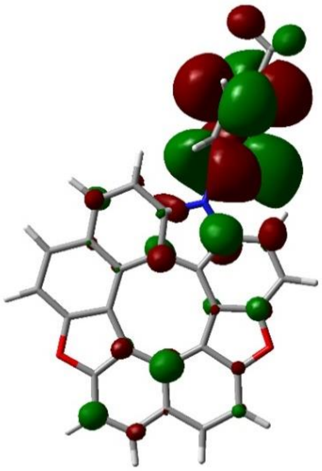<br>LUMO+2 (0. 067eV)	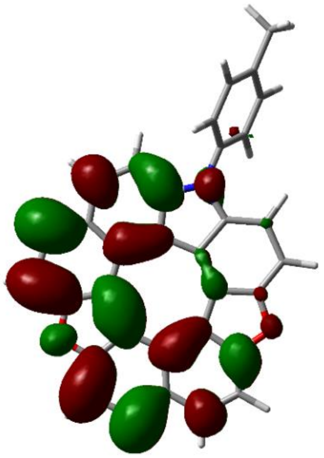<br>LUMO+1 (−0.7 80eV)
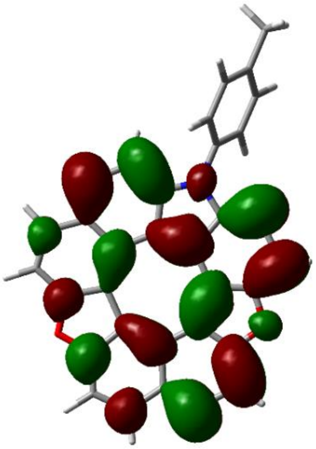<br>LUMO (−1. 351eV)	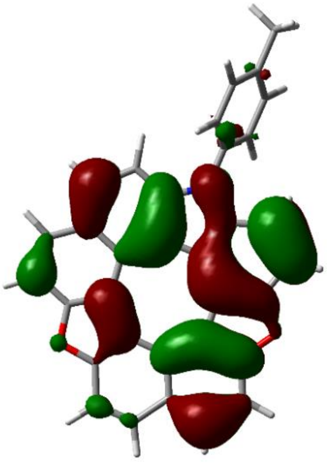<br>HOMO (−5. 989eV)	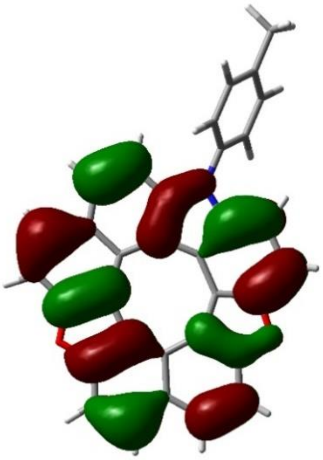<br>HOMO−1 (−6.2 66eV)
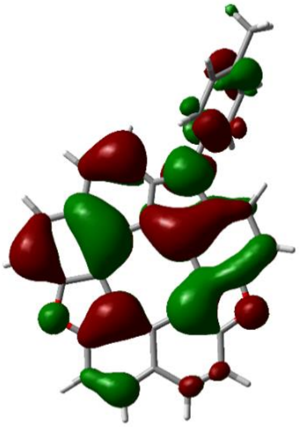<br>HOMO−2 (−6. 536eV)	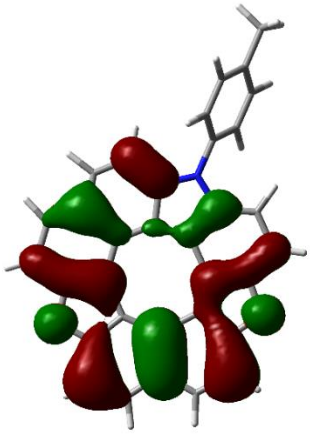<br>HOMO−3 (−7. 005eV)	

Table S17. Selected molecular orbitals of **3b** optimized in the excited state S_1 calculated at MN15/cc-PVTZ/ PCM = chloroform level of theory (isosurface value = 0.02 a.u.).

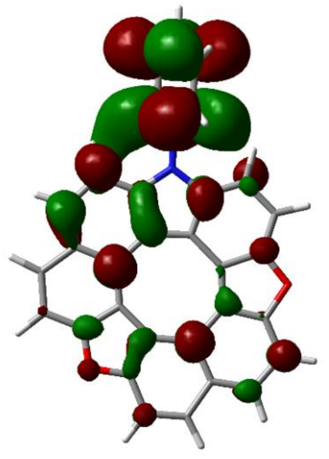 LUMO+3 (0.099 eV)	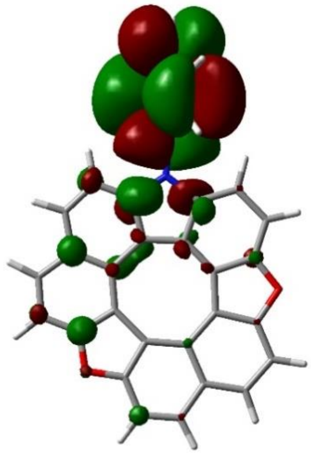 LUMO+2 (0.013 eV)	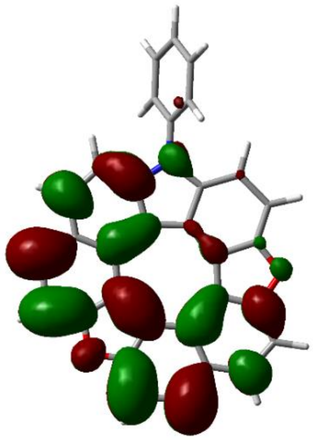 LUMO+1 (−0.809 eV)
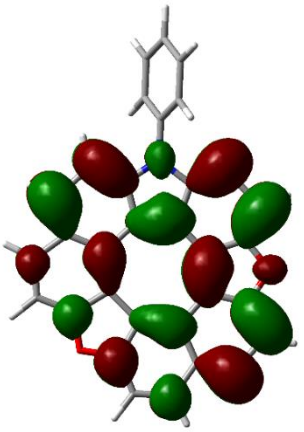 LUMO (−1.365 eV)	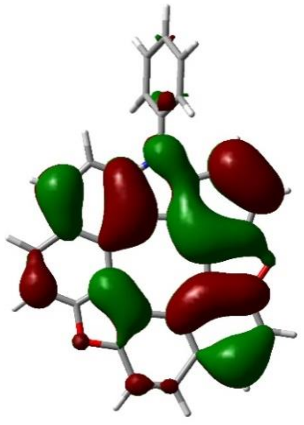 HOMO (−6.004 eV)	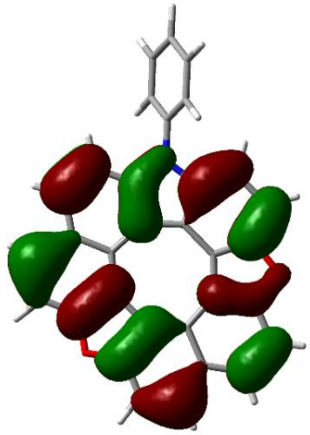 HOMO−1 (−6.277 eV)
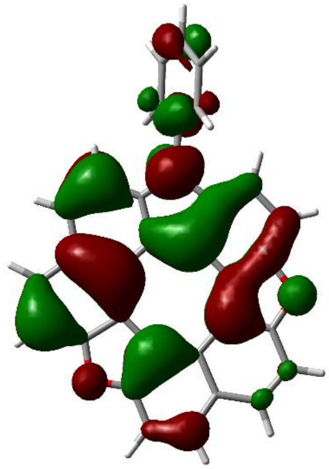 HOMO−2 (−6.567 eV)	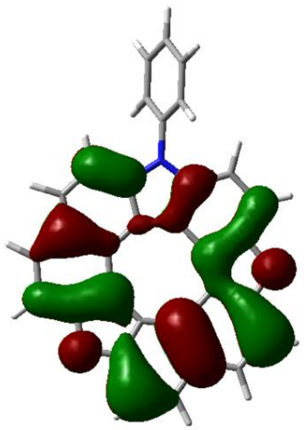 HOMO−3 (−7.015 eV)	

- Aromaticity of dioxaza[8]circulenes **3a** and **3b**
  - Aromaticity of dioxaza[8]circulene **3a**

calculated at MN15/cc-PVTZ level of theory

- *NICS(r)iso and NICS(r)zz of dioxaza[8]circulene* ***3a***

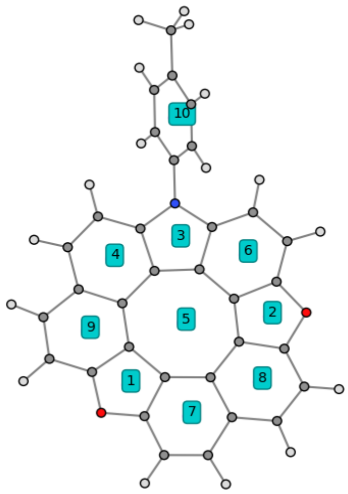

Ring	NICS(0)iso	NICS(0)zz	NICS(1)iso	NICS(1)zz	NICS(−1)iso	NICS(−1)zz	NICS(2)iso	NICS(2)zz	NICS(−2)iso	NICS(−2)zz
1	−4.2097	15.0752	−5.1693	−10.9581	−5.1575	−11.2075	−3.4746	−12.0934	−3.4794	−12.4177
2	−6.3414	8.4579	−6.7688	−16.2227	−6.8349	−16.0228	−3.9213	−13.8739	−3.9258	−13.4699
3	−8.2252	2.5563	−8.1592	−21.3414	−8.094	−21.3123	−4.277	−15.3429	−4.2331	−15.2624
4	−6.889	−6.1115	−8.7571	−22.6336	−9.0154	−23.1964	−5.0603	−16.7772	−5.321	−17.3067
5	7.3303	33.2544	3.7603	15.5929	3.7469	15.5877	−0.5963	−2.9824	−0.6075	−2.9921
6	−10.7024	−14.7373	−11.092	−29.3513	−10.881	−28.6711	−5.7482	−18.7906	−5.5053	−18.0743
7	−8.8519	−12.1799	−10.4131	−27.5175	−10.3222	−27.6218	−5.6292	−18.1832	−5.6015	−18.414
8	−8.2615	−10.1468	−9.7062	−25.5191	−9.7351	−25.3296	−5.465	−17.8655	−5.4645	−17.6417
9	−9.0342	−12.4488	−10.3941	−27.5317	−10.4896	−27.9071	−5.5698	−18.0726	−5.6457	−18.4004
10	−6.4764	−1.236	−8.5576	−1.9096	−8.5138	−1.9703	−3.8543	0.9495	−3.8576	0.8748

- *NICS-XY-Scan of dioxaza[8]circulene* ***3a***

Path of NICS-XY-Scan	NICS(iso)-XY-Scan	NICS(zz)-XY-Scan
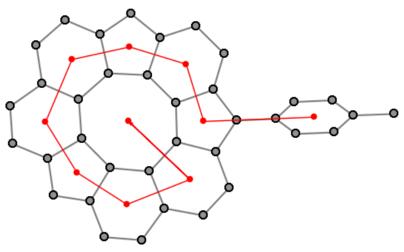	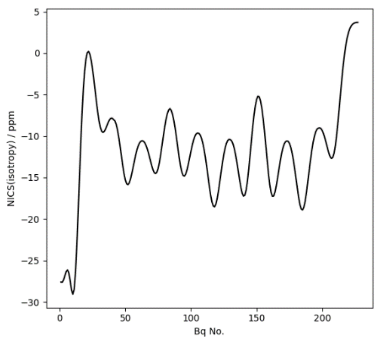	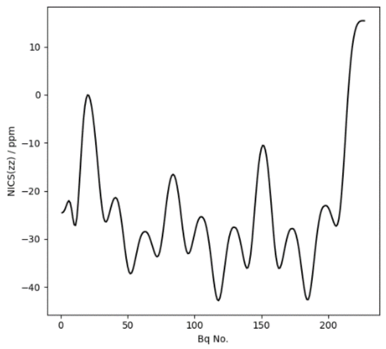

- *Integral NICS of dioxaza[8]circulene* ***3a***

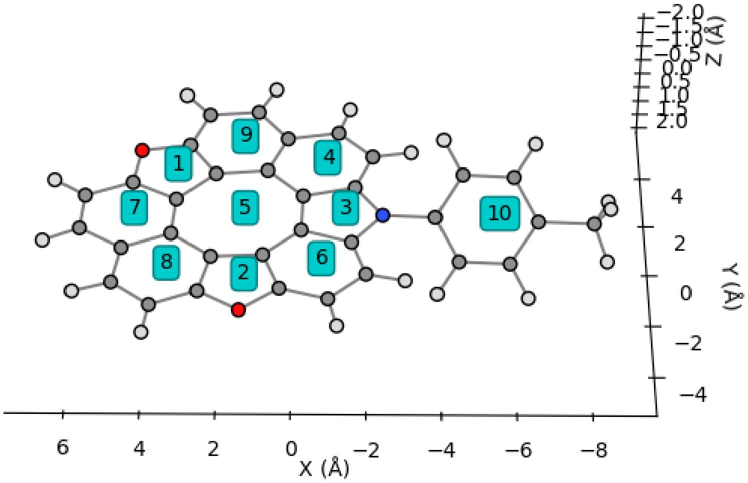

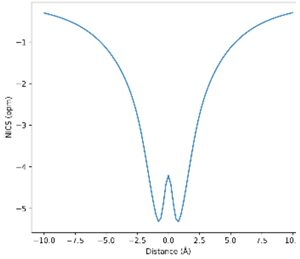

NICS(iso) plot of **ring 1**

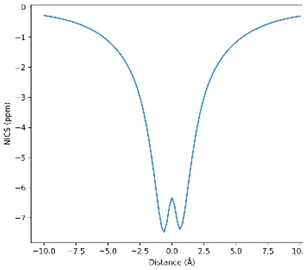

NICS(iso) plot of **ring 2**

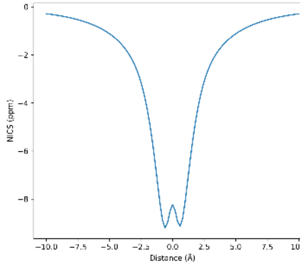

NICS(iso) plot of **ring 3**

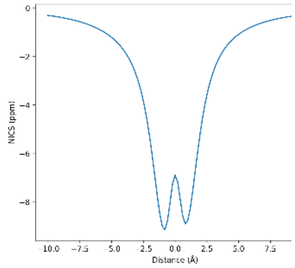

NICS(iso) plot of **ring 4**

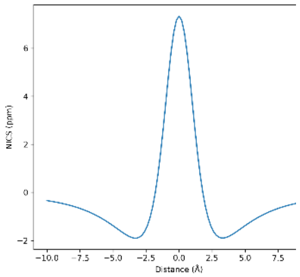

NICS(iso) plot of **ring 5**

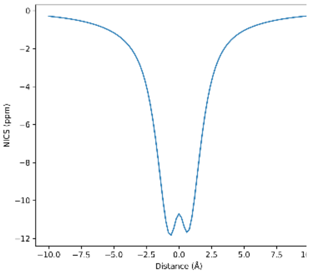

NICS(iso) plot of **ring 6**

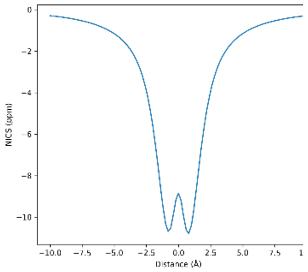

NICS(iso) plot of **ring 7**

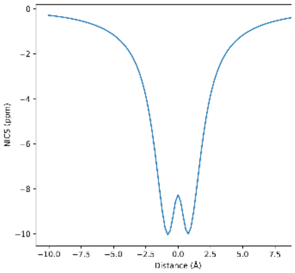

NICS(iso) plot of **ring 8**

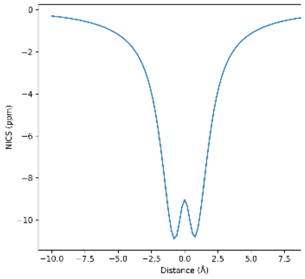

NICS(iso) plot of **ring 9**

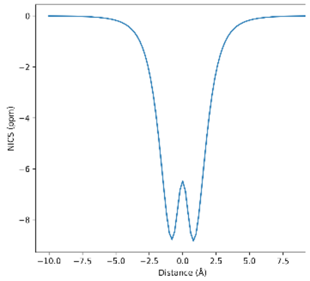

NICS(iso) plot of **ring 10**

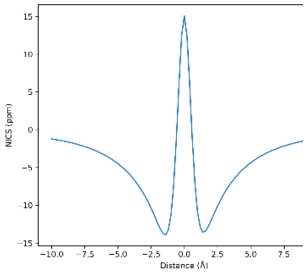

NICS(zz) plot of **ring 1**

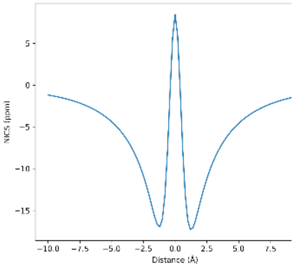

NICS(zz) plot of **ring 2**

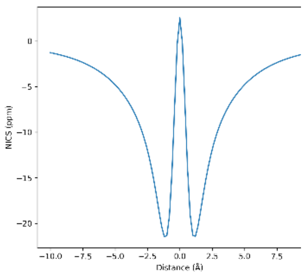

NICS(zz) plot of **ring 3**

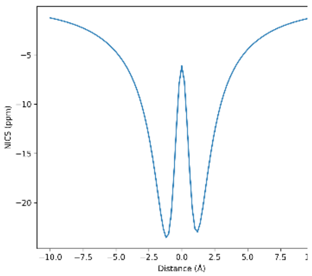

NICS(zz) plot of **ring 4**

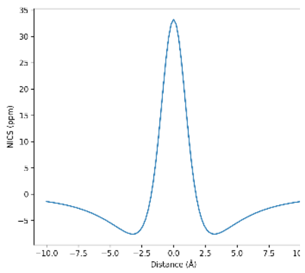

NICS(zz) plot of **ring 5**

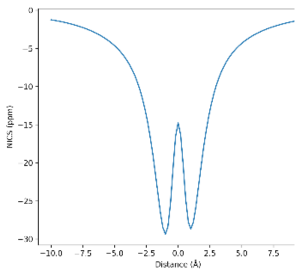

NICS(zz) plot of **ring 6**

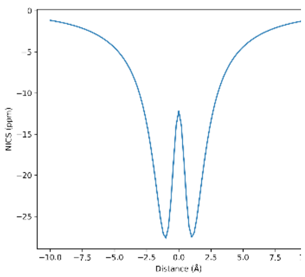

NICS(zz) plot of **ring 7**

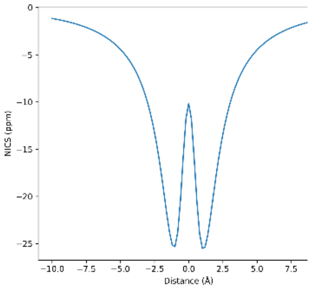

NICS(zz) plot of **ring 8**

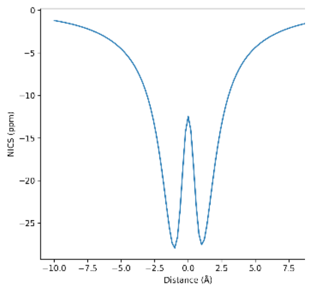

NICS(zz) plot of **ring 9**

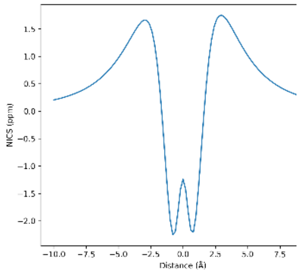

NICS(zz) plot of **ring 10**

- *2D NICS/ICSS of dioxaza[8]circulene **3a***

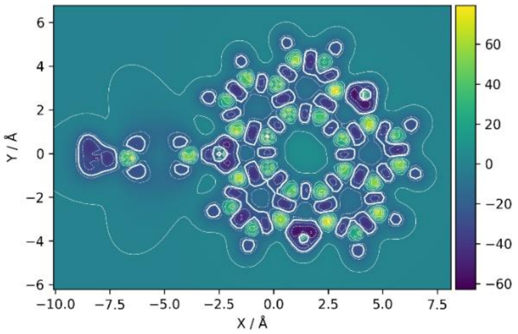

2D heatmap on the XY plane
Component of shielding tensors: isotropy

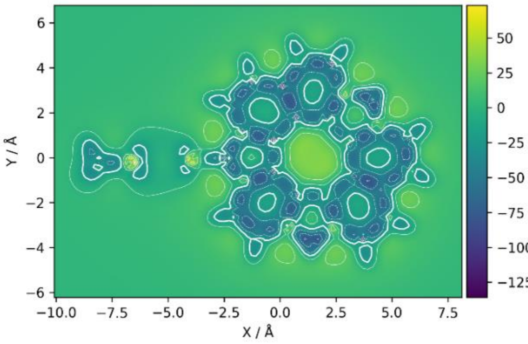

2D heatmap on the XY plane
Component of shielding tensors: zz

- *HOMA & HOMER of dioxaza[8]circulene **3a***

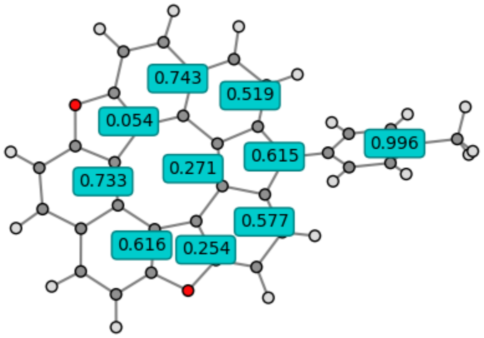

HOMA

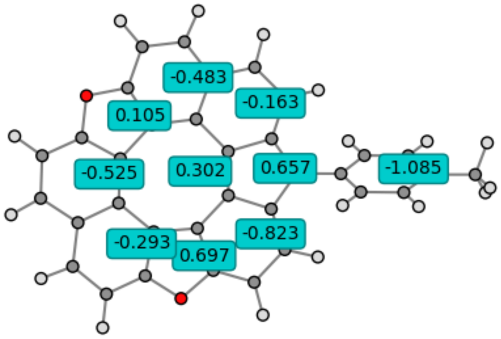

HOMER

Aromaticity of dioxaza[8]circulene **3b**

calculated at MN15/cc-PVTZ level of theory

- *NICS(r)iso and NICS(r)zz of dioxaza[8]circulene* ***3b***

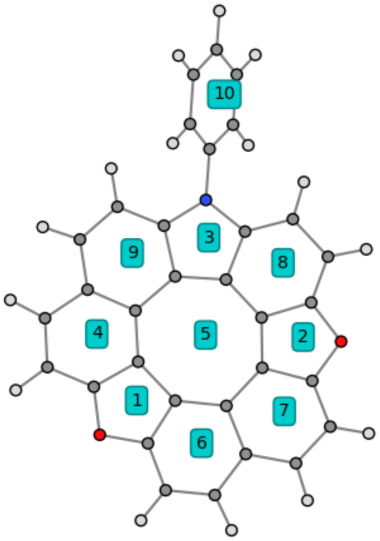

Ring	NICS(0)iso	NICS(0)zz	NICS(1)iso	NICS(1)zz	NICS(−1)iso	NICS(−1)zz	NICS(2)iso	NICS(2)zz	NICS(−2)iso	NICS(−2)zz
**1**	−4.1864	15.1234	−5.1412	−11.1729	−5.152	−10.9296	−3.4715	−12.4063	−3.4663	−12.094
**2**	−6.3448	8.4014	−6.8361	−16.0731	−6.7758	−16.2815	−3.9149	−13.4796	−3.9166	−13.8926
**3**	−8.159	2.6574	−8.0408	−21.2363	−8.0944	−21.2486	−4.1998	−15.2526	−4.2403	−15.3177
**4**	−9.0261	−12.463	−10.4877	−27.9207	−10.391	−27.5447	−5.6429	−18.4132	−5.5649	−18.0805
**5**	7.3469	33.2658	3.7632	15.5971	3.7755	15.6021	−0.5935	−2.9881	−0.5836	−2.9792
**6**	−8.8513	−12.2042	−10.3189	−27.6299	−10.4096	−27.5419	−5.595	−18.4059	−5.6231	−18.1948
**7**	−8.2279	−10.0759	−9.7182	−25.3004	−9.6944	−25.5082	−5.4537	−17.626	−5.457	−17.8635
**8**	−10.6704	−14.7094	−10.8403	−28.631	−11.0621	−29.3294	−5.4756	−18.0621	−5.7274	−18.7839
**9**	−6.8937	−6.1765	−9.0146	−23.2553	−8.7413	−22.6434	−5.3164	−17.3461	−5.0424	−16.7762
**10**	−6.5775	−1.5068	−8.7934	−2.6648	−8.774	−2.6058	−4.0022	0.3674	−3.9784	0.4852

- *NICS-XY-Scan of dioxaza[8]circulene* ***3b***

Path of NICS-XY-Scan	NICS(iso)-XY-Scan	NICS(zz)-XY-Scan
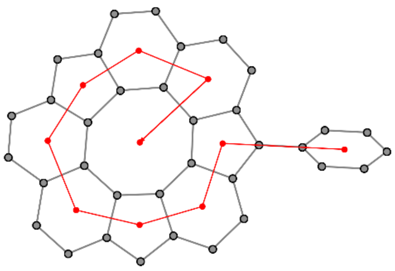	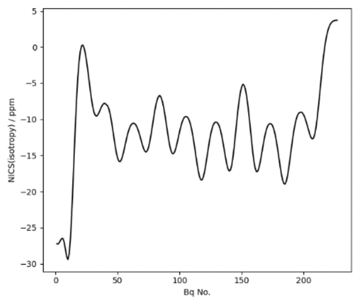	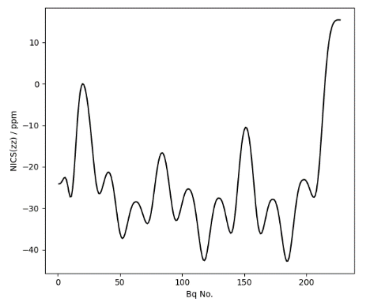

- *Integral NICS of dioxaza[8]circulene **3b***

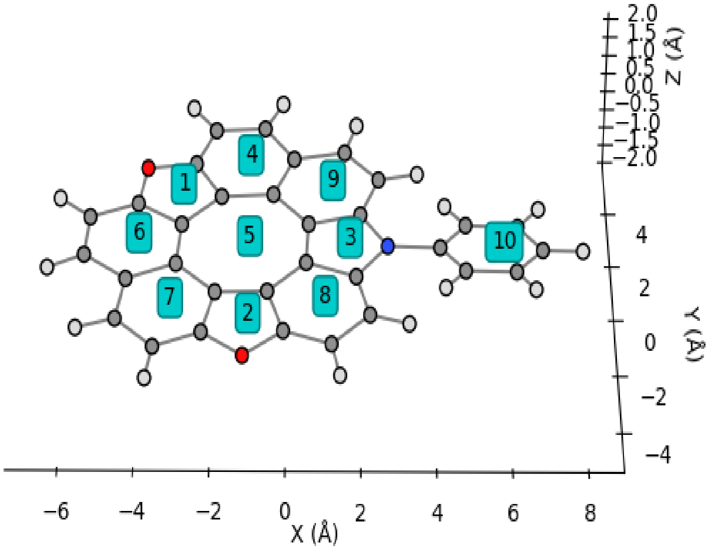

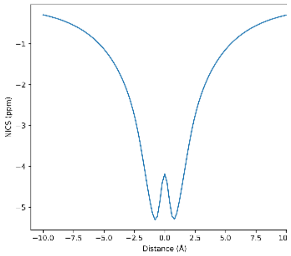

NICS(iso) plot of **ring 1**

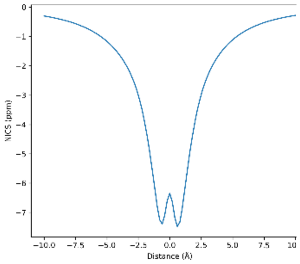

NICS(iso) plot of **ring 2**

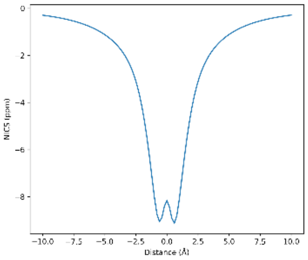

NICS(iso) plot of **ring 3**

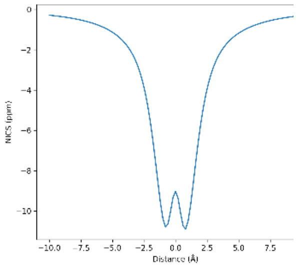

NICS(iso) plot of **ring 4**

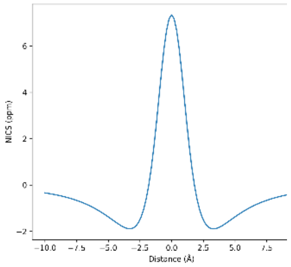

NICS(iso) plot of **ring 5**

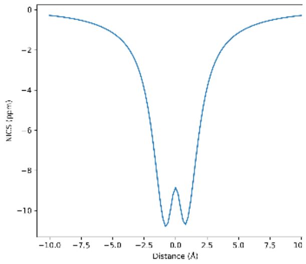

NICS(iso) plot of **ring 6**

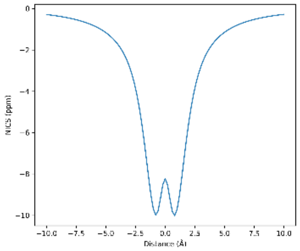

NICS(iso) plot of **ring 7**

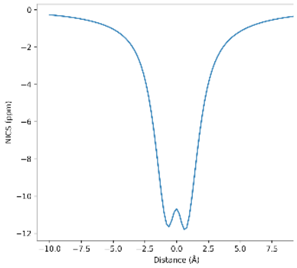

NICS(iso) plot of **ring 8**

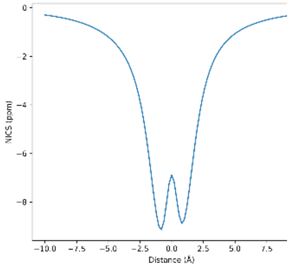

NICS(iso) plot of **ring 9**

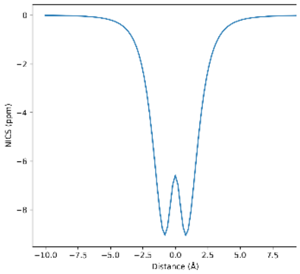

NICS(iso) plot of **ring 10**

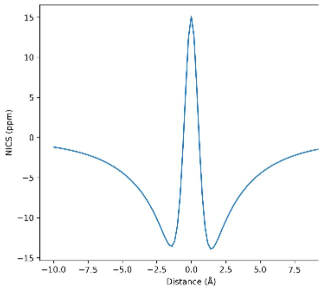

NICS(zz) plot of **ring 1**

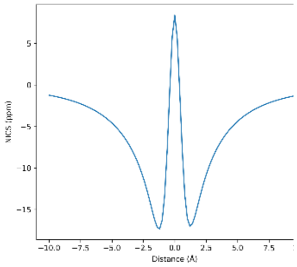

NICS(zz) plot of **ring 2**

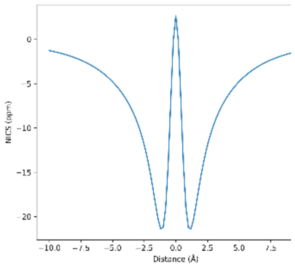

NICS(zz) plot of **ring 3**

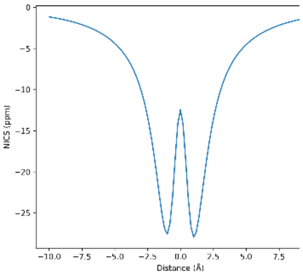

NICS(zz) plot of **ring 4**

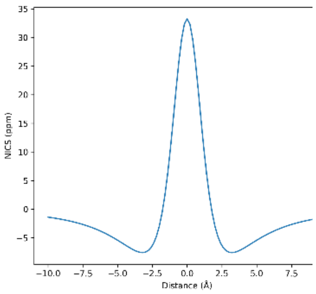

NICS(zz) plot of **ring 5**

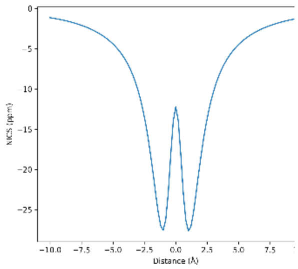

NICS(zz) plot of **ring 6**

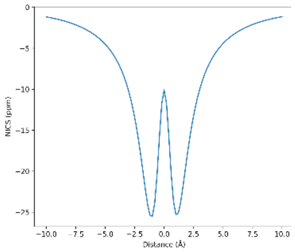

NICS(zz) plot of **ring 7**

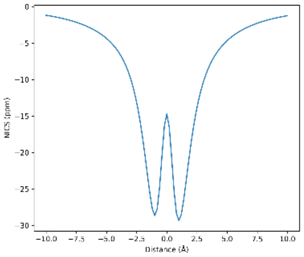

NICS(zz) plot of **ring 8**

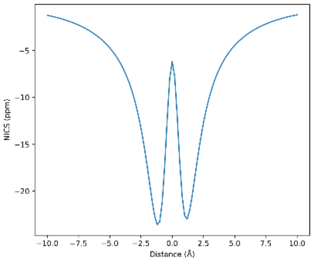

NICS(zz) plot of **ring 9**

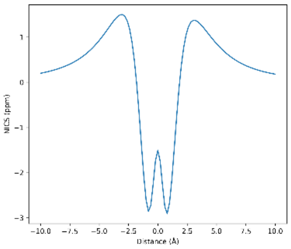

NICS(zz) plot of **ring 10**

- *2D NICS/ICSS of dioxaza[8]circulene* ***3b***

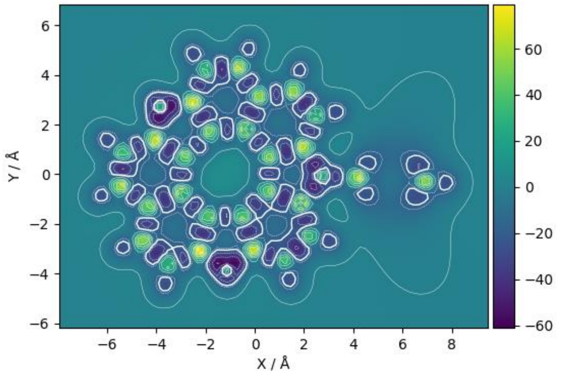

2D heatmap on the XY plane
Component of shielding tensors: isotropy

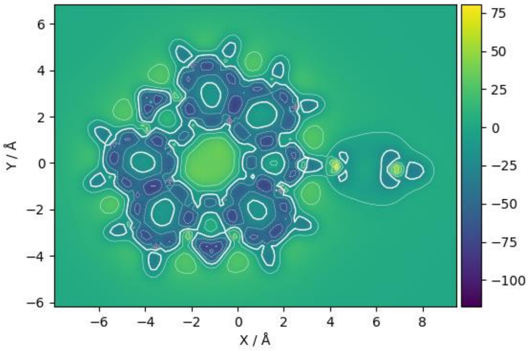

2D heatmap on the XY plane
Component of shielding tensors: zz

- *HOMA & HOMER of dioxaza[8]circulene* ***3b***

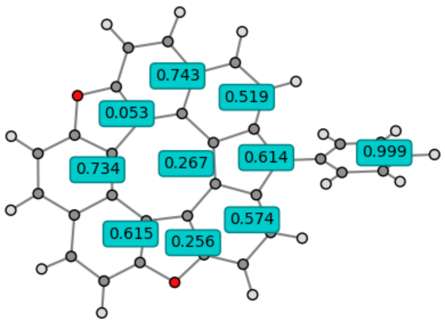

HOMA

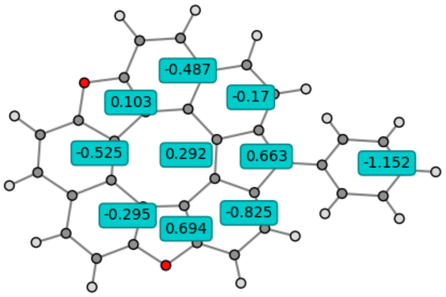

HOMER

- ACID plots of **3a** and **3b**

calculated at the B3LYP/6-311G(d, p) level of theory (isosurface value: 0.05)

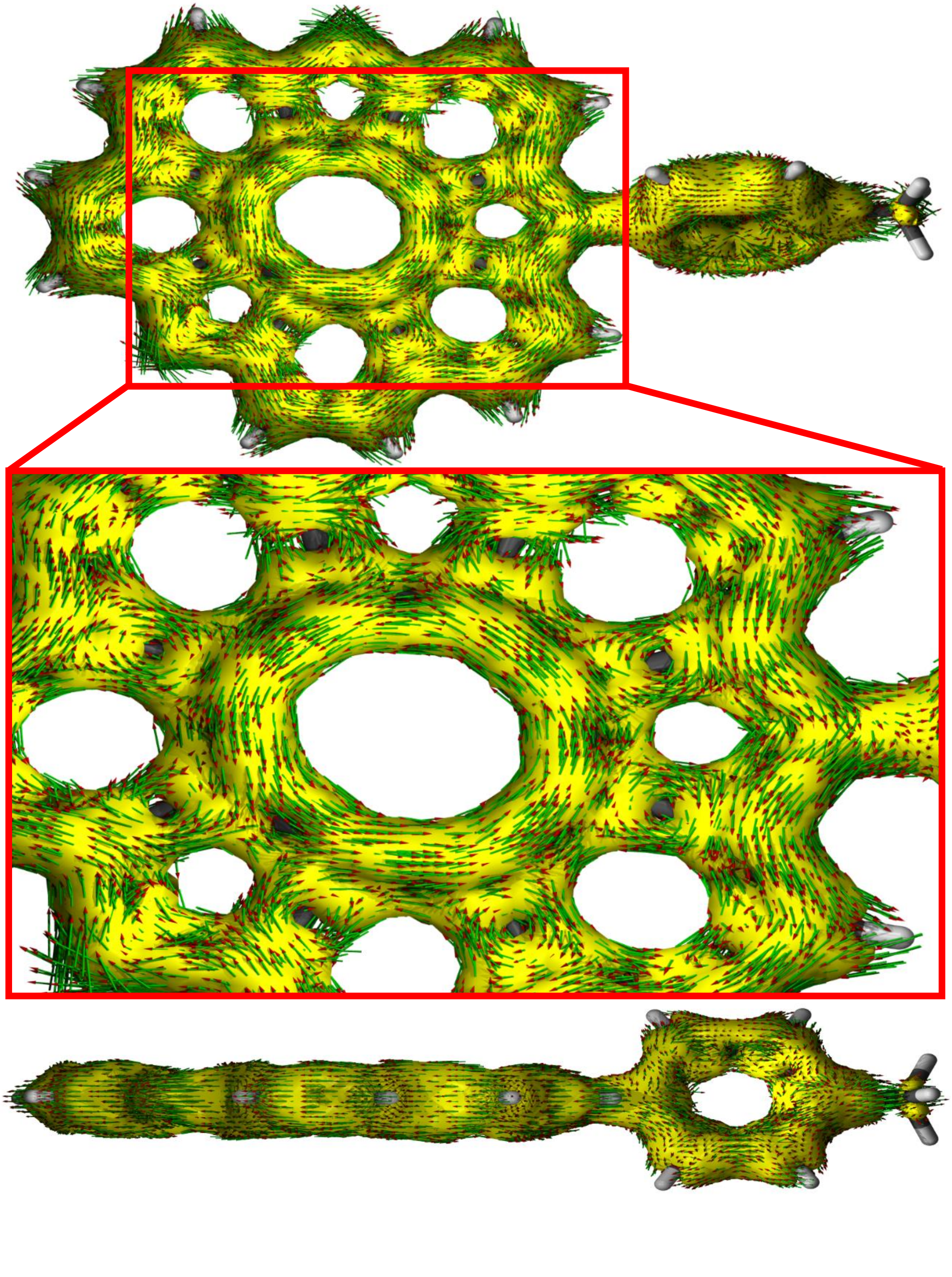

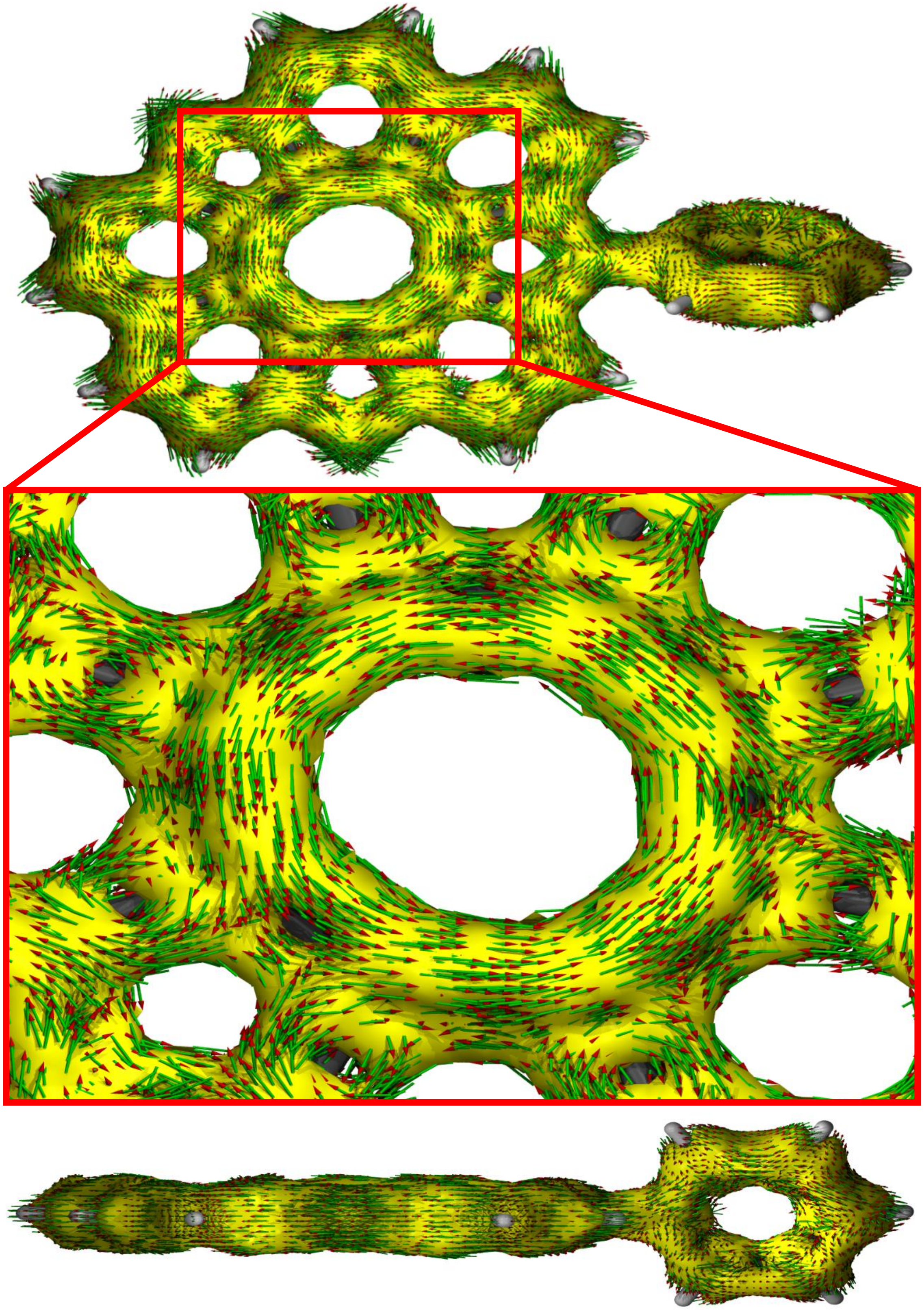

- Time-dependent density-functional theory (TD-DFT) calculations
  - Summary of the TD-DFT calculation results

Table S18. Summary of the TD-DFT calculation results of **3a** (S_0 state geometry) at MN15/cc-PVTZ level of theory.

Excited states	Energy (eV)	Wavelength (nm)	Oscillator strength	Major contributions (%)		
S_1	3.5422	350.02	0.0736	HOMO−1	LUMO	13
				HOMO−1	LUMO+1	3
				HOMO	LUMO	69
				HOMO	LUMO+1	6
S_2	3.765	329.31	0.0139	HOMO−3	LUMO+1	5
				HOMO−2	LUMO	34
				HOMO−2	LUMO+1	8
				HOMO−1	LUMO	28
				HOMO	LUMO	14
				HOMO	LUMO+1	7
S_3	3.7711	328.77	0.1033	HOMO−2	LUMO	45
				HOMO−2	LUMO+1	2
				HOMO−1	LUMO	28
				HOMO	LUMO	5
				HOMO	LUMO+1	11
S_4	3.9723	312.12	0.3572	HOMO−3	LUMO	6
				HOMO−2	LUMO+1	10
				HOMO−1	LUMO	14
				HOMO−1	LUMO+1	21
				HOMO	LUMO+1	40
S_5	4.0516	306.01	0.0486	HOMO−3	LUMO	24
				HOMO−3	LUMO+1	7
				HOMO−1	LUMO	6
				HOMO−1	LUMO+1	35
				HOMO	LUMO+1	17
S_6	4.2787	289.77	0.0216	HOMO−3	LUMO	6
				HOMO−2	LUMO	9
				HOMO−2	LUMO+1	62
				HOMO	LUMO+1	12
S_7	4.425	280.19	0.1745	HOMO−3	LUMO	15
				HOMO−3	LUMO+1	25
				HOMO−2	LUMO	3
				HOMO−2	LUMO+1	3
				HOMO−1	LUMO+1	21
				HOMO−1	LUMO+4	2
				HOMO−1	LUMO+5	6
				HOMO	LUMO	6
				HOMO	LUMO+4	4

S_8	4.6079	269.07	0.1055	HOMO−4	LUMO	2
				HOMO−3	LUMO	27
				HOMO−3	LUMO+1	21
				HOMO−2	LUMO+1	7
				HOMO−1	LUMO+1	12
				HOMO−1	LUMO+5	2
				HOMO	LUMO+3	10
				HOMO	LUMO+4	5
				HOMO	LUMO+5	2
S_9	4.6523	266.5	0.1254	HOMO−5	LUMO+2	3
				HOMO−3	LUMO	3
				HOMO−2	LUMO+2	20
				HOMO−1	LUMO+2	29
				HOMO	LUMO+2	34
				HOMO	LUMO+3	2
S_{10}	4.766	260.14	0.2103	HOMO−5	LUMO+3	3
				HOMO−3	LUMO	5
				HOMO−3	LUMO+1	4
				HOMO−2	LUMO+2	2
				HOMO−2	LUMO+3	19
				HOMO−1	LUMO+2	2
				HOMO−1	LUMO+3	26
				HOMO	LUMO+3	30

S_1~S_{10}

Table S19. Summary of the TD-DFT calculation results of **3a** (S_0 state geometry) at MN15/cc-PVTZ/ PCM = chloroform level of theory.

Excited states	Energy (eV)	Wavelength (nm)	Oscillator strength	Major contributions (%)		
S_1	3.5301	351.22	0.1454	HOMO−1	LUMO	5
				HOMO−1	LUMO+1	3
				HOMO	LUMO	81
				HOMO	LUMO+1	2
S_2	3.7321	332.21	0.2135	HOMO−2	LUMO	10
				HOMO−1	LUMO	66
				HOMO	LUMO	5
				HOMO	LUMO+1	14
S_3	3.7806	327.95	0.0096	HOMO−3	LUMO+1	3
				HOMO−2	LUMO	68
				HOMO−2	LUMO+1	11
				HOMO−1	LUMO	4
				HOMO	LUMO	4
				HOMO	LUMO+1	3

S_4	3.9218	316.14	0.5791	HOMO−3	LUMO	3
				HOMO−2	LUMO	3
				HOMO−2	LUMO+1	8
				HOMO−1	LUMO	11
				HOMO−1	LUMO+1	31
				HOMO	LUMO+1	36
S_5	4.0435	306.63	0.0861	HOMO−3	LUMO	22
				HOMO−3	LUMO+1	5
				HOMO−1	LUMO	5
				HOMO−1	LUMO+1	33
				HOMO	LUMO+1	26
S_6	4.2774	289.86	0.0321	HOMO−3	LUMO	6
				HOMO−2	LUMO	8
				HOMO−2	LUMO+1	66
				HOMO	LUMO+1	10
S_7	4.394	282.16	0.3431	HOMO−3	LUMO	26
				HOMO−3	LUMO+1	21
				HOMO−2	LUMO	3
				HOMO−2	LUMO+1	2
				HOMO−1	LUMO+1	19
				HOMO−1	LUMO+5	5
				HOMO	LUMO	5
				HOMO	LUMO+1	4
S_8	4.607	269.12	0.1993	HOMO−3	LUMO	27
				HOMO−3	LUMO+1	29
				HOMO−2	LUMO+1	6
				HOMO−1	LUMO+1	9
				HOMO−1	LUMO+5	2
				HOMO	LUMO+3	11
S_9	4.7947	258.59	0.2095	HOMO−2	LUMO+2	14
				HOMO−2	LUMO+3	3
				HOMO−1	LUMO+2	22
				HOMO	LUMO+2	37
				HOMO	LUMO+3	9
S_{10}	4.8044	258.06	0.3099	HOMO−4	LUMO+1	3
				HOMO−3	LUMO	7
				HOMO−3	LUMO+1	4

				HOMO−2	LUMO+3	3
				HOMO−1	LUMO+2	2
				HOMO−1	LUMO+3	12
				HOMO−1	LUMO+4	3
				HOMO	LUMO+2	11
				HOMO	LUMO+3	31

S_1~S_{10}

Table S20. Summary of the TD-DFT calculation results of **3b** (S_0 state geometry) at MN15/cc-PVTZ level of theory.

Excited states	Energy (eV)	Wavelength (nm)	Oscillator strength	Major contributions (%)		
S_1	3.5715	347.15	0.0686	HOMO−2	LUMO	2
				HOMO−1	LUMO	56
				HOMO−1	LUMO+1	6
				HOMO−1	LUMO+4	2
				HOMO	LUMO	24
				HOMO	LUMO+1	4
S_2	3.7586	329.86	0.0765	HOMO−2	LUMO	65
				HOMO−2	LUMO+1	11
				HOMO	LUMO	11
				HOMO	LUMO+1	3
				HOMO	LUMO+4	2
S_3	3.7675	329.09	0.0526	HOMO−3	LUMO+1	6
				HOMO−2	LUMO	7
				HOMO−2	LUMO+1	3
				HOMO−1	LUMO	28
				HOMO−1	LUMO+1	7
				HOMO	LUMO	44
S_4	3.9575	313.29	0.3	HOMO−3	LUMO	9
				HOMO−2	LUMO	6
				HOMO−2	LUMO+1	11
				HOMO−1	LUMO+1	37
				HOMO	LUMO	5
				HOMO	LUMO+1	24
S_5	4.0228	308.21	0.0765	HOMO−3	LUMO	22
				HOMO−3	LUMO+1	5
				HOMO−2	LUMO+1	7
				HOMO−1	LUMO+1	23
				HOMO	LUMO	5

				HOMO	LUMO+1	30
S_6	4.2505	291.69	0.0287	HOMO−3	LUMO	5
				HOMO−2	LUMO	11
				HOMO−2	LUMO+1	53
				HOMO−1	LUMO+1	20
S_7	4.4016	281.68	0.1991	HOMO−3	LUMO	21
				HOMO−3	LUMO+1	18
				HOMO−2	LUMO	2
				HOMO−2	LUMO+1	2
				HOMO−1	LUMO	9
				HOMO−1	LUMO+4	2
				HOMO	LUMO+1	23
				HOMO	LUMO+2	4
				HOMO	LUMO+5	5
S_8	4.5767	270.9	0.0282	HOMO−3	LUMO	4
				HOMO−3	LUMO+1	11
				HOMO−2	LUMO+2	12
				HOMO−1	LUMO+2	27
				HOMO−1	LUMO+3	2
				HOMO−1	LUMO+4	3
				HOMO	LUMO+2	22
				HOMO	LUMO+5	3
S_9	4.606	269.18	0.2926	HOMO−3	LUMO	20
				HOMO−3	LUMO+1	16
				HOMO−2	LUMO+1	6
				HOMO−2	LUMO+2	12
				HOMO−1	LUMO+2	2
				HOMO−1	LUMO+4	4
				HOMO	LUMO+1	8
				HOMO	LUMO+2	19
S_{10}	4.698	263.91	0.0618	HOMO−6	LUMO+2	4
				HOMO−5	LUMO+3	5
				HOMO−3	LUMO	2
				HOMO−3	LUMO+1	3
				HOMO−2	LUMO+3	24
				HOMO−1	LUMO+3	18
				HOMO	LUMO+3	41

S_1~S_{10}

Table S21. Summary of the TD-DFT calculation results of **3b** (S_0 state geometry) at MN15/cc-PVTZ/ PCM = chloroform level of theory.

Excited states	Energy (eV)	Wavelength (nm)	Oscillator strength	Major contributions (%)		
S_1	3.5611	348.17	0.148	HOMO−2	LUMO	2
				HOMO−1	LUMO	48
				HOMO−1	LUMO+1	4
				HOMO−1	LUMO+4	2
				HOMO	LUMO	36
				HOMO	LUMO+1	3
S_2	3.7245	332.89	0.2282	HOMO−2	LUMO	14
				HOMO−2	LUMO+1	3
				HOMO−1	LUMO	25
				HOMO−1	LUMO+1	3
				HOMO	LUMO	45
				HOMO	LUMO+1	4
S_3	3.7775	328.21	0.0005	HOMO−3	LUMO+1	5
				HOMO−2	LUMO	57
				HOMO−2	LUMO+1	13
				HOMO−1	LUMO	12
				HOMO−1	LUMO+1	3
				HOMO	LUMO	4
S_4	3.9103	317.07	0.5224	HOMO−3	LUMO	4
				HOMO−2	LUMO	7
				HOMO−2	LUMO+1	11
				HOMO−1	LUMO+1	52
				HOMO	LUMO	4
				HOMO	LUMO+1	16
S_5	4.0132	308.94	0.1065	HOMO−3	LUMO	21
				HOMO−3	LUMO+1	4
				HOMO−2	LUMO+1	2
				HOMO−1	LUMO+1	15
				HOMO	LUMO	3
				HOMO	LUMO+1	45
S_6	4.2495	291.76	0.0471	HOMO−3	LUMO	5
				HOMO−2	LUMO	10
				HOMO−2	LUMO+1	59
				HOMO−1	LUMO+1	16

				HOMO	LUMO	2
S_7	4.3705	283.68	0.3813	HOMO−3	LUMO	32
				HOMO−3	LUMO+1	16
				HOMO−2	LUMO	3
				HOMO−1	LUMO	8
				HOMO	LUMO+1	23
				HOMO	LUMO+5	4
S_8	4.5958	269.78	0.1928	HOMO−4	LUMO	3
				HOMO−3	LUMO	21
				HOMO−3	LUMO+1	36
				HOMO−2	LUMO+1	6
				HOMO−1	LUMO+2	3
				HOMO−1	LUMO+3	4
				HOMO−1	LUMO+4	4
				HOMO−1	LUMO+5	3
				HOMO	LUMO+1	5
S_9	4.6952	264.06	0.2843	HOMO−2	LUMO+2	20
				HOMO−1	LUMO+2	21
				HOMO	LUMO+2	49
S_{10}	4.8302	256.68	0.2368	HOMO−4	LUMO+1	4
				HOMO−3	LUMO	6
				HOMO−3	LUMO+1	4
				HOMO−2	LUMO+3	7
				HOMO−1	LUMO+3	25
				HOMO−1	LUMO+4	20
				HOMO	LUMO+3	4
				HOMO	LUMO+4	9

S_1~S_{10}

Table S22. Summary of the TD-DFT calculation results of **3a** (S_1 state geometry) at MN15/cc-PVTZ level of theory.

Excited states	Energy (eV)	Wavelength (nm)	Oscillator strength	Major contributions (%)		
S_1	3.0842	401.99	0.1401	HOMO	LUMO	92
S_2	3.5669	347.6	0.0836	HOMO−2	LUMO	45
				HOMO−2	LUMO+1	2
				HOMO−1	LUMO	20
				HOMO	LUMO+1	23
				HOMO	LUMO+4	3

S_3	3.59	345.36	0.0634	HOMO−3	LUMO+1	4
				HOMO−2	LUMO	25
				HOMO−2	LUMO+1	5
				HOMO−1	LUMO	64
S_4	3.832	323.55	0.0689	HOMO−3	LUMO	18
				HOMO−2	LUMO	12
				HOMO−1	LUMO	5
				HOMO−1	LUMO+1	6
				HOMO	LUMO+1	50
S_5	3.9384	314.81	0.192	HOMO−3	LUMO	17
				HOMO−2	LUMO+1	11
				HOMO−1	LUMO	2
				HOMO−1	LUMO+1	45
				HOMO	LUMO+1	15
S_6	4.1258	300.51	0.1496	HOMO−3	LUMO	28
				HOMO−3	LUMO+1	3
				HOMO−2	LUMO	4
				HOMO−2	LUMO+1	43
				HOMO−1	LUMO+1	5
				HOMO	LUMO+1	8
S_7	4.324	286.73	0.1649	HOMO−3	LUMO	7
				HOMO−3	LUMO+1	10
				HOMO−2	LUMO	9
				HOMO−2	LUMO+1	21
				HOMO−1	LUMO+1	24
				HOMO−1	LUMO+5	5
				HOMO	LUMO+4	11
S_8	4.4913	276.05	0.0347	HOMO−2	LUMO+2	9
				HOMO−2	LUMO+3	3
				HOMO	LUMO+2	56
				HOMO	LUMO+3	21
S_9	4.5611	271.83	0.076	HOMO−4	LUMO	2
				HOMO−3	LUMO	9
				HOMO−3	LUMO+1	14
				HOMO−2	LUMO+1	8
				HOMO−2	LUMO+2	4

				HOMO−2	LUMO+3	3
				HOMO−1	LUMO+1	7
				HOMO	LUMO+2	19
				HOMO	LUMO+3	22
				HOMO	LUMO+4	4
S_{10}	4.6519	266.53	0.1723	HOMO−3	LUMO	10
				HOMO−3	LUMO+1	22
				HOMO−2	LUMO+3	11
				HOMO−1	LUMO+1	3
				HOMO−1	LUMO+5	3
				HOMO	LUMO+2	4
				HOMO	LUMO+3	33
				HOMO	LUMO+4	4

S_1~S_{10}

Table S23. Summary of the TD-DFT calculation results of **3a** (S_1 state geometry) at MN15/cc-PVTZ/ PCM = chloroform level of theory.

Excited states	Energy (eV)	Wavelength (nm)	Oscillator strength	Major contributions (%)		
S_1	3.0686	404.04	0.2464	HOMO	LUMO	94
S_2	3.5287	351.36	0.2682	HOMO−2	LUMO	11
				HOMO−1	LUMO	69
				HOMO	LUMO+1	14
S_3	3.5877	345.58	0.0109	HOMO−3	LUMO+1	3
				HOMO−2	LUMO	65
				HOMO−2	LUMO+1	6
				HOMO−1	LUMO	18
S_4	3.8229	324.32	0.1382	HOMO−3	LUMO	16
				HOMO−2	LUMO	7
				HOMO−1	LUMO	5
				HOMO−1	LUMO+1	3
				HOMO	LUMO+1	62
S_5	3.9145	316.73	0.3056	HOMO−3	LUMO	14
				HOMO−2	LUMO+1	14
				HOMO−1	LUMO+1	55
				HOMO	LUMO+1	8
S_6	4.1188	301.02	0.2469	HOMO−3	LUMO	38
				HOMO−3	LUMO+1	3

				HOMO−2	LUMO	3
				HOMO−2	LUMO+1	34
				HOMO−1	LUMO+1	3
				HOMO	LUMO+1	10
S_7	4.2879	289.15	0.27	HOMO−3	LUMO	7
				HOMO−3	LUMO+1	7
				HOMO−2	LUMO	10
				HOMO−2	LUMO+1	29
				HOMO−1	LUMO+1	24
				HOMO−1	LUMO+5	4
				HOMO	LUMO+3	3
				HOMO	LUMO+4	5
S_8	4.5711	271.24	0.1708	HOMO−5	LUMO	6
				HOMO−3	LUMO	13
				HOMO−3	LUMO+1	30
				HOMO−2	LUMO+1	7
				HOMO−1	LUMO+1	8
				HOMO−1	LUMO+5	4
				HOMO	LUMO+3	18
				HOMO	LUMO+4	6
S_9	4.6965	263.99	0.1244	HOMO−2	LUMO+2	11
				HOMO−2	LUMO+3	4
				HOMO	LUMO+2	63
				HOMO	LUMO+3	8
				HOMO	LUMO+4	2
S_{10}	4.7581	260.58	0.7056	HOMO−3	LUMO	2
				HOMO−3	LUMO+1	15
				HOMO−2	LUMO+3	3
				HOMO−2	LUMO+5	3
				HOMO−1	LUMO+4	5
				HOMO	LUMO+2	5
				HOMO	LUMO+3	32
				HOMO	LUMO+4	4
				HOMO	LUMO+5	10

S_1~S_{10}

Table S24. Summary of the TD-DFT calculation results of **3b** (S_1 state geometry) at MN15/cc-PVTZ level of theory.

Excited states	Energy (eV)	Wavelength (nm)	Oscillator strength	Major contributions (%)

S_1	3.084	402.03	0.1369	HOMO	LUMO	92
S_2	3.572	347.1	0.1015	HOMO−2	LUMO	37
				HOMO−1	LUMO	29
				HOMO	LUMO+1	24
				HOMO	LUMO+4	3
S_3	3.5885	345.5	0.0472	HOMO−3	LUMO+1	4
				HOMO−2	LUMO	32
				HOMO−2	LUMO+1	5
				HOMO−1	LUMO	55
S_4	3.836	323.21	0.0539	HOMO−3	LUMO	20
				HOMO−2	LUMO	12
				HOMO−1	LUMO	4
				HOMO−1	LUMO+1	8
				HOMO	LUMO+1	47
S_5	3.9423	314.5	0.1893	HOMO−3	LUMO	16
				HOMO−2	LUMO+1	10
				HOMO−1	LUMO	2
				HOMO−1	LUMO+1	43
				HOMO	LUMO+1	18
S_6	4.1281	300.34	0.1619	HOMO−3	LUMO	28
				HOMO−3	LUMO+1	3
				HOMO−2	LUMO	3
				HOMO−2	LUMO+1	41
				HOMO−1	LUMO+1	6
				HOMO	LUMO+1	8
S_7	4.3299	286.35	0.1547	HOMO−3	LUMO	6
				HOMO−3	LUMO+1	10
				HOMO−2	LUMO	9
				HOMO−2	LUMO+1	23
				HOMO−1	LUMO+1	22
				HOMO−1	LUMO+5	5
				HOMO	LUMO+4	11
S_8	4.4334	279.66	0.0378	HOMO−2	LUMO+2	12
				HOMO	LUMO+2	78
S_9	4.5766	270.91	0.0942	HOMO−3	LUMO	11

				HOMO−3	LUMO+1	15
				HOMO−2	LUMO+1	8
				HOMO−2	LUMO+3	6
				HOMO−1	LUMO+1	6
				HOMO	LUMO+3	40
				HOMO	LUMO+4	4
S_{10}	4.6416	267.12	0.1243	HOMO−3	LUMO	8
				HOMO−3	LUMO+1	20
				HOMO−2	LUMO+2	2
				HOMO−2	LUMO+3	8
				HOMO−1	LUMO+1	3
				HOMO−1	LUMO+2	2
				HOMO−1	LUMO+5	3
				HOMO	LUMO+3	39
				HOMO	LUMO+4	3

S_1~S_{10}

Table S25. Summary of the TD-DFT calculation results of **3b** (S_1 state geometry) at MN15/cc-PVTZ/ PCM = chloroform level of theory.

Excited states	Energy (eV)	Wavelength (nm)	Oscillator strength	Major contributions (%)		
S_1	3.0688	404.01	0.2425	HOMO	LUMO	94
S_2	3.5302	351.21	0.2771	HOMO−2	LUMO	8
				HOMO−1	LUMO	74
				HOMO	LUMO+1	13
S_3	3.5918	345.18	0.0048	HOMO−3	LUMO+1	3
				HOMO−2	LUMO	67
				HOMO−2	LUMO+1	6
				HOMO−1	LUMO	14
				HOMO	LUMO+1	4
S_4	3.8282	323.87	0.1114	HOMO−3	LUMO	18
				HOMO−2	LUMO	8
				HOMO−1	LUMO	5
				HOMO−1	LUMO+1	4
				HOMO	LUMO+1	58
S_5	3.9181	316.44	0.316	HOMO−3	LUMO	12
				HOMO−2	LUMO+1	13
				HOMO−1	LUMO+1	54
				HOMO	LUMO+1	11

S_6	4.1195	300.97	0.2681	HOMO−3	LUMO	39
				HOMO−3	LUMO+1	3
				HOMO−2	LUMO	2
				HOMO−2	LUMO+1	32
				HOMO−1	LUMO+1	5
				HOMO	LUMO+1	10
S_7	4.2962	288.59	0.2513	HOMO−3	LUMO	6
				HOMO−3	LUMO+1	7
				HOMO−2	LUMO	10
				HOMO−2	LUMO+1	32
				HOMO−1	LUMO+1	22
				HOMO−1	LUMO+5	4
				HOMO	LUMO+3	3
				HOMO	LUMO+4	6
S_8	4.5638	271.67	0.1245	HOMO−4	LUMO	6
				HOMO−3	LUMO	10
				HOMO−3	LUMO+1	25
				HOMO−2	LUMO+1	7
				HOMO−2	LUMO+2	2
				HOMO−1	LUMO+1	7
				HOMO−1	LUMO+5	4
				HOMO	LUMO+2	17
				HOMO	LUMO+3	8
				HOMO	LUMO+4	8
S_9	4.6453	266.9	0.2021	HOMO−3	LUMO	6
				HOMO−3	LUMO+1	7
				HOMO−2	LUMO+2	14
				HOMO	LUMO+2	59
				HOMO	LUMO+3	3
				HOMO	LUMO+4	2
S_{10}	4.7625	260.34	0.6602	HOMO−3	LUMO+1	14
				HOMO−2	LUMO+3	4
				HOMO−2	LUMO+5	2
				HOMO−1	LUMO+4	4
				HOMO	LUMO+3	36
				HOMO	LUMO+4	11
				HOMO	LUMO+5	9

S_1~S_{10}

Analysis of the molecular contribution in the UV-Vis absorption pattern of dioxaza[8]circulene **3a**

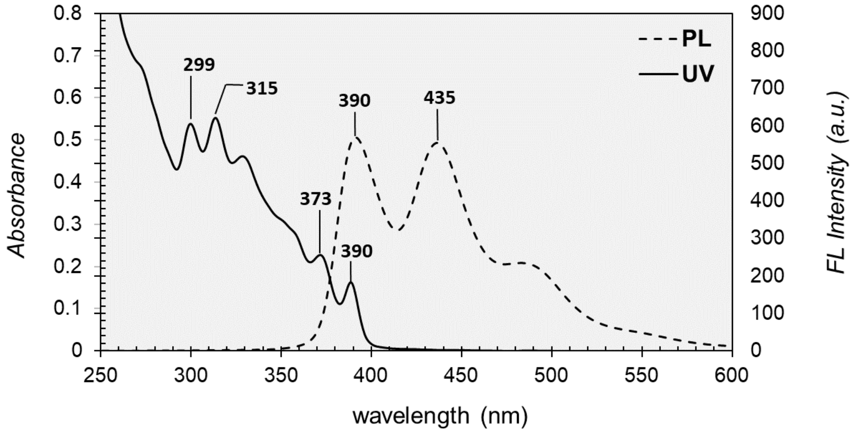

UV/vis absorption and PL spectra of **3a** in $CHCl_3$ solution (20 μM).

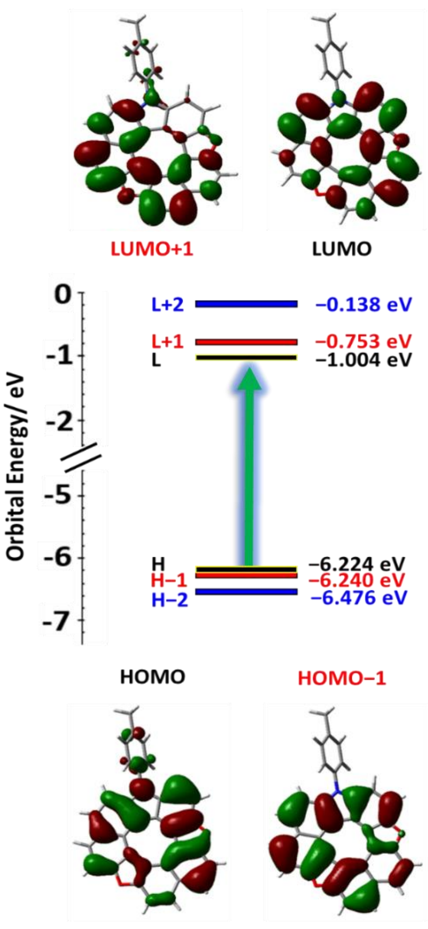

Frontier Kohn-Sham molecular orbitals of **3a** (S_0 state geometry) and TD-DFT calculated electronic transitions at MN15/cc-PVTZ level of theory.

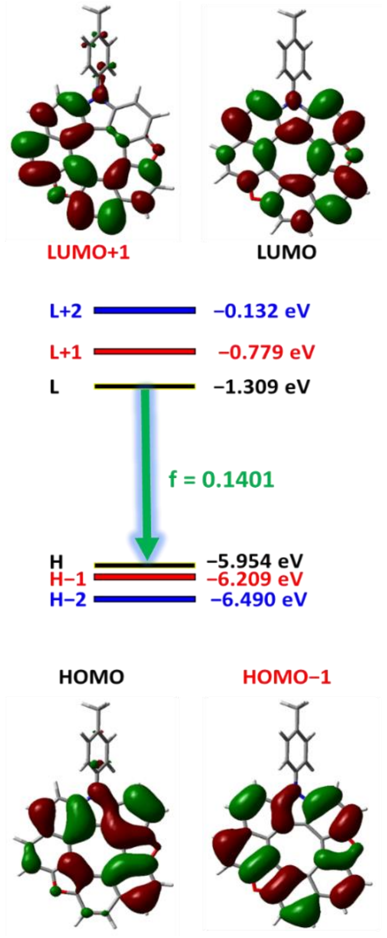

Frontier Kohn-Sham molecular orbitals of **3a** (S_1 state geometry) and TD-DFT calculated electronic transitions at MN15/cc-PVTZ level of theory.

The first absorption peak of **3a** were attributed to the HOMO → LOMO transitions (H → L), where the electron cloud distributed across the entire molecule. The absorption band at 373 nm possibly attributed to the equal contribution of both HOMO−1 → LUMO and HOMO−2 → LUMO transitions.

Figure S2. Simulated UV-Vis spectra of oxaza[8]circulene **3a** and **3b**

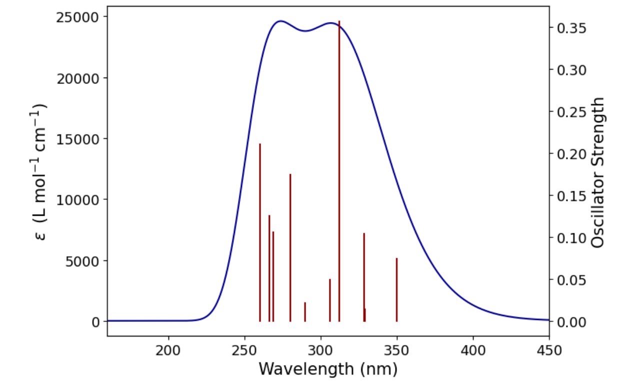

FIGURE S2a. Simulated UV-Vis absorption spectra of **3a** at MN15/cc-PVTZ; Wavelength (nm) = 350.02, Oscillator Strength = 0.0736.

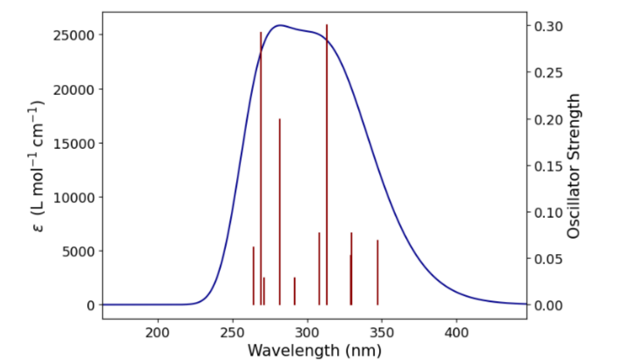

FIGURE S2b. Simulated UV-Vis absorption spectra of **3b** at MN15/cc-PVTZ; Wavelength (nm) = 347.15, Oscillator Strength = 0.0686.

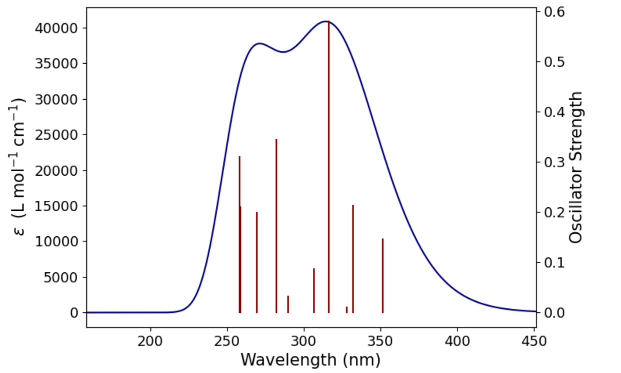

FIGURE S2c. Simulated UV-Vis absorption spectra of **3a** at MN15/cc-PVTZ/ PCM = chloroform; Wavelength (nm) = 351.22, Oscillator Strength = 0.1454.

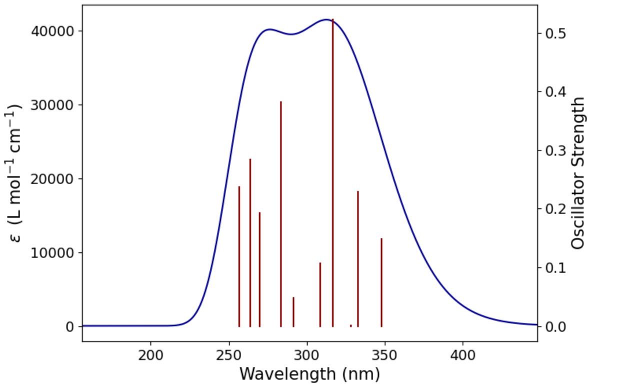

FIGURE S2d. Simulated UV-Vis absorption spectra of **3b** at MN15/cc-PVTZ/ PCM = chloroform; Wavelength (nm) = 348.17, Oscillator Strength = 0.1480.

Theoretically calculated radiative rate constants $k_{f,calcd}$

$$\boldsymbol{k_{f,calcd}} = \frac{16\pi^3}{3h\varepsilon_0}\nu^3(\mathrm{D}+\mathrm{G}) \quad Equation\ (S1)$$

Table S26. Calculation of the radiative rate constants $k_{f,calcd}$ of dioxaza[*8*]circulene **3a**

	λ_{ex}[a] /nm	ν^b /cm^{-1}	ν^3 (× 10^{13})	(D) (×10^{-40} (esu^2 cm^2)[c]	(G) (×10^{-40} (erg^2 G^{-2})[d]	(D + G) (×10^{-40})	$k_{f,calcd}$ /ns^{-1}	Φ_f
**3a**	379	26385.2	1.8				0.06899	6.4%
	340	29411.8	2.5	119915.5 [e]	7.852 [e]	119923.3 [e]	0.09556	6.3%
	261	38314.2	5.6				0.21124	6.3%
	379	26385.2	1.8				0.12196	6.4%
	340	29411.8	2.5	211996.4 [f]	6.474 [f]	212002.9 [f]	0.16893	6.3%
	261	38314.2	5.6				0.37344	6.3%
**3b**	379	26385.2	1.8				0.06744	
	340	29411.8	2.5	117214.1 [e]	8.252 [e]	117222.3 [e]	0.09340	--
	261	38314.2	5.6				0.20647	
	379	26385.2	1.8				0.12002	
	340	29411.8	2.5	208620.8 [f]	6.936 [f]	208627.8 [f]	0.16623	--
	261	38314.2	5.6				0.36748	

[a] For the $S_1 \rightarrow S_0$ transitions. [b] Deexcitation energy (in cm^{-1}) was converted as follows: ν (cm^{-1}) = 10^7 / λ_{ex} (nm). [c] Electric dipole strength = $|\mu|^2$. [d] Magnetic dipole strength = $|m|^2$. [e] Calculated at MN15/cc-PVTZ level of theory. [f] Calculated at MN15/cc-PVTZ/ PCM = chloroform level of theory.

ETDM and MTDM of dioxaza[8]circulene

Table S27. Calculation of ETDM and MTDM of dioxaza[*8*]circulene **3a**

	$S_1 \rightarrow S_0$ transition											
	$\lvert\mu_x\rvert$	$\lvert\mu_y\rvert$	$\lvert\mu_z\rvert$	$\lvert\mu'\rvert$	$\lvert m_x\rvert$	$\lvert m_y\rvert$	$\lvert m_z\rvert$	$\lvert m'\rvert$	$\theta_{\mu,m}$ (deg)[c]	cos($\theta_{\mu,m}$)	(R) (×10^{-40} (erg esu cm G^{-1})[d]	g_{cal}[e]
	(×10^{-20} esu cm)[a]				(×10^{-20} erg G^{-1})[b]							
**3a**[f]	283.5	−198.9	−2.6	346.3	0.0	−0.1	2.8	2.8	89.9°	0.001	1.2	~ 0.000
**3b**[g]	377.4	−263.8	−3.4	460.4	0.0	−0.1	2.5	2.5	89.9°	0.002	2.7	~ 0.000
**3a**[f]	−280.7	−196.0	−3.0	342.4	0.0	0.1	−2.9	2.9	90.1°	−0.001	−1.3	~ 0.000
**3b**[g]	−375.2	−260.5	−3.9	456.8	0.0	0.1	−2.6	2.6	90.1°	−0.002	−2.6	~ 0.000

[a] Electric transition dipole moments (ETDM) for the $S_1 \rightarrow S_0$ transitions. [b] Magnetic transition dipole moments (MTDM) for the $S_1 \rightarrow S_0$ transitions. [c] The angle between ETDM and MTDM vectors. [d] Rotational strength. [e] Dimensionless values. [f] Calculated at MN15/cc-PVTZ level of theory. [g] Calculated at MN15/cc-PVTZ/ PCM = chloroform level of theory.

❖ *TEDM densities for the $S_1 \rightarrow S_0$ transition of dioxaza[8]circulenes*

Table S28. The transition electric dipole moment (TEDM) densities for the $S_1 \rightarrow S_0$ transition (isosurface value: 0.003 a.u.) of **3a** calculated at the MN15/cc-PVTZ level of theory.

	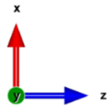	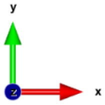	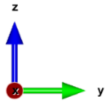
**3a**	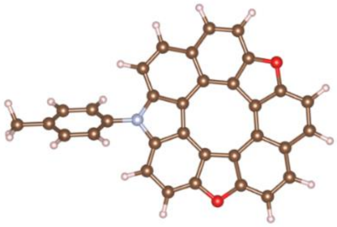	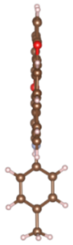	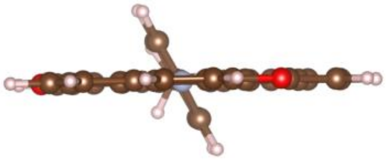
μ_x	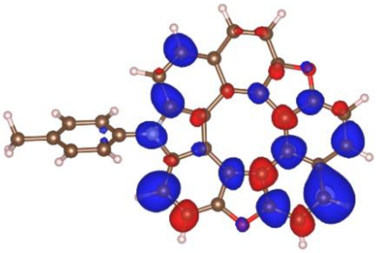	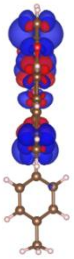	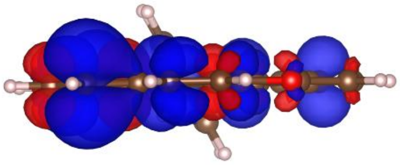
μ_y	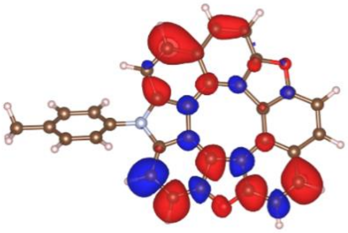	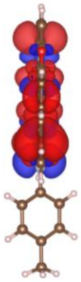	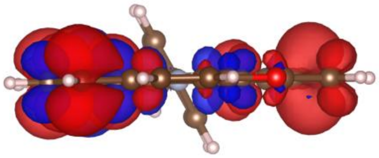
μ_z	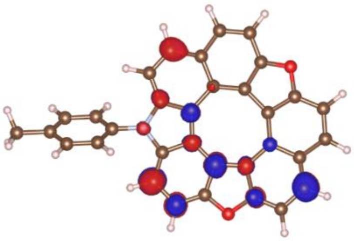	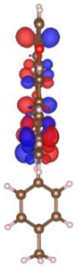	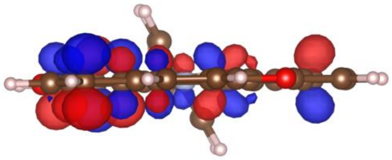
$\lvert\mu'\rvert$	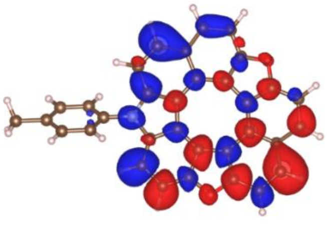	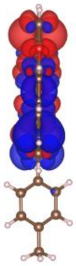	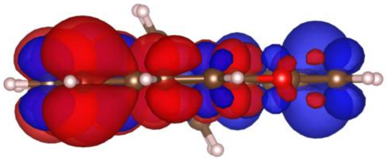

Table S29. The transition electric dipole moment (TEDM) densities for the $S_1 \rightarrow S_0$ transition (isosurface value: 0.003 a.u.) of **3b** calculated at the MN15/cc-PVTZ level of theory.

	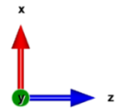	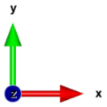	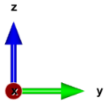
**3b**	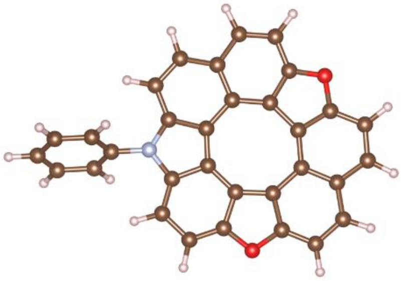	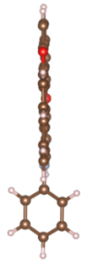	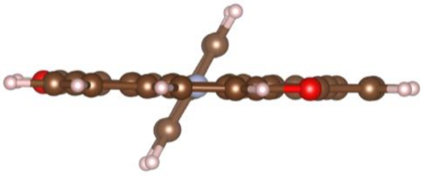
μ_x	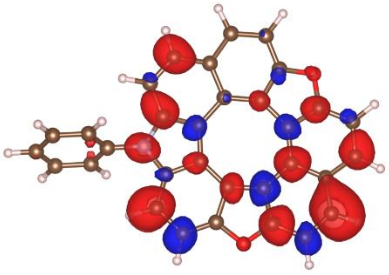	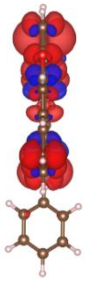	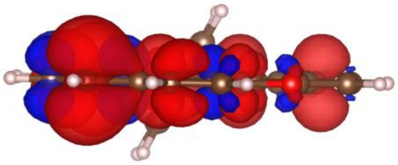
μ_y	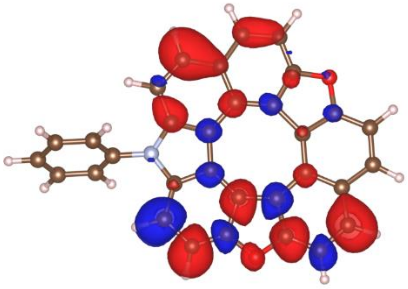	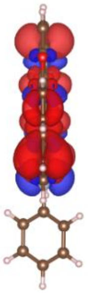	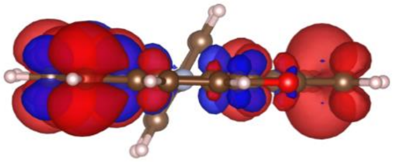
μ_z	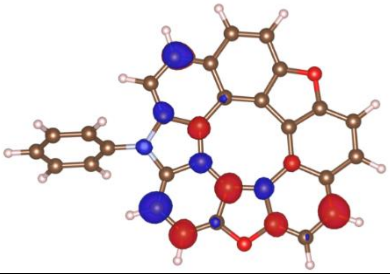	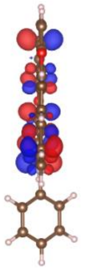	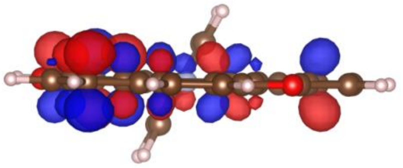
$\|\mu'\|$	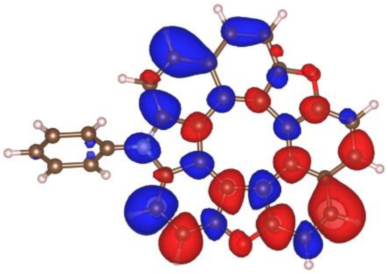	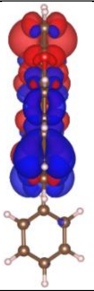	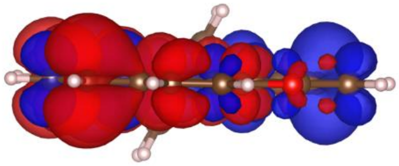

❖ *TMDM densities for the $S_1 \rightarrow S_0$ transition of dioxaza[8]circulenes*

Table S30. The transition magnetic dipole moment (TMDM) densities for the $S_1 \rightarrow S_0$ transition (isosurface value: 0.003 a.u.) of **3a** calculated at the MN15/cc-PVTZ level of theory.

	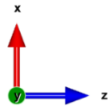	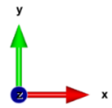	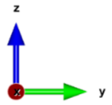
**3a**	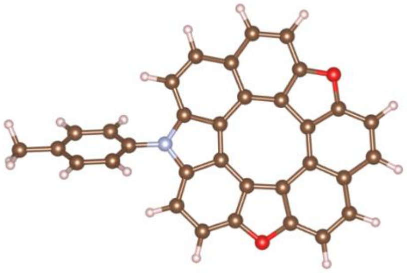	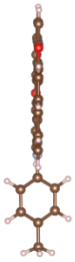	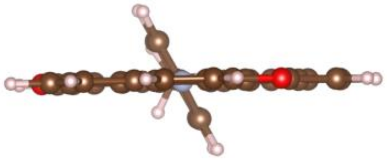
m_x	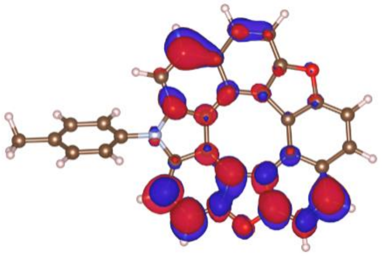	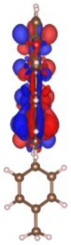	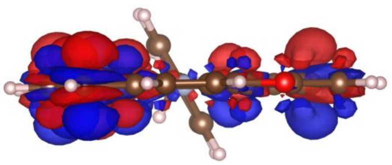
m_y	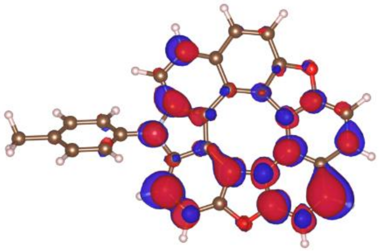	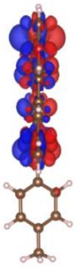	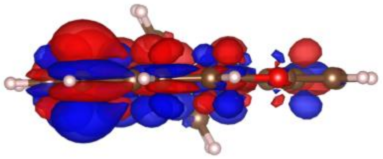
m_z	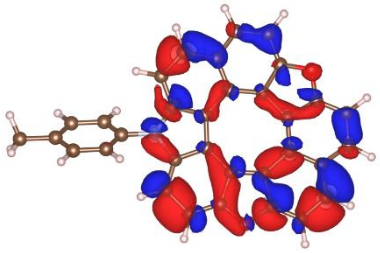	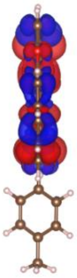	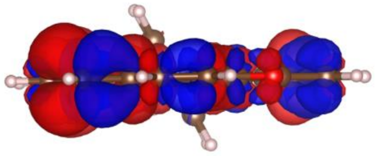
$\lvert m' \rvert$	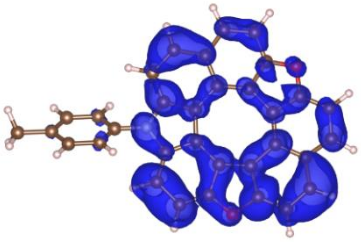	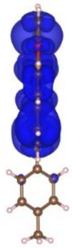	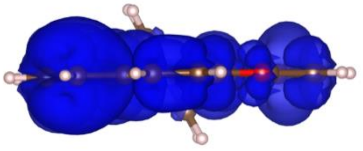

Table S31. The transition magnetic dipole moment (TMDM) densities for the $S_1 \rightarrow S_0$ transition (isosurface value: 0.003 a.u.) of **3b** calculated at the MN15/cc-PVTZ level of theory.

	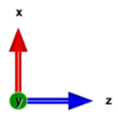	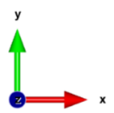	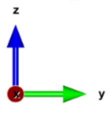
**3b**	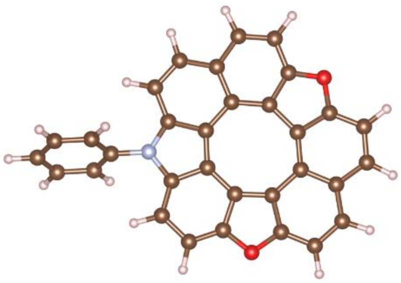	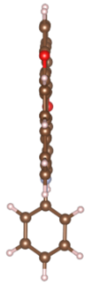	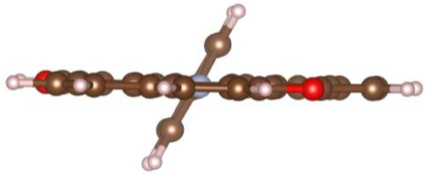
m_x	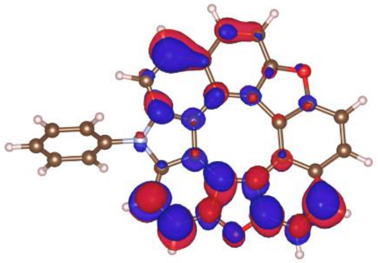	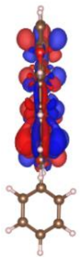	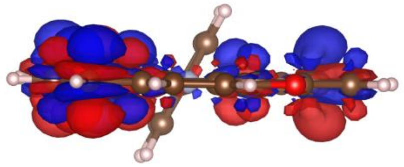
m_y	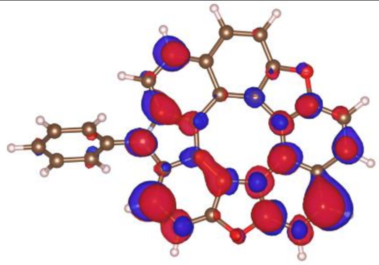	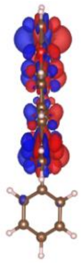	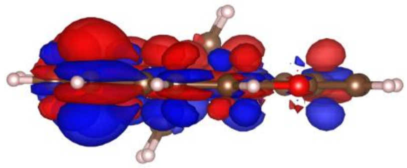
m_z	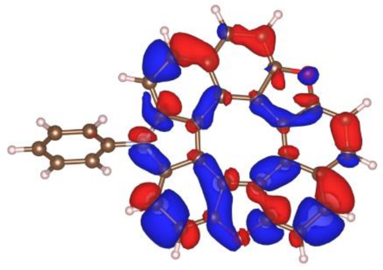	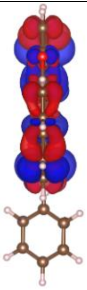	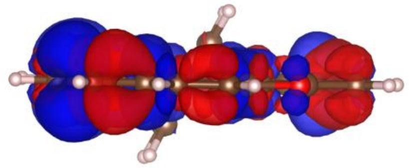
$\|m'\|$	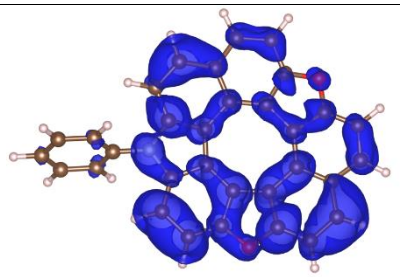	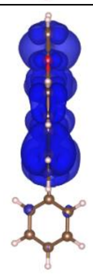	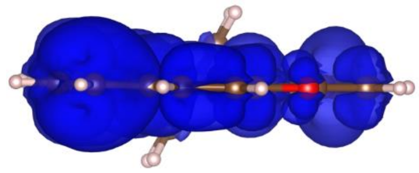

❖ *Hole-electron analysis for the $S_1 \rightarrow S_0$ transition of dioxaza[8]circulenes*

Table S32. The hole-electron analysis for the $S_1 \rightarrow S_0$ transition (isosurface value: 0.0005 a.u.) calculated at the MN15/cc-PVTZ level of theory.

	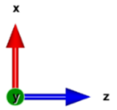	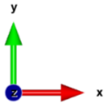	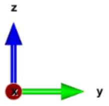
**3a**	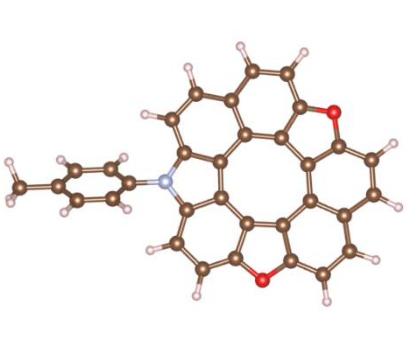	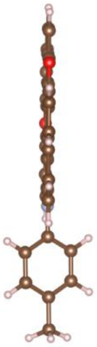	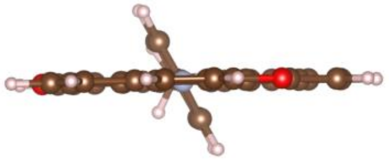
**3a**	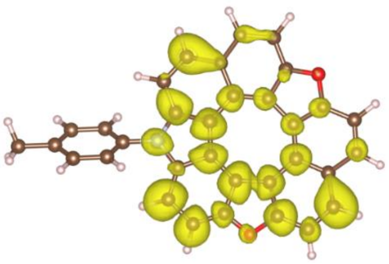	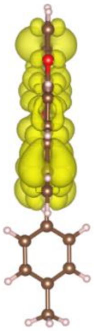	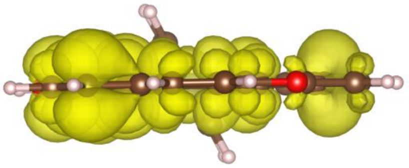
**3b**	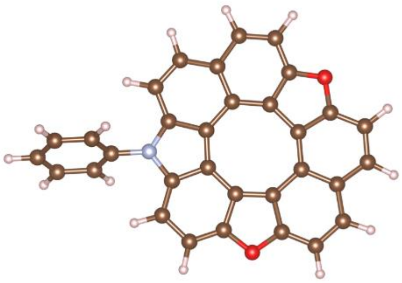	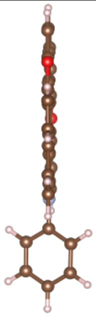	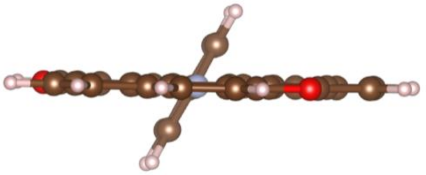
**3b**	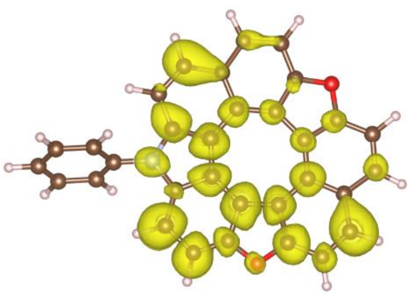	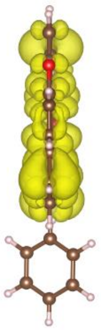	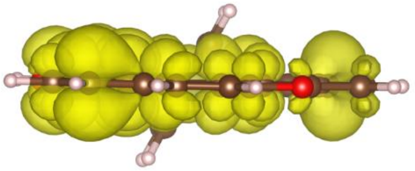

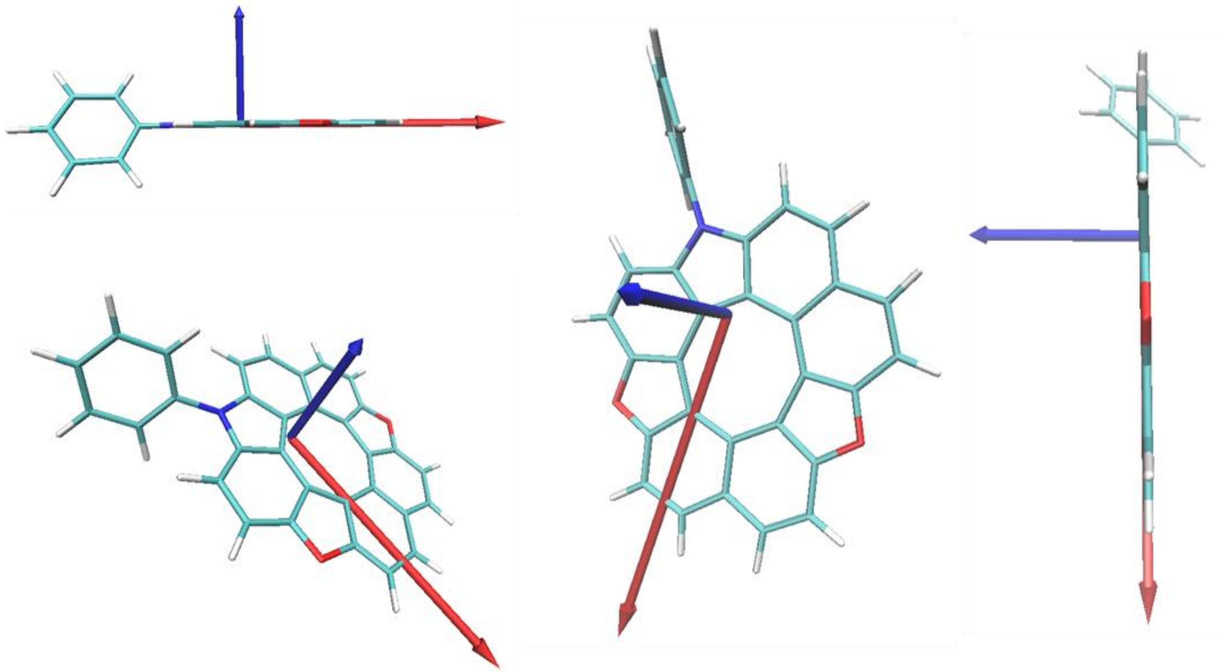

The vectors of transition electric **|μ'|** (red), and magnetic **|m'|** (blue) dipole moment (TEDM) & (TMDM) densities for the $S_1 \rightarrow S_0$ transition of **3a** calculated at the MN15/cc-PVTZ level of theory. the length of vectors is amplified for clarity.

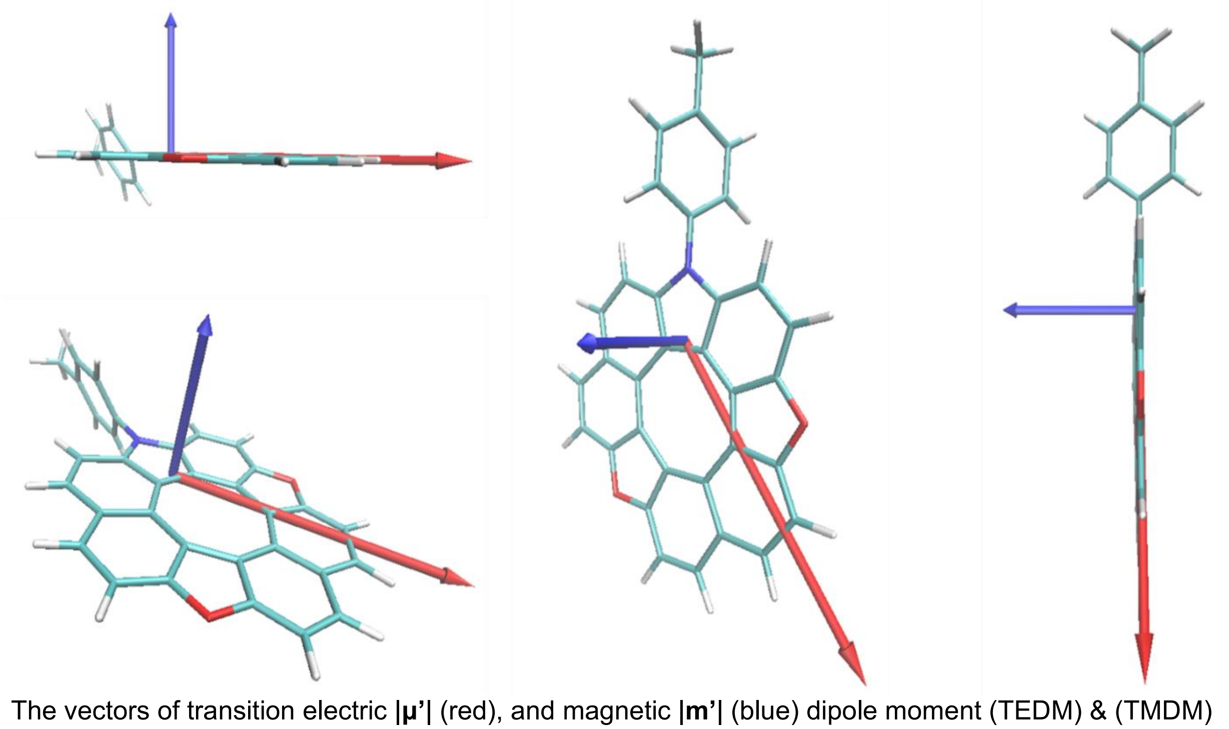

The vectors of transition electric **|μ'|** (red), and magnetic **|m'|** (blue) dipole moment (TEDM) & (TMDM) densities for the $S_1 \rightarrow S_0$ transition of **3b** calculated at the MN15/cc-PVTZ level of theory. the length of vectors is amplified for clarity.

14. **Supplementary Note 7:** *X-ray crystal data*

Dioxaza[8]circulene **3a**

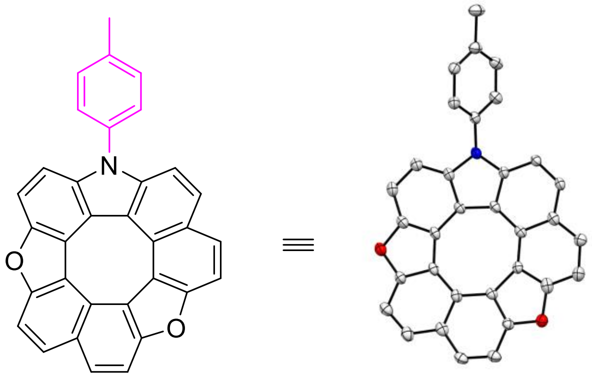

3a (CCDC 2255588) with ellipsoids at 30% probability. (H atoms were omitted for clarity).

Bond precision: C-C = 0.0045 A Wavelength=1.54184

Cell:	a=8.1169(4)	b=11.6015(6)	c=11.6925(7)
	alpha=77.613(5)	beta=75.660(5)	gamma=76.727(4)
Temperature:	123 K		

	Calculated	Reported
Volume	1023.65(10)	1023.65(10)
Space group	P -1	P -1
Hall group	-P 1	-P 1
Moiety formula	C33 H17 N O2	C33 H17 N O2
Sum formula	C33 H17 N O2	C33 H17 N O2
Mr	459.48	459.48
Dx,g cm-3	1.491	1.491
Z	2	2
Mu (mm-1)	0.734	0.734
F000	476.0	476.0
F000'	477.39	
h,k,lmax	10,14,14	10,14,14
Nref	4268	4109
Tmin,Tmax	0.970,0.980	0.936,1.000
Tmin'	0.951	

Correction method= # Reported T Limits: Tmin=0.936 Tmax=1.000
AbsCorr = MULTI-SCAN

Data completeness= 0.963 Theta(max)= 75.790

R(reflections)= 0.0684(3202) wR2(reflections)= 0.1872(4109)

S = 1.051 Npar= 326

```
The following ALERTS were generated. Each ALERT has the format
       test-name_ALERT_alert-type_alert-level.
Click on the hyperlinks for more details of the test.
```

Alert level C

```
DIFMX02_ALERT_1_C  The maximum difference density is > 0.1*ZMAX*0.75
            The relevant atom site should be identified.
PLAT094_ALERT_2_C Ratio of Maximum / Minimum Residual Density ....       2.47 Report
PLAT097_ALERT_2_C Large Reported Max.  (Positive) Residual Density       0.70 eA-3
PLAT230_ALERT_2_C Hirshfeld Test Diff for    O001       --C00S     .      6.0 s.u.
PLAT230_ALERT_2_C Hirshfeld Test Diff for    O002       --C00F     .      6.3 s.u.
PLAT230_ALERT_2_C Hirshfeld Test Diff for    C007       --C00S     .      5.5 s.u.
PLAT340_ALERT_3_C Low Bond Precision on  C-C Bonds ...............     0.0045 Ang.
PLAT906_ALERT_3_C Large K Value in the Analysis of Variance ......      7.995 Check
PLAT911_ALERT_3_C Missing FCF Refl Between Thmin & STh/L=    0.600         14 Report
```

Alert level G

```
PLAT003_ALERT_2_G Number of Uiso or Uij Restrained non-H Atoms ...          5 Report
PLAT178_ALERT_4_G The CIF-Embedded .res File Contains SIMU Records          1 Report
PLAT187_ALERT_4_G The CIF-Embedded .res File Contains RIGU Records          1 Report
PLAT190_ALERT_3_G A Non-default RIGU Restraint Value for First Par     0.0020 Report
PLAT190_ALERT_3_G A Non-default RIGU Restraint Value for SecondPar    0.0020 Report
PLAT398_ALERT_2_G Deviating  C-O-C   Angle From 120 for O001     .      103.9 Degree
PLAT398_ALERT_2_G Deviating  C-O-C   Angle From 120 for O002     .      105.3 Degree
PLAT720_ALERT_4_G Number of Unusual/Non-Standard Labels ..........         53 Note
PLAT860_ALERT_3_G Number of Least-Squares Restraints .............         48 Note
PLAT912_ALERT_4_G Missing # of FCF Reflections Above STh/L=  0.600        145 Note
PLAT933_ALERT_2_G Number of HKL-OMIT Records in Embedded .res File          2 Note
PLAT941_ALERT_3_G Average HKL Measurement Multiplicity ...........        2.6 Low
PLAT978_ALERT_2_G Number C-C Bonds with Positive Residual Density.         15 Info
PLAT992_ALERT_5_G Repd & Actual _reflns_number_gt Values Differ by          2 Check
```

```
  0 ALERT level A - Most likely a serious problem - resolve or explain
  0 ALERT level B - A potentially serious problem, consider carefully
  9 ALERT level C - Check. Ensure it is not caused by an omission or oversight
 14 ALERT level G - General information/check it is not something unexpected

  1 ALERT type 1 CIF construction/syntax error, inconsistent or missing data
 10 ALERT type 2 Indicator that the structure model may be wrong or deficient
  7 ALERT type 3 Indicator that the structure quality may be low
  4 ALERT type 4 Improvement, methodology, query or suggestion
  1 ALERT type 5 Informative message, check
```

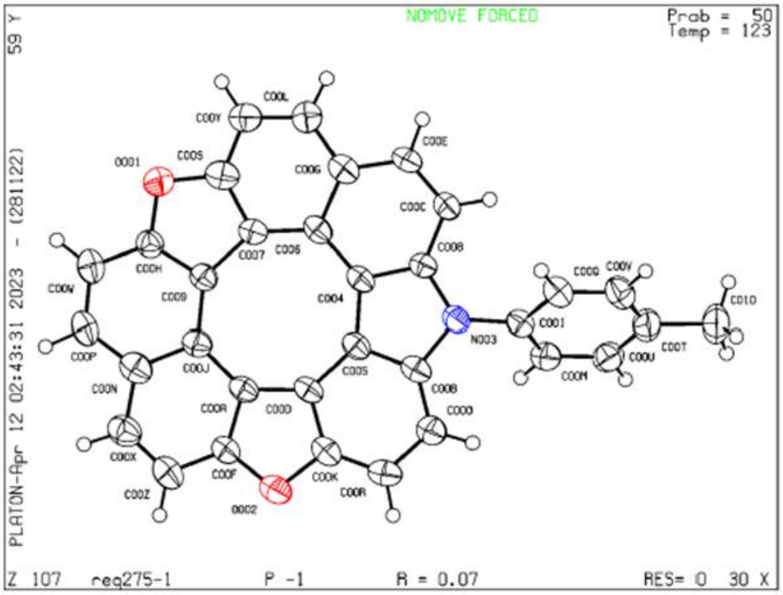

Dioxaza[8]circulene **3b**

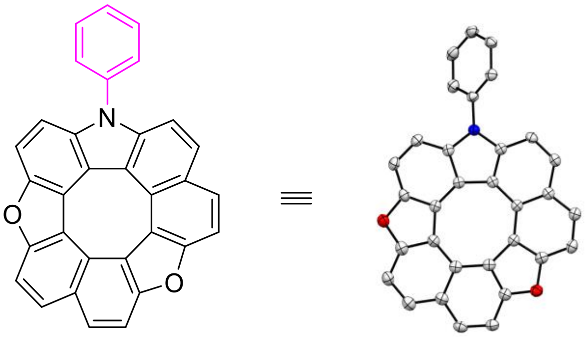

3b (CCDC 2254634) with ellipsoids at 50% probability. (H atoms were omitted for clarity).

Bond precision: C-C = 0.0026 A Wavelength=1.54184

Cell:	a=11.3563(3)	b=22.4385(6)	c=7.6520(2)
	alpha=90	beta=95.694(2)	gamma=90
Temperature:	103 K		

	Calculated	Reported
Volume	1940.25(9)	1940.25(9)
Space group	P 21/c	P 1 21/c 1
Hall group	-P 2ybc	-P 2ybc
Moiety formula	C32 H15 N O2	C32 H15 N O2
Sum formula	C32 H15 N O2	C32 H15 N O2
Mr	445.45	445.45
Dx,g cm-3	1.525	1.525
Z	4	4
Mu (mm-1)	0.756	0.756
F000	920.0	920.0
F000'	922.70	
h,k,lmax	14,28,9	14,27,9
Nref	4036	3959
Tmin,Tmax	0.939,0.979	0.772,1.000
Tmin'	0.934	

Correction method= # Reported T Limits: Tmin=0.772 Tmax=1.000
AbsCorr = MULTI-SCAN

Data completeness= 0.981 Theta(max)= 75.630

R(reflections)= 0.0464(3205) wR2(reflections)= 0.1187(3959)

S = 1.062 Npar= 316

```
The following ALERTS were generated. Each ALERT has the format
       test-name_ALERT_alert-type_alert-level.
Click on the hyperlinks for more details of the test.
```

Alert level C

```
PLAT906_ALERT_3_C Large K Value in the Analysis of Variance ......     2.101 Check
PLAT911_ALERT_3_C Missing FCF Refl Between Thmin & STh/L=    0.600         5 Report
PLAT918_ALERT_3_C Reflection(s) with I(obs) much Smaller I(calc) .         1 Check
```

Alert level G

```
PLAT398_ALERT_2_G Deviating  C-O-C   Angle From 120 for O001      .    105.4 Degree
PLAT398_ALERT_2_G Deviating  C-O-C   Angle From 120 for O002      .    105.4 Degree
PLAT720_ALERT_4_G Number of Unusual/Non-Standard Labels ..........        50 Note
PLAT912_ALERT_4_G Missing # of FCF Reflections Above STh/L=  0.600        69 Note
PLAT933_ALERT_2_G Number of HKL-OMIT Records in Embedded .res File         4 Note
PLAT941_ALERT_3_G Average HKL Measurement Multiplicity ...........       4.8 Low
PLAT978_ALERT_2_G Number C-C Bonds with Positive Residual Density.         7 Info
```

0 **ALERT level A** = Most likely a serious problem - resolve or explain
0 **ALERT level B** = A potentially serious problem, consider carefully
3 **ALERT level C** = Check. Ensure it is not caused by an omission or oversight
7 **ALERT level G** = General information/check it is not something unexpected

0 ALERT type 1 CIF construction/syntax error, inconsistent or missing data
4 ALERT type 2 Indicator that the structure model may be wrong or deficient
4 ALERT type 3 Indicator that the structure quality may be low
2 ALERT type 4 Improvement, methodology, query or suggestion
0 ALERT type 5 Informative message, check

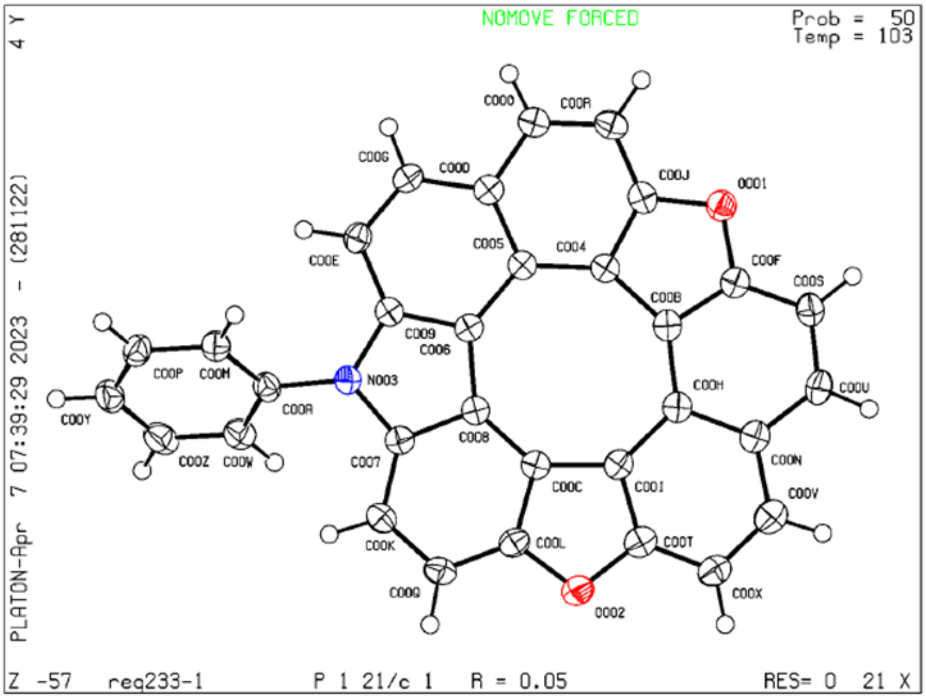

1-(4-(Thiophen-2-yl)phenyl)ethan-1-one **8a**

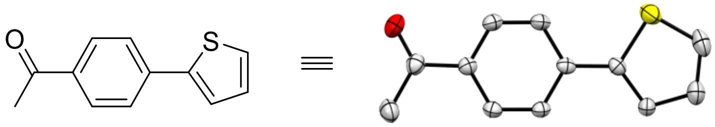

8a (CCDC 2310697) with ellipsoids at 30% probability. (H atoms were omitted for clarity).

Bond precision: C-C = 0.0038 A Wavelength=1.54184

Cell:	a=10.9123(2)	b=7.6526(2)	c=24.0181(5)
	alpha=90	beta=90	gamma=90
Temperature:	263 K		

	Calculated	Reported
Volume	2005.69(8)	2005.69(8)
Space group	P b c a	P b c a
Hall group	-P 2ac 2ab	-P 2ac 2ab
Moiety formula	C12 H10 O S	C12 H10 O S
Sum formula	C12 H10 O S	C12 H10 O S
Mr	202.26	202.26
Dx,g cm-3	1.340	1.340
Z	8	8
Mu (mm-1)	2.537	2.537
F000	848.0	848.0
F000'	852.73	
h,k,lmax		13,9,29
Nref		2029
Tmin,Tmax	0.779,0.955	0.792,1.000
Tmin'	0.685	

Correction method= # Reported T Limits: Tmin=0.792 Tmax=1.000
AbsCorr = MULTI-SCAN

Data completeness= Theta(max)= 74.985

R(reflections)= 0.0778(1660) wR2(reflections)= 0.2474(2029)

S = 1.053 Npar= 128

The following ALERTS were generated. Each ALERT has the format
test-name_ALERT_alert-type_alert-level.
Click on the hyperlinks for more details of the test.

Alert level C

PLAT230_ALERT_2_C Hirshfeld Test Diff for C00C --C00D . 6.3 s.u.
PLAT906_ALERT_3_C Large K Value in the Analysis of Variance ...... 2.450 Check
PLAT911_ALERT_3_C Missing FCF Refl Between Thmin & STh/L= 0.600 4 Report
6 0 0, 1 9 1, 1 9 2, 5 0 2,

Alert level G

PLAT072_ALERT_2_G SHELXL First Parameter in WGHT Unusually Large 0.18 Report
PLAT720_ALERT_4_G Number of Unusual/Non-Standard Labels .... 24 Note

S001	O002	C003	H003	C004	C005	H005	C006
C007	C008	H008	C009	H009	C00A	H00A	C00B
C00C	H00C	C00D	H00D	C00E	H00B	H00E	H00F

PLAT912_ALERT_4_G Missing # of FCF Reflections Above STh/L= 0.600 35 Note
PLAT978_ALERT_2_G Number C-C Bonds with Positive Residual Density. 2 Info

---

0 **ALERT level A** = Most likely a serious problem - resolve or explain
0 **ALERT level B** = A potentially serious problem, consider carefully
3 **ALERT level C** = Check. Ensure it is not caused by an omission or oversight
4 **ALERT level G** = General information/check it is not something unexpected

0 ALERT type 1 CIF construction/syntax error, inconsistent or missing data
3 ALERT type 2 Indicator that the structure model may be wrong or deficient
2 ALERT type 3 Indicator that the structure quality may be low
2 ALERT type 4 Improvement, methodology, query or suggestion
0 ALERT type 5 Informative message, check

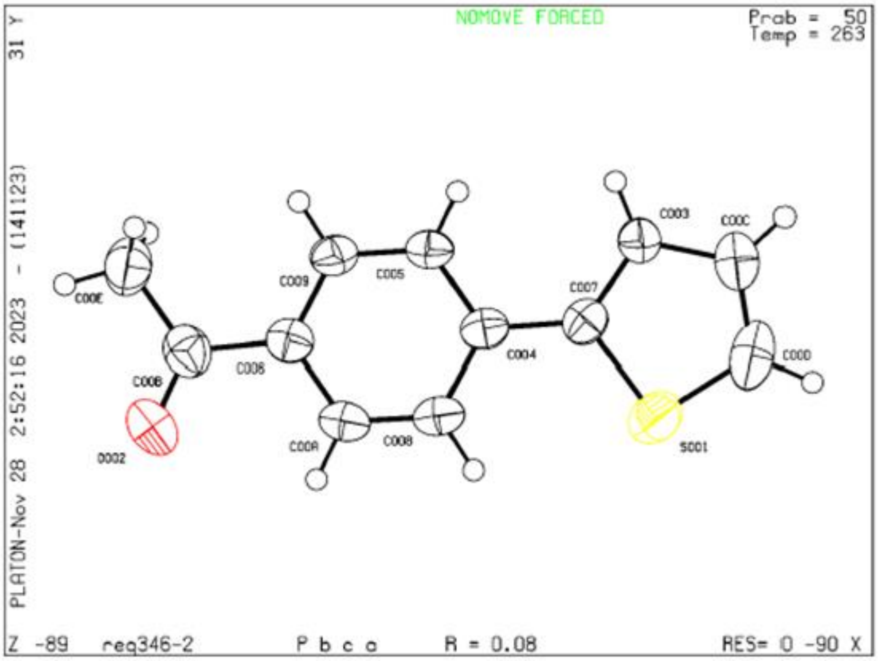

15. Supplementary Note 8: *CV charts*

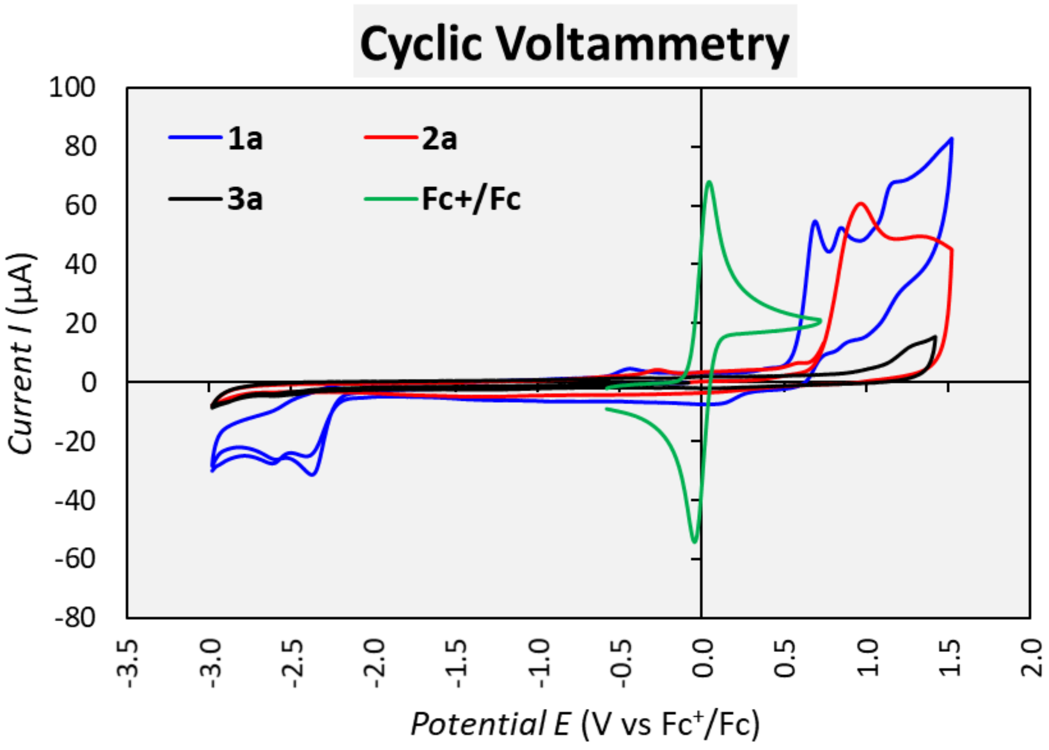

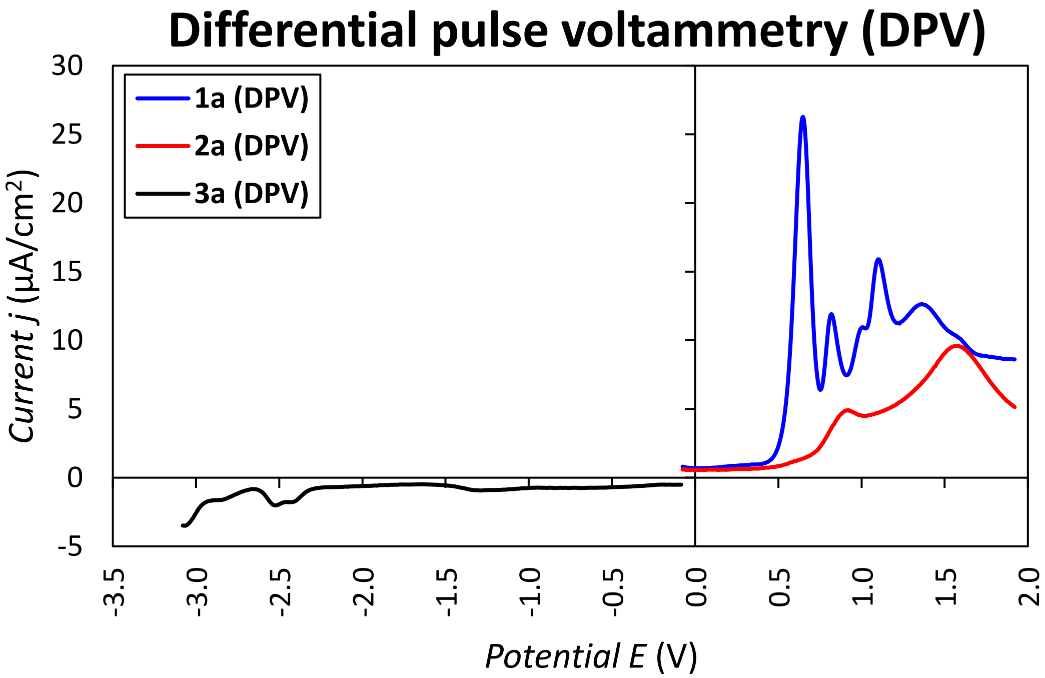

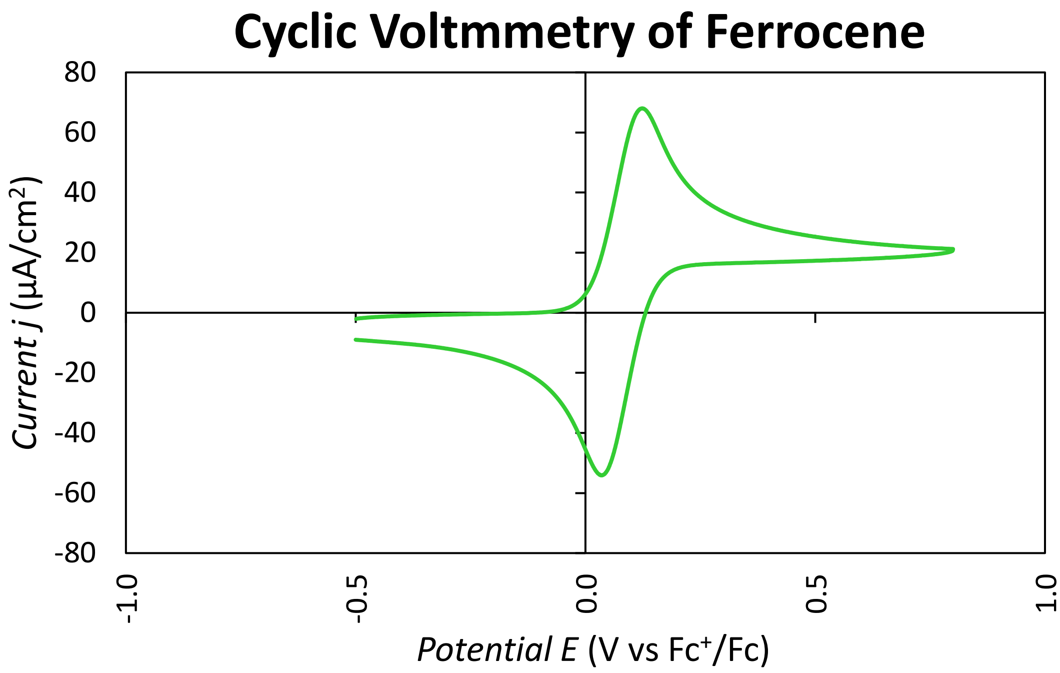

Uncorrected potential for ferrocene = 0.0795 V

In our cyclic voltammetry (CV) measurements, we used the **Fc^+/Fc** redox couple as a reference. To standardize the reported redox potentials, all measured values of compounds **1a**, **2**, and **3** were subsequently corrected by setting the Fc^+/Fc potential to zero. This adjustment ensured that the redox potentials of all other species were recalibrated accordingly, allowing for direct comparison with literature values referenced to the same standard.

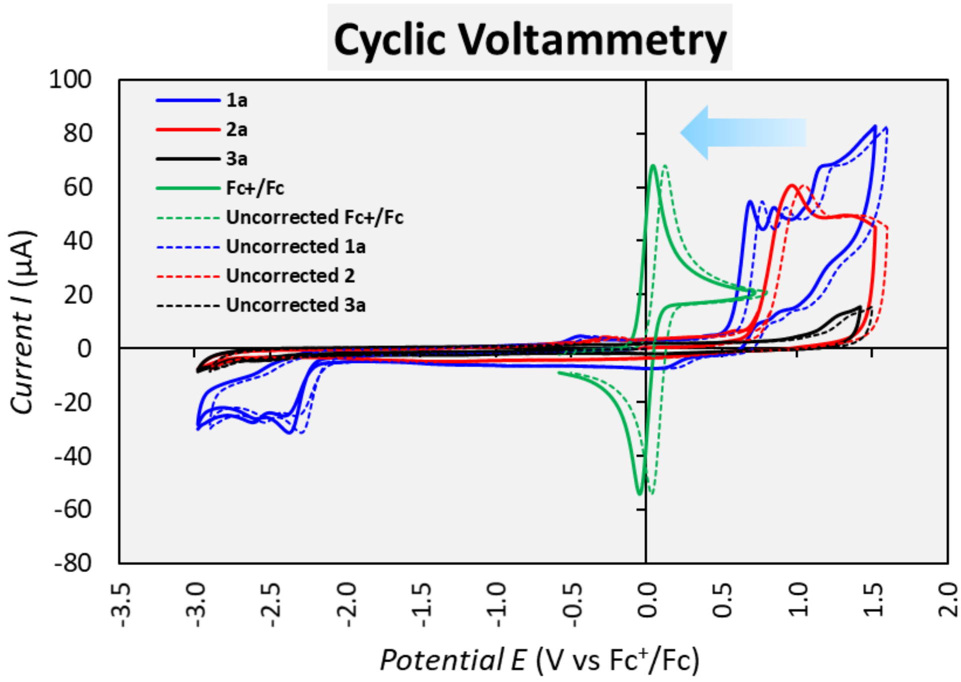

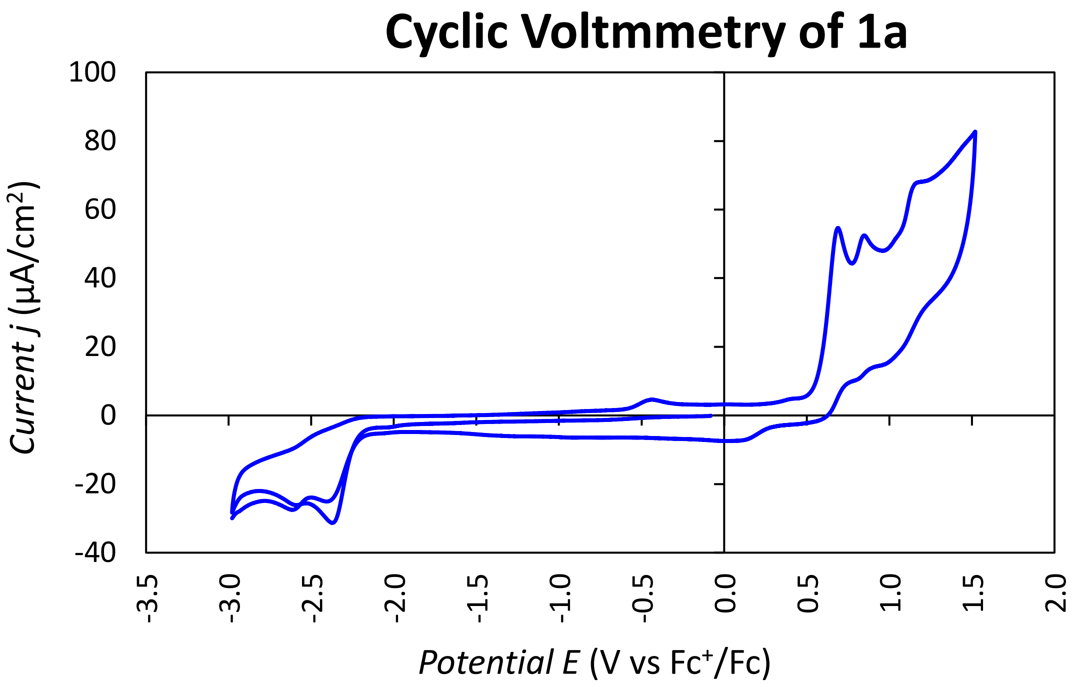

Oxidation potential of **1a** vs Fc^+/Fc = 0.6485 V.

Oxidation potential of **1a** vs SCE = 1.0285 V.

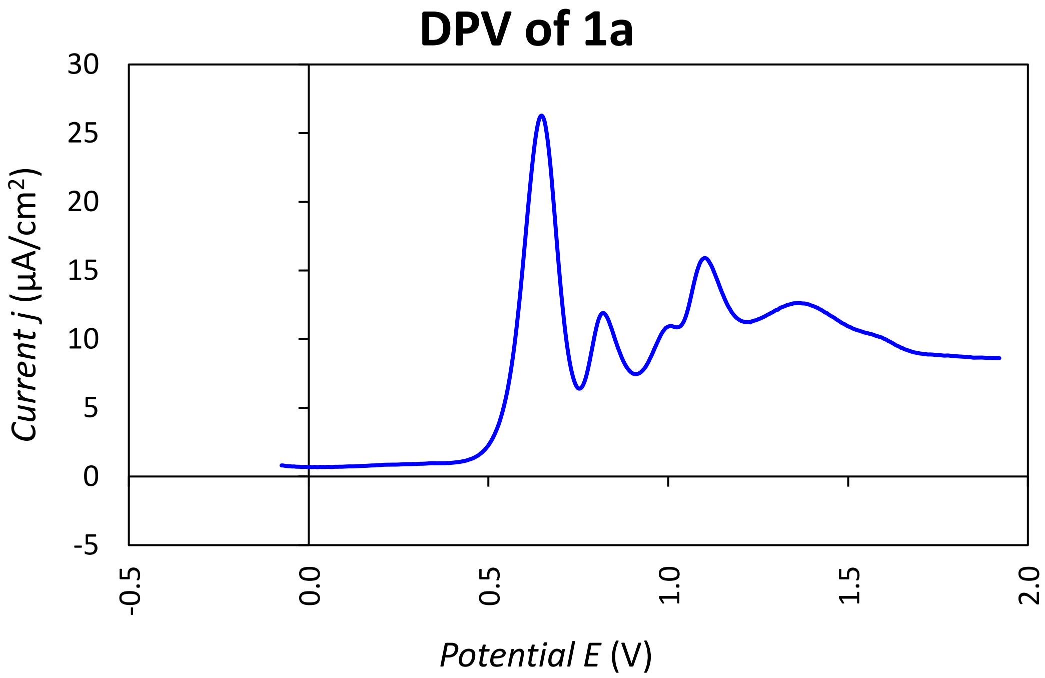

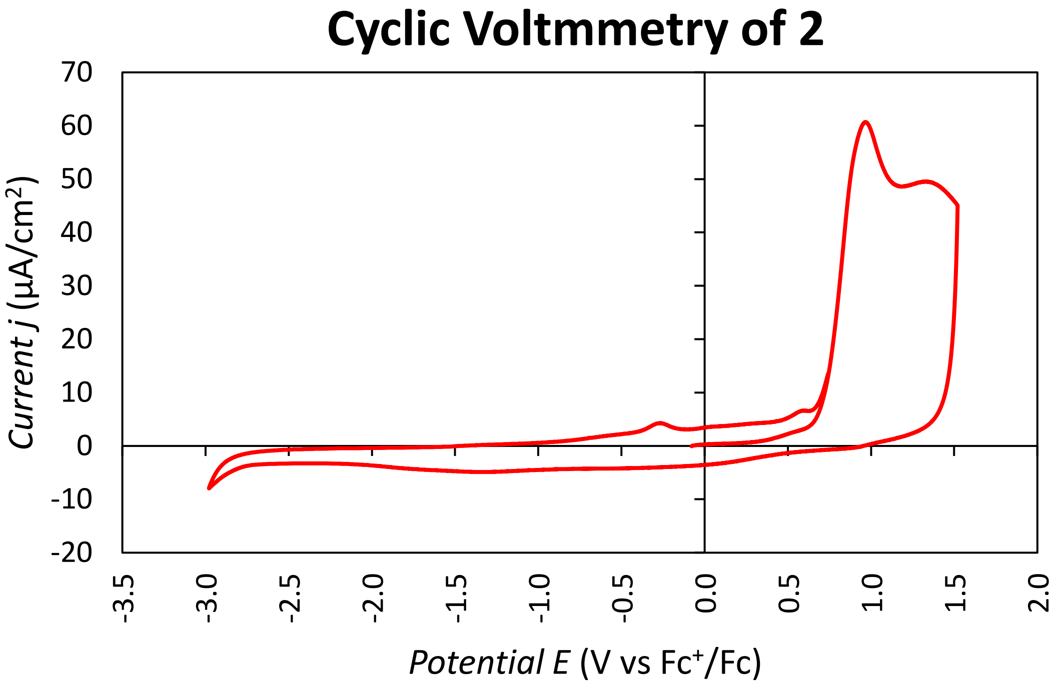

Oxidation potential of **2** vs Fc^+/Fc = 0.9205 V.

Oxidation potential of **2** vs SCE = 1.3005 V.

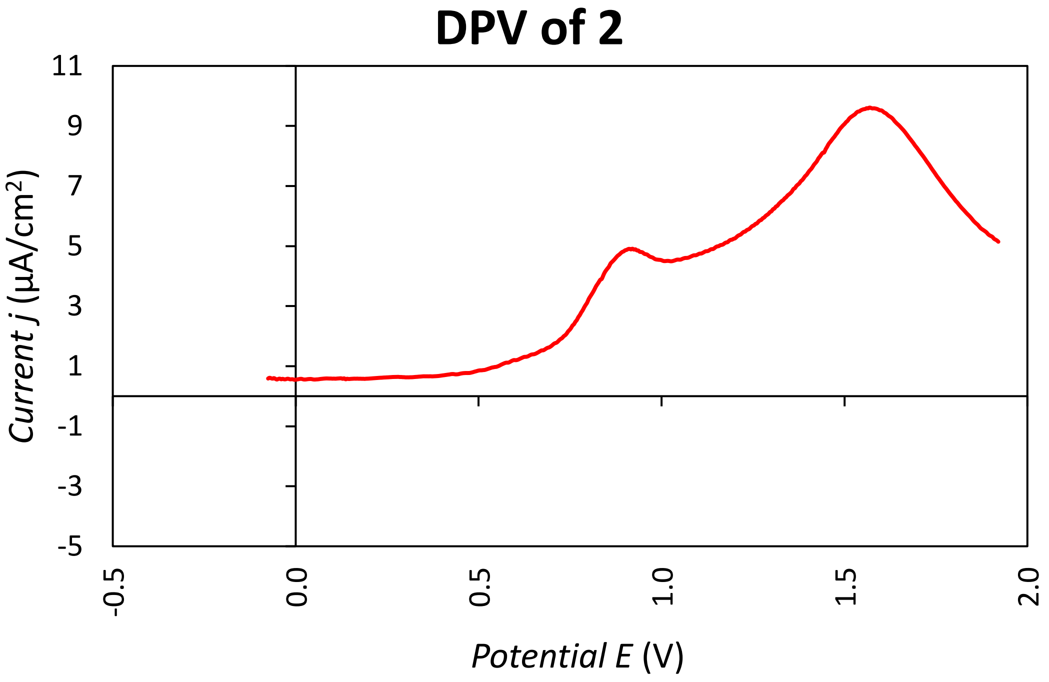

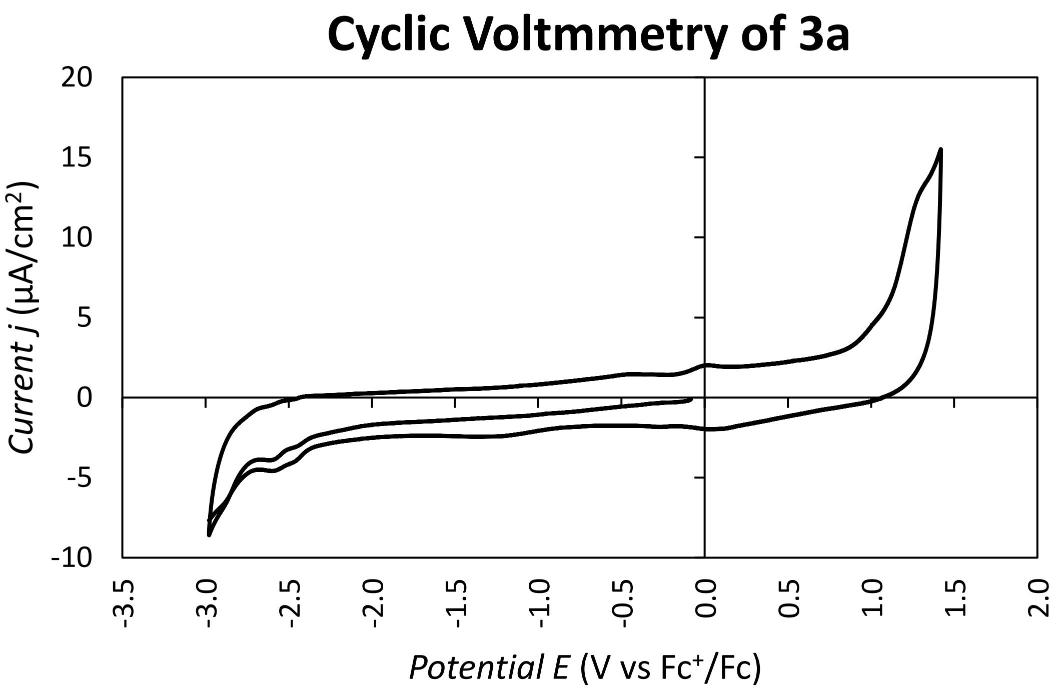

Reduction potential of **3a** vs Fc^+/Fc = −2.4315, −2.52, −2.84 V.

Reduction potential of **3a** vs SCE = −2.0515, −2.14, −2.46 V.

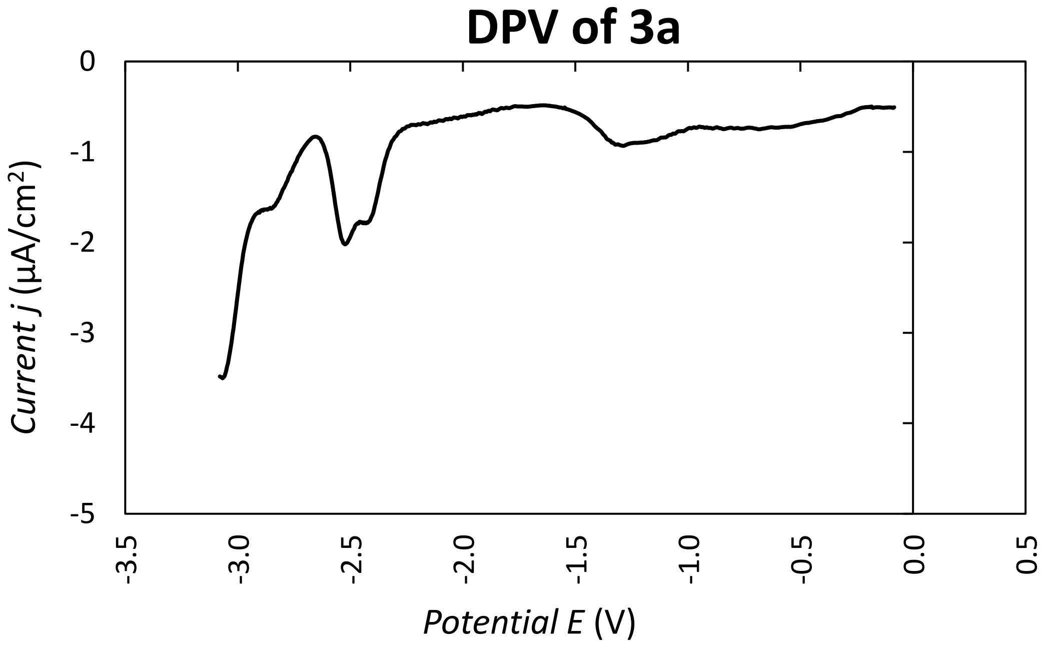

16. **Supplementary Note 9:** *calculation of band gap energy from UV–Vis absorption (Tauc Plots)*

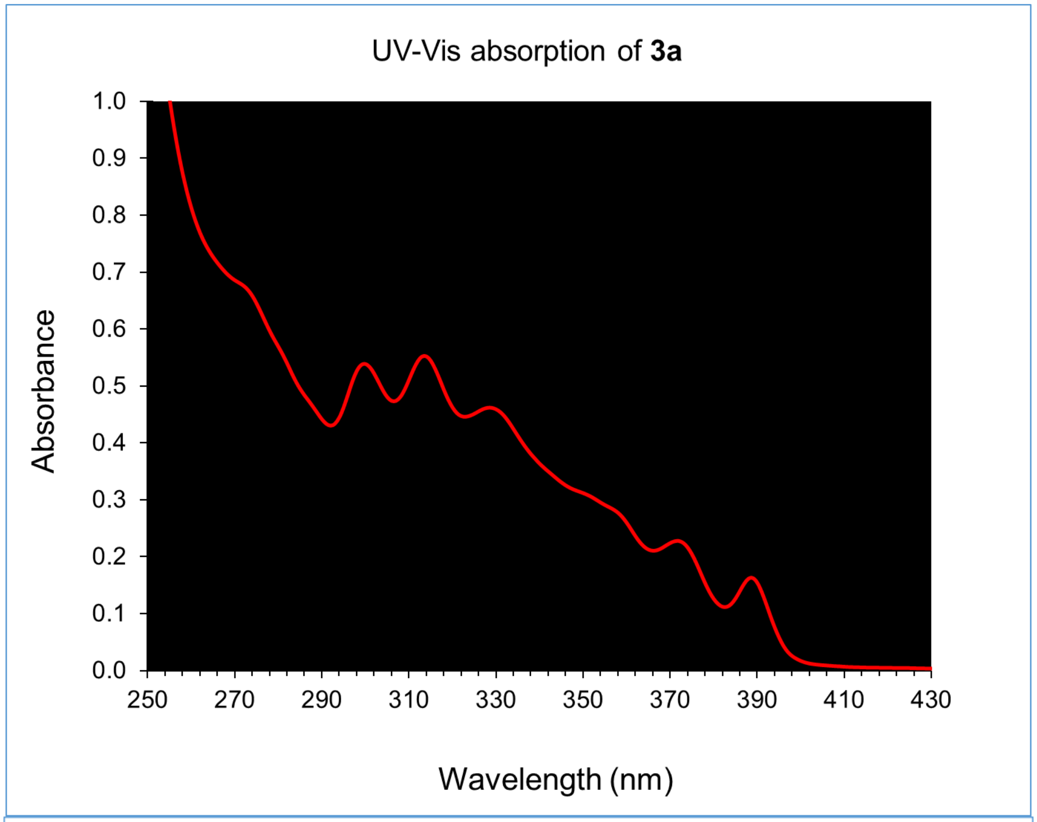

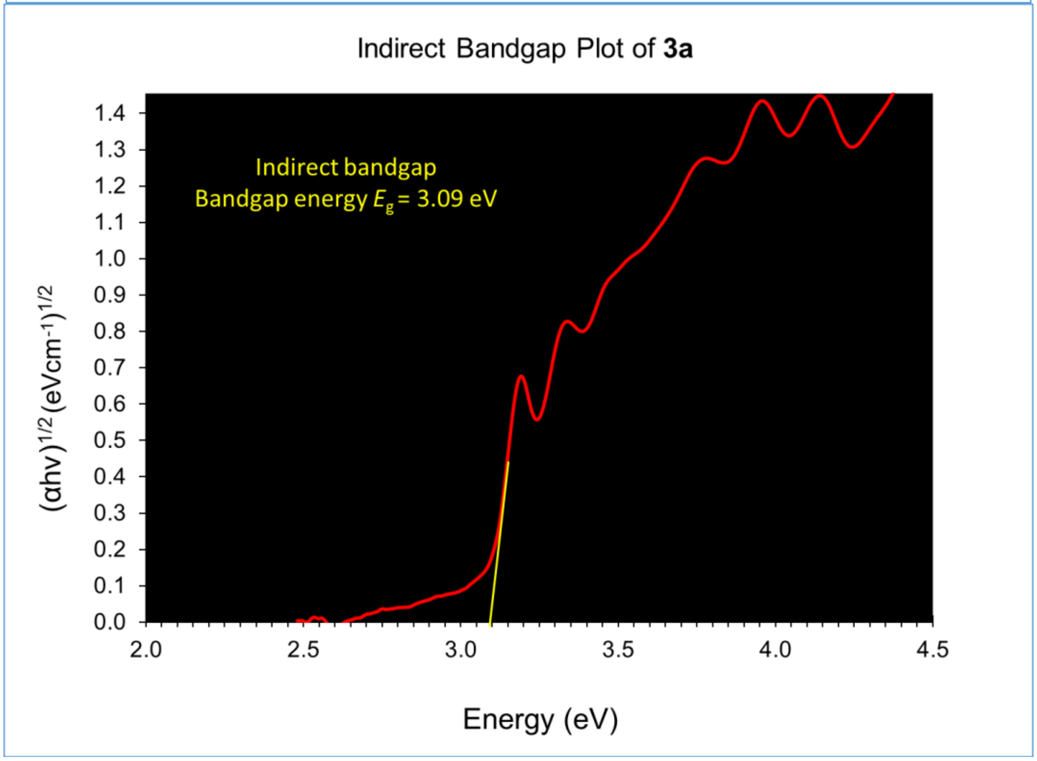

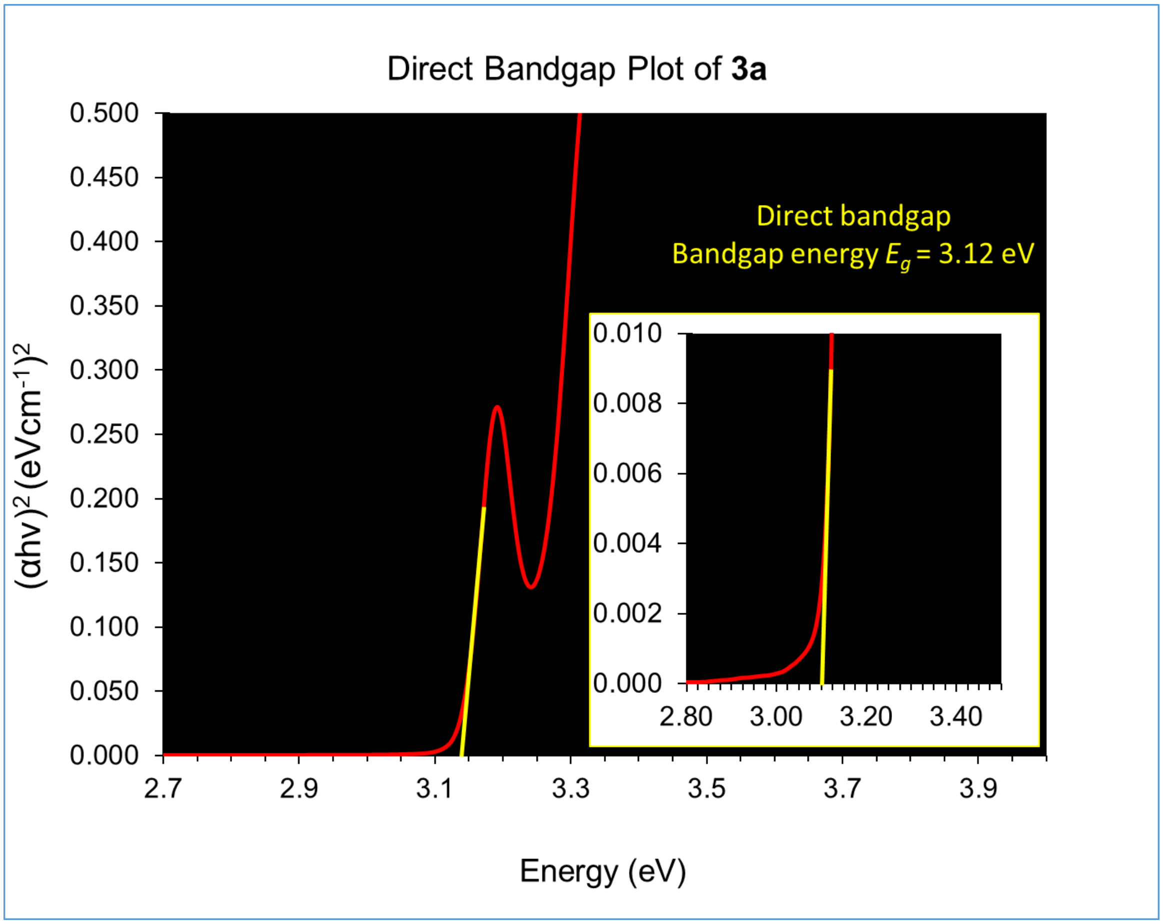

Direct Bandgap Plot of 3a
(αhv)2(eVcm-1)2
Energy (eV)
Direct bandgap
Bandgap energy Eg = 3.12 eV
0.500
0.450
0.400
0.350
0.300
0.250
0.200
0.150
0.100
0.050
0.000
2.7
2.9
3.1
3.3
3.5
3.7
3.9
0.010
0.008
0.006
0.004
0.002
0.000
2.80
3.00
3.20
3.40

17. **Supplementary Note 10:** *structural features of previously reported hetero[8]circulenes (Types I – IV) and hetero[9, 10]circulenes*

16.1. Alternating bond lengths of reported hetero[*n*]circulenes (comparative study)

- Hetero[8]circulenes (**Type I**)

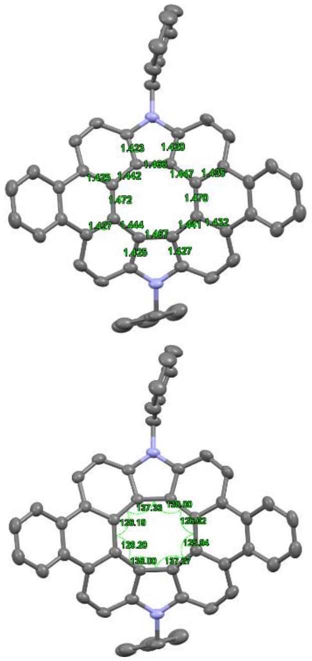

Maeda *et al.*, *Chem. Eur. J*, **2021**, *27*, 15699[69].

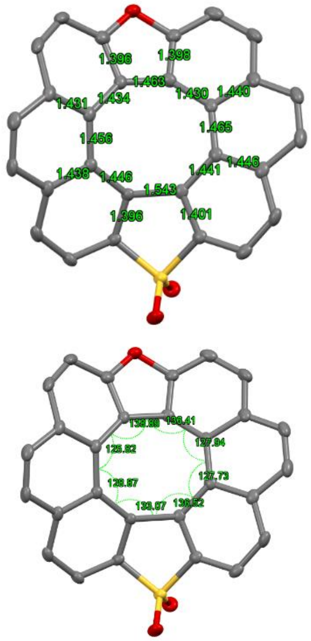

Maeda *et al.*, *Org. Lett.* **2023**, *25*, 3932[70].

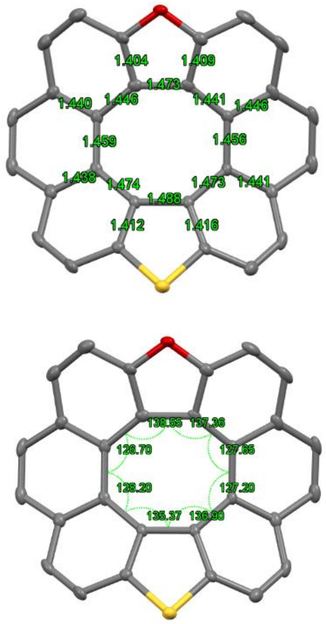

Maeda *et al.*, *Org. Lett.* **2023**, *25*, 3932[70].

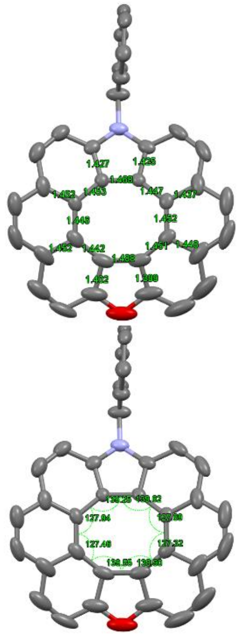

Maeda *et al.*, *Org. Lett.* **2023**, *25*, 3932[70].

- Hetero[8]circulenes (**Type II**)

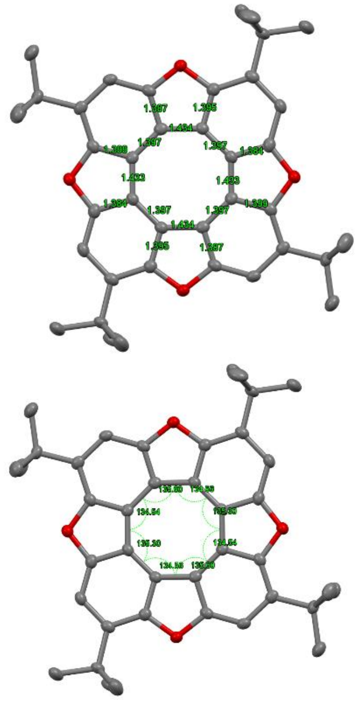

Brock-Nannestad *et al.*, *EJOC*, **2011**, *2011*, 6320[71].

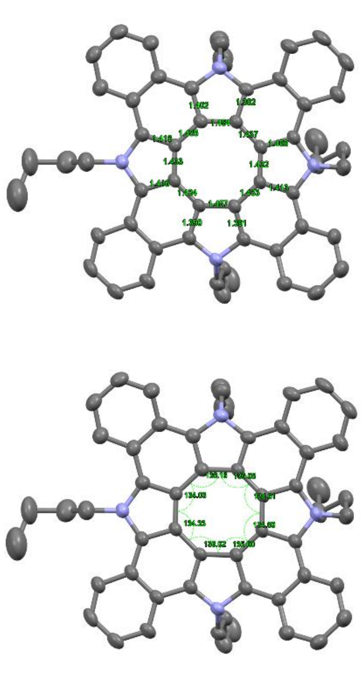

Chen *et al.*, *ACIE*, **2015**, *54*, 10639[72].

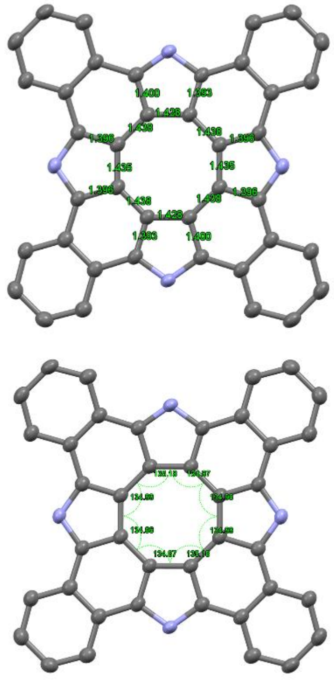

Chen *et al.*, *ACIE*, **2015**, *54*, 10639[72].

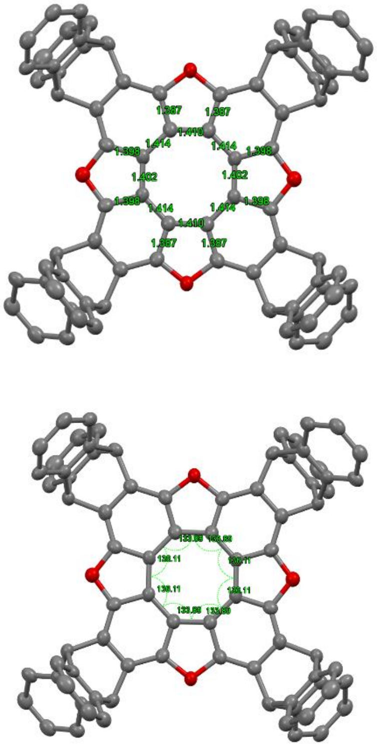

Nino *et al.*, *JACS*, **2021**, *144*, 556[73].

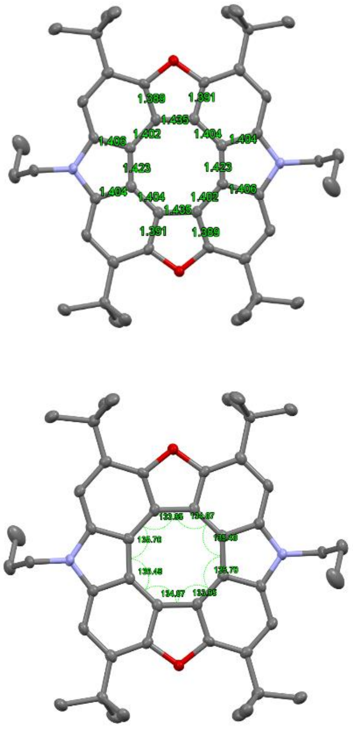

Hensel *et al.*, *Chem. Eur. J*, **2013**, *19*, 17097[74].

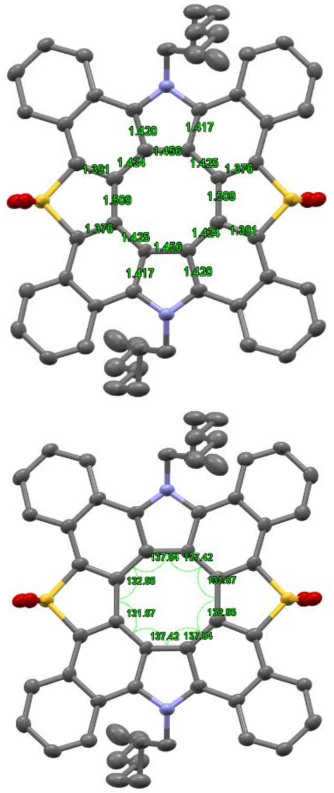

Matsuo *et al.*, *Chem. Eur. J*, **2020**, *26*, 8144[75].

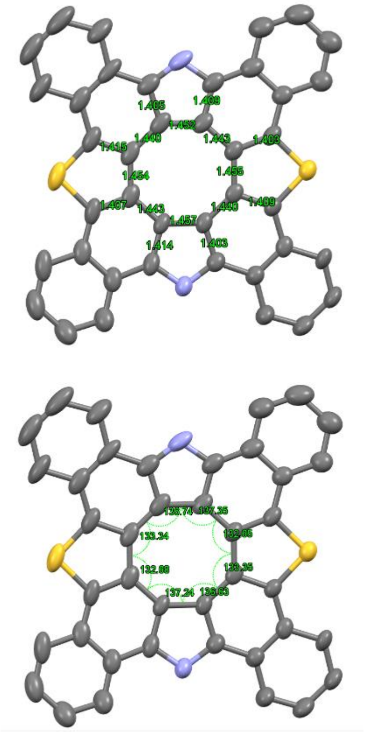

Matsuo *et al.*, *Chem. Eur. J*, **2020**, *26*, 8144[75].

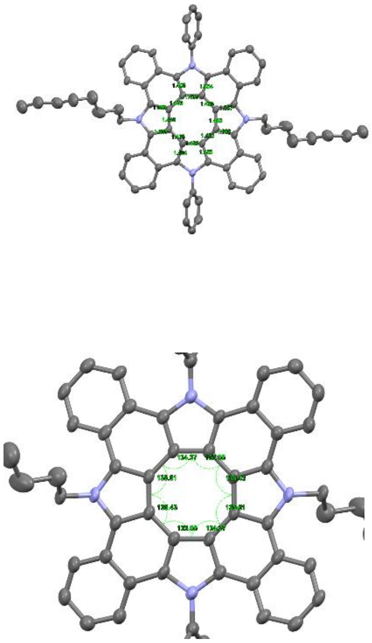

Matsuo *et al.*, *Chem. Eur. J*, **2020**, *26*, 8144[75].

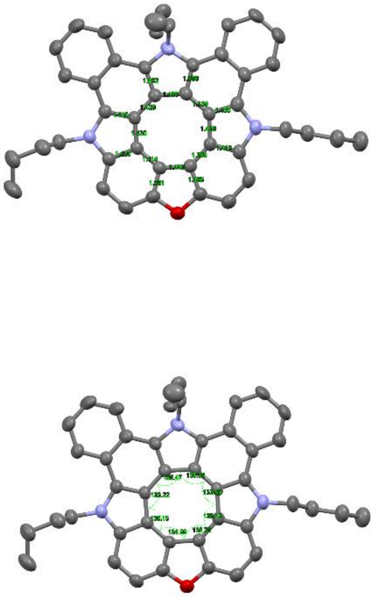

Matsuo *et al.*, *Chem. Sci.* **2019**, *10*, 11006[76].

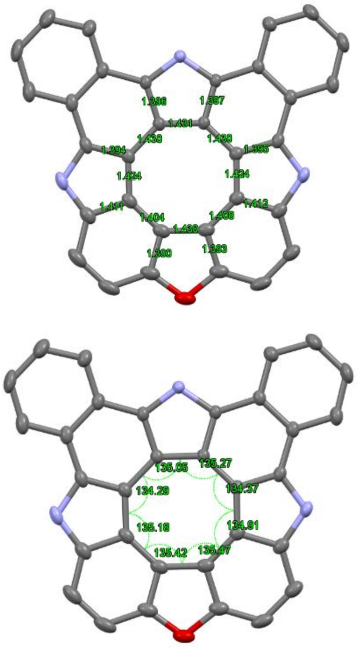

Matsuo *et al.*, *Chem. Sci.* **2019**, *10*, 11006[76].

- Hetero[8]circulenes (**Type III**)

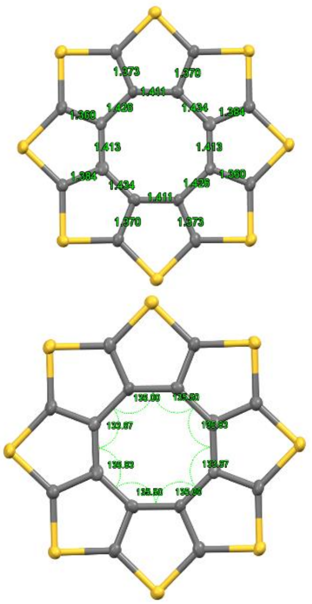

Fujimoto *et al.*, *Chem. Eur. J*, **2008**, *14*, 6053[77].

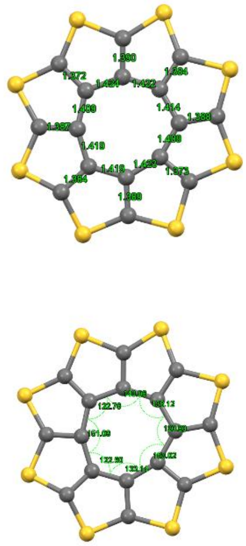

Chernichenko *et al.*, *ACIE*, **2006**, *45*, 7367[78].

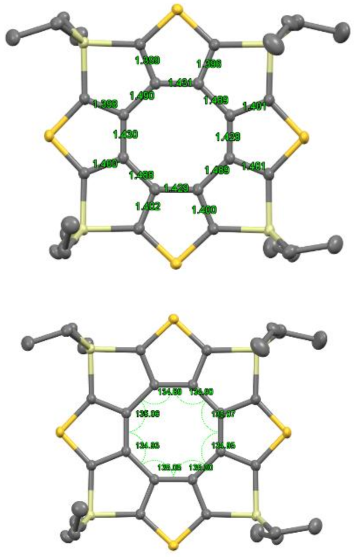

Serizawa *et al.*, *Chem. Eur. J*, **2017**, *23*, 6948[79].

- Hetero[8]circulenes (**Type IV**)

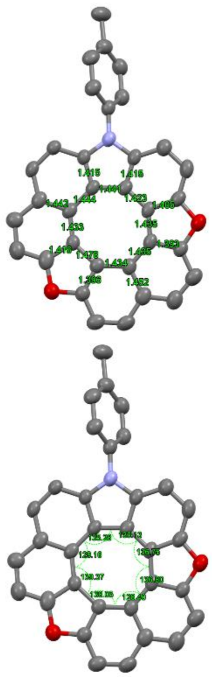

This work

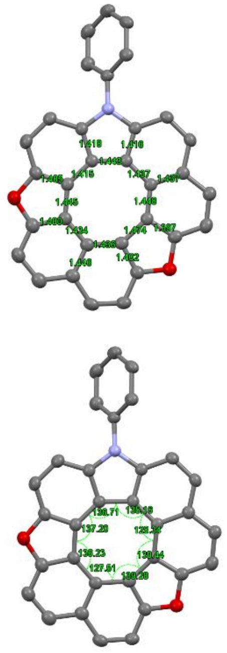

This work

- Hetero[9, 10]circulenes

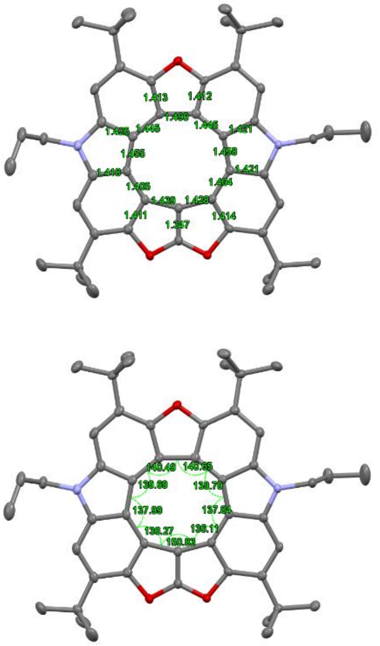

Pedersen *et al.*, *JACS*, **2020**, *142*, 14058[80].

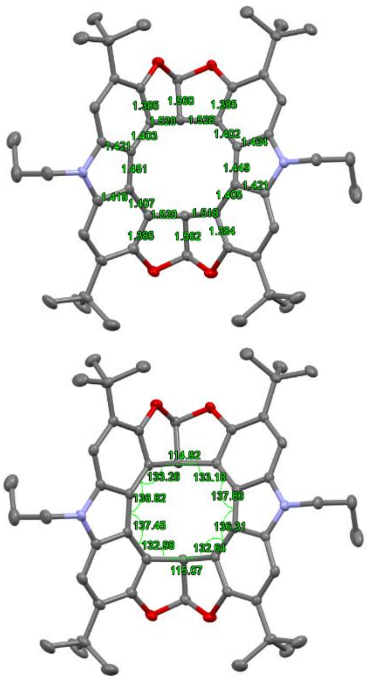

Pedersen *et al.*, *JACS*, **2020**, *142*, 14058[80].

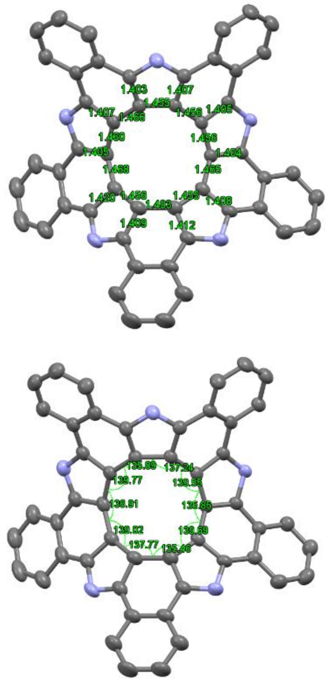

Matsuo *et al.*, *ACIE*, **2022**, *61*, e202116789[81].

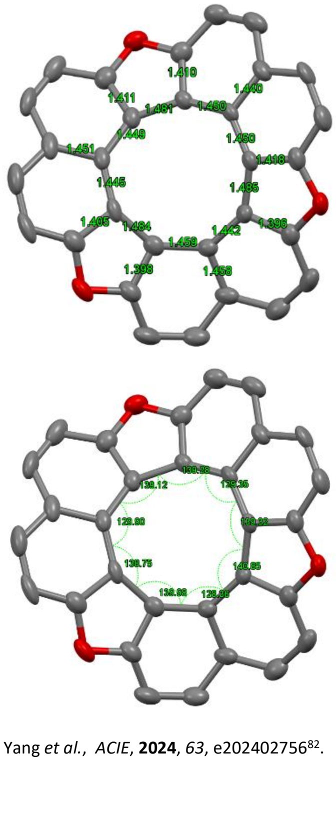

Yang *et al.*, *ACIE*, **2024**, *63*, e202402756[82].

16.2. Aromaticity of reported hetero[*n*]circulenes (comparative study)

- Hetero[8]circulene (**Type I**)

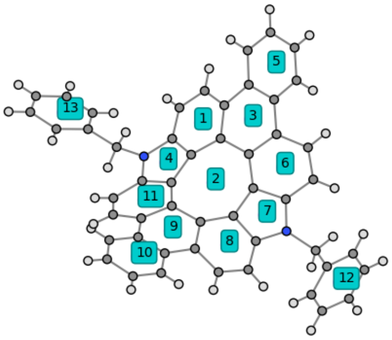

Maeda *et al.*, *Chem. Eur. J*, **2021**, *27*, 15699[69].

Ring	NICS(0)$_{iso}$	NICS(0)$_{zz}$	NICS(1)$_{iso}$	NICS(1)$_{zz}$	NICS(−1)$_{iso}$	NICS(−1)$_{zz}$
1	−5.3907	−6.8579	−8.2865	−5.9859	−7.9803	−0.5994
2	8.5546	2.908	4.5599	2.4557	5.9283	2.7913
3	−2.4258	−2.9068	−7.6668	−11.049	−5.2229	−5.4894
4	−7.925	−14.3954	−6.8861	−2.5417	−10.2459	−3.2823
5	−6.2944	−4.5482	−10.6629	−10.7343	−9.4723	−12.3422
6	−5.4286	0.0078	−8.5595	−11.4484	−8.0284	−11.5177
7	−8.06	−5.8615	−7.1656	−6.1246	−10.6786	−12.8617
8	−5.4145	−8.2925	−8.1377	−4.2906	−8.2876	−9.1227
9	−2.352	−8.3386	−7.7469	−3.4373	−5.2281	−4.0596
10	−6.3702	−9.8522	−9.6014	−3.6365	−10.7036	−4.0038
11	−5.5248	−12.3362	−10.6314	−5.198	−10.2736	−7.7658
12	−6.6623	−4.2926	−10.4519	−13.8078	−10.6942	−14.2572
13	−6.3011	−3.7177	−10.2682	−20.9615	−10.2838	−20.754

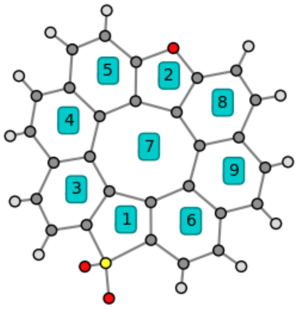

Maeda *et al.*, *Org. Lett.* **2023**, *25*, 3932[70].

Ring	NICS(0)$_{iso}$	NICS(0)$_{zz}$	NICS(1)$_{iso}$	NICS(1)$_{zz}$	NICS(−1)$_{iso}$	NICS(−1)$_{zz}$
1	1.5901	−10.8714	0.916	−5.4761	−1.6899	−2.7859
2	−7.3853	−19.7967	−6.5459	−6.79	−9.7605	−3.8555
3	−6.5254	−9.8887	−10.2016	−7.7217	−8.7712	−1.646
4	−5.1974	−8.7631	−9.6245	−7.3555	−8.0324	0.617
5	−6.4883	−10.2054	−8.1825	−6.6889	−9.8686	−3.5992
6	−5.8804	−7.9203	−8.4043	−8.5586	−9.2405	−5.3842
7	7.4587	−8.0715	1.4166	−13.4153	4.5362	2.1146
8	−6.45	−9.1712	−9.6576	−7.7486	−8.6534	−0.6015
9	−4.6894	−8.9931	−9.8403	−9.0931	−7.1624	0.7047

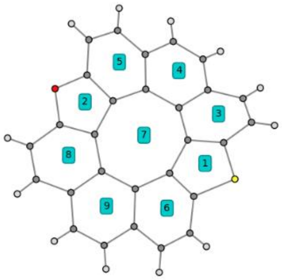

Maeda *et al.*, *Org. Lett.* **2023**, *25*, 3932[70].

Ring	NICS(0)$_{iso}$	NICS(0)$_{zz}$	NICS(1)$_{iso}$	NICS(1)$_{zz}$	NICS(−1)$_{iso}$	NICS(−1)$_{zz}$
1	−6.6604	−14.2589	−9.165	−6.7938	−5.0286	−1.1587
2	−7.2047	−14.1131	−6.0516	−3.0341	−9.2218	−8.932
3	−5.25	−8.5543	−8.1464	−7.5263	−8.729	−5.3453
4	−5.9708	−10.1294	−10.4011	−5.9058	−8.0199	−4.8401
5	−6.6753	−11.8845	−9.0701	−3.5003	−9.6247	−6.3222
6	−5.2257	−9.1968	−8.4937	−5.3778	−8.1963	−2.2916
7	7.5005	−5.357	4.2975	−1.4577	4.8009	−5.5766
8	−6.6104	−8.5592	−9.0036	−5.0395	−9.0786	−8.3716
9	−5.936	−9.734	−8.1361	−5.6588	−10.4166	−6.728

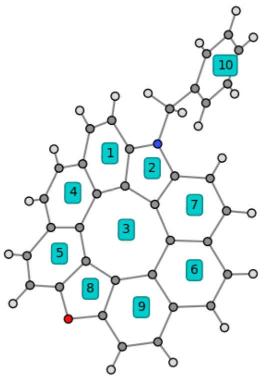

Maeda *et al.*, *Org. Lett.* **2023**, *25*, 3932[70].

Ring	NICS(0)$_{iso}$	NICS(0)$_{zz}$	NICS(1)$_{iso}$	NICS(1)$_{zz}$	NICS(−1)$_{iso}$	NICS(−1)$_{zz}$
1	−6.4576	−5.6483	−9.0282	−14.2077	−9.0345	−8.2188
2	−8.3337	3.1525	−9.9621	−15.7195	−7.826	−10.5423
3	7.8226	22.1877	5.1956	11.4357	4.5973	13.7839
4	−5.9709	−4.2248	−8.4662	−11.9758	−10.1032	−5.0238
5	−6.9901	−7.3038	−9.236	−11.1024	−9.3469	−8.6397
6	−6.4967	−1.9556	−8.7242	−16.4891	−10.5019	−22.1038
7	−6.151	1.7832	−8.7137	−15.3402	−8.8255	−16.6044
8	−6.9868	2.1683	−8.8408	−9.9223	−6.2226	−7.452
9	−6.7394	−1.5486	−9.2224	−13.9332	−9.0112	−16.4549
10	−6.2218	−5.8112	−10.1518	−8.7665	−10.2618	−9.6009

- Hetero[8]circulene (Type II)

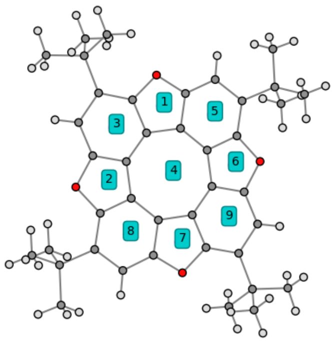

Brock–Nannestad *et al.*, *EJOC*, **2011**, *2011*, 6320[71].

Ring	NICS(0)$_{iso}$	NICS(0)$_{zz}$	NICS(1)$_{iso}$	NICS(1)$_{zz}$	NICS(−1)$_{iso}$	NICS(−1)$_{zz}$
1	−7.0441	4.9746	−7.1743	−11.1748	−7.2208	−8.2161
2	−7.0088	7.5398	−7.1834	−10.1324	−7.1154	−8.6204
3	−8.156	−2.5774	−9.326	−15.323	−9.2957	−13.1832
4	8.0194	27.2181	5.1684	15.3285	5.1636	15.3186
5	−8.1223	−1.5636	−9.3292	−13.559	−9.2219	−14.0741
6	−6.9788	7.6066	−7.1331	−8.638	−7.1531	−10.082
7	−7.032	5.0219	−7.2341	−8.2314	−7.1479	−11.1085
8	−8.1106	−1.5319	−9.2109	−14.0644	−9.3287	−13.5382
9	−8.1698	−2.6041	−9.2848	−13.1598	−9.3463	−15.3598

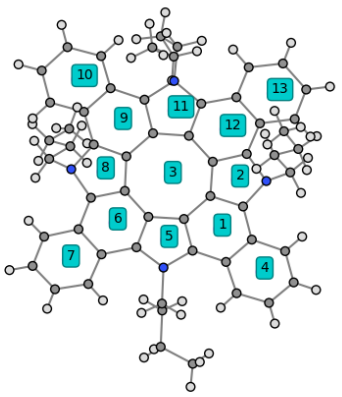

Chen *et al.*, *ACIE*, **2015**, *54*, 10639[72].

Ring	NICS(0)$_{iso}$	NICS(0)$_{zz}$	NICS(1)$_{iso}$	NICS(1)$_{zz}$	NICS(−1)$_{iso}$	NICS(−1)$_{zz}$
1	−6.2657	1.3498	−7.8104	−11.9632	−8.5587	−12.8956
2	−5.2691	11.5903	−6.981	−10.2226	−6.4372	−9.0367
3	6.39	23.763	4.0369	12.8278	3.7868	12.4765
4	−6.617	−3.6224	−9.7332	−16.3045	−10.197	−20.22
5	−9.8533	1.0072	−9.6136	−15.0071	−9.9004	−18.0562
6	−6.0106	1.3608	−7.7122	−12.681	−8.075	−12.4449
7	−6.7102	−3.4316	−9.7892	−17.8952	−10.2741	−20.9853
8	−5.8304	8.9059	−7.403	−11.9194	−6.6851	−9.5635
9	−5.7147	1.8469	−6.9471	−10.9527	−8.6647	−13.427
10	−6.5938	−1.0238	−10.4619	−18.0717	−9.3179	−18.1793
11	−7.4212	0.1974	−7.8636	−18.068	−8.1174	−10.6471
12	−5.8722	2.3447	−7.1497	−11.2967	−8.6735	−13.8907
13	−6.5799	−0.8859	−9.2091	−17.9066	−10.557	−19.399

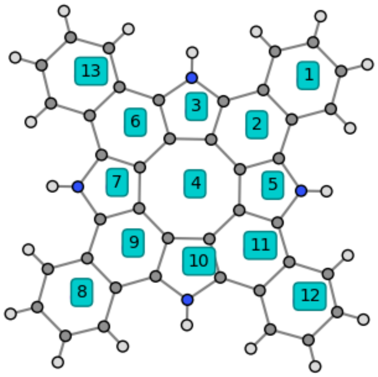

Chen *et al.*, *ACIE*, **2015**, *54*, 10639[72].

Ring	NICS(0)$_{iso}$	NICS(0)$_{zz}$	NICS(1)$_{iso}$	NICS(1)$_{zz}$	NICS(−1)$_{iso}$	NICS(−1)$_{zz}$
1	−6.8397	−8.9787	−10.2051	−7.4804	−9.9466	−4.7106
2	−6.0435	−10.075	−8.0306	−6.5557	−7.7214	−5.4917
3	−8.7695	−18.4597	−8.8547	−7.5862	−8.8713	−5.5116
4	6.5382	−1.8607	4.0631	−0.2049	4.0788	−0.2021
5	−8.9267	−11.5113	−8.9683	−6.4269	−8.9719	−6.2049
6	−6.0478	−10.1066	−7.778	−6.6738	−7.9157	−5.5901
7	−8.8227	−11.546	−8.9575	−6.2161	−8.8731	−6.4299
8	−6.8086	−8.9838	−9.9241	−4.6911	−10.1987	−7.5032
9	−6.0721	−10.065	−7.7253	−5.4702	−8.0481	−6.5638
10	−8.8403	−18.488	−8.8794	−5.4861	−8.8738	−7.6093
11	−6.0501	−10.1091	−7.8894	−5.5973	−7.8116	−6.6703
12	−6.919	−8.745	−10.1679	−4.7329	−10.1186	−8.0058
13	−6.9204	−8.7358	−10.0953	−7.9994	−10.1904	−4.7297

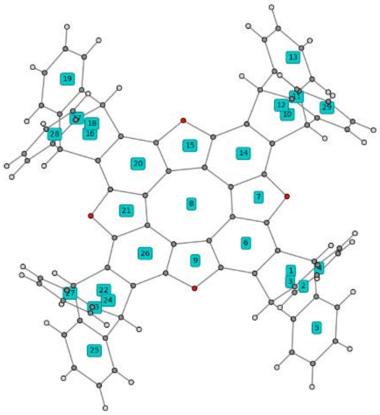

Nino *et al.*, *JACS*, **2021**, *144*, 556[73].

Ring	NICS(0)$_{iso}$	NICS(0)$_{zz}$	NICS(1)$_{iso}$	NICS(1)$_{zz}$	NICS(−1)$_{iso}$	NICS(−1)$_{zz}$
1	3.8625	−3.4565	−2.0697	7.7965	−7.0241	−8.8135
2	4.1281	1.2141	−3.556	−2.0308	−0.016	1.6626
3	3.5203	6.5513	−4.2102	3.4232	1.4521	−4.7418
4	−6.4912	−9.9831	−10.1584	−5.367	−10.2516	−5.0332
5	−6.7389	−3.7841	−10.23	−11.1775	−10.6124	−11.3285
6	−8.025	−8.924	−9.2454	−7.4524	−8.6578	−8.5926
7	−7.4459	−6.8957	−7.4072	−7.9394	−7.4309	−7.9328
8	8.9755	4.387	5.8016	3.2592	5.8287	3.255
9	−7.5511	−12.3001	−7.5605	−4.3438	−7.2684	−6.7794
10	3.7847	−3.409	−7.128	−8.7879	−1.9037	7.7929
11	3.8491	1.1984	0.0946	1.7001	−3.7621	−2.0492
12	3.5812	6.5472	1.1856	−4.727	−3.9978	3.4056
13	−6.7425	−3.7941	−10.5953	−11.3388	−10.2576	−11.1854
14	−8.0043	−8.9133	−8.632	−8.5862	−9.2282	−7.4518
15	−7.6006	−12.2995	−7.2657	−6.7727	−7.5866	−4.3588
16	3.7847	−3.409	−7.128	−8.7879	−1.9037	7.7929
17	3.8491	1.1984	0.0946	1.7001	−3.7621	−2.0492
18	3.5812	6.5472	1.1856	−4.727	−3.9978	3.4056
19	−6.7425	−3.7941	−10.5953	−11.3388	−10.2576	−11.1854
20	−8.0043	−8.9133	−8.632	−8.5862	−9.2282	−7.4518
21	−7.4459	−6.8957	−7.4072	−7.9394	−7.4309	−7.9328
22	3.8625	−3.4565	−2.0697	7.7965	−7.0241	−8.8135
23	4.1281	1.2141	−3.556	−2.0308	−0.016	1.6626
24	3.5203	6.5513	−4.2102	3.4232	1.4521	−4.7418
25	−6.7389	−3.7841	−10.23	−11.1775	−10.6124	−11.3285
26	−8.025	−8.924	−9.2454	−7.4524	−8.6578	−8.5927
27	−6.4912	−9.9831	−10.1584	−5.367	−10.2516	−5.0332

**28**	−6.5424	−9.9777	−10.2826	−5.0261	−10.2137	−5.3636
**29**	−6.5424	−9.9777	−10.2826	−5.0261	−10.2137	−5.3636

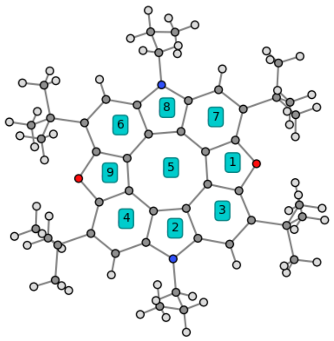

Hensel *et al.*, *Chem. Eur. J*, **2013**, *19*, 17097[74].

Ring	NICS(0)iso	NICS(0)zz	NICS(1)iso	NICS(1)zz	NICS(−1)iso	NICS(−1)zz
**1**	−6.4576	−5.6483	−7.1693	−6.3774	−7.2915	−6.2669
**2**	−8.3337	3.1525	−9.0018	−6.7146	−8.833	−9.2275
**3**	7.8226	22.1877	−9.5628	−6.1888	−9.2597	−6.9197
**4**	−5.9709	−4.2248	−9.2944	−6.0347	−9.2099	−6.8222
**5**	−6.9901	−7.3038	5.2546	−0.8756	5.2293	−0.8723
**6**	−6.4967	−1.9556	−9.2477	−6.8877	−9.5251	−6.2092
**7**	−6.151	1.7832	−9.3004	−6.8466	−9.2496	−6.0148
**8**	−6.9868	2.1683	−8.7496	−9.2437	−8.993	−6.7125
**9**	−6.2218	−5.8112	−7.1509	−6.2203	−7.1833	−6.3701

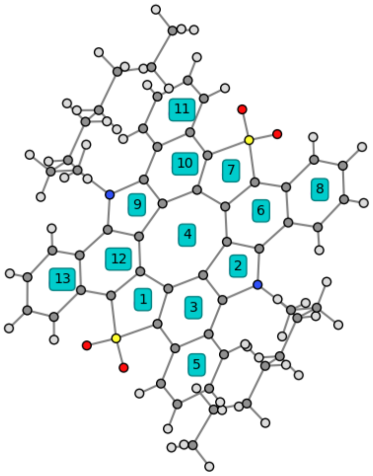

Matsuo *et al.*, *Chem. Eur. J*, **2020**, *26*, 8144[75].

Ring	NICS(0)iso	NICS(0)zz	NICS(1)iso	NICS(1)zz	NICS(−1)iso	NICS(−1)zz
**1**	1.3835	−17.2272	−1.9705	−3.4537	−3.6727	−9.9832
**2**	−6.9359	−16.5656	−8.0595	−0.9794	−8.0662	−5.6779
**3**	−6.485	−10.8195	−7.9068	−0.939	−8.8914	−4.7809
**4**	6.061	−9.0604	3.818	−4.4878	3.8122	−4.501
**5**	−6.0117	−8.5281	−9.1918	−3.2704	−10.2226	−2.8728
**6**	−7.0102	−16.2951	−9.7042	−9.0983	−10.2522	−6.6414
**7**	1.3027	−17.2423	−3.7247	−9.9875	−1.9925	−3.4596
**8**	−6.2063	−9.9959	−9.5863	−3.9385	−10.1572	−3.3173
**9**	−6.9871	−16.5928	−8.074	−5.6788	−8.1141	−1.0111
**10**	−6.555	−10.863	−8.9725	−4.8528	−7.9144	−0.9116
**11**	−5.9956	−8.5409	−10.2364	−2.8836	−9.1748	−3.269
**12**	−6.9997	−16.2934	−10.2493	−6.6468	−9.6809	−9.1097
**13**	−6.1981	−9.9808	−10.1561	−3.3226	−9.5793	−3.9206

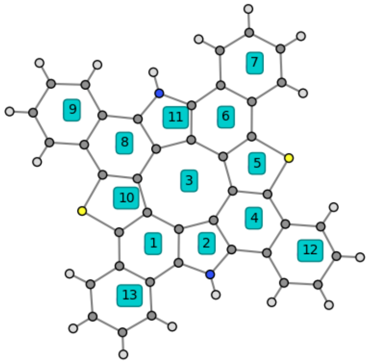

Matsuo *et al.*, *Chem. Eur. J*, **2020**, *26*, 8144[75].

Ring	NICS(0)iso	NICS(0)zz	NICS(1)iso	NICS(1)zz	NICS(−1)iso	NICS(−1)zz
**1**	−5.9522	−11.4298	−8.0346	−3.7566	−7.9814	−4.0453
**2**	−8.9341	−22.8358	−8.9985	−4.195	−9.0359	−4.956
**3**	6.2737	−8.7541	3.7922	−3.9907	3.8604	−3.7603
**4**	−5.9294	−13.4067	−8.1889	−5.3959	−7.7658	−5.2946
**5**	−6.3103	−15.3065	−6.2679	−3.6852	−6.0897	−3.5175
**6**	−5.887	−11.4248	−7.9661	−4.1273	−7.9373	−3.6229
**7**	−6.3621	−10.3325	−9.8895	−4.7408	−9.8054	−3.2734
**8**	−5.789	−13.3356	−7.7529	−5.4573	−7.9454	−5.237
**9**	−6.6147	−10.0349	−9.9361	−4.2084	−10.1302	−3.4466
**10**	−6.3761	−15.1966	−6.1997	−3.6721	−6.2306	−3.6667
**11**	−8.8686	−22.9051	−8.9066	−5.1251	−8.9768	−4.0453
**12**	−6.4111	−10.08	−9.984	−3.6129	−9.6966	−4.4309

13	−6.4748	−10.2331	−9.9022	−3.4415	−9.8925	−4.9135

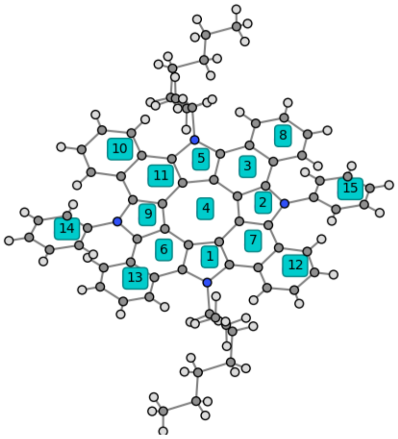

Matsuo *et al.*, *Chem. Eur. J*, **2020**, *26*, 8144[75].

Ring	$NICS(0)_{iso}$	$NICS(0)_{zz}$	$NICS(1)_{iso}$	$NICS(1)_{zz}$	$NICS(-1)_{iso}$	$NICS(-1)_{zz}$
1	−6.2845	−7.0818	−7.1333	−15.8528	−7.257	−6.6383
2	−7.9621	−0.7657	−8.172	−11.1618	−8.1295	−11.3269
3	−5.867	−4.0562	−8.5413	−11.7378	−6.8081	−10.7456
4	6.5636	12.2736	4.0729	6.23	4.2014	6.2605
5	−5.8019	−6.8977	−7.1363	−6.6563	−6.9638	−15.7502
6	−5.8833	−3.9495	−6.6925	−10.6955	−8.705	−11.6862
7	−6.1554	−5.7738	−7.4412	−12.7592	−8.4102	−10.8645
8	−6.9266	−2.8033	−10.8052	−15.6768	−9.4798	−17.0193
9	−8.4146	−1.6004	−8.3597	−11.6585	−8.2367	−11.5506
10	−7.1008	−5.4961	−10.6616	−14.3881	−10.0124	−16.3282
11	−5.9623	−5.4184	−8.1265	−10.5263	−7.5031	−12.7599
12	−7.0398	−5.3676	−9.9144	−16.2764	−10.6989	−14.3417
13	−6.8821	−2.7575	−9.4444	−16.9852	−10.7978	−15.6569
14	−5.5493	−4.0293	−8.5559	−11.5471	−8.8463	−9.7523
15	−5.5703	−4.025	−8.87	−9.8455	−8.6005	−11.424

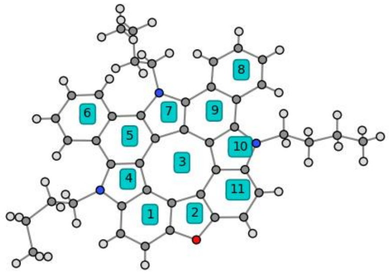

Matsuo *et al.*, *Chem. Sci.* **2019**, *10*, 11006[76].

Ring	$NICS(0)_{iso}$	$NICS(0)_{zz}$	$NICS(1)_{iso}$	$NICS(1)_{zz}$	$NICS(-1)_{iso}$	$NICS(-1)_{zz}$
1	−9.0834	−12.3492	−10.4746	−4.8302	−10.1154	−2.8988
2	−7.0238	−21.8472	−7.3924	−4.8876	−7.1008	−3.2212
3	6.9047	−6.9607	4.2229	−2.9915	4.312	−2.3024
4	−9.1922	−14.2387	−9.3848	−4.8014	−9.2596	−3.2474
5	−5.822	−12.641	−7.5795	−5.673	−7.7639	−5.4402
6	−6.7789	−9.8553	−9.9139	−2.5694	−10.1684	−4.6739
7	−7.186	−19.8763	−8.2518	−5.8791	−7.6603	−4.392
8	−6.8136	−9.8104	−9.6231	−2.2769	−10.5038	−4.3649
9	−5.7415	−11.6201	−7.1952	−3.0302	−8.0277	−4.894
10	−8.6604	−13.6005	−9.1259	−3.3325	−8.6952	−4.1885
11	−8.9983	−12.1467	−10.573	−5.2117	−9.986	−3.73

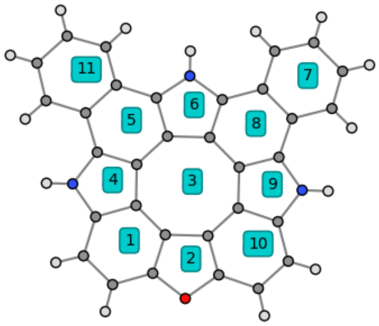

Matsuo *et al.*, *Chem. Sci.* **2019**, *10*, 11006[76].

Ring	$NICS(0)_{iso}$	$NICS(0)_{zz}$	$NICS(1)_{iso}$	$NICS(1)_{zz}$	$NICS(-1)_{iso}$	$NICS(-1)_{zz}$
1	−8.841	−1.4732	−10.0455	−21.8125	−10.1343	−20.1682
2	−7.0184	13.7126	−7.324	−13.737	−7.3369	−10.8285
3	6.9077	34.9058	4.2822	18.1855	4.2088	18.2972
4	−8.8285	10.8916	−8.9037	−17.4602	−9.0765	−17.8663
5	−5.8606	7.6125	−7.7052	−12.9614	−7.6653	−13.6061
6	−9.1319	10.0875	−9.092	−16.9967	−9.1957	−18.6449
7	−6.7121	−0.4796	−9.9826	−20.7133	−9.9937	−22.5728
8	−5.8968	7.4865	−7.6927	−13.0392	−7.7685	−13.7215
9	−8.7856	10.9473	−8.8597	−17.6798	−9.0235	−17.4241

**10**	−8.8694	−1.5311	−10.0715	−22.0372	−10.1722	−20.0974
**11**	−6.7381	−0.4383	−10.0408	−20.8156	−9.9861	−22.922

- Hetero[8]circulene (Type III)

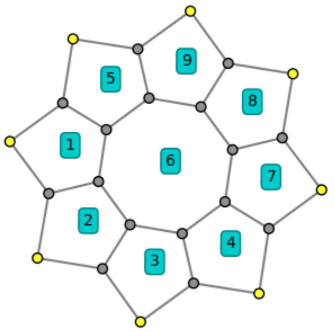

Fujimoto *et al.*, *Chem. Eur. J*, **2008**, *14*, 6053[77].

Ring	NICS(0)iso	NICS(0)zz	NICS(1)iso	NICS(1)zz	NICS(−1)iso	NICS(−1)zz
1	−7.958	−18.1701	−6.254	−5.2059	−6.2545	−5.4374
2	−7.6645	−19.5418	−6.0042	−5.0215	−6.0127	−4.5677
3	−8.1409	−22.1827	−6.3649	−4.3165	−6.3592	−3.0735
4	−7.5757	−20.8141	−5.9464	−4.7438	−5.9556	−3.7948
5	−7.6235	−20.822	−5.9537	−3.7708	−5.9822	−4.7796
6	5.61	−8.0918	3.0441	−3.9636	3.0602	−3.9178
7	−7.9614	−18.1644	−6.2384	−5.4455	−6.2709	−5.1779
8	−7.7326	−19.5397	−6.0304	−4.585	−6.023	−4.9855
9	−7.9336	−22.1299	−6.2786	−3.0207	−6.2437	−4.3157

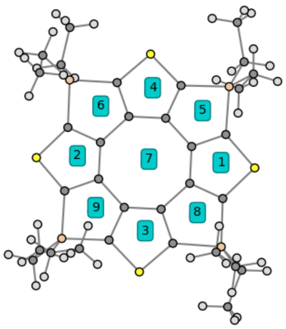

Serizawa *et al.*, *Chem. Eur. J*, **2017**, *23*, 6948[79].

Ring	NICS(0)iso	NICS(0)zz	NICS(1)iso	NICS(1)zz	NICS(−1)iso	NICS(−1)zz
1	−7.2006	−9.1656	−7.6781	−8.8965	−7.6418	−9.3907
2	−7.1226	−7.8153	−7.6458	−8.3719	−7.613	−7.979
3	−7.1248	−9.9837	−7.6484	−3.243	−7.6405	−8.192
4	−7.4055	−10.5088	−7.7618	−8.2977	−7.8268	−3.0982
5	2.9927	−2.0318	1.3623	0.2617	1.205	−2.2794
6	3.1482	−0.9763	1.4795	2.1405	1.3755	−0.9392
7	4.4598	2.0923	2.2021	0.8701	2.1588	0.9753
8	3.0816	−1.5012	1.3022	−2.0521	1.5052	0.9726
9	2.9001	−0.0246	1.3242	−1.2641	1.2674	1.7396

- Hetero[8]circulene (Type IV)

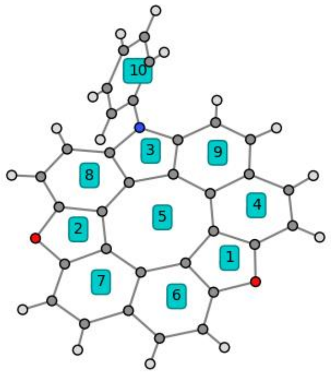

This work

Ring	NICS(0)iso	NICS(0)zz	NICS(1)iso	NICS(1)zz	NICS(−1)iso	NICS(−1)zz
1	−6.3531	11.5536	−6.7757	−8.6124	−6.8694	−11.2403
2	−6.6841	11.2546	−6.8695	−9.804	−7.1846	−11.6593
3	−7.3862	9.8215	−7.9559	−15.7769	−8.054	−13.0732
4	−8.0382	−2.9251	−9.8734	−18.3754	−10.2013	−19.7407
5	6.5803	28.8611	4.0937	15.037	3.9516	14.9272
6	−8.0833	−3.1295	−10.0514	−17.5878	−10.1972	−20.9512
7	−7.7135	−2.3183	−9.6176	−16.8078	−9.92	−19.7128
8	−9.0231	−3.0262	−10.3557	−20.6596	−10.2687	−19.7446
9	−6.9421	0.5797	−9.0276	−18.971	−9.5943	−16.3821
10	−6.1272	−3.1913	−9.6548	−4.6231	−9.3831	−5.1179

- Hetero[9]circulenes

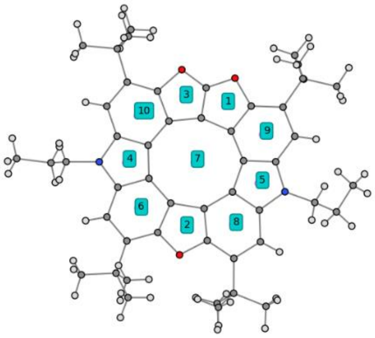

Pedersen *et al.*, *JACS*, **2020**, *142*, 14058[80].

Ring	NICS(0)iso	NICS(0)zz	NICS(1)iso	NICS(1)zz	NICS(−1)iso	NICS(−1)zz
1	−7.2957	−20.9246	−6.4424	−2.8621	−6.6897	−3.5133
2	−7.4625	−23.9703	−7.2072	−5.5336	−7.1002	−5.1647
3	−7.3706	−23.3608	−6.6729	−3.0812	−6.6459	−3.889
4	−9.0831	−15.9156	−8.8622	−3.1582	−9.3317	−5.5305
5	−8.6029	−16.3786	−8.5626	−4.5667	−9.2259	−4.55
6	−9.6351	−13.9355	−10.4587	−5.0367	−11.175	−7.0247
7	3.3466	−8.5266	1.9747	−3.5801	1.8091	−4.7429
8	−9.6985	−13.6684	−10.7749	−5.5159	−10.9753	−5.1342
9	−9.8978	−13.9639	−10.5332	−5.3427	−10.7392	−6.004
10	−10.0942	−13.9339	−10.7626	−5.0404	−10.7405	−5.7566

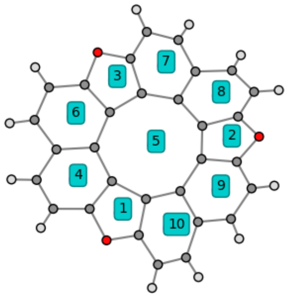

Yang *et al.*, *ACIE*, **2024**, *63*, e202402756[82].

Ring	NICS(0)iso	NICS(0)zz	NICS(1)iso	NICS(1)zz	NICS(−1)iso	NICS(−1)zz
1	−7.7964	−17.0145	−6.8477	−5.1547	−9.6727	−7.8816
2	−7.3519	−8.9615	−8.3663	−7.8445	−7.4914	−4.2851
3	−7.54	−19.0311	−7.1332	−1.5579	−8.9398	−7.3186
4	−7.5425	−7.5821	−10.3245	−7.9881	−9.2043	−7.0493
5	4.2097	−5.4651	2.3444	−4.8351	1.323	−10.2284
6	−7.5242	−10.2744	−8.8479	−3.2936	−10.8254	−9.8092
7	−7.5745	−10.9883	−10.7386	−4.2201	−9.1107	−4.5668
8	−7.2887	−9.3731	−10.7204	−5.9704	−8.6324	−6.2947
9	−7.2738	−10.676	−10.7752	−6.8343	−8.7522	−2.1639
10	−7.5479	−10.4593	−9.5923	−4.8612	−10.0683	−3.7605

- Hetero[10]circulenes

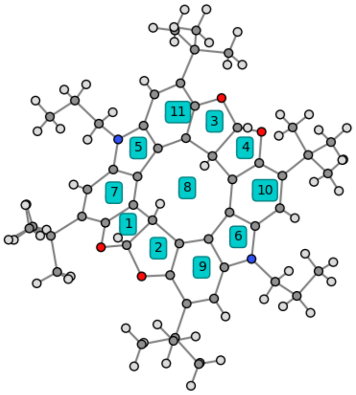

Pedersen *et al.*, *JACS*, **2020**, *142*, 14058[82].

Ring	NICS(0)iso	NICS(0)zz	NICS(1)iso	NICS(1)zz	NICS(−1)iso	NICS(−1)zz
1	−2.5747	−3.2392	−2.2568	−3.0064	−0.341	−1.1923
2	−2.3885	−22.6964	−1.0394	−3.0229	−1.7083	−5.0503
3	−2.2081	−23.0311	−2.4079	−4.2623	−1.0137	−2.2364
4	−2.6615	−10.6998	−0.4176	−1.6587	−2.6024	−5.0405
5	−8.2309	−15.5767	−9.2718	−7.9079	−8.2447	−3.9201
6	−8.3091	−18.3364	−8.3011	−3.6587	−9.1548	−6.8782
7	−9.1391	−10.5256	−11.0837	−9.6629	−9.6728	−7.9404
8	3.3533	−4.3288	0.7533	−6.7847	1.5194	−5.3546
9	−8.854	−13.551	−10.2987	−4.2878	−10.2599	−5.4227
10	−9.0766	−13.0002	−10.1132	−6.2686	−10.6973	−8.711
11	−8.8995	−12.9672	−9.9939	−6.1358	−10.5485	−4.7316

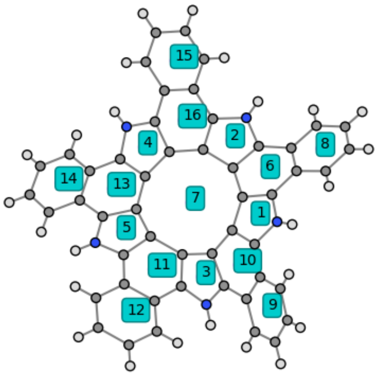

Matsuo *et al.*, *ACIE*, **2022**, *61*, e202116789[83].

Ring	$NICS(0)_{iso}$	$NICS(0)_{zz}$	$NICS(1)_{iso}$	$NICS(1)_{zz}$	$NICS(-1)_{iso}$	$NICS(-1)_{zz}$
**1**	−8.3625	−10.5646	−10.6677	−11.8555	−7.072	−2.1263
**2**	−8.6176	−13.1689	−6.8611	−9.0787	−10.9389	−9.8282
**3**	−8.8095	−21.2965	−7.9925	−6.8051	−10.2333	−2.5511
**4**	−8.5748	−18.3953	−10.194	−7.6488	−7.7059	−0.2315
**5**	−8.855	−11.841	−8.8742	−9.1195	−9.5793	−4.9109
**6**	−4.1331	−1.5212	−6.2496	−8.92	−6.9986	−5.3603
**7**	5.0759	−3.7453	3.5439	−8.2097	3.7022	1.8193
**8**	−6.4092	−4.6246	−9.8276	−13.9933	−9.7283	−15.2056
**9**	−6.3266	−10.422	−9.4521	−4.6316	−9.9091	−2.6054
**10**	−4.4703	−13.4177	−16.4663	−19.9253	−10.2168	−3.567
**11**	−4.2542	−10.3791	−4.8335	−7.1934	−8.0156	−4.505
**12**	−6.2939	−8.6239	−9.9496	−5.848	−9.4213	−7.7697
**13**	−4.5044	−12.1843	−8.0155	−9.9332	−5.6396	−2.2429
**14**	−6.4078	−7.9742	−9.403	−5.3771	−10.1347	−4.5413
**15**	−6.4736	−9.309	−9.9975	−5.9598	−9.63	−8.2587
**16**	−4.48	−9.5406	−5.9635	−6.608	−7.5553	−4.1813

18. **Supplementary Note 11:** *DFT calculations of the redox potentials*

Calculations of the excited reduction potential of dioxaza[8]circulene $E^{1/2}(3a^{\bullet+}/3a^{*})$

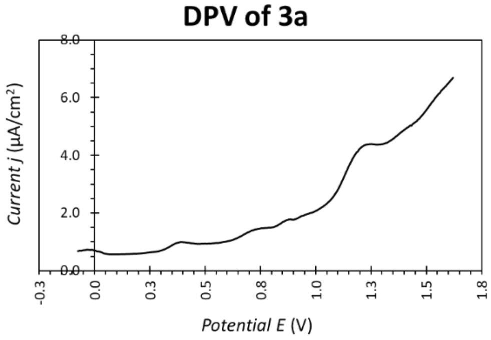

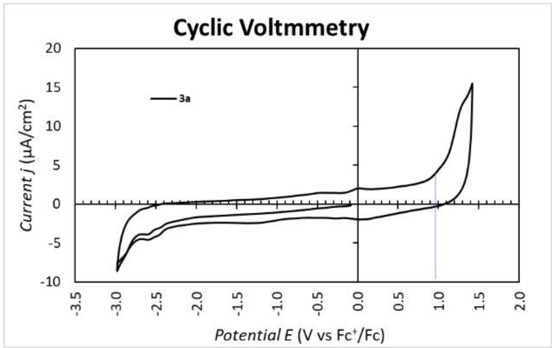

Oxidation potential of **3a** vs. SCE = 1.34 V

$E_{1/2}$ (PC^{+}/PC) 1.34 V

$E_{0,0}$ = hc/λ = 1240/377.5 = 3.29 (eV)

$E_{1/2}$ (PC^{+}/PC^{*}) = $E_{1/2}$ (PC^{+}/PC) − $E_{0,0}$ = 1.34 − 3.29 = −1.95

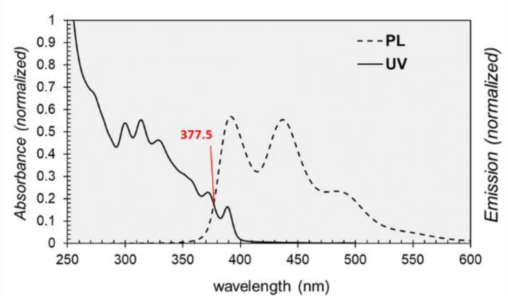

DFT calculation of solution phase electrochemical redox potential

*We followed Nicewicz and coworkers' protocol to calculate redox potentials for our dioxaza[8]circulene **3a** using the B3LYP functional, combined with the 6-31+G(d,p) basis set and the CPCM solvent continuum model for MeCN solvation[45]. All calculations were performed using Gaussian 09, with structures subjected to geometry optimization and subsequent frequency calculations to confirm that the geometries were true minima and to compute free energies at 298 K. Solution-phase energies were referenced to SCE by subtracting 4.281 V (the absolute potential of SHE) and 0.141 V (the conversion of SHE to SCE in MeCN).*

$$E_{1/2}^{\mathrm{o,calc}} = -\frac{(G_{298}[\mathrm{reduced}] - G_{298}[\mathrm{oxidized}])}{n_e \mathcal{F}} - E_{1/2}^{\mathrm{o,SHE}} + E_{1/2}^{\mathrm{o,SCE}}$$

Where n_e is the number of electrons transferred (here, $n_e = 1$), $\mathcal{F}$ is the Faraday constant (23.06 kcal mol^{-1} V^{-1}), $E_{1/2}^{o,SHE}$ is the absolute value for the standard hydrogen electrode (SHE, 4.281 V), $E_{1/2}^{o,SCE}$ is the potential of the saturated calomel electrode (SCE) relative to SHE in acetonitrile (−0.141 V), and G oxidized and G reduced are the Gibbs free energies in acetonitrile as obtained from DFT calculations.

*Compound **3a***

G_{298} [ground state]	G_{298} [excited state]	G_{298} [anion radical]	G_{298} cation radical]
−1472.76019 Hartree	−1472.652439 Hartree	−1472.841678 Hartree	−1472.561233 Hartree

$\Delta G^{o}_{1/2}$ [Red]	$\Delta G^{o}_{1/2}$ [Ox]	$\Delta G^{o}_{1/2}$ ($CAT^{\bullet -}/CAT^{*}$)	$\Delta G^{o}_{1/2}$ ($CAT^{\bullet +}/CAT^{*}$)
−0.081488 Hartree	−0.198957 Hartree	−0.189239 Hartree	−0.091206 Hartree
−51.1339645 kcal·mol^{-1}	−124.846114 kcal·mol^{-1}	−118.748040 kcal·mol^{-1}	−57.2320386 kcal·mol^{-1}

$E^{1/2}$(**3a**/**3a**$^{\bullet -}$)	$E^{1/2}$(**3a**$^{\bullet +}$/**3a**)	$E^{1/2}$(**3a***/**3a**$^{\bullet -}$)	$E^{1/2}$(**3a**$^{\bullet +}$/**3a***)
−2.20 V	0.99 V	0.73 V	−1.94

*Compound **2***

G_{298} [ground state]	G_{298} [excited state]	G_{298} [anion radical]	G_{298} cation radical]
−536.26224 Hartree	−536.126824 Hartree	−536.329388 Hartree	−536.051141 Hartree

$\Delta G^{o}_{1/2}$ [Red]	$\Delta G^{o}_{1/2}$ [Ox]	$\Delta G^{o}_{1/2}$ ($CAT^{\bullet -}/CAT^{*}$)	$\Delta G^{o}_{1/2}$ ($CAT^{\bullet +}/CAT^{*}$)
−0.067148 Hartree	−0.211099 Hartree	−0.202564 Hartree	−0.075683 Hartree
−42.1355714 kcal·mol^{-1}	−132.465256 kcal·mol^{-1}	−127.109518 kcal·mol^{-1}	−47.4913096 kcal·mol^{-1}

$E^{1/2}$(**2**/**2**$^{\bullet -}$)	$E^{1/2}$(**2**$^{\bullet +}$/**2**)	$E^{1/2}$(**2***/**2**$^{\bullet -}$)	$E^{1/2}$(**2**$^{\bullet +}$/**2***)
−2.59 V	1.32 V	1.09 V	−2.36

*Compound **1a'***

G_{298} [ground state]	G_{298} [excited state]	G_{298} [anion radical]	G_{298} cation radical]
−1016.48772 Hartree	−1016.372095 Hartree	−1016.560261 Hartree	−1016.289879 Hartree

$\Delta G^{o}_{1/2}$ [Red]	$\Delta G^{o}_{1/2}$ [Ox]	$\Delta G^{o}_{1/2}$ ($CAT^{\bullet-}/CAT^{*}$)	$\Delta G^{o}_{1/2}$ ($CAT^{\bullet+}/CAT^{*}$)
−0.072541 Hartree	−0.197841 Hartree	−0.188166 Hartree	−0.082216 Hartree
−45.5196951 kcal·mol^{-1}	−124.14582 kcal·mol^{-1}	−118.074730 kcal·mol^{-1}	−51.5907867 kcal·mol^{-1}

$E^{1/2}$(**1a'**/**1a'**$^{\bullet-}$)	$E^{1/2}$(**1a'**$^{\bullet+}$/**1a'**)	$E^{1/2}$(**1a'***/**1a'**$^{\bullet-}$)	$E^{1/2}$(**1a'**$^{\bullet+}$/**1a'***)
−2.45 V	0.96 V	0.70 V	−2.18 V

19. **Supplementary Note 12:** NMR Spectra

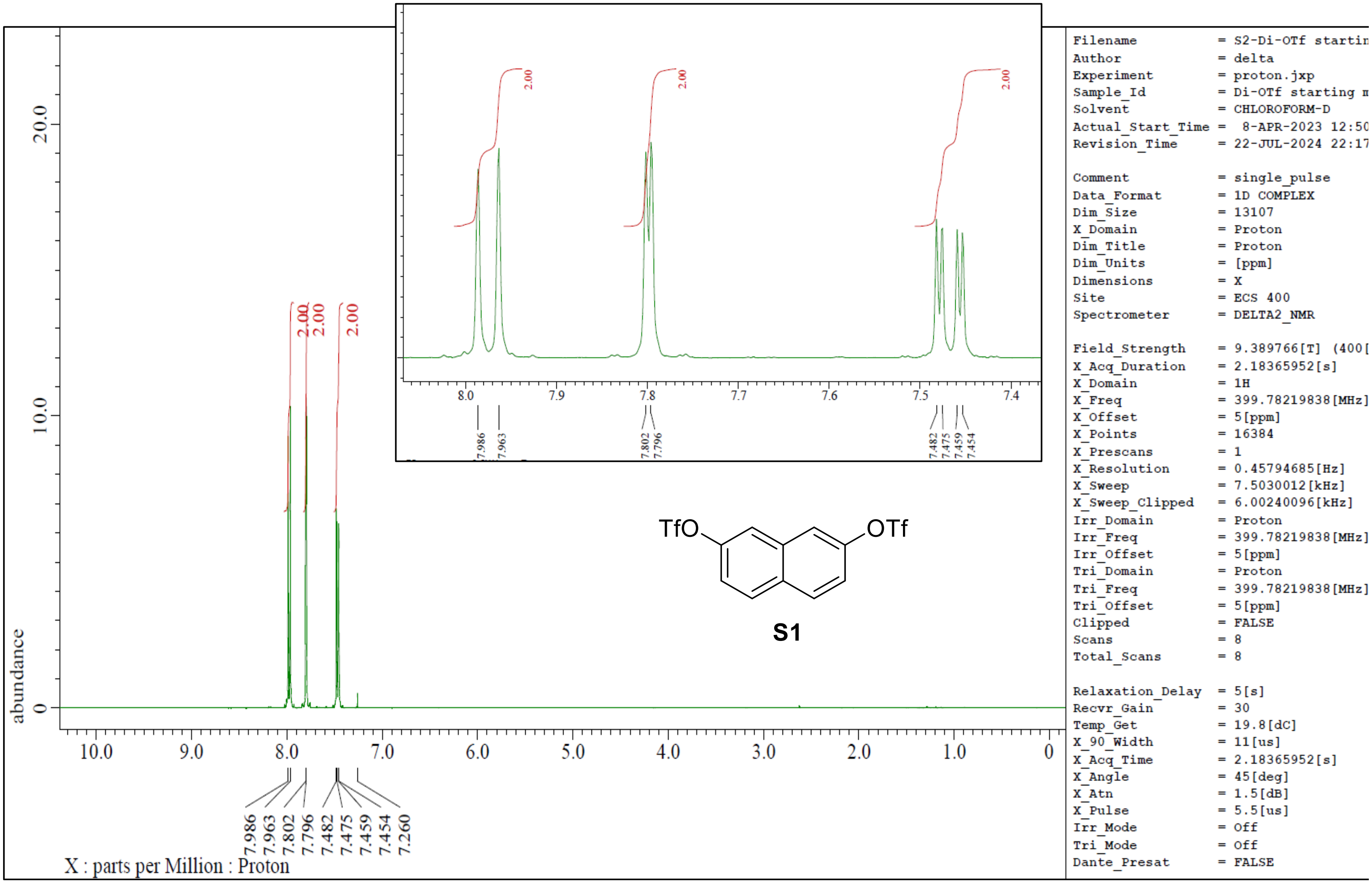

Compound **S1** (^{1}H NMR, 400 MHz, $CDCl_3$).

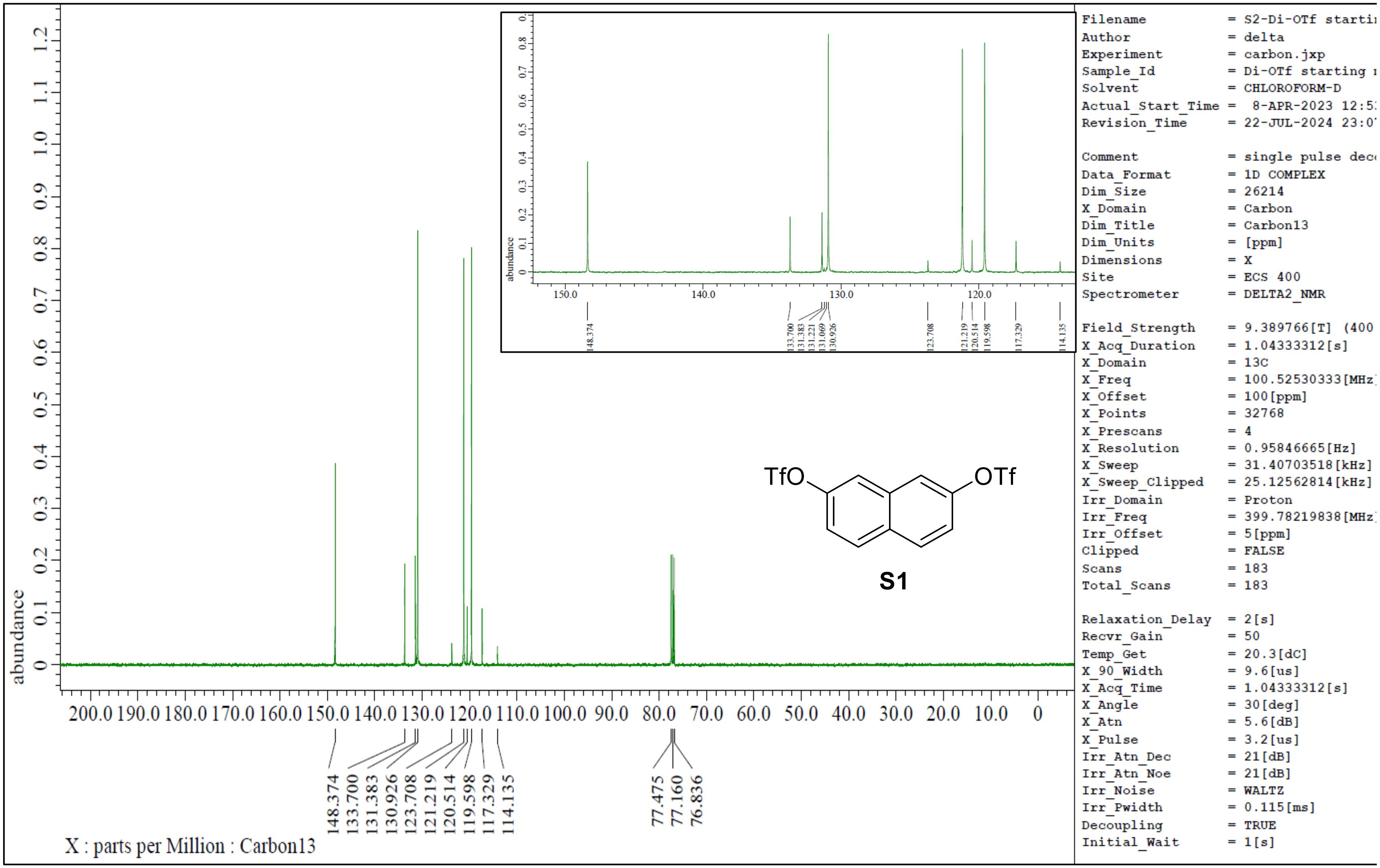

Compound **S1** (^{13}C NMR, 100 MHz, $CDCl_3$).

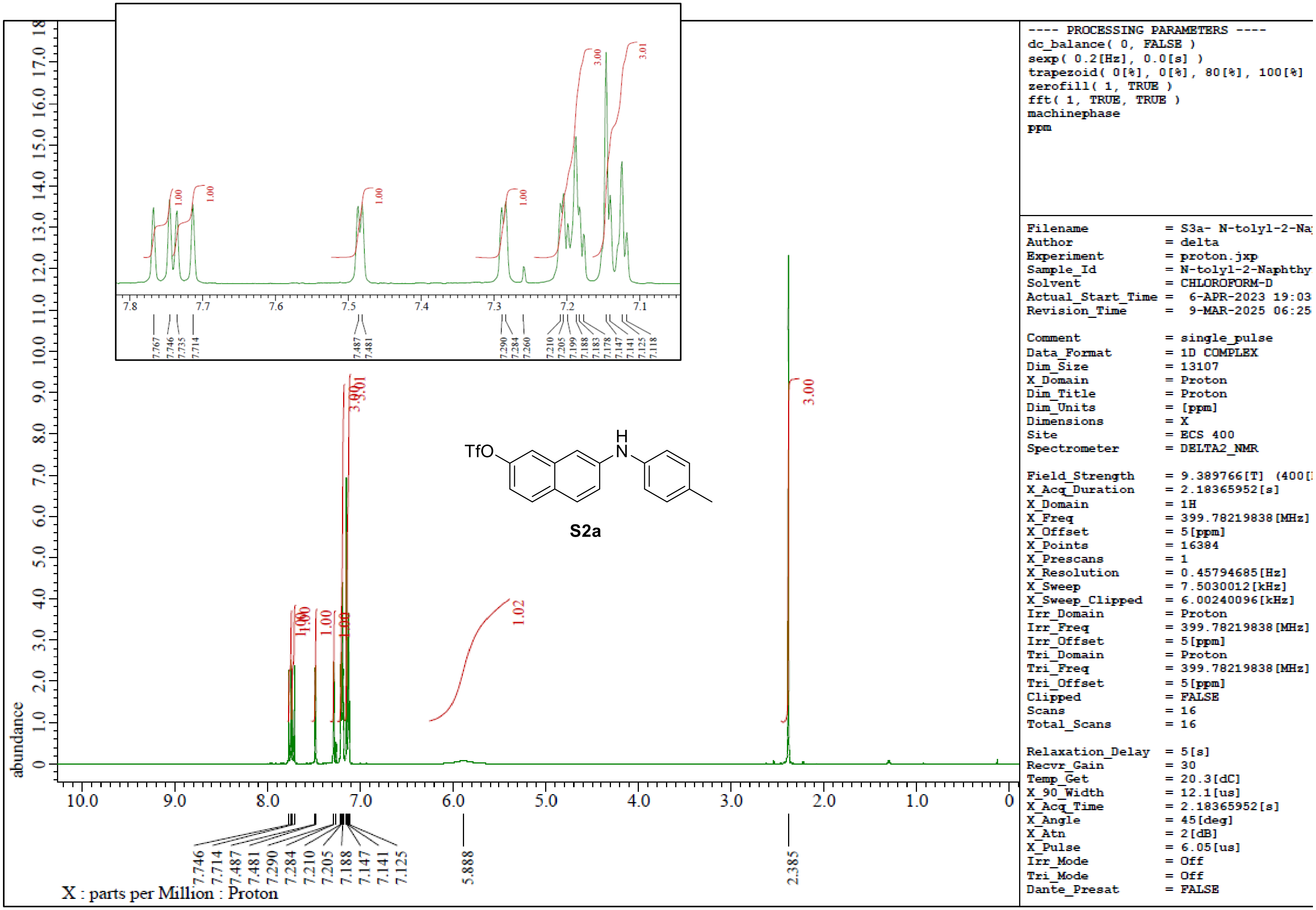

---- PROCESSING PARAMETERS ----
dc_balance(0, FALSE)
sexp(0.2[Hz], 0.0[s])
trapezoid(0[%], 0[%], 80[%], 100[%]
zerofill(1, TRUE)
fft(1, TRUE, TRUE)
machinephase
ppm
Filename = S3a- N-tolyl-2-Na
Author = delta
Experiment = proton.jxp
Sample_Id = N-tolyl-2-Naphthy
Solvent = CHLOROFORM-D
Actual_Start_Time = 6-APR-2023 19:03
Revision_Time = 9-MAR-2025 06:25
Comment = single_pulse
Data_Format = 1D COMPLEX
Dim_Size = 13107
X_Domain = Proton
Dim_Title = Proton
Dim_Units = [ppm]
Dimensions = X
Site = ECS 400
Spectrometer = DELTA2_NMR
Field_Strength = 9.389766[T] (400[
X_Acq_Duration = 2.18365952[s]
X_Domain = 1H
X_Freq = 399.78219838[MHz]
X_Offset = 5[ppm]
X_Points = 16384
X_Prescans = 1
X_Resolution = 0.45794685[Hz]
X_Sweep = 7.5030012[kHz]
X_Sweep_Clipped = 6.00240096[kHz]
Irr_Domain = Proton
Irr_Freq = 399.78219838[MHz]
Irr_Offset = 5[ppm]
Tri_Domain = Proton
Tri_Freq = 399.78219838[MHz]
Tri_Offset = 5[ppm]
Clipped = FALSE
Scans = 16
Total_Scans = 16
Relaxation_Delay = 5[s]
Recvr_Gain = 30
Temp_Get = 20.3[dC]
X_90_Width = 12.1[us]
X_Acq_Time = 2.18365952[s]
X_Angle = 45[deg]
X_Atn = 2[dB]
X_Pulse = 6.05[us]
Irr_Mode = Off
Tri_Mode = Off
Dante_Presat = FALSE
TfO
H
N
S2a
abundance
X : parts per Million : Proton
7.746
7.714
7.487
7.481
7.290
7.284
7.210
7.205
7.188
7.147
7.141
7.125
5.888
2.385

Compound **S2a** (^{1}H NMR, 400 MHz, $CDCl_3$).

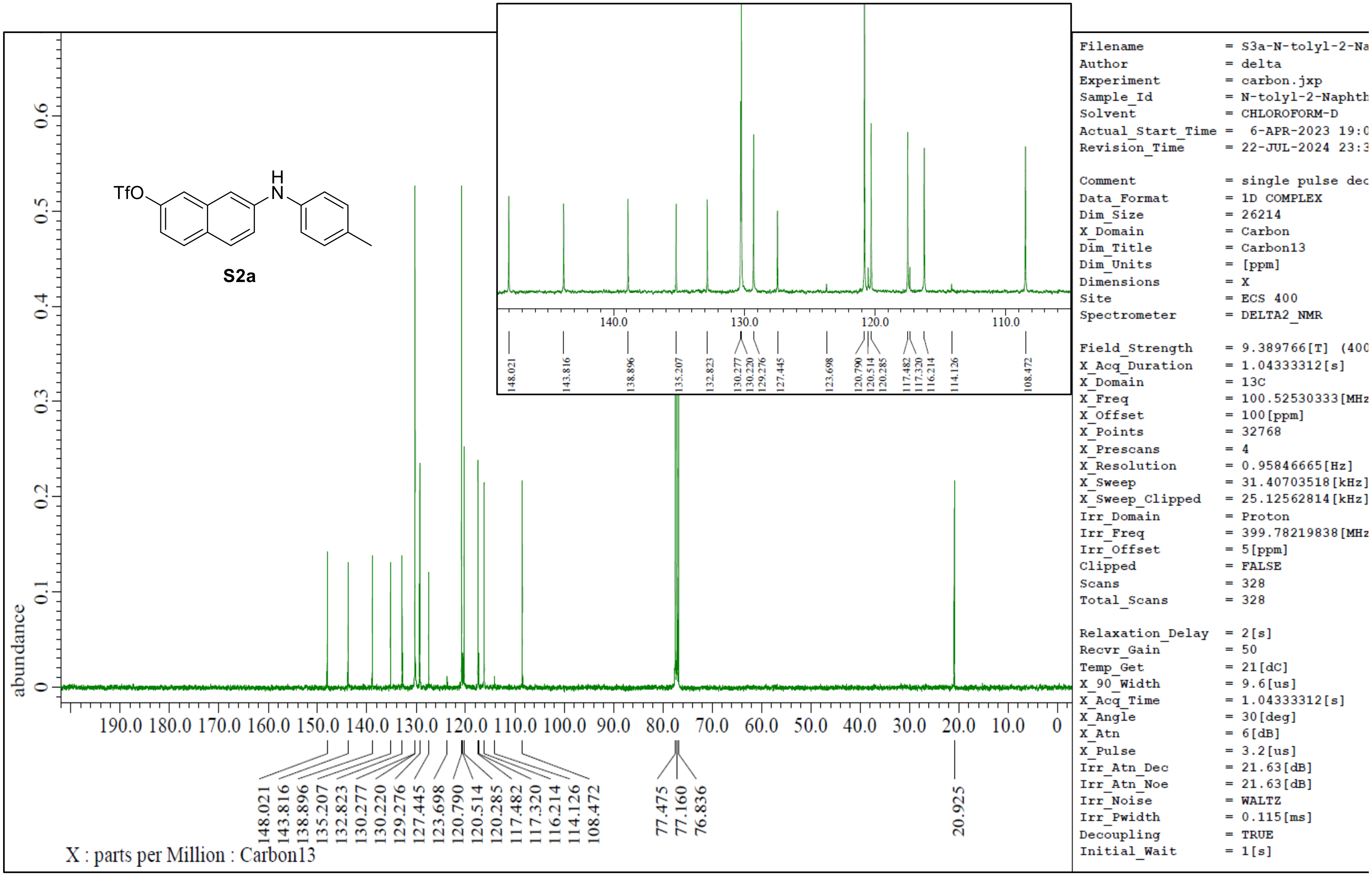

Compound **S2a** (^{13}C NMR, 100 MHz, $CDCl_3$).

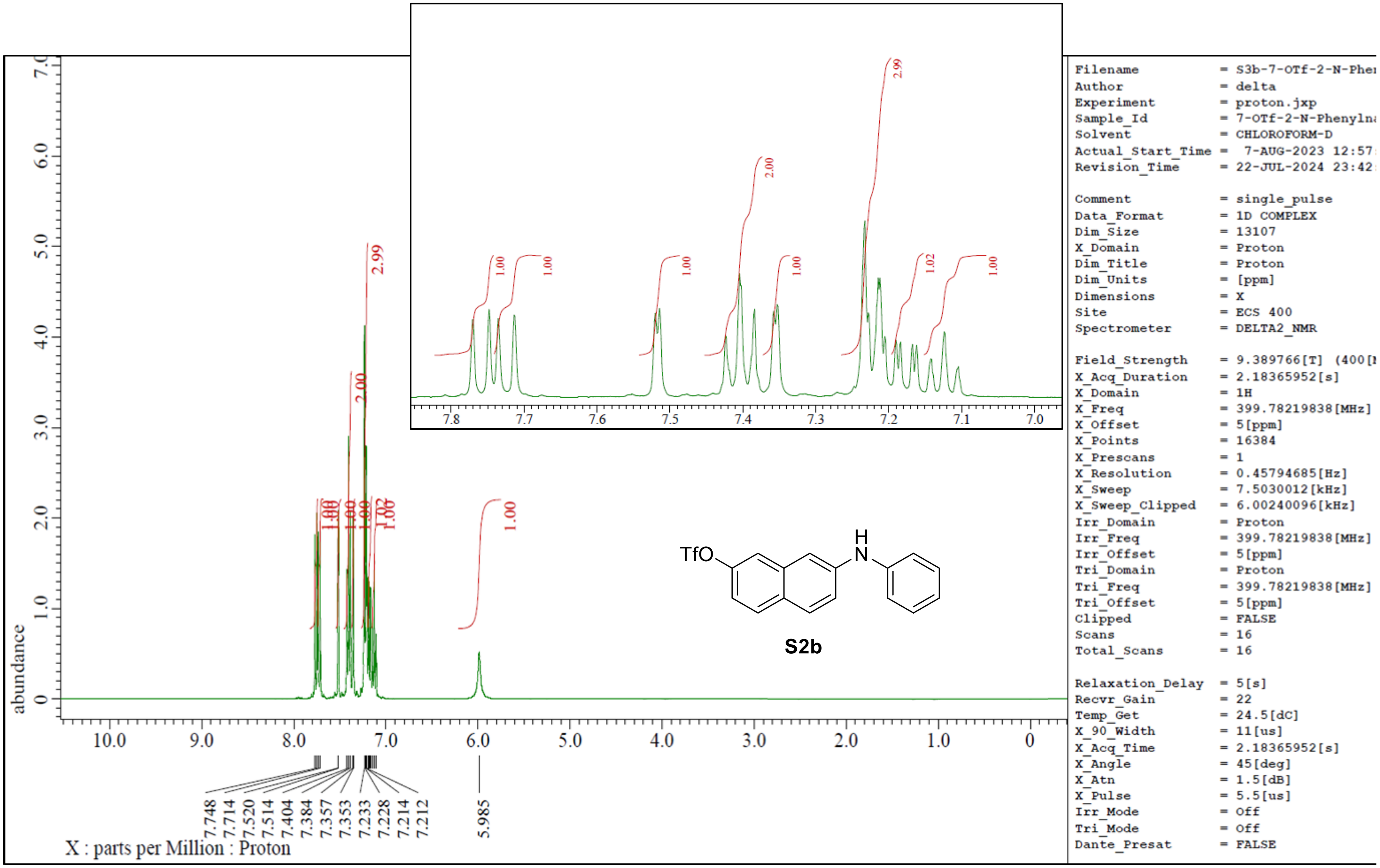

Compound **S2b** (^1H NMR, 400 MHz, $CDCl_3$).

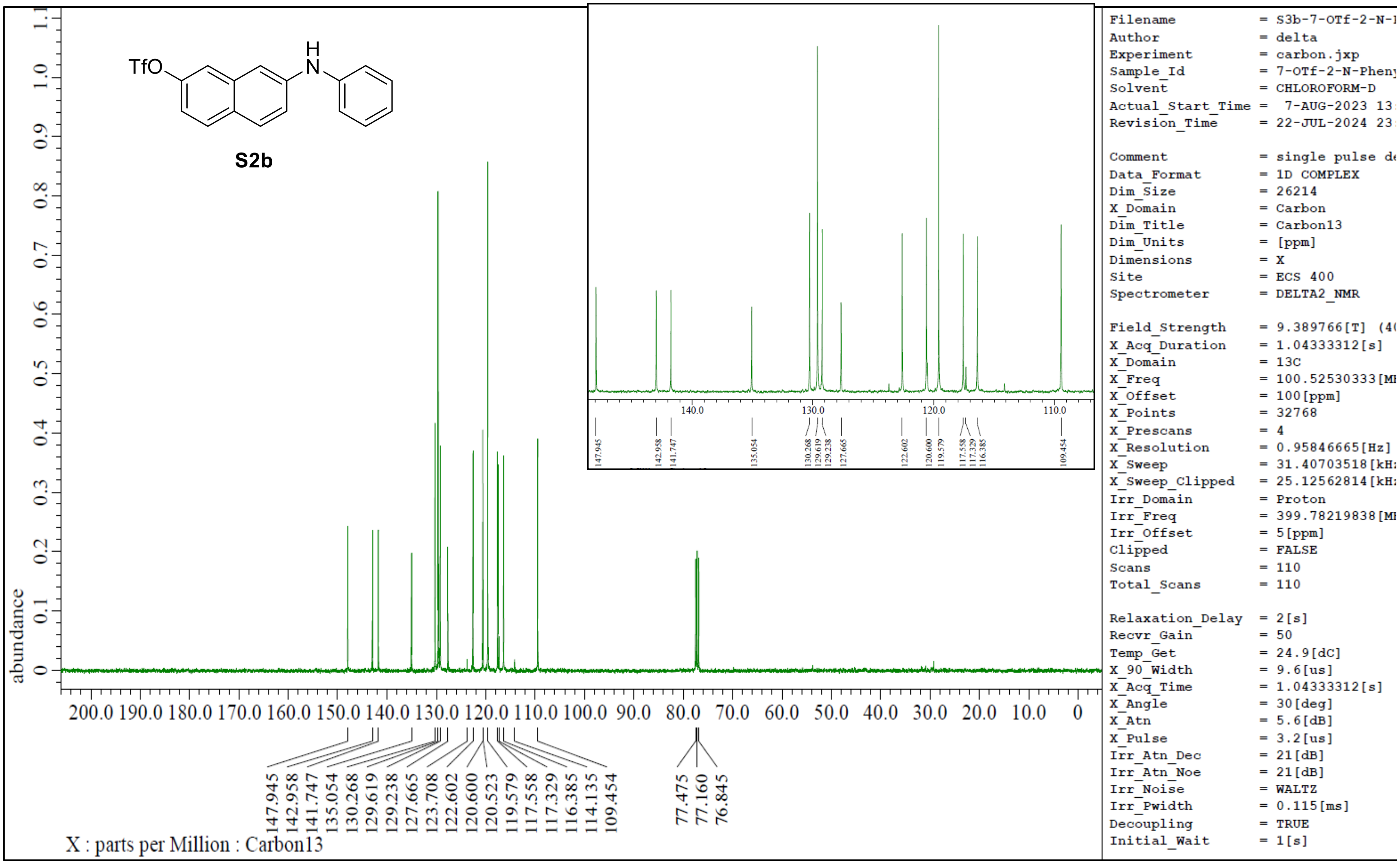

Compound **S2b** (^{13}C NMR, 100 MHz, $CDCl_3$).

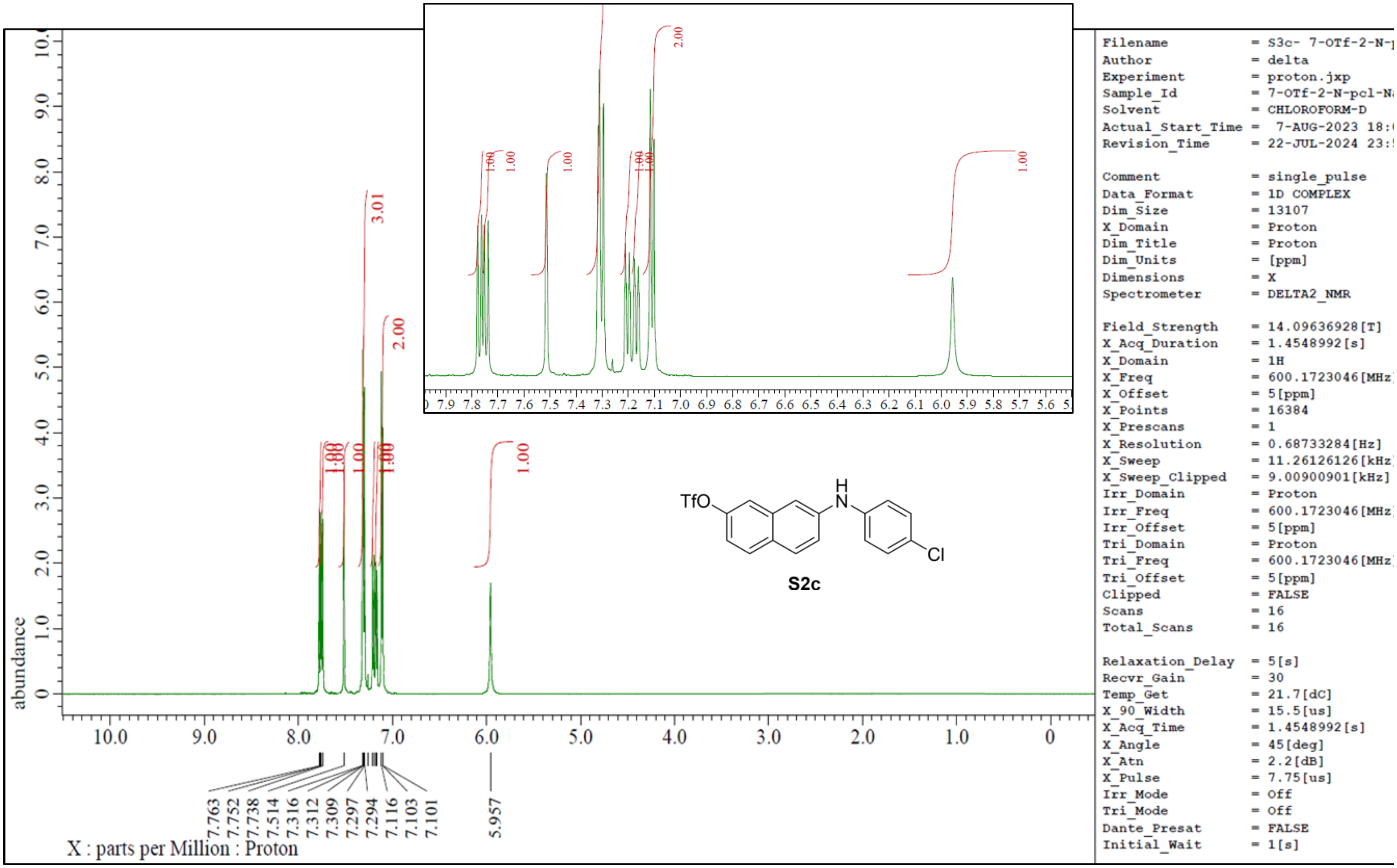

Compound **S2c** (^{1}H NMR, 600 MHz, $CDCl_3$).

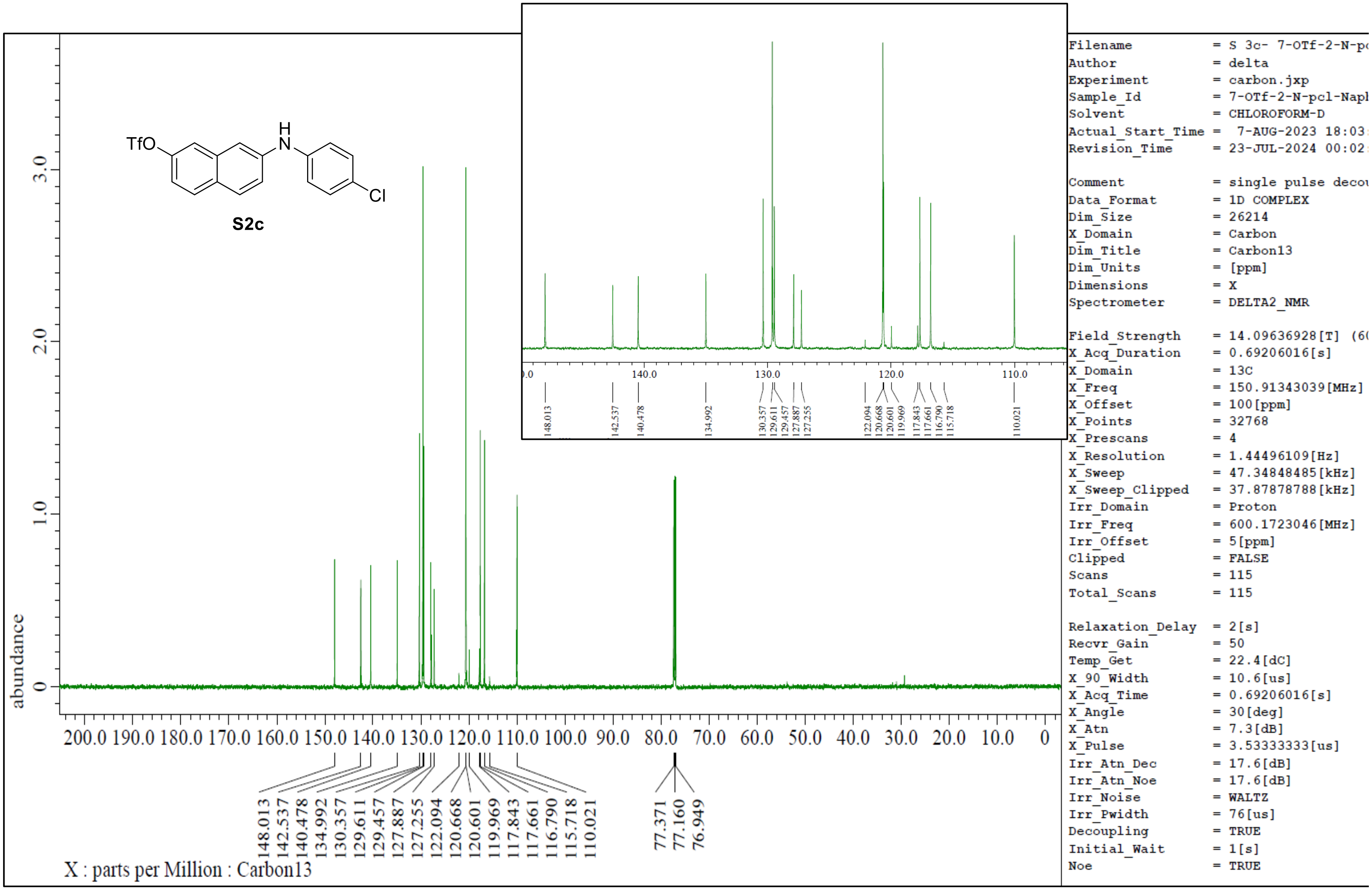

Compound **S2c** (^{13}C NMR, 150 MHz, $CDCl_3$).

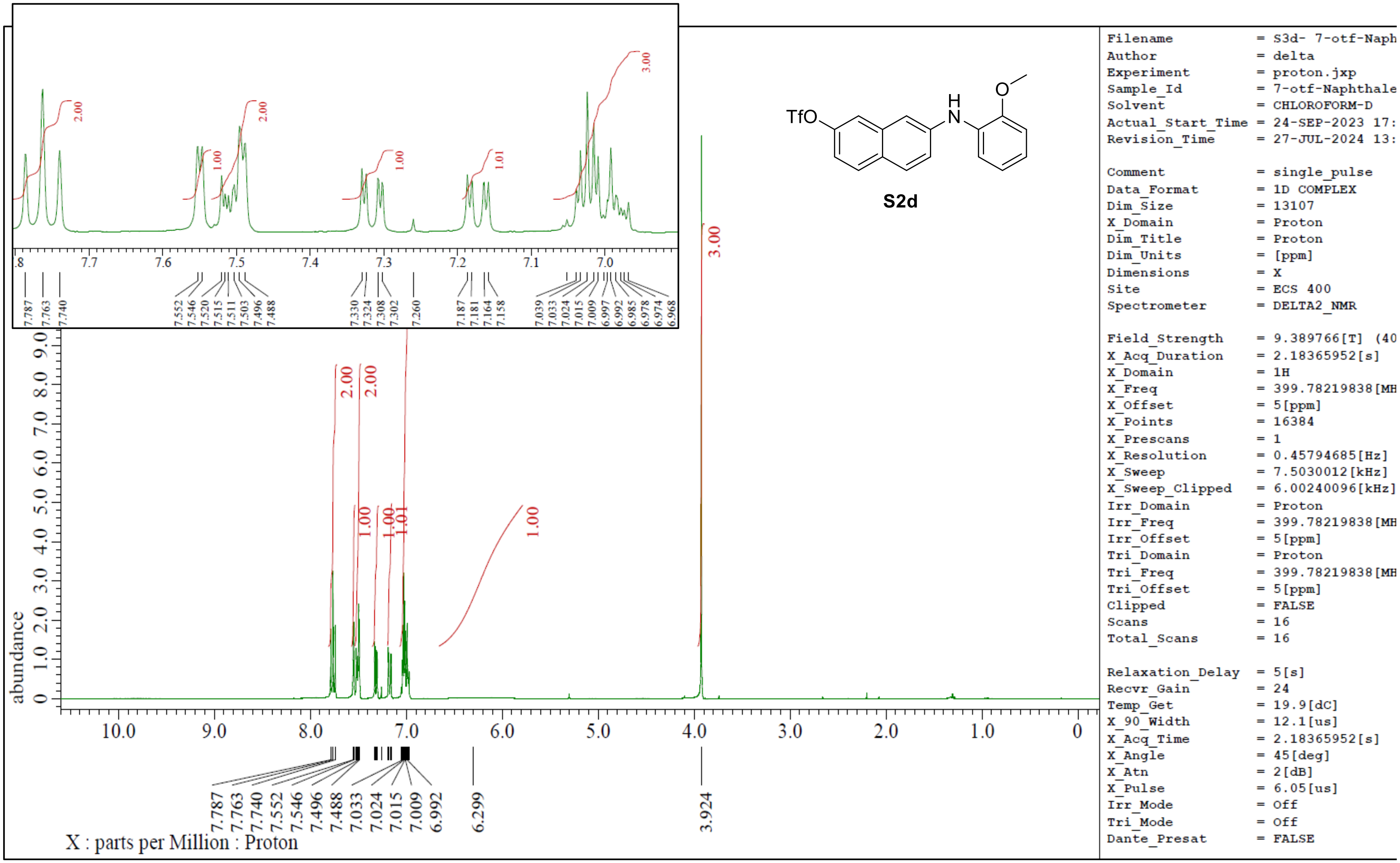

Compound **S2d** (^{1}H NMR, 400 MHz, $CDCl_3$).

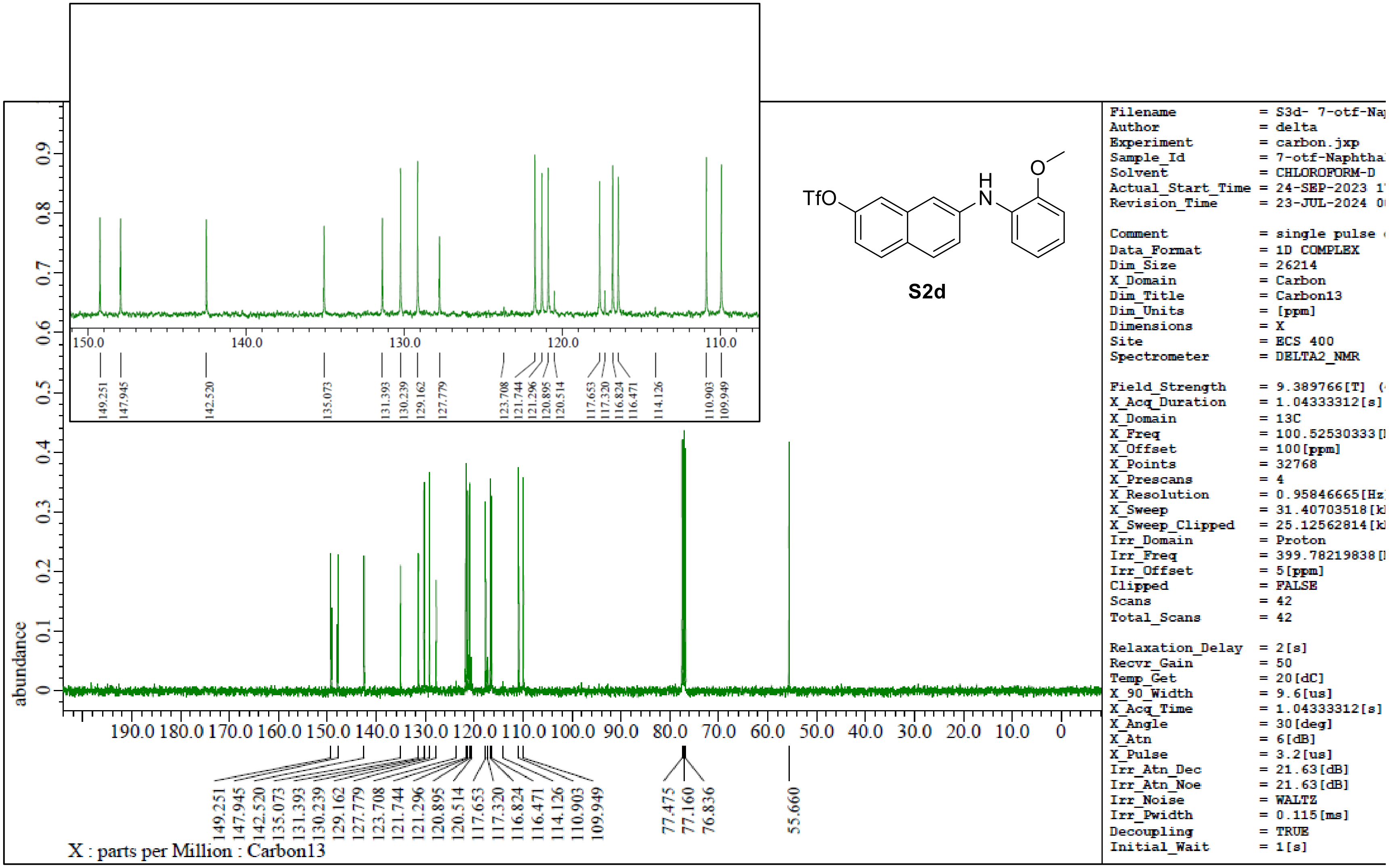

Compound **S2d** (^{13}C NMR, 100 MHz, $CDCl_3$).

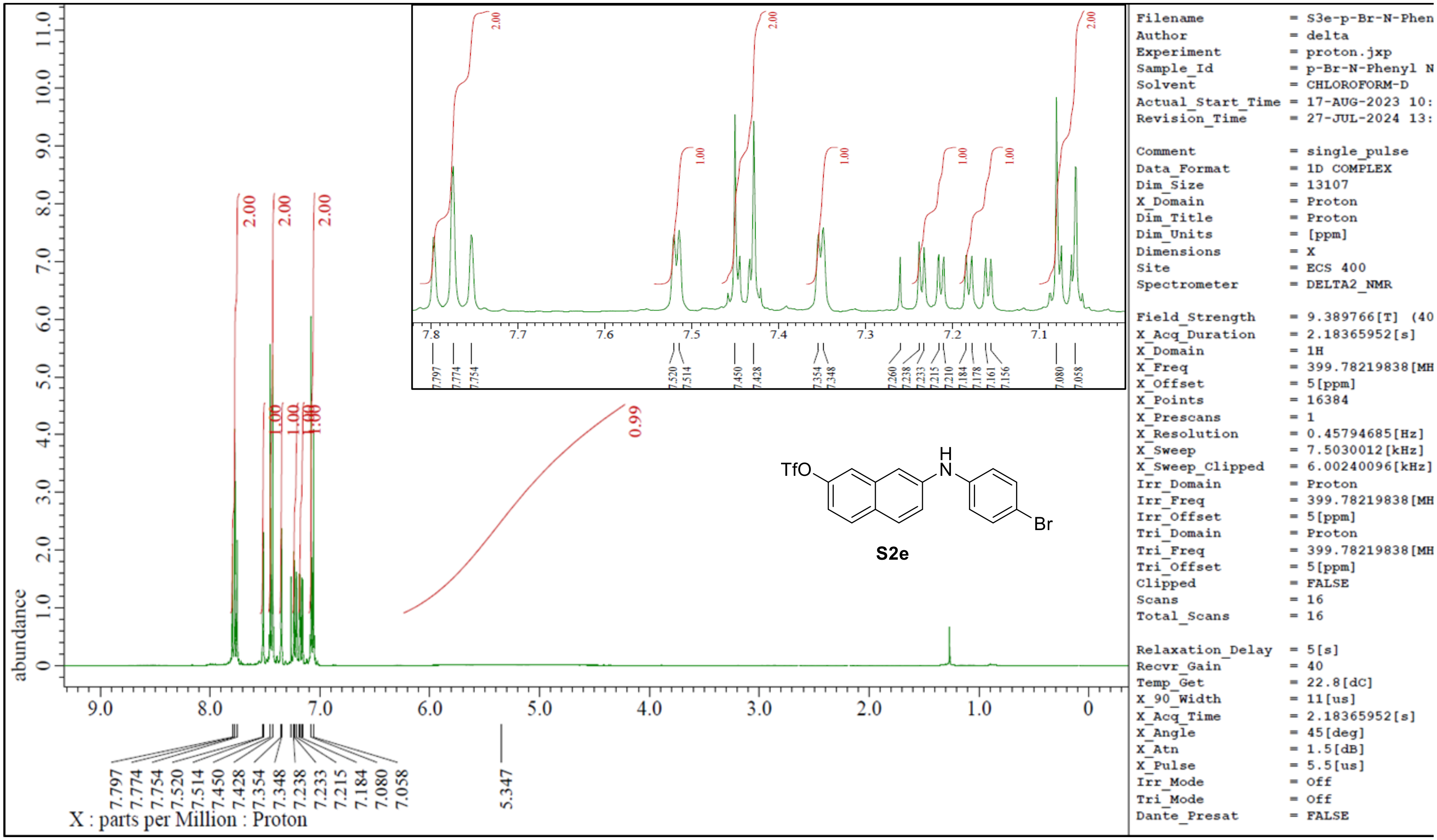

Compound **S2e** (^{1}H NMR, 400 MHz, $CDCl_3$).

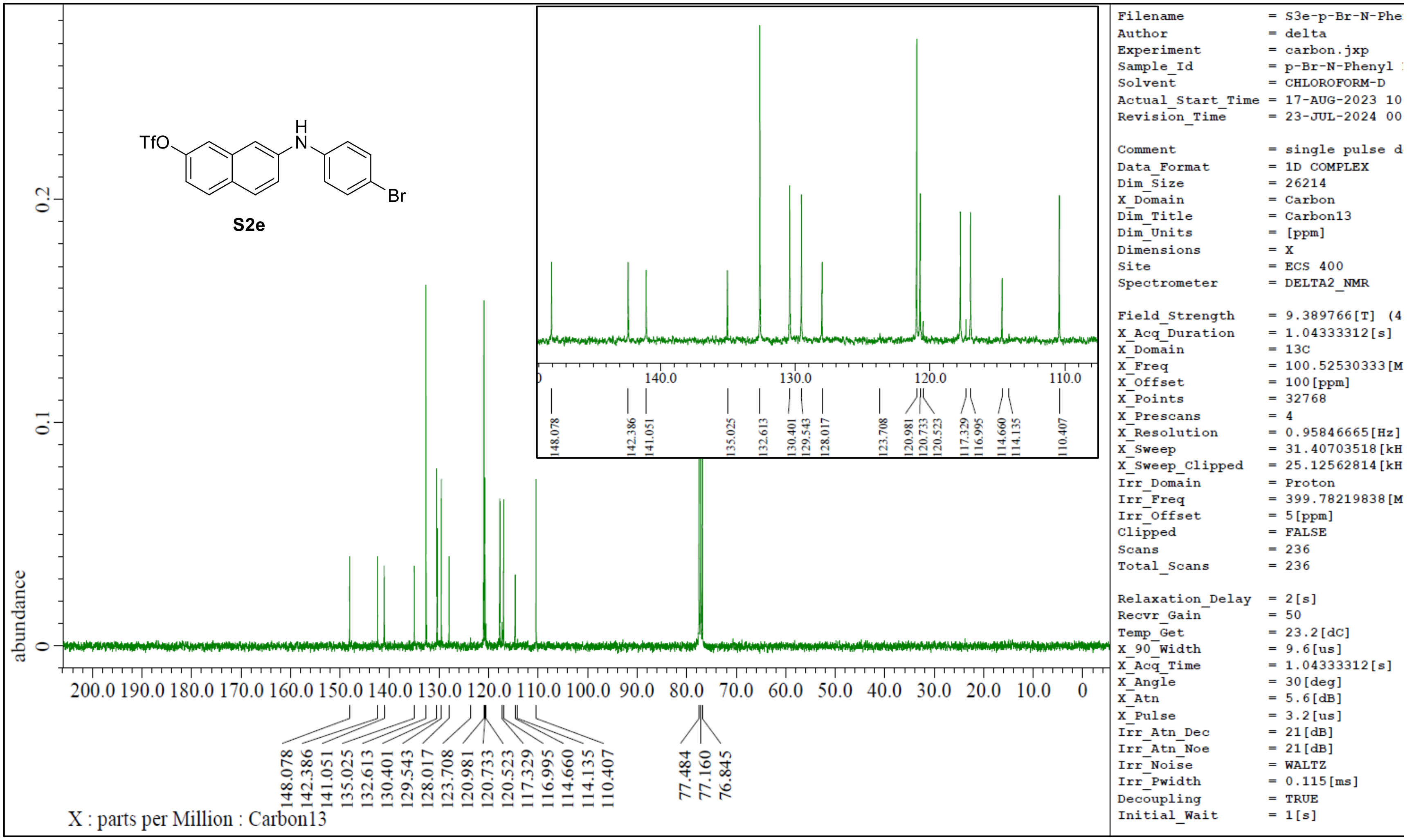

Compound **S2e** (^{13}C NMR, 100 MHz, $CDCl_3$).

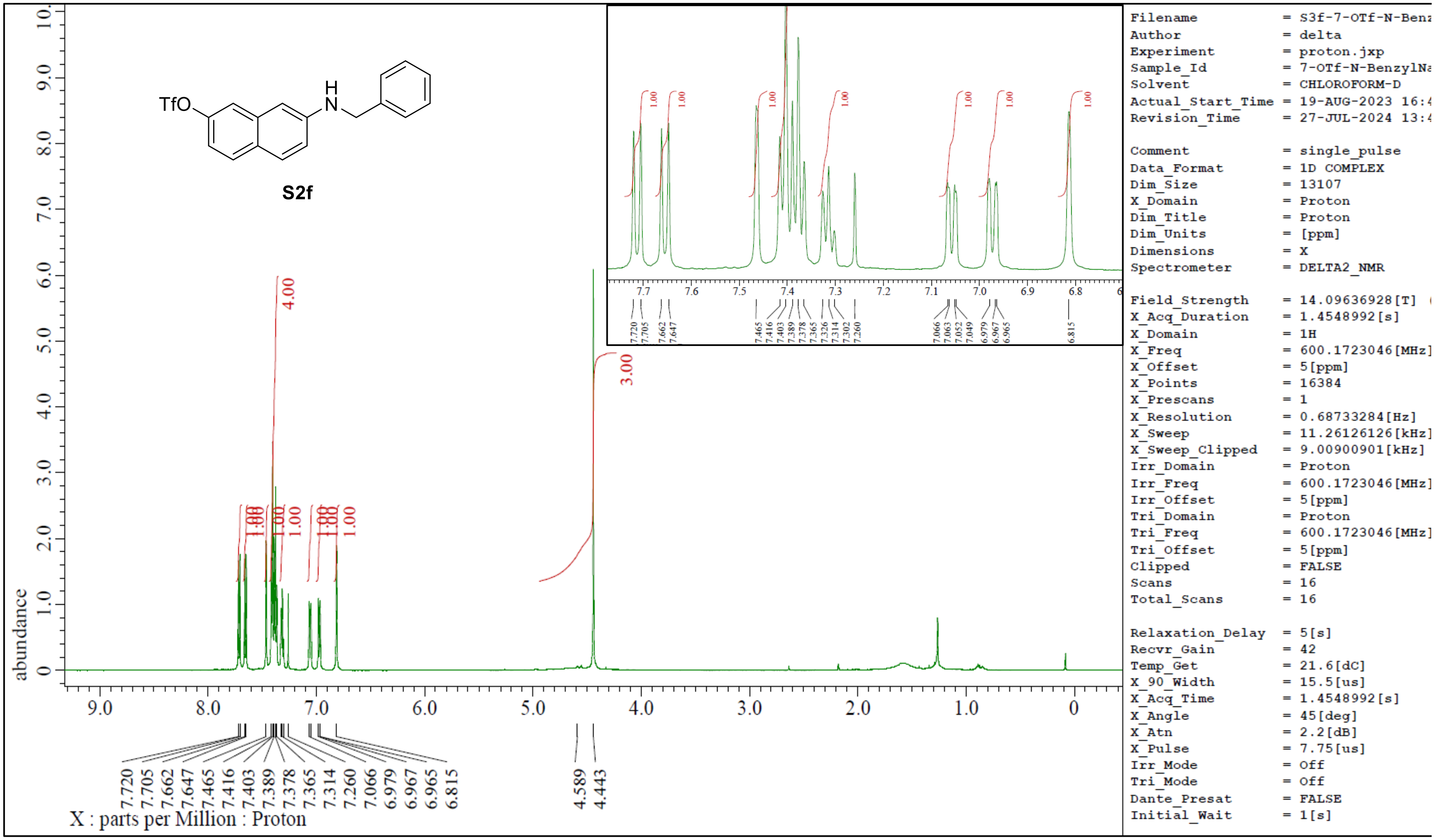

Compound **S2f** (^{1}H NMR, 600 MHz, $CDCl_3$).

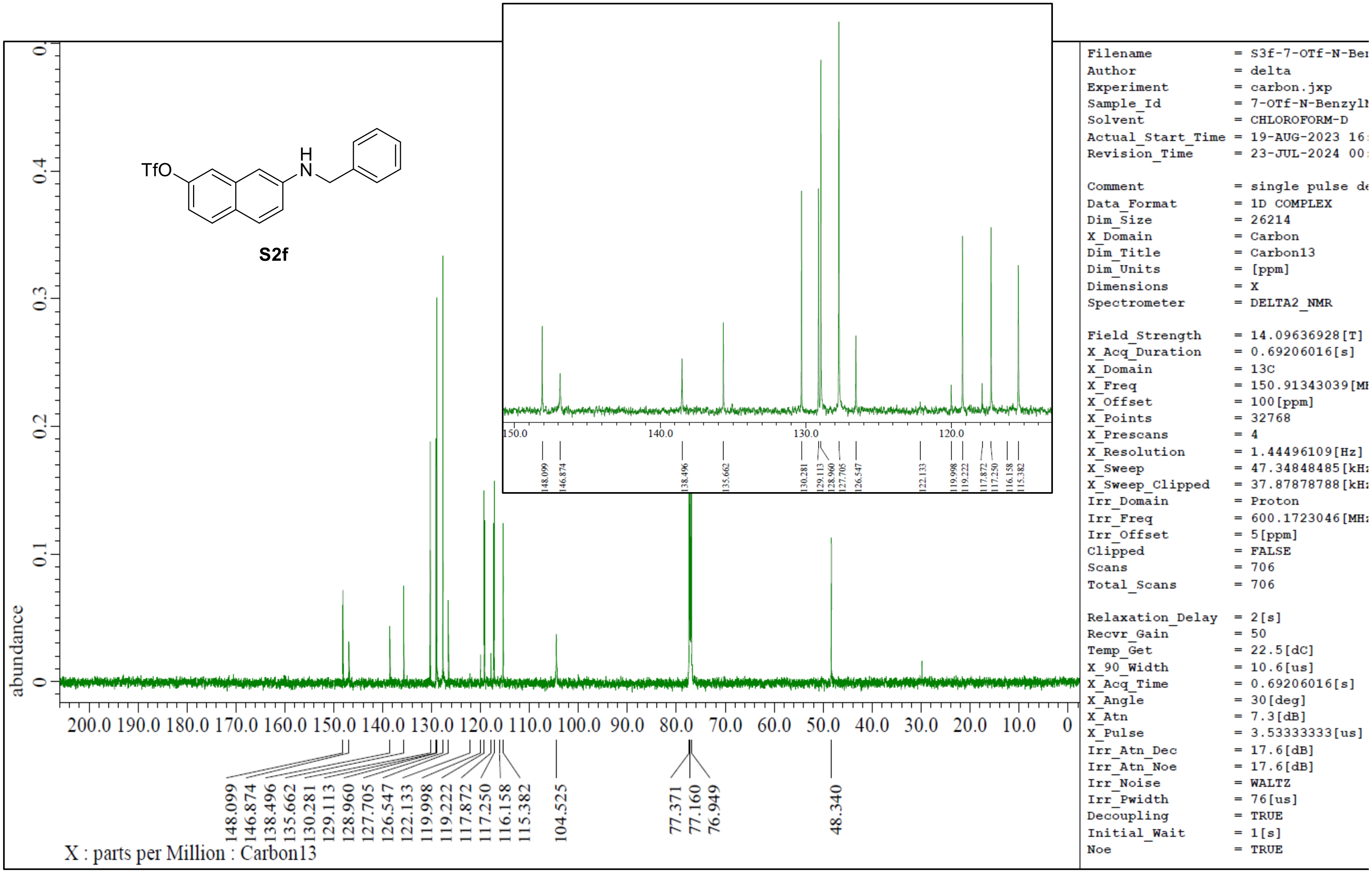

Compound **S2f** (^{13}C NMR, 150 MHz, $CDCl_3$).

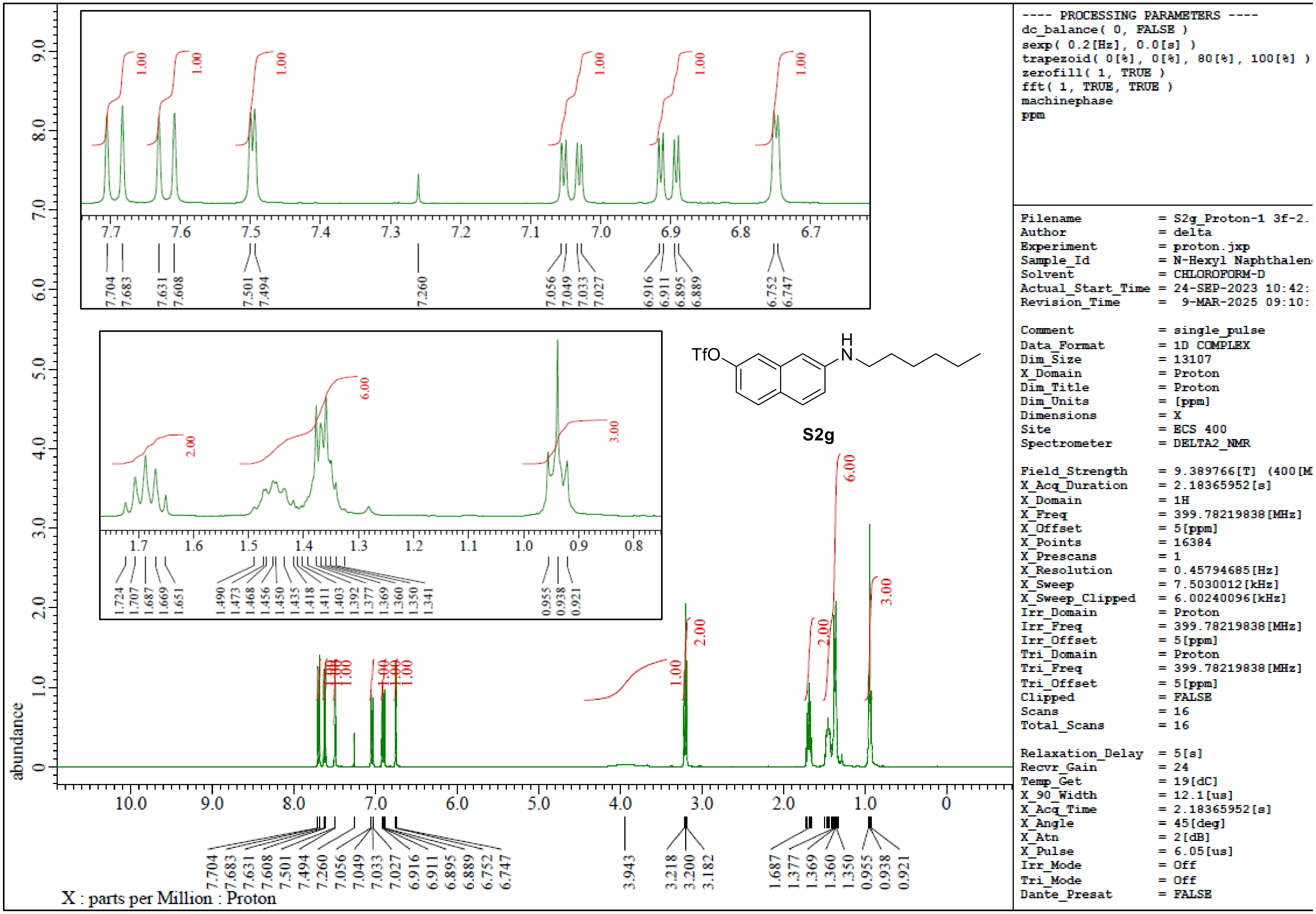

Compound **S2g** (^{1}H NMR, 400 MHz, $CDCl_3$).

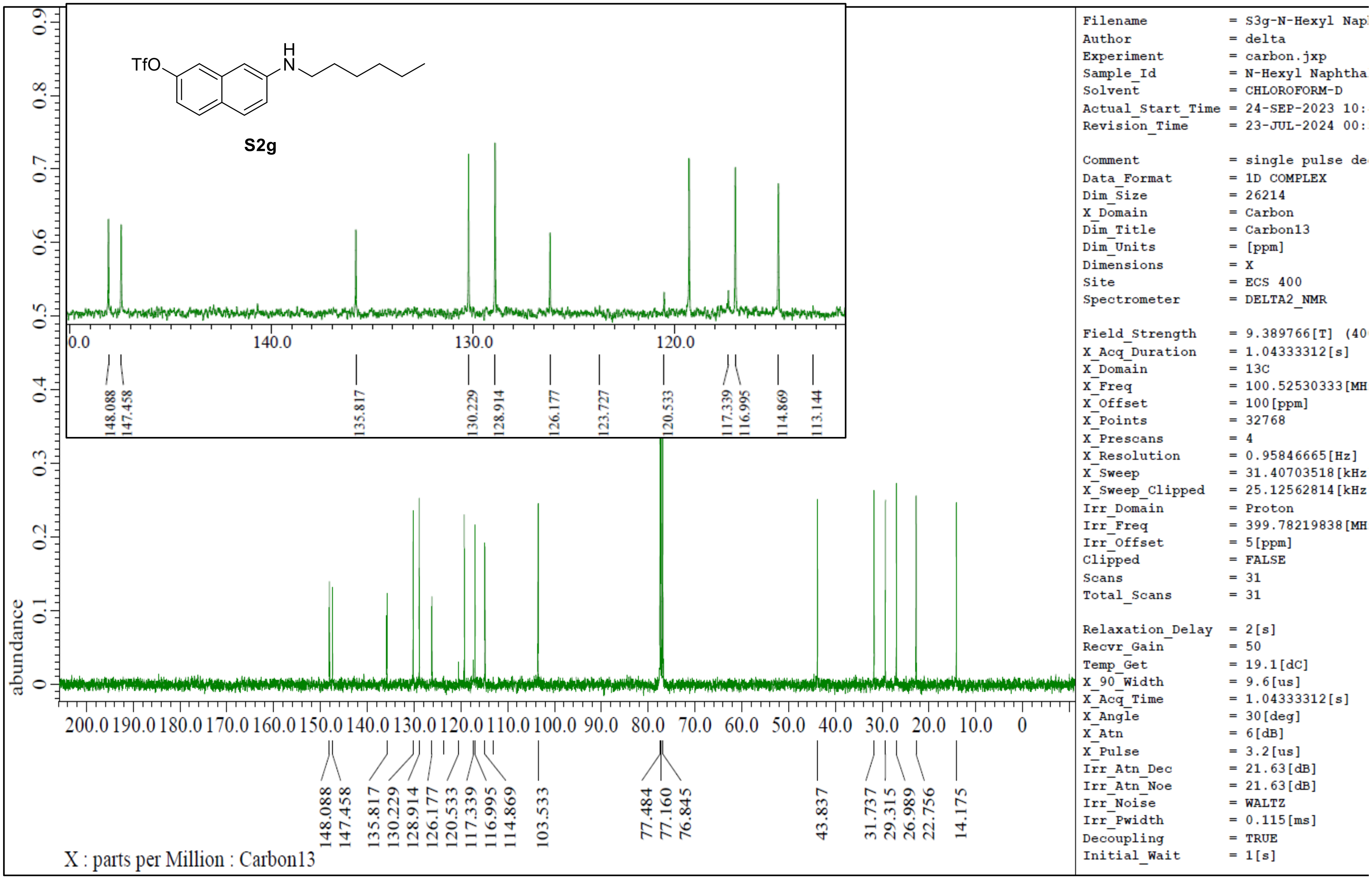

Compound **S2g** (^{13}C NMR, 100 MHz, $CDCl_3$).

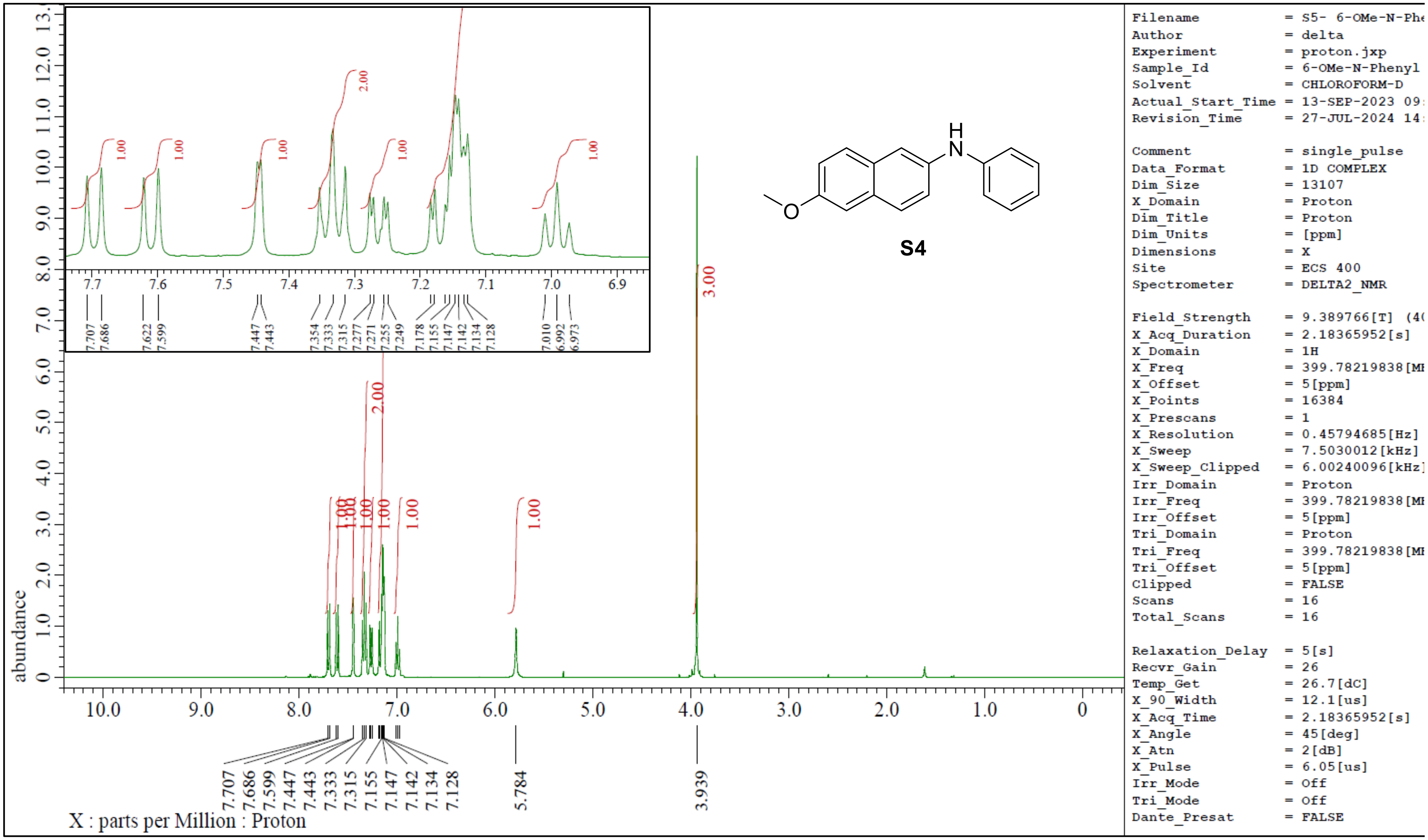

Compound **S4** (^{1}H NMR, 400 MHz, $CDCl_3$).

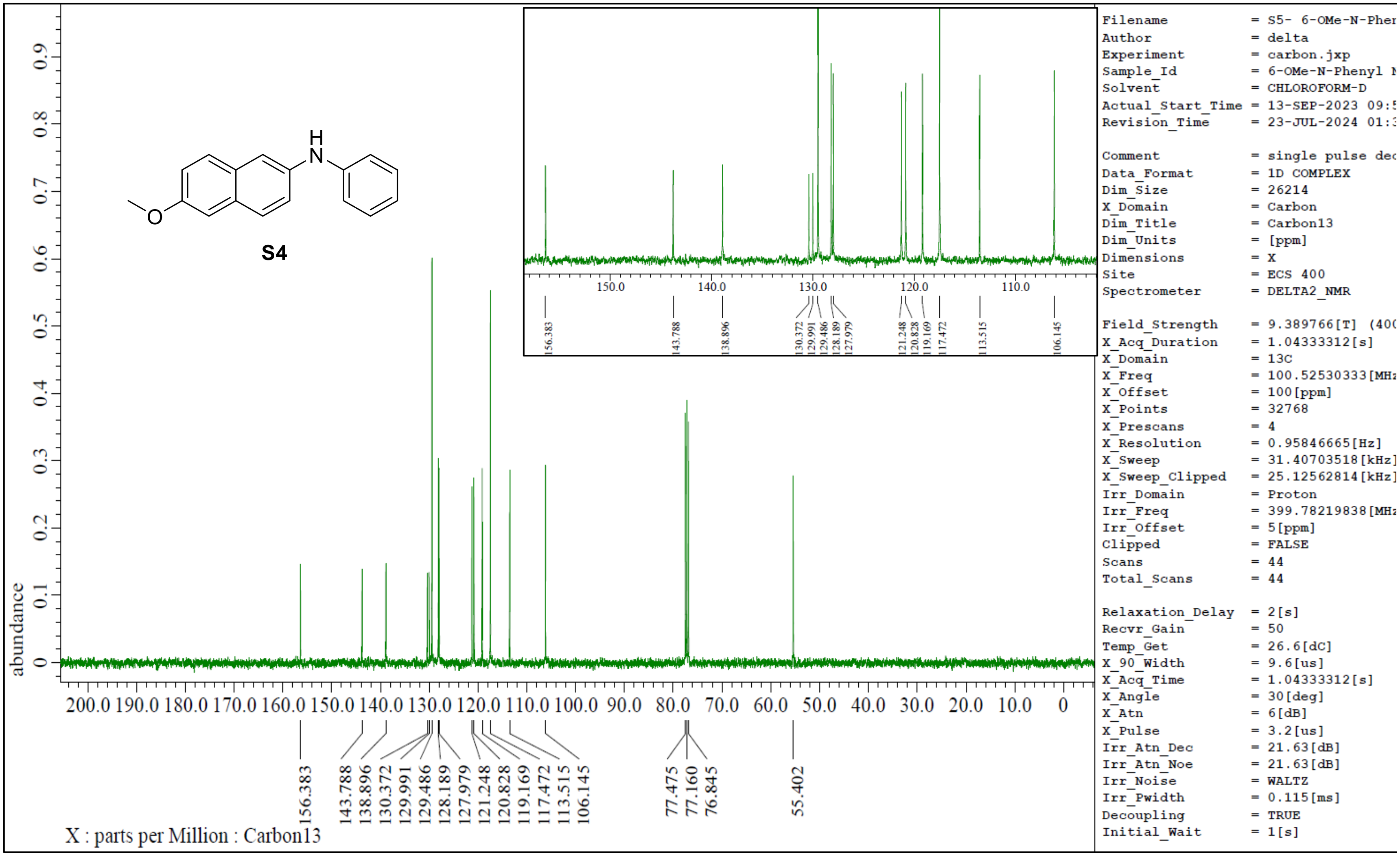

Compound **S4** (^{13}C NMR, 100 MHz, $CDCl_3$).

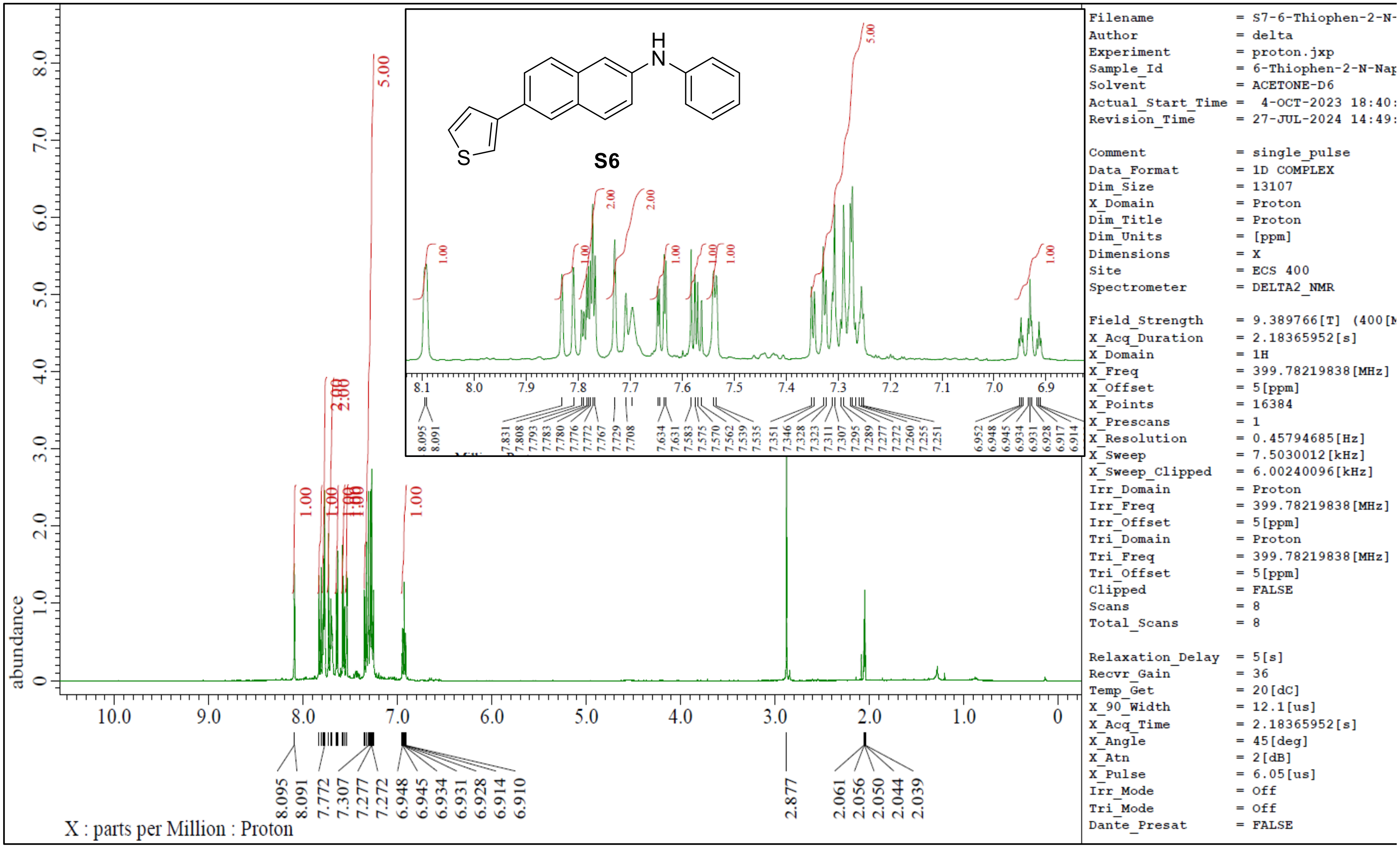

Compound **S6** (^{1}H NMR, 400 MHz, $(CD_3)_2CO$).

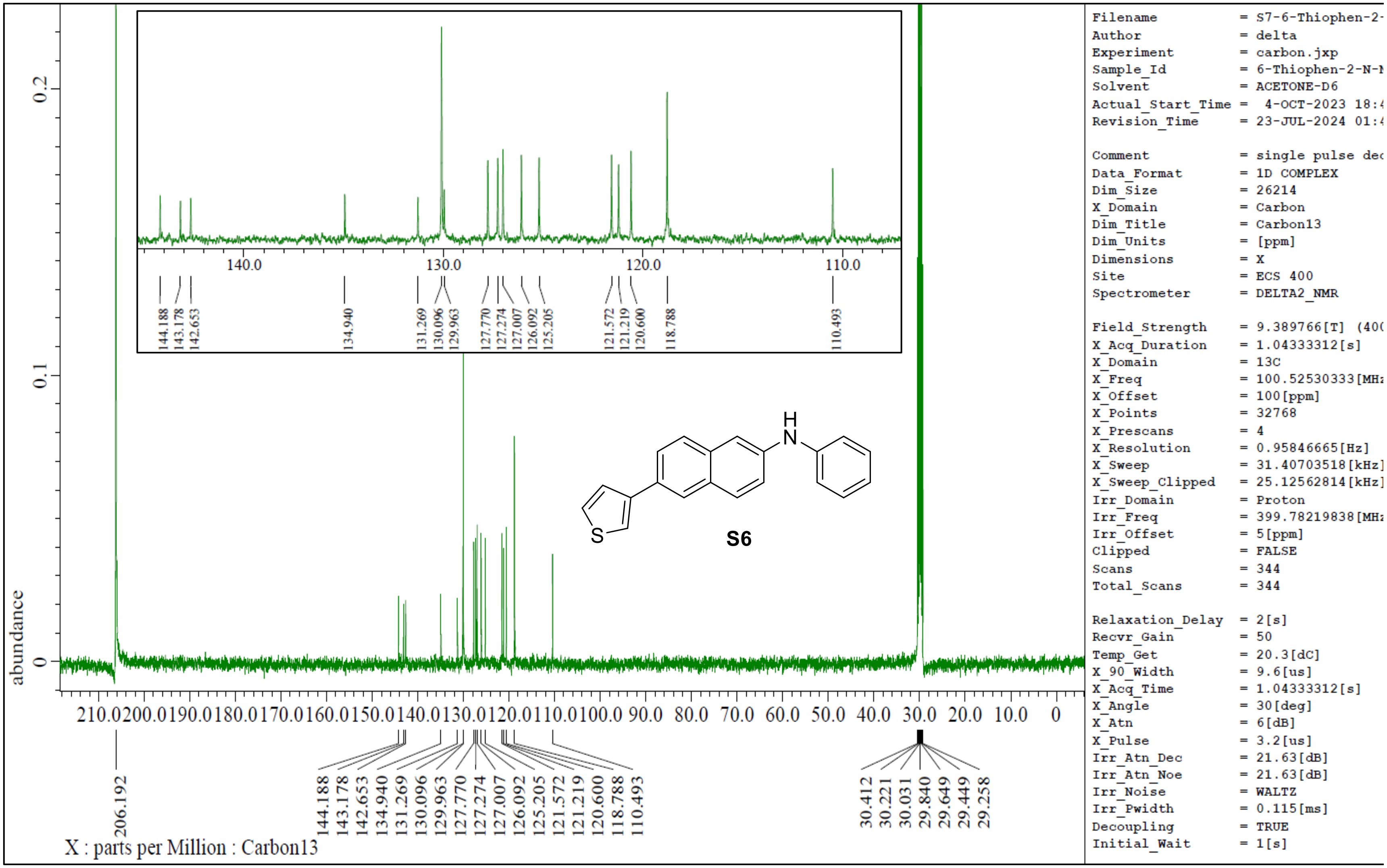

Compound **S6** (^{13}C NMR, 100 MHz, $(CD_3)_2CO$).

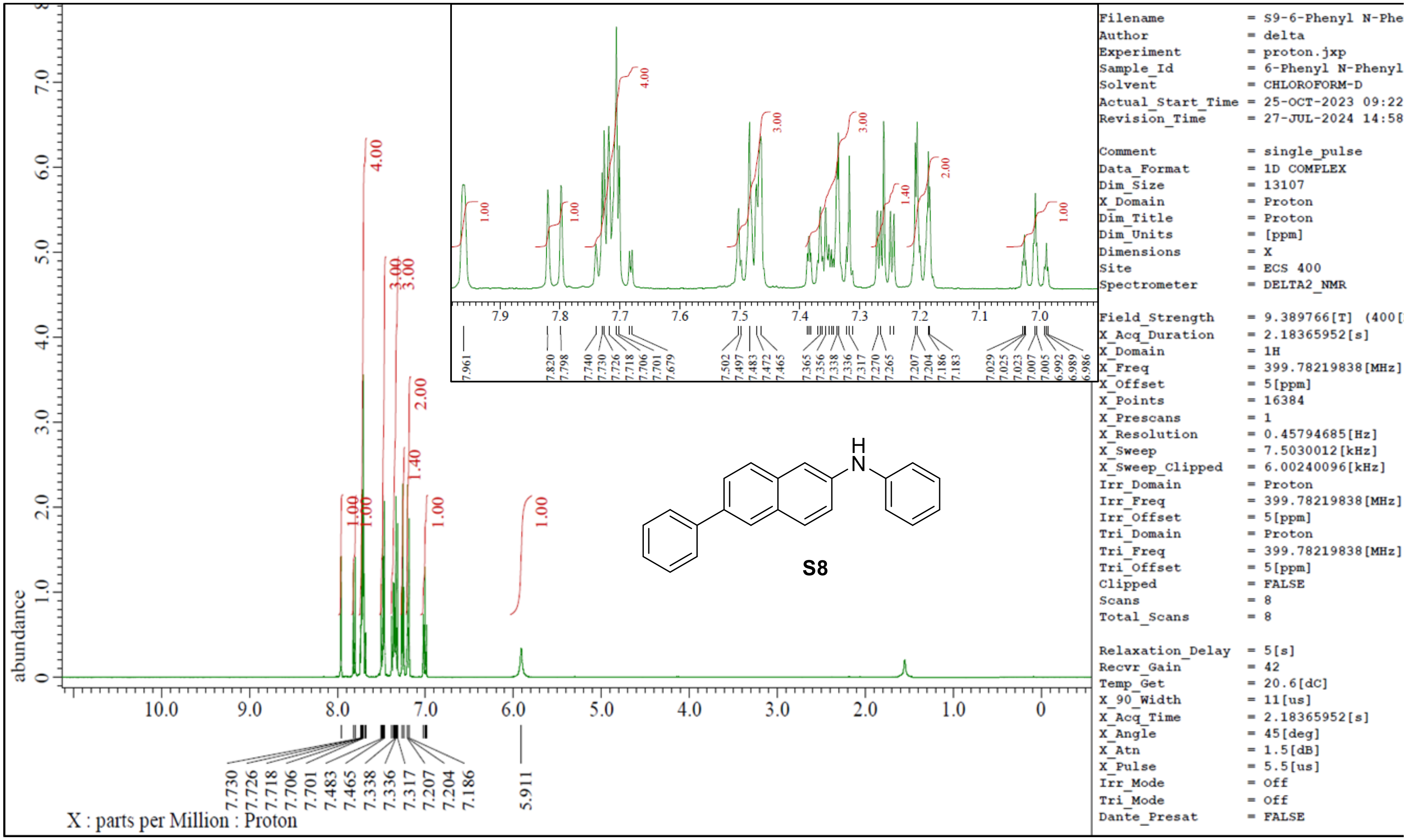

Compound **S8** (^{1}H NMR, 400 MHz, $CDCl_3$).

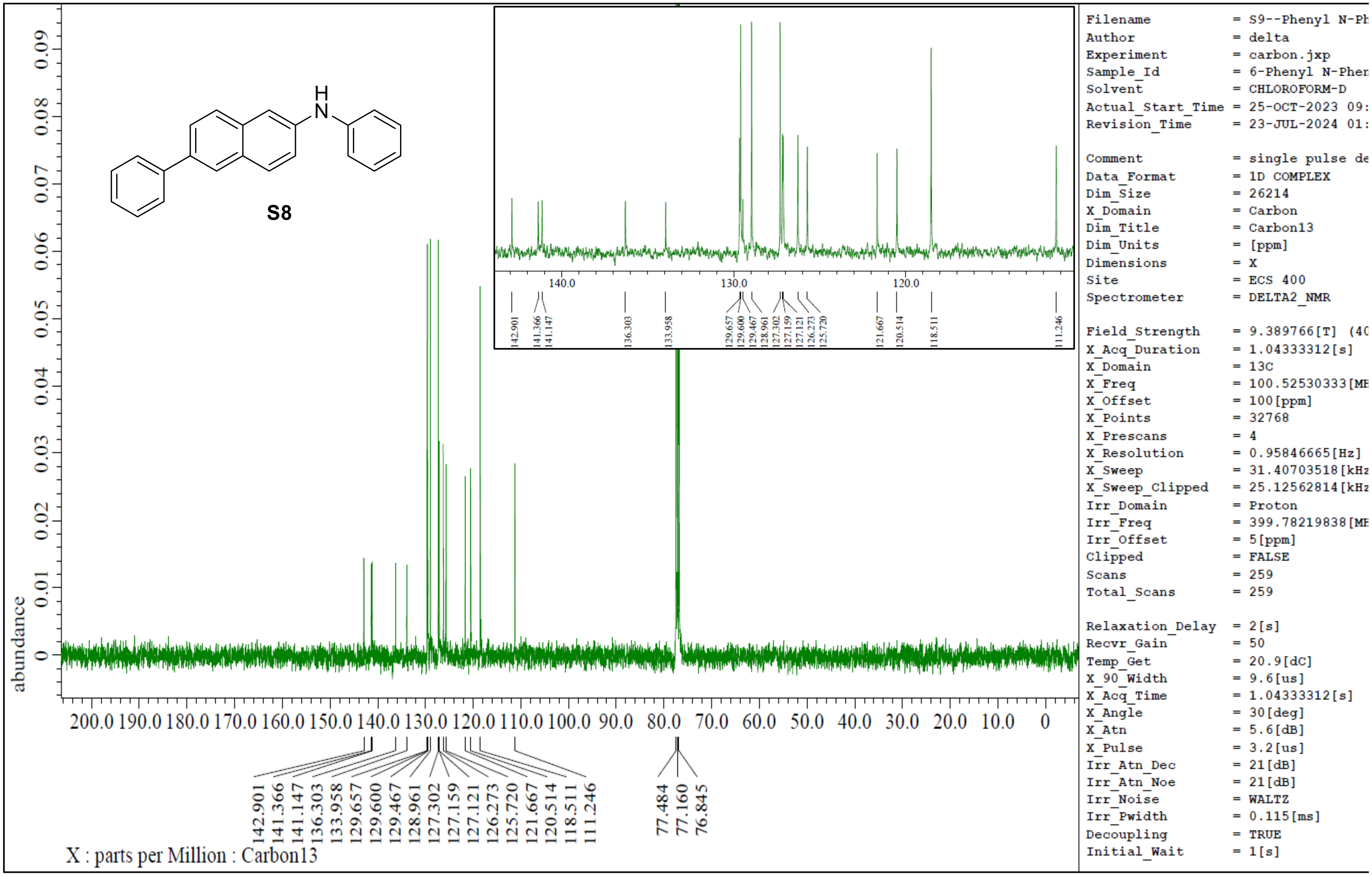

Compound **S8** (^{13}C NMR, 100 MHz, $CDCl_3$).

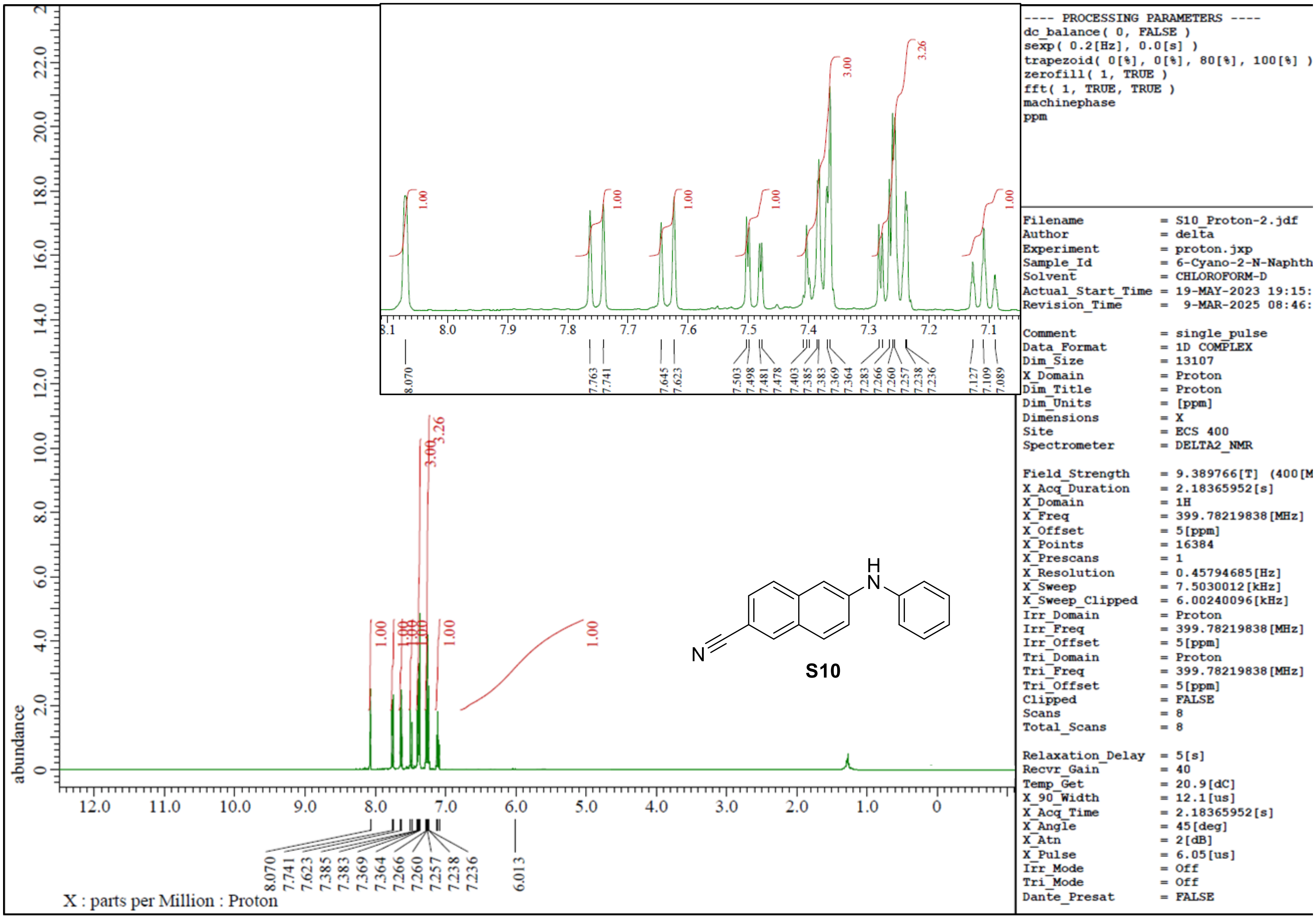

Compound **S10** (^{1}H NMR, 400 MHz, $CDCl_3$).

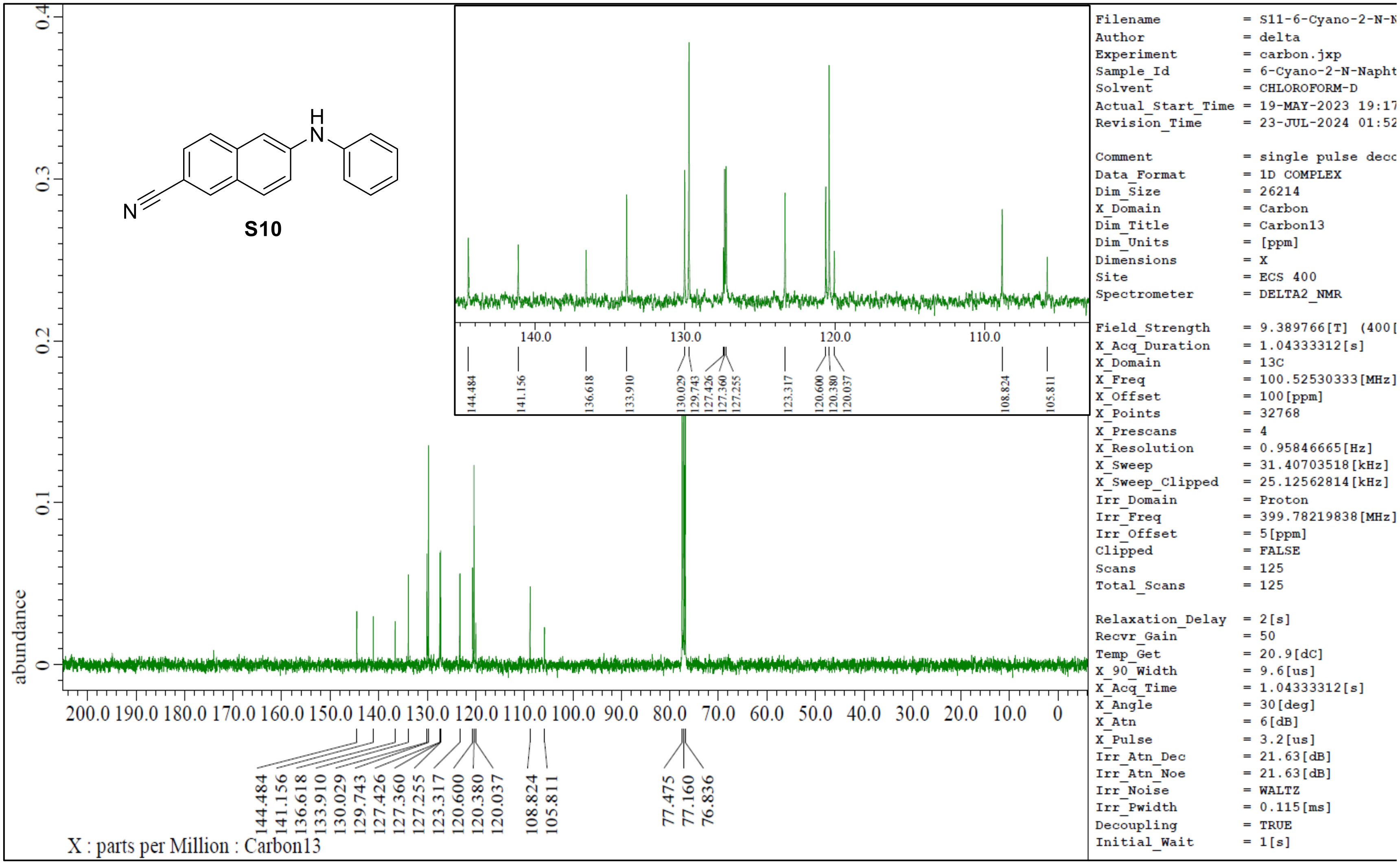

Compound **S10** (^{13}C NMR, 100 MHz, $CDCl_3$).

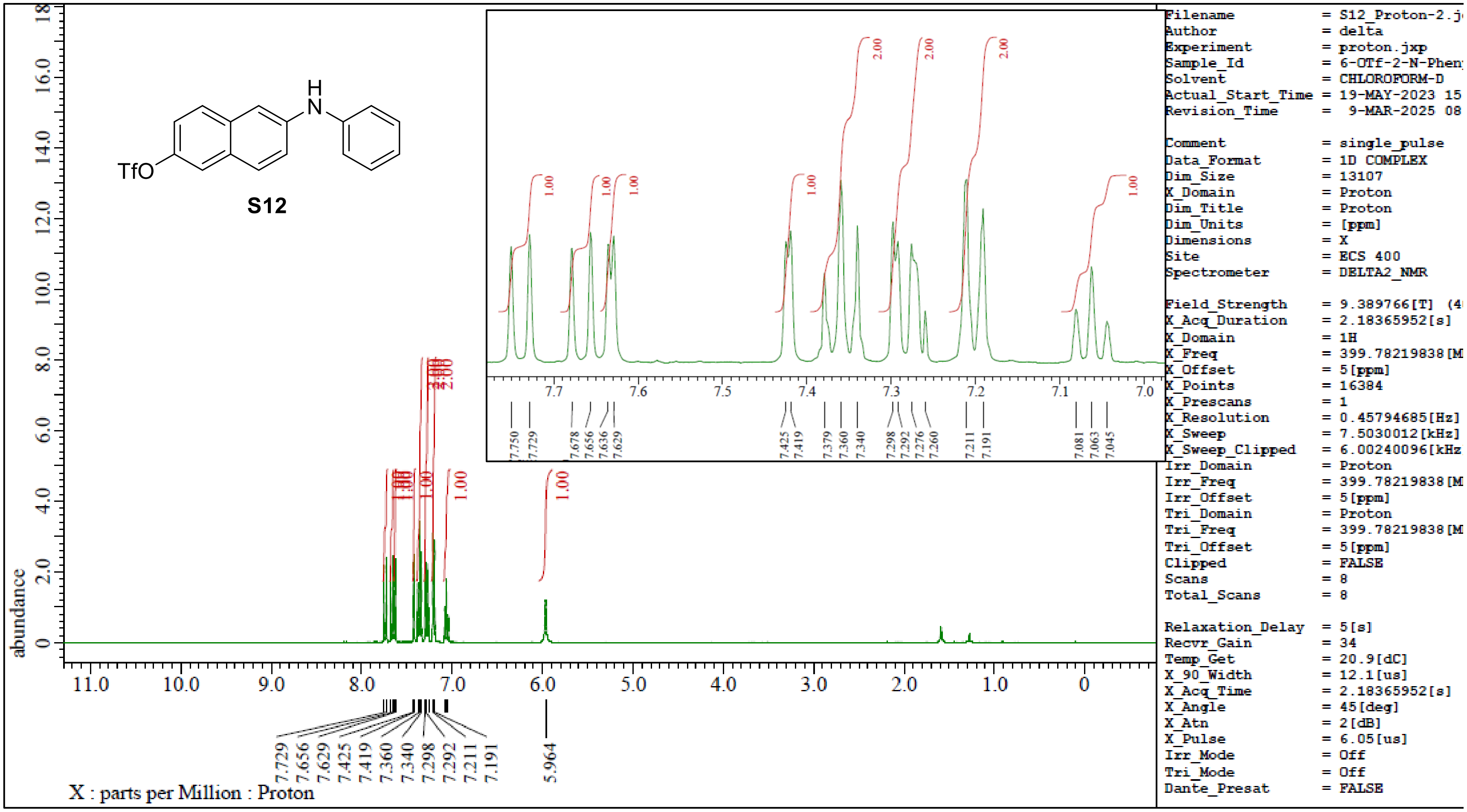

Compound **S12** (^{1}H NMR, 400 MHz, $CDCl_3$).

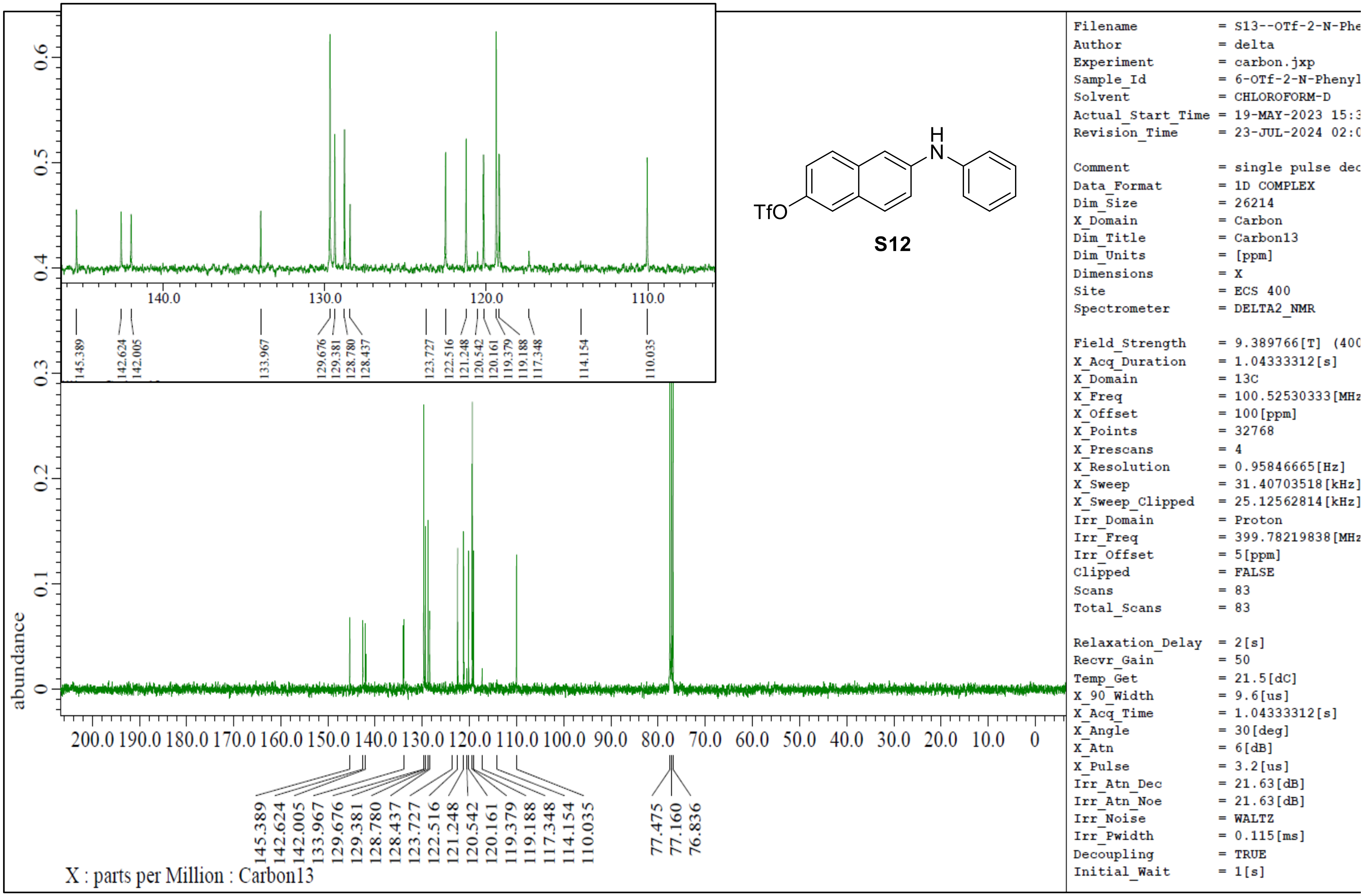

Compound **S12** (^{13}C NMR, 100 MHz, $CDCl_3$).

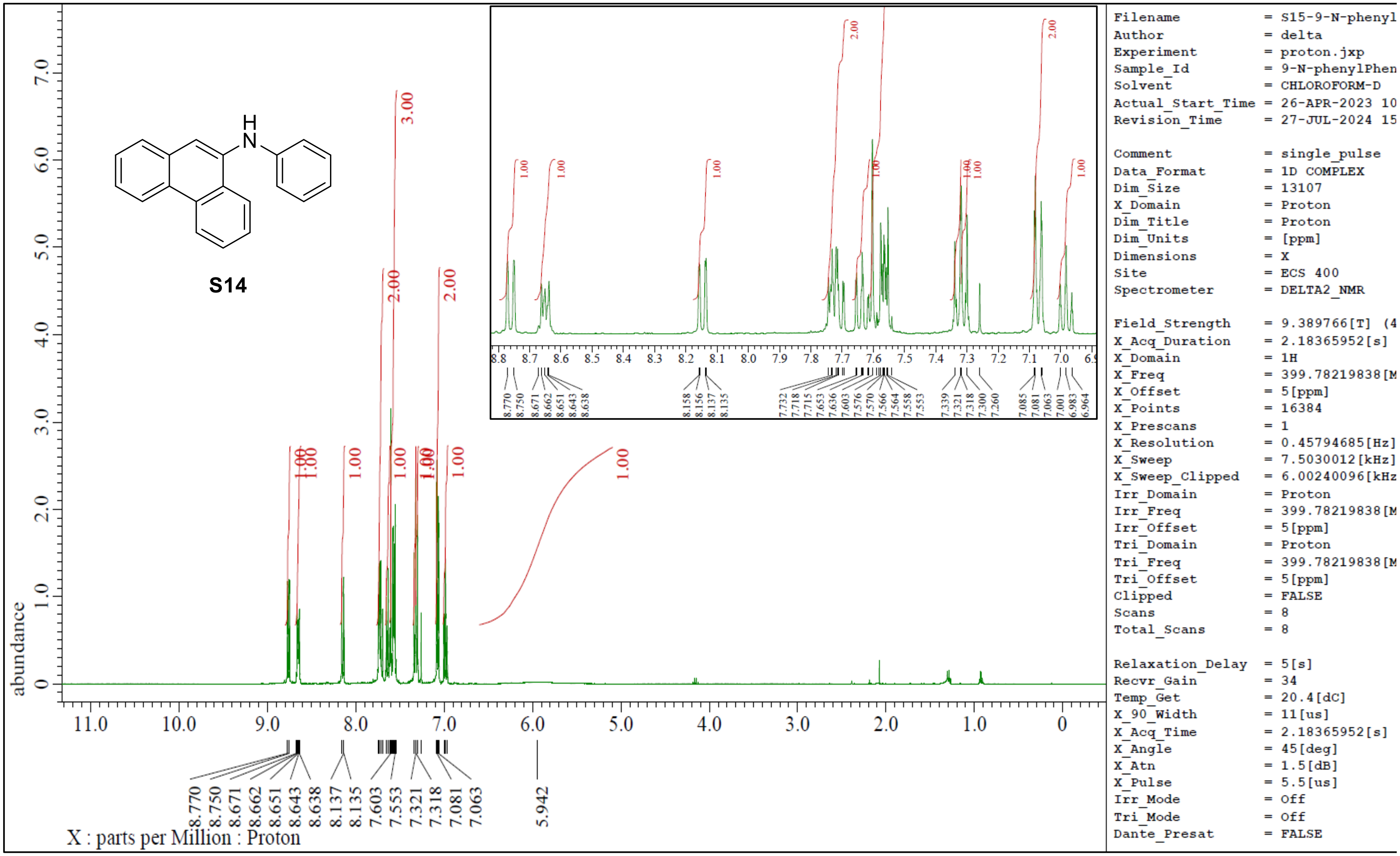

Compound **S14** (^{1}H NMR, 400 MHz, $CDCl_3$).

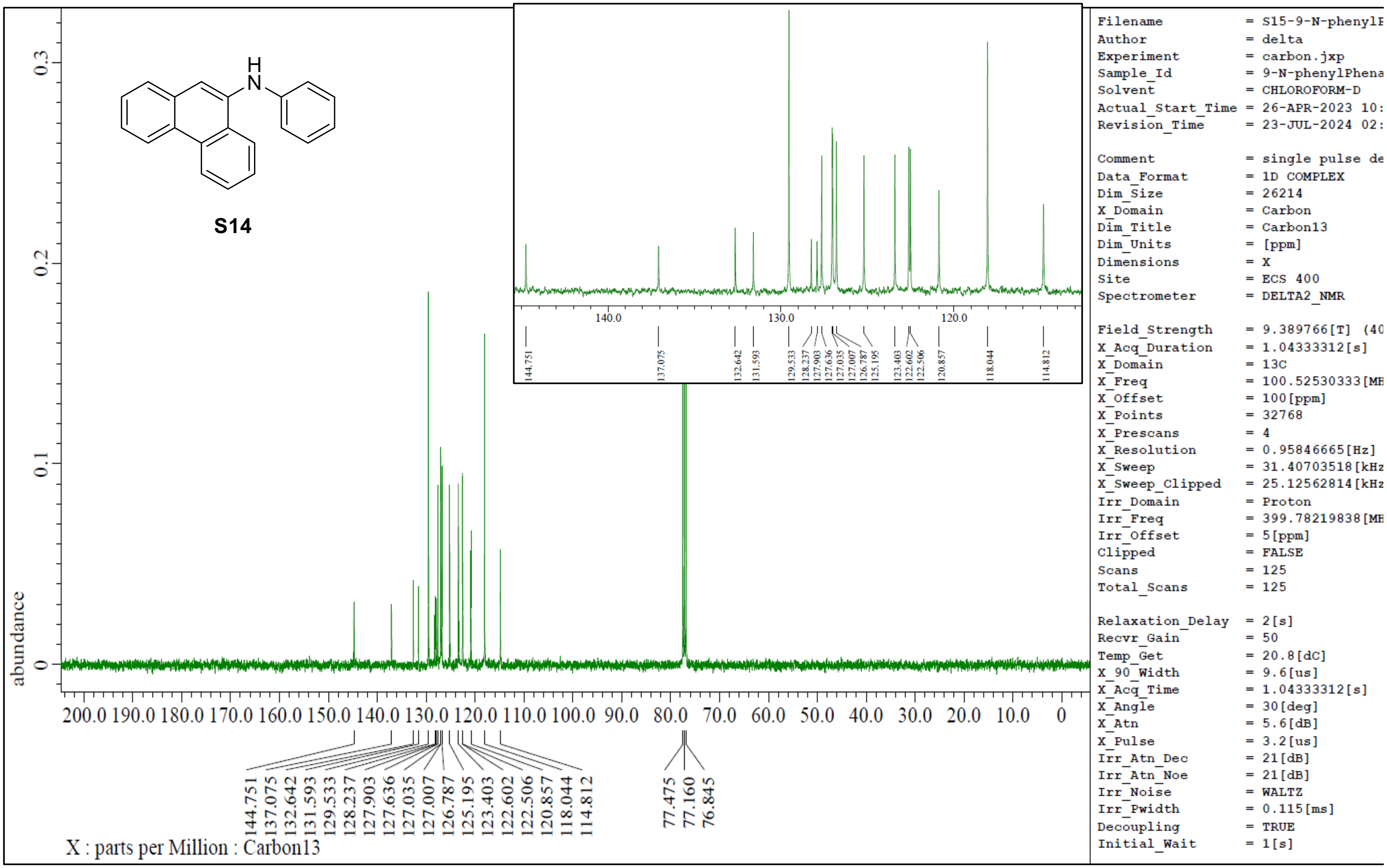

Compound **S14** (^{13}C NMR, 100 MHz, $CDCl_3$).

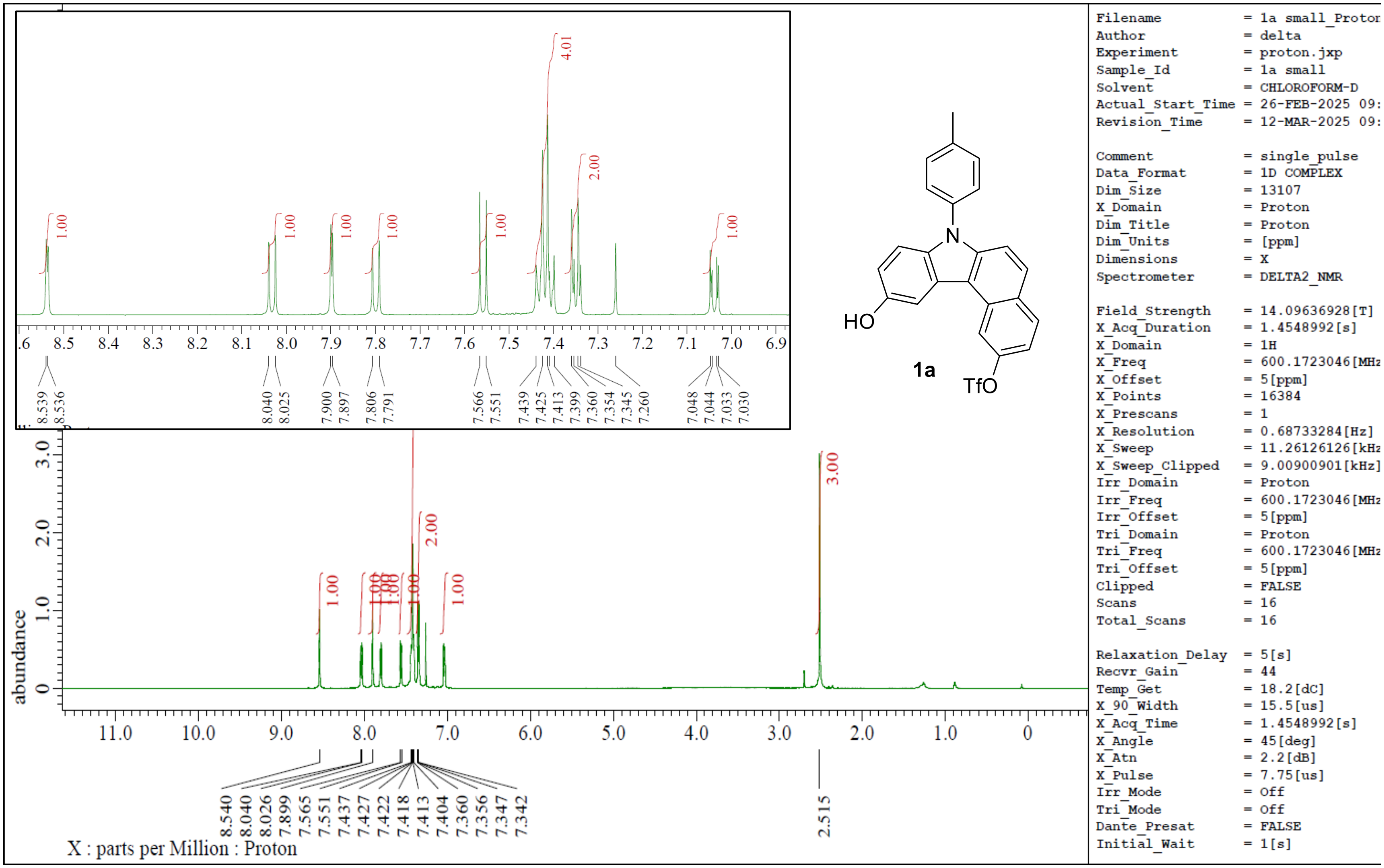

Compound **1a** (^{1}H NMR, 600 MHz, $CDCl_3$).

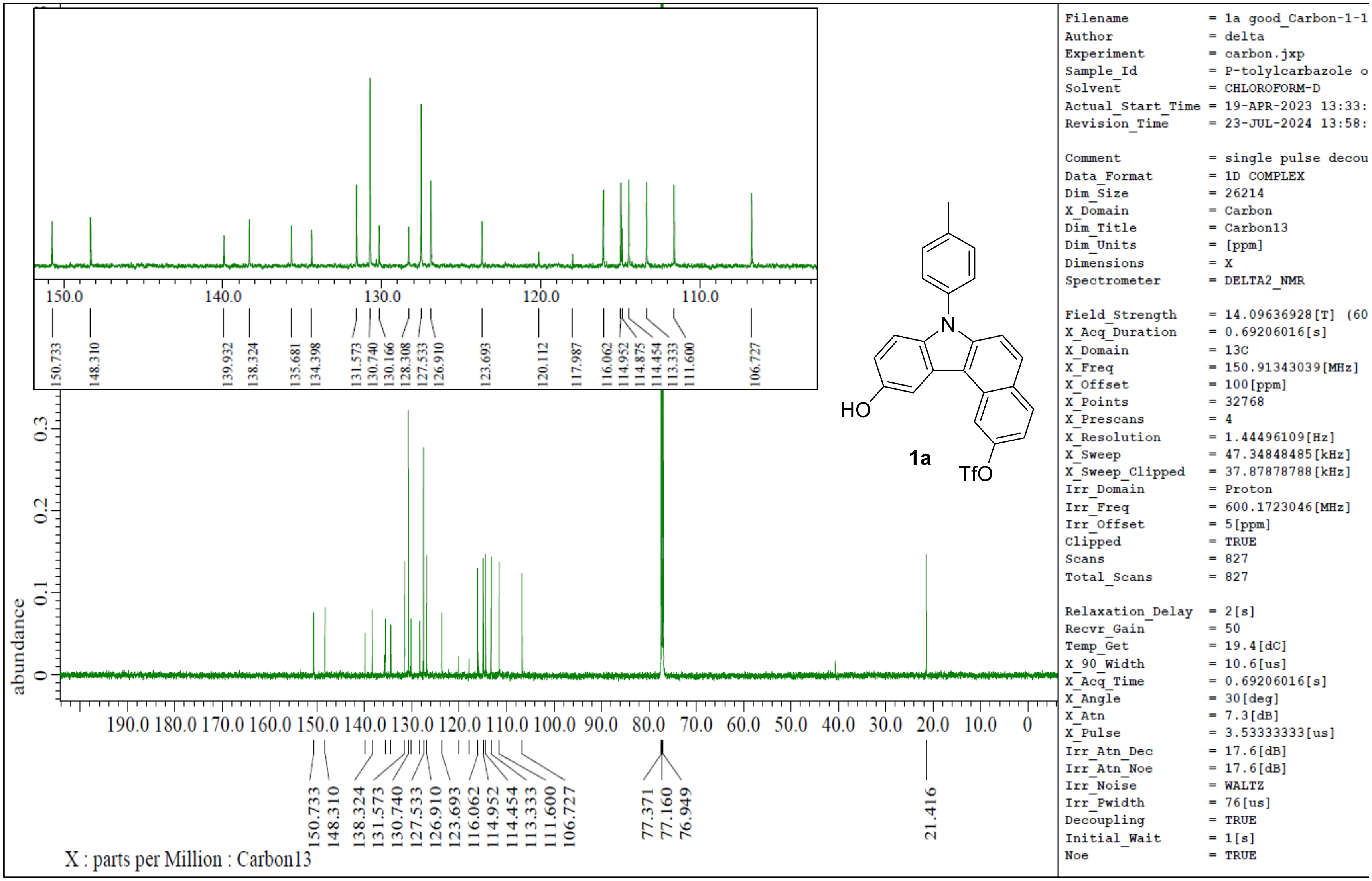

Compound **1a** (^{13}C NMR, 150 MHz, $CDCl_3$).

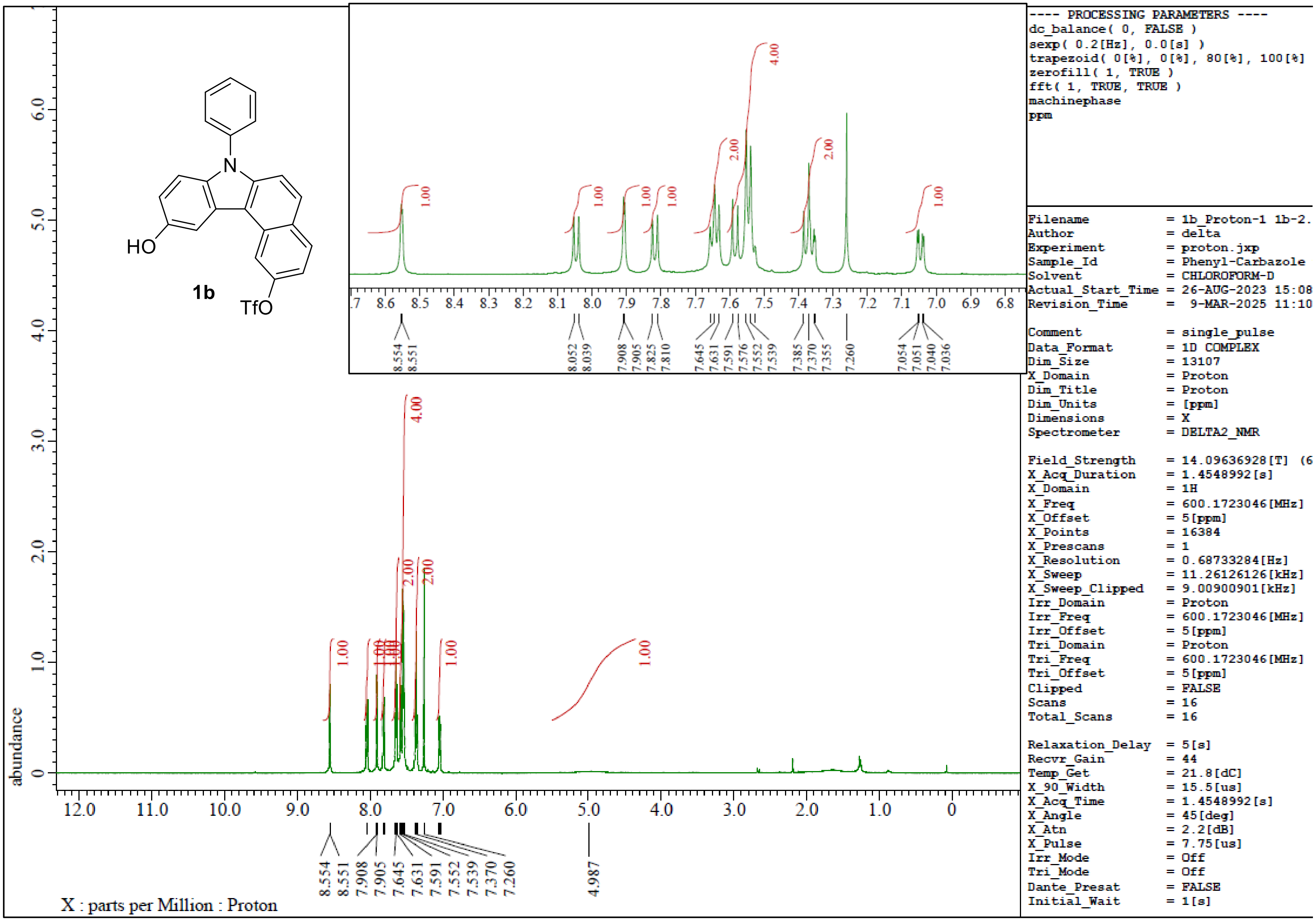

Compound **1b** (^{1}H NMR, 600 MHz, $CDCl_3$).

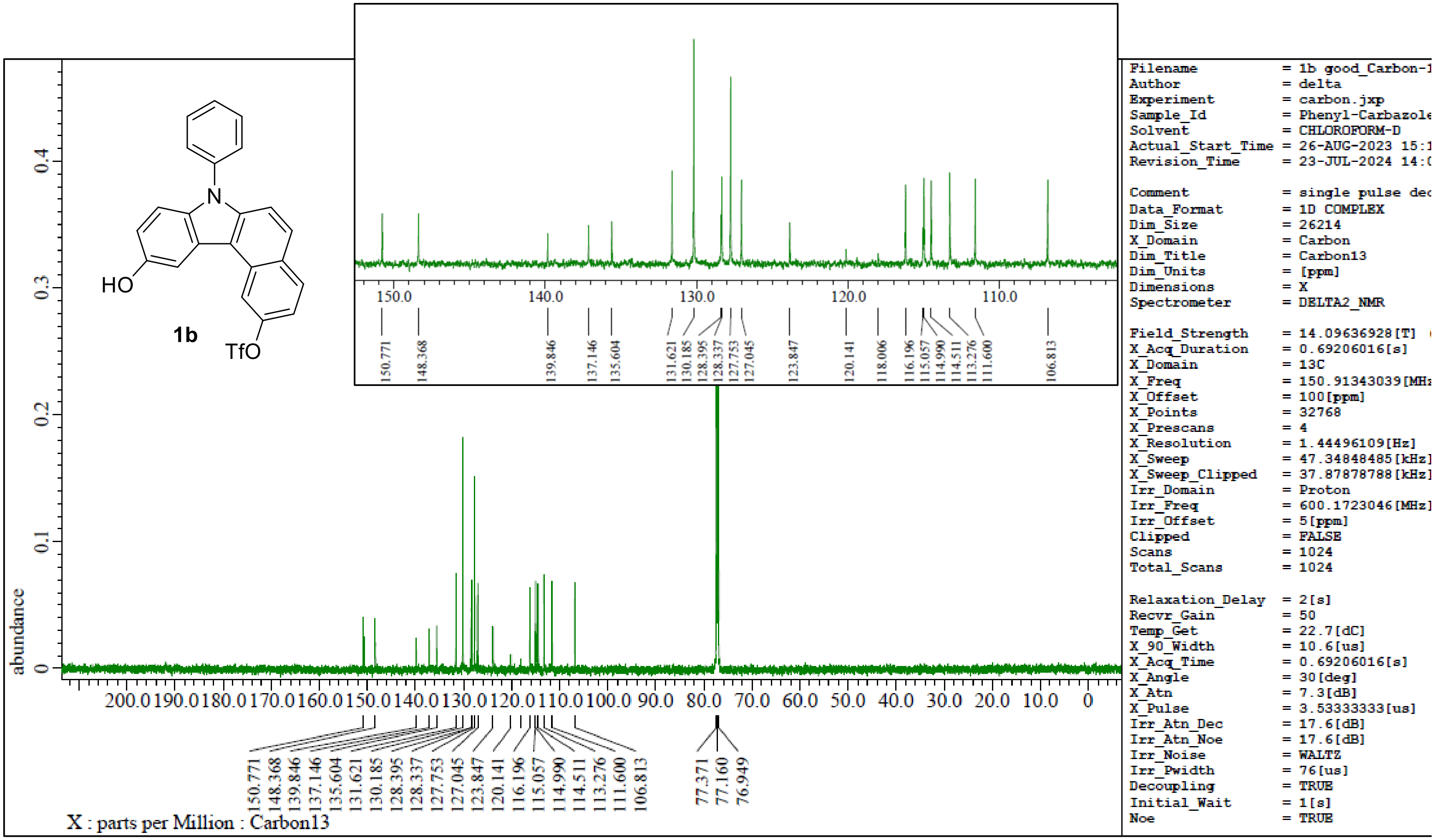

Compound **1b** (^{13}C NMR, 150 MHz, $CDCl_3$).

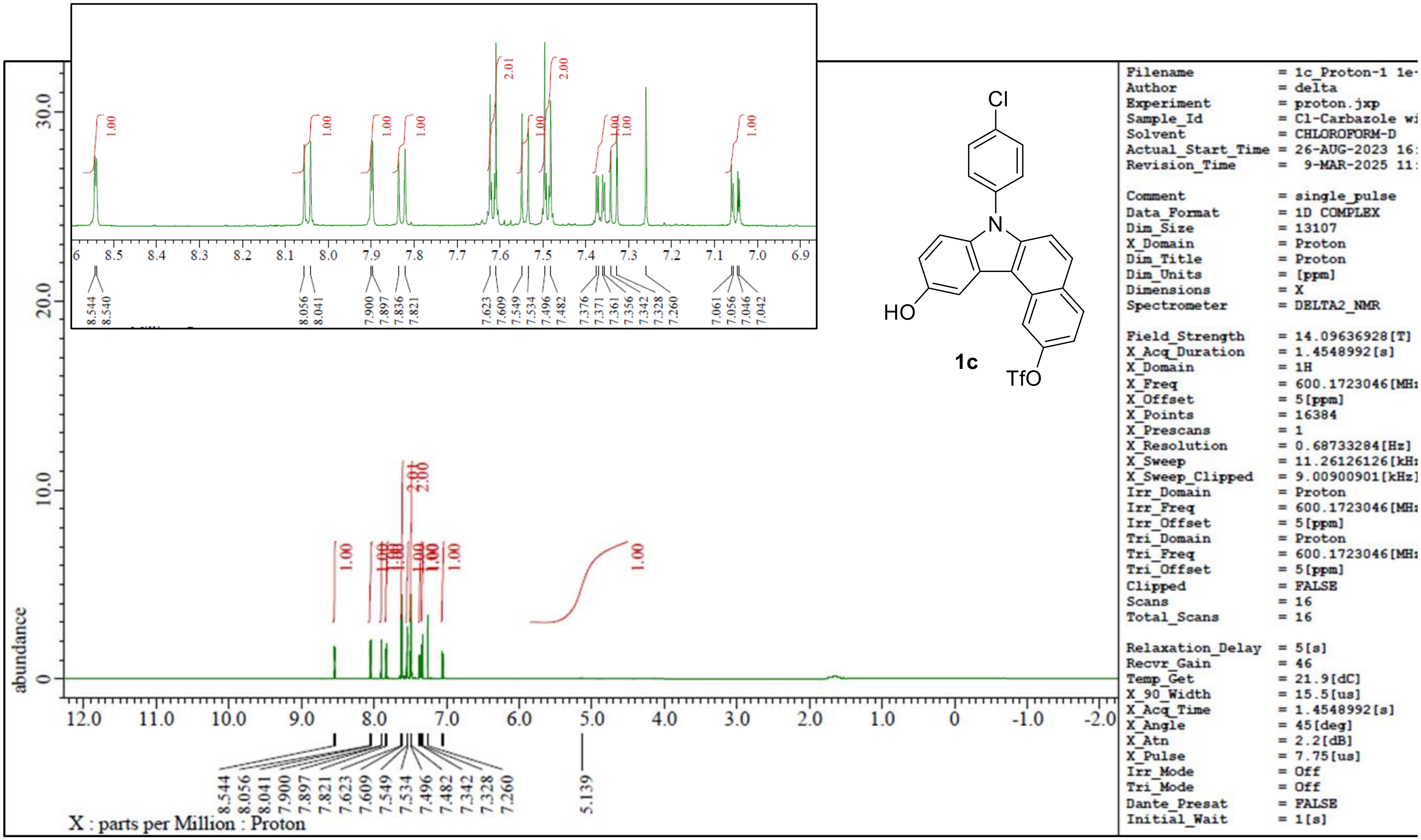

Compound **1c** (^{1}H NMR, 600 MHz, $CDCl_3$).

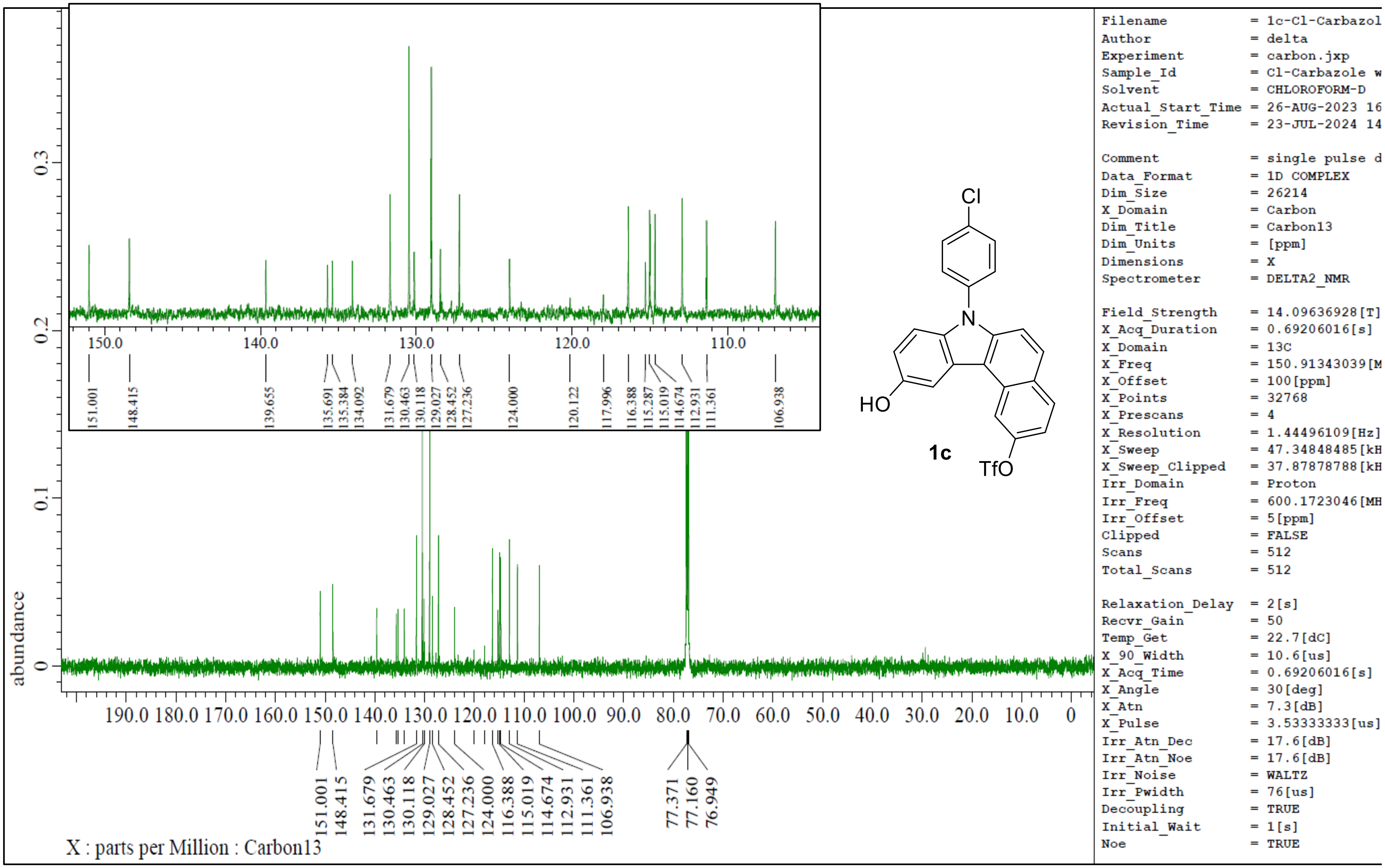

Compound **1c** (^{13}C NMR, 150 MHz, $CDCl_3$).

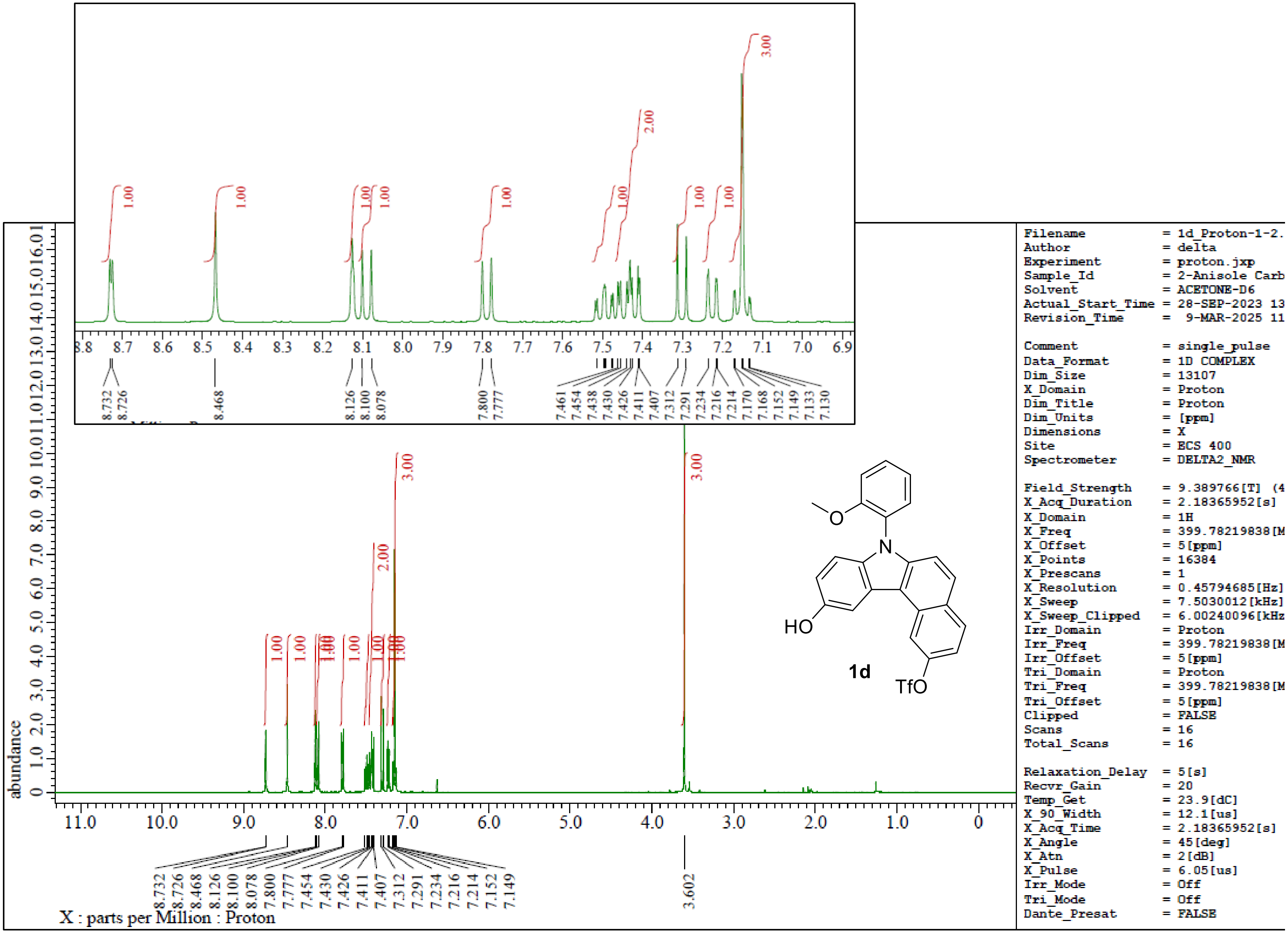

Compound **1d** (^{1}H NMR, 400 MHz, $(CD_3)_2CO$).

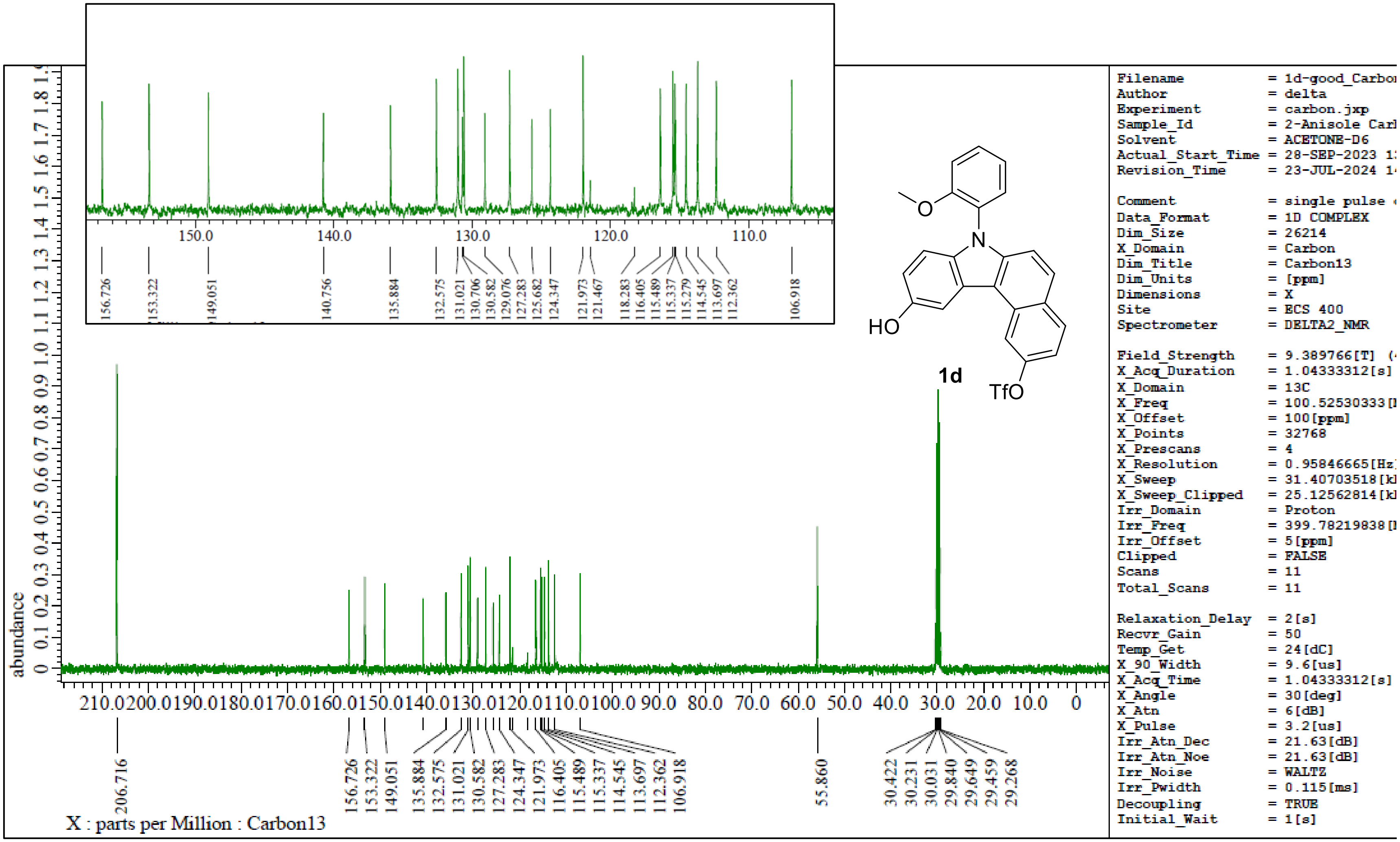

Compound **1d** (^{13}C NMR, 100 MHz, $(CD_3)_2CO$).

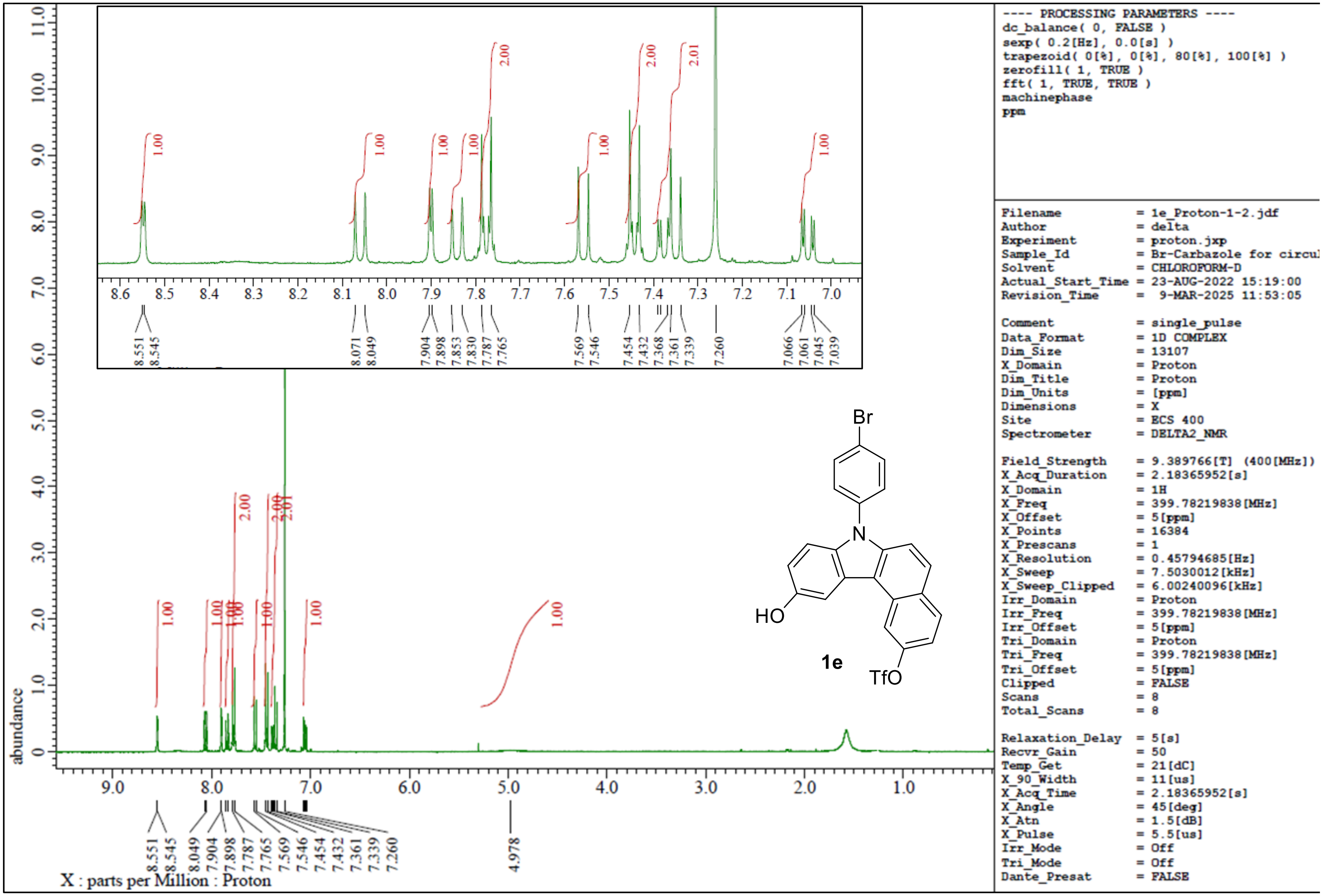

Compound **1e** (^1H NMR, 400 MHz, $CDCl_3$).

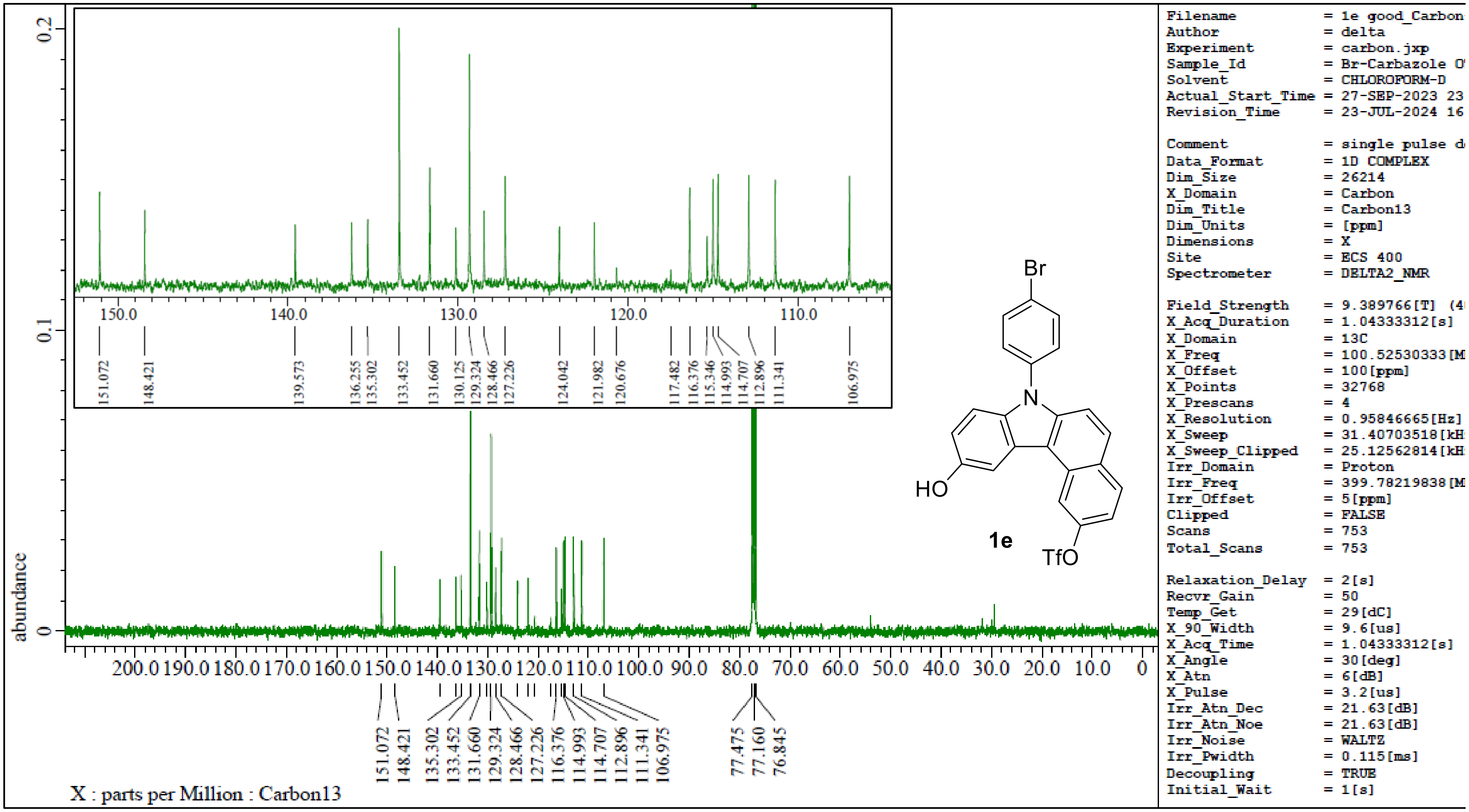

Compound **1e** (^{13}C NMR, 100 MHz, $CDCl_3$).

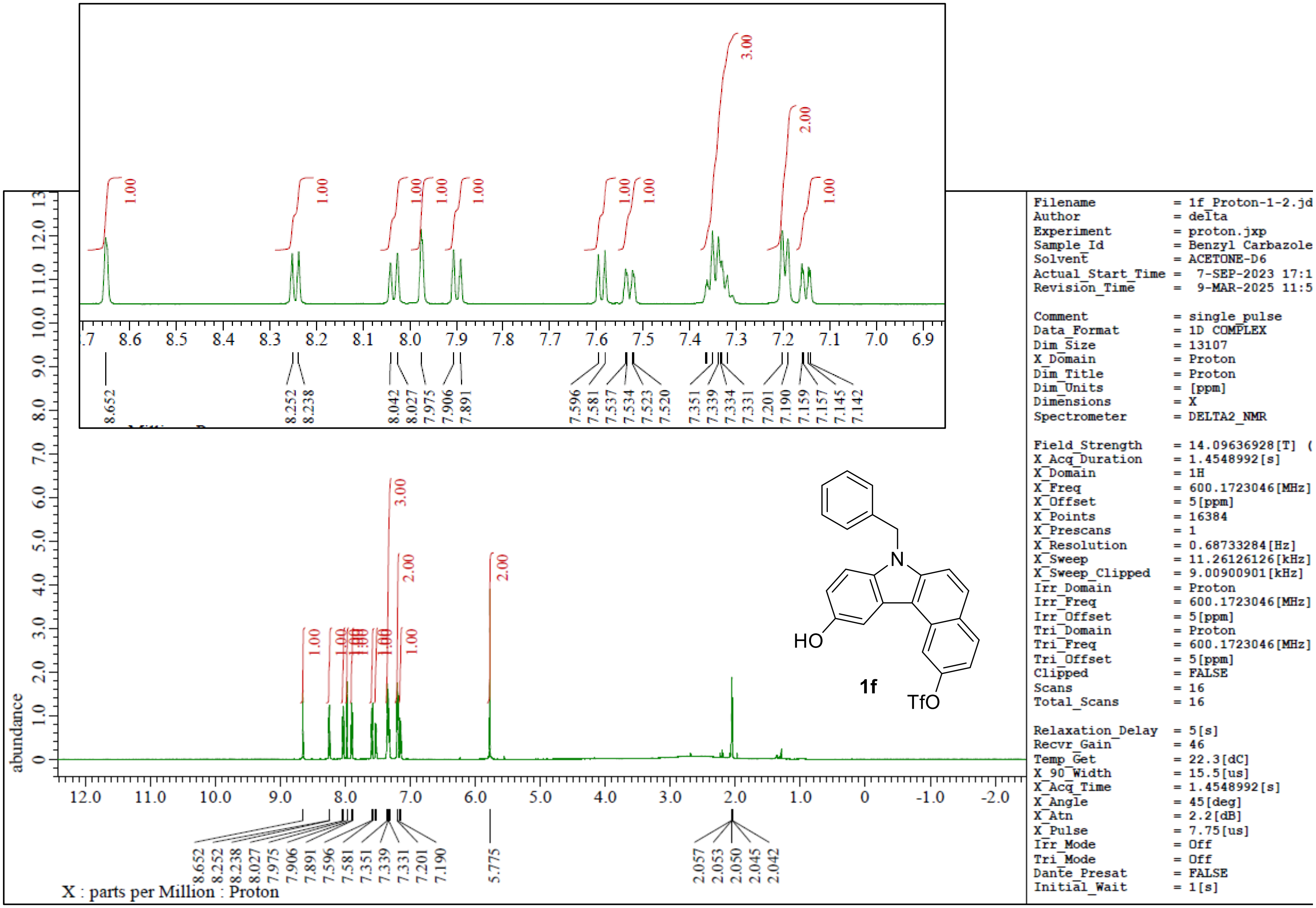

Compound **1f** (^{1}H NMR, 600 MHz, $(CD_3)_2CO$).

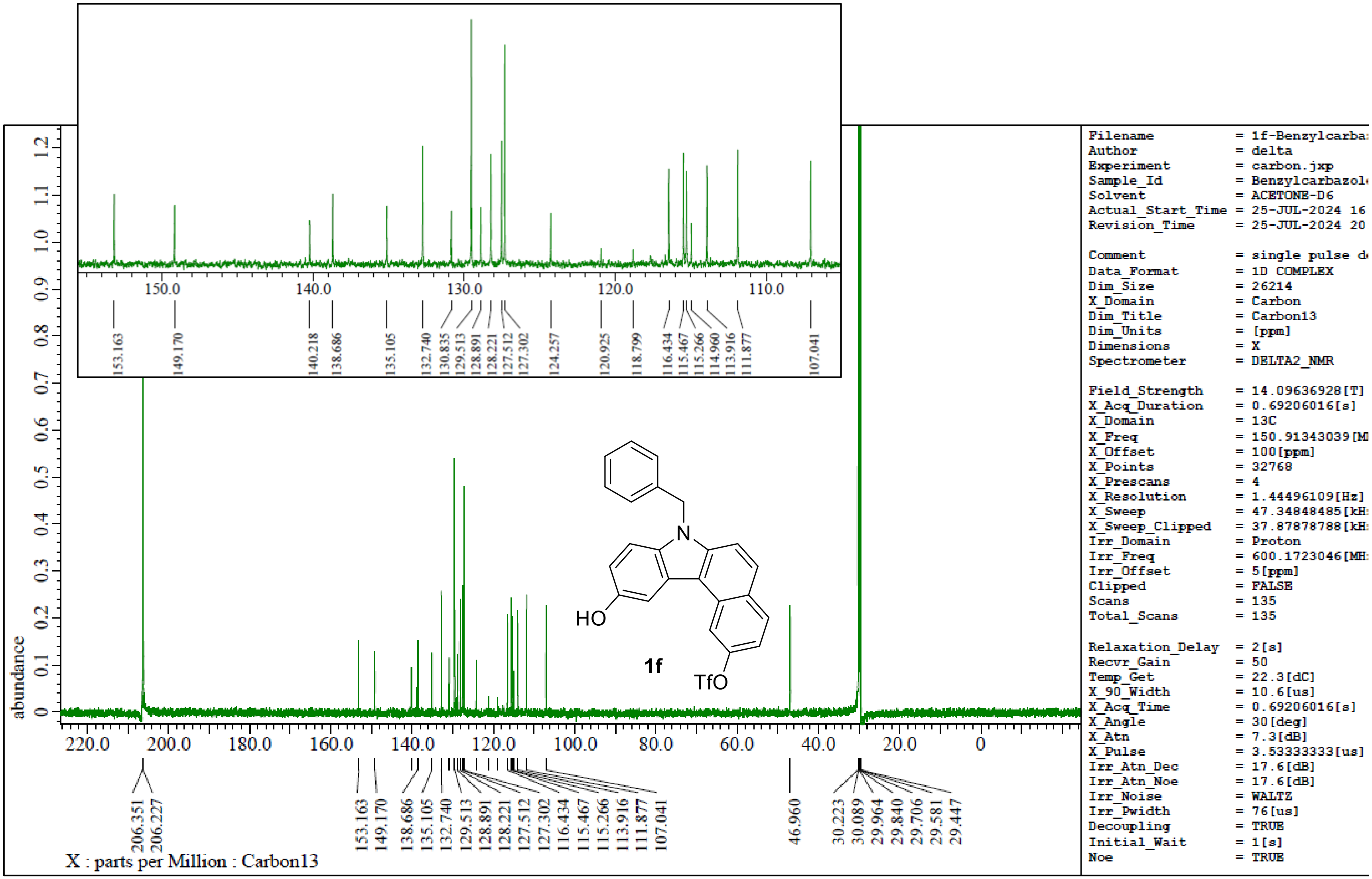

Compound **1f** (^{13}C NMR, 150 MHz, $(CD_3)_2CO$).

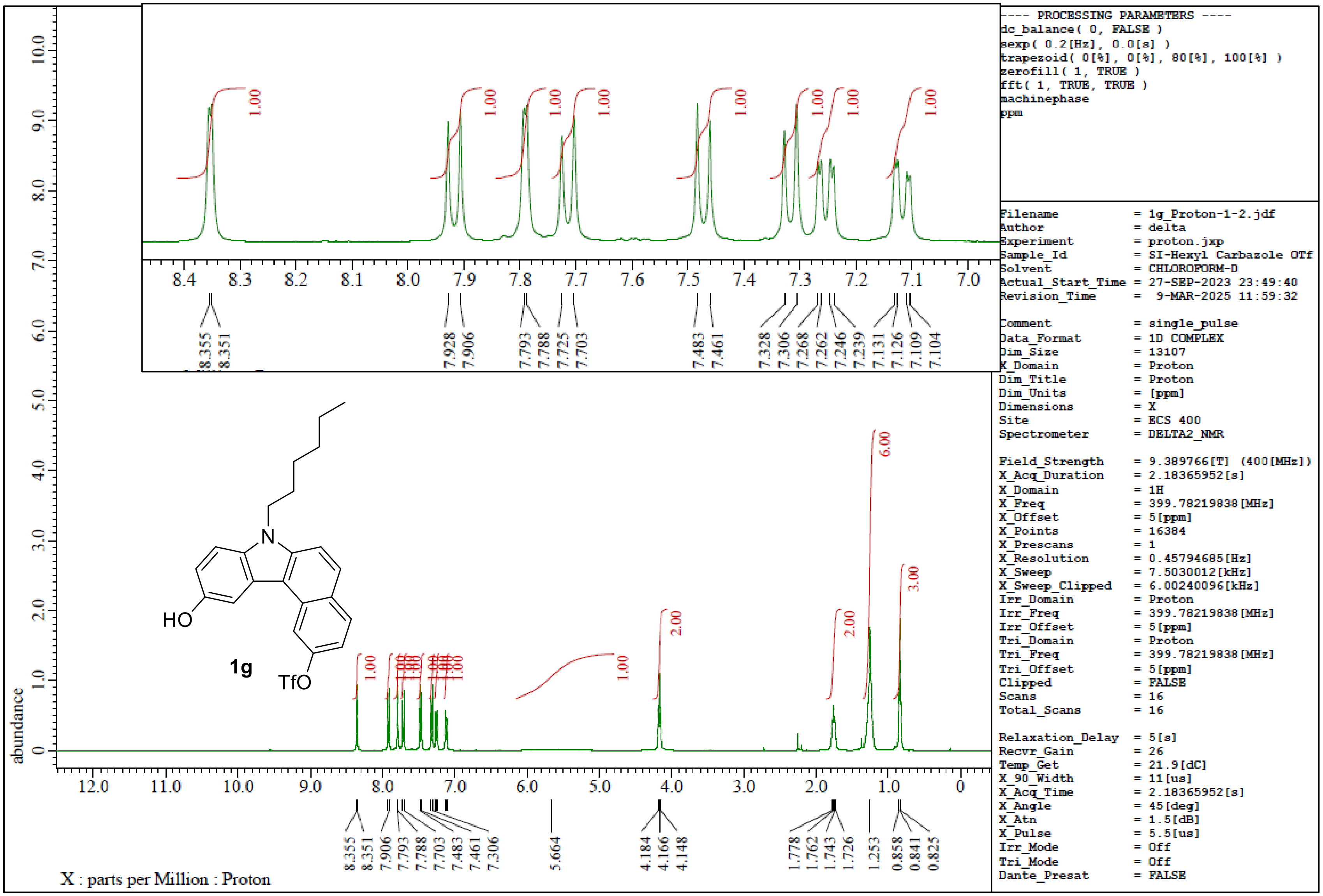

Compound **1g** (^{1}H NMR, 400 MHz, $CDCl_3$).

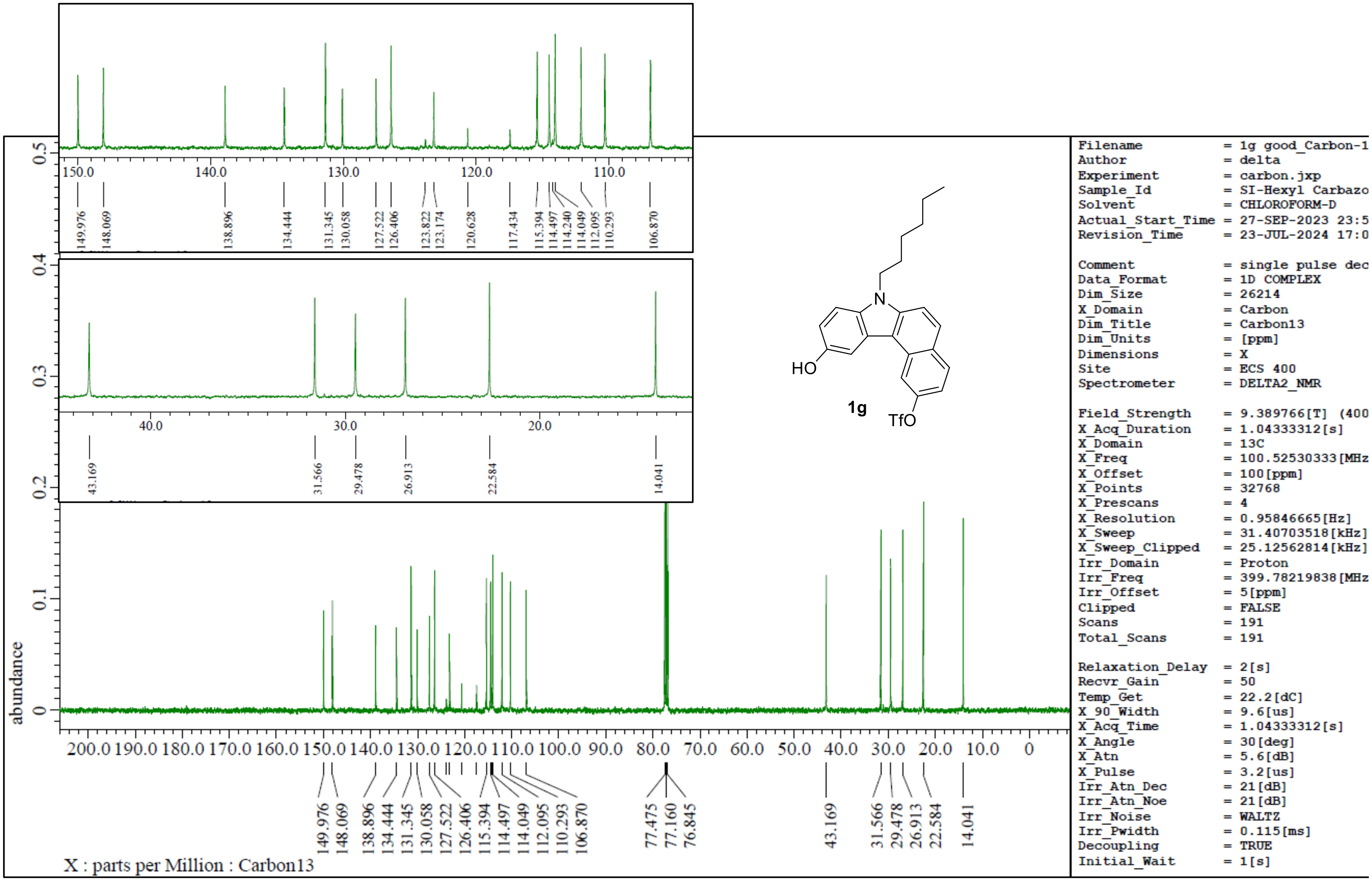

Compound **1g** (^{13}C NMR, 100 MHz, $CDCl_3$).

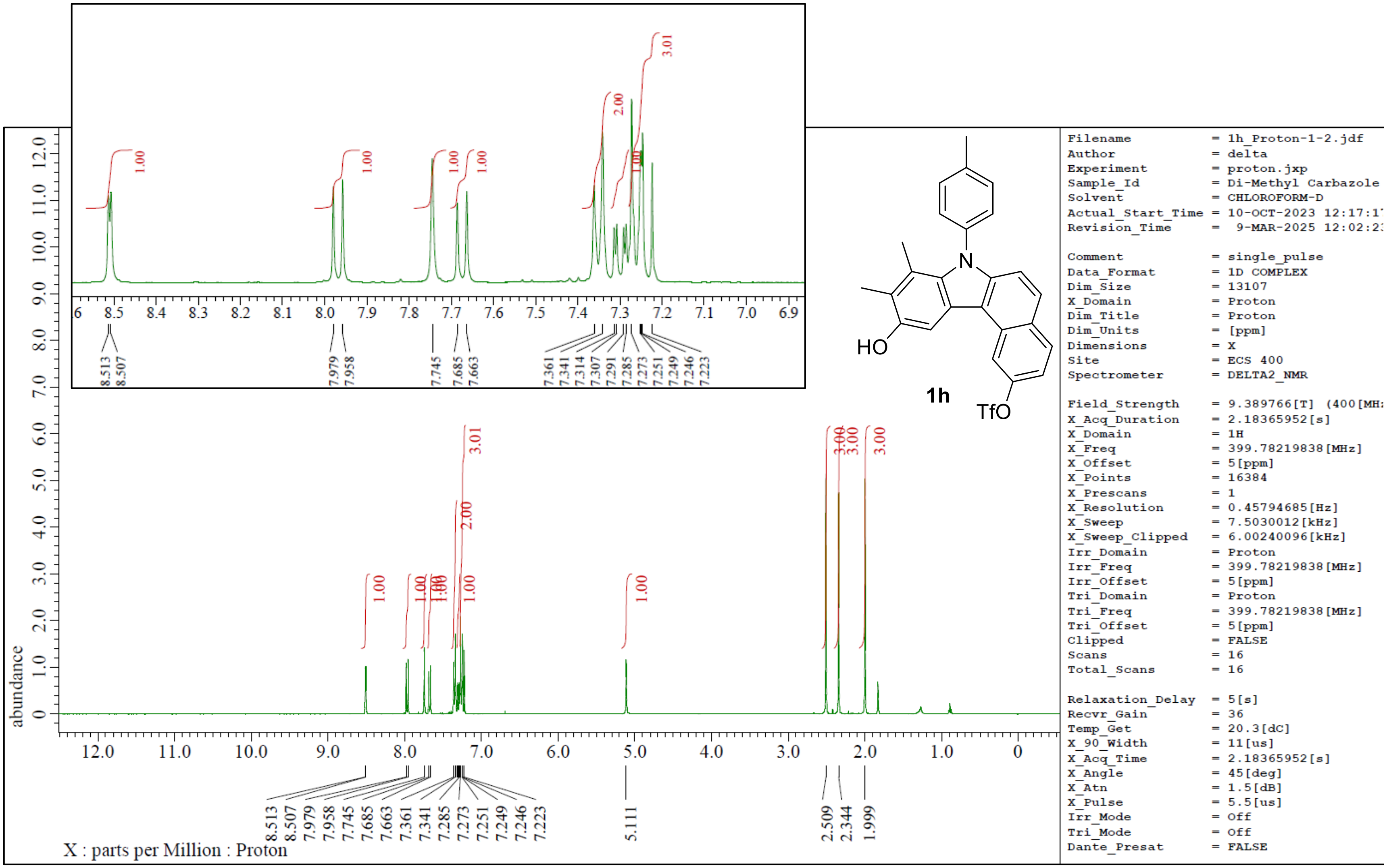

Compound **1h** (^{1}H NMR, 400 MHz, $CDCl_3$).

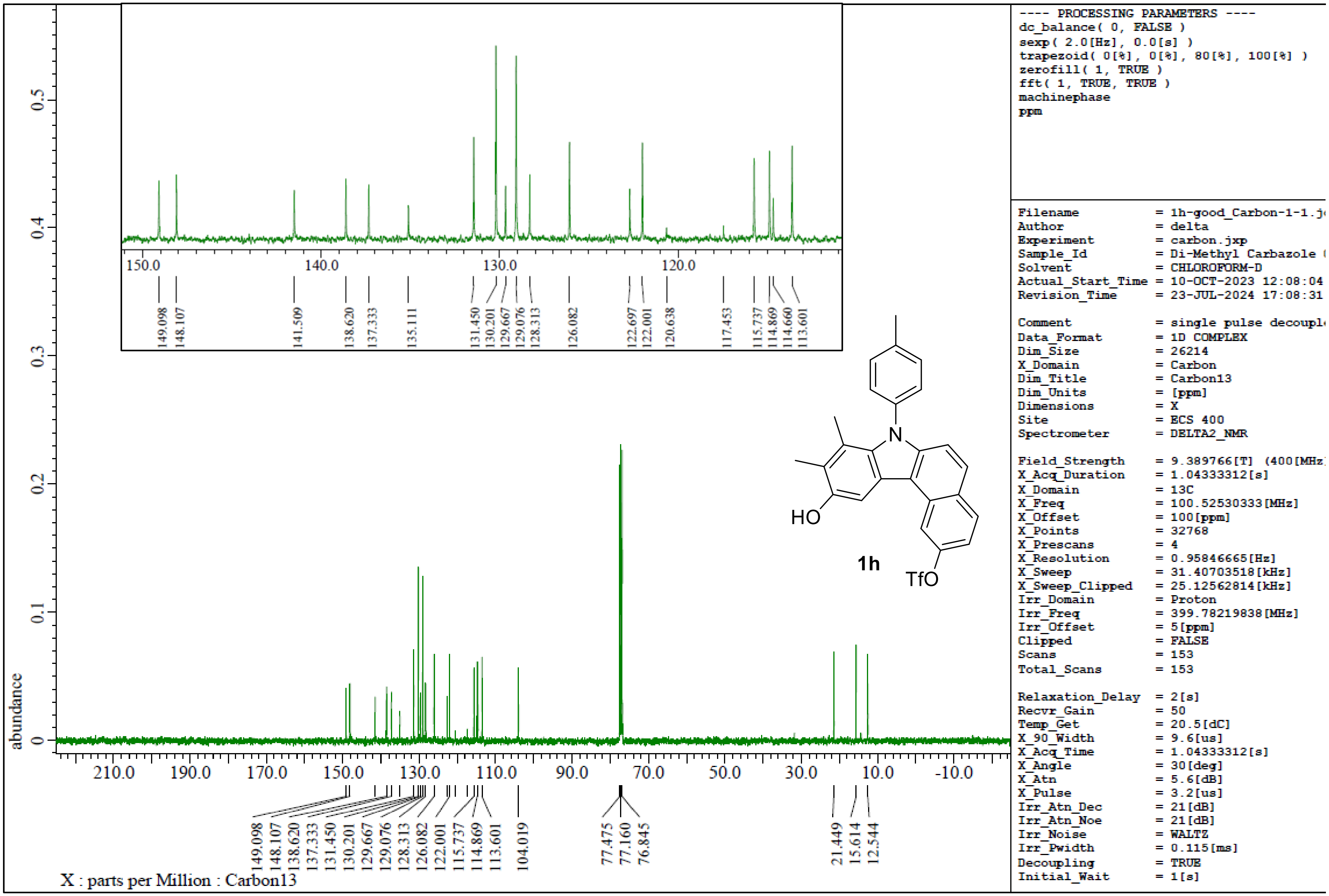

Compound **1h** (^{13}C NMR, 100 MHz, $CDCl_3$).

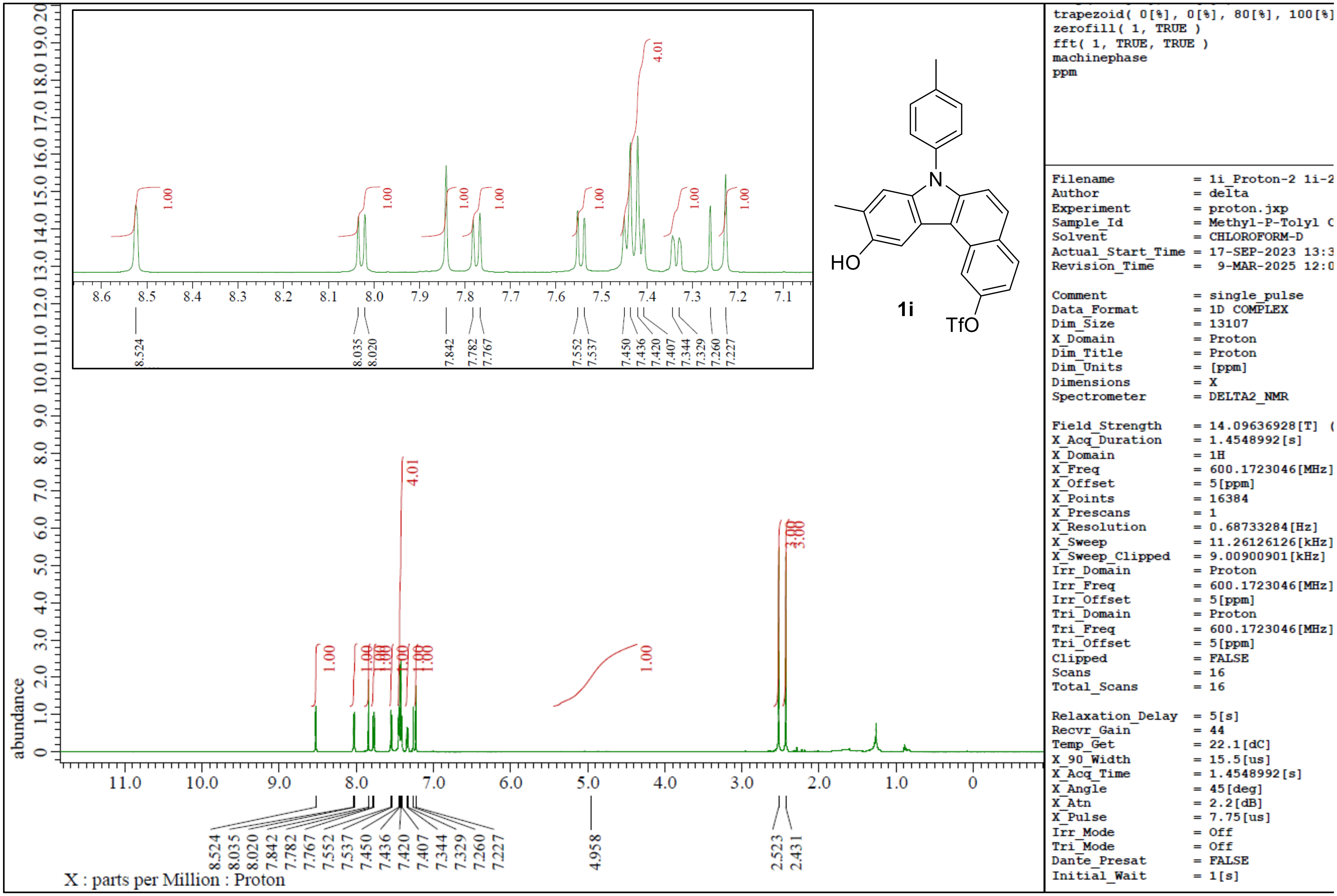

Compound **1i** (^{1}H NMR, 600 MHz, $CDCl_3$).

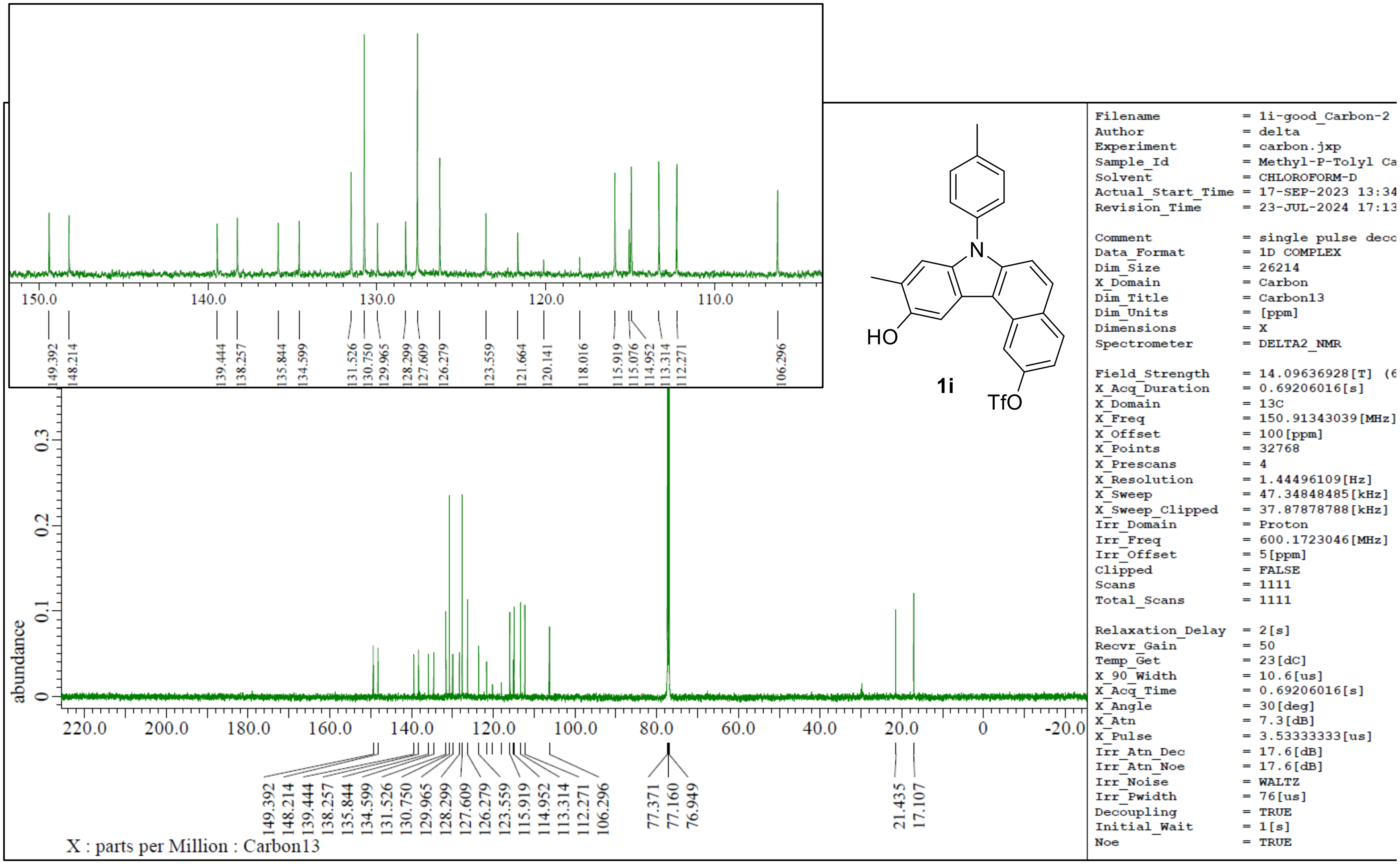

Compound **1i** (^{13}C NMR, 150 MHz, $CDCl_3$).

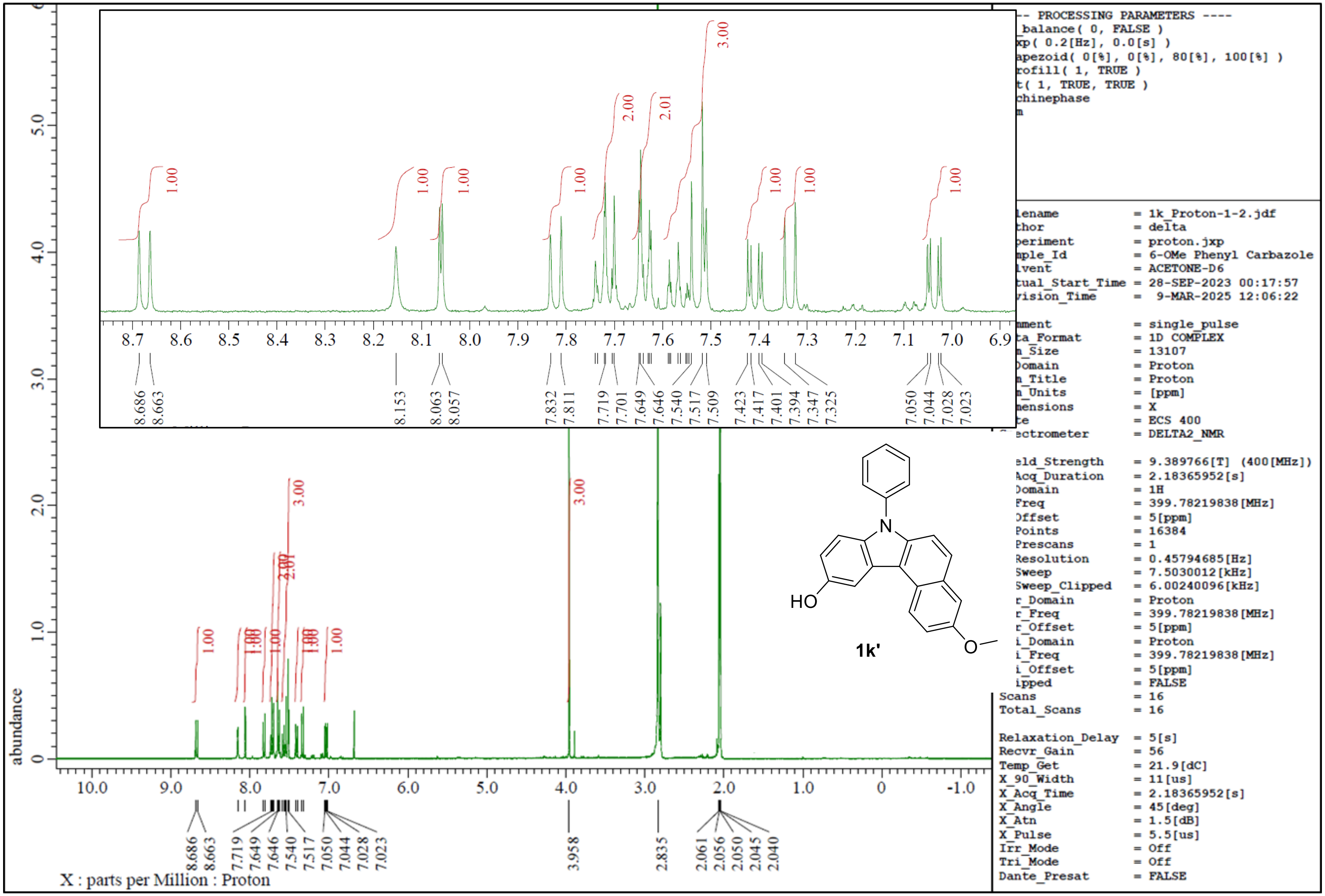

Compound **1k'** (^{1}H NMR, 400 MHz, $(CD_3)_2CO$).

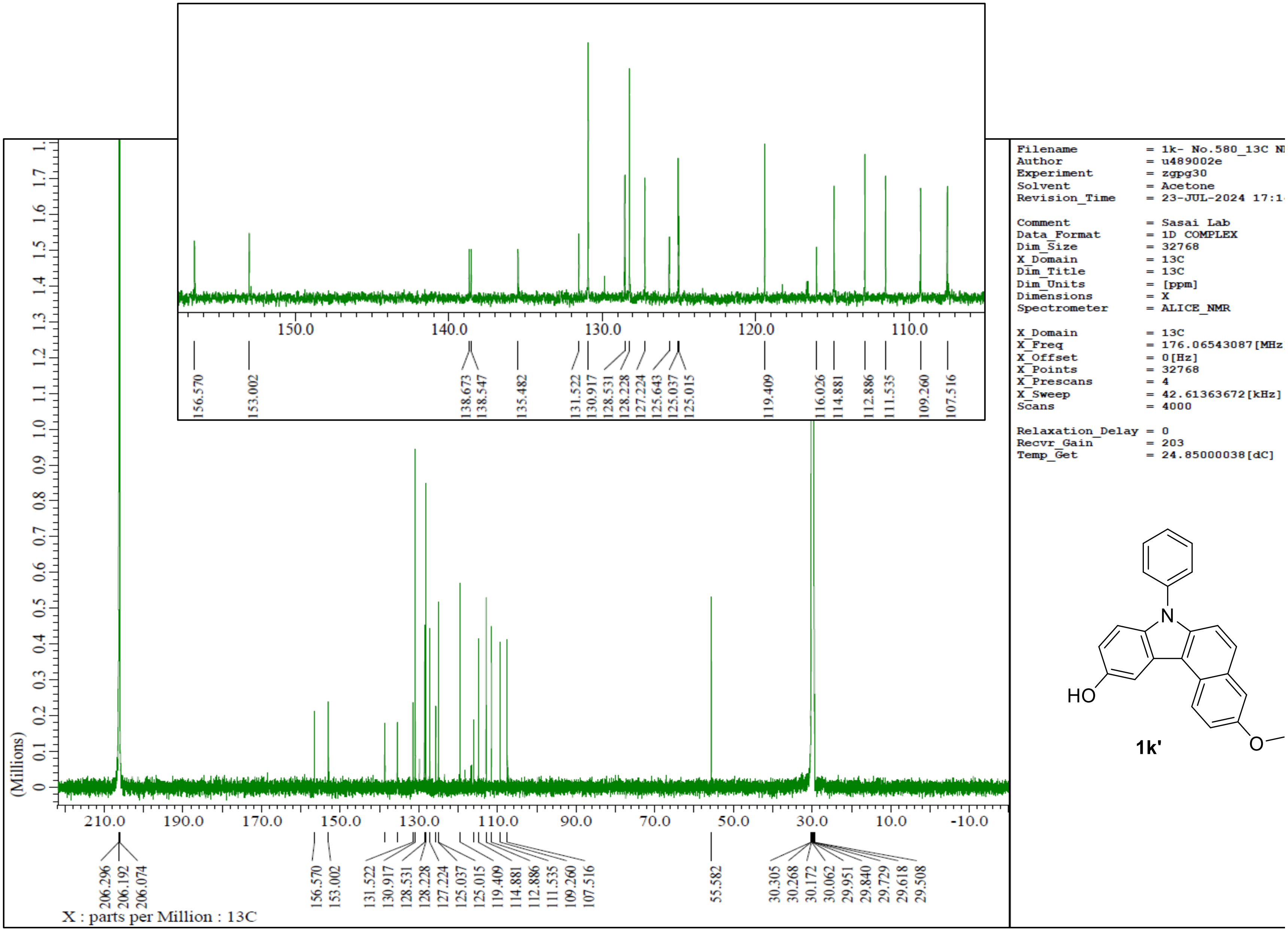

Compound **1k'** (^{13}C NMR, 175 MHz, $(CD_3)_2CO$).

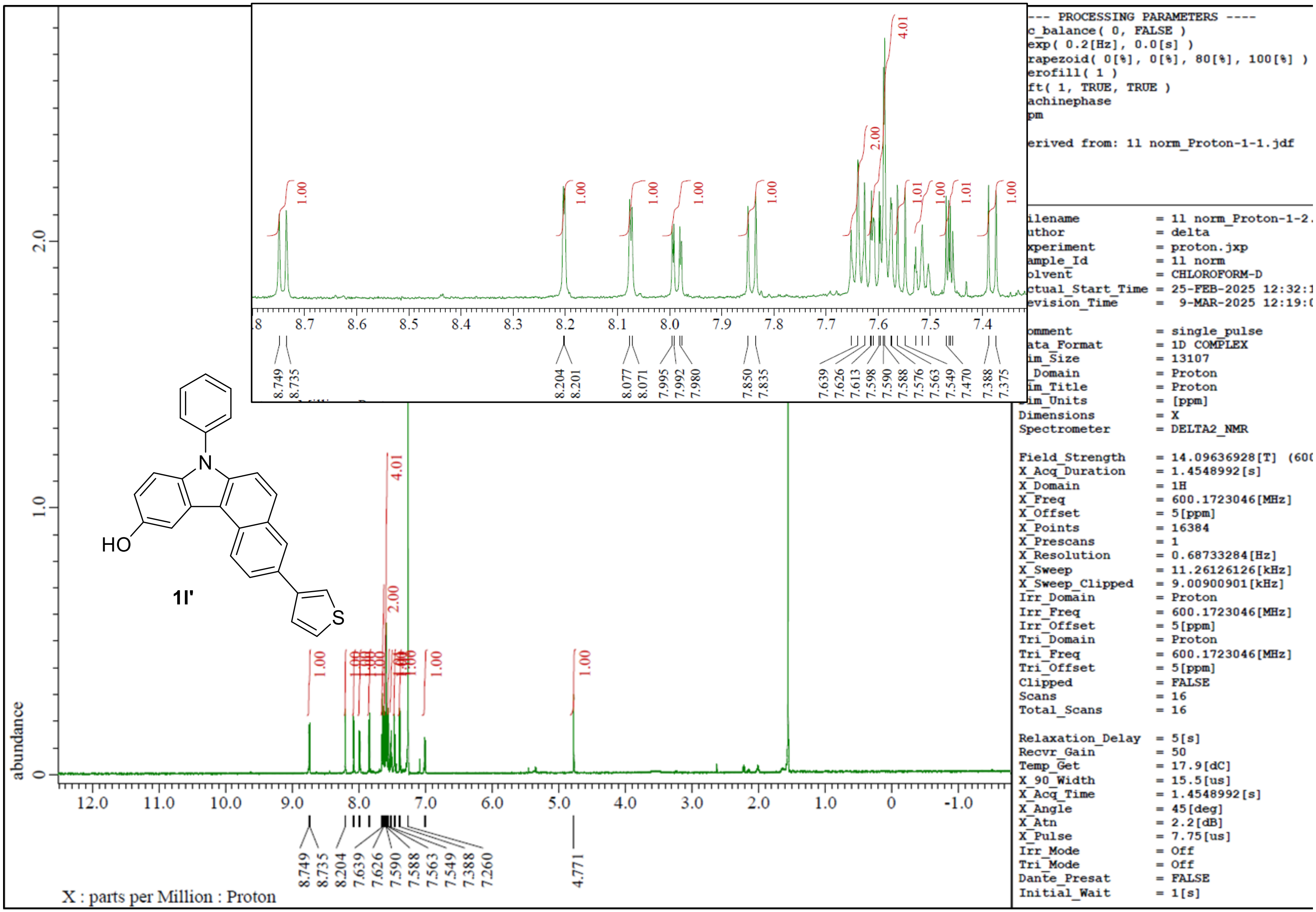
1l'
HO
N
S
abundance
X : parts per Million : Proton
8.749
8.735
8.204
8.201
8.077
8.071
7.995
7.992
7.980
7.850
7.835
7.639
7.626
7.613
7.598
7.590
7.588
7.576
7.563
7.549
7.470
7.388
7.375
7.260
4.771
1.00
2.00
4.01
1.01
---- PROCESSING PARAMETERS ----
c_balance(0, FALSE)
exp(0.2[Hz], 0.0[s])
rapezoid(0[%], 0[%], 80[%], 100[%])
erofill(1)
ft(1, TRUE, TRUE)
achinephase
pm
erived from: 11 norm_Proton-1-1.jdf
ilename = 11 norm_Proton-1-2.
uthor = delta
xperiment = proton.jxp
ample_Id = 11 norm
olvent = CHLOROFORM-D
ctual_Start_Time = 25-FEB-2025 12:32:1
evision_Time = 9-MAR-2025 12:19:(
omment = single_pulse
ata_Format = 1D COMPLEX
im_Size = 13107
Domain = Proton
im_Title = Proton
im_Units = [ppm]
Dimensions = X
Spectrometer = DELTA2_NMR
Field_Strength = 14.09636928[T] (60(
X_Acq_Duration = 1.4548992[s]
X_Domain = 1H
X_Freq = 600.1723046[MHz]
X_Offset = 5[ppm]
X_Points = 16384
X_Prescans = 1
X_Resolution = 0.68733284[Hz]
X_Sweep = 11.26126126[kHz]
X_Sweep_Clipped = 9.00900901[kHz]
Irr_Domain = Proton
Irr_Freq = 600.1723046[MHz]
Irr_Offset = 5[ppm]
Tri_Domain = Proton
Tri_Freq = 600.1723046[MHz]
Tri_Offset = 5[ppm]
Clipped = FALSE
Scans = 16
Total_Scans = 16
Relaxation_Delay = 5[s]
Recvr_Gain = 50
Temp_Get = 17.9[dC]
X_90_Width = 15.5[us]
X_Acq_Time = 1.4548992[s]
X_Angle = 45[deg]
X_Atn = 2.2[dB]
X_Pulse = 7.75[us]
Irr_Mode = Off
Tri_Mode = Off
Dante_Presat = FALSE
Initial_Wait = 1[s]

Compound **1l'** (^{1}H NMR, 600 MHz, $CDCl_3$).

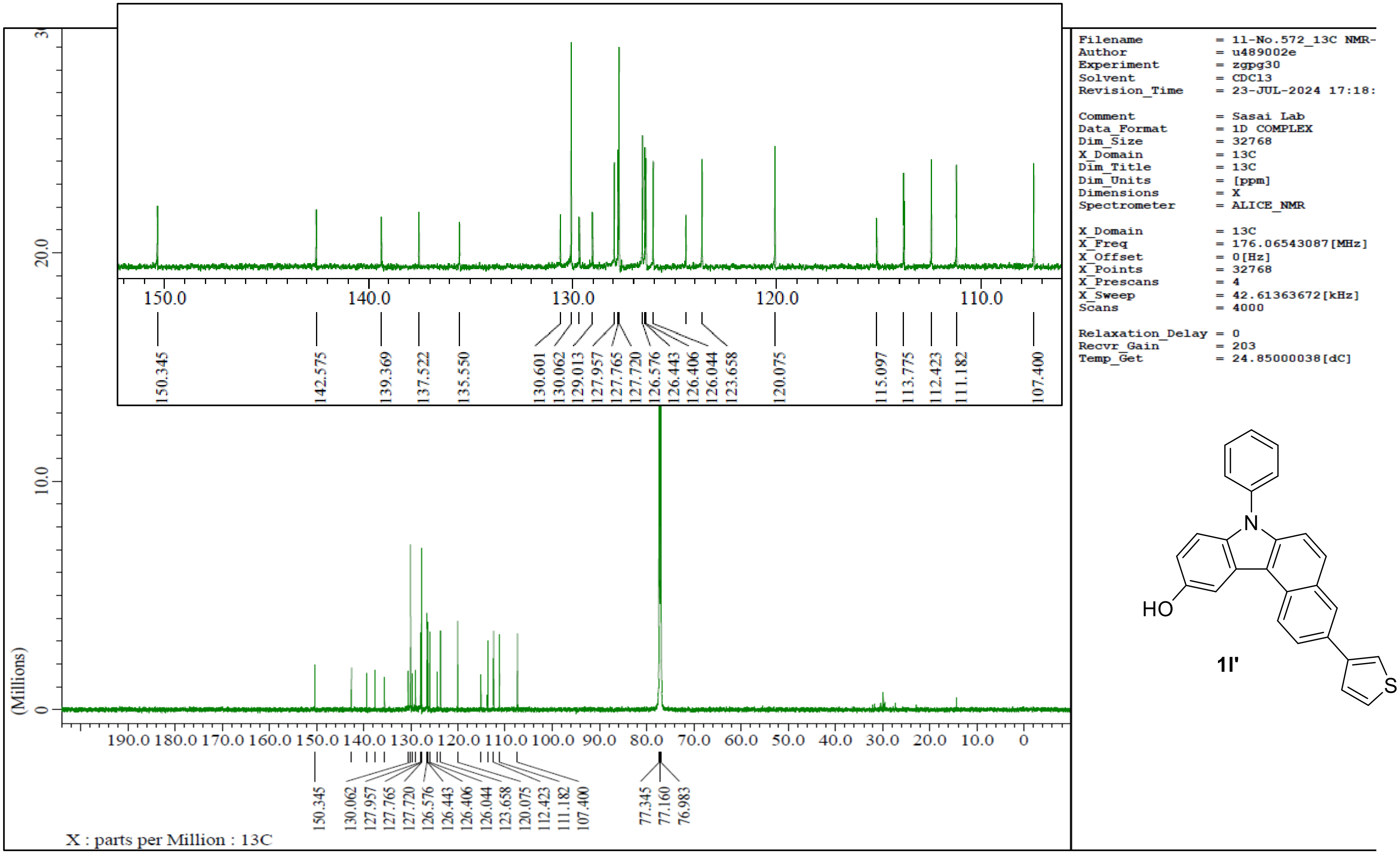

Compound **1l'** (^{13}C NMR, 175 MHz, $CDCl_3$).

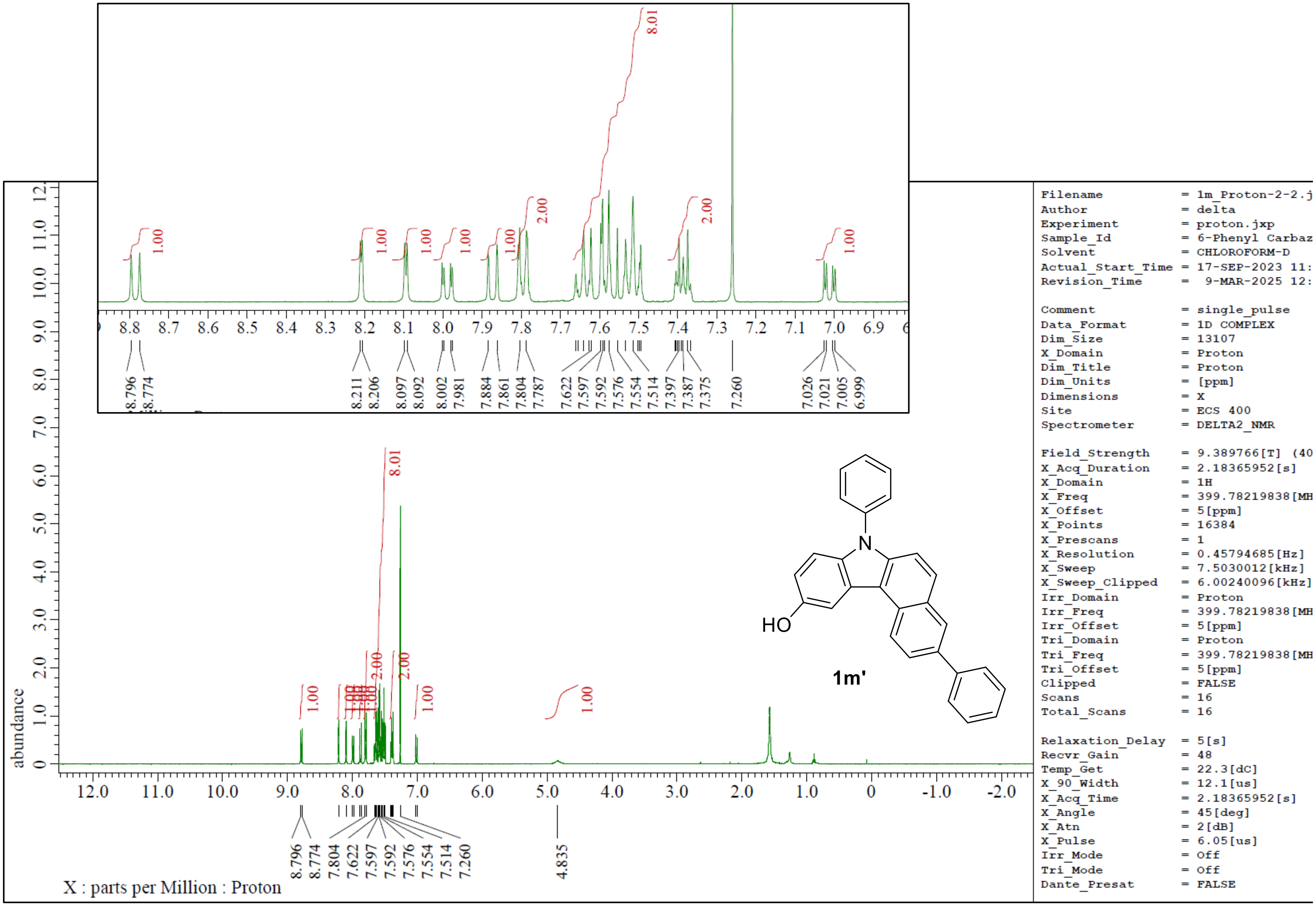

Compound **1m'** (^{1}H NMR, 400 MHz, $CDCl_3$).

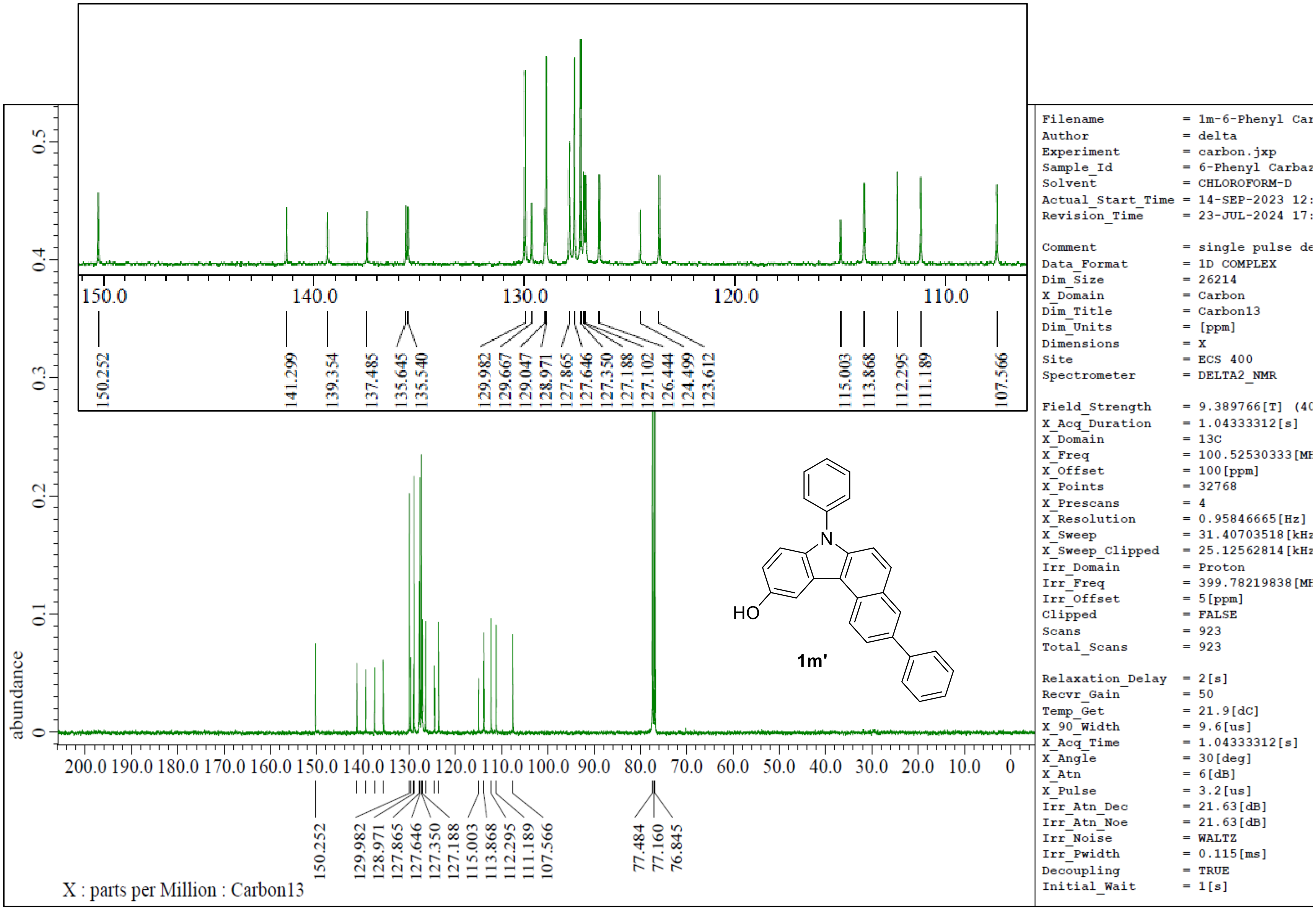

Compound **1m'** (^{13}C NMR, 100 MHz, $CDCl_3$).

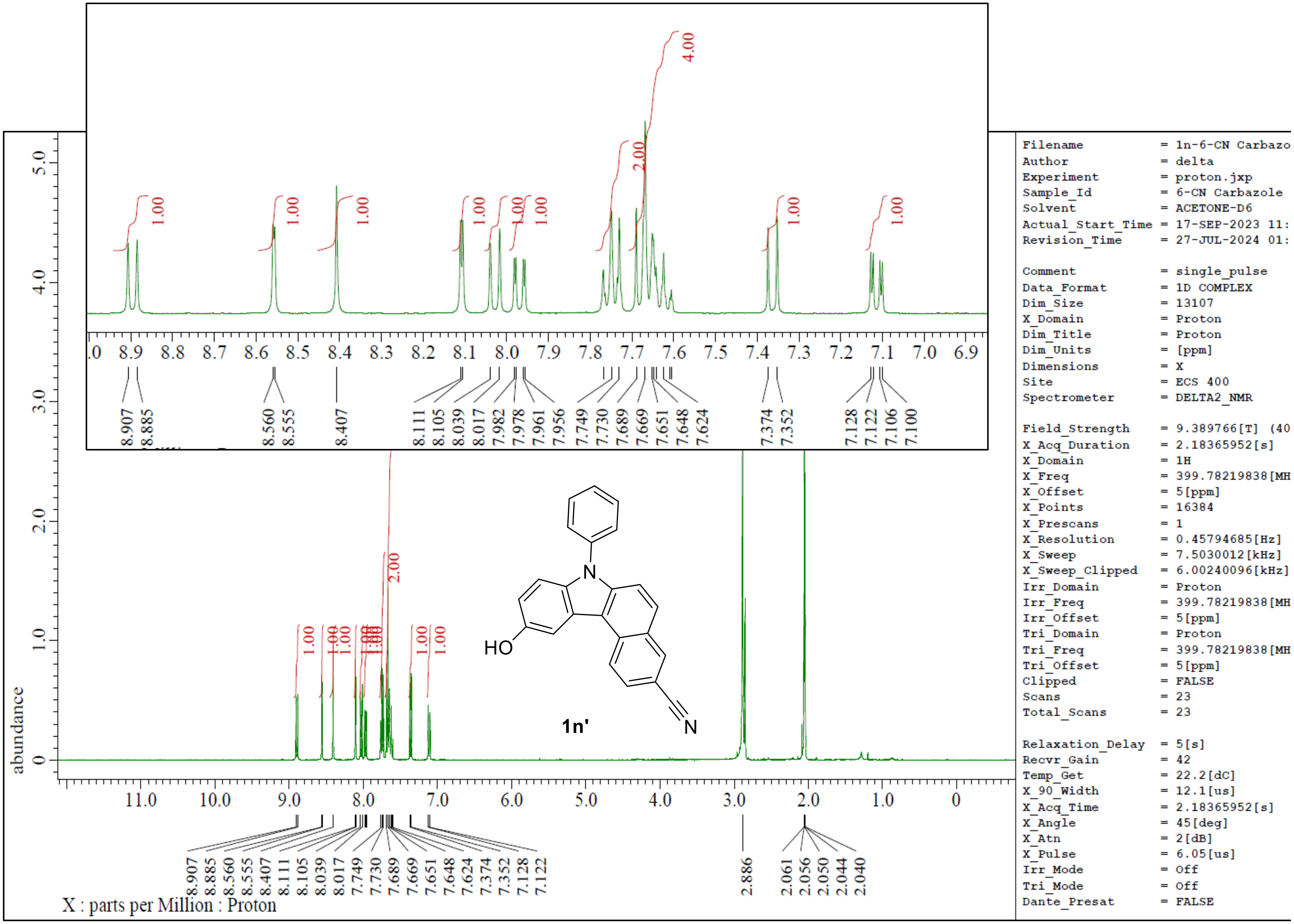

Compound **1n'** (^{1}H NMR, 400 MHz, $(CD_3)_2CO$).

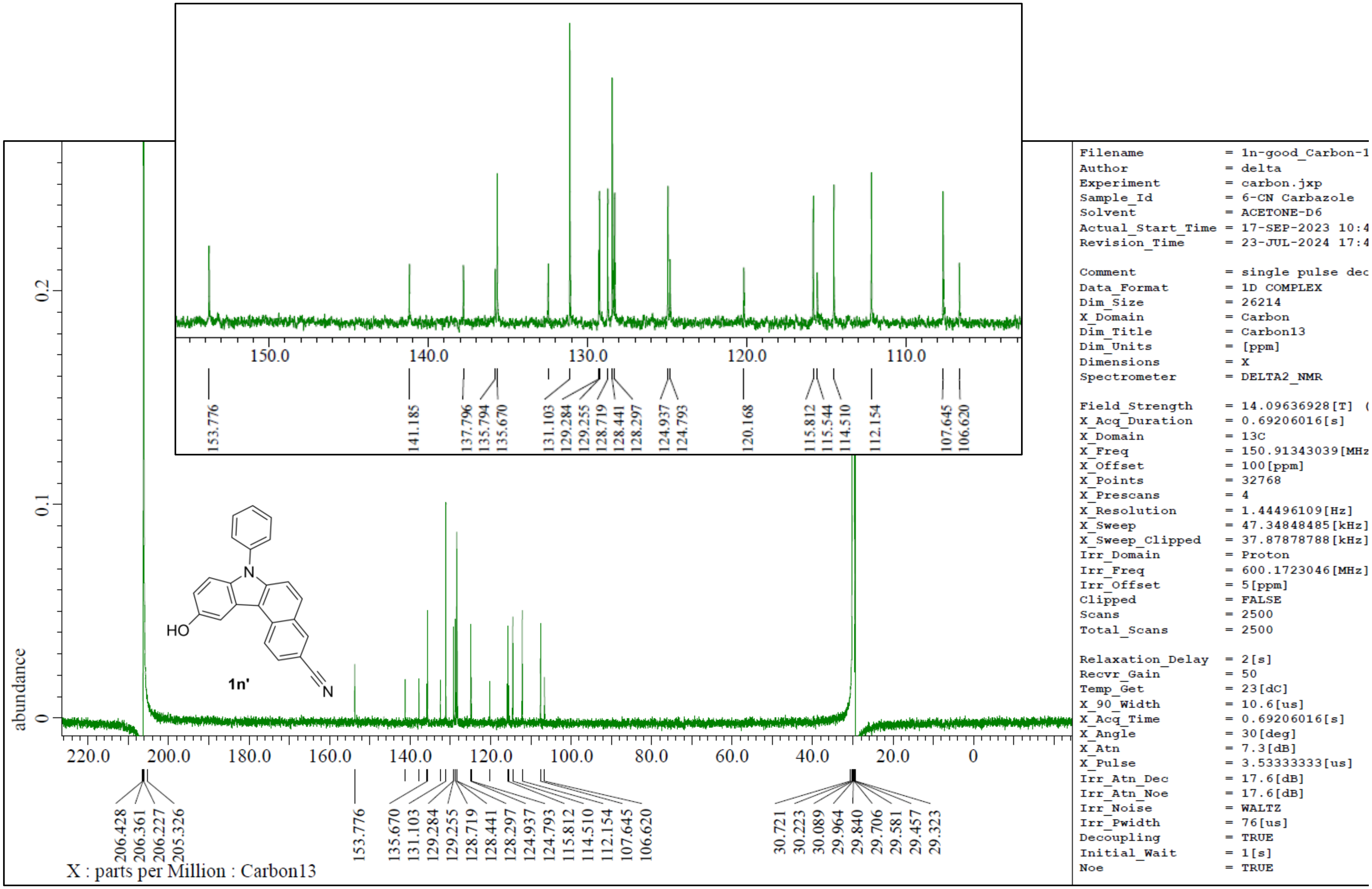

Compound **1n'** (^{13}C NMR, 150 MHz, $(CD_3)_2CO$).

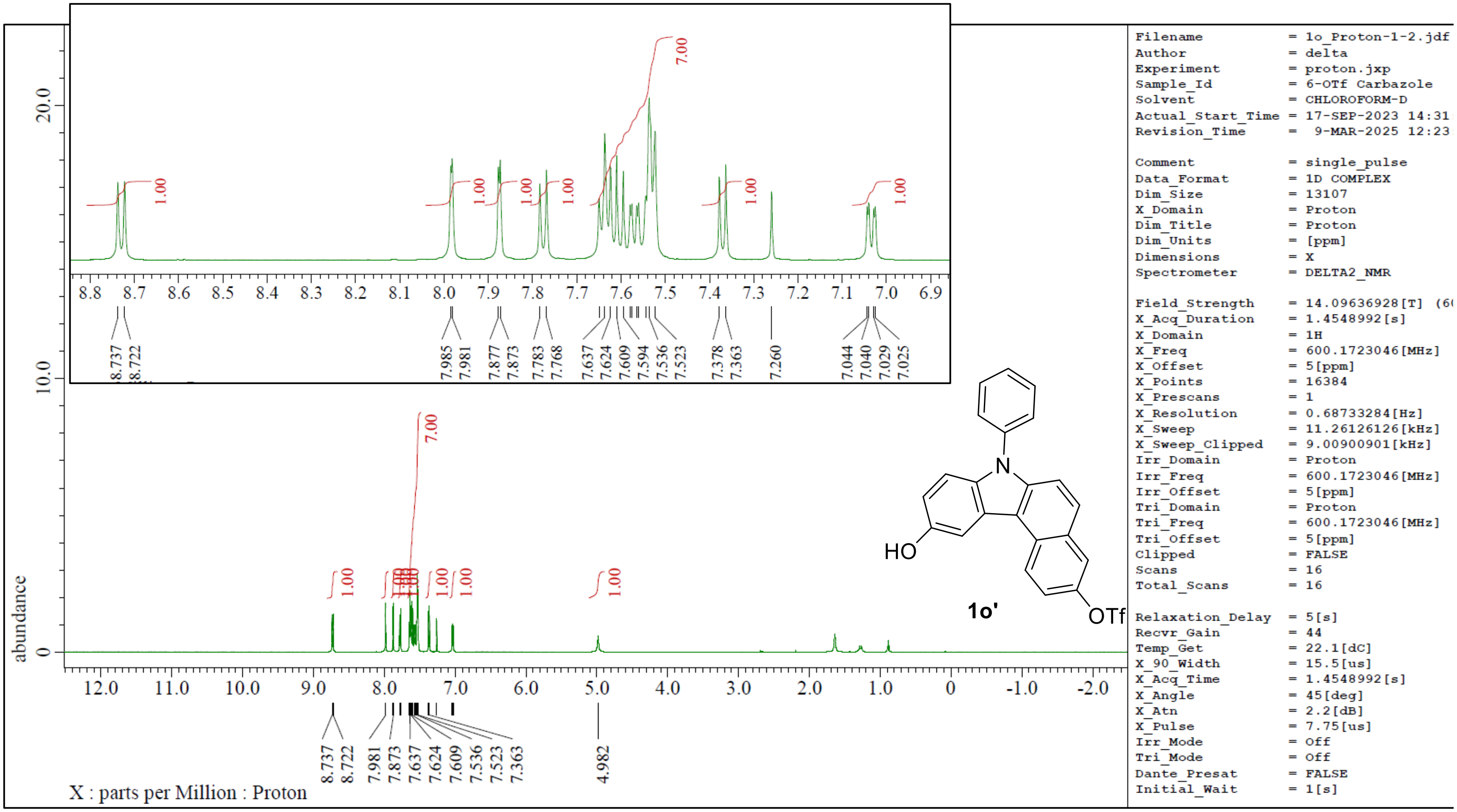

Compound **1o'** (^{1}H NMR, 600 MHz, $CDCl_3$).

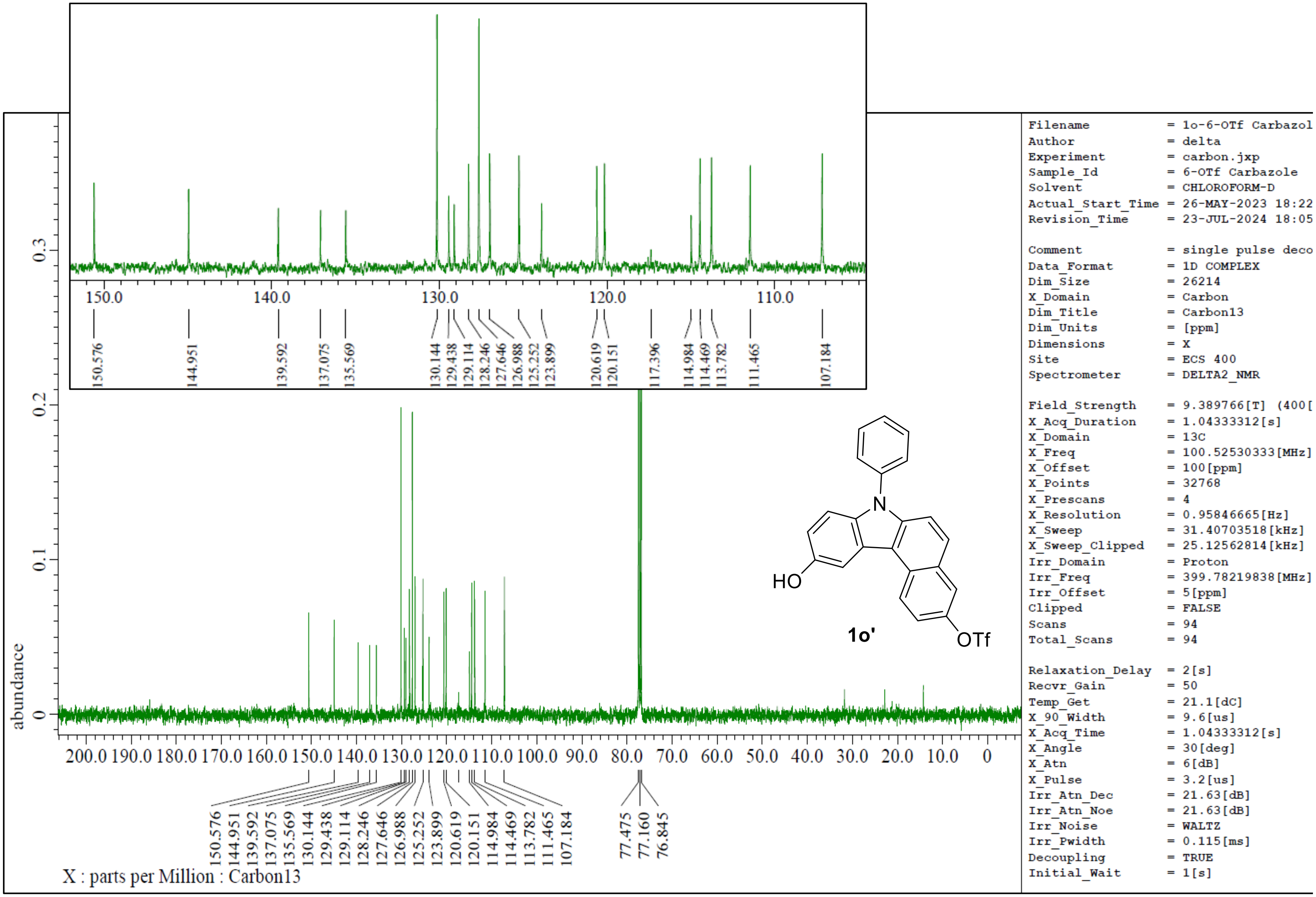

Compound **1o'** (^{13}C NMR, 100 MHz, $CDCl_3$).

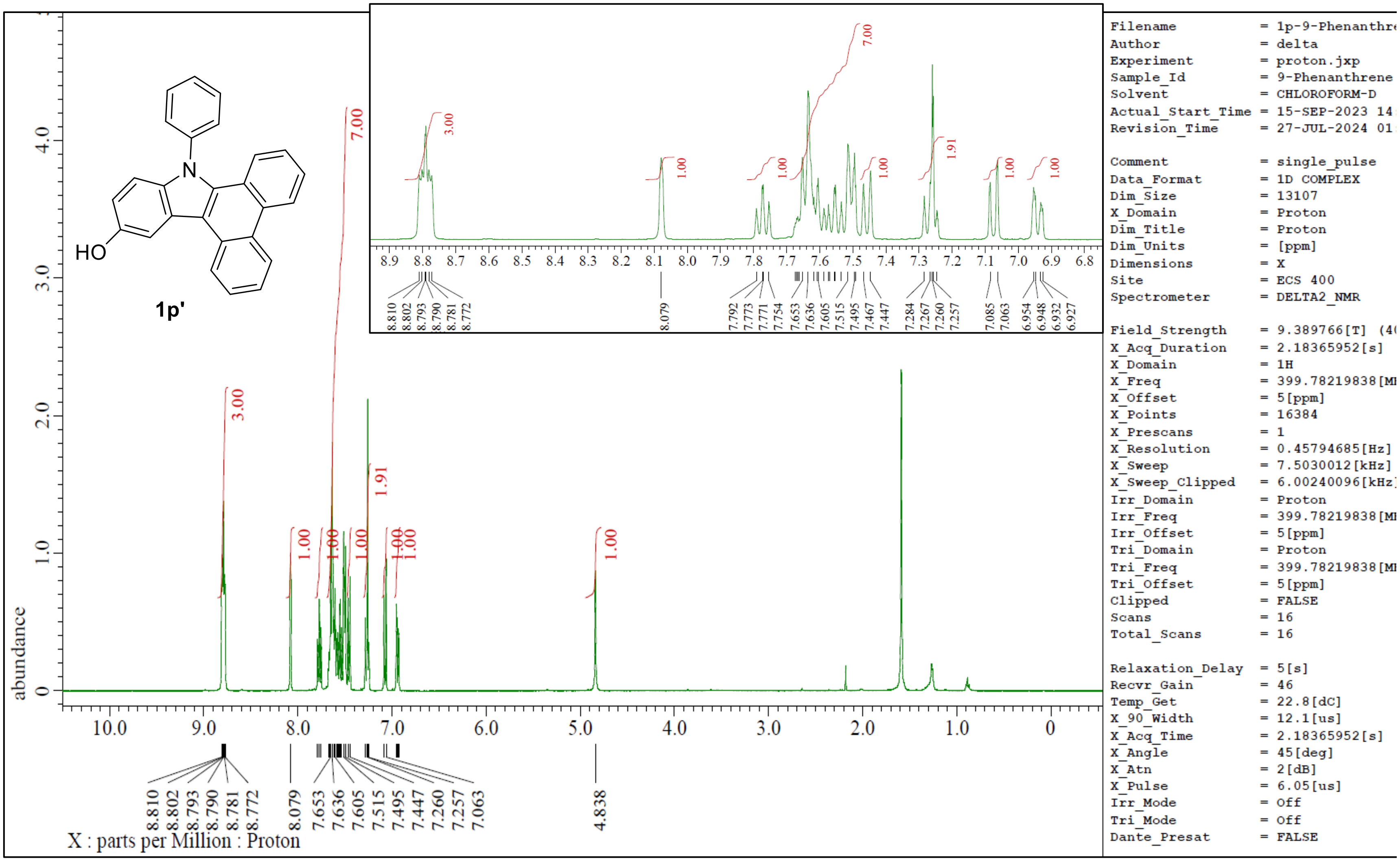

Compound **1p'** (^{1}H NMR, 400 MHz, $CDCl_3$).

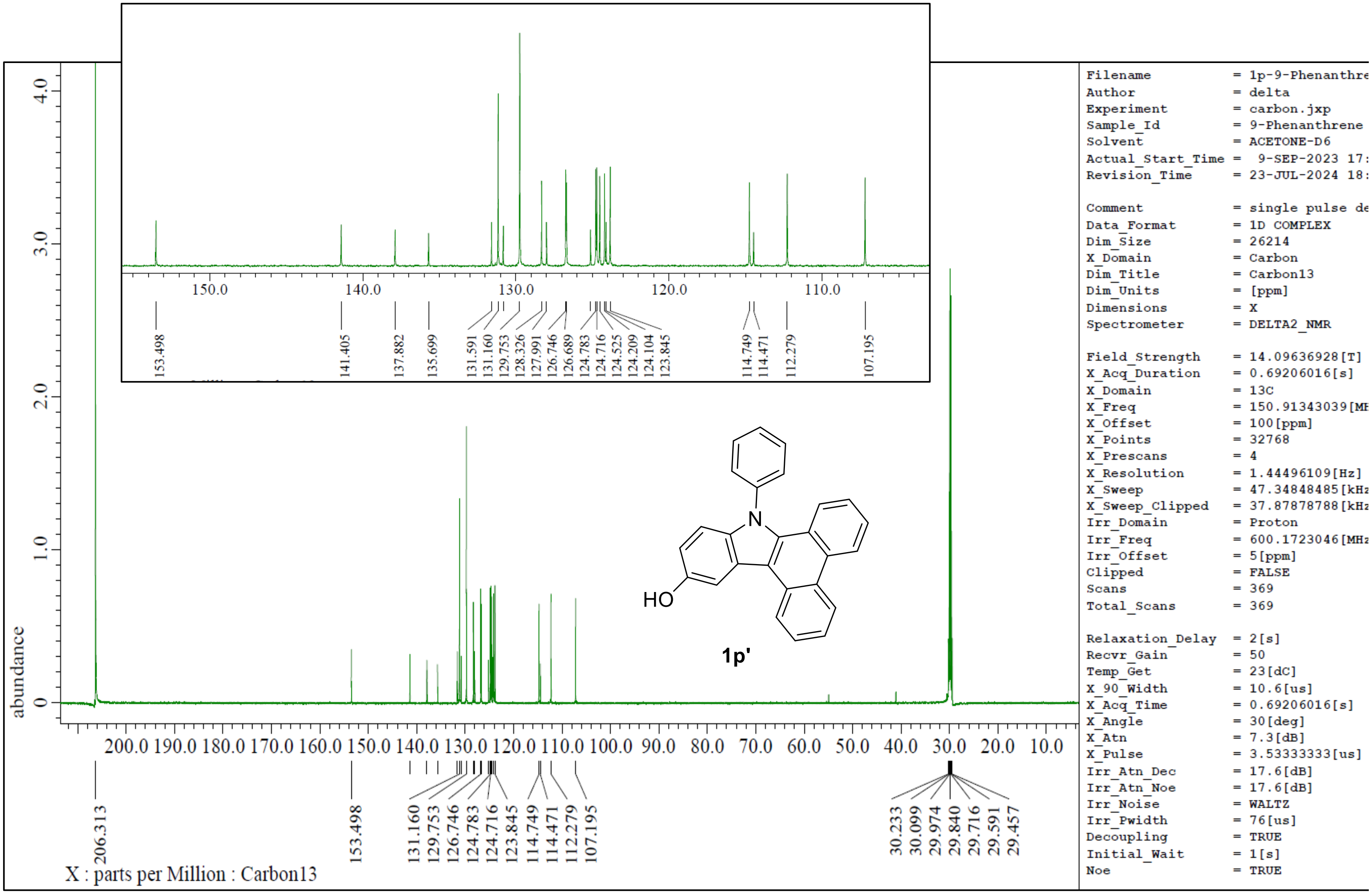

Compound **1p'** (^{13}C NMR, 150 MHz, $(CD_3)_2CO$).

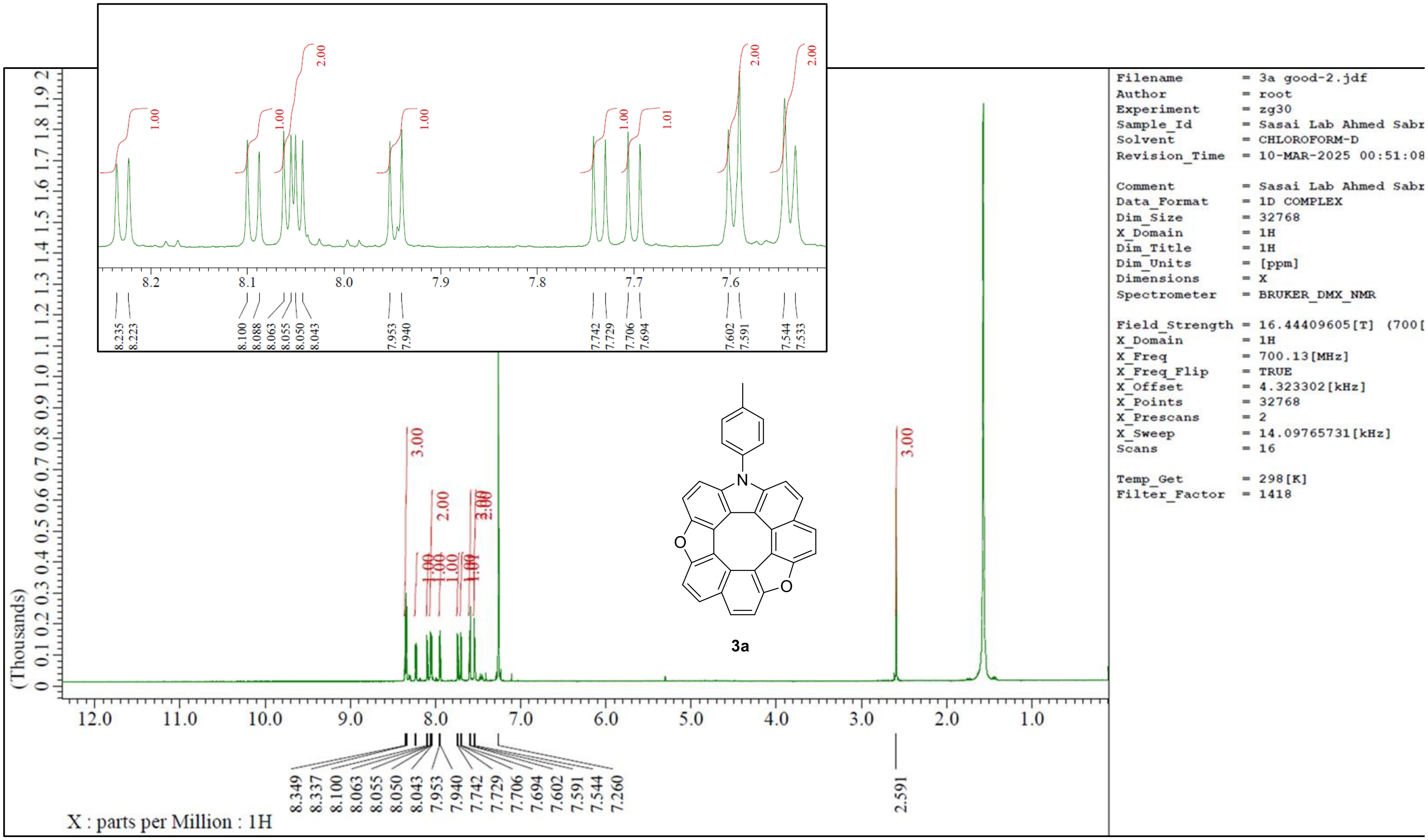

Compound **3a** (^{1}H NMR, 700 MHz, $CDCl_3$).

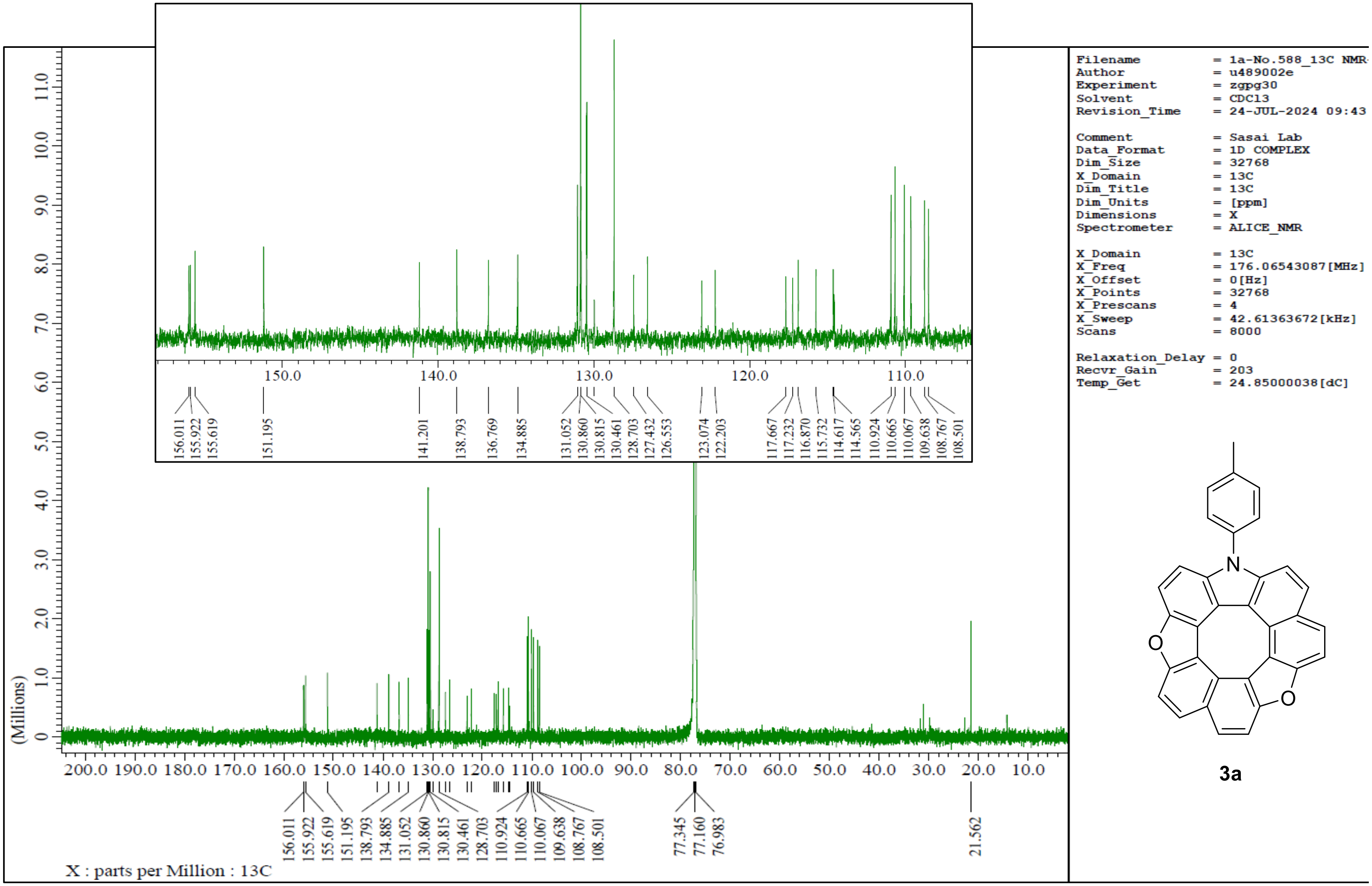

Compound **3a** (^{13}C NMR, 175 MHz, $CDCl_3$).

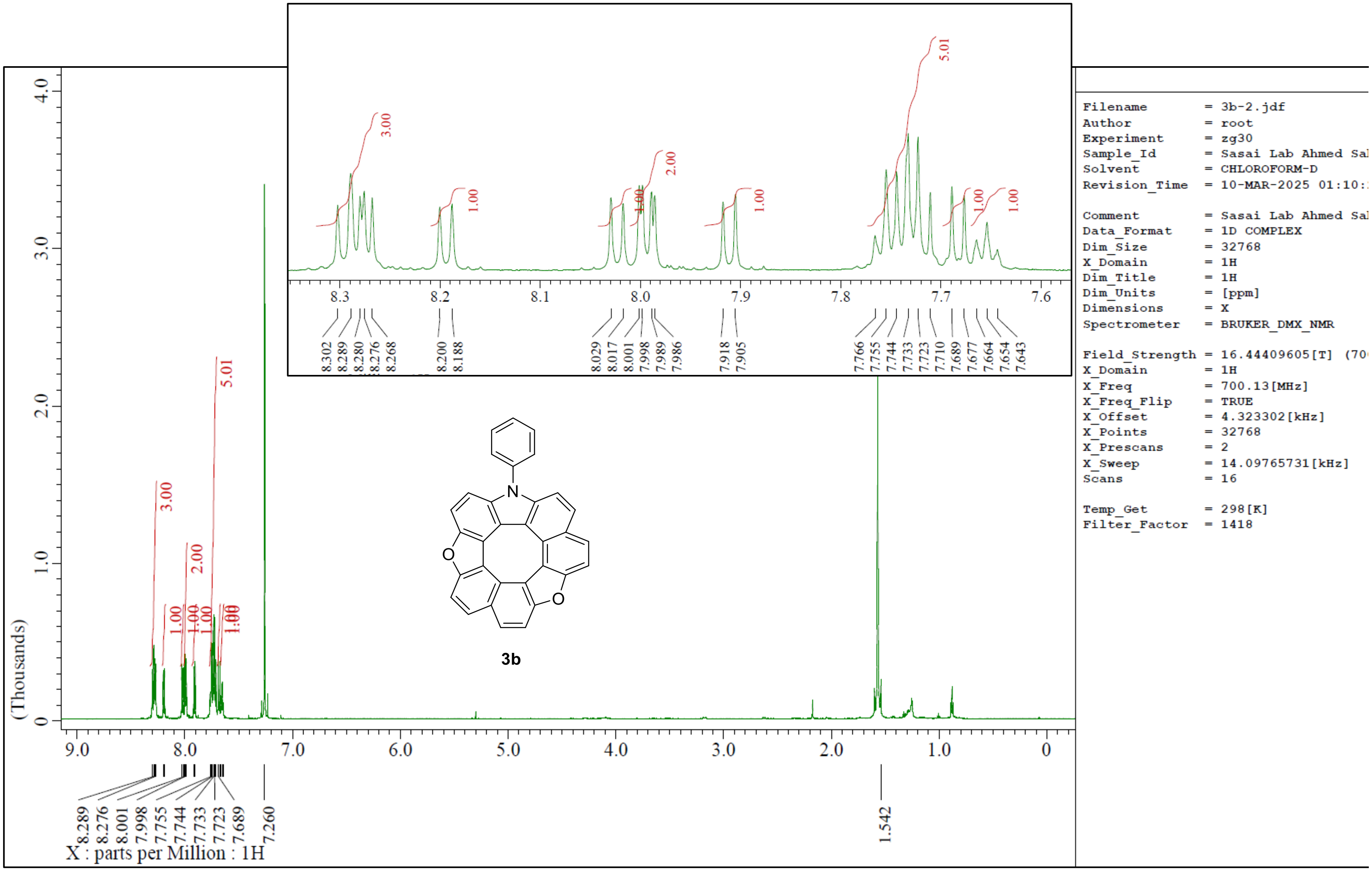

Compound **3b** (^{1}H NMR, 700 MHz, $CDCl_3$).

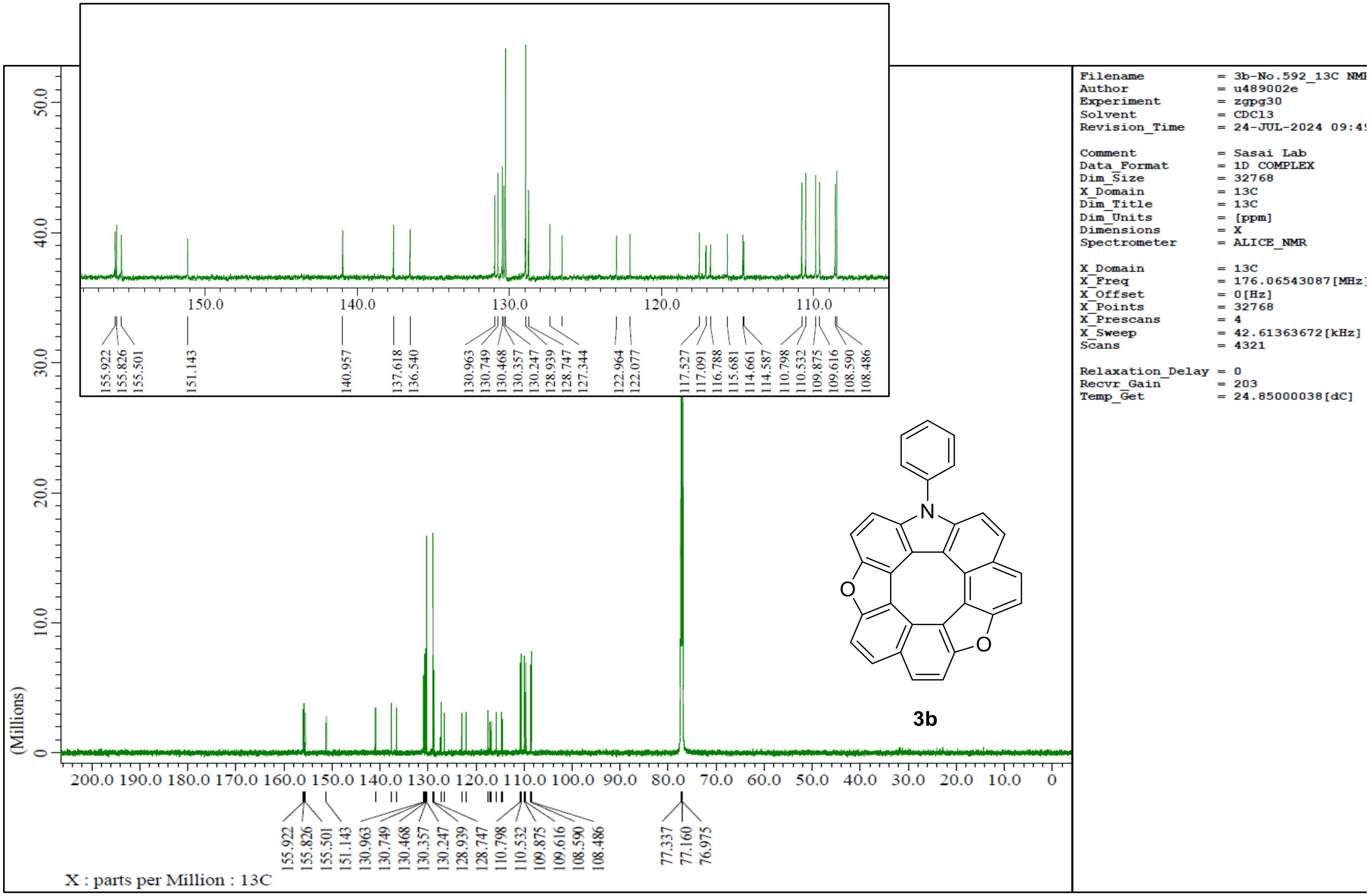

Compound **3b** (^{13}C NMR, 175 MHz, $CDCl_3$).

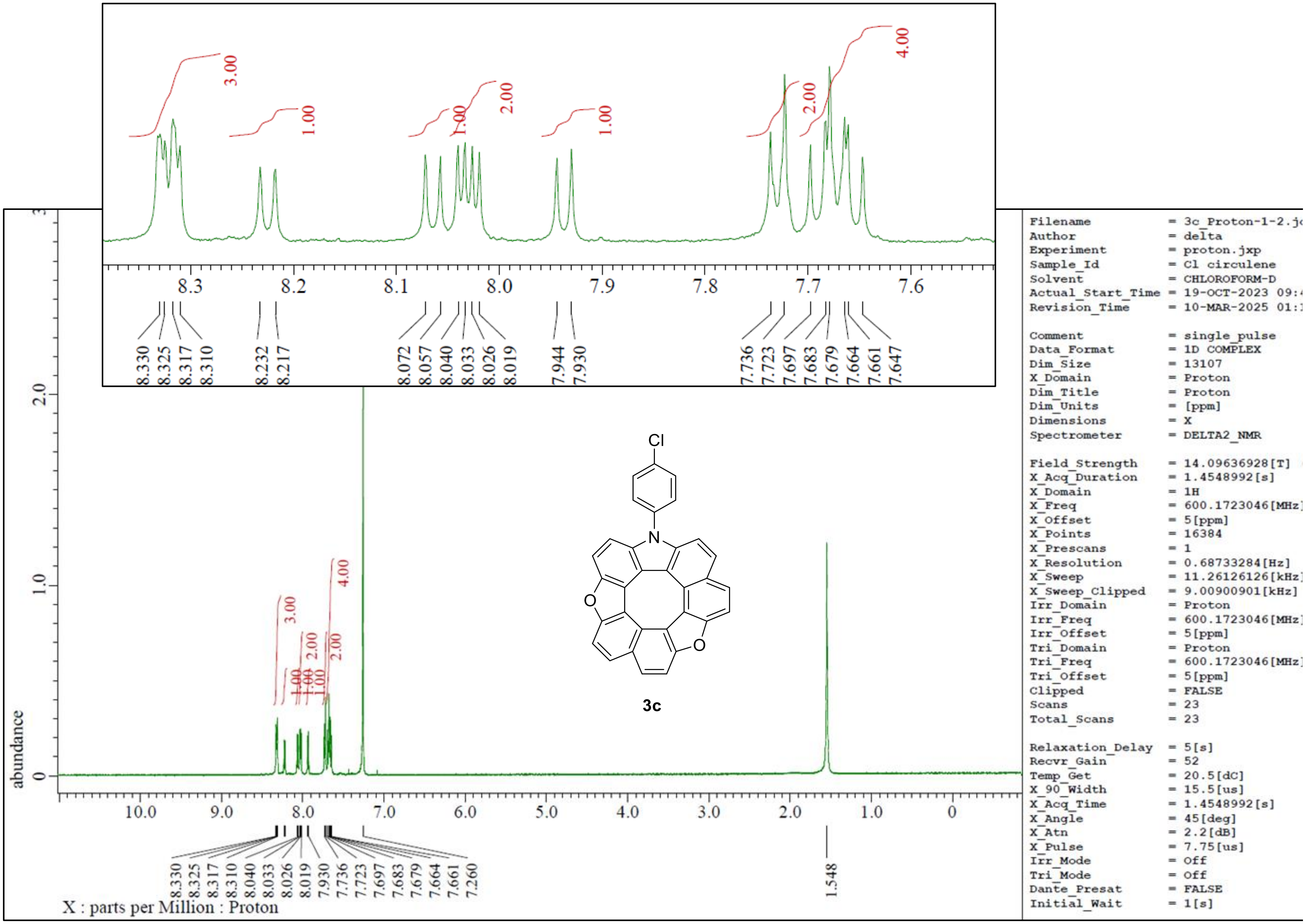

Compound **3c** (^{1}H NMR, 600 MHz, $CDCl_3$).

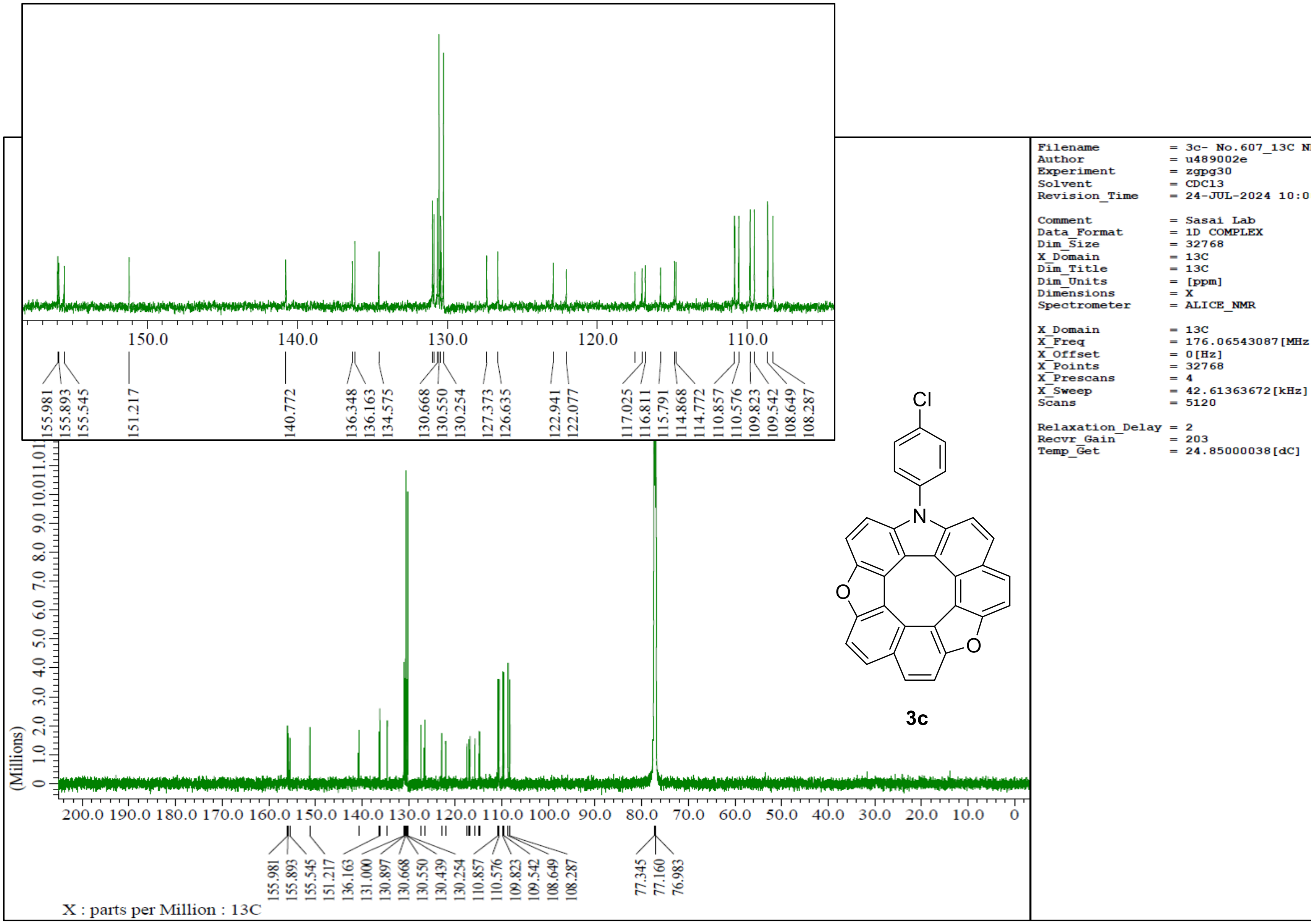

Compound **3c** (^{13}C NMR, 175 MHz, $CDCl_3$).

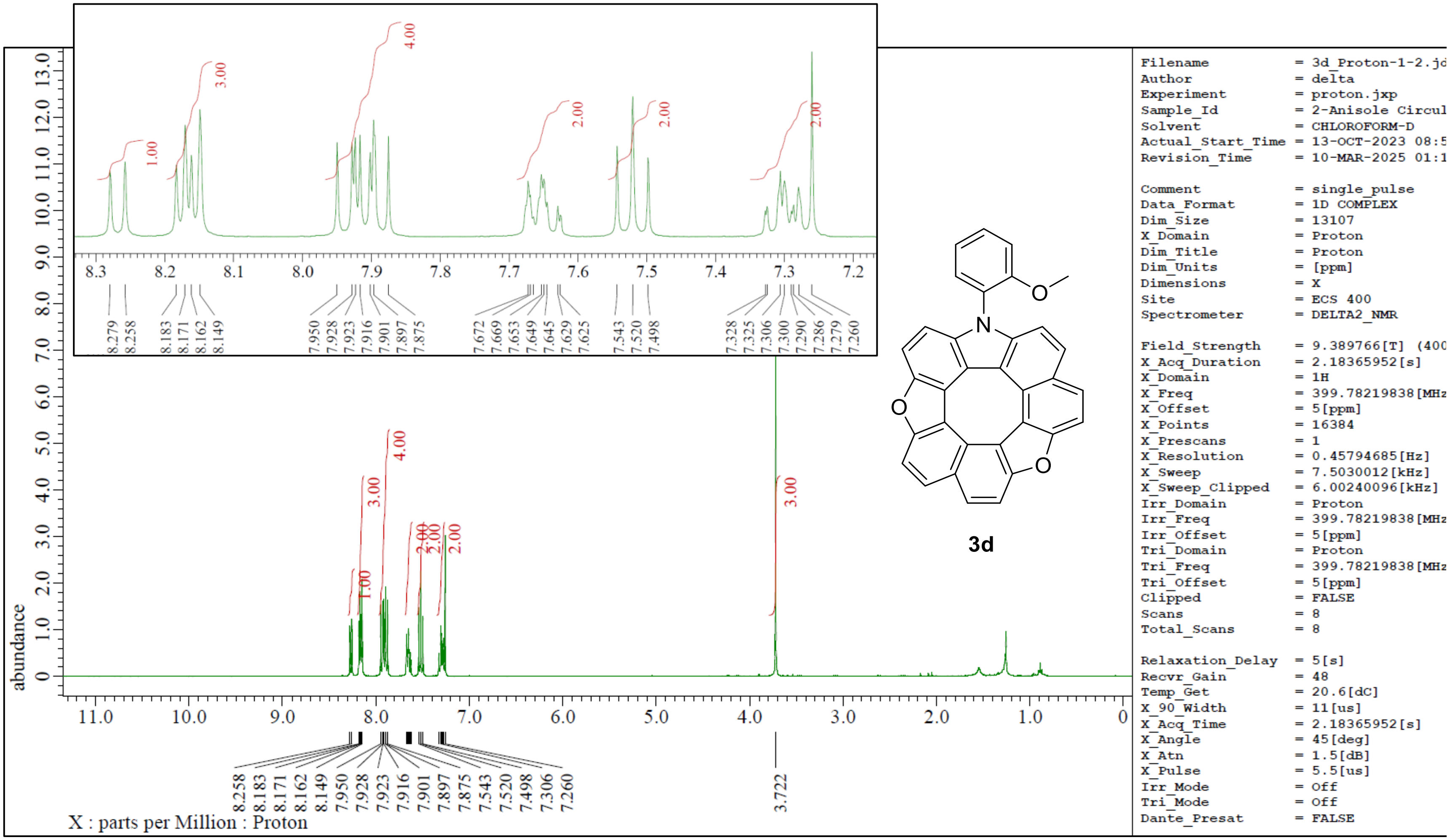

Compound **3d** (^{1}H NMR, 400 MHz, $CDCl_3$).

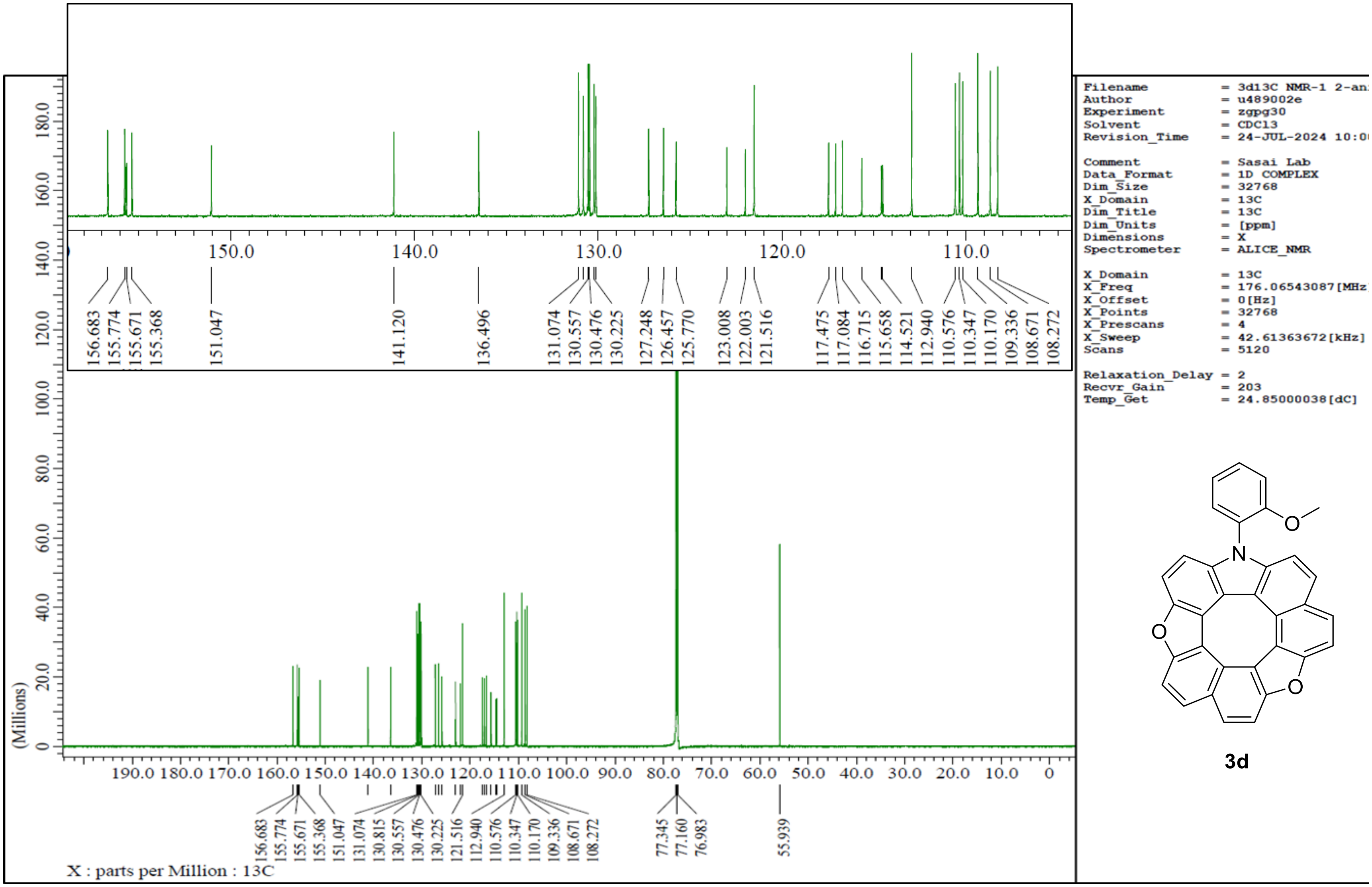

Compound **3d** (^{13}C NMR, 175 MHz, $CDCl_3$).

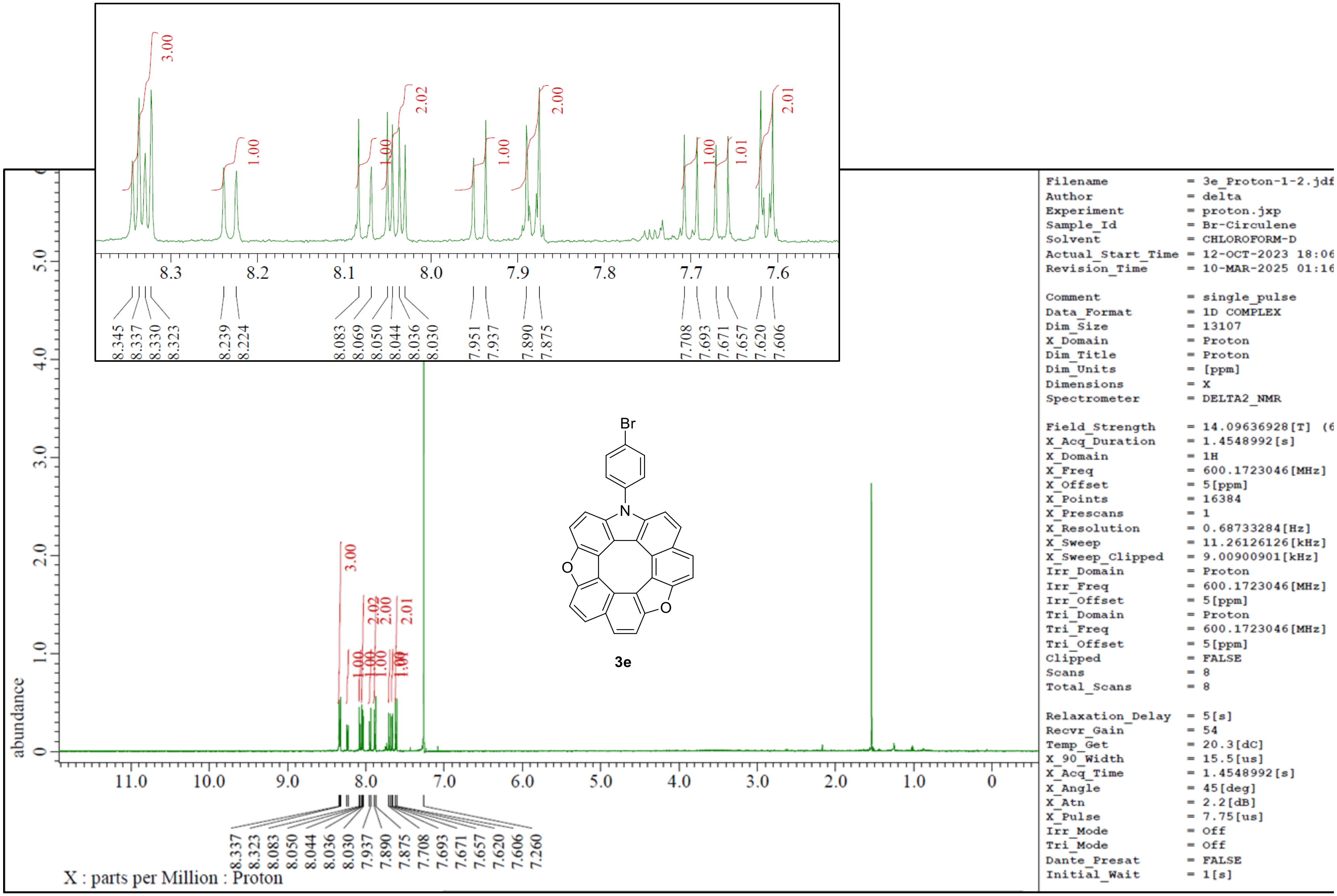

Compound **3e** (^{1}H NMR, 600 MHz, $CDCl_3$).

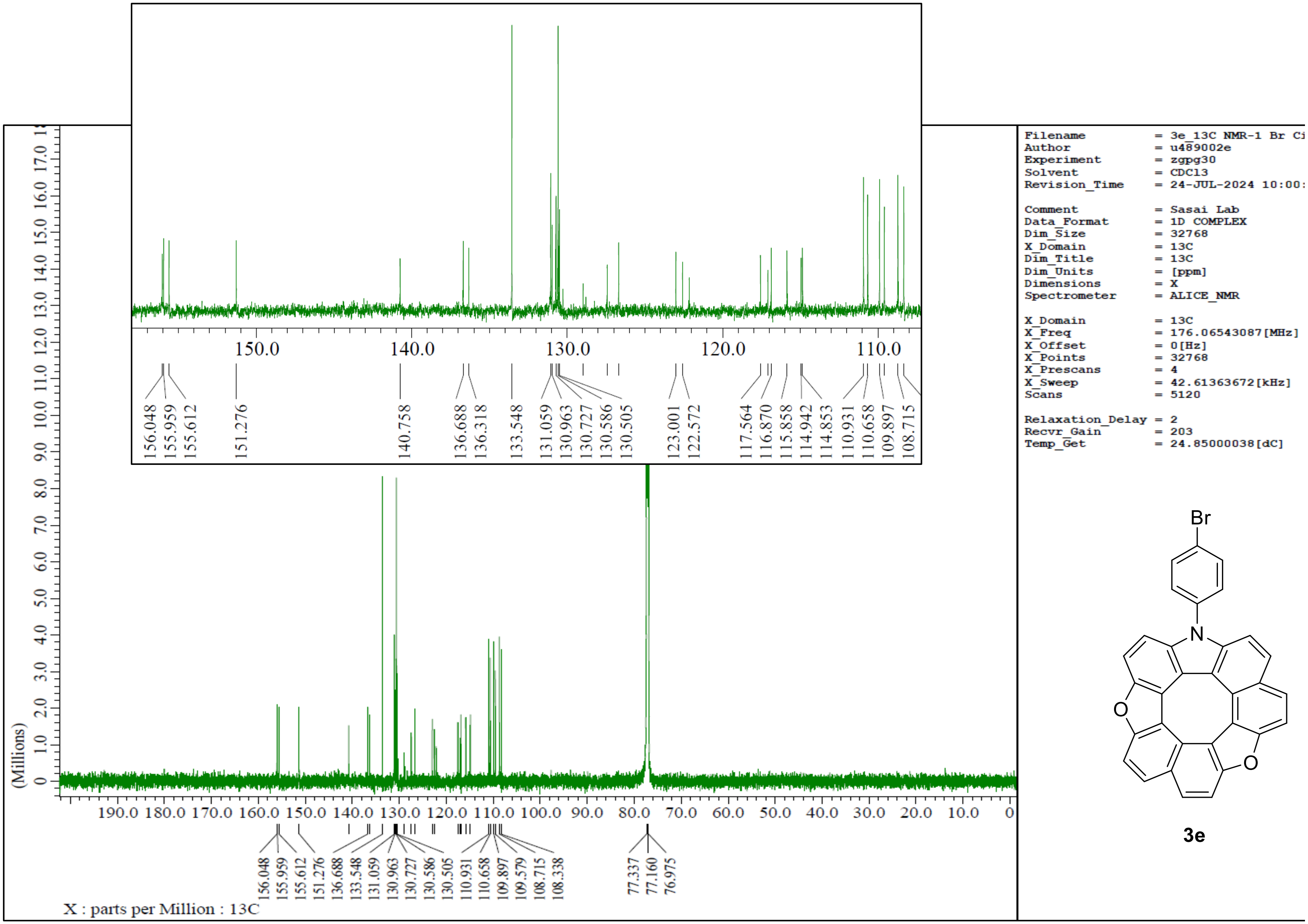

3e

Compound **3e** (^{13}C NMR, 175 MHz, $CDCl_3$).

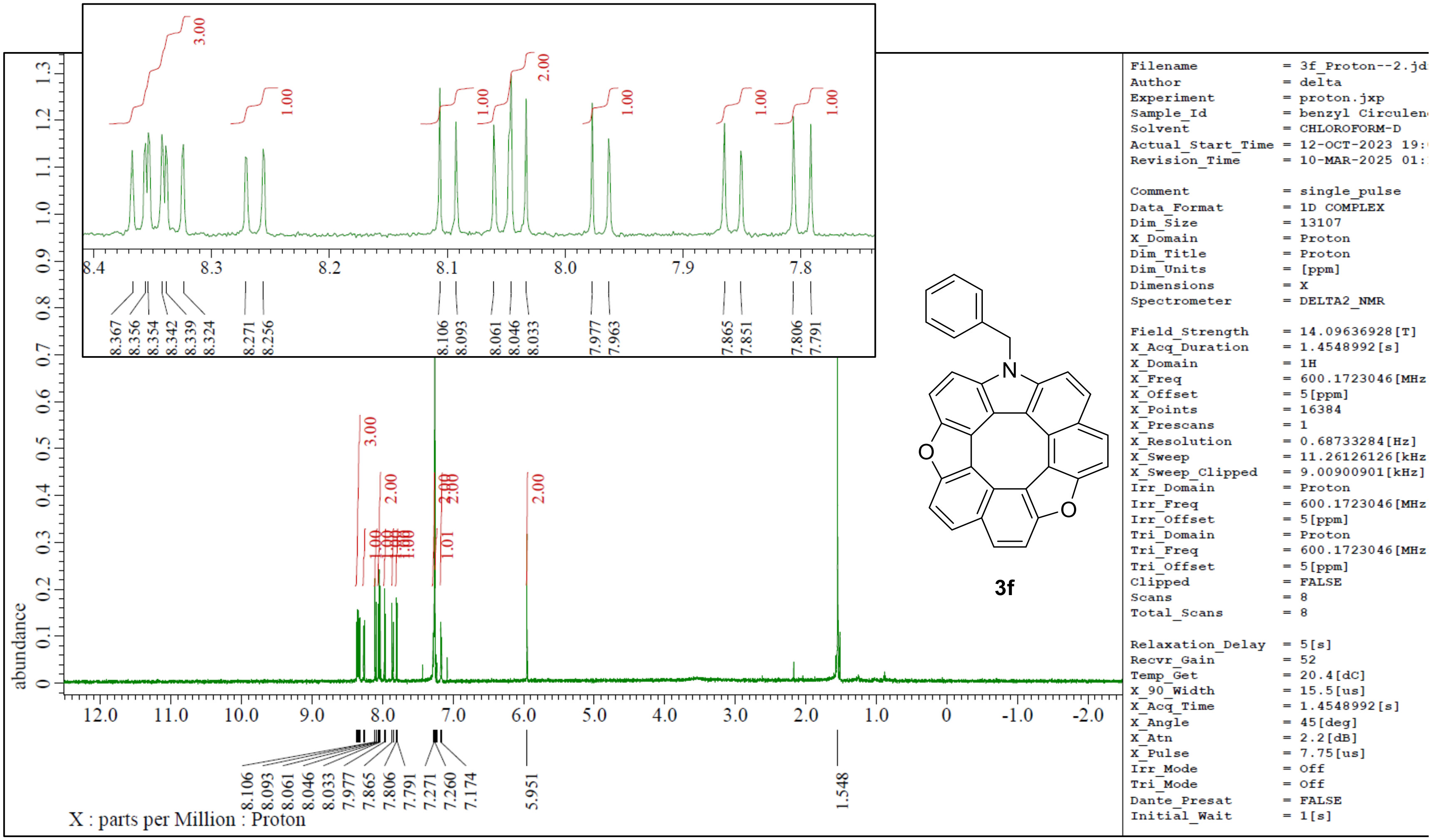

Compound **3f** (^{1}H NMR, 600 MHz, $CDCl_3$).

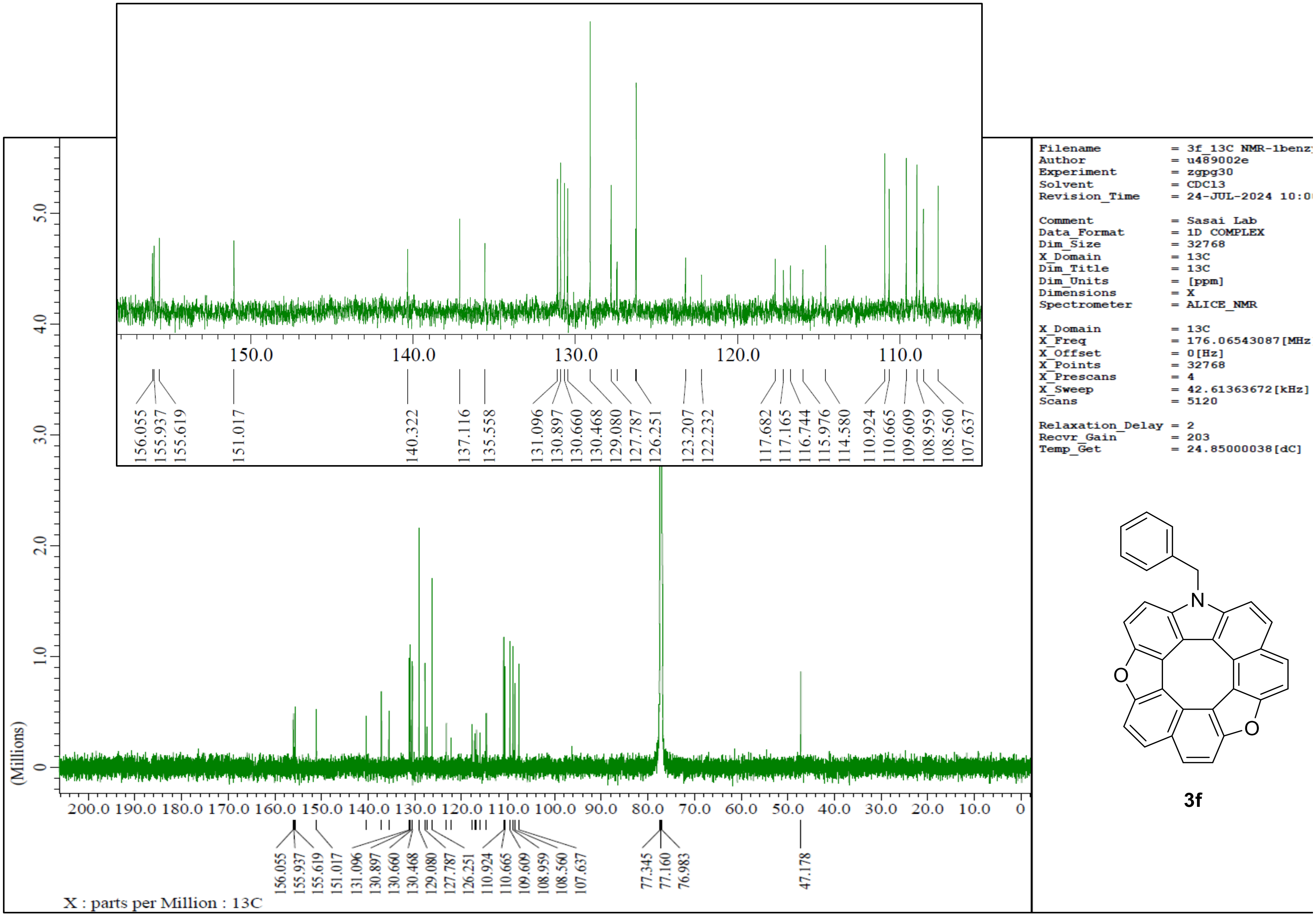

Compound **3f** (^{13}C NMR, 175 MHz, $CDCl_3$).

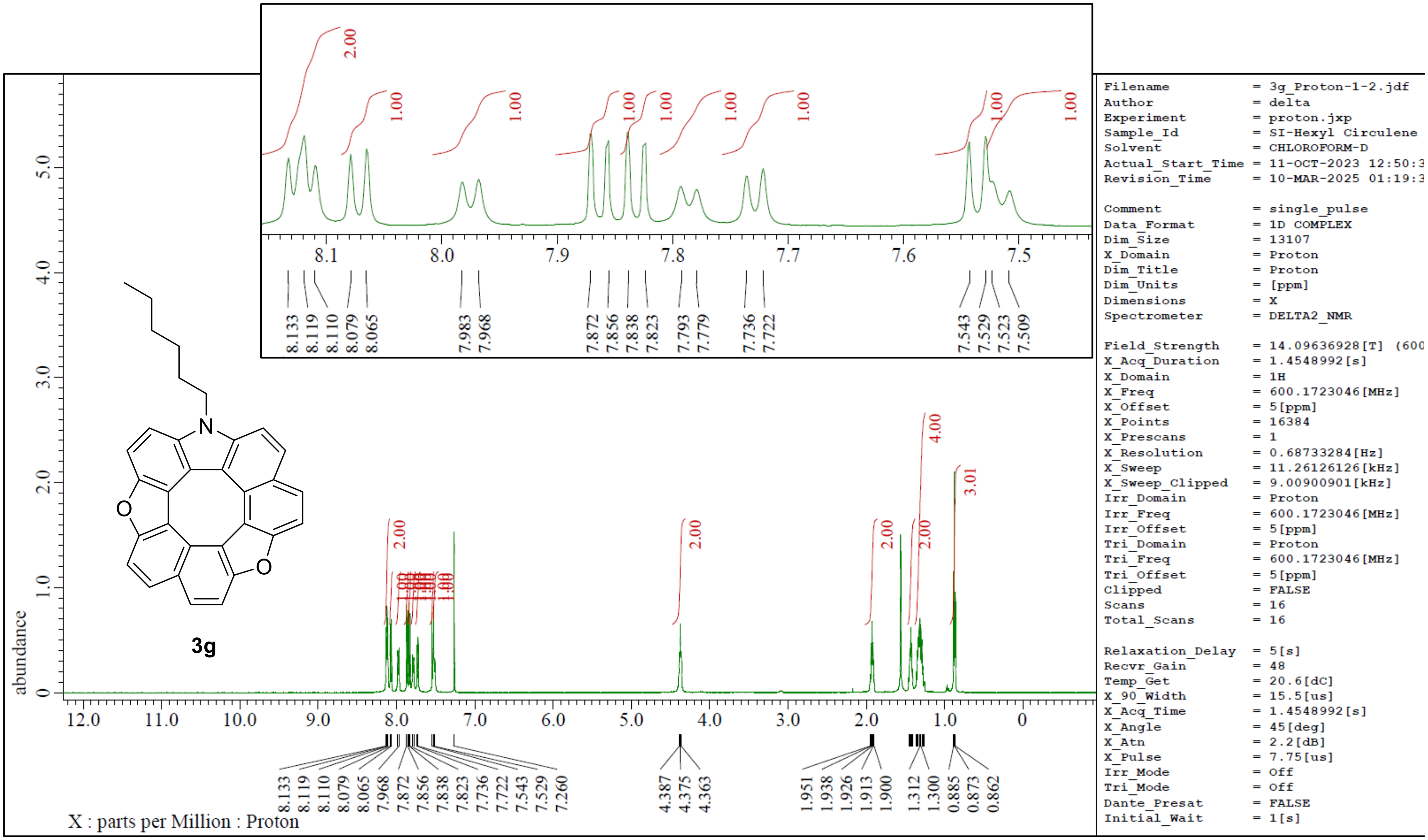

Compound **3g** (^{1}H NMR, 600 MHz, $CDCl_3$).

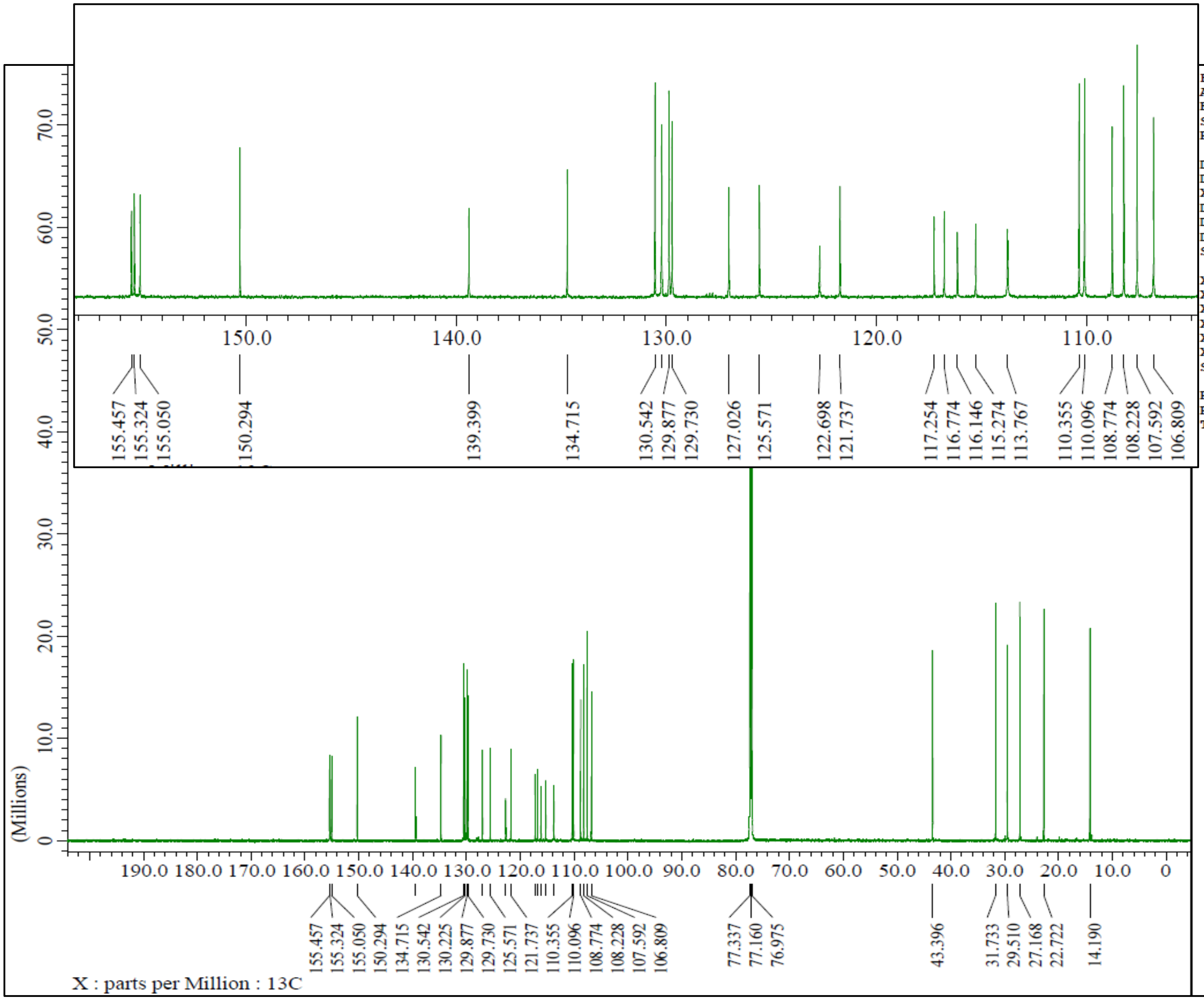

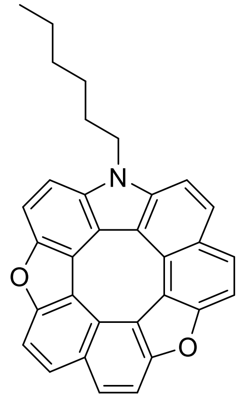

3g

Compound **3g** (^{13}C NMR, 175 MHz, $CDCl_3$).

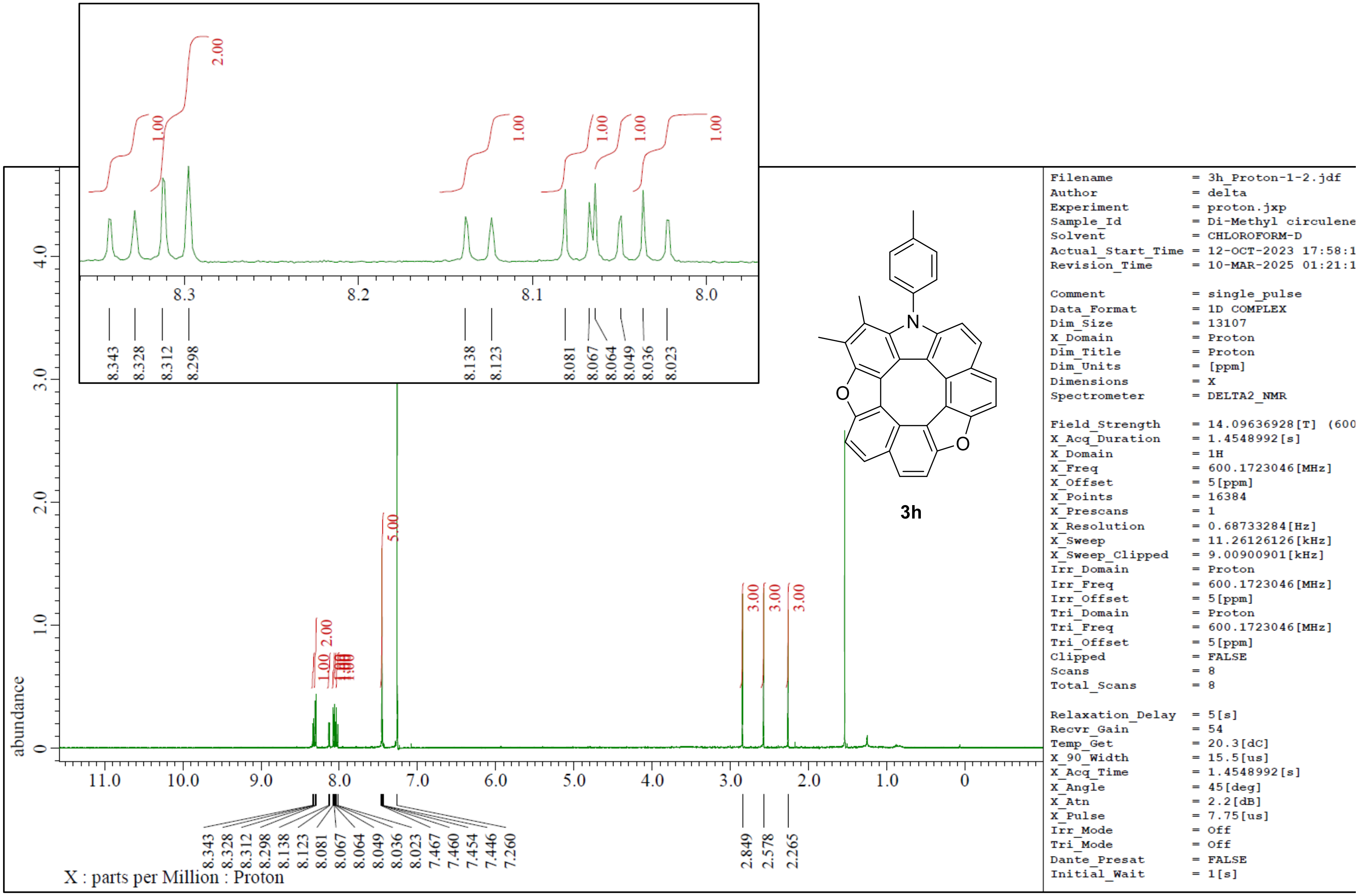

Compound **3h** (^{1}H NMR, 600 MHz, $CDCl_3$).

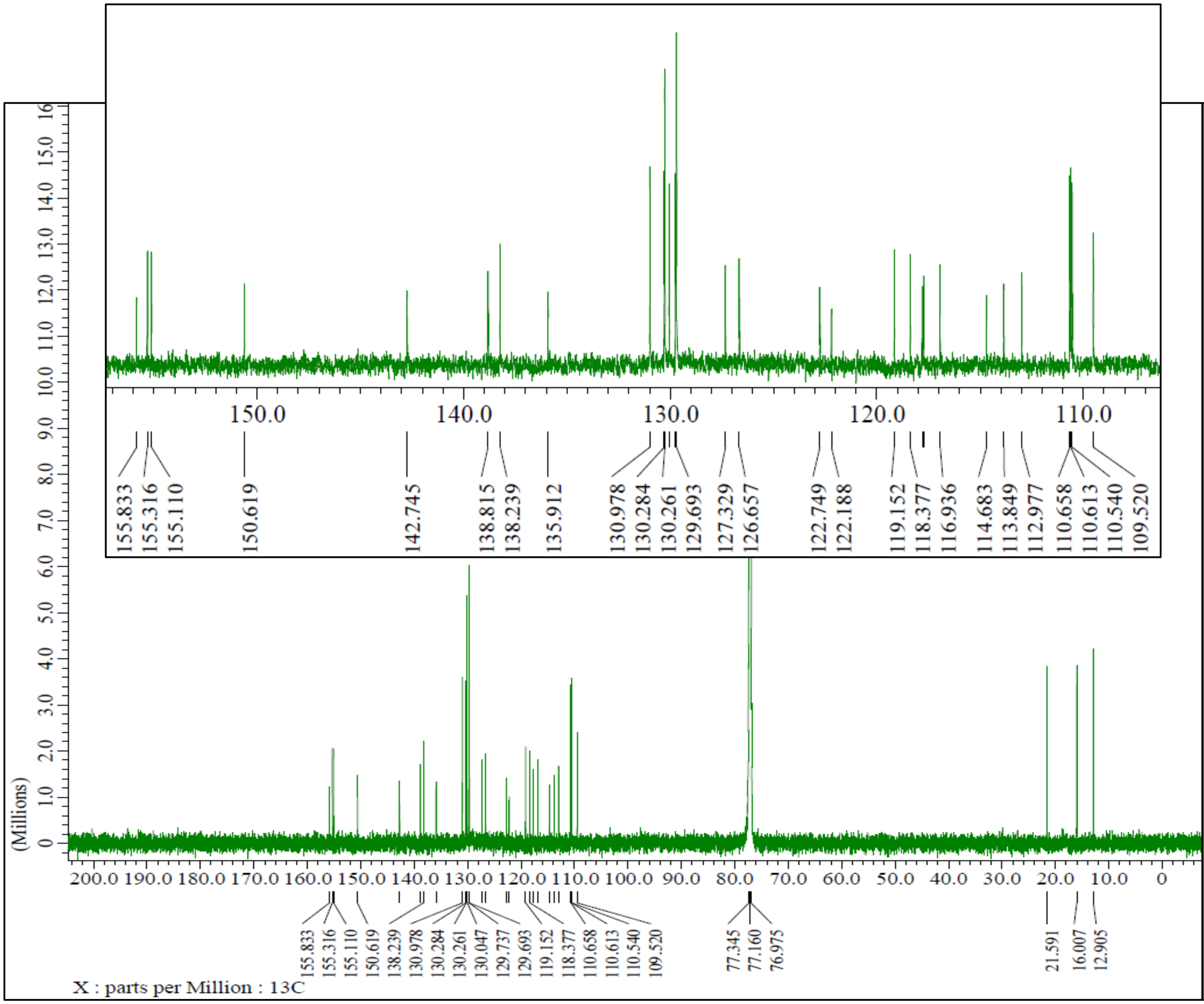

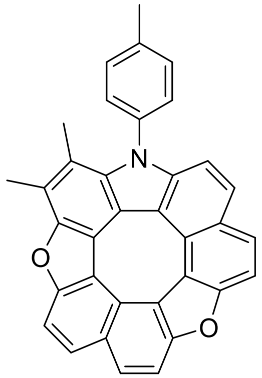

3h

Compound **3h** (^{13}C NMR, 175 MHz, $CDCl_3$).

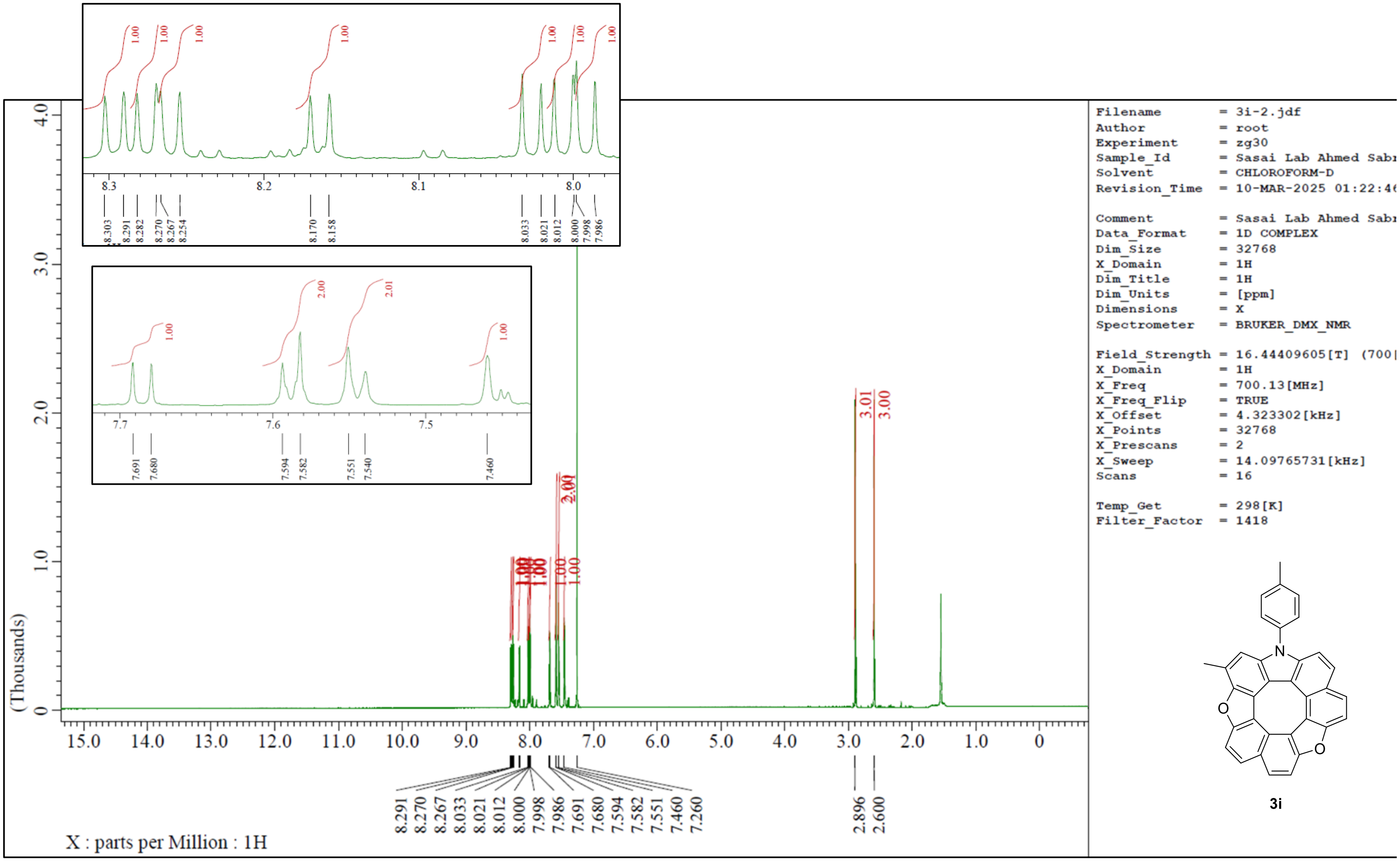

Compound **3i** (^{1}H NMR, 700 MHz, $CDCl_3$).

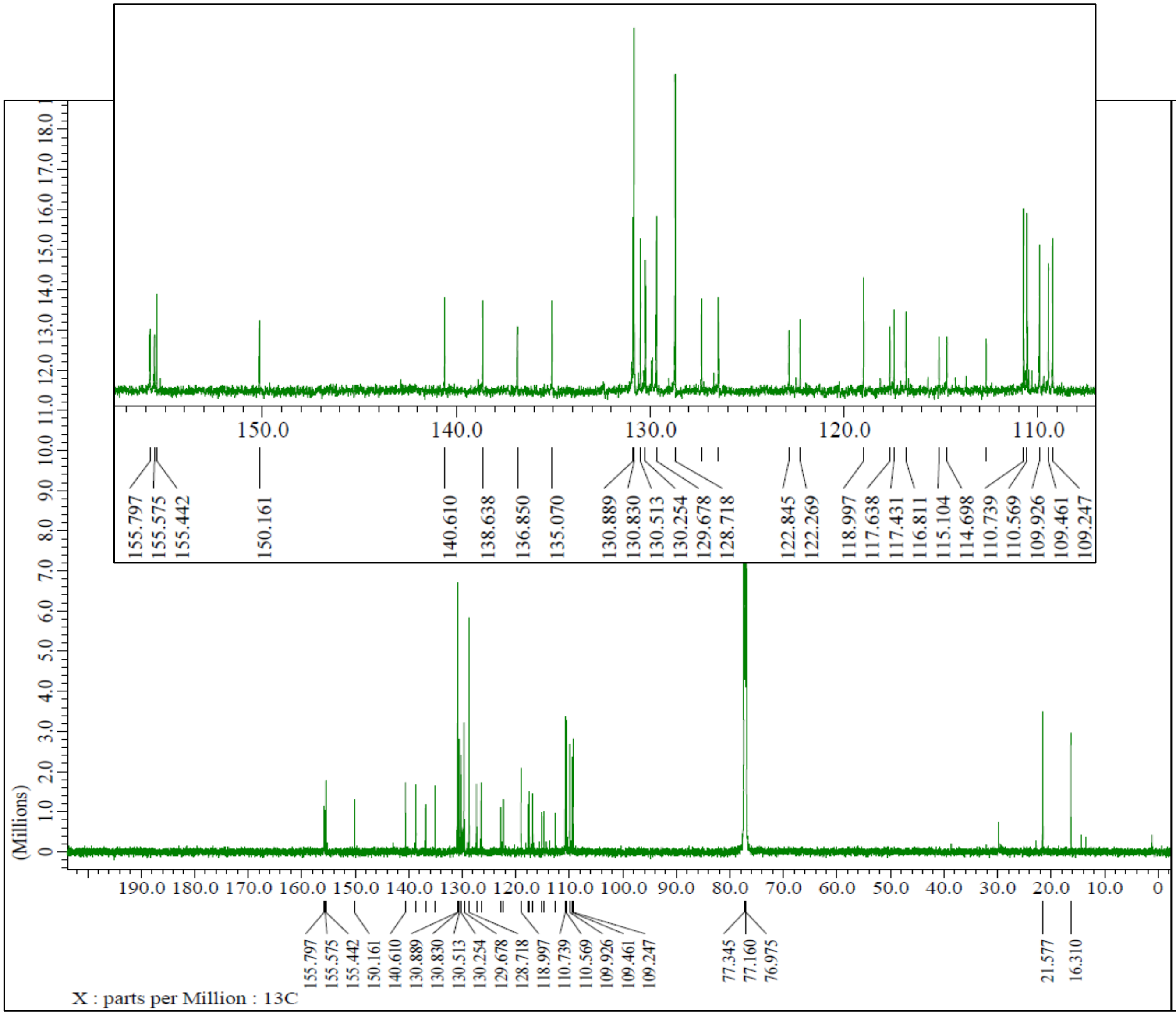

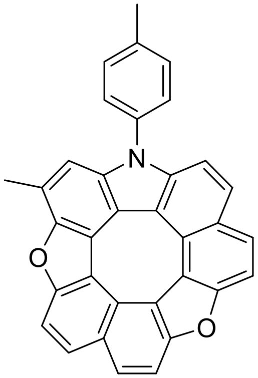

3i

Compound **3i** (^{13}C NMR, 175 MHz, $CDCl_3$).

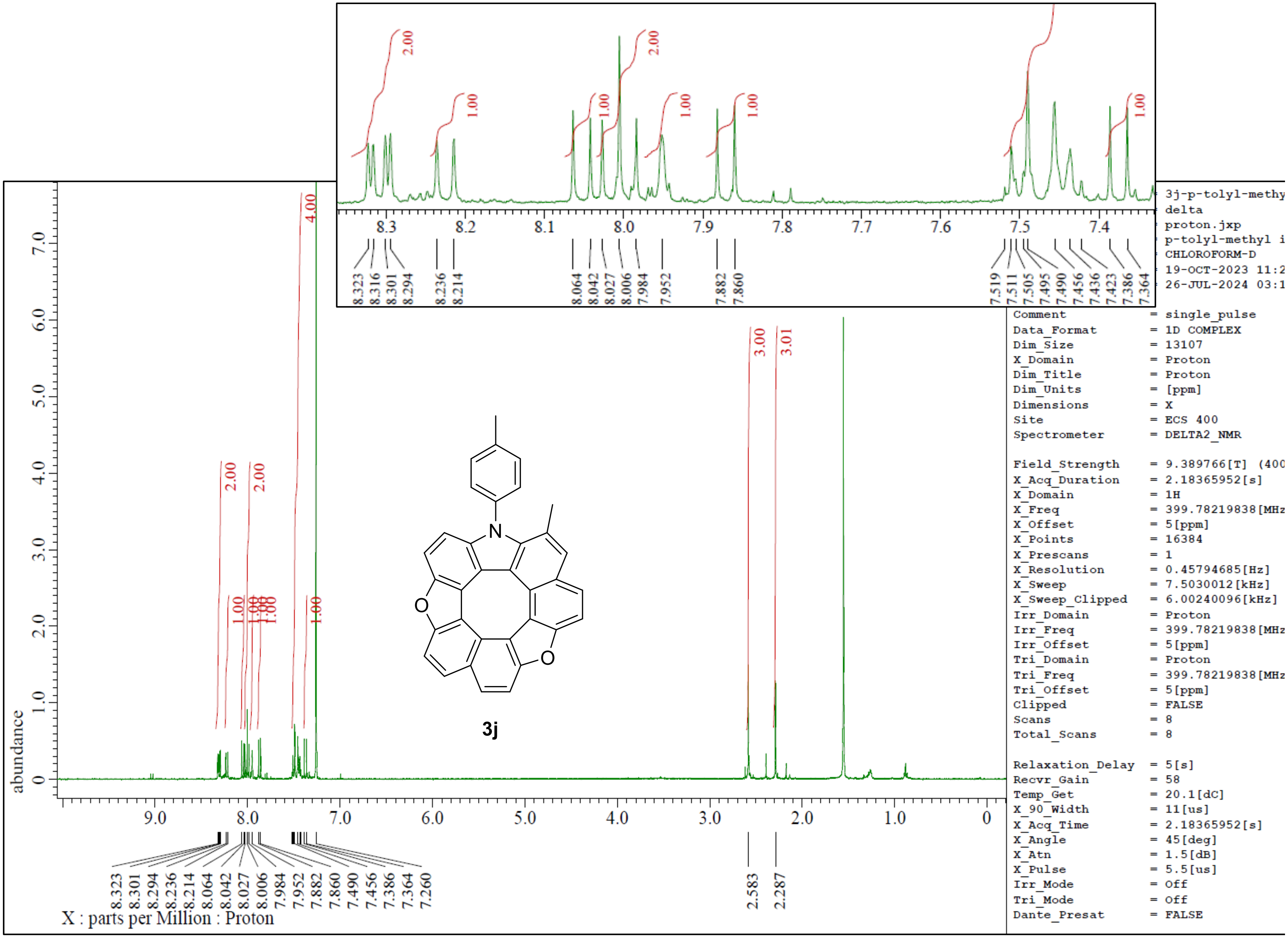

3j

Compound **3j** (^{1}H NMR, 400 MHz, $CDCl_3$).

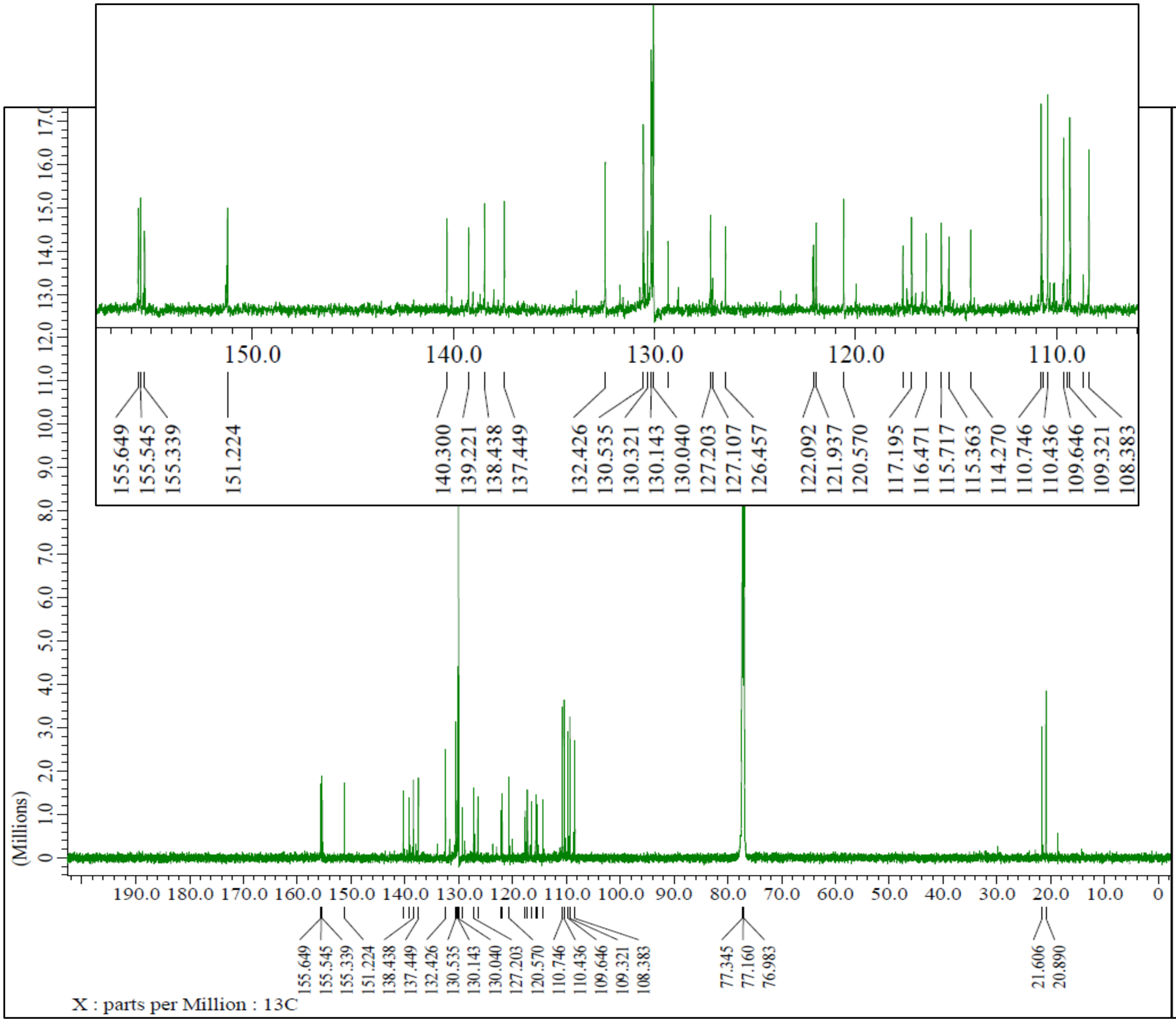

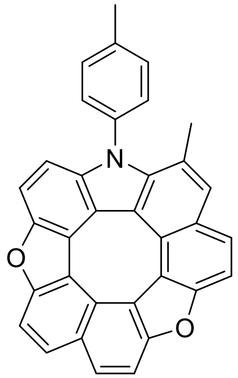

3j

Compound **3j** (^{13}C NMR, 175 MHz, $CDCl_3$).

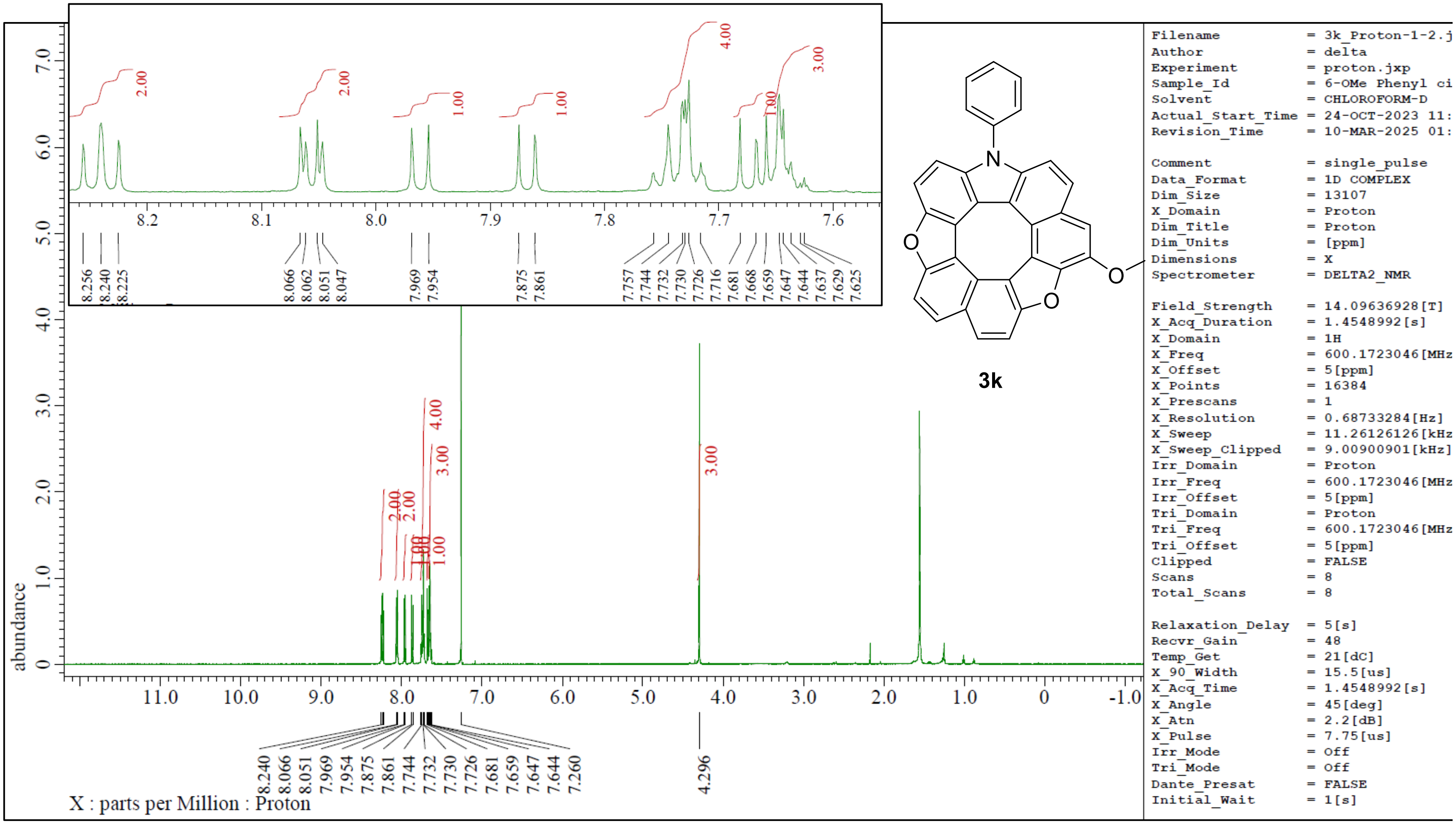

Compound **3k** (^{1}H NMR, 600 MHz, $CDCl_3$).

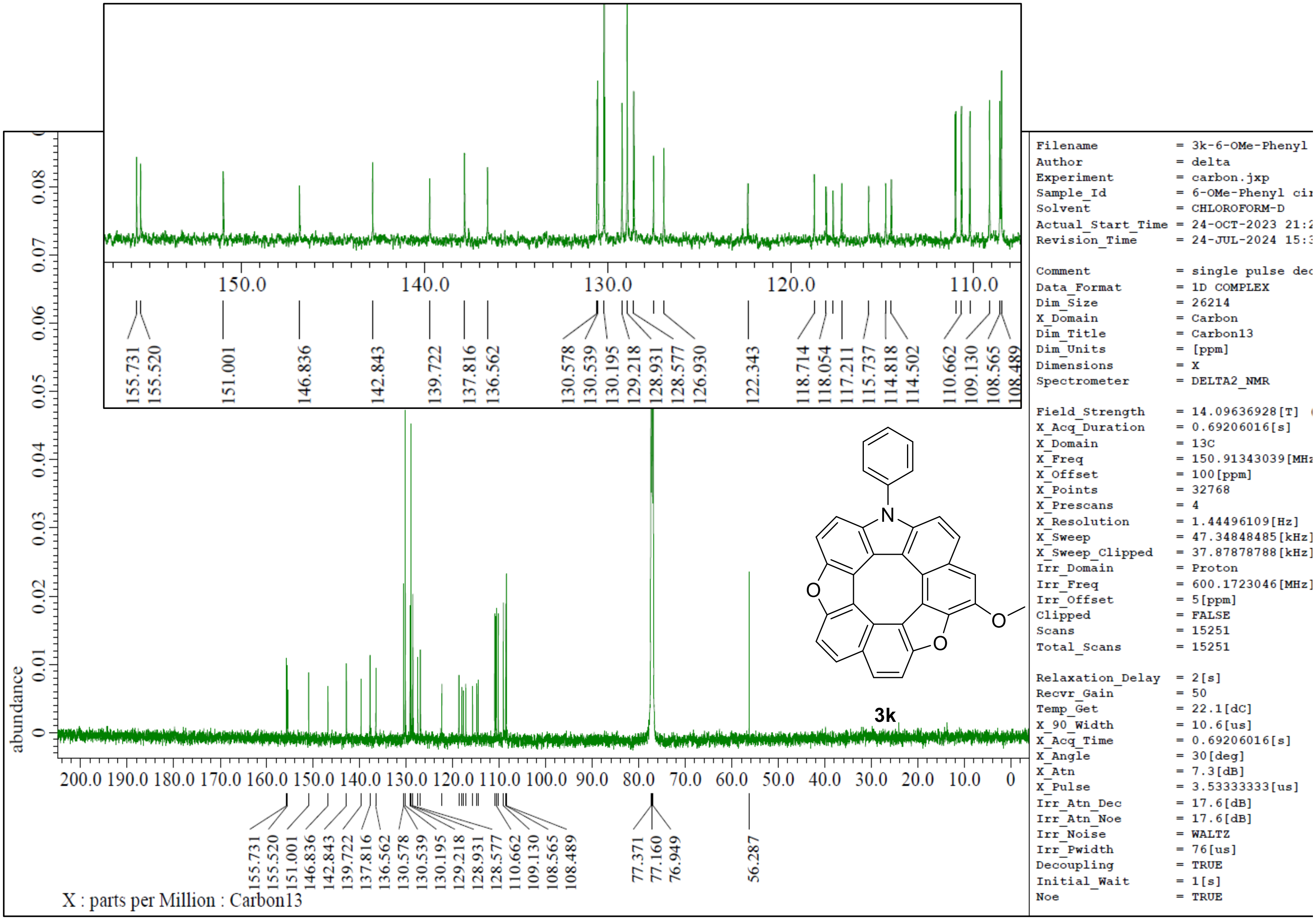

Compound **3k** (^{13}C NMR, 150 MHz, $CDCl_3$).

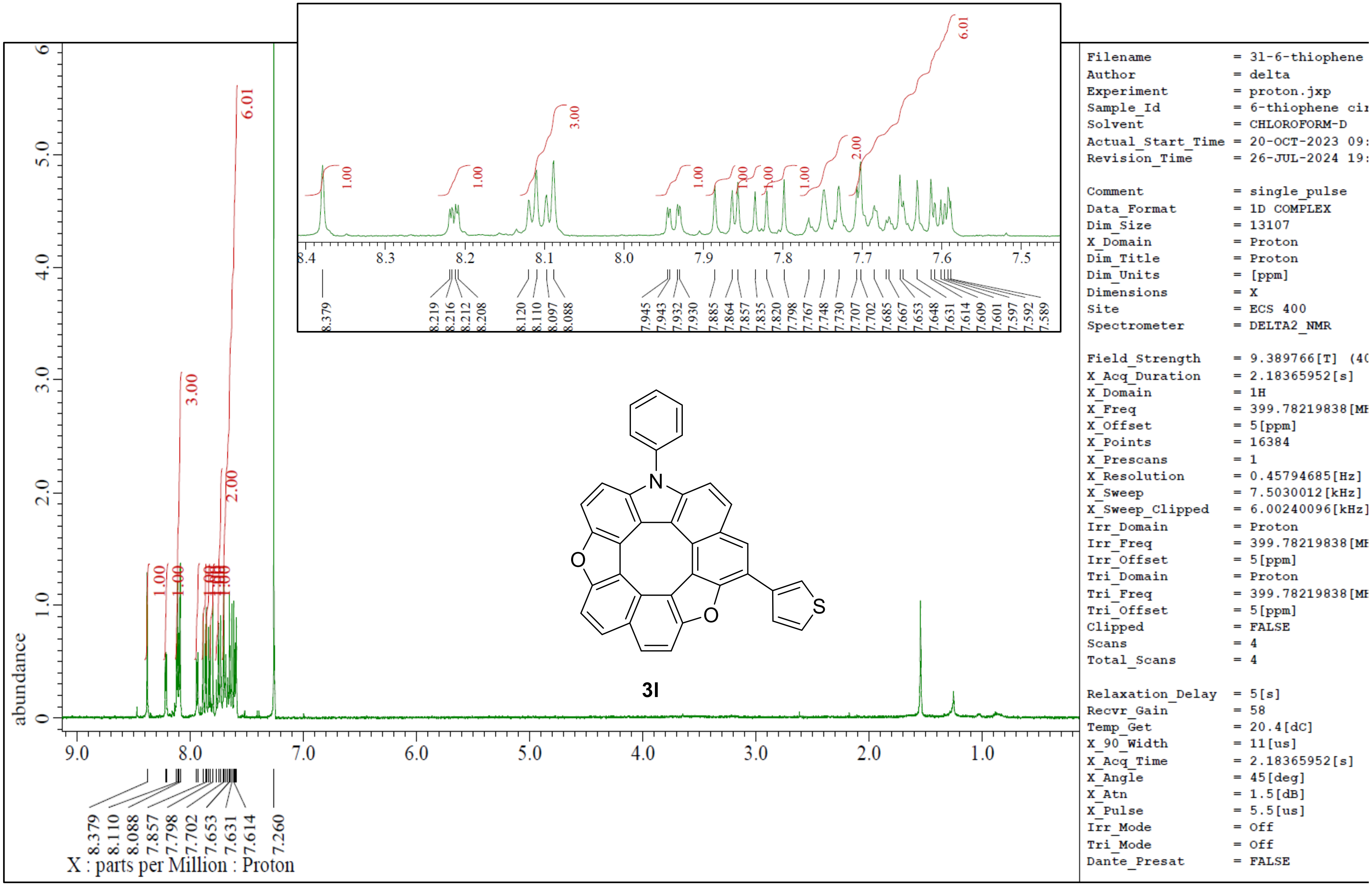

Compound **3l** (^{1}H NMR, 400 MHz, $CDCl_3$).

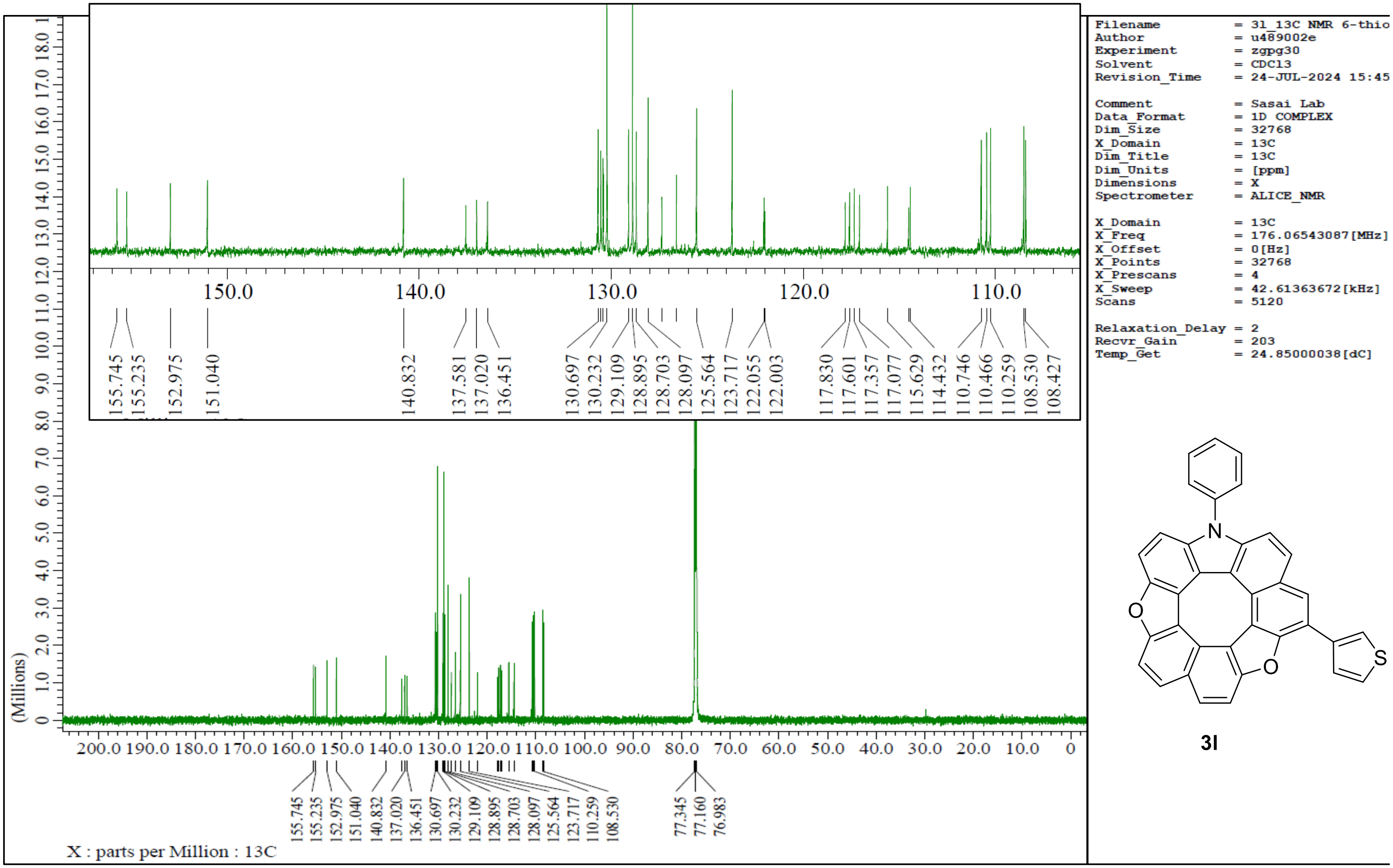

Compound **3l** (^{13}C NMR, 175 MHz, $CDCl_3$).

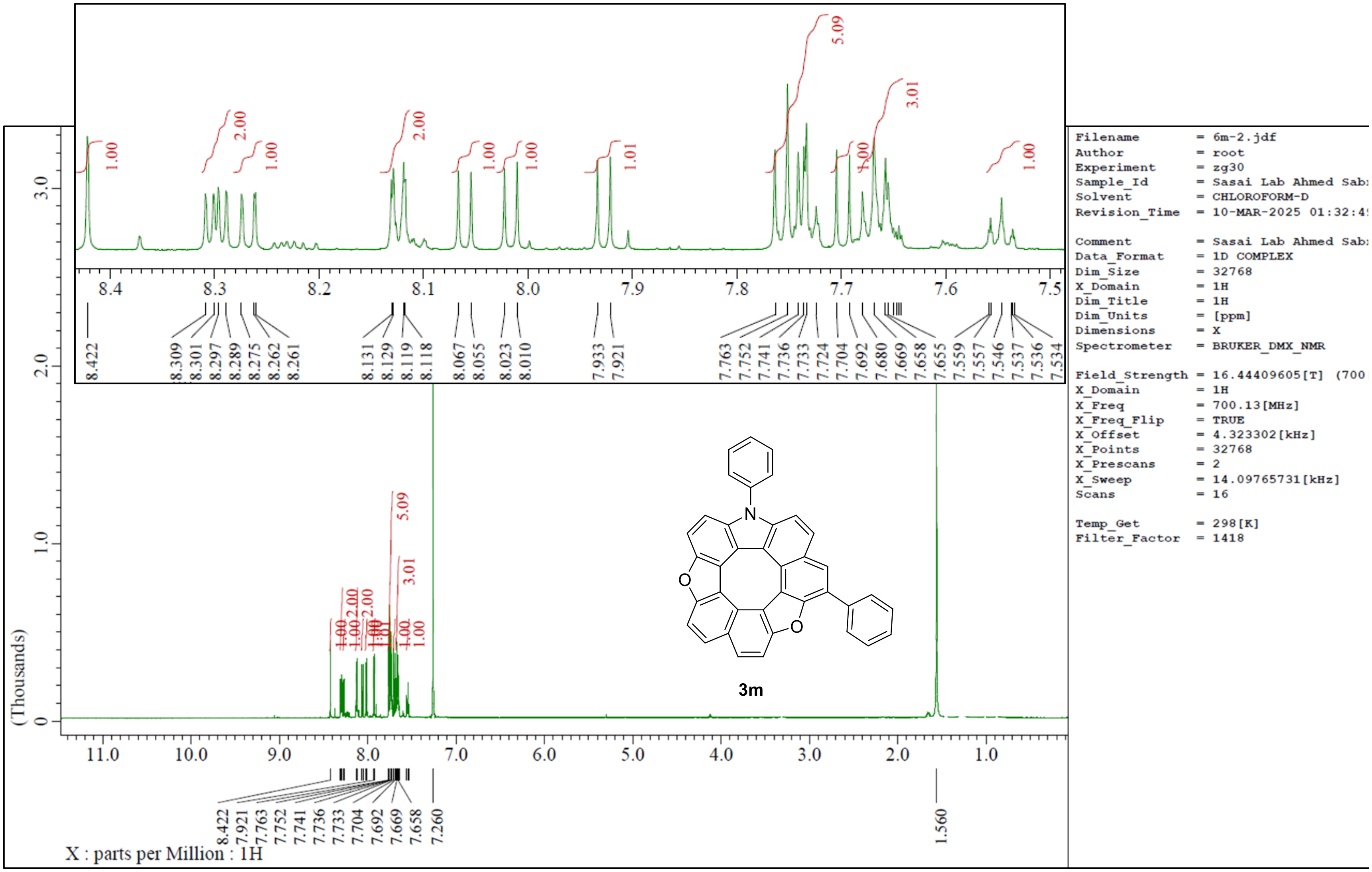

Compound **3m** (^{1}H NMR, 700 MHz, $CDCl_3$).

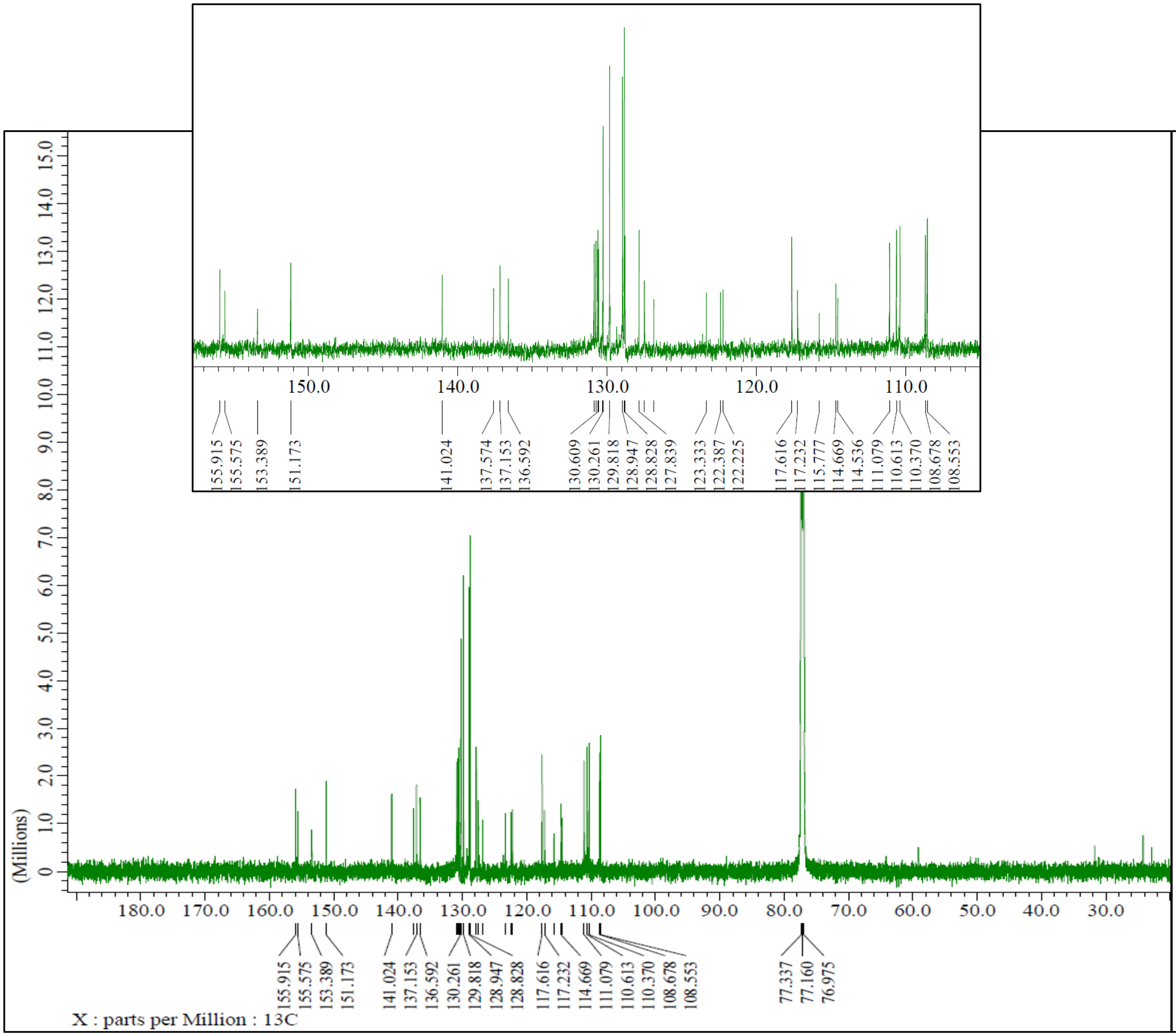

Filename = 6m-No.620_13C NM
Author = u489002e
Experiment = zgpg30
Solvent = CDCl3
Revision_Time = 24-JUL-2024 15:4

Comment = Sasai Lab
Data_Format = 1D COMPLEX
Dim_Size = 32768
X_Domain = 13C
Dim_Title = 13C
Dim_Units = [ppm]
Dimensions = X
Spectrometer = ALICE_NMR

X_Domain = 13C
X_Freq = 176.06543087[MHz
X_Offset = 0[Hz]
X_Points = 32768
X_Prescans = 4
X_Sweep = 42.61363672[kHz]
Scans = 5120

Relaxation_Delay = 2
Recvr_Gain = 203
Temp_Get = 24.85000038[dC]

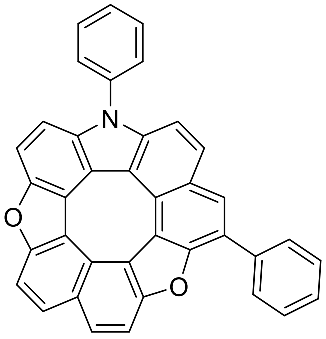

3m

Compound **3m** (^{13}C NMR, 175 MHz, $CDCl_3$).

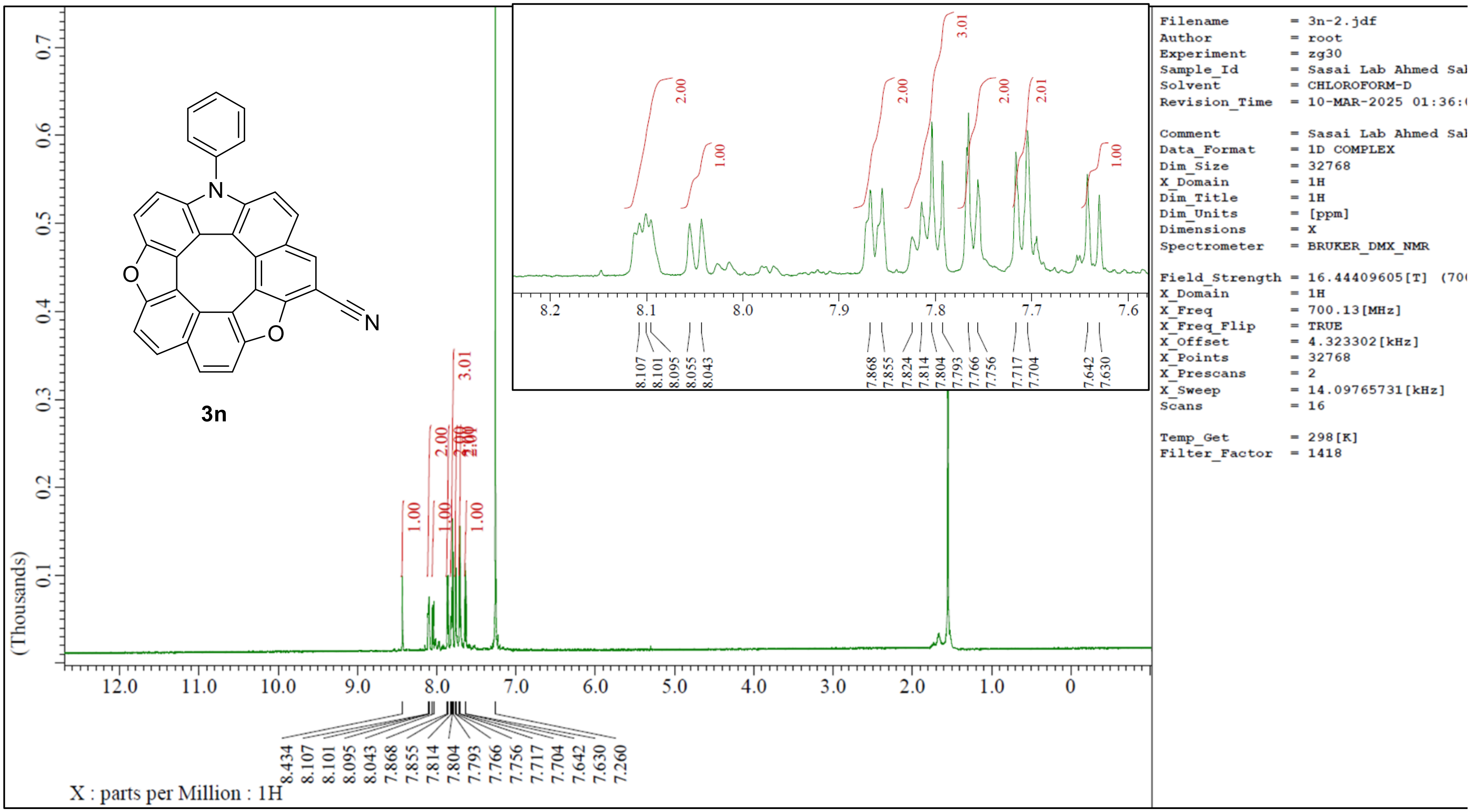

Compound **3n** (^{1}H NMR, 700 MHz, $CDCl_3$).

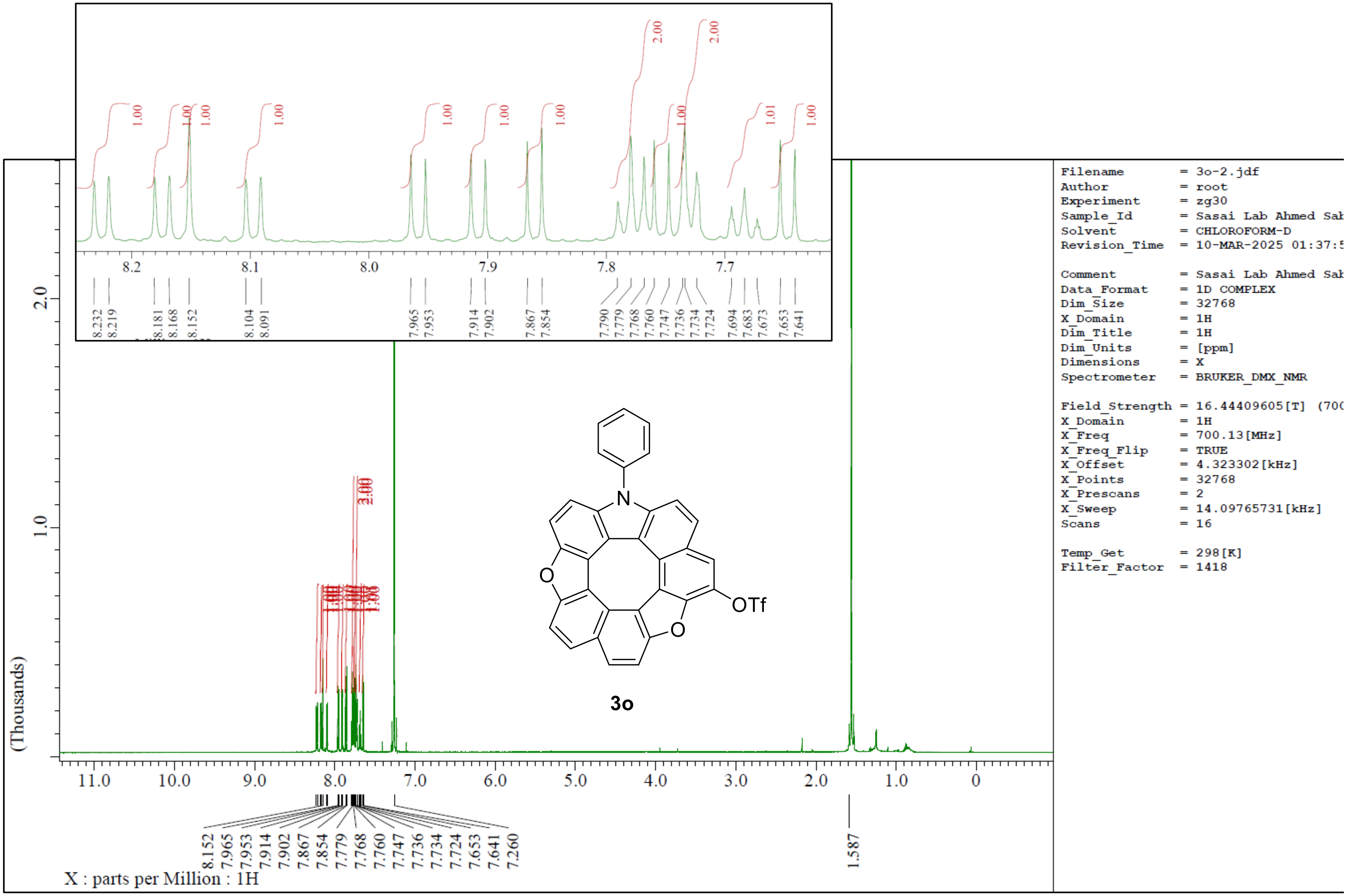

Compound **3o** (^{1}H NMR, 700 MHz, $CDCl_3$).

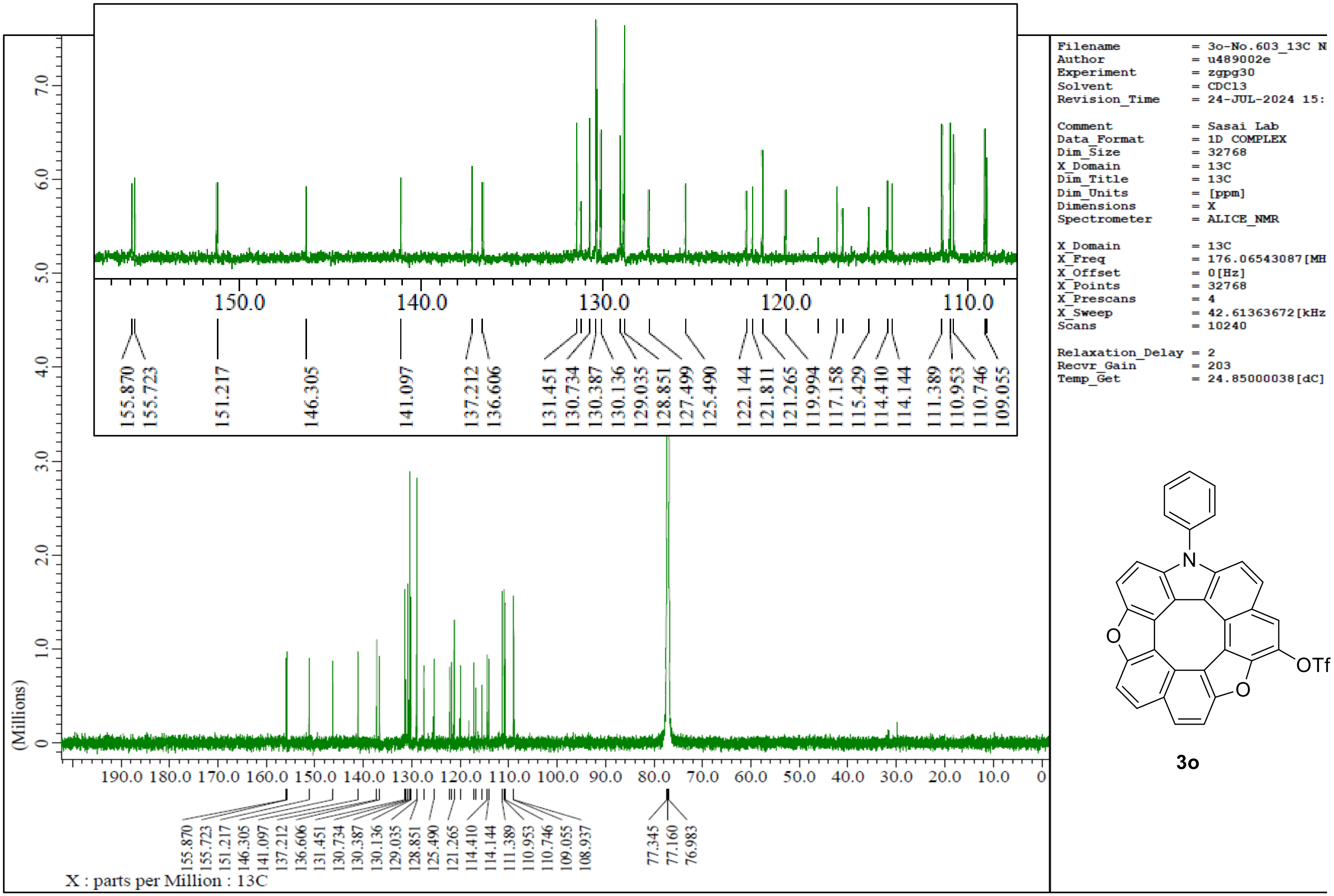

Compound **3o** (^{13}C NMR, 175 MHz, $CDCl_3$).

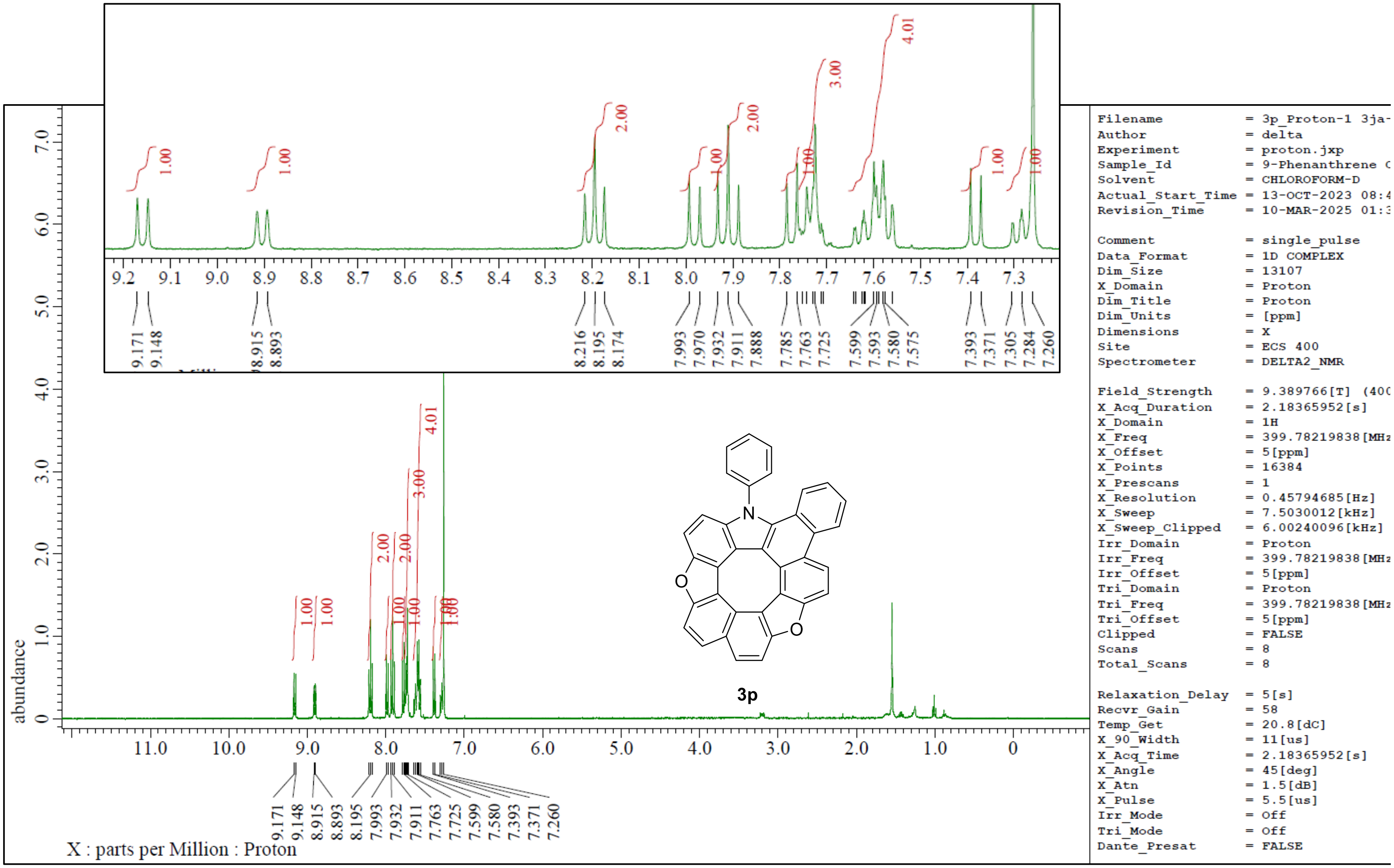

Compound **3p** (^{1}H NMR, 400 MHz, $CDCl_3$).

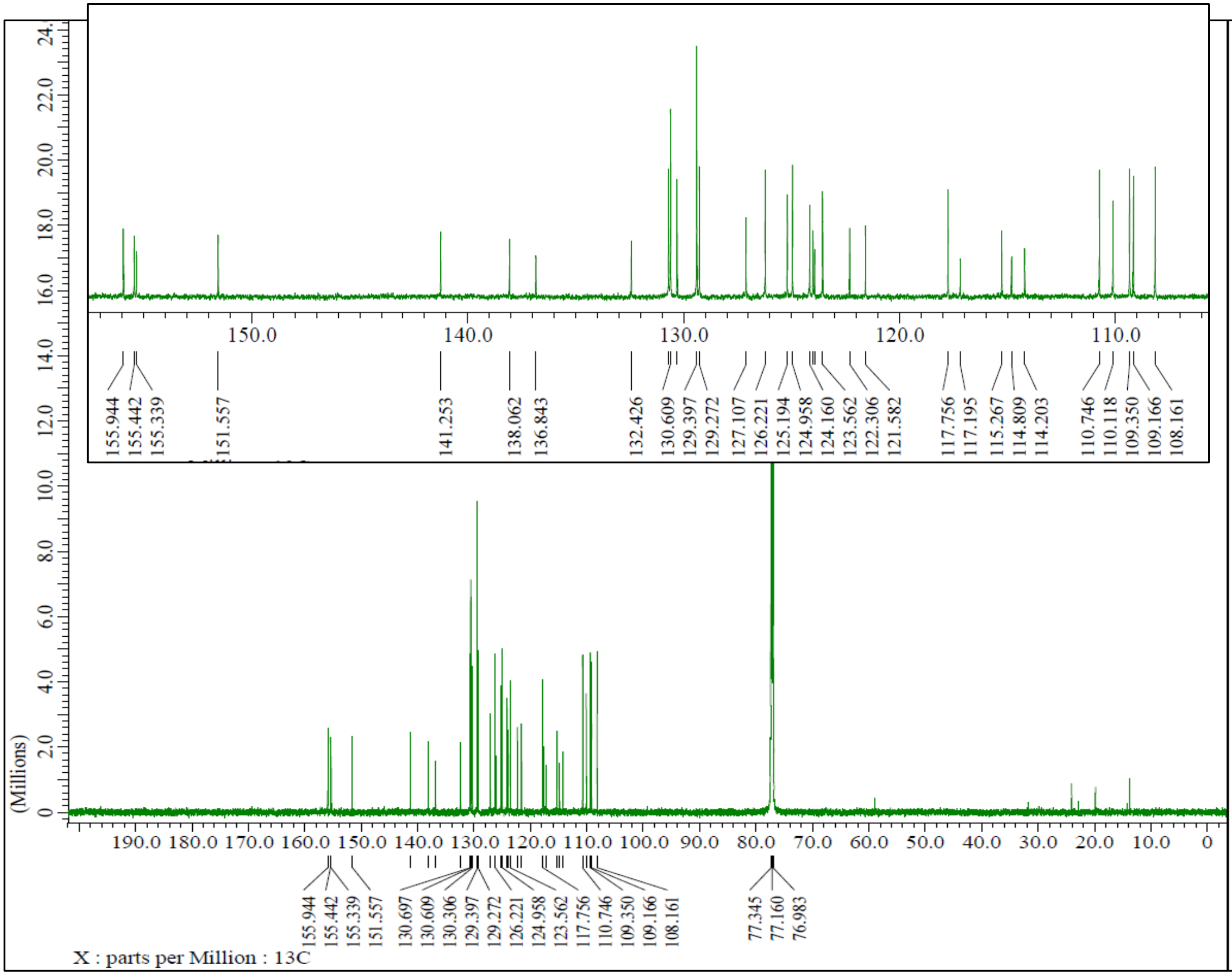

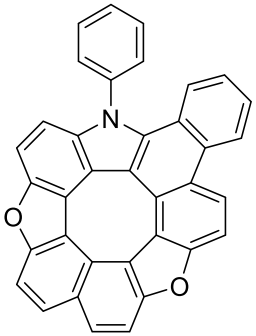

3p

Compound **3p** (^{13}C NMR, 175 MHz, $CDCl_3$).

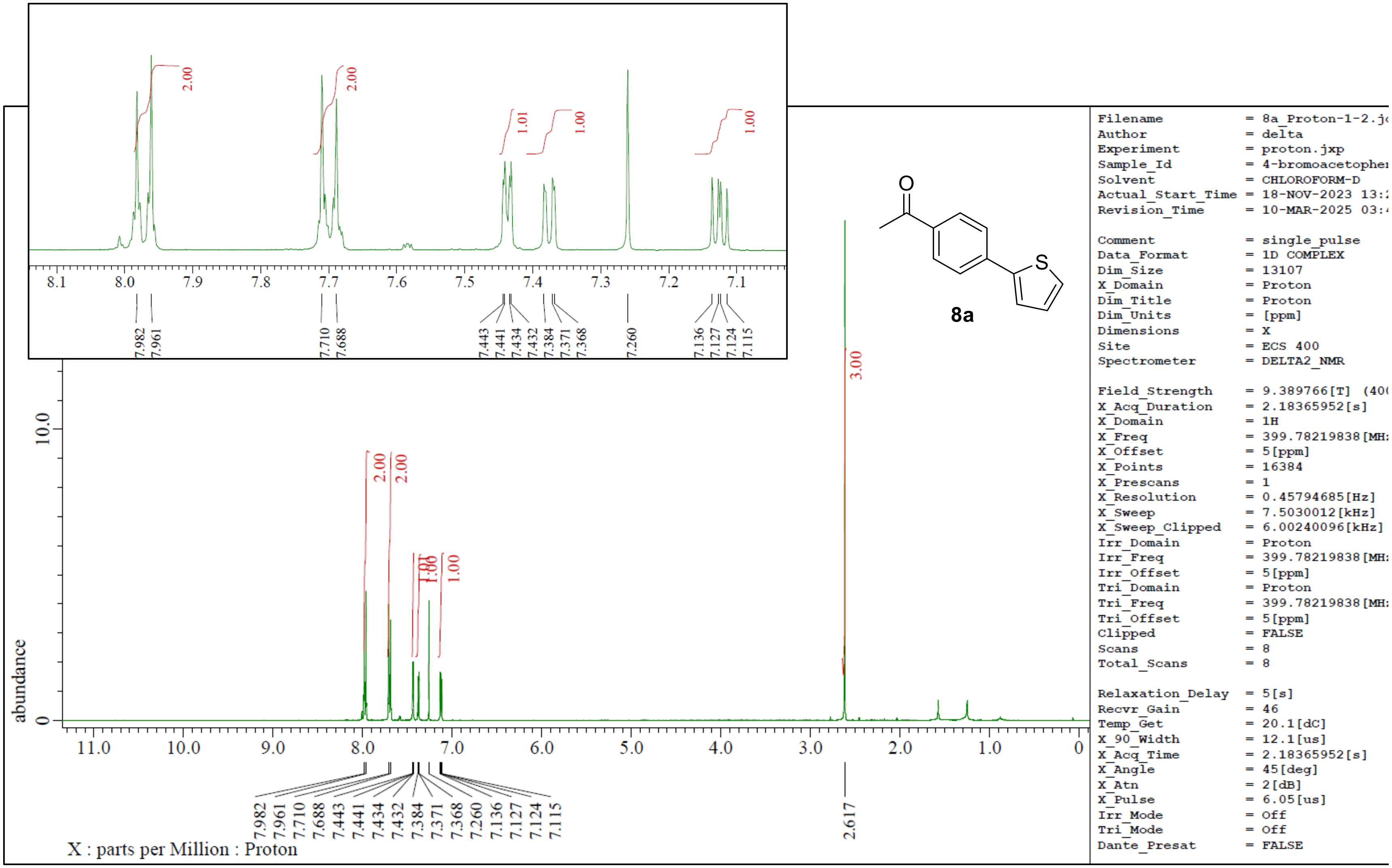

Compound **8a** (^{1}H NMR, 400 MHz, $CDCl_3$).

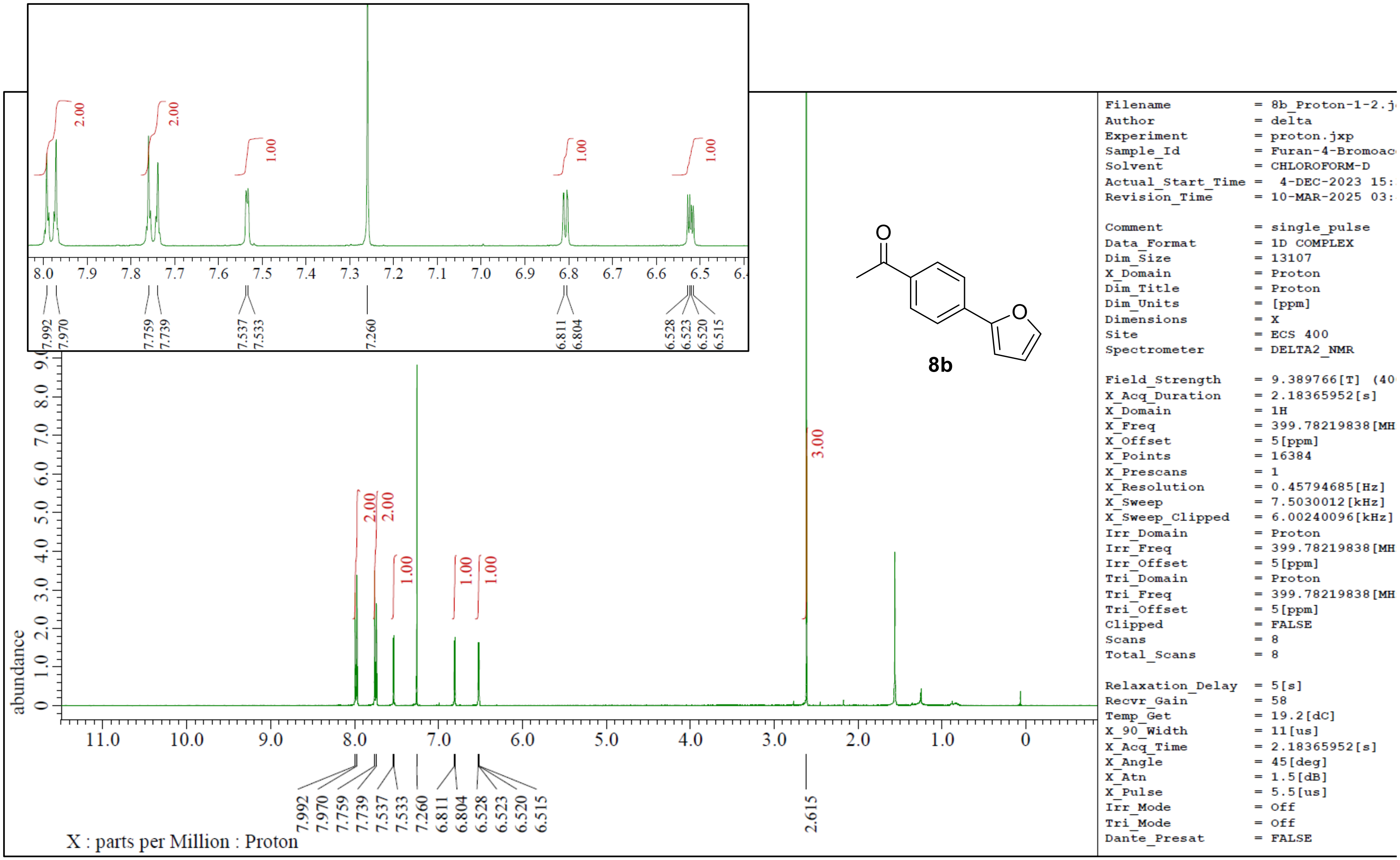

Compound **8b** (^{1}H NMR, 400 MHz, $CDCl_3$).

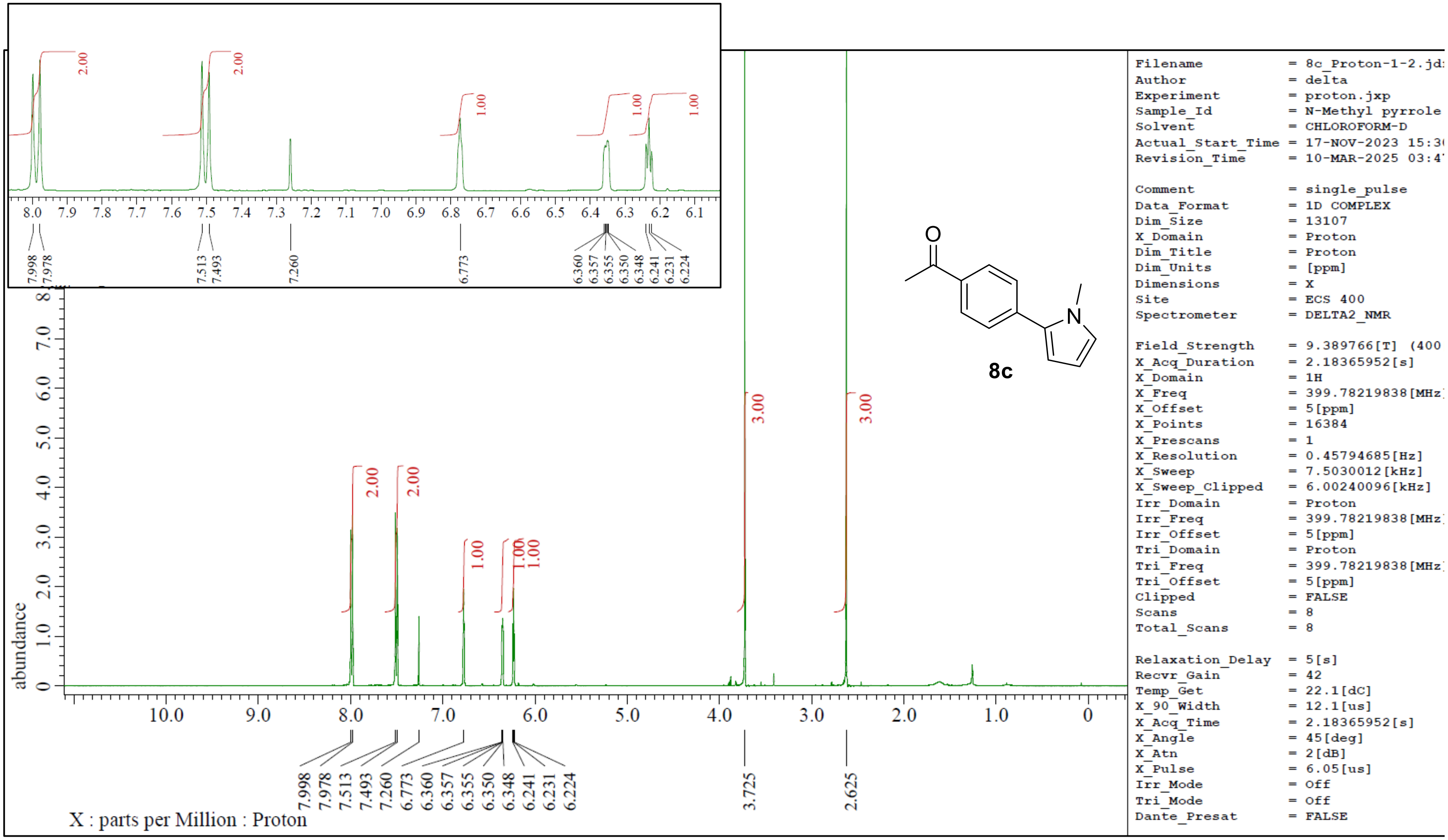

Compound **8c** (^{1}H NMR, 400 MHz, $CDCl_3$).

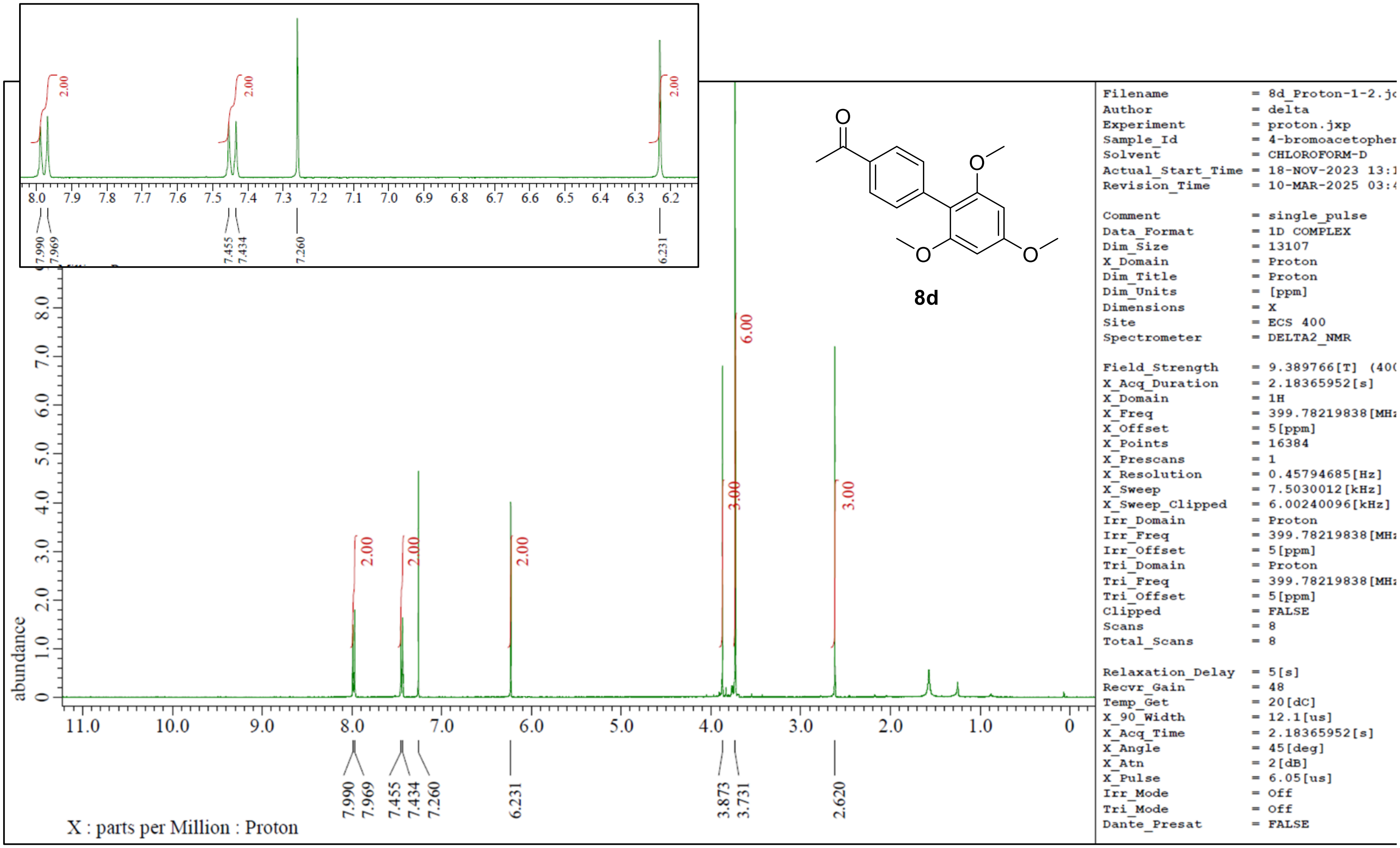

Compound **8d** (^{1}H NMR, 400 MHz, $CDCl_3$).

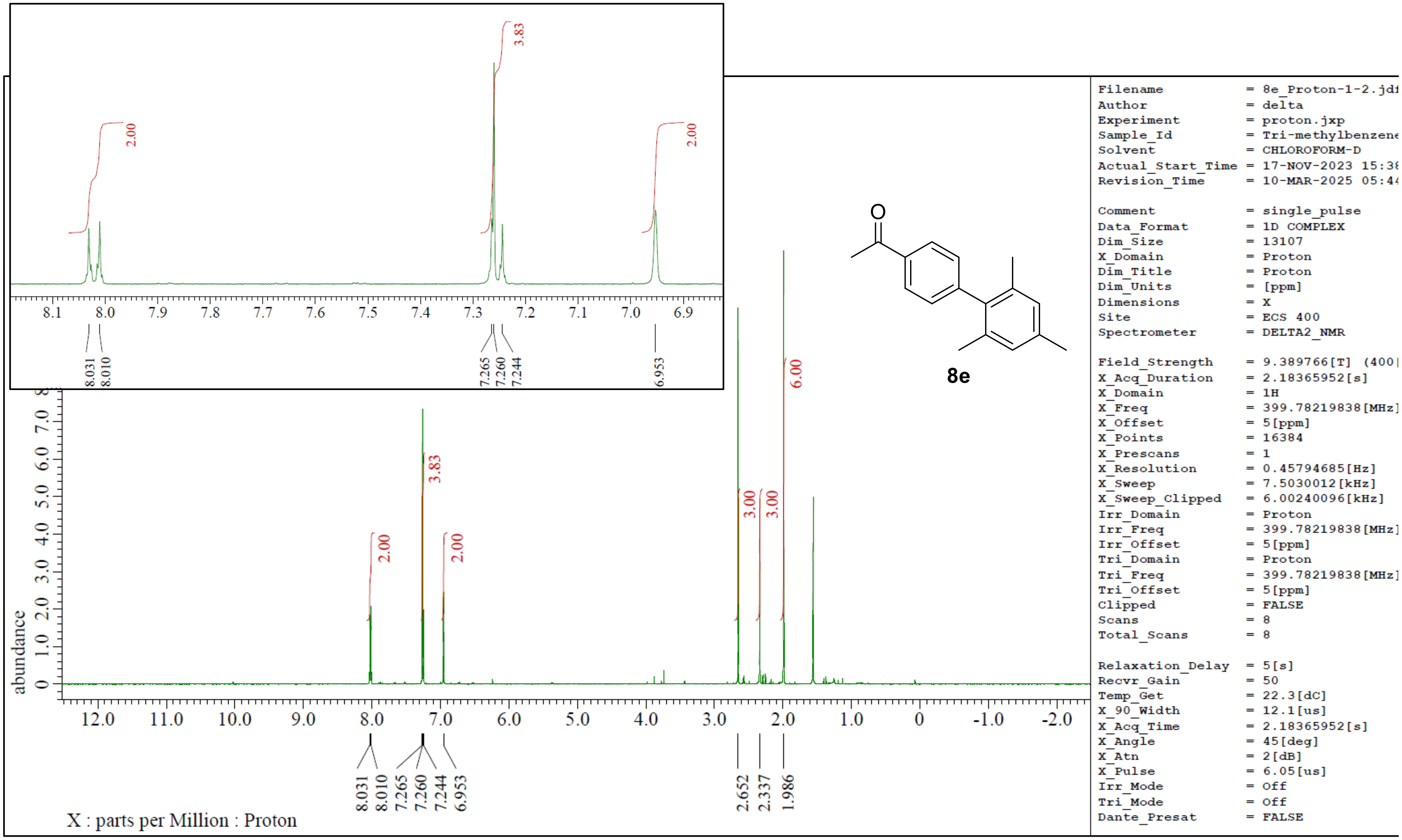

Compound **8e** (^{1}H NMR, 400 MHz, $CDCl_3$).

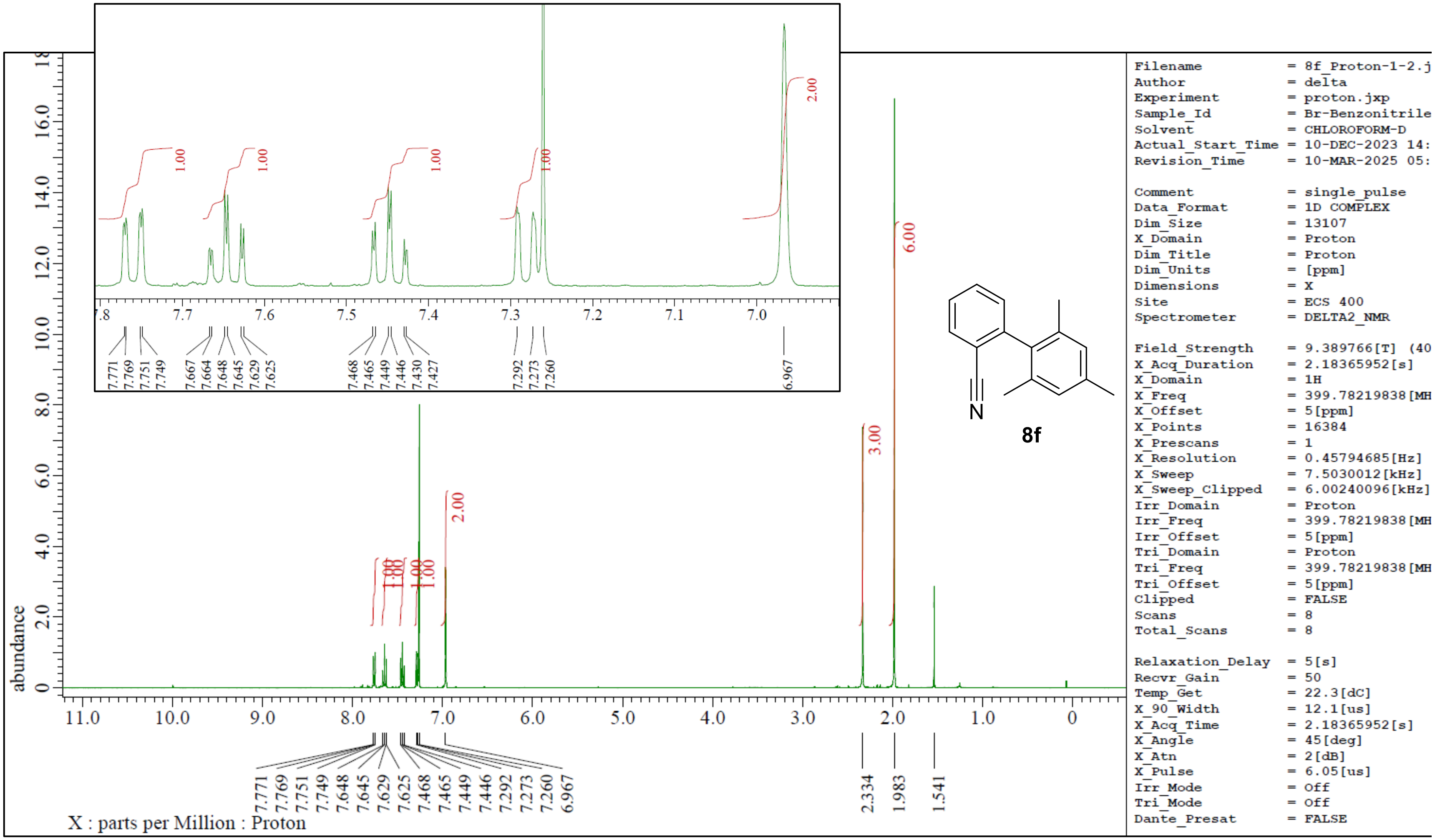

Compound **8f** (^{1}H NMR, 400 MHz, $CDCl_3$).

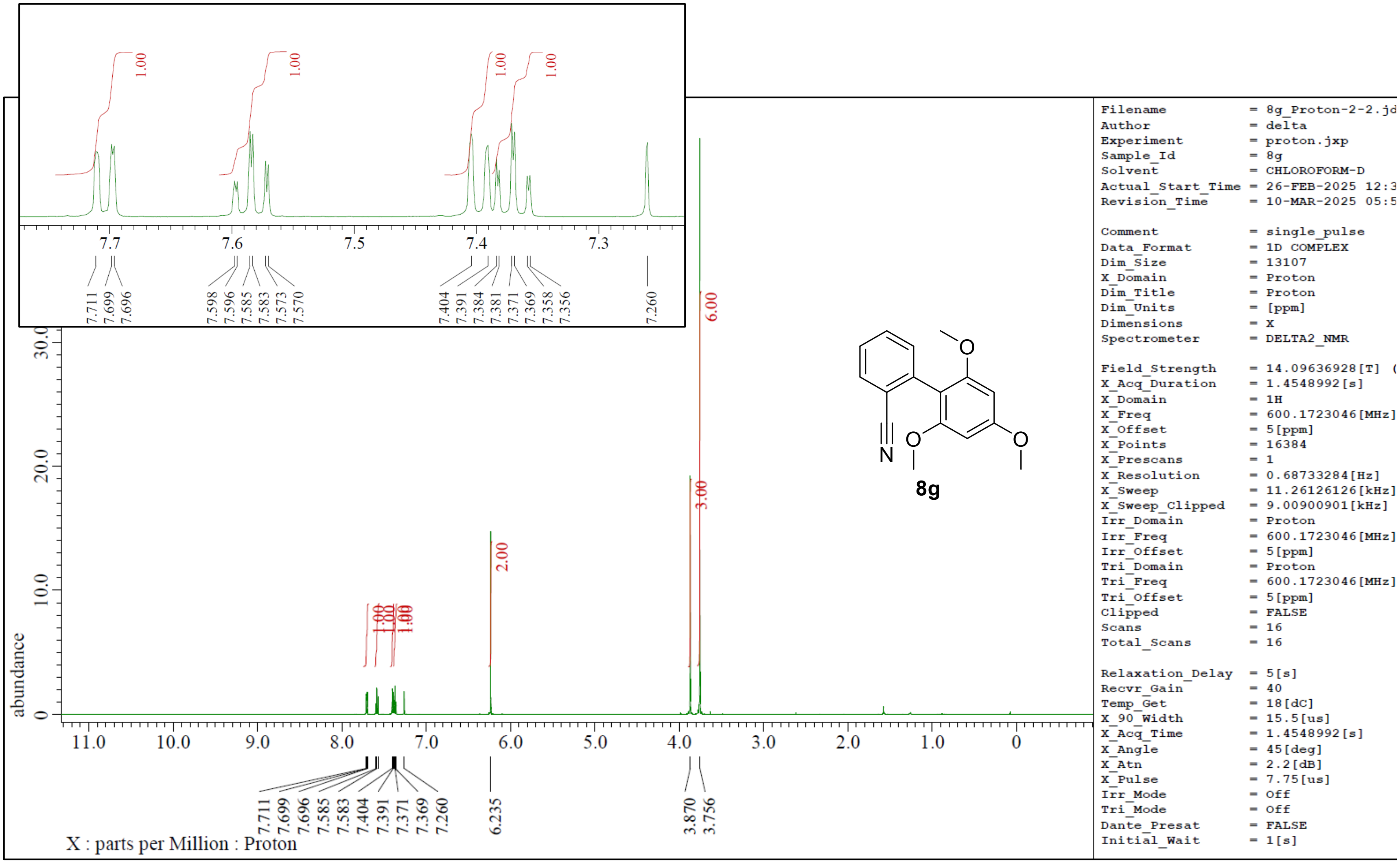

Compound **8g** (^{1}H NMR, 600 MHz, $CDCl_3$).

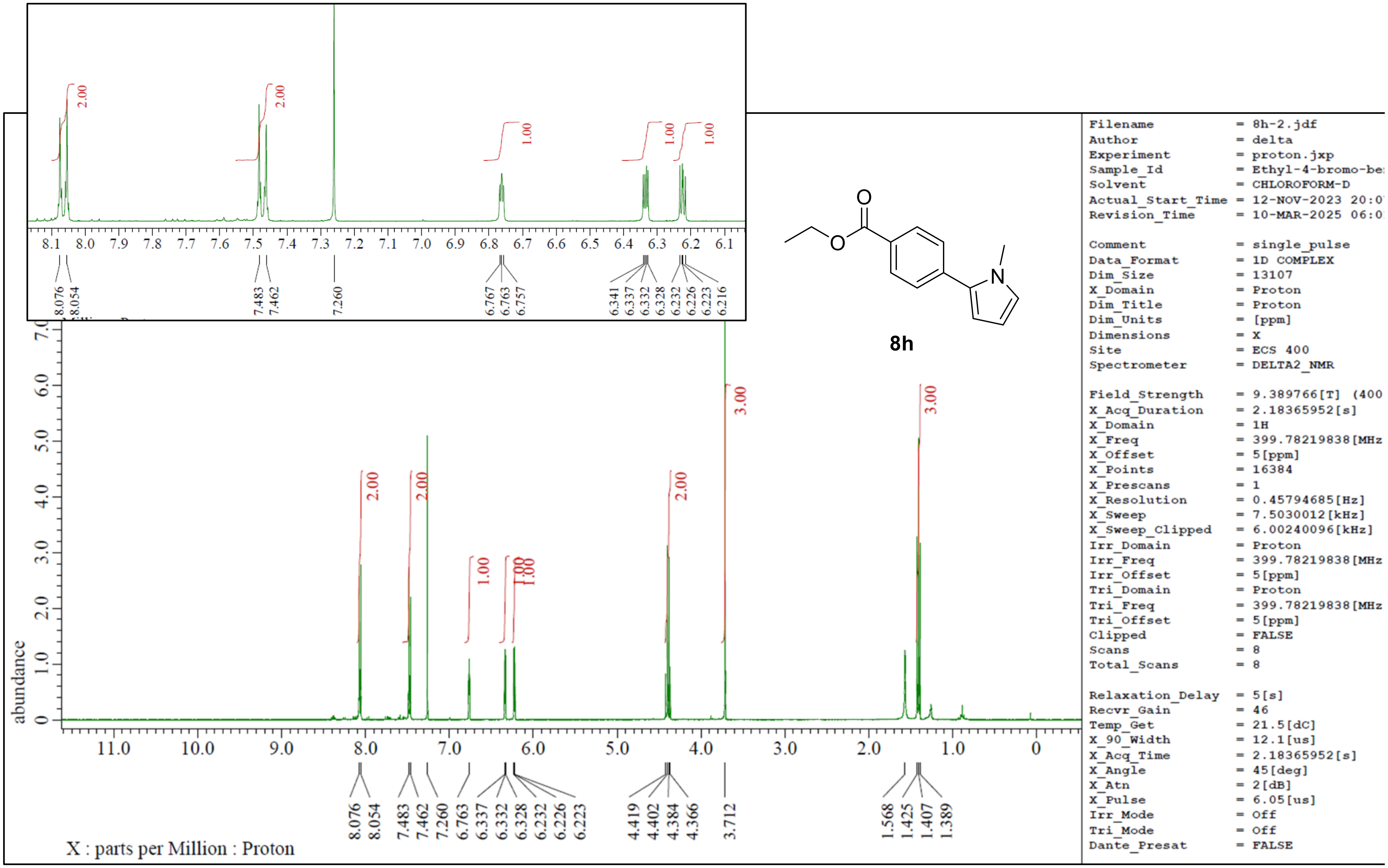

Compound **8h** (^{1}H NMR, 400 MHz, $CDCl_3$).

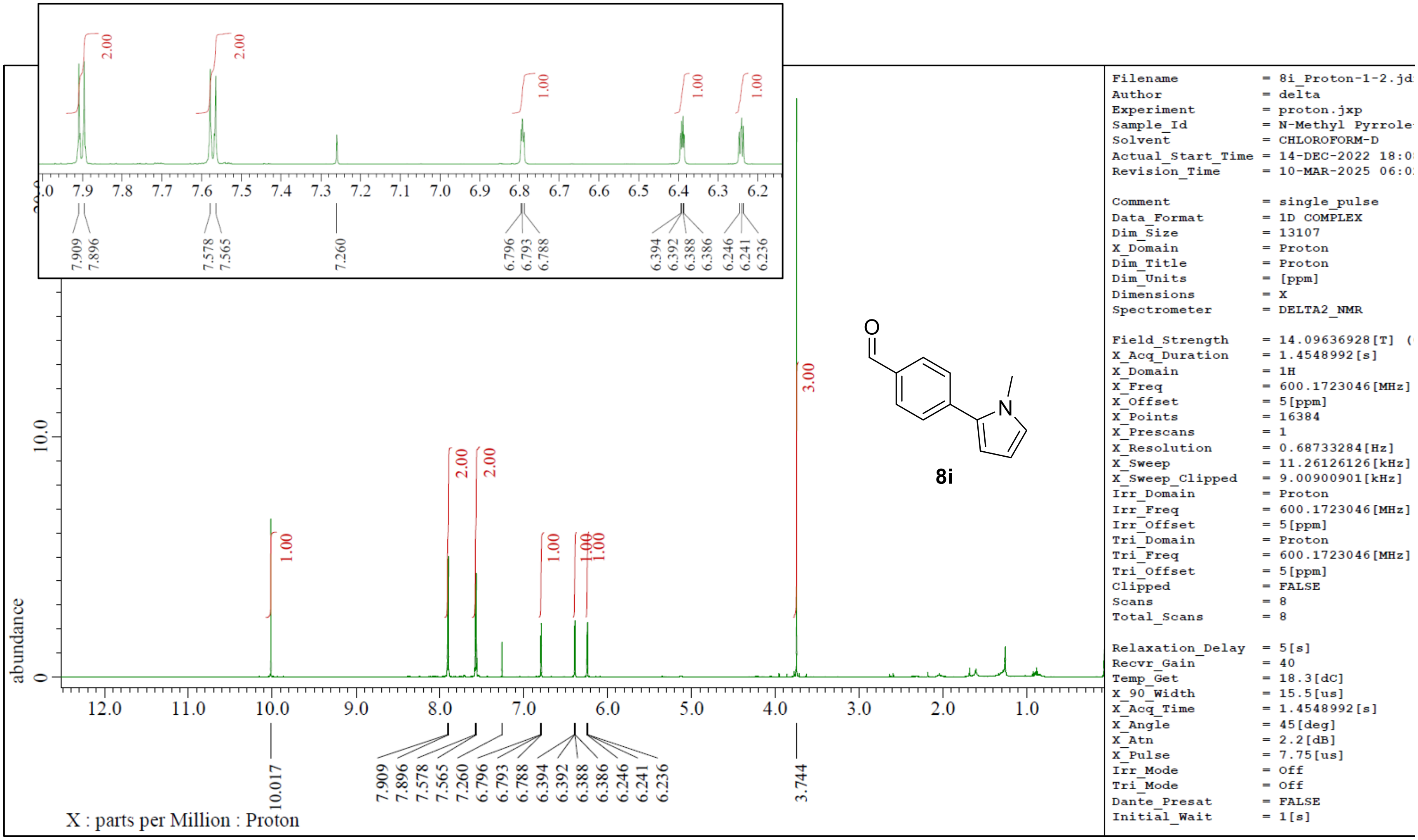

Compound **8i** (^{1}H NMR, 600 MHz, $CDCl_3$).

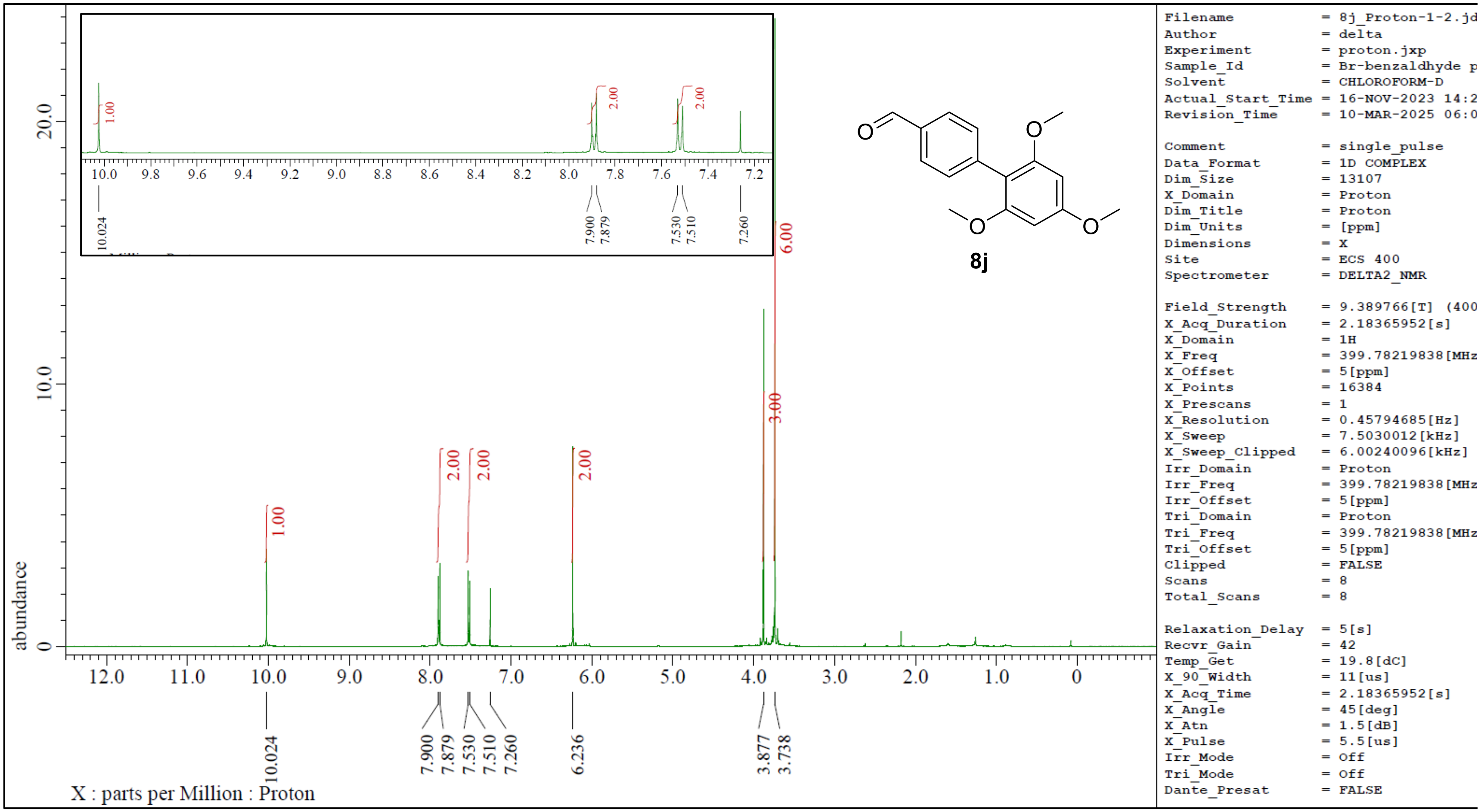

Compound **8j** (^{1}H NMR, 400 MHz, $CDCl_3$).

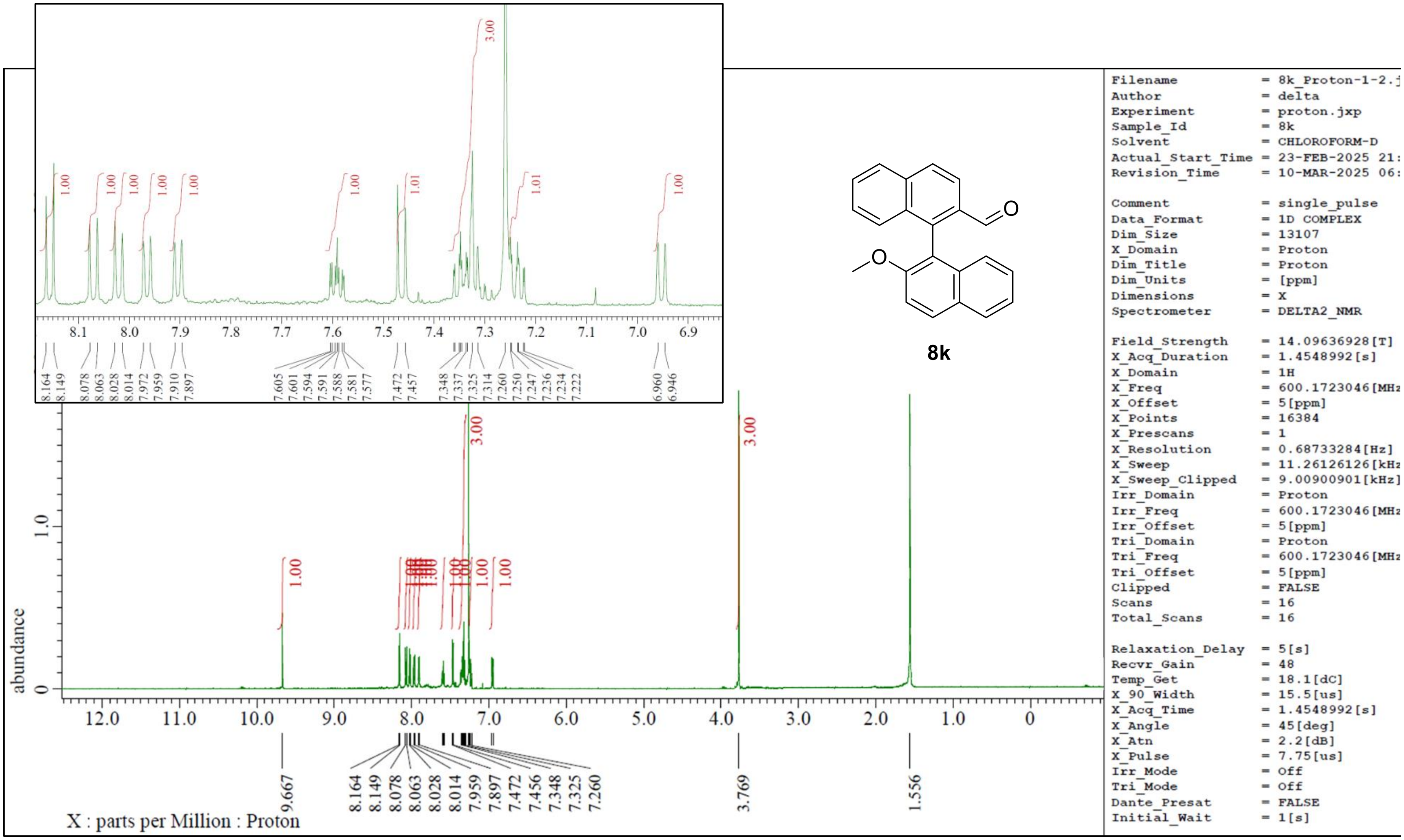

Compound **8k** (^{1}H NMR, 600 MHz, $CDCl_3$).

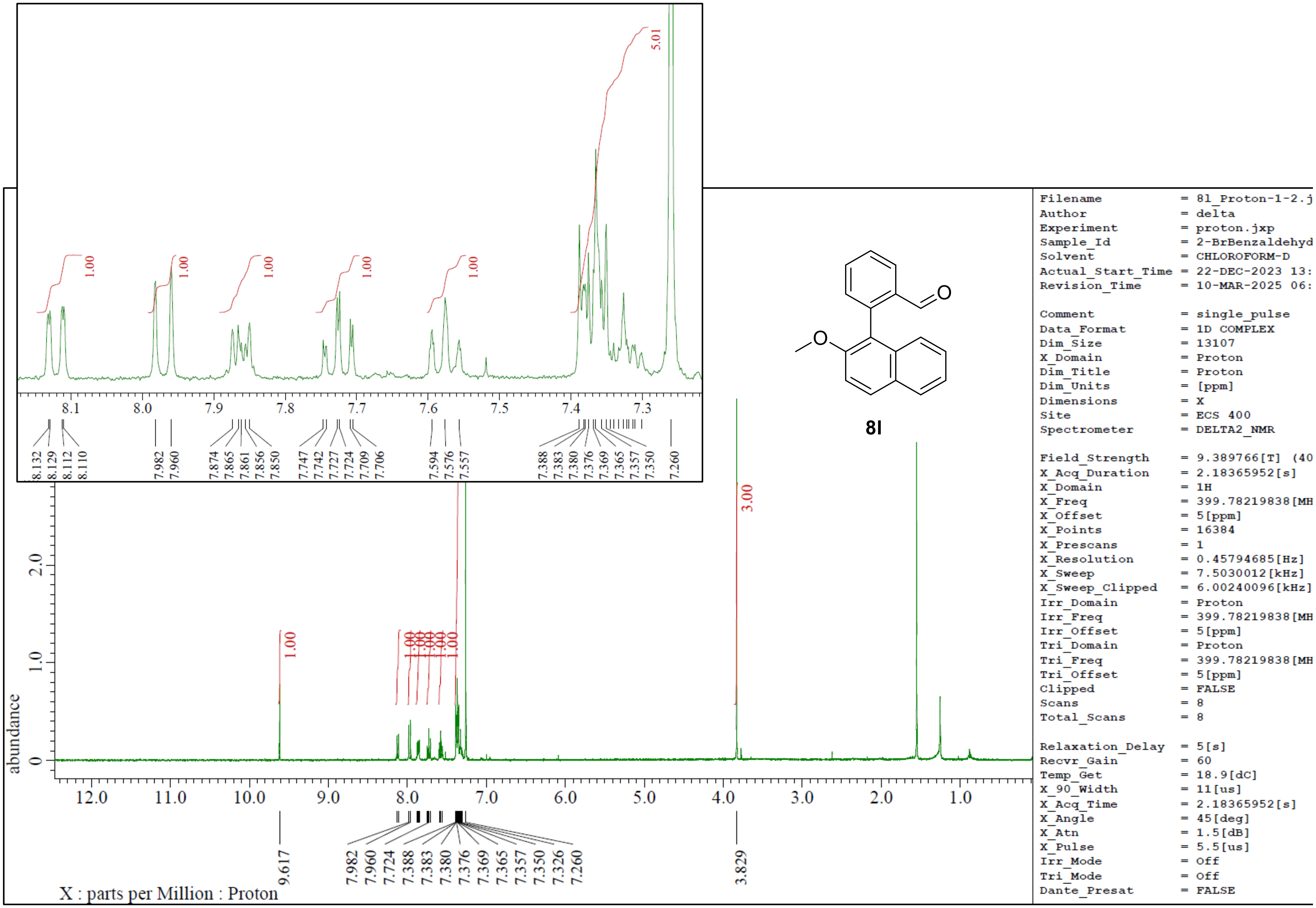

Compound **8l** (^{1}H NMR, 400 MHz, $CDCl_3$).

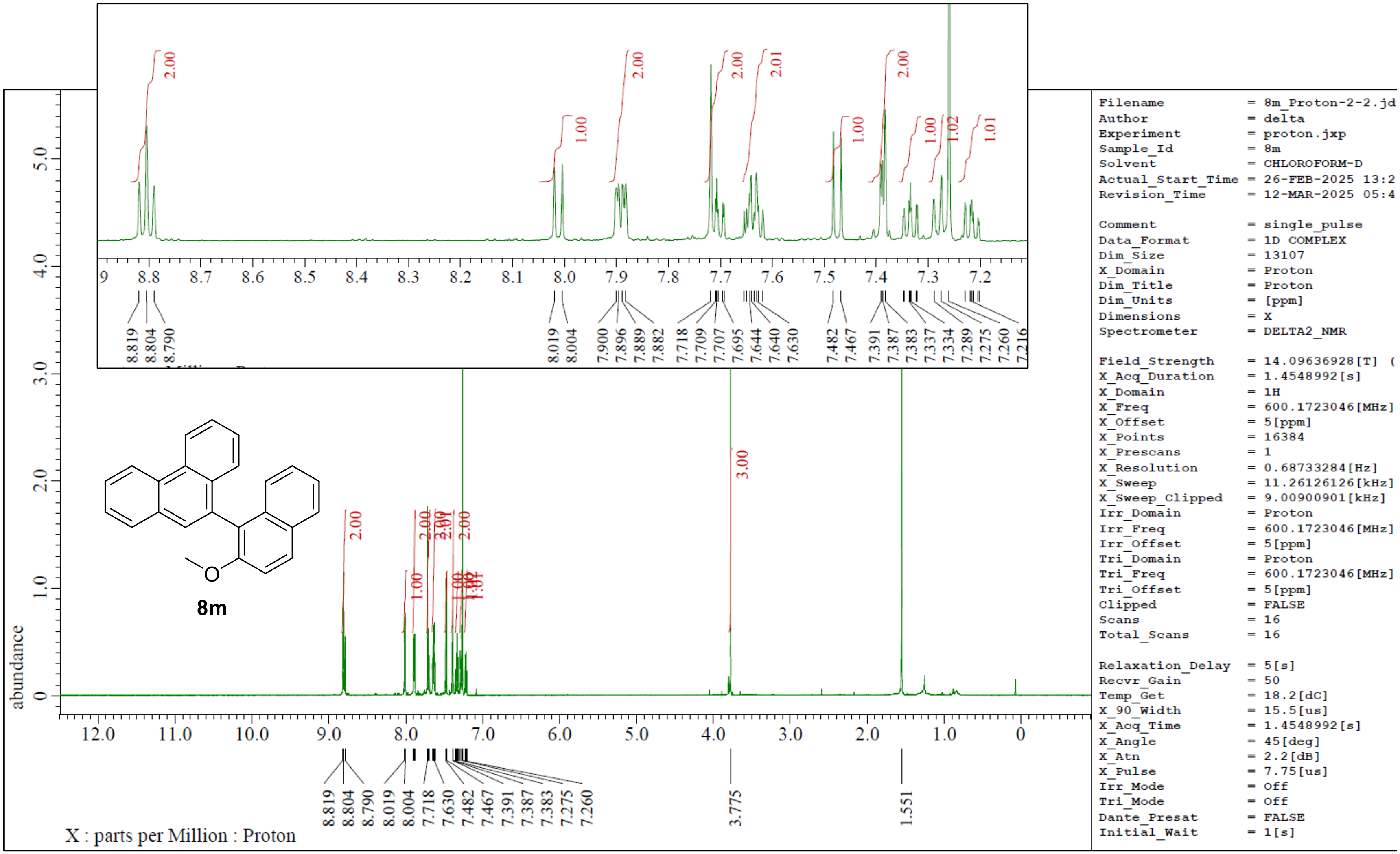

Compound **8m** (^{1}H NMR, 600 MHz, $CDCl_3$).

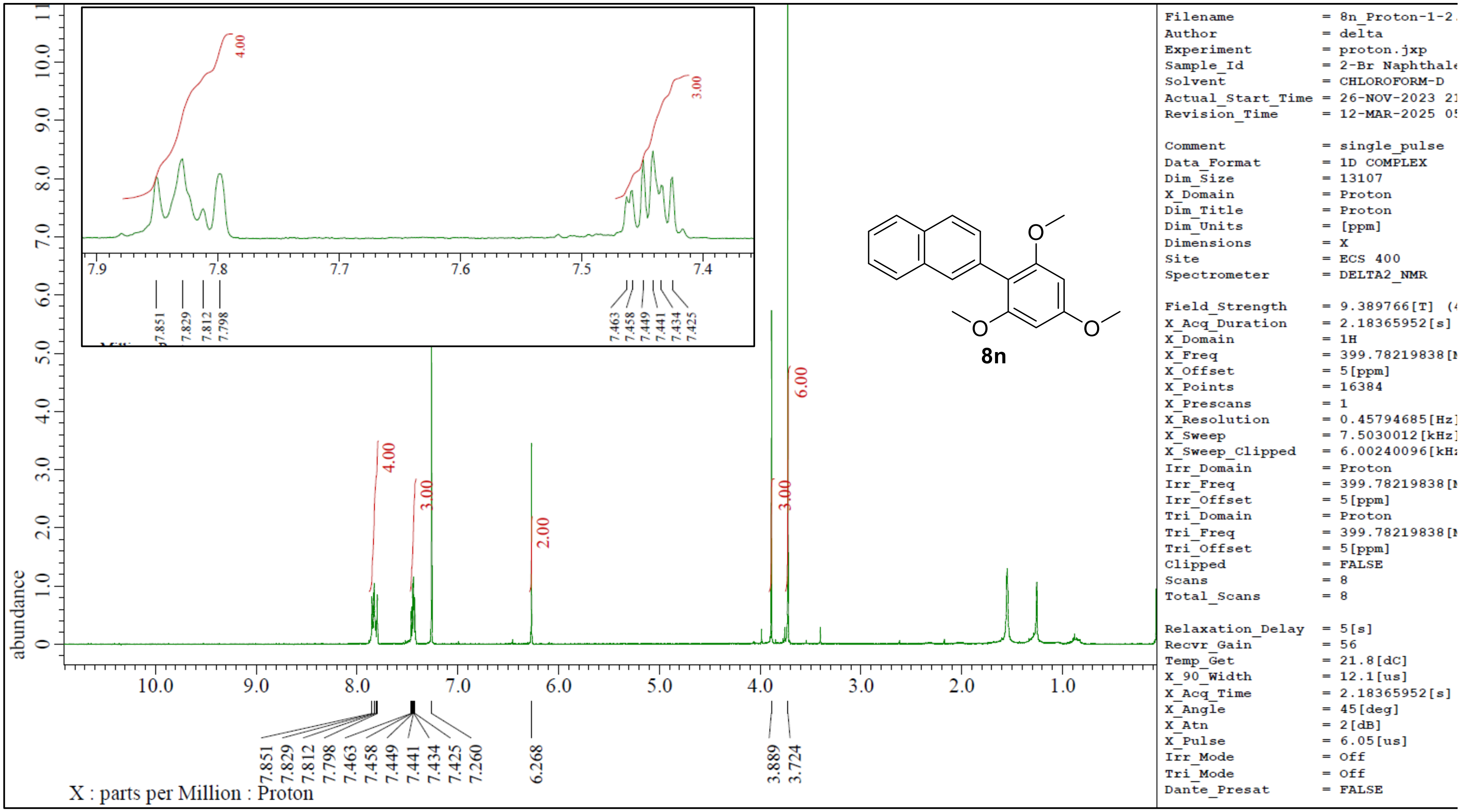

Compound **8n** (^{1}H NMR, 400 MHz, $CDCl_3$).

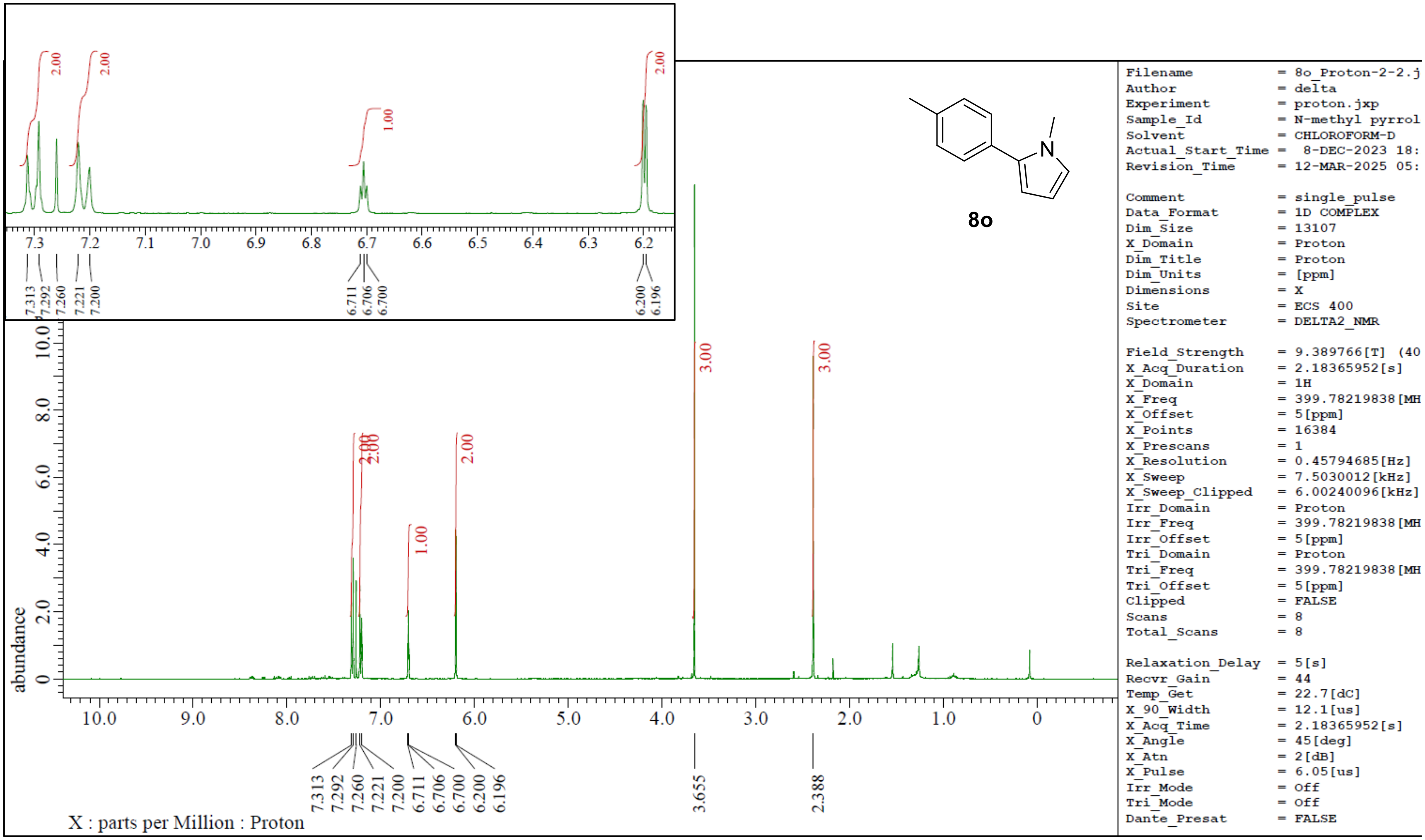

Compound **8o** (^{1}H NMR, 400 MHz, $CDCl_3$).

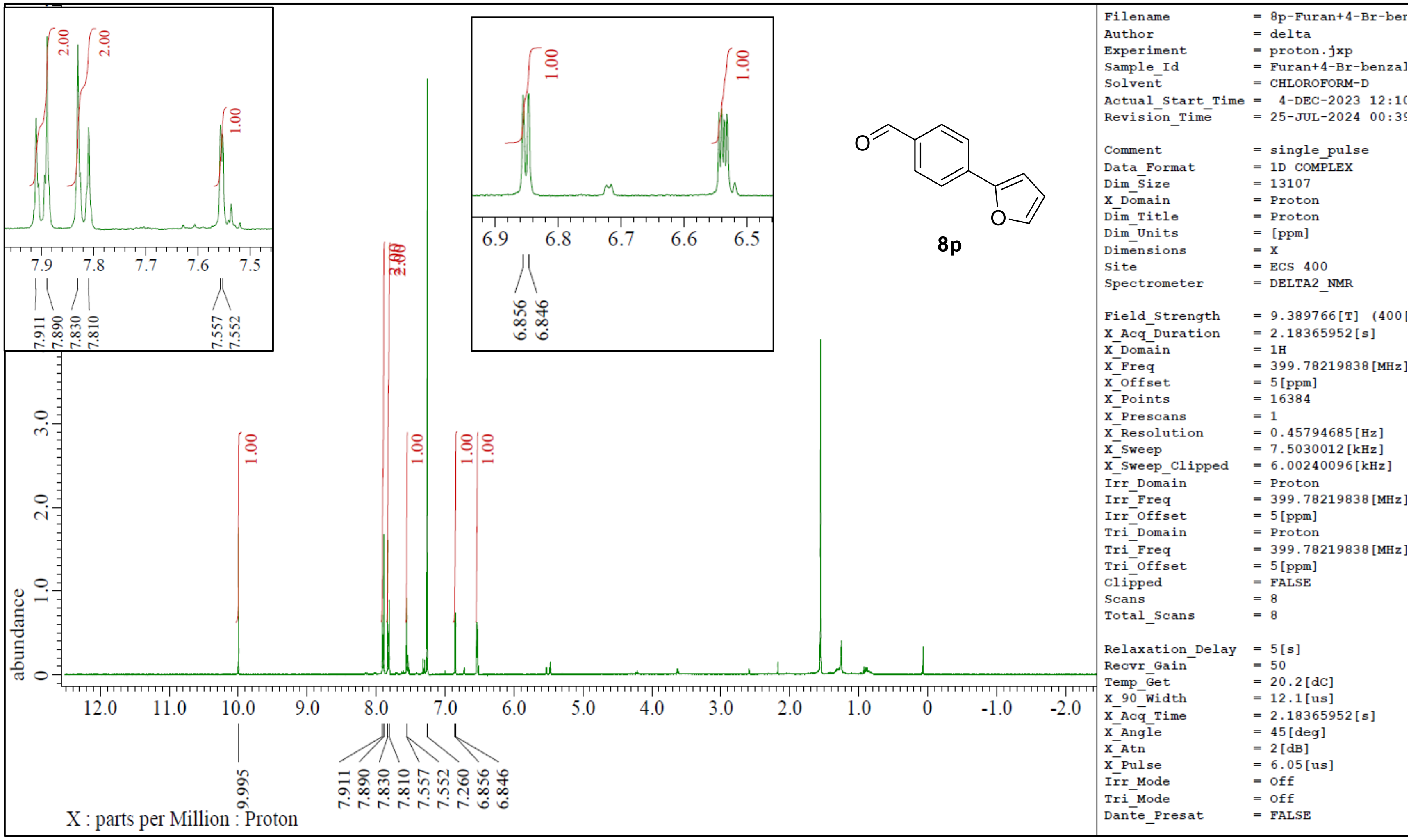

Compound **8p** (^{1}H NMR, 400 MHz, $CDCl_3$).

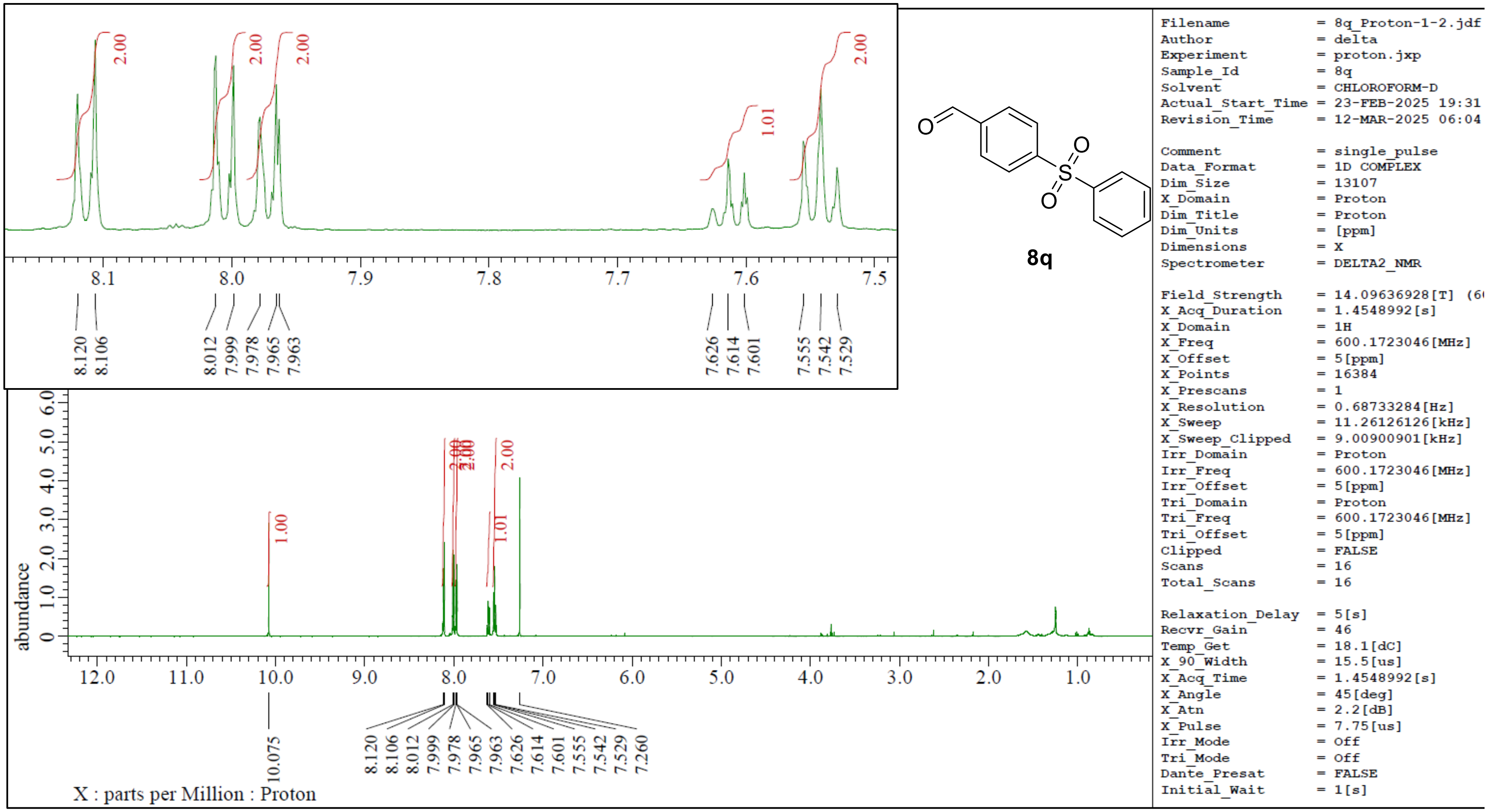

Compound **8q** (^{1}H NMR, 600 MHz, $CDCl_3$).

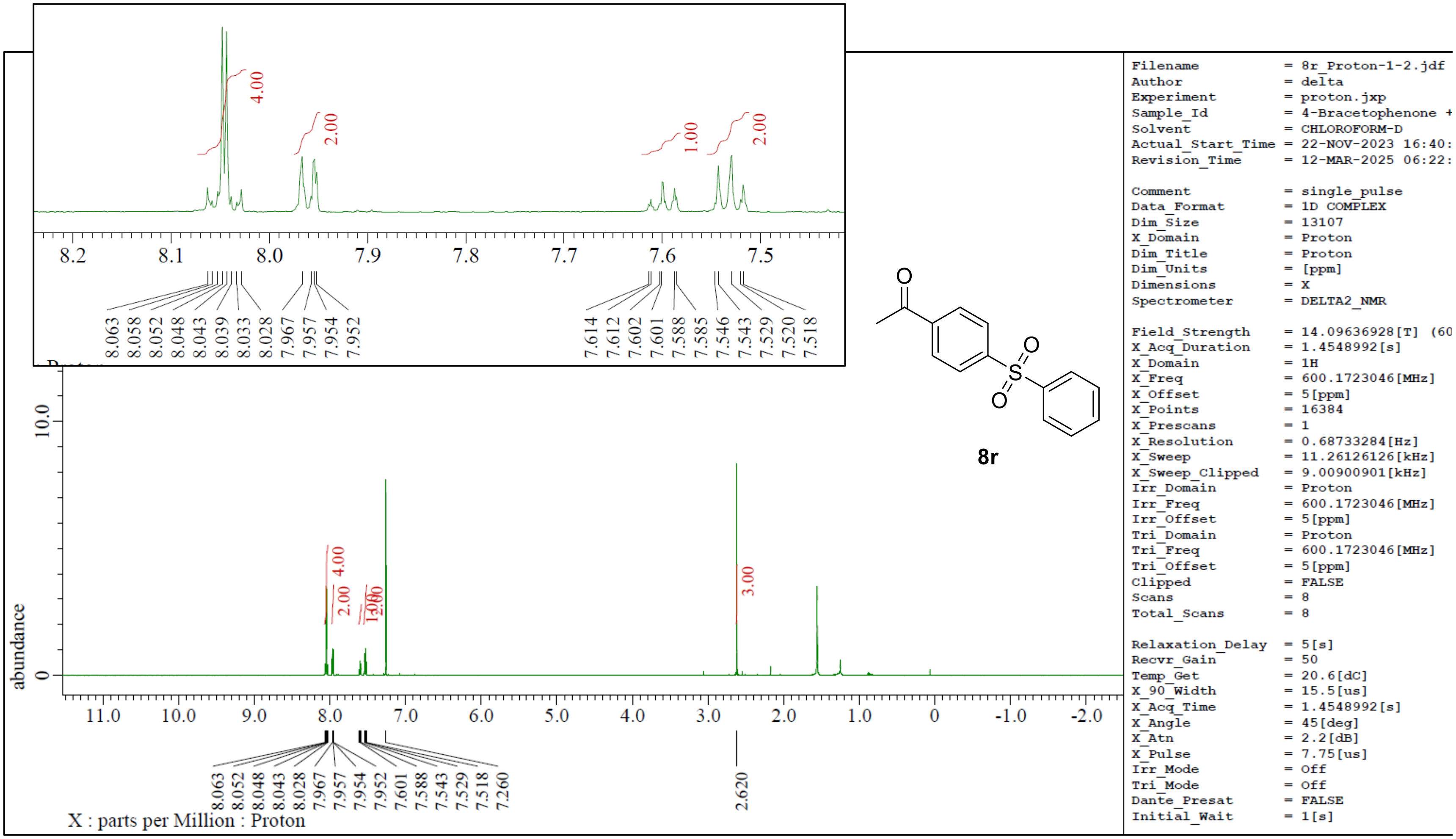

Compound **8r** (^{1}H NMR, 600 MHz, $CDCl_3$).

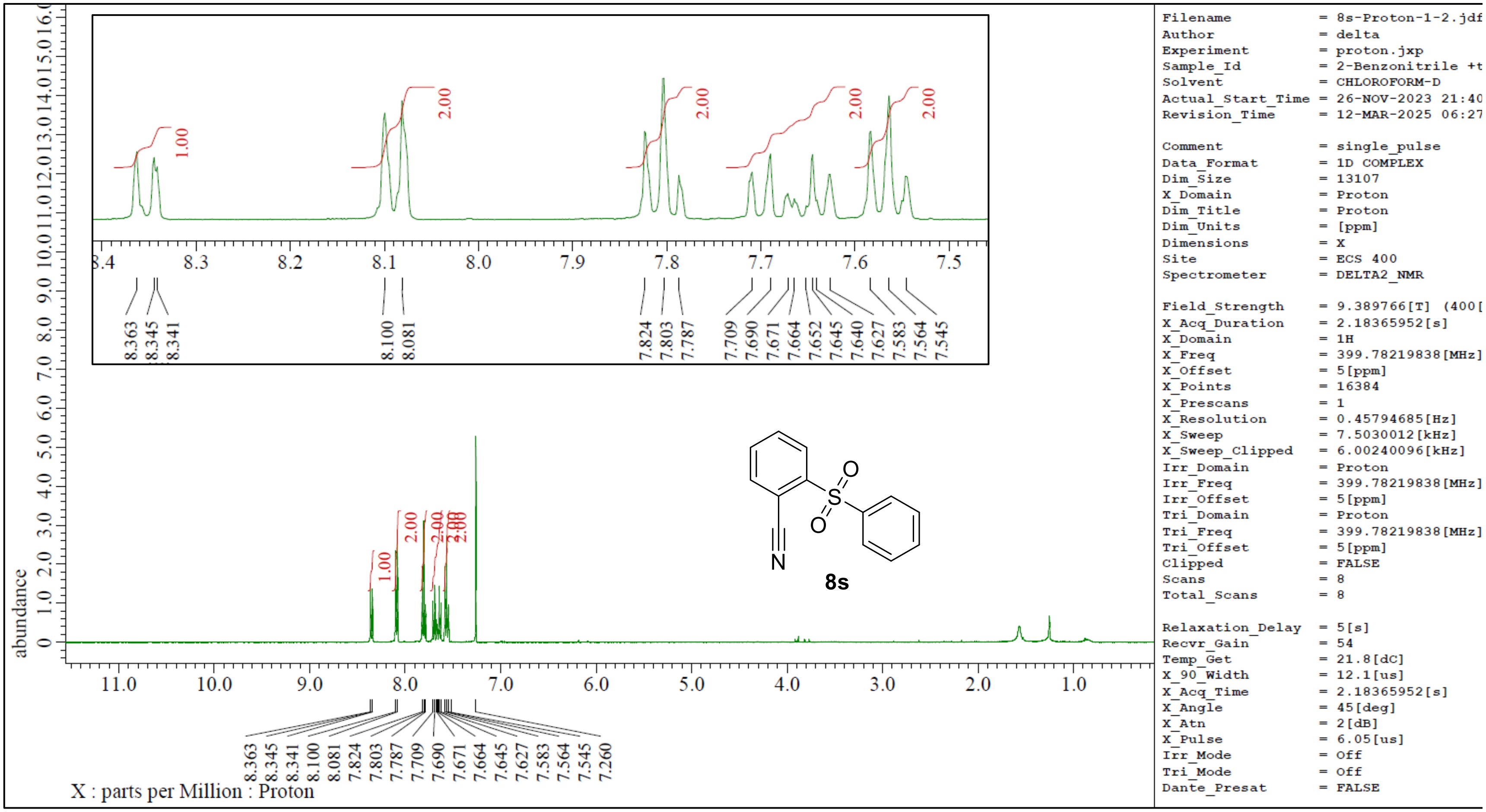

Compound **8s** (^{1}H NMR, 400 MHz, $CDCl_3$).

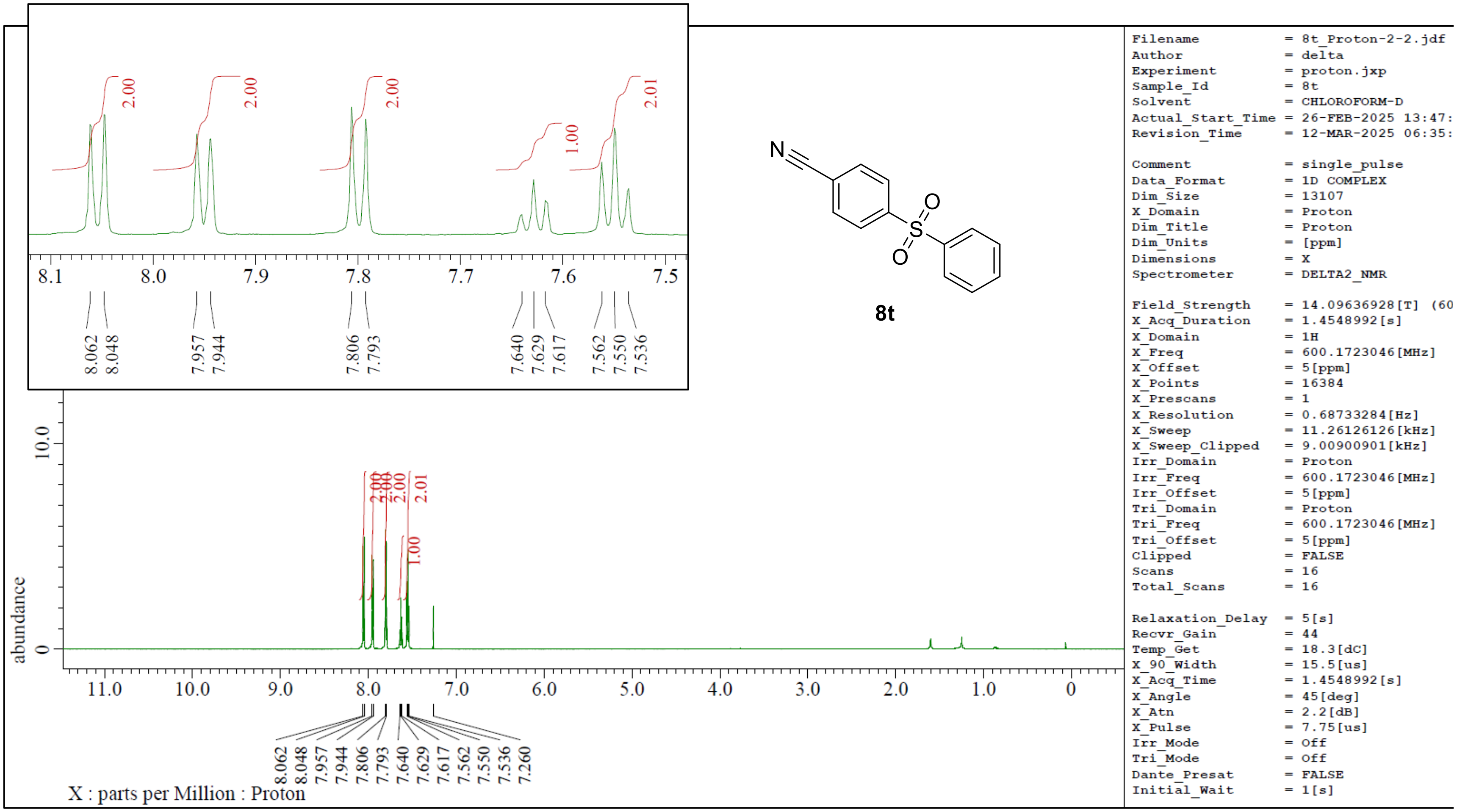

Compound **8t** (^{1}H NMR, 600 MHz, $CDCl_3$).

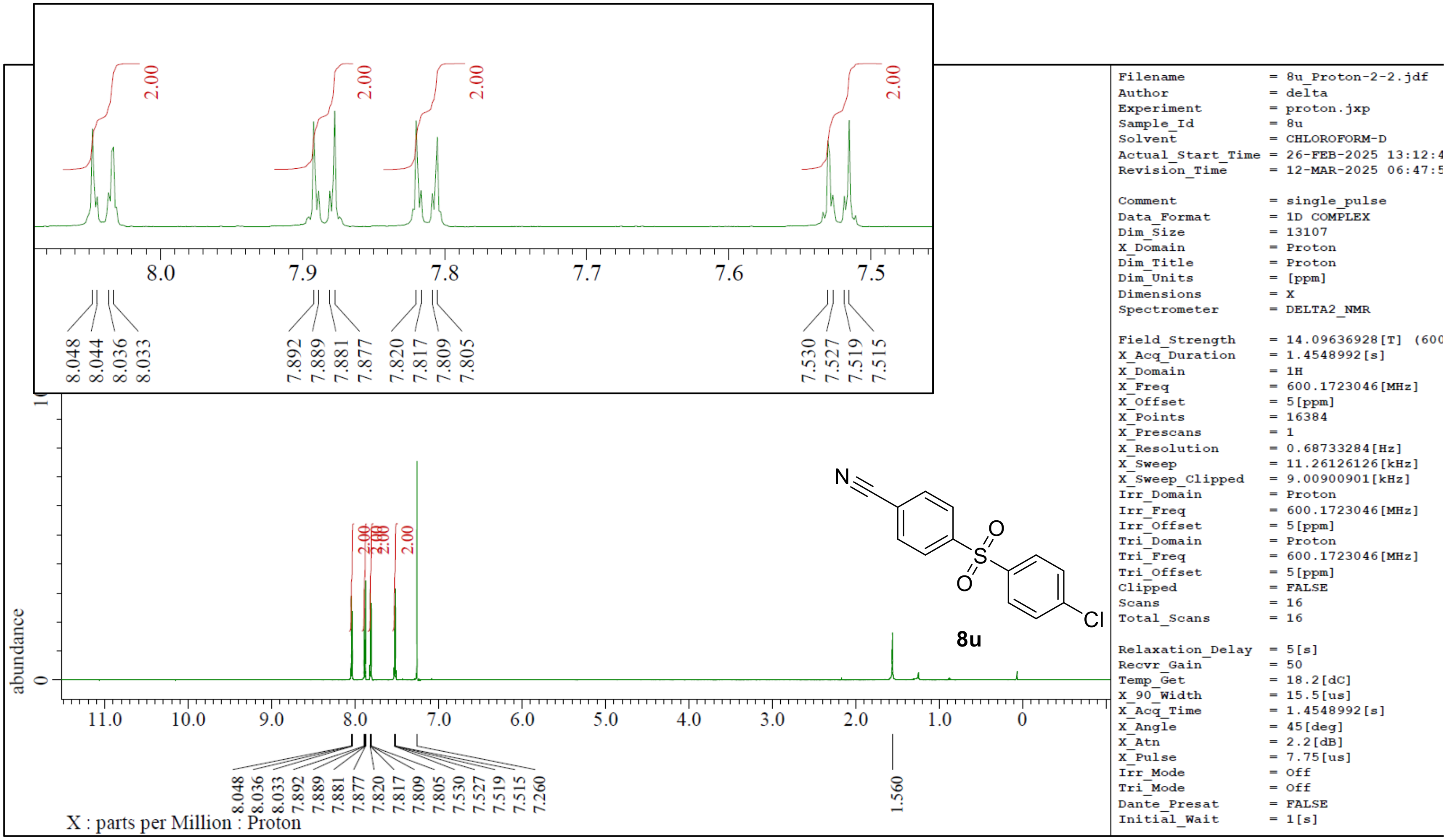

Compound **8u** (^{1}H NMR, 600 MHz, $CDCl_3$).

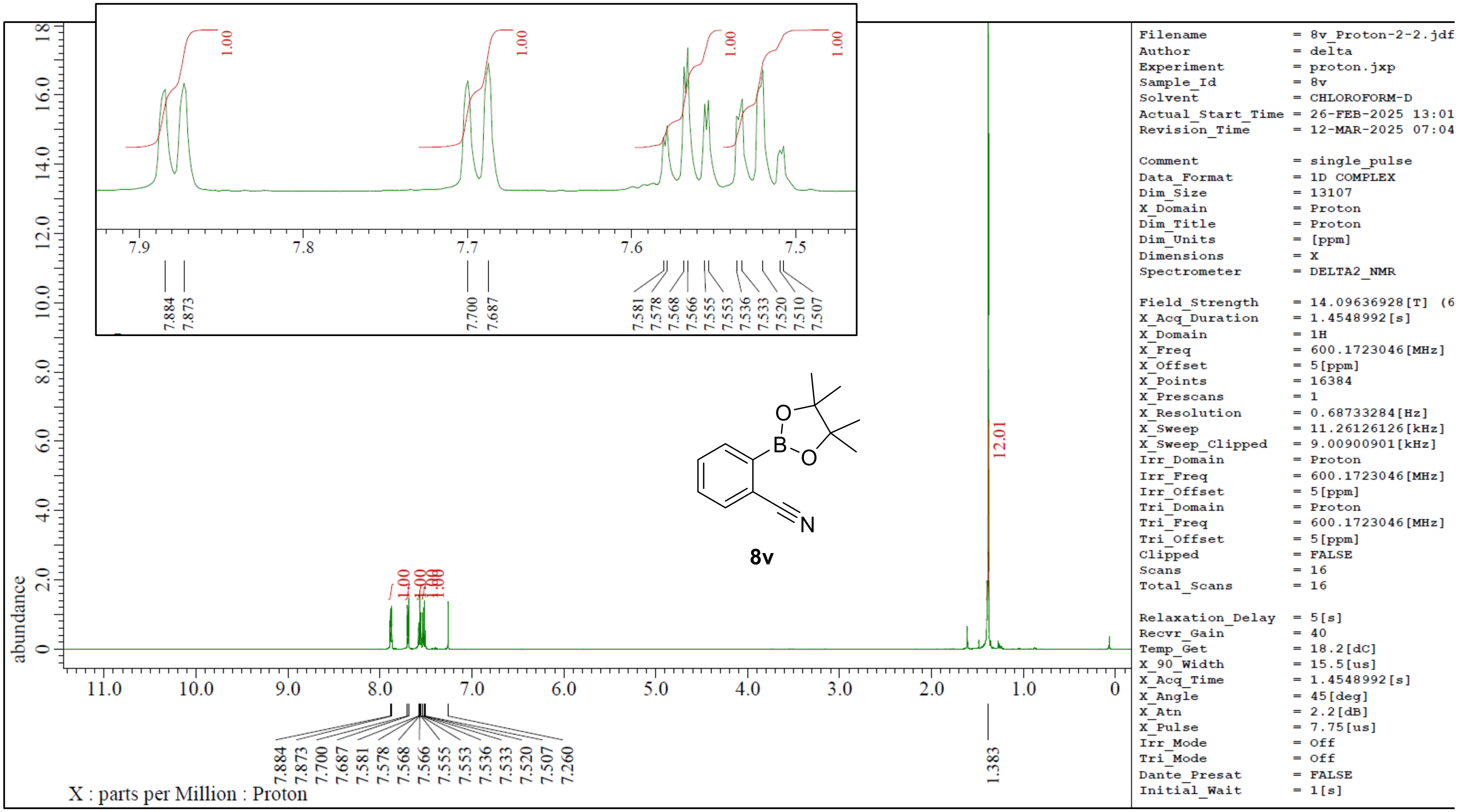

Compound **8v** (^{1}H NMR, 600 MHz, $CDCl_3$).

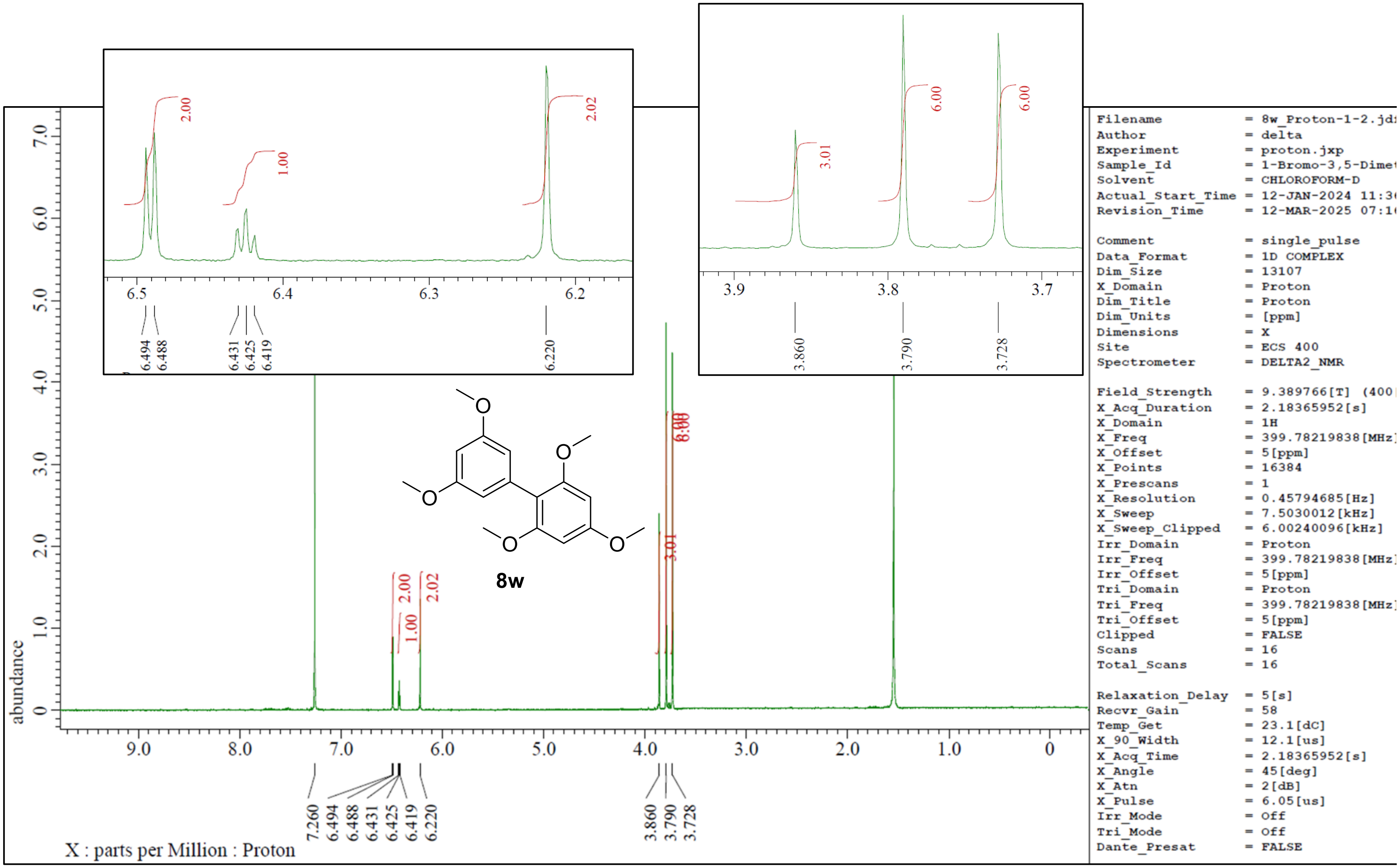

Compound **8w** (^{1}H NMR, 400 MHz, $CDCl_3$).

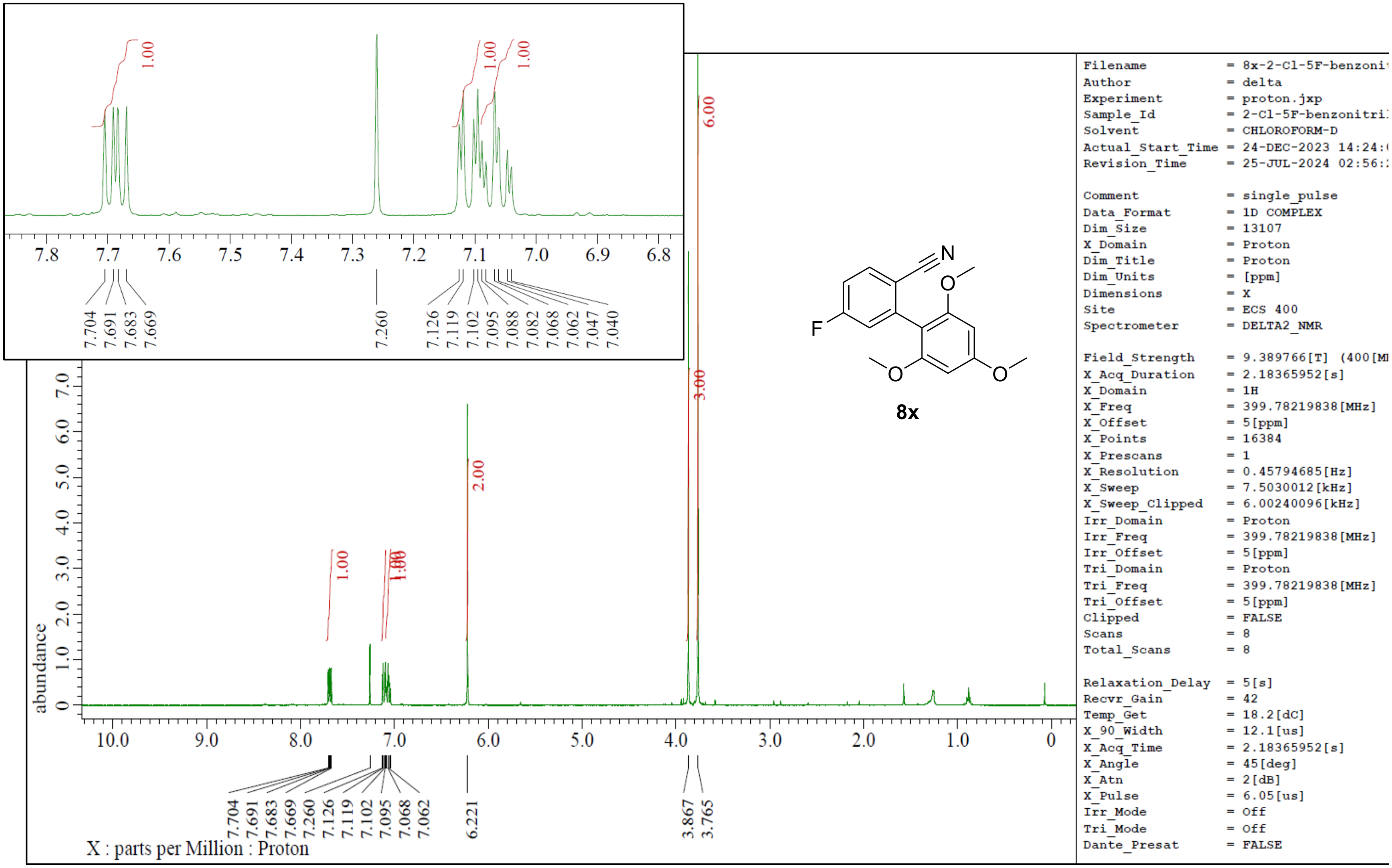

Compound **8x** (^1H NMR, 400 MHz, $CDCl_3$).

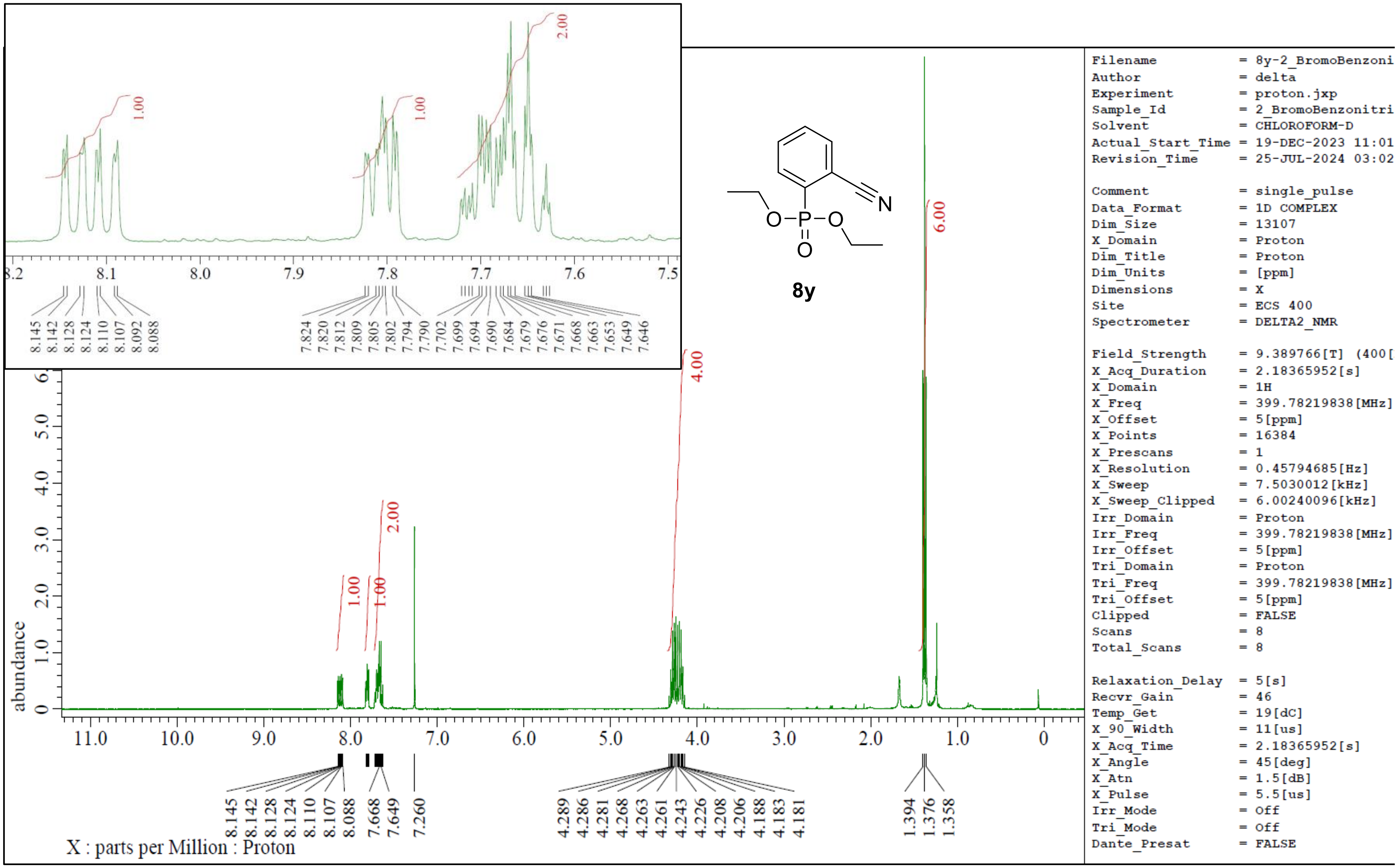

Compound **8y** (^{1}H NMR, 400 MHz, $CDCl_3$).

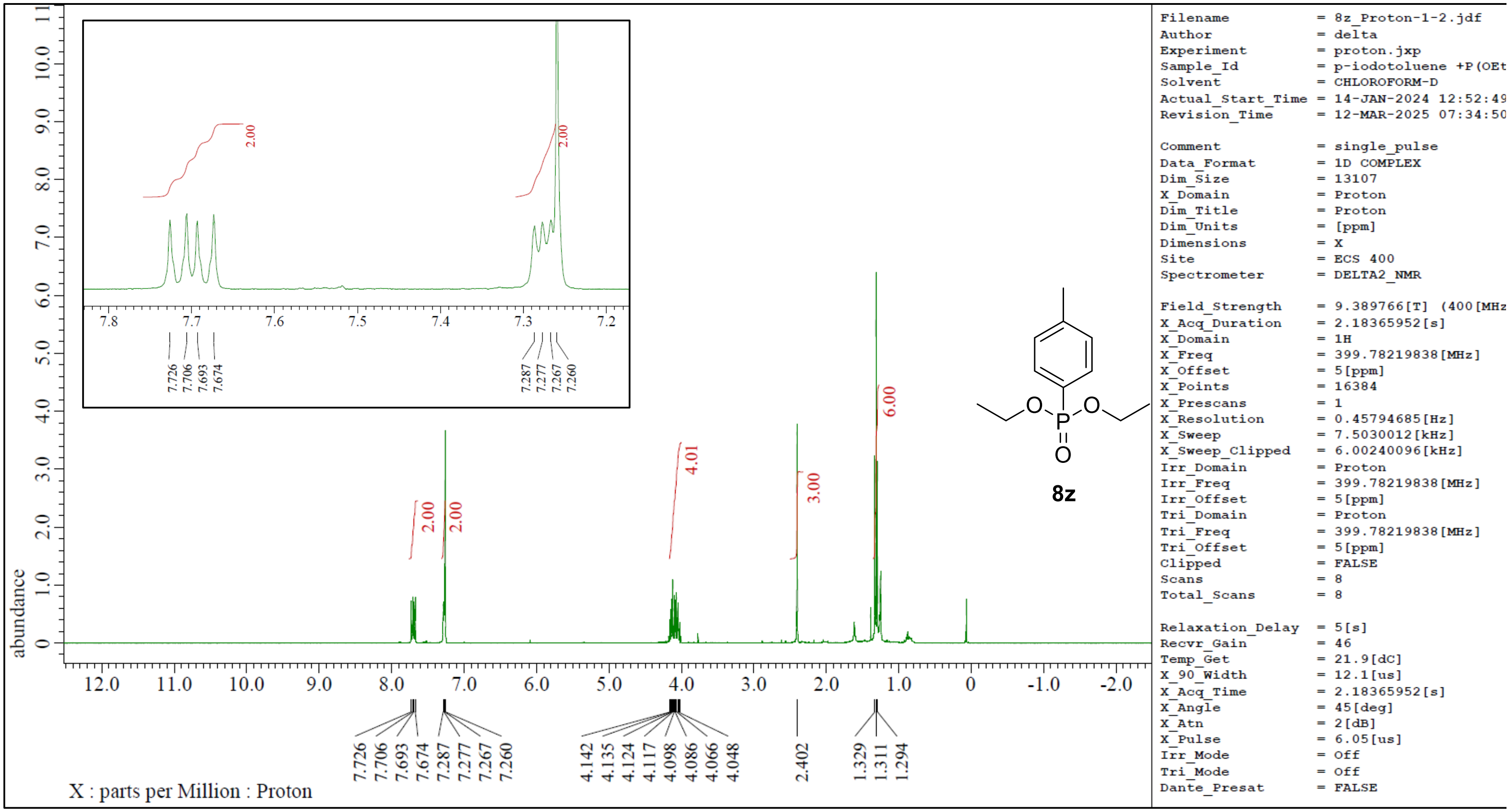

Compound **8z** (^{1}H NMR, 400 MHz, $CDCl_3$).

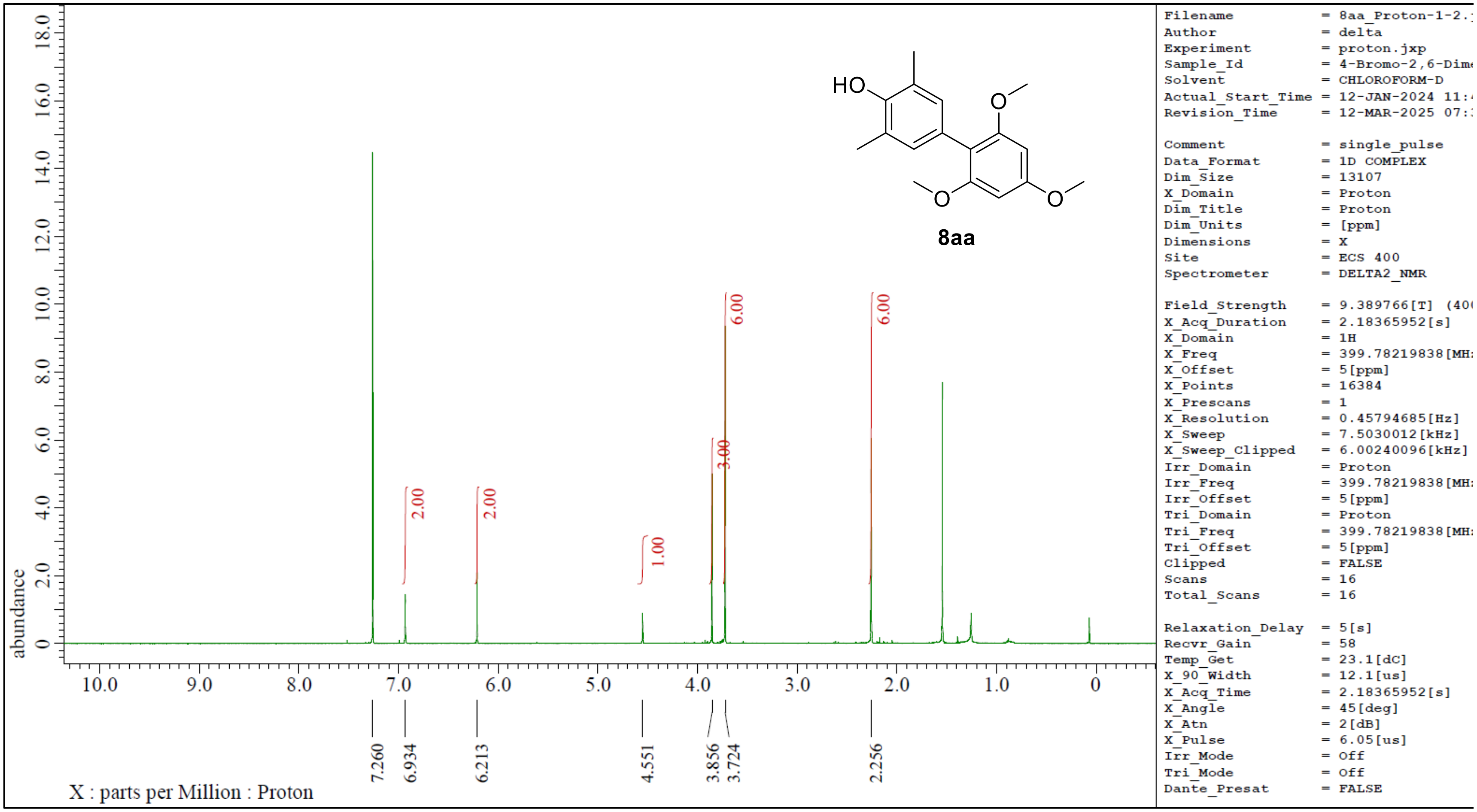

Compound **8aa** (^{1}H NMR, 400 MHz, $CDCl_3$).

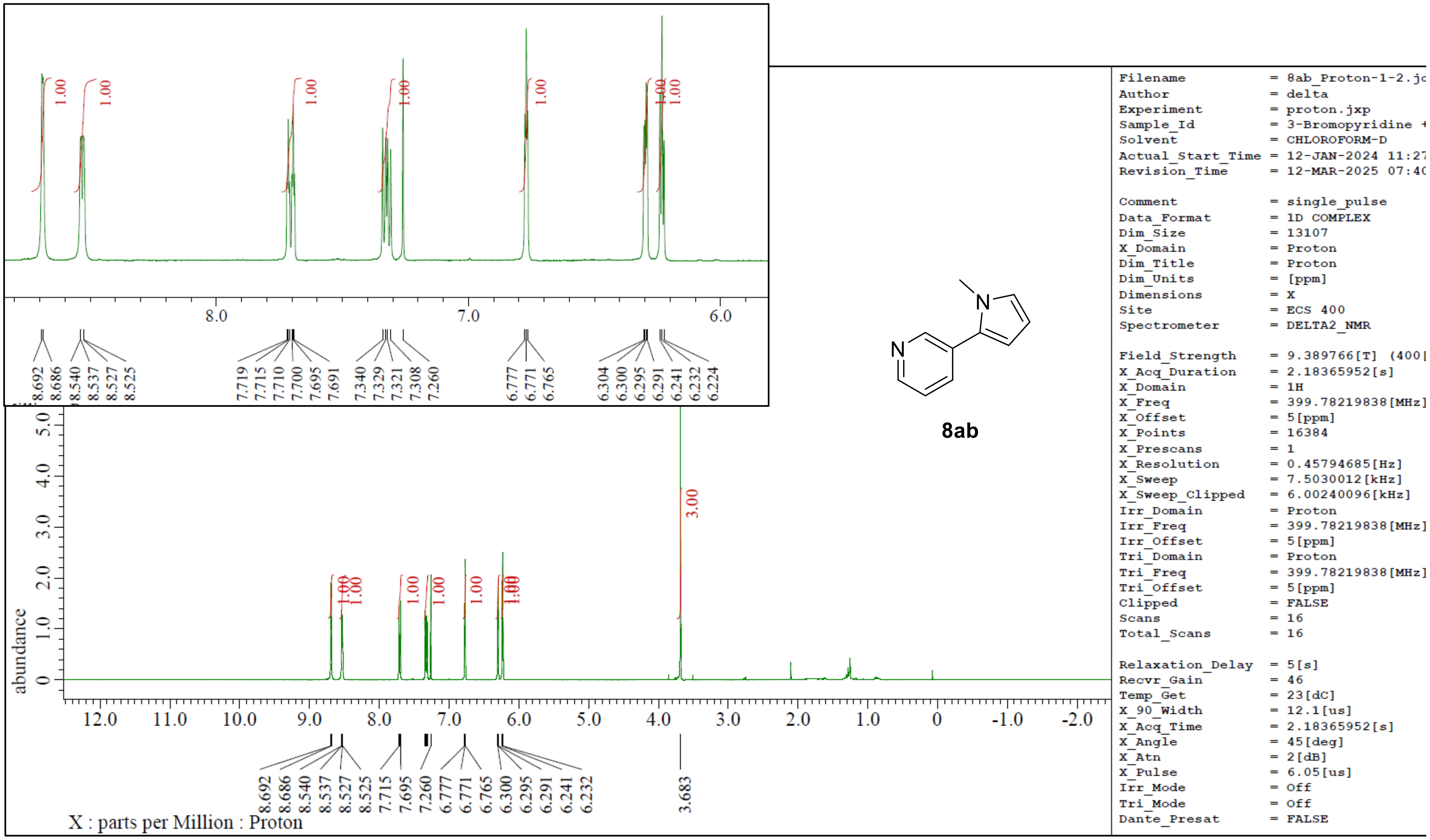

8ab

Filename	=	8ab_Proton-1-2.jc
Author	=	delta
Experiment	=	proton.jxp
Sample_Id	=	3-Bromopyridine +
Solvent	=	CHLOROFORM-D
Actual_Start_Time	=	12-JAN-2024 11:27
Revision_Time	=	12-MAR-2025 07:40
Comment	=	single_pulse
Data_Format	=	1D COMPLEX
Dim_Size	=	13107
X_Domain	=	Proton
Dim_Title	=	Proton
Dim_Units	=	[ppm]
Dimensions	=	X
Site	=	ECS 400
Spectrometer	=	DELTA2_NMR
Field_Strength	=	9.389766[T] (400[
X_Acq_Duration	=	2.18365952[s]
X_Domain	=	1H
X_Freq	=	399.78219838[MHz]
X_Offset	=	5[ppm]
X_Points	=	16384
X_Prescans	=	1
X_Resolution	=	0.45794685[Hz]
X_Sweep	=	7.5030012[kHz]
X_Sweep_Clipped	=	6.00240096[kHz]
Irr_Domain	=	Proton
Irr_Freq	=	399.78219838[MHz]
Irr_Offset	=	5[ppm]
Tri_Domain	=	Proton
Tri_Freq	=	399.78219838[MHz]
Tri_Offset	=	5[ppm]
Clipped	=	FALSE
Scans	=	16
Total_Scans	=	16
Relaxation_Delay	=	5[s]
Recvr_Gain	=	46
Temp_Get	=	23[dC]
X_90_Width	=	12.1[us]
X_Acq_Time	=	2.18365952[s]
X_Angle	=	45[deg]
X_Atn	=	2[dB]
X_Pulse	=	6.05[us]
Irr_Mode	=	Off
Tri_Mode	=	Off
Dante_Presat	=	FALSE

Compound **8ab** (^{1}H NMR, 400 MHz, $CDCl_3$).

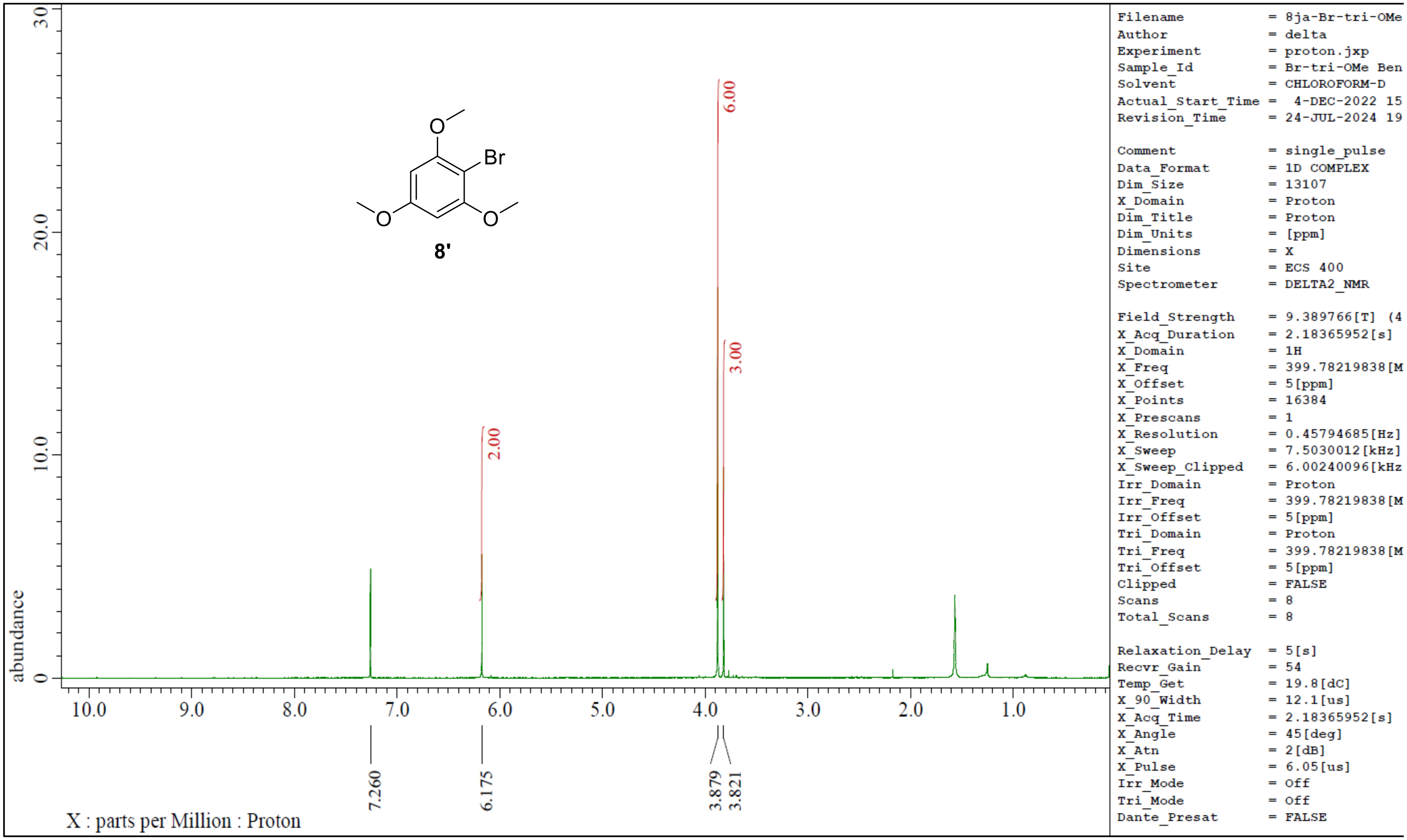

Compound **8'** (^{1}H NMR, 400 MHz, $CDCl_3$).

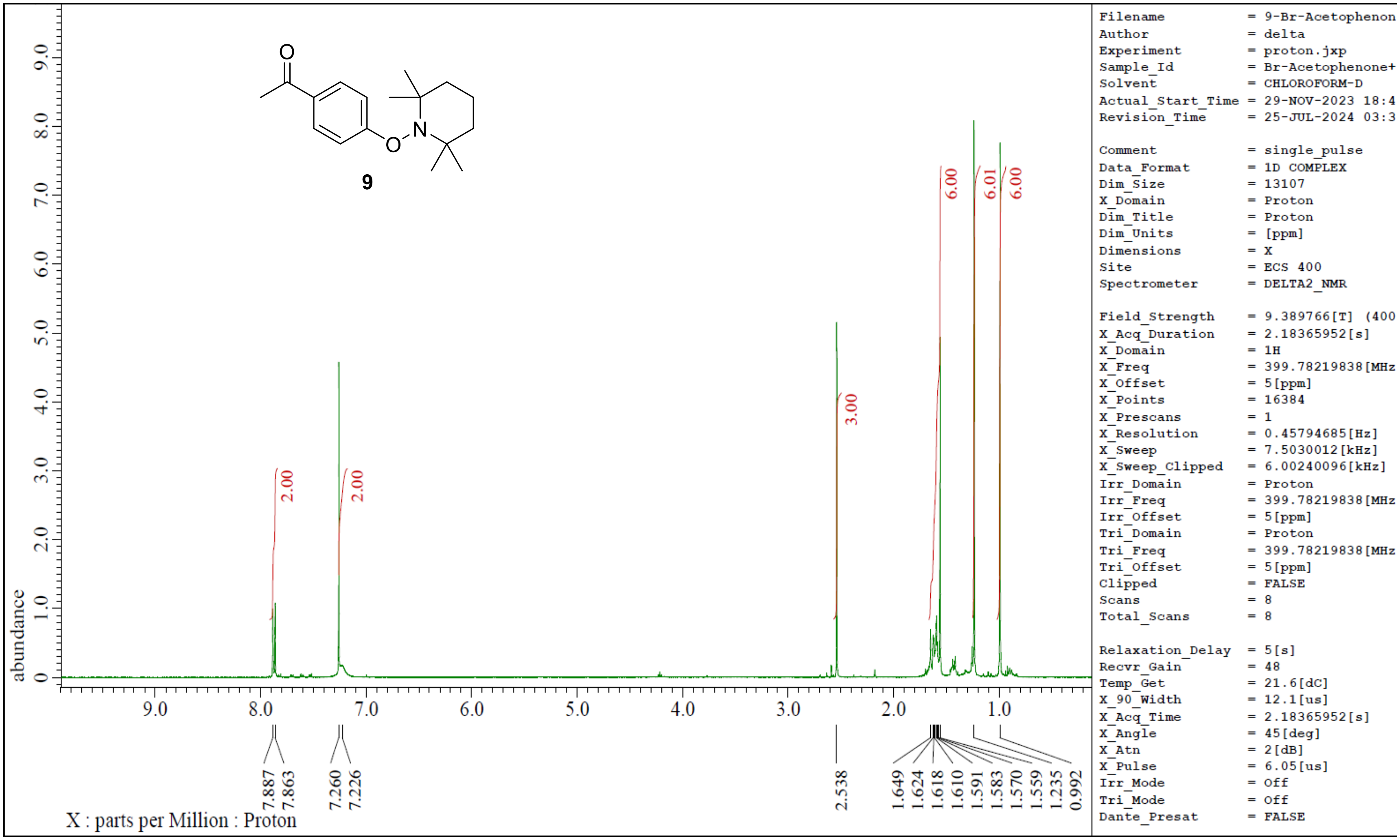

Compound **9** (^{1}H NMR, 400 MHz, $CDCl_3$).

20. Supplementary References

1 Hacker, A. S. *et al.* Synthesis and electronic properties of fluoreno [2,1-*a*] fluorenedione and fluoreno [1,2-*a*] fluorenedione. *J. Org. Chem.* **83**, 510–515 (2018).

2 Li, G., Liu, Y. & Du, H. B (C_6F_5)$_3$-catalyzed metal-free hydrogenation of naphthylamines. *Org. Biomol. Chem.* **13**, 2875–2878 (2015).

3 Khalid, M. I. *et al.* Electrochemical synthesis of heterodehydro[7]helicenes. *Commun. Chem.* **5**, 166 (2022).

4 Rajabi, F. & Thiel, W. R. An efficient palladium *N*-heterocyclic carbene catalyst allowing the Suzuki-Miyaura cross-coupling of aryl chlorides and arylboronic acids at room temperature in aqueous solution. *Adv. Synth. Catal.* **356**, 1873–1877 (2014).

5 Zhang, J. *et al.* An air-stable, well-defined palladium–BIAN–NHC chloro dimer: a fast-activating, highly efficient catalyst for cross-coupling. *Chem. Commun.* **58**, 7404–7407 (2022).

6 Pezzetta, C. *et al. peri*-Xanthenoxanthene (PXX): a versatile organic photocatalyst in organic synthesis. *Adv. Synth. Catal.* **363**, 4740–4753 (2021).

7 Yuan, T., Zheng, M., Antonietti, M. & Wang, X. Ceramic boron carbonitrides for unlocking organic halides with visible light. *Chem. Sci.* **12**, 6323–6332 (2021).

8 Crespi, S., Protti, S. & Fagnoni, M. Wavelength selective generation of aryl radicals and aryl cations for metal-free photoarylations. *J. Org. Chem.* **81**, 9612–9619 (2016).

9 Zhou, Y., Deng, S., Mai, S. & Song, Q. Cu-catalyzed denitrogenative ring-opening of 3-aminoindazoles for the synthesis of aromatic nitrile-containing (hetero) arenes. *Org. Lett.* **20**, 6161–6165 (2018).

10 Mao, S., Shi, X., Soulé, J. F. & Doucet, H. Exploring green solvents associated to Pd/C as heterogeneous catalyst for direct arylation of heteroaromatics with aryl bromides. *Adv. Synth. Catal.* **360**, 3306–3317 (2018).

11 Wanner, D. M. *et al.* Cooperative Lewis acid-1, 2, 3-triazolium-aryloxide catalysis: pyrazolone addition to nitroolefins as entry to diaminoamides. *Angew. Chem. Int. Ed.* **62**, e202307317 (2023).

12 Pankhade, Y. A., Pandey, R., Fatma, S., Ahmad, F. & Anand, R. V. TfOH-catalyzed intramolecular annulation of 2-(aryl)-phenyl-substituted *p*-quinone methides under continuous flow: total syntheses of selaginpulvilin I and isoselagintamarlin A. *J. Org. Chem.* **87**, 3363–3377 (2022).

13 Mohammad-Pour, G. S. *et al.* Modular design of fluorescent dibenzo-and naphtho-fluoranthenes: structural rearrangements and electronic properties. *J. Org. Chem.* **83**, 8036–8053 (2018).

14 Ban, I., Sudo, T., Taniguchi, T. & Itami, K. Copper-mediated C– H bond arylation of arenes with arylboronic acids. *Org. Lett.* **10**, 3607–3609 (2008).

15 Heijnen, D., Helbert, H., Luurtsema, G., Elsinga, P. H. & Feringa, B. L. Synthesis of substituted benzaldehydes *via* a two-step, one-pot reduction/cross-coupling procedure. *Org. Lett.* **21**, 4087–4091 (2019).

16 Liu, N.-W., Hofman, K., Herbert, A. & Manolikakes, G. Visible-light photoredox/nickel dual catalysis for the cross-coupling of sulfinic acid salts with aryl iodides. *Org. Lett.* **20**, 760–763 (2018).

17 Wang, S.-D., Yang, B., Zhang, H., Qu, J.-P. & Kang, Y.-B. Reductive cleavage of C–X or N–S bonds catalyzed by super organoreductant CBZ6. *Org. Lett.* **25**, 816–820 (2023).

18 Chen, Y.-J. *et al.* Transition-metal-free, site-selective C–F arylation of polyfluoroarenes *via* electrophotocatalysis. *J. Am. Chem. Soc.* **144**, 17261–17268 (2022).

19 Bai, Y. *et al.* Nickel-catalyzed electrochemical phosphorylation of aryl bromides. *Org. Lett.* **21**, 6835–6838 (2019).
20 Dyadyuk, A. *et al.* Direct synthesis of polyaryls by consecutive oxidative cross-coupling of phenols with arenes. *Org. Lett.* **18**, 4324–4327 (2016).
21 Shen, N. *et al.* Photocatalytic cross-couplings of aryl halides enabled by *o*-phosphinophenolate and *o*-phosphinothiophenolate. *ACS Catal.* **12**, 2788–2795 (2022).
22 Mondal, M., Puranik, V. G. & Argade, N. P. Facile synthesis of 1,3,7-trihydroxyxanthone and its regioselective coupling reactions with prenal: Simple and efficient access to osajaxanthone and nigrolineaxanthone F. *J. Org. Chem.* **71**, 4992–4995 (2006).
23 Abrams, J. Alternative method for the synthesis of triazenes from aryl diazonium salts. *Tetrahedron* **89**, 132185 (2021).
24 Becke, A. D. Density-functional thermochemistry. III. The role of exact exchange. *J. Chem. Phys.* **98**, 5648–5652 (1993).
25 Salem, M. S. H. *et al.* Data-driven electrochemical one-pot synthesis of double hetero [7] dehydrohelicene. *Electrochemistry* **91**, 112015–112015 (2023).
26 Salem, M. S. H. *et al.* Impact of helical elongation of symmetric oxa[*n*]helicenes on their structural, photophysical, and chiroptical characteristics. *Chirality* **36**, e23673 (2024).
27 Grimme, S., Antony, J., Ehrlich, S. & Krieg, H. A consistent and accurate ab initio parametrization of density functional dispersion correction (DFT-D) for the 94 elements H-Pu. *J. Chem. Phys.* **132**, 154104 (2010).
28 Maeda, S., Harabuchi, Y., Ono, Y., Taketsugu, T. & Morokuma, K. Intrinsic reaction coordinate: Calculation, bifurcation, and automated search. *Int. J. Quantum Chem* **115**, 258–269 (2015).
29 Hashemi, A., Peljo, P. & Laasonen, K. Understanding electron transfer reactions using constrained density functional theory: complications due to surface interactions. *J. Phys. Chem. C* **127**, 3398–3407 (2023).
30 van der Zee, L. J., Hofman, J., van Gaalen, J. M. & Slootweg, J. C. Mechanistic studies on single-electron transfer in frustrated Lewis pairs and its application to main-group chemistry. *Chem. Soc. Rev.* (2024).
31 Rempala, P., Kroulík, J. & King, B. T. A slippery slope: mechanistic analysis of the intramolecular Scholl reaction of hexaphenylbenzene. *J. Am. Chem. Soc.* **126**, 15002–15003 (2004).
32 Rempala, P., Kroulík, J. & King, B. T. Investigation of the mechanism of the intramolecular Scholl reaction of contiguous phenylbenzenes. *J. Org. Chem.* **71**, 5067–5081 (2006).
33 Domingo, L. R., Perez-Ruiz, R., Argüello, J. E. & Miranda, M. A. DFT study on the cycloreversion of thietane radical cations. *J. Phys. Chem. A* **115**, 5443–5448 (2011).
34 Riehl, B., Dyballa, K. M., Franke, R. & Waldvogel, S. R. Electro-organic synthesis as a sustainable alternative for dehydrogenative cross-coupling of phenols and naphthols. *Synthesis* **49**, 252–259 (2017).
35 Selt, M., Mentizi, S., Schollmeyer, D., Franke, R. & Waldvogel, S. R. Selective and scalable dehydrogenative electrochemical synthesis of 3, 3, 5, 5-Tetramethyl-2, 2-biphenol. *Synlett* **30**, 2062–2067 (2019).
36 Röckl, J. L., Pollok, D., Franke, R. & Waldvogel, S. R. A decade of electrochemical dehydrogenative C, C-coupling of aryls. *Acc. Chem. Res.* **53**, 45–61 (2019).
37 Dahms, B. *et al.* Selective Formation of 4, 4'-Biphenols by Anodic Dehydrogenative Cross- and Homo-Coupling Reaction. *Chem. Eur. J.* **25**, 2713–2716 (2019).
38 Elsler, B., Schollmeyer, D., Dyballa, K. M., Franke, R. & Waldvogel, S. R. Metal- and reagent-free highly selective anodic cross-coupling reaction of phenols. *Angew. Chem. Int. Ed.* **53**, 5210–5213 (2014).

39 Röckl, J. L., Schollmeyer, D., Franke, R. & Waldvogel, S. R. Dehydrogenative Anodic C− C Coupling of Phenols Bearing Electron ‐ Withdrawing Groups. *Angew. Chem. Int. Ed.* **59**, 315–319 (2020).
40 Kirste, A., Schnakenburg, G., Stecker, F., Fischer, A. & Waldvogel, S. R. Anodic Phenol‐Arene Cross-Coupling Reaction on Boron-Doped Diamond Electrodes. *Angew. Chem. Int. Ed.* **49**, 971–975 (2010).
41 Dutra, F. R., Silva, C. d. S. & Custodio, R. On the accuracy of the direct method to calculate pKa from electronic structure calculations. *J. Phys. Chem. A* **125**, 65–73 (2020).
42 Zou, Y. *et al.* Scholl Reaction of Perylene-Based Polyphenylene Precursors under Different Conditions: Formation of Hexagon or Octagon? *Angew. Chem. Int. Ed.* **60**, 17654–17663 (2021).
43 Zhang, Y., Pun, S. H. & Miao, Q. The Scholl reaction as a powerful tool for synthesis of curved polycyclic aromatics. *Chem. Rev.* **122**, 14554–14593 (2022).
44 Okada, Y. Redox-neutral radical-cation reactions: Multiple carbon–carbon bond formations enabled by single-electron transfer. *Electrochemistry* **88**, 497–506 (2020).
45 Roth, H. G., Romero, N. A. & Nicewicz, D. A. Experimental and calculated electrochemical potentials of common organic molecules for applications to single-electron redox chemistry. *Synlett* **27**, 714-723 (2016).
46 Marcus, R. A. On the theory of oxidation-reduction reactions involving electron transfer. I. *J. Chem. Phys.* **24**, 966–978 (1956).
47 Piechota, E. J. & Meyer, G. J. Introduction to electron transfer: Theoretical foundations and pedagogical examples. *J. Chem. Edu.* **96**, 2450–2466 (2019).
48 Buda, M. On calculating reorganization energies for electrochemical reactions using density functional theory and continuum solvation models. *Electrochim. Acta.* **113**, 536–549 (2013).
49 Trasatti, S. The absolute electrode potential: an explanatory note (Recommendations 1986). *Pure App. Chem.* **58**, 955–966 (1986).
50 Zhao, J. *et al.* An overview of Cu-based heterogeneous electrocatalysts for CO_2 reduction. *J. Mater. Chem. A* **8**, 4700–4734 (2020).
51 Salem, M. S. H. *et al.* Electrochemical synthesis of hetero[7]helicenes containing pyrrole and furan rings *via* an oxidative heterocoupling and dehydrative cyclization sequence. *Adv. Synth. Catal.* **365**, 373–380 (2023).
52 Rafiee, M., Mayer, M. N., Punchihewa, B. T. & Mumau, M. R. Constant potential and constant current electrolysis: an introduction and comparison of different techniques for organic electrosynthesis. *J. Org. Chem.* **86**, 15866–15874 (2021).
53 Alabugin, I. V., Eckhardt, P., Christopher, K. M. & Opatz, T. The Photoredox Paradox: Electron and Hole Upconversion as the Hidden Secrets of Photoredox Catalysis. *J. Am. Chem. Soc.* **146**, 27233–27254 (2024).
54 Julliard, M. & Chanon, M. Photoelectron-transfer catalysis: its connections with thermal and electrochemical analogs. *Chem. Rev.* **83**, 425–506 (1983).
55 Cismesia, M. A. & Yoon, T. P. Characterizing chain processes in visible light photoredox catalysis. *Chem. Sci.* **6**, 5426–5434 (2015).
56 Frisch, M. *et al.* Gaussian16; Gaussian, Inc. *Wallingford, CT* (2016).
57 Frisch, M. *et al.* Gaussian 09, revision D.1. Gaussian, Inc. *Wallingford, CT* (2009).
58 Haoyu, S. Y., He, X., Li, S. L. & Truhlar, D. G. MN15: A Kohn–Sham global-hybrid exchange–correlation density functional with broad accuracy for multi-reference and single-reference systems and noncovalent interactions. *Chem. Sci.* **7**, 5032–5051 (2016).
59 Salem, M. S. H., Khalid, M. I., Sasai, H. & Takizawa, S. Two-pot synthesis of unsymmetrical hetero[7]helicenes with intriguing optical properties. *Tetrahedron* **133**, 133266 (2023).
60 Salem, M. S. H., Sabri, A., Khalid, M. I., Sasai, H. & Takizawa, S. Two-step synthesis, structure, and optical features of a double hetero[7]helicene. *Molecules* **27**, 9068 (2022).

61 Schleyer, P. v. R., Maerker, C., Dransfeld, A., Jiao, H. & van Eikema Hommes, N. J. Nucleus-independent chemical shifts: a simple and efficient aromaticity probe. *J. Am. Chem. Soc.* **118**, 6317–6318 (1996).
62 Chen, Z., Wannere, C. S., Corminboeuf, C., Puchta, R. & Schleyer, P. v. R. Nucleus-independent chemical shifts (NICS) as an aromaticity criterion. *Chem. Rev.* **105**, 3842–3888 (2005).
63 McLean, A. & Chandler, G. Contracted Gaussian basis sets for molecular calculations. I. Second row atoms, Z = 11–18. *J. Chem. Phys.* **72**, 5639–5648 (1980).
64 Wang, Z. py. Aroma: An Intuitive Graphical User Interface for Diverse Aromaticity Analyses. *Chemistry* **6**, 1692–1703 (2024).
65 Geuenich, D., Hess, K., Köhler, F. & Herges, R. Anisotropy of the induced current density (ACID), a general method to quantify and visualize electronic delocalization. *Chem. Rev.* **105**, 3758–3772 (2005).
66 Casida, M. E. & Huix-Rotllant, M. Progress in time-dependent density-functional theory. *Annu. Rev. Phys. Chem.* **63**, 287–323 (2012).
67 Lu, T. & Chen, F. Multiwfn: A multifunctional wavefunction analyzer. *J. Comput. Chem.* **33**, 580–592 (2012).
68 Humphrey, W., Dalke, A. & Schulten, K. VMD: visual molecular dynamics. *J. Mol. Graph.* **14**, 33–38 (1996).
69 Maeda, C., Nomoto, S., Akiyama, K., Tanaka, T. & Ema, T. Facile synthesis of azahelicenes and diaza[8]circulenes through the intramolecular Scholl reaction. *Chem. Eur. J.* **27**, 15699–15705 (2021).
70 Maeda, C., Akiyama, K. & Ema, T. Synthesis and photophysical properties of dihetero[8]circulenes. *Org. Lett.* **25**, 3932–3935 (2023).
71 Brock-Nannestad, T. *et al.* Tetra-*tert*-butyltetraoxa[8]circulene and its unusual aggregation behaviour. *Eur. J. Org. Chem.* **2011**, 6320–6325 (2011).
72 Chen, F. *et al.* Synthesis of a tetrabenzotetraaza[8]circulene by a “Fold-In” oxidative fusion reaction. *Angew. Chem. Int. Ed.* **54**, 10639–10642 (2015).
73 Petrov, N. G., Chartier, P., Maris, T. & Wuest, J. D. Designing tetraoxa[8]circulenes to serve as hosts and sensors. *J. Am. Chem. Soc.* **144**, 556–572 (2022).
74 Hensel, T. *et al.* Diazadioxa[8]circulenes: Planar antiaromatic cyclooctatetraenes. *Chem. Eur. J.* **19**, 17097–17102 (2013).
75 Matsuo, Y., Tanaka, T. & Osuka, A. Highly stable radical cations of *N*, *N*′-diarylated tetrabenzotetraaza[8]circulene. *Chem. Eur. J.* **26**, 8144–8152 (2020).
76 Matsuo, Y., Chen, F., Kise, K., Tanaka, T. & Osuka, A. Facile synthesis of fluorescent hetero[8]circulene analogues with tunable solubilities and optical properties. *Chem. Sci.* **10**, 11006–11012 (2019).
77 Fujimoto, T., Suizu, R., Yoshikawa, H. & Awaga, K. Molecular, crystal, and thin-film structures of octathio[8]circulene: Release of antiaromatic molecular distortion and lamellar structure of self-assembling thin films. *Chem. Eur. J.* **14**, 6053–6056 (2008).
78 Chernichenko, K. Y., Sumerin, V. V., Shpanchenko, R. V., Balenkova, E. S. & Nenajdenko, V. G. “Sulflower”: A new form of carbon sulfide. *Angew. Chem. Int. Ed.* **45**, 7367–7370 (2006).
79 Serizawa, Y. *et al.* Synthesis of tetrasilatetrathia[8]circulenes by a fourfold intramolecular dehydrogenative silylation of C−H bonds. *Chem. Eur. J.* **23**, 6948–6952 (2017).
80 Pedersen, S. K. *et al.* A fully conjugated planar heterocyclic[9]circulene. *J. Am. Chem. Soc.* **142**, 14058–14063 (2020).
81 Matsuo, Y., Kise, K., Morimoto, Y., Osuka, A. & Tanaka, T. Fold-in Synthesis of a pentabenzopentaaza[10]circulene. *Angew. Chem. Int. Ed.* **61**, e202116789 (2022).

82 Yang, D. *et al.* Synthesis, structures and properties of trioxa[9]circulene and diepoxycyclononatrinaphthalene. *Angew. Chem. Int. Ed.* **63**, e202402756 (2024).
